# Electron-Poor Butenolides: The Missing Link between Acrylates and Maleic Anhydride in Radical Polymerization

**DOI:** 10.1021/jacs.3c04314

**Published:** 2023-07-27

**Authors:** Mathieu L. Lepage, Georgios Alachouzos, Johannes G. H. Hermens, Niels Elders, Keimpe J. van den Berg, Ben L. Feringa

**Affiliations:** † Stratingh Institute for Chemistry, Advanced Research Center Chemical Building Blocks Consortium (ARC CBBC), University of Groningen, Nijenborgh 4, 9747 AG Groningen, The Netherlands; ‡ Department Resin Technology, Akzo Nobel Car Refinishes BV, Rijksstraatweg 31, 2171 AJ Sassenheim, The Netherlands

## Abstract

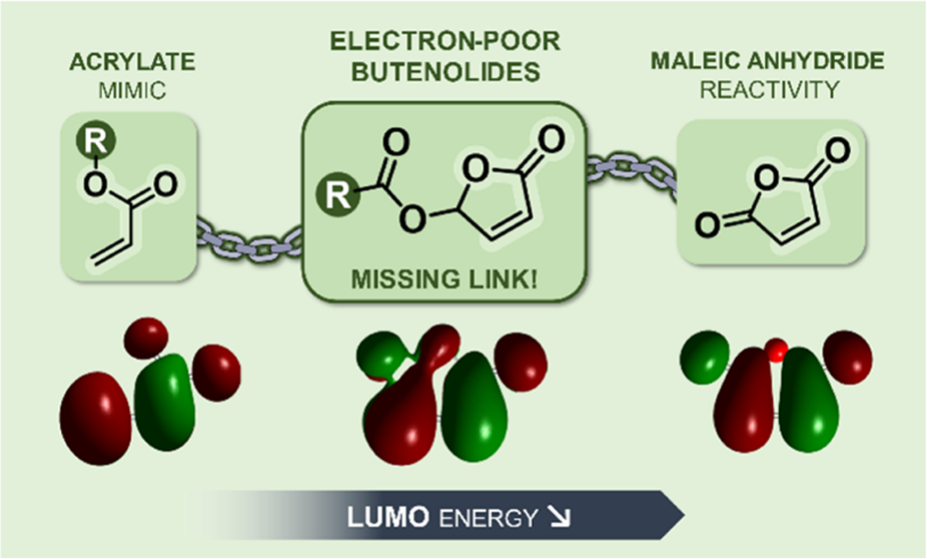

Butenolides are a class of 5-membered lactones that hold great potential as bio-based monomers to replace oil-derived acrylates, of which they are cyclic analogues. Despite this structural resemblance, the reactivity of the unsaturated ester moiety of electron-poor butenolides leans toward that of maleic anhydride, another essential monomer that does not homopolymerize but copolymerizes in a highly alternating fashion with polarized electron-rich comonomers. By studying the reactivity of 5-methoxy and 5-acyloxy butenolides through a combination of kinetics and density functional theory (DFT) experiments, we explain why electron-poor butenolides constitute a missing link between acrylates and maleic anhydride in radical polymerization.

## Introduction

**Figure 1:**
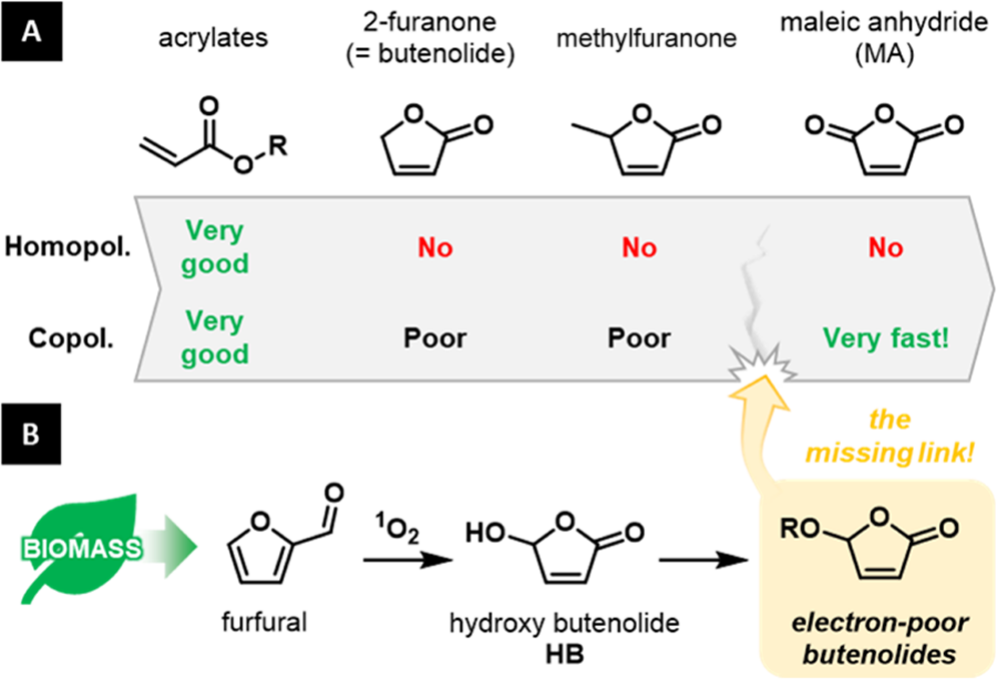
(A) General scheme showing the change of reactivity between acrylates, butenolides, and maleic anhydride. (B) Electron-poor butenolides can be prepared from bio-based furfural *via* hydroxy butenolide (**HB**), and they are the missing link between acrylate and maleic anhydride.

Acrylates and maleic anhydride (MA) are monomers of particular interest for many polymer applications in everyday materials. The former class has found ubiquitous use in plastics, paints, coatings, or adhesives,^1^ whereas the latter (together with its hydrolyzed forms) is a versatile substance used as a comonomer (*e.g.*, for unsaturated polyester resins), as a curing agent (*e.g.*, in epoxy resins), or as a food additive.^2^ Both acrylates and MA share an α,β-unsaturated ester functionality as the core feature of their structure (Figure 1A). Nonetheless, a key difference is their behavior regarding conventional free radical polymerization, a favorite methodology among industrial processes:^3^ acrylates readily homopolymerize while maleic anhydride does not. Interestingly, MA does copolymerize very well (and generally very fast) with other vinyl comonomers such as styrene or vinyl ethers to form alternating copolymers.^4^

α,β-Butenolides (furan-2(5*H*)-ones) are a class of 5-membered rings featuring an endocyclic acrylate moiety, which have been and still are intensively used in the synthesis of natural products.^5^ Recently, they are also attracting attention in the ongoing energy and feedstock transitions, for instance, as biofuel, building blocks, or potential monomers.^6^ Indeed, many of such oxygenated 5-membered rings can be obtained from biomass on a large scale *via* the conversion of nonedible lignocellulose.^7^ In particular, butenolides derived from furfural^6a,8^ or hydroxymethylfurfural (*via* levulinic acid)^6e^ are finding increasing applications. Unfortunately, the unsubstituted butenolide (furan-2(*5H*)-one or simply furanone)^9^ or its 5-methyl derivative (β-angelica lactone)^6e^ are unreactive toward radical polymerization (Figure 1A).

**Figure 2:**
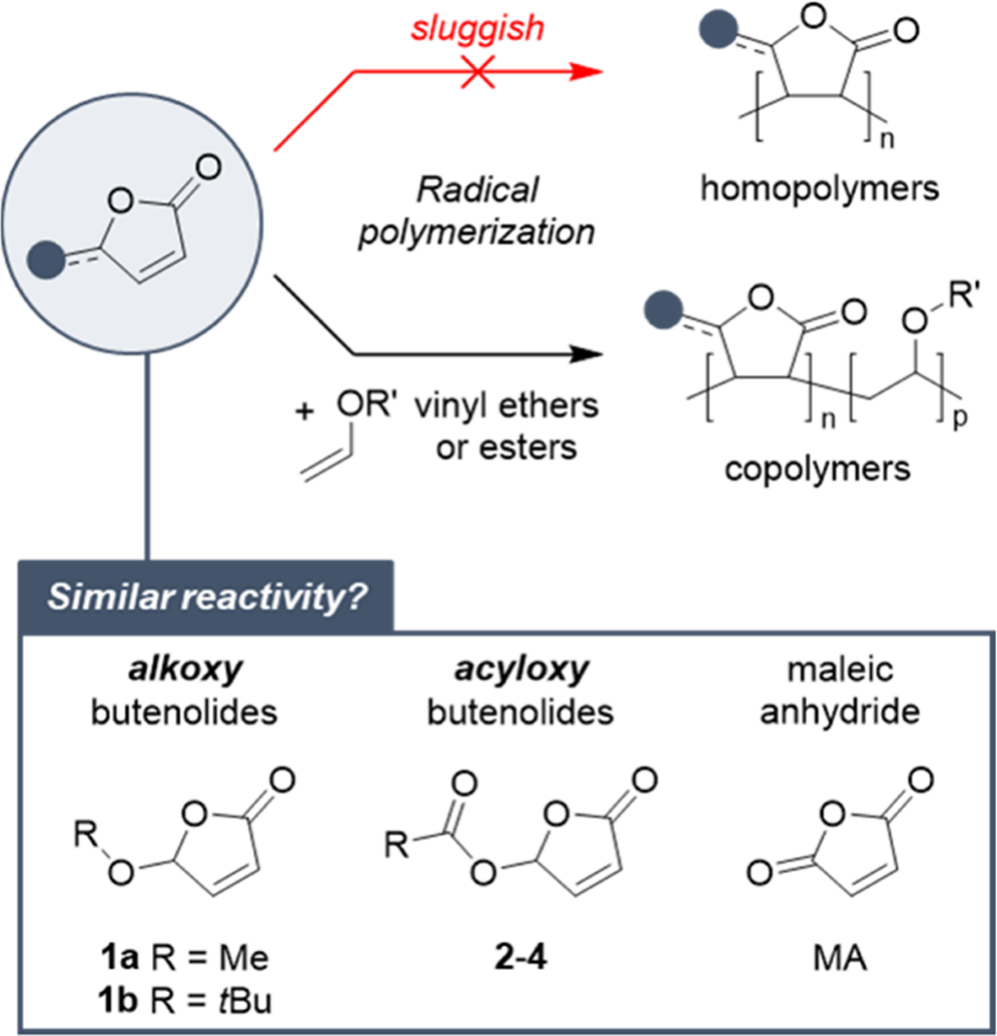
Polymerization reactivity of 5-alkoxy butenolides (**1**) and the new class of 5-acyloxy butenolides (**2**–**4**) is reminiscent of that of maleic anhydride.

We have recently introduced 5-alkoxy butenolides **1** as a very versatile bio-based alternative for acrylates in coating compositions (Figure 2).^6a−6d,10^ These acetals are readily prepared from the condensation of alcohols onto hydroxy butenolide **HB**, itself obtained *via* highly selective and quantitative photooxygenation of the platform chemical furfural (Figure 1B).^11^ In our prior work, it was shown that substitution with an electron-withdrawing alkoxy substituent at the 5-position allows for smooth copolymerization of this butenolide subclass with vinyl ethers and esters, affording polymers and coatings with a high content of bio-based carbon in excellent yields. However, the radical homopolymerization of 5-alkoxy butenolides **1** was sluggish even at 120 °C, affording mere oligomers (Figure 2) with poor conversion.

It struck us how reminiscent this reactivity was of that of maleic anhydride and stimulated us to further study the relationship between acrylates, maleic anhydride, and butenolides. Therefore, we report here the new subclass of 5-acyloxy butenolides **2**–**4** (Figure 2), whose fine-tuned electronics enable an enhanced reactivity compared to alkoxy derivatives **1**, ever more similar to that of MA. After evaluating their polymerization properties, we used a combination of kinetics experiments and density functional theory (DFT) calculations to show that electron-poor 5-oxybutenolides, in general, may be viewed as a “missing link” connecting the reactivity of the acrylate motif with that of MA (Figure 1B).

## Results and Discussion

**Figure 3:**
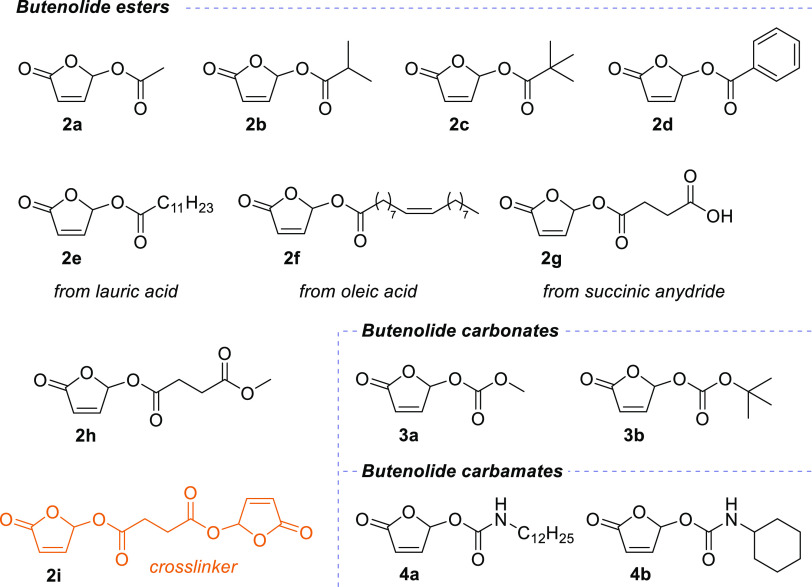
5-Acyloxy butenolide monomers.

5-Acyloxy butenolides are readily synthesized in one step from the parent hydroxy butenolide **HB**, itself obtainable at a 100 gram-scale *via* continuous photooxygenation of furfural using our recently introduced photoflow reactor.^11^ Acylation of this cyclic hemiacetal with anhydrides^12^ or acyl chlorides^12b,13^ afforded esters **2** and carbonates **3**, whereas we found that the tin-catalyzed reaction with isocyanates produces carbamates **4** in high yields (Figure 3). For the preparation of esters, Steglich esterification^14^ also proved to be highly suitable. Using bio-based carboxylic acids like acetic, lauric, or oleic acid, this mild procedure yields butenolides (**2a, 2e, 2f**) virtually made of 100% bio-based carbon. Furthermore, the ring-opening of succinic anhydride, an isomer of **HB**, affords the carboxylic acid-functionalized derivative **2g**, which can be further transformed into its methyl ester **2h** or the difunctional compound **2i**. The free carboxylic acid **2g** itself proved unstable at high temperatures (120 °C), where it reverted to its components (**HB** and succinic anhydride, see the Supporting Information). Moc (methoxycarbonyl)- and Boc-functionalized butenolides **3a**–**3b**, as well as carbamates **4a**–**4b**, were conceived to modulate the reactivity of the butenolide core and/or the properties of the corresponding polymers (*e.g.*, through hydrogen bonding).

To assess the effect of the 5-acyloxy substituents on the reactivity of the butenolides’ double bond, we compared their initial copolymerization rate when mixed with an equimolar amount of dodecyl vinyl ether (DVE). Under our typical conditions using a peroxide as a radical initiator (Trigonox 42S) activated at 120 °C, acetoxy butenolide **2a** proved significantly more reactive than methoxy butenolide **1a** (Table 1, entries 1 and 3, with *p* < 0.01 in Student’s *t*-test), with an initial pseudo-first-order rate constant more than twice as high. In general, most acyloxy butenolides **2**–**4** copolymerized faster than alkoxy butenolides **1a** or **1b**, with the exception of oleic acid-derived butenolide **2f** (Table 1). We attributed this deviation to a combination of dilution (increased amount of unreactive mass) and decreased polarity of the reaction medium (see the Supporting Information). Of note, the internal double bond was mostly preserved even after 2 h at 120 °C (as seen by ^1^H NMR), meaning that it was unreactive in the radical polymerization (a minor side-reaction seems to increase the polydispersity index (PDI), probably *via* abstraction of allylic hydrogens, see the Supporting Information). When bis-butenolide **2i** was used, the expected cross-linking of the product occurred within 3 min only, forming an insoluble gel that prevented sampling and accurate measurement of the initial kinetic rate, which was nonetheless evidently high. This observation augurs well for the usage of **2i** as a cross-linker for coating compositions.^6a,6c,6d^ It is also noteworthy that the steric bulk of the 5-substituent does not impede copolymerization, as evidenced by the rates observed for butenolides **1a**–**b** and **2a**–**c** (Table 1).

**Table 1:**
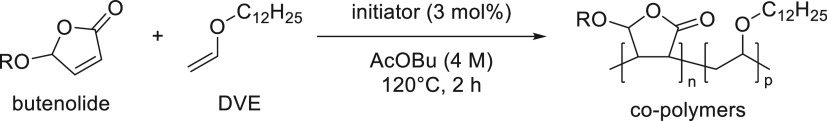
Comparison of Initial Copolymerization Rates and Molecular Weight Distribution for Equimolar Mixtures of Butenolide and Dodecyl Vinyl Ether (DVE) in *n*-Butyl Acetate (AcOBu) at 120 °C, Initiated by the Addition of Trigonox 42S (3 mol % *vs* Total Monomer Content).^a^

butenolide	*k*_ini_ [10^–3^ s^–1^]	*M*_n_ [kDa]	PDI	DP	*T*_g_ (°C)
**1a**	0.79 ± 0.15^b^	2.3	1.8	14	–4
**1b**	1.04	2.1	1.7	11	–14
**2a**	2.13 ± 0.11^b^	4.0	2.5	22	30
**2b**	2.55	2.2	2.6	11	6
**2c**	2.59	4.1	2.5	20	21
**2d**	3.68	4.3	2.9	21	34
**2e**	2.66	4.5	3.1	18	10
**2f**	0.77	5.1	6.6	18	–4
**2h**	3.25	4.3	3.1	20	13
**2i**	gelation time = 3 min				
**3a**	2.35	4.1	2.4	21	41
**3b**	1.75	3.3	2.1	15	18
**4a**	1.49	3.8	2.8	14	58
**4b**	1.38	3.8	2.3	17	60

a *k*_ini_ = initial observed pseudo-first-order copolymerization rate; *M*_n_ = number-average molecular weight; PDI = polydispersity index; DP = n + p, average degree of polymerization; *T*_g_ = glass transition temperature.

b Average and standard deviation were calculated from three replicates.

The generally increased rate of copolymerization correlates well with increased molecular weight (Table 1): the degree of polymerization (DP) is fairly constant at around 20 units, which is higher than for **1a**–**b** and perfectly well-suited for usage in high solids, two-component coating technology.^15^ The glass transition temperature (*T*_g_) for each copolymer follows a logical trend where more rigid butenolides (such as **2a** and **2d**) have a higher *T*_g_ compared to more flexible ones (such as **2e**, **2f**, **2h**). One notable exception to these patterns is observed in the case of isobutyroxy butenolide **2b**, for which the DP is about half as for the other derivatives and the *T*_g_ is markedly low. We attributed this to the weak tertiary C–H bond of the isobutyric moiety, which may facilitate chain termination by hydrogen atom transfer (HAT) and thus the formation of shorter polymer chains.

The copolymerization behavior of carbonate **3a** appeared really similar to that of ester **2a**, whereas Boc-functionalized derivative **3b** suffered from a slightly reduced rate compared to the bulky ester counterpart **2c**. This was also reflected in the slightly lower degree of polymerization. Nonetheless, the installation of an acid-labile Boc group in **3b** will certainly be of interest for the post-functionalization of the obtained copolymer *via* the masked acetal moiety. Both carbamate butenolides **4a**–**4b** also exhibited enhanced reactivity compared with methoxy butenolide **1a**, although to a lesser extent. The higher amount of hydrogen-bond donors and acceptors in carbonate **3a** and carbamates **4a**–**4b** translated into increased glass transition temperature (*T*_g_).

Altogether, acyloxy butenolides **2**–**4** generally show an increased reactivity and form copolymers with higher molecular weights compared to alkoxy butenolides **1**. Furthermore, the polymer properties can be finely tuned by varying the side-chain without impeding polymerization performance: the polarity and the glass transition temperature can be easily adjusted, as well as the content in hydrogen-bond donors and acceptors.

**Figure 4:**
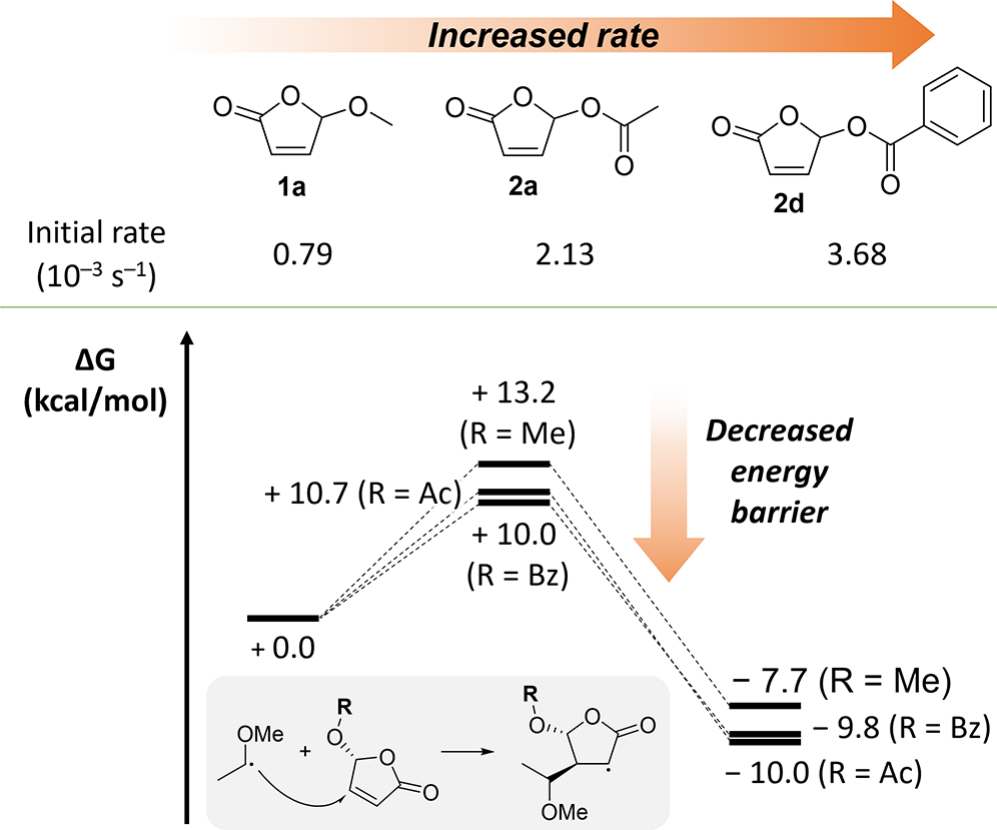
DFT calculations (at the MN15/def2tzvpp/SMD=THF level) show that the energy barrier for the addition of the radical derived from methyl vinyl ether (MVE) on butenolides is lower with an electron-withdrawing acyloxy substituent (**2a**,**d**) than with a methoxy group (**1a**). Of note, the exothermicity is also higher.

The differences observed in the rate of copolymerization seem to correlate to the electron-withdrawing character of the acetal substituent, and we performed DFT calculations to support these observations (at the MN15/def2tzvpp/SMD=THF level of theory).^16^ Considering an alternating radical copolymerization mechanism,^4b,17^ we were happy to find that the addition of the methyl vinyl ether (MVE) radical to a butenolide has a lower activation barrier with acetyl and benzoyl substituents than with a methoxy group and that there is a higher gain in energy as well (Figure 4).

**Figure 5:**
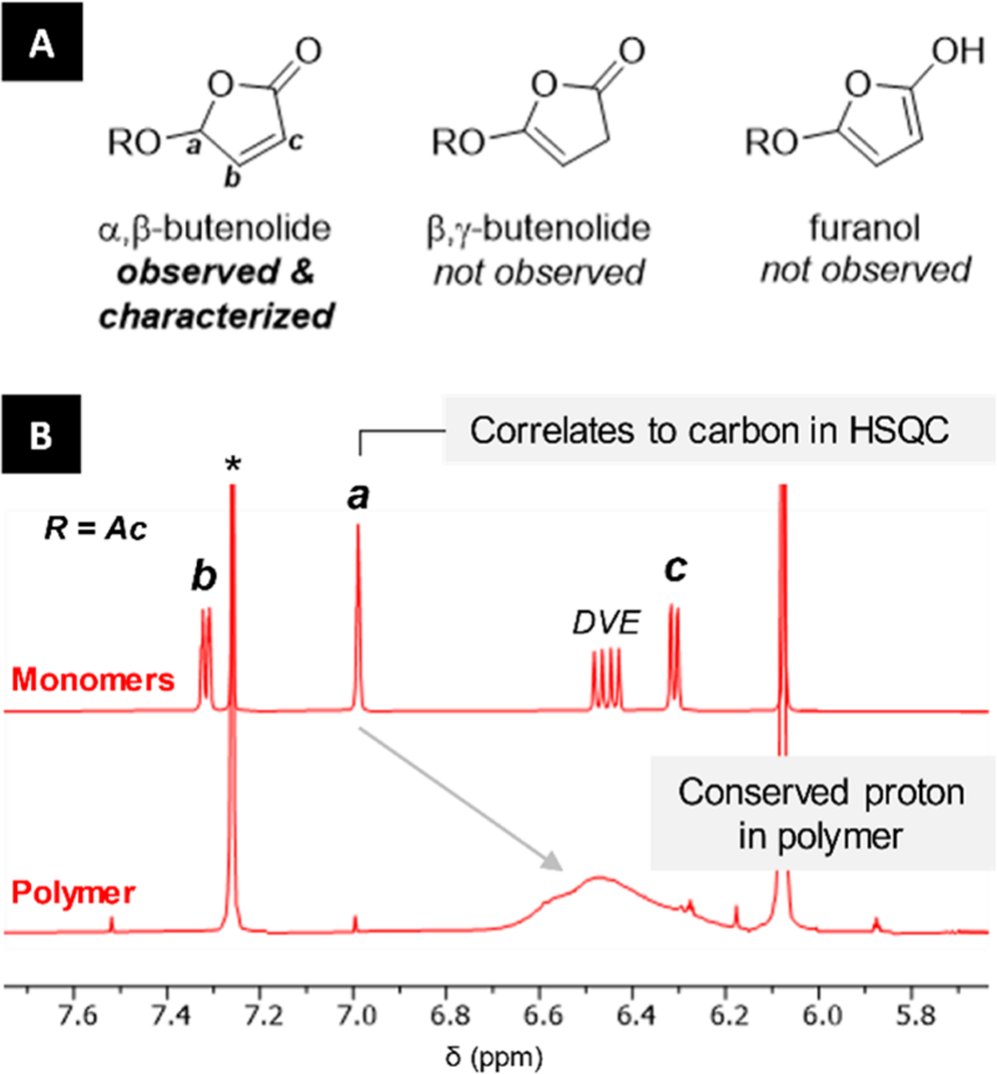
(A) Studied α,β-butenolide class, along with its isomers, which were not observed at any moment during polymerization. (B) Stacked spectra of **2a** and DVE (top) compared to the resulting poly(**2a**-*co*-DVE) copolymer (2 h reaction, bottom) show that the acetal proton is conserved during the reaction. Resonances noted **b** and **c**, as well as that for the DVE proton, are associated with the vinyl moieties and thus disappear during polymerization. The * symbol indicates the residual solvent peak in CDCl_3_.

Given this correlation between the reactivity and electron-withdrawing character of the 5-substituent, it is legitimate to wonder about the participation of isomers of α,β-butenolides, namely, the β,γ-butenolide or furanol forms, which could arise from a facilitated deprotonation at the acetal position (noted **a** in Figure 5). The involvement of those species was ruled out because no isomerization of any of the butenolides was observed (see Figure 5 and the Supporting Information). In our copolymerization experiments, as the resonance for the acetal proton (noted **a**) of the monomer disappears, a broad resonance rises with a 0.5 ppm upfield shift, corresponding to that same acetal proton now adjacent to an sp^3^-hybridized carbon center in the polymer. The aromaticity of the furanol would rather prevent polymerization and thus decrease the observed rate, which conflicts with the experimental data (*vide supra*). Finally, we calculated that the furanol form is disfavored by >12 kcal/mol compared to the α,β-butenolide form (R = H, Me, Ac, see the Supporting Information), hence our confidence that only the latter is involved.

Considering the structural and reactive kinship of 5-oxybutenolides with MA, whose copolymerization with polarized vinyl comonomers and inability to homopolymerize in conventional radical conditions are both well-documented,^4,18^ we envisioned that 5-acyloxy butenolides **2**–**4** would exhibit similar behaviors. Indeed, our model acetoxy butenolide **2a** only produces very short homo-oligomers (DP < 5, see the Supporting Information) when used as a single monomer, leaving most of the butenolide unreacted,^19^ whereas copolymerization with the DVE monomer proceeds quickly to completion (Table 2, entries 1 and 2) to afford polymers with DP > 20.

**Table 2:**
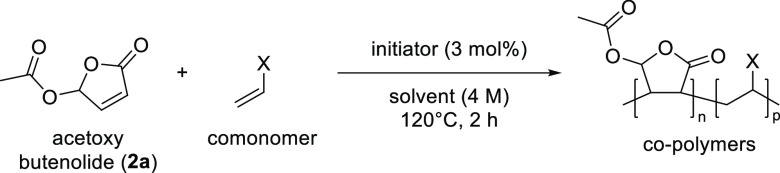

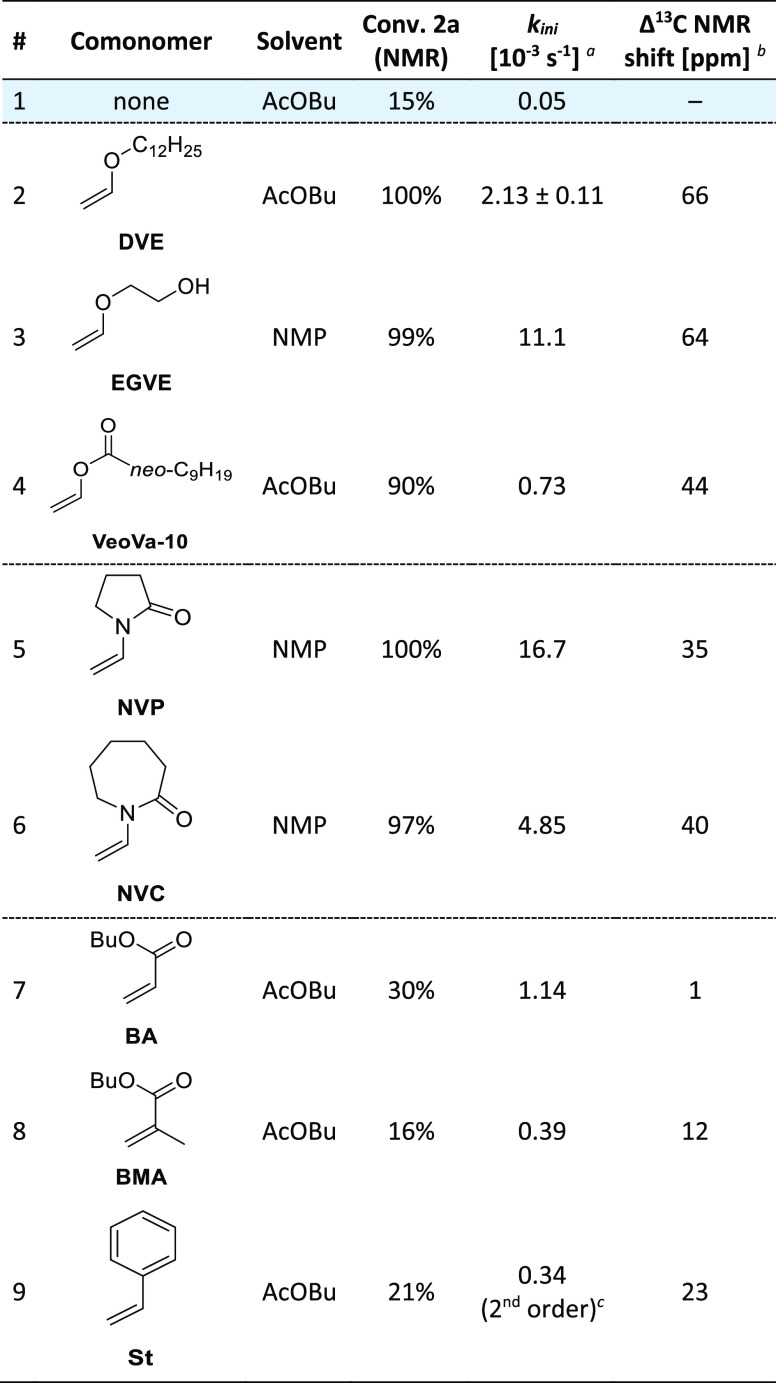
Comparison of Butenolide Conversion and Initial Copolymerization Rates for Equimolar Mixtures of Acetoxy Butenolide and Various Comonomers at 120 °C, Initiated by the Addition of Trigonox 42S (3 mol % *vs* Total Monomer Content); the Solvent was Adjusted for Solubility Purposes.

a *k*_ini_ = initial observed pseudo-first-order copolymerization rate.

b Δ^13^C NMR shift = difference in the ^13^C chemical shift between the two alkene carbons of the comonomer; see the Supporting Information.

c Pseudo-second-order rate was a better fit to the data.

When replacing DVE with the shorter and more hydrophilic ethylene glycol vinyl ether (EGVE), a 5-fold increase of the initial kinetic rate (entry 3 *vs* entry 2) is observed, which we propose is due to the increased polarity of the reaction medium: we used the more polar solvent *N*-methyl pyrrolidone (NMP) for solubility reasons, and this inherently increases the rates (see the Supporting Information). Conversely, the replacement of DVE by slightly less polarized vinyl ester VeoVa-10 (see the Δ^13^C NMR shift in Table 2) induced a reduction of the copolymerization rate (entry 4). In sharp contrast, we observe high rates and conversions with *N*-vinyl lactams such as *N*-vinyl pyrrolidone (NVP, entry 5) and *N*-vinyl caprolactam (NVC, entry 6). This acceleration is particularly striking with NVP, a comonomer of relevance since the corresponding polymer (povidone) is a versatile substance used in a wide variety of applications, including pharmaceutical materials.^20^ On the other hand and similarly to previous observations with alkoxy butenolides,^6a^ copolymerization of acyloxy butenolides with (meth)acrylates or styrene does not proceed well (entries 7–9); whereas the comonomer is consumed within 2 h, in all cases less than 30% of the butenolide monomer is incorporated (see the Supporting Information). Considering the ability of those comonomers to homopolymerize, this indicates a mismatch in reactivity. Altogether, this confirms that butenolides preferably copolymerize with polarized comonomers (*i.e.*, monomers with a large Δ^13^C NMR shift across the alkene moiety, see Table 2). This is a feature they share with MA, the radical copolymerization of which is highly alternating.^4b,4d^ One can easily imagine that the closely related butenolides may exhibit similar alternating behavior.

**Figure 6:**
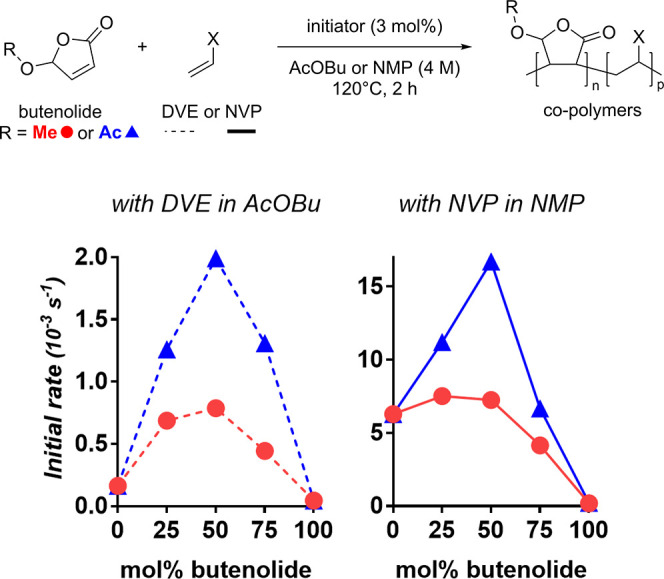
Initial (co-)polymerization rates for mixtures of butenolides (**1a** or **2a**) with DVE or NVP in various ratios, showing an acceleration for copolymers *versus* homopolymers. This acceleration is particularly marked for equimolar compositions (50 mol % butenolide). Values for polymers with **1a** are indicated with red dots, whereas those for polymers with **2a** are indicated with blue triangles. Polymers with DVE (left graph with dotted lines) were prepared in butyl acetate, whereas polymers with NVP (right graph, solid lines) were prepared in *N*-methyl pyrrolidone (NMP) for solubility reasons. See tabulated values in the Supporting Information, along with further details.

Indeed, 5-oxybutenolides show kinetics and conversions typical of alternating copolymerizations when combined with a vinyl ether or a vinyl lactam (Figure 6 and the Supporting Information).^21^ We compared the initial rates of copolymerization for various mixtures of our methoxy (**1a**) and acetoxy (**2a**) derivatives with DVE or NVP. When combined with DVE, both butenolides exhibit a maximum rate for equimolar mixtures (Figure 6, left graph). This observed behavior for mixtures of non-homopolymerizable monomers strongly supports an alternating addition mechanism.^4b^ A similar observation can be made when combining acetoxy butenolide **2a** with NVP (blue curve in Figure 6, right graph), and we find this a critical result given that NVP is capable of homopolymerization. On the contrary, for mixtures of methoxy butenolide **1a** and NVP, the maximum rate is measured for more NVP-rich mixtures (red curve in Figure 6, right graph). This suggests that the homopolymerization of NVP competes with its copolymerization with **1a**.

**Figure 7:**
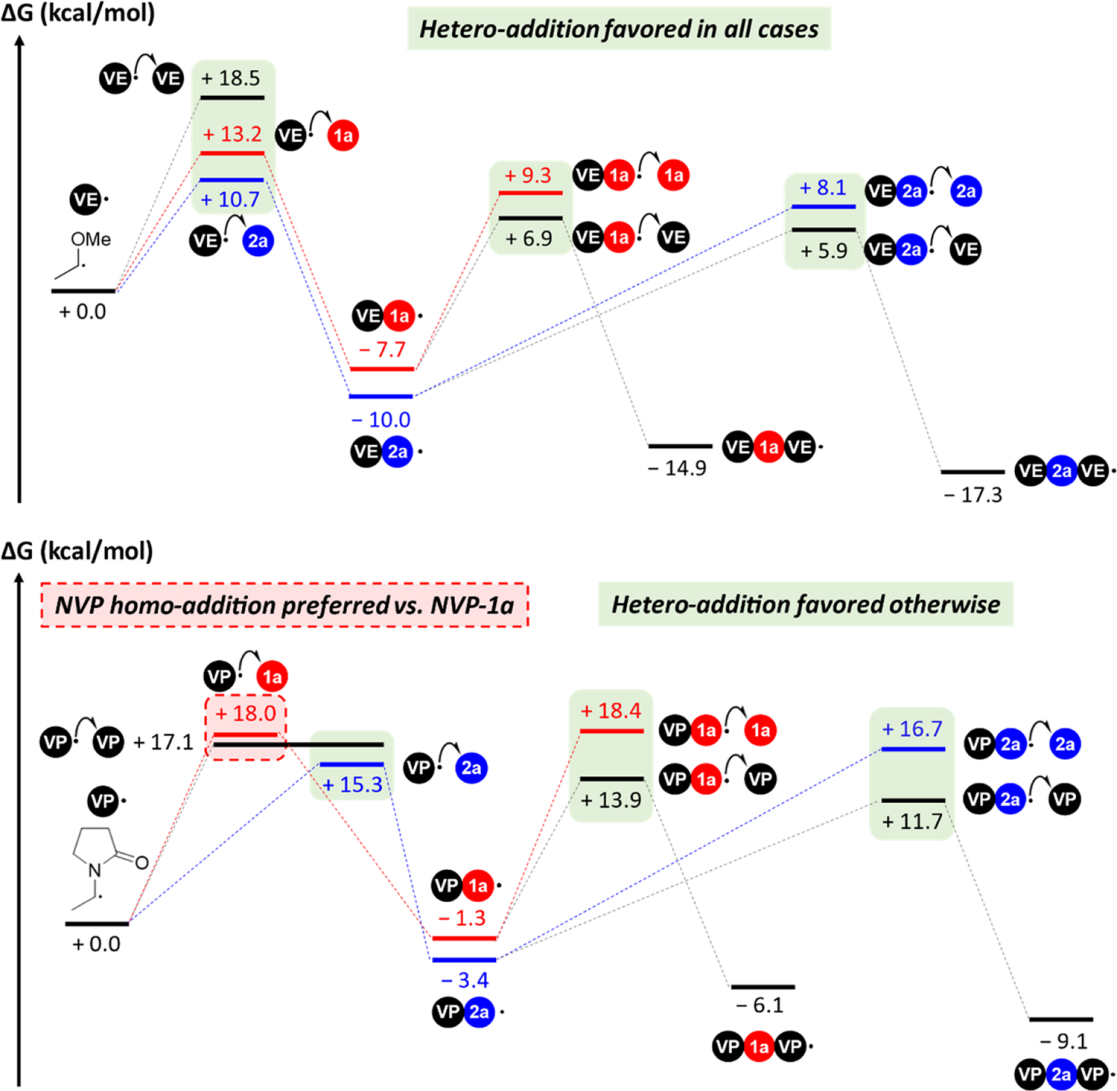
Energy diagrams at the MN15/def2tzvpp/SMD=THF level of theory for the successive addition of relevant radicals to **1a**, **2a**, methyl vinyl ether (VE), or *N*-vinyl pyrrolidone (VP), generally showing a preferred addition of butenolide radicals to comonomers, and vice-versa (the only exception being the addition of the VP radical to **1a**, see red dashed boxes). See tabulated values in the Supporting Information, along with further details.

The agreement of these observations with our DFT thermochemistry calculations is remarkable. We calculated the energy barrier for the addition of MVE- or NVP-based radicals to their parent monomers or to butenolides (**1a** or **2a**) and repeated the operation for the subsequent addition of the resulting radicals (Figure 7 at the MN15/def2tzvpp/SMD=THF level of theory).^17^ In nearly all cases, homoaddition is disfavored by 1.8–7.8 kcal/mol compared to heteroaddition, altogether supporting an alternating copolymerization mechanism. The only exception to this trend was the homoaddition NVP–NVP being slightly favored (by 0.9 kcal/mol) compared to the heteroaddition of an NVP radical to **1a**. This is in excellent agreement with the experimental results described above (Figure 6) and strengthens our rationale that homopolymerization of NVP competes with copolymerization only when combined with less-reactive alkoxy butenolide **1a**. Of course, at the considered temperature (120 °C), other minor side processes are likely concomitant, but our analysis provides a solid explanation for the experimentally observed alternating copolymerization of butenolides with polarized comonomers. Once again, this behavior is highly reminiscent of the reactivity of MA.^4b,4c^

**Figure 8:**
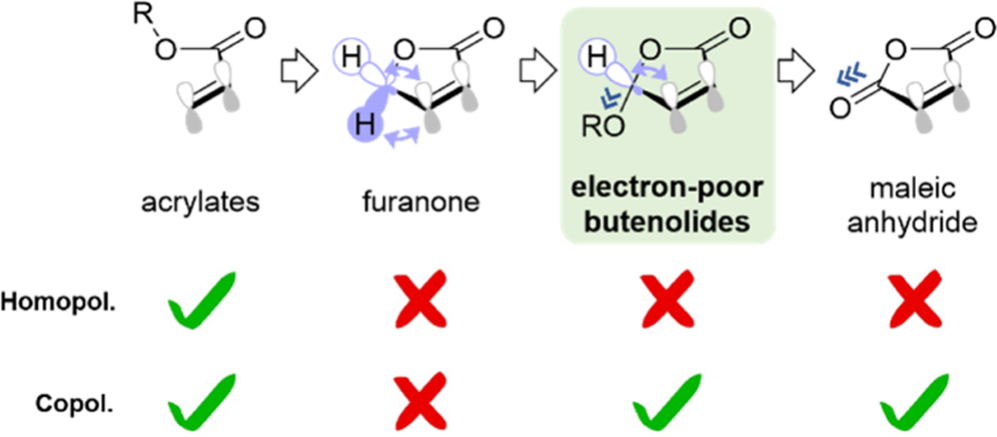
Electron-poor butenolides are the missing link connecting the acrylate motif with the reactivity of maleic anhydride.

As mentioned in the introduction, the unsubstituted furanone or its 5-methyl derivative performs poorly in radical polymerization, despite being cyclic versions of methyl and ethyl acrylates, respectively. Yet, when adjusting the electronic density on butenolides through modification of the 5-position, one can switch their reactivity toward that of MA, and the more electron-poor the butenolide, the more so (*vide supra*). It struck us that such a dramatic shift of reactivity occurs within this closely related family of species, and we hypothesized that electron-poor butenolides constitute a missing link connecting these opposite reactivities (Figure 8).

We envisioned that the σ_C–H_ bond(s) in position 5 may interact with the π system of the acrylate moiety through hyperconjugation (Figure 8).^22^ Such participation was previously invoked by Kayser *et al.* to explain unexpected regioselectivities in the reductions of substituted maleic anhydrides.^23^ In furanone, the dramatically reduced capacity for radical homopolymerization would be caused by the two σ_C–H_ bonds both contributing to this effect. The same reasoning would hold for 5-methylfuranone, although in that case, with one σ_C–H_ and one σ_C–C_. Moving to more electron-poor butenolides would effectively decrease the electron density of the σ_C–H_ bond, thereby indirectly influencing the alkene moiety by hyperconjugation. This qualitative analysis does not include the potential influence of classical anomeric effects,^24^ which cannot be excluded yet seems less relevant.

**Figure 9:**
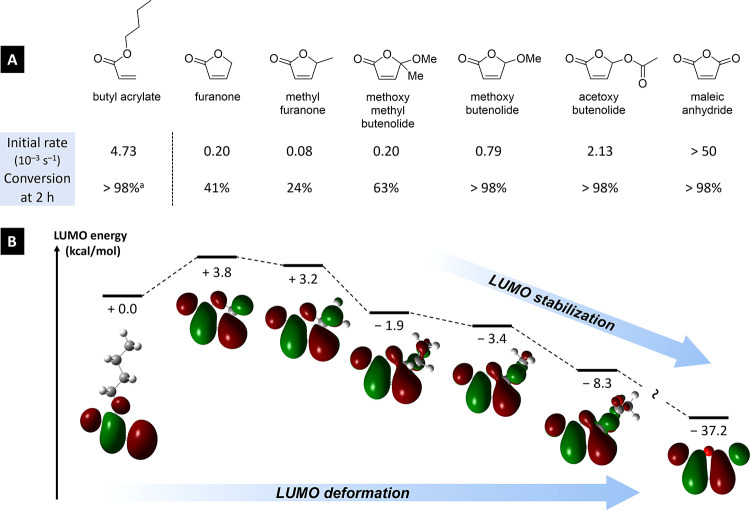
(A) Compared conversion and initial kinetic rates of copolymerization of butyl acrylate, various butenolides, and maleic anhydride with dodecyl vinyl ether (DVE). Trigonox 42S was used as an initiator. (B) Lowest unoccupied molecular orbital (LUMO) energy (at the MN15/def2tzvpp/SMD=THF level of theory) and shape (isovalue = 0.02) for select butenolides compared with butyl acrylate and maleic anhydride, showing both a deformation and a stabilization of the LUMO for more electron-poor butenolides, similarly to maleic anhydride. ^a^ >30% DVE remained at the end of the reaction.

We confirmed our initial presumption by measuring conversion and initial copolymerization rates of butyl acrylate, MA, and a series of butenolides with DVE (Figure 9A). Unsurprisingly, butyl acrylate was mostly homopolymerized, leaving a significant amount of DVE unreacted after 2 h, whereas all MA copolymerized with DVE in <1 min. Between these extremes, five butenolides spanned the reactivity landscape. As expected, furanone and 5-methylfuranone were quite unreactive, whereas methoxy (**1a**) and acetoxy butenolide (**2a**) were entirely consumed and at much higher rates. Interestingly, the doubly substituted 5-methoxy-5-methyl butenolide proved relatively unreactive. This suggested that not only an electron-withdrawing substituent but also a C–H bond at this position is critical to trigger the copolymerization reactivity.

Our DFT calculations support our hypothesis through two aspects regarding the LUMO of the studied systems (Figure 9B). First, the LUMO of furanone is destabilized by 3.8 kcal/mol compared to that of butyl acrylate. The LUMO of methylfuranone is also destabilized but to a slightly smaller extent (3.2 kcal/mol), which fits our analysis above. From there, substitution at the 5-position by electron-withdrawing groups (methoxy or acyloxy) incrementally stabilizes the LUMO and enables the copolymerization with polarized comonomers (particularly the electron-rich ones). In the extreme case of MA (conceptually a butenolide with a strongly electron-withdrawing 5-oxo substituent), the LUMO is stabilized by >40 kcal/mol *versus* furanone. The particular case of 5-methoxy-5-methyl butenolide, the LUMO of which lays “only” 1.5 kcal/mol above that of **1a**, is intriguing; the C–C bond appears less influenced by the geminal electron-withdrawing methoxy than the corresponding C–H bond in **1a**. In this case, we emphasize that steric hindrance may have a larger impact, with both faces of the enone moiety being cluttered, explaining the significant difference in reactivity in spite of relatively close LUMO energies.

Second and finally, the shape of the LUMO also undergoes a progressive deformation toward symmetrization as one spans the depicted series toward the more electron-poor compounds, with the various butenolides exhibiting a hybrid character between butyl acrylate and maleic anhydride (Figure 9B and the Supporting Information). In particular, the σ_C–H_ bond at position 5 of butenolides seems to mimic one of the lobes of MA’s LUMO. Together with the LUMO stabilization, these elements support our concept hypothesis that butenolides are a missing link between the acrylate motif and the reactivity of maleic anhydride (Figure 8).

## Conclusions

Studying the bio-based butenolide platform to replace widely used acrylate monomers, we introduced electron-poor 5-acyloxy butenolides **2**–**4**. We discovered that much faster copolymerization kinetics and an increased tendency to alternate with polarized vinyl comonomers are achieved when compared to 5-alkoxy butenolides **1**, and even more so compared to the unsubstituted furanone and methylfuranone. DFT studies complement the experimental kinetic data by identifying the energy barrier of radical additions as a meaningful predictor of relative kinetic rates and of the experimentally observed alternating copolymerization behavior. Despite the concurrent structural kinship of butenolides with acrylates and maleic anhydride, the reactivity of the electron-poor derivatives (such as 5-acyloxy butenolides) leans toward that of MA. The energy of the LUMO, lowered through hyperconjugation of an electron-depleted σ_C–H_ bond with the alkenyl π-system, appears as the main factor explaining this distinct reactivity. By extending the methodology used in this work to other systems, the evaluation of hyperconjugation might shed light on unexplained reactivity patterns in polymer chemistry. This novel subclass of electron-poor butenolides, which constitutes structural and electronic intermediates between acrylates and maleic anhydride, holds great promise for future bio-based polymers and coatings.

## Supporting Information Available

Extended discussion and accompanying figures; experimental details, materials, and methods; ^1^H and ^13^C NMR spectra for all novel compounds; data summaries for polymerization and DFT calculations; (co-)polymerization data; differential scanning calorimetry (DSC) and gel permeation chromatography (GPC) analysis of polymers; and DFT data (PDF)
