# Supplementary material for: Electron-Poor Butenolides: The Missing Link between Acrylates and Maleic Anhydride in Radical Polymerization
Source: J Am Chem Soc. 2023 Jul 27;145(31):17211–9. doi: 10.1021/jacs.3c04314 (PMC10416300; doi:10.1021/jacs.3c04314)
Supplement: Supplementary file 1 — ja3c04314_si_001.pdf [file ja3c04314_si_001.pdf]

Supporting information

Electron-poor butenolides: the missing link between acrylates and maleic anhydride in radical polymerization

Mathieu L. Lepage,[a,b,*] Georgios Alachouzos,[a] Johannes G. H. Hermens,[a] Niels Elders,[c] Keimpe J. van den Berg,[c] Ben L. Feringa[a,*]

[a] Stratingh Institute for Chemistry, Advanced Research Center Chemical Building Blocks Consortium (ARC CBBC), University of Groningen, Nijenborgh 4, 9747 AG Groningen, The Netherlands. E-mail: b.l.feringa@rug.nl

[b] Present address: CNRS / Université Paul Sabatier, Laboratoire Hétérochimie Fondamentale et Appliquée (LHFA, UMR 5069), 118 route de Narbonne, 31062 Toulouse Cedex 09, France. E-mail: mathieu.lepage@cnrs.fr

[c] Department Resin Technology, AkzoNobel Car Refinishes BV, Rijksstraatweg 31, 2171 AJ Sassenheim, The Netherlands

Extended discussion 7

Polar solvent effect on copolymerization rate 7

Degradation of succinyloxy butenolide (2g) at 120°C 8

Stability of furanols versus α,β-butenolides 9

Polarization of comonomers through Δ^{13}C NMR shift across the alkene moiety (Table 2) 9

Initial rates and final conversion for mixtures of 1a and 2a with DVE and NVP (Fig. 6) 10

HOMO and LUMO energies (Figure 9B) 11

LUMOs shape (Figure 9B) 12

General 13

Synthesis 14

Method A (with anhydrides) 14

Method B (by Steglich esterification with acids) 14

Method C (with isocyanates) 14

Tert-butoxy butenolide (1b) 15

Acetoxy butenolide (2a) 17

Isobutyroxy butenolide (2b) 19

Pivaloyloxy butenolide (2c) 21

Benzoyloxy butenolide (2d) 23

Lauryloxy butenolide (2e) 25

Oleyloxy butenolide (2f) 27

Succinyloxy butenolide (2g) 29

Succinyloxy butenolide methyl ester (2h) 31

Succinyloxy bis-butenolide (2i) 33

Methyl carbonoxy butenolide (3a) 35

Tert-butyl carbonoxy butenolide (3b) 37

Dodecyl carbamoxy butenolide (4a) 39

Cyclohexyl carbamoxy butenolide (4b) 42

Methods 45

(Co)polymerizations for conversion and kinetic rate measurement 45

Copolymerizations with DVE for molecular weight distribution and glass transition temperature 45

Reaction kinetics 45

DFT calculations 47

Data summary 48

Summary of (co)polymerization data 48

Summary of DFT results for radical propagation reactions 50
(Co)polymerization data 54
Methoxy butenolide (1a) and DVE in AcOBu – replicate 1 54
Methoxy butenolide (1a) and DVE in AcOBu – replicate 2 56
Methoxy butenolide (1a) and DVE in AcOBu – replicate 3 58
Methoxy butenolide (1a) and DVE in GVL 60
Methoxy butenolide (1a) and DVE in diethyl carbonate 62
Methoxy butenolide (1a) and DVE in 1M2P 64
Tert-butoxy butenolide (1b) and DVE in AcOBu 64
Acetoxy butenolide (2a) and DVE in AcOBu – replicate 1 66
Acetoxy butenolide (2a) and DVE in AcOBu – replicate 2 68
Acetoxy butenolide (2a) and DVE in AcOBu – replicate 3 70
Acetoxy butenolide (2a) and DVE in 1-methoxy-2-propanol 72
Isobutyroxy butenolide (2b) and DVE in AcOBu 74
Pivaloyloxy butenolide (2c) and DVE in AcOBu 76
Benzoyloxy butenolide and DVE in AcOBu 78
Benzoyloxy butenolide (2d) and DVE in 1-methoxy-2-propanol 80
Lauryloxy butenolide (2e) and DVE in AcOBu 82
Lauryloxy butenolide (2e) and DVE in 1-methoxy-2-propanol 84
Oleyloxy butenolide (2f) and DVE in AcOBu 86
Methyl succinyloxy butenolide (2h) and DVE in AcOBu 88
Methyl carbonoxy butenolide (3a) and DVE in AcOBu 90
Tert-butyl carbonoxy butenolide (3b) and DVE in AcOBu 92
Dodecyl carbamoxy butenolide (4a) and DVE in AcOBu 94
Cyclohexyl carbamoxy butenolide (4b) and DVE in AcOBu 96
Homopolymerization of DVE in AcOBu 98
Homopolymerization of NVP in NMP 100
Methoxy butenolide (1a) and DVE (1:3) in AcOBu 102
Methoxy butenolide (1a) and DVE (3:1) in AcOBu 104
Methoxy butenolide (1a) in AcOBu 106
Methoxy butenolide (1a) and NVP (1:3) in NMP 108
Methoxy butenolide (1a) and NVP (1:1) in NMP 110
Methoxy butenolide (1a) and NVP (3:1) in NMP 112
Methoxy butenolide (1a) in NMP 114
Methoxy butenolide (1a) and NVP (1:1) in 1M2P 116

Acetoxy butenolide (2a) and DVE (1:3) in AcOBu 118
Acetoxy butenolide (2a) and DVE (3:1) in AcOBu 120
Acetoxy butenolide (2a) in AcOBu 122
Acetoxy butenolide (2a) and EGVE in NMP 124
Acetoxy butenolide (2a) and NVP (1:3) in NMP 126
Acetoxy butenolide (2a) and NVP (1:1) in NMP 128
Acetoxy butenolide (2a) and NVP (3:1) in NMP 130
Acetoxy butenolide (2a) in NMP 132
Acetoxy butenolide (2a) and VeoVa-10 in AcOBu 134
Acetoxy butenolide (2a) and NVC in NMP 136
Acetoxy butenolide (2a) and butyl acrylate in AcOBu 138
Acetoxy butenolide (2a) and butyl methacrylate in AcOBu 140
Acetoxy butenolide (2a) and styrene in AcOBu 142
Butyl acrylate and DVE in AcOBu 144
Furanone and DVE in AcOBu 146
Methyl furanone and DVE in AcOBu 148
Methoxy methyl butenolide and DVE in AcOBu 150
Maleic anhydride and DVE in AcOBu 152

Differential scanning calorimetry (DSC) 154

Gel permeation chromatography (GPC) 161

Copolymers with DVE 161
Homopolymer of 2a 166

DFT data: optimized geometries, energies and XYZ Coordinates.. 167

Butyl acrylate 167
Furanone 168
Furanol 168
Methyl furanone 169
Methoxy methyl butenolide 169
Furanol 170
Hydroxy furanol 170
Methoxy butenolide 1a 171
Methoxy furanol 171
Acetoxy butenolide 2a 172
Acetoxy furanol 172
Benzoyloxy butenolide 2d 173

Maleic anhydride 173
Methyl vinyl ether (MVE) 174
Methyl vinyl ether (MVE) radical 174
MVE-MVE radical 175
Transition state for addition of MVE radical to MVE 175
N-vinyl pyrrolidone (NVP) 176
N-vinyl pyrrolidone (NVP) radical 176
NVP-NVP radical 177
Transition state for addition of NVP radical to NVP 177
MVE-1a radical 178
Transition state for addition of MVE radical to 1a 178
NVP-1a radical 179
Transition state for addition of NVP radical to 1a 179
MVE-2a radical 180
Transition state for addition of MVE radical to 2a 180
NVP-2a radical 181
Transition state for addition of NVP radical to 2a 181
MVE-2d radical 182
Transition state for addition of MVE radical to 2d 182
MVE-1a-1a radical 183
Transition state for addition of MVE-1a radical to 1a 184
MVE-1a-MVE radical 185
Transition state for addition of MVE-1a radical to MVE 185
NVP-1a-1a radical 186
Transition state for addition of NVP-1a radical to 1a 187
NVP-1a-NVP radical 188
Transition state for addition of NVP-1a radical to NVP 189
MVE-2a-2a radical 190
Transition state for addition of MVE-2a radical to 2a 191
MVE-2a-MVE radical 192
Transition state for addition of MVE-2a radical to MVE 193
NVP-2a-2a radical 194
Transition state for addition of NVP-2a radical to 2a 195
NVP-2a-NVP radical 196
Transition state for addition of NVP-2a radical to NVP 197

Extended discussion

Polar solvent effect on copolymerization rate

Copolymerizations of methoxy- (**1a**), acetoxy- (**2a**), benzoyloxy- (**2d**) and lauryloxy butenolide (**2e**) were performed in several solvents of different polarity: diethyl carbonate (Et_2CO_3), butyl acetate (AcOBu), γ-valerolactone (GVL), 1-methoxy-2-propanol (1M2P) or *N*-methyl pyrrolidone (NMP).

Initial rates were dependent on the polarity of solvent, as shown in the table below. When a rather apolar solvent was used (diethyl carbonate or AcOBu), the rate was lower than with more polar solvents like GVL or 1M2P. Note that GVL is classified as a dipolar aprotic solvent that is more polar than acyclic esters (Stolte, Kunz *et al.*, *Green Chem.* **2021**, *23*, 2962).

In the copolymerization of methoxy butenolide (**1a**) with NVP, using NMP instead of 1M2P increased the rate by more than 2-fold.

We can conclude that a more polar reaction mixture promotes a faster copolymerization.

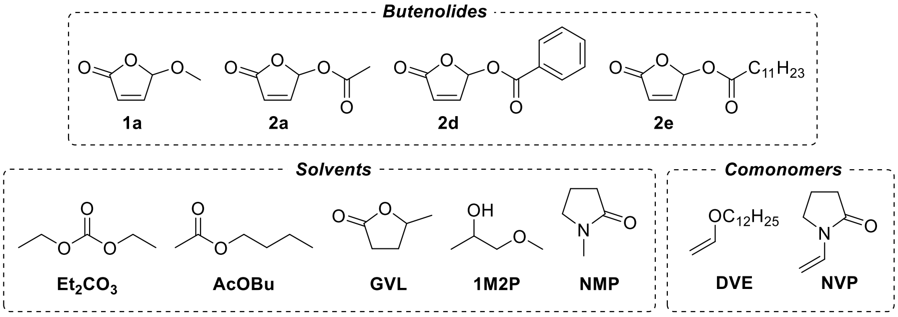

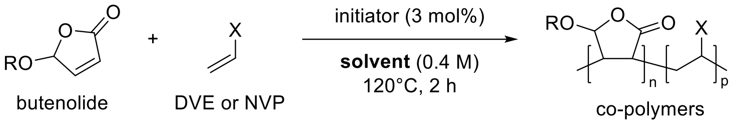

Butenolide	Comonomer	Solvent	k_{ini} [10^{-3} s^{-1}]
**1a**	DVE	Et_2CO_3	0.70
		AcOBu	0.79
		GVL	1.1
		1M2P	1.4
	NVP	1M2P	3.0
		NMP	7.2
**2a**	DVE	AcOBu	2.0
		1M2P	3.4
**2d**	DVE	AcOBu	3.7
		1M2P	4.5
**2e**	DVE	AcOBu	2.7
		1M2P	3.1

Degradation of succinyloxy butenolide (2g) at 120°C

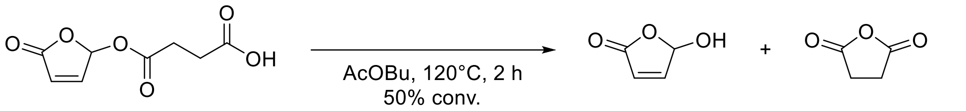

2g **HB** **succinic anhydride**

The free carboxylic acid **2g** decomposes upon heating at the temperature of the polymerization reaction. When dissolved in butyl acetate and heated in a capped vial at 120°C for 2 h, ca. 50% of the material was converted back to hydroxy butenolide (**HB**) and succinic anhydride, as visible in the NMR spectra below.

Stacked NMR spectra showing decomposition of **2g** upon heating at 120°C for 2 h in butyl acetate:

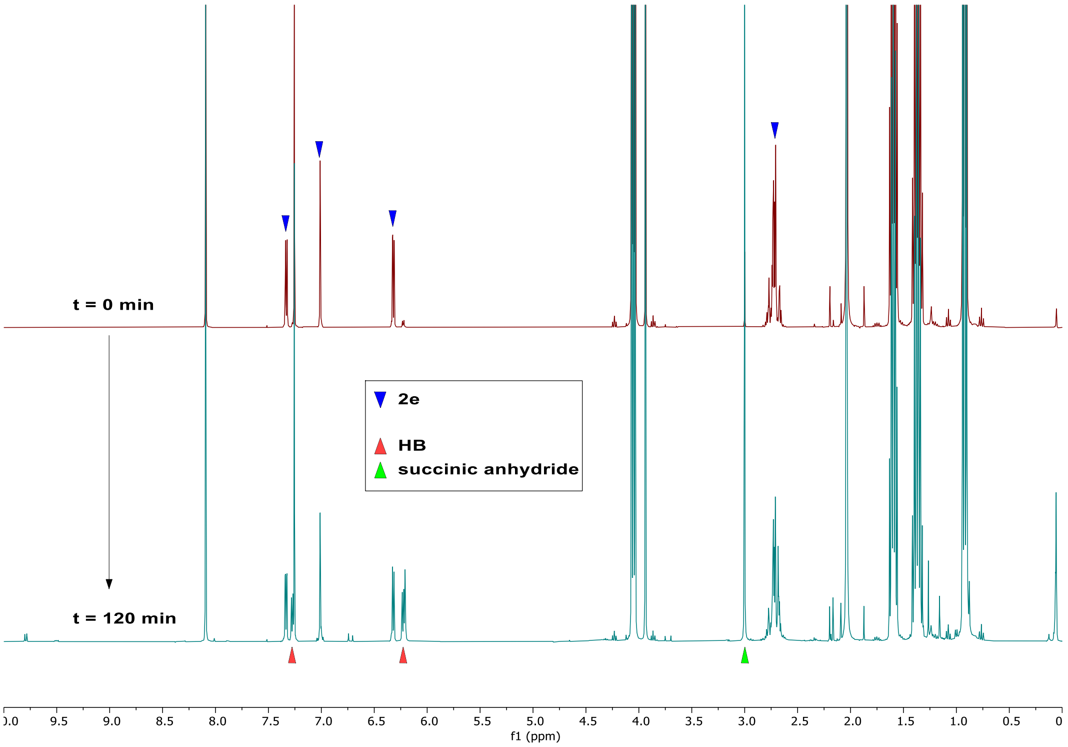

Stability of furanols versus α,β-butenolides

We computed the optimized structures and zero-point energy at the MN15/def2tzvpp/SMD=THF level of theory for a series of butenolides and their corresponding furanol forms. In all cases, the α,β-butenolide form is more stable by more than 12 kcal/mol.

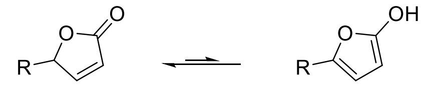

α,β-butenolide form

furanol form

R = H, OH, OMe, OAc

	R	Energy α,β-butenolide *(in Hartrees)*	Energy furanol *(in Hartrees)*	Δ *(in Hartrees)*	Δ *(in kcal/mol)*
**Furanone**	H	-305.019153	-304.999574	0.019579	**+12.3**
**HB**	OH	-380.208096	-380.187757	0.020339	**+12.8**
**1a**	OMe	-419.441938	-419.420238	0.021700	**+13.6**
**2a**	OAc	-532.727121	-532.704948	0.022173	**+13.9**

Polarization of comonomers through Δ^{13}C NMR shift across the alkene moiety (Table 2)

In Table 2 of the main text, we estimate the polarized character of the comonomers's double bond by comparing the difference in ^{13}C NMR shift between the two alkenyl carbons. Below is the table summarizing the NMR data we collated.

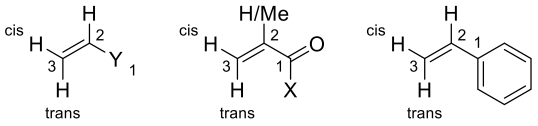

DVE
EGVE
VeoVa-10
NVP
NVC

BA
BMA

St

all data in $CDCl_3$

Compound	^{1}H NMR (ppm)						^{13}C NMR (ppm)			
	H-2	H-3$_{cis}$	H-3$_{trans}$	Δ(H2-H3$_{cis}$)	Δ(H2-H3$_{trans}$)	Δ(H3$_{cis}$-H3$_{trans}$)	C-1	C-2	C-3	**Δ(C2-C3)**
**dodecyl vinyl ether (DVE)**	6.46	3.97	4.17	2.49	2.29	-0.20	-	152.18	86.34	**65.84**
**ethylene glycol vinyl ether (EGVE)**	6.48	4.03	4.21	2.45	2.27	-0.18	-	151.66	87.23	**64.43**
**vinyl neodecanoate (VeoVa-10)**	7.26	4.55	4.87	2.71	2.39	-0.32	-	141.59	97.47	**44.12**
***N*-vinyl pyrrolidone (NVP)**	7.05	4.40	4.35	2.65	2.70	0.05	-	129.40	94.35	**35.05**
***N*-vinyl pyrrolidone (NVP)** [a]	7.08	4.45	4.42	2.63	2.66	0.03	-	129.28	94.22	**35.06**
***N*-vinyl caprolactam (NVC)** [a]	7.38	4.40	4.47	2.98	2.92	-0.07	-	132.19	92.62	**39.57**
**Butyl acrylate (BA)** [a]	6.11	5.80	6.36	0.31	-0.25	-0.57	166.19	130.21	128.92	**1.29**
**Butyl methacrylate (BMA)** [a]	-	5.53	6.09	-	-	-0.56	167.52	136.79	125.04	**11.75**
**Styrene (St)** [a]	6.69	5.23	5.74	1.47	0.96	-0.51	137.61	136.92	113.78	**23.14**

[a] from AIST-SDBS https://sdbs.db.aist.go.jp/sdbs/cgi-bin/direct_frame_top.cgi

Initial rates and final conversion for mixtures of 1a and 2a with DVE and NVP (Fig. 6)

The top two graphs are represented in Figure 6, where red curves relate to **1a** and blue curves to **2a**. The lower part (not displayed in the main text) indicates the combined conversion (of both butenolide and comonomer) at 2 h reaction, with light red bar relating to mixture with **1a**, and light blue bars to mixtures with **2a**. The graphs on the left relate to mixtures with DVE, graphs on the right related to mixtures with NVP.

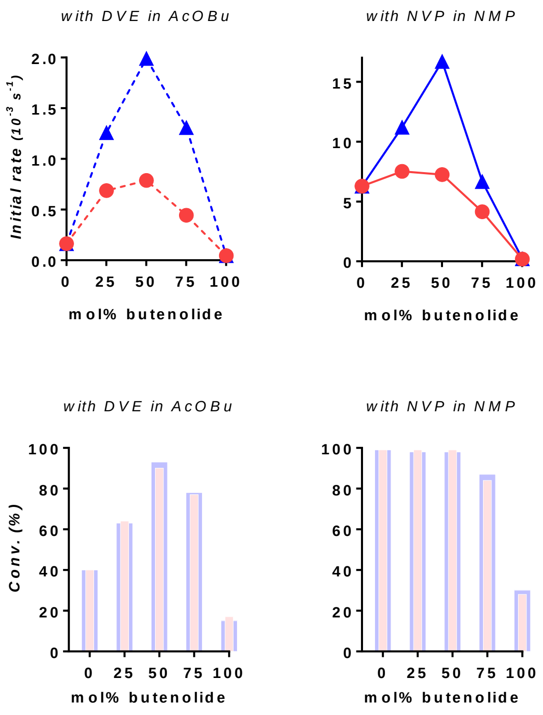

These graphs support an alternative copolymerization mechanism between electron-poor butenolides and polarized comonomers like DVE and NVP: 1) the copolymerization rate is maximal for equimolar mixtures (except for **1a**-NVP, see main text); 2) for non-homopolymerizable monomers (**1a**, **2a**, DVE), the combined conversion at 2 h is maximal for equimolar mixtures. For NVP-rich mixtures (less than 50% butenolide), the conversion is always high because the excess NVP can homopolymerize.

HOMO and LUMO energies (Figure 9B)

Below are the tabulated values for the graph in Figure 9B. The table also includes data for butenolide **2d**, which is not displayed in Figure 9B in the manuscript.

Compound	HOMO energy		Δ vs. BA	LUMO energy		Δ vs. BA	ΔLUMO-HOMO	
	Hartree	*kcal/mol*	*kcal/mol*	*Hartree*	*kcal/mol*	*kcal/mol*	*Hartree*	*kcal/mol*
**Furanone**	-0.32035	-201	+3.1	-0.01615	-10	**+3.8**	0.304	191
**Methyl furanone**	-0.32043	-201	+3.0	-0.01706	-11	**+3.2**	0.303	190
**Butyl acrylate (BA)**	-0.32527	-204	0.0	-0.02215	-14	**0.0**	0.303	190
**5-methoxy-5-methyl-butenolide**	-0.32410	-203	+0.7	-0.02515	-16	**-1.9**	0.299	188
**1a**	-0.32697	-205	-1.1	-0.02758	-17	**-3.4**	0.299	188
**2a**	-0.33486	-210	-6.0	-0.03542	-22	**-8.3**	0.299	188
**2d**	-0.33819	-212	-8.1	-0.03721	-23	**-9.5**	0.301	189
**Maleic anhydride**	-0.34717	-218	-13.7	-0.08139	-51	**-37.2**	0.266	167

LUMOs shape (Figure 9B)

Below are gathered the visualization of LUMOs (isovalue = 0.02) for relevant derivatives used for the graph in Figure 9B.

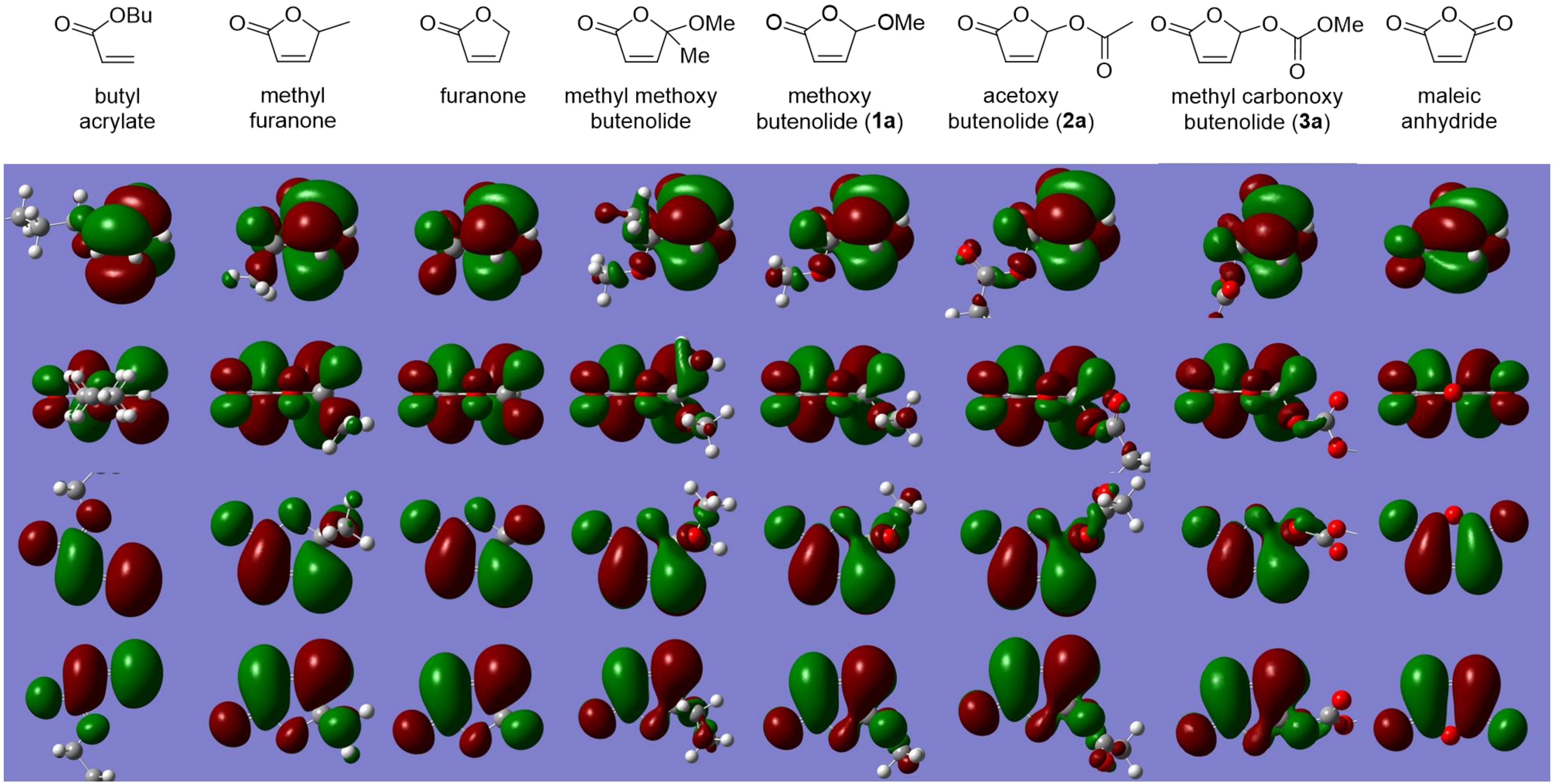

General

Commercial reagents and solvents: Unless stated otherwise, all commercially available reagents and solvents were purchased from the following commercial sources: Sigma–Aldrich, Acros, Macron, AkzoNobel Coatings BV and were used as received. Anhydrous solvents were purified by passage through solvent purification columns (MBraun SPS-800). For aqueous solutions, demineralized water was used. DCM means dichloromethane, THF means tetrahydrofuran, NMP means *N*-methylpyrrolidone.

Specific reagents: Vinyl neodecanoate (VeoVa-10) and tert-Butyl peroxy-3,5,5-trimethylhexanoate (Trigonox 42S) were obtained from Hexion and Nouryon, respectively.

General Considerations: Thin Layer Chromatography (TLC) analyses were performed on commercial Kieselgel 60, F254 silica gel plates with fluorescence-indicator UV_{254} (*Merck*, TLC silica gel *60 F_{254}*). For detection of components, UV light at 254 nm was used. Alternatively, oxidative staining using aqueous basic potassium permanganate solution ($KMnO_4$) was used. Drying of solutions was performed with Na_2SO_4 or $MgSO_4$ and volatiles were removed with a rotary evaporator. Flash column chromatography was conducted with a Büchi Pure Chromatography System at room temperature with pre-packed silica cartridges purchased from Macherey-Nagel.

General Analytical Information: Nuclear Magnetic Resonance spectra were measured with an Agilent Technologies 400-MR (400/54 Premium Shielded) spectrometer (400 MHz). All spectra were measured at room temperature (22–24°C). Chemical shifts for the specific NMR spectra were reported relative to the residual solvent peak [in ppm; $CDCl_3$: δ_H = 7.26; $CDCl_3$: δ_C = 77.16]. The multiplicities of the signals are denoted by *s* (singlet), *d* (doublet), *t* (triplet), *q* (quartet), *m* (multiplet), *br s* (broad signal), *app* (apparent). All ^{13}C-NMR spectra are ^{1}H-broadband decoupled.

High-resolution mass spectrometric measurements were performed using a Thermo scientific LTQ OrbitrapXL spectrometer with ESI or APPCI ionization. The results are given in *m/z*-units.

Molecular weight distribution was measured by GPC with a Hewlett Packard 1100 Series equipped with a GBC 1240 RID for detection. The flow rate of the mobile phase (THF) was set at 1 mL/min. The stationary phase was a set of 3 columns Agilent Technologies PLgel Mixed E 300 × 7.5 mm 3 µm, kept at 40°C. Injection volume was 20 µL. The GPC was calibrated using narrow band polystyrene standards. Toluene was added to samples as an internal standard for correction of retention time.

Glass temperatures (T_g) were measured with a Differential Scanning Calorimeter (DSC) Q1000 (TA Instruments) in aluminium pans covered with lids, both prepared from 0.1 mm aluminium sheets.

For some of the new products described herein, thermal properties were derived from differential scanning calorimetry (DSC) data when possible: melting point (m.p. in °C) was derived from the onset temperature of the detected endotherm and fusion enthalpy (in J/g) was calculated by integration of that endotherm.

Synthesis

Hydroxy butenolide **HB** was synthesized at 100 g-scale by continuous flow photooxygenation using a modular photoreactor (Hermens *et al.*, *React. Chem. Eng.* **2022**, *7*, 2280). Methoxy butenolide **1a** and 5-methoxy-5-methyl butenolide were synthesized as described previously (Hermens *et al.*, *Green Chem.* **2022**, *24*, 9772 and Hermens *et al.*, *Sci. Adv.* **2020**, *6*, eabe0026).

Method A (with anhydrides)

Hydroxy butenolide (1 eq.) was dissolved in DCM (0.5 to 1 M) in a flask under nitrogen atmosphere and the resulting solution was cooled to 0°C with an ice bath. The appropriate anhydride (1.2 eq.) was added, followed by *N*,*N*-dimethylaminopyridine (DMAP, 5 mol%). The mixture was stirred until full consumption of hydroxy butenolide (typically 1 to 2 h, monitored by TLC or NMR), allowing the reaction mixture to slowly warm up to room temperature. The reaction was quenched with methanol (2 eq. or more) and the mixture was stirred for an additional 10 min at room temperature. The reaction mixture was concentrated, and the crude residue was purified by automatic column chromatography to afford the pure acyloxy butenolide.

Method B (by Steglich esterification with acids)

In a flask under nitrogen atmosphere, hydroxy butenolide (1.50 eq.) was dissolved in DCM (0.75 M). The appropriate acid (1.00 eq.) and *N*,*N*-dimethylaminopyridine (DMAP, 5 mol%) were added. The clear mixture was cooled to 0°C with an ice bath. *N*,*N'*-Dicyclohexylcarbodiimide (DCC, 1.20 eq.) was dissolved in DCM (5 mL) and added dropwise at 0°C over 5 min. After the addition, the mixture was stirred at 0°C for 1 h, and then at room temperature until consumption of the acid. The reaction mixture was then filtered on cotton wool to remove the precipitated urea, rinsing with DCM. The filtrate was concentrated *in vacuo* and the crude residue was purified by automatic column chromatography.

Method C (with isocyanates)

Hydroxy butenolide (1.00 eq.) and an olive-shaped stir bar were added to a round-bottom flask, which was placed under vacuum and gently heated to melt the starting material and allow residual solvents to evaporate. Upon cooling back to room temperature, hydroxy butenolide solidified again. The flask was backfilled with nitrogen and dry THF was added (for a butenolide concentration of 1 to 2 M). To the clear solution were added successively the appropriate isocyanate (1.10 eq.) and dibutyl tin dilaurate (1 mol%). The clear reaction mixture was stirred at room temperature for 16 h or until full consumption of hydroxy butenolide (monitored by TLC or NMR). The reaction mixture was slowly concentrated, and the crude residue was purified by automatic column chromatography.

Tert-butoxy butenolide (1b)

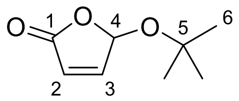

via

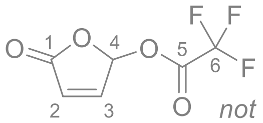

not isolated

5-(*tert*-butoxy)furan-2(5*H*)-one
Chemical Formula: $C_8H_{12}O_3$
Exact Mass: 156,0786
Molecular Weight: 156,1810

5-oxo-2,5-dihydrofuran-2-yl 2,2,2-trifluoroacetate
Chemical Formula: $C_6H_3F_3O_4$
Exact Mass: 195,9983
Molecular Weight: 196,0812

Hydroxy butenolide (1 eq., 2.00 g, 20.0 mmol, solid) was dissolved in anhydrous DCM (40 mL) and cooled to 0°C with an ice bath. Trifluoroacetic anhydride (1.2 eq., 3.40 mL, 24.0 mmol) was added, followed by a solution of DMAP (5 mol%, 122 mg, 1.00 mmol) in anhydrous DCM (0.5 mL), which caused immediate coloration of the reaction mixture. The mixture was stirred for 1 h, allowing it to warm up to room temperature. After 45 min, a sample (Pasteur pipet tip) from the reaction mixture was diluted in $CDCl_3$ and analyzed by ^{1}H NMR to confirm full and clean conversion to intermediate trifluoroacetoxy butenolide:

^{1}H NMR (400 MHz, Chloroform-d) δ 7.41 (d, J = 5.7 Hz, 1H, H-3), 7.05 (s, 1H, H-4), 6.46 (d, J = 5.7 Hz, 1H, H-2).

Tert-butanol (5 eq., 9.40 mL, 100 mmol) was added and the mixture was allowed to stir overnight (16 h) at room temperature. The mixture was then heated at reflux for 22.5 h, and then concentrated *in vacuo* to afford a light brown oil with colorless droplets (likely the excess trifluoroacetic anhydride or fluorinated side products). The residue was dissolved in AcOEt (25 mL), treated with sat. aq. $NaHCO_3$ (25 mL) and stirred until the gas evolution (CO_2) stopped. Then another portion of sat. aq. $NaHCO_3$ (25 mL) was added, without further gas release. The biphasic mixture was transferred to a separatory funnel and the layers were separated. The combined organic layers were further washed with sat. aq. $NaHCO_3$ (25 mL), brine (25 mL), dried with sodium sulfate and concentrated. The crude residue was further purified by column chromatography (40 g SiO2 cartridge, 0-20% AcOEt/pentane over 20 CV) to afford *tert*-butoxy butenolide **1b** as a colorless oil (1.99 g, 12.7 mmol, 64% yield).

^{1}H NMR (400 MHz, Chloroform-d) δ 7.12 (d, J = 5.6, 1H, H-3), 6.17 (d, J = 5.6, 1H, H-2), 6.12 (s, 1H, H-4), 1.35 (s, 9H, H-6).

^{13}C NMR (101 MHz, Chloroform-d) δ 171.26 (C-1), 151.76 (C-3), 124.52 (C-2), 98.75 (C-4), 78.00 (C-5), 28.56 (C-6).

HRMS ESI pos $[M+H]^+$ $C_8H_{13}O_3$ calc. 157.0859, found 157.0857.

m.p. 43°C, fusion enthalpy 119 J/g.

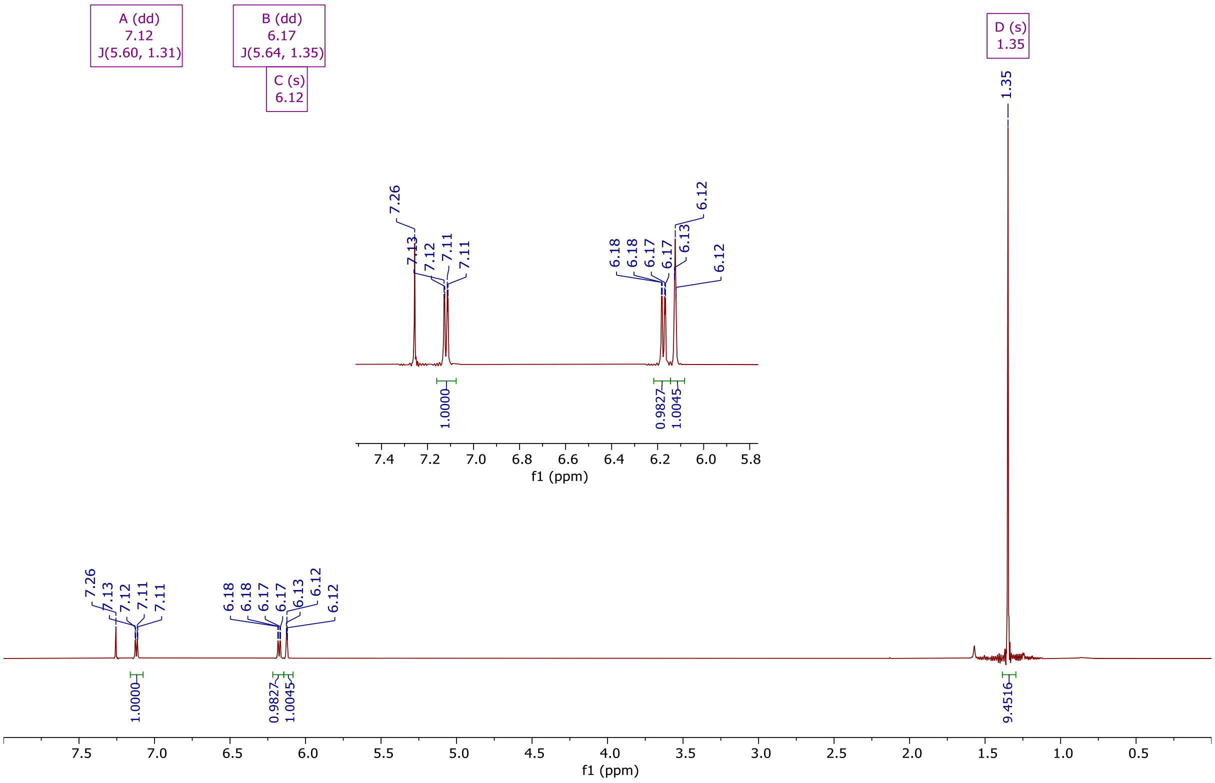
A (dd)
7.12
J(5.60, 1.31)
B (dd)
6.17
J(5.64, 1.35)
C (s)
6.12
D (s)
1.35
7.26
7.13
7.12
7.11
7.11
6.18
6.18
6.17
6.17
6.13
6.12
6.12
1.00
0.98
1.00
9.45
1.0000
0.9827
1.0045
9.4516
f1 (ppm)

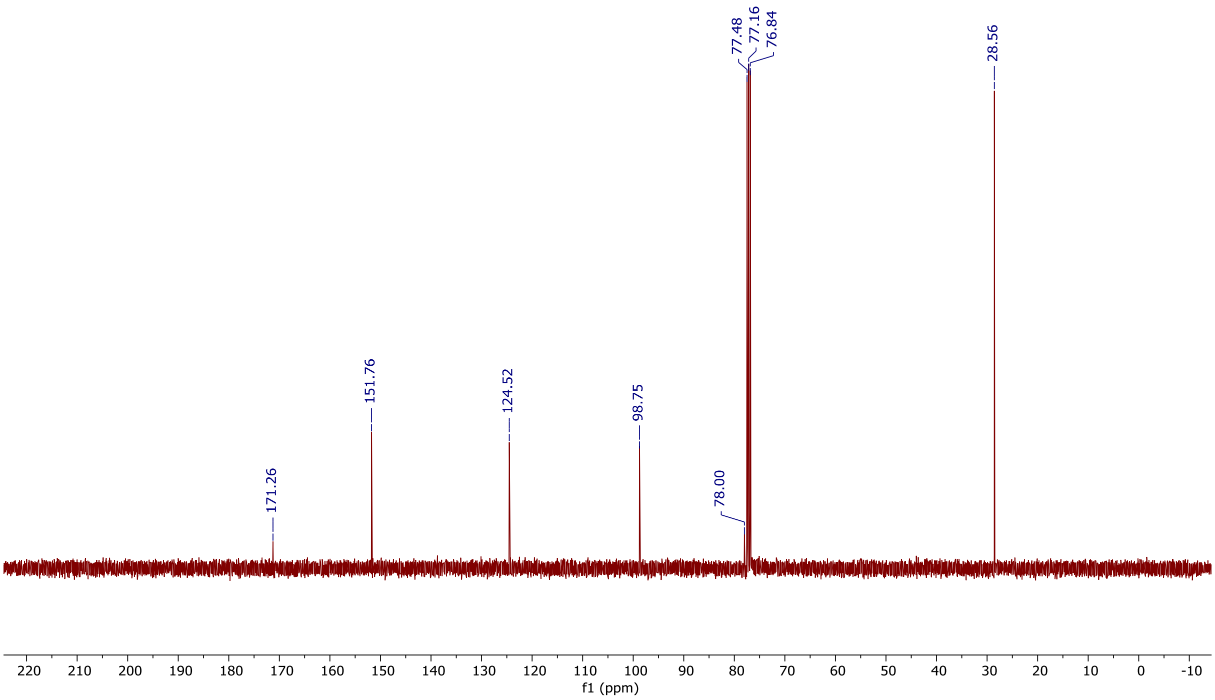
77.48
77.16
76.84
28.56
171.26
151.76
124.52
98.75
78.00
f1 (ppm)

Acetoxy butenolide (2a)

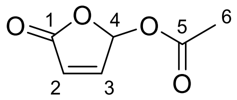

5-oxo-2,5-dihydrofuran-2-yl acetate
Chemical Formula: $C_6H_6O_4$
Exact Mass: 142.0266
Molecular Weight: 142.1100

This product and its synthesis were previously described in:

G.C. Resende, E.S. Alvarenga, J.C.G. Galindo, F.A. Macias, *J. Braz. Chem. Soc.* **2012,** *23* (12), 2266-2270.

Compound **2a** was synthesized from hydroxy butenolide (10.00 g, 100 mmol) and acetic anhydride following **Method A**. The crude residue was purified by automatic column chromatography (120 g SiO_2 cartridge, 5-35% AcOEt/pentane over 20 CV), using DCM for liquid injection. Concentration of the collected fraction afforded pure **2a** as a colorless liquid (10.75 g, 75.6 mmol, 76% yield).

^{1}H NMR (400 MHz, Chloroform-d) δ 7.32 (d, J = 5.7 Hz, 1H, H-3), 7.01 (s, 1H, H-4), 6.31 (d, J = 5.7 Hz, 1H, H-2), 2.16 ppm (s, 3H, H-6).

^{13}C NMR (101 MHz, Chloroform-d) δ 169.75 (C-1), 169.00 (C-5), 149.85 (C-3), 125.28 (C-2), 93.87 (C-4), 20.72 (C-6).

HRMS ESI-pos $[M+H]^+$ $C_6H_7O_4$ calc. 143.0339, found 143.0331.

m.p. < –50°C.

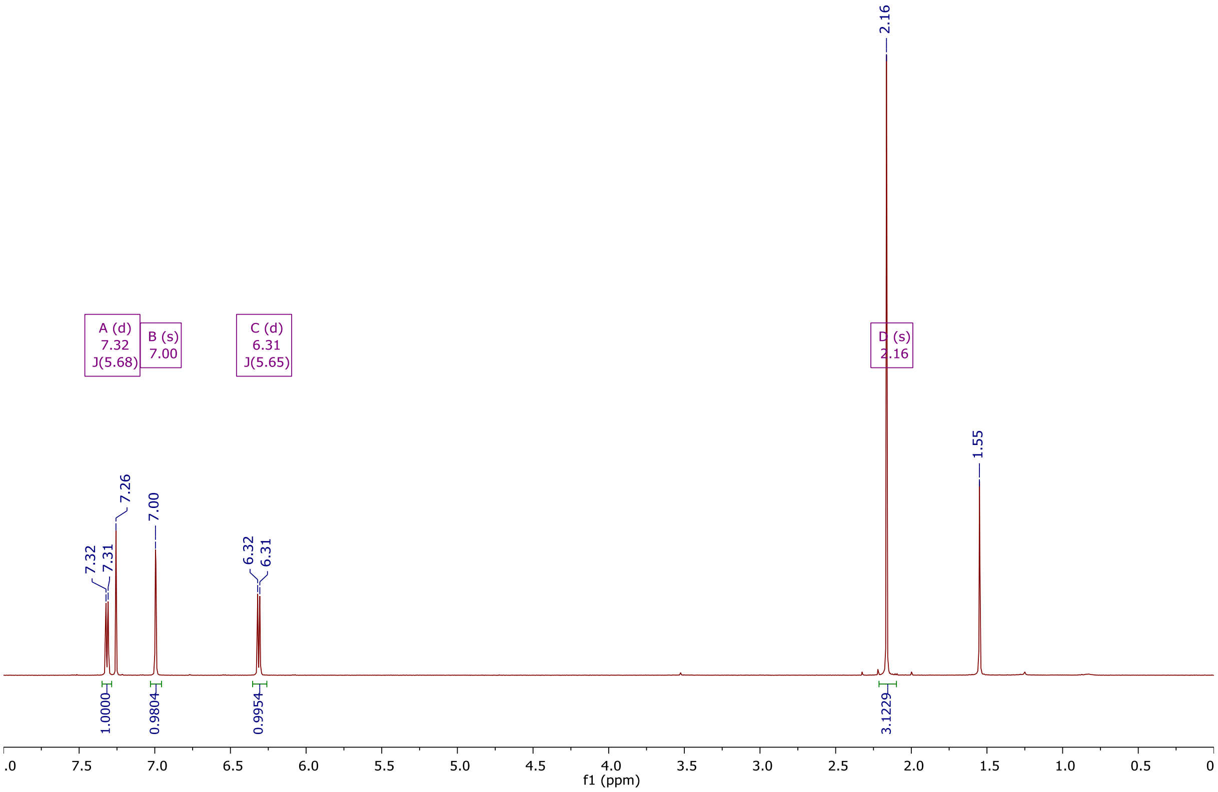
A (d)
7.32
J(5.68)
B (s)
7.00
C (d)
6.31
J(5.65)
D (s)
2.16
7.32
7.31
7.26
7.00
6.32
6.31
2.16
1.55
1.0000
0.9804
0.9954
3.1229
f1 (ppm)

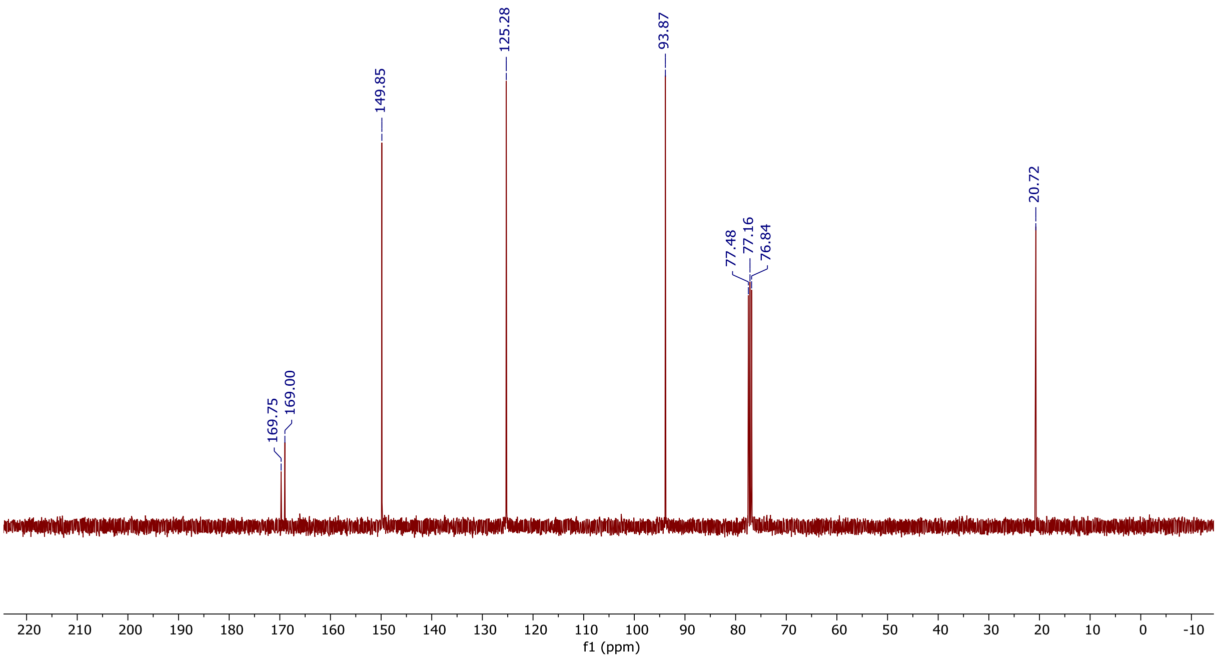
169.75
169.00
149.85
125.28
93.87
77.48
77.16
76.84
20.72
f1 (ppm)

Isobutyroxy butenolide (2b)

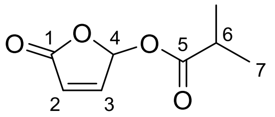

5-oxo-2,5-dihydrofuran-2-yl isobutyrate
Chemical Formula: $C_8H_{10}O_4$
Exact Mass: 170.0579
Molecular Weight: 170.1640

Compound **2b** was synthesized from hydroxy butenolide and isobutyric acid (3.70 mL, 40.0 mmol) following **Method B**. The crude residue was purified by automatic column chromatography (80 g SiO_2 cartridge, 5-30% AcOEt/pentane over 15 CV), using DCM for liquid injection. Concentration of the collected fraction afforded pure **2b** as a yellow oil (4.84 g, 28.4 mmol, 71% yield).

^{1}H NMR (400 MHz, $CDCl_3$) δ 7.32 (dd, J = 5.6, 1.4 Hz, 1H, H-3), 7.01 (t, J = 1.3 Hz, 1H, H-4), 6.31 (dd, J = 5.7, 1.2 Hz, 1H, H-2), 2.62 (hept, J = 7.0 Hz, 1H, H-6), 1.21 (dd, J = 7.0, 1.8 Hz, 6H, H-7).

^{13}C NMR (101 MHz, $CDCl_3$) δ 175.16 (C-5), 169.84 (C-1), 149.92 (C-3), 125.32 (C-2), 93.99 (C-4), 33.97 (C-6), 18.75, 18.71 (C-7).

HRMS APPCI-neg $[M-H]^-$ $C_8H_9O_4$ calc. 169.0495, found 169.0507.

m.p. < –50°C.

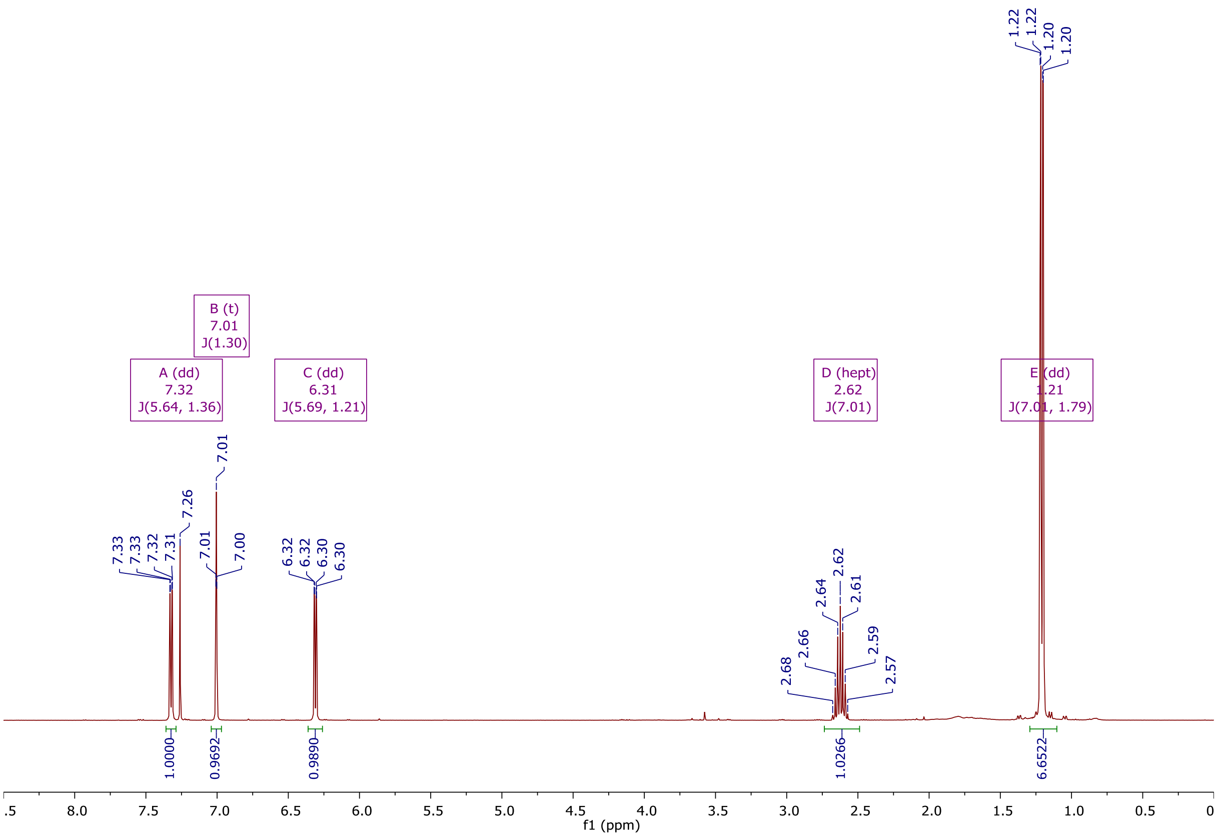
A (dd)
7.32
J(5.64, 1.36)
B (t)
7.01
J(1.30)
C (dd)
6.31
J(5.69, 1.21)
D (hept)
2.62
J(7.01)
E (dd)
1.21
J(7.01, 1.79)
7.33 7.33 7.32 7.31 7.26 7.01 7.01 7.00
6.32 6.32 6.30 6.30
2.68 2.66 2.64 2.62 2.61 2.59 2.57
1.22 1.22 1.20 1.20
1.0000 0.9692 0.9890 1.0266 6.6522
f1 (ppm)

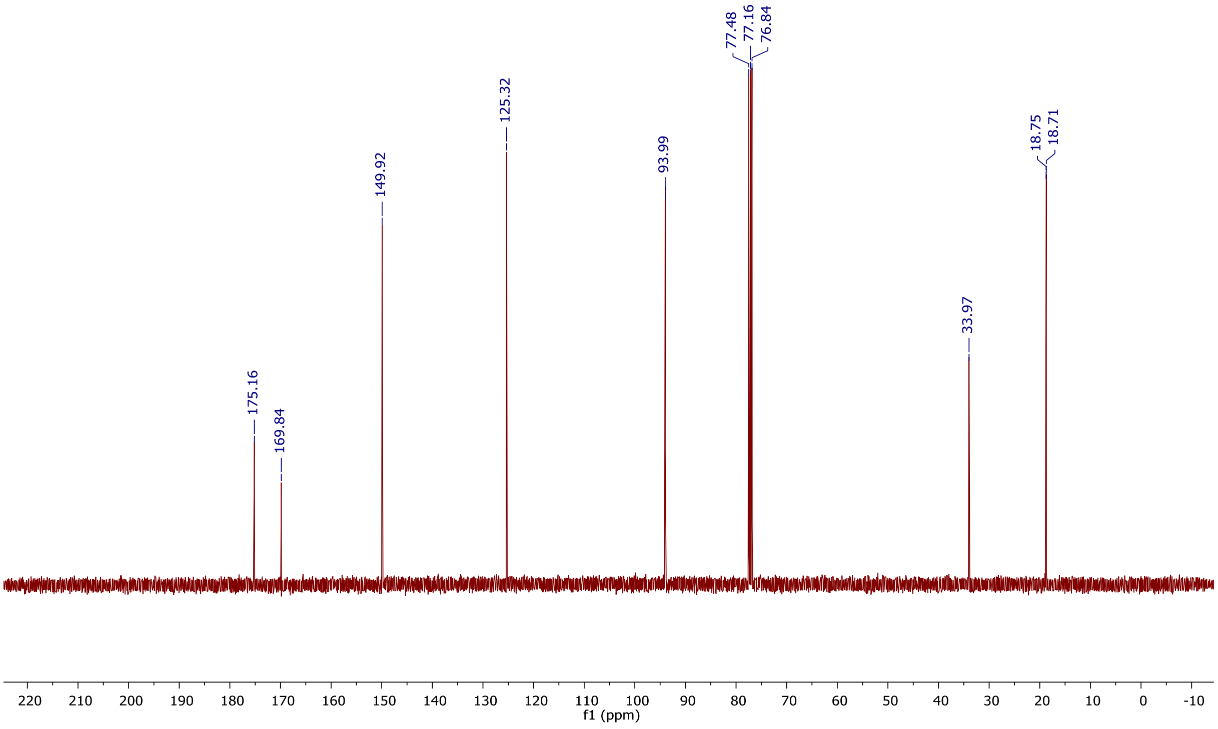
175.16
169.84
149.92
125.32
93.99
77.48 77.16 76.84
33.97
18.75 18.71
f1 (ppm)

Pivaloyloxy butenolide (2c)

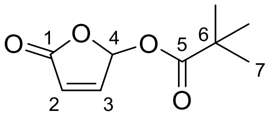

5-oxo-2,5-dihydrofuran-2-yl pivalate
Chemical Formula: $C_9H_{12}O_4$
Exact Mass: 184.0736
Molecular Weight: 184.1910

Compound **2c** was synthesized from hydroxy butenolide (5.00 g, 50.0 mmol) and pivalic anhydride following **Method A**. The crude residue was purified by automatic column chromatography (80 g SiO_2 cartridge, 5-30% AcOEt/pentane over 15 CV), using DCM for liquid injection. Concentration of the collected fraction afforded pure **2c** as a pale yellow oil (6.74 g, 36.6 mmol, 73% yield).

^{1}H NMR (400 MHz, $CDCl_3$) δ 7.32 (d, J = 5.7 Hz, 1H, H-3), 6.99 (s, 1H, H-4), 6.31 (d, J = 5.7 Hz, 1H, H-2), 1.24 (s, 9H, H-7).

^{13}C NMR (101 MHz, $CDCl_3$) δ 176.62 (C-5), 169.89 (C-1), 149.97 (C-3), 125.34 (C-2), 94.18 (C-4), 39.11 (C-6), 26.98 (C-7).

HRMS APPCI-neg $[M-H]^-$ $C_9H_{11}O_4$ calc. 183.0652, found 183.0662.

m.p. 28–35°C, fusion enthalpy 80 J/g.

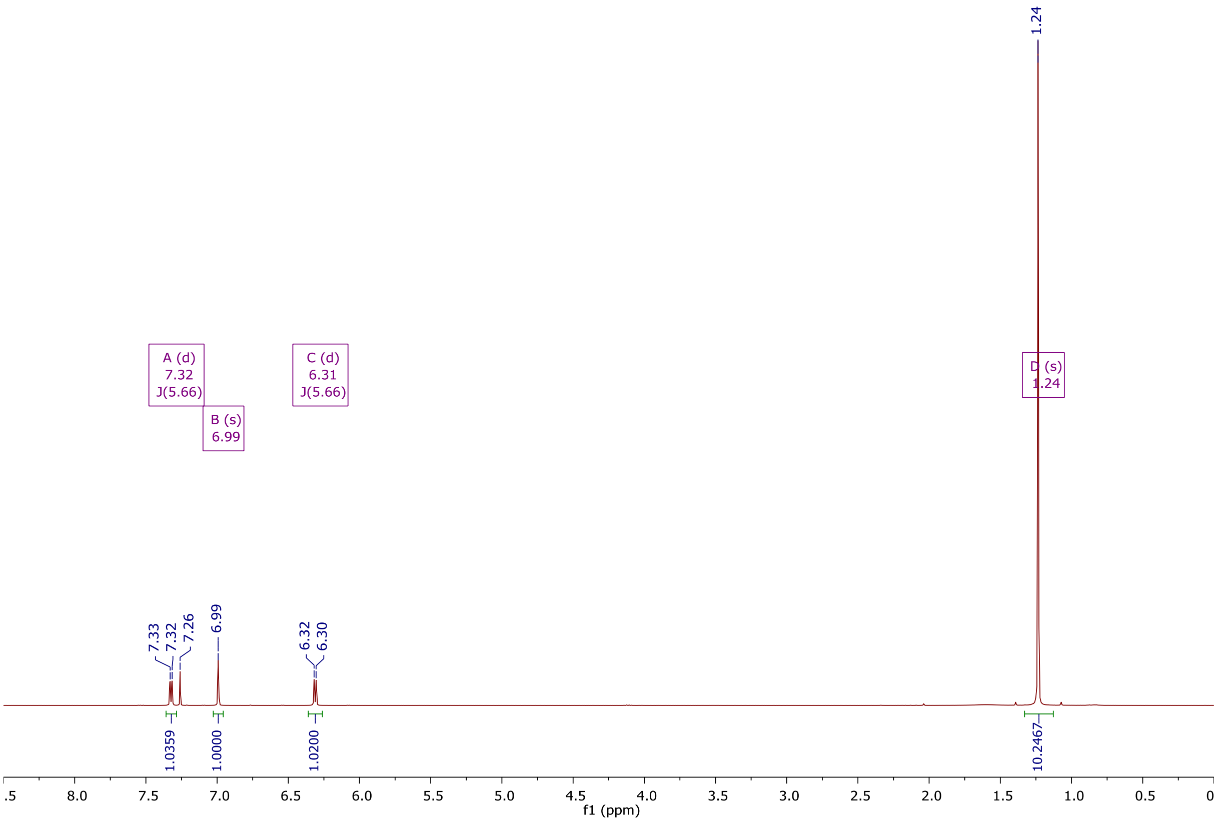
1.24
A (d)
7.32
J(5.66)
B (s)
6.99
C (d)
6.31
J(5.66)
D (s)
1.24
7.33
7.32
7.26
6.99
6.32
6.30
1.0359
1.0000
1.0200
10.2467
f1 (ppm)

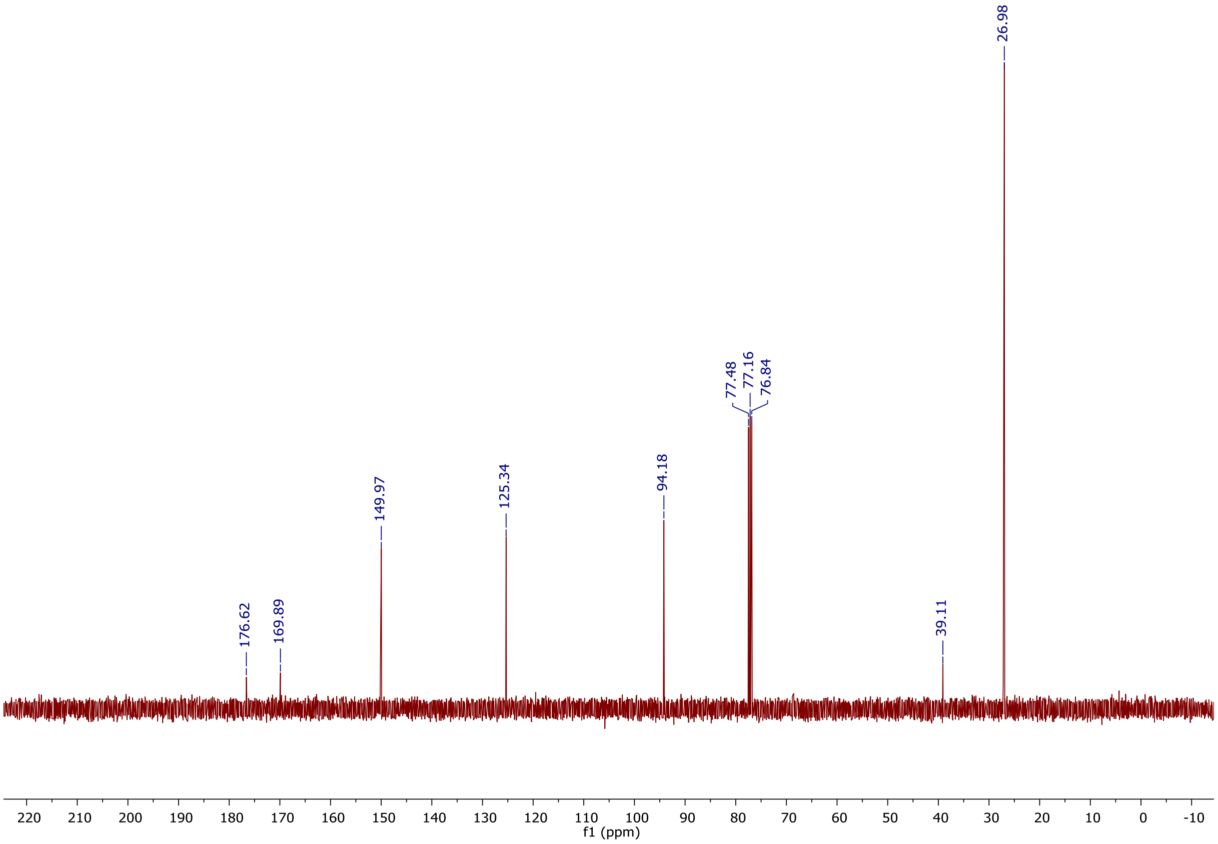
26.98
77.48
77.16
76.84
94.18
125.34
149.97
176.62
169.89
39.11
f1 (ppm)

Benzoyloxy butenolide (2d)

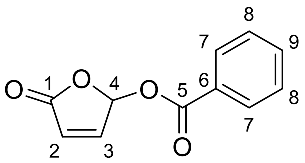

5-oxo-2,5-dihydrofuran-2-yl benzoate
Chemical Formula: $C_{11}H_8O_4$
Exact Mass: 204.0423
Molecular Weight: 204.1810

This product was previously described in:

B.M. Trost, F.D. Toste, *J. Am. Chem. Soc.* **2003,** *125* (10), 3090-3100.

Compound **2d** was synthesized from hydroxy butenolide (160 mg, 1.60 mmol) and benzoic anhydride following **Method A**. The crude residue was purified by automatic column chromatography (15 g SiO_2 cartridge, 0-30% AcOEt/pentane over 20 CV), using DCM for liquid injection. Concentration of the collected fraction afforded pure **2d** as a white solid (236 mg, 1.16 mmol, 73% yield).

^{1}H NMR (400 MHz, Chloroform-d) δ 8.05 (d, J = 8.0 Hz, 2H, H-8), 7.61 (t, 7.4 Hz, 1H, H-9), 7.47 (m, 3H, H-3 and H-7), 7.25 (s, 1H, H-4, overlaps with residual $CHCl_3$ peak), 6.39 ppm (dd, J = 5.7 Hz, 1.1 Hz, 1H, H-2).

^{13}C NMR (101 MHz, Chloroform-d) δ 169.80 (C-1), 164.68 (C-5), 149.96 (C-3), 134.33 (C-9), 130.29, 128.81 (C-7 and C-8), 128.25 (C-6), 125.54 (C-2), 94.58 (C-4).

HRMS ESI-pos $[M+Na]^+$ $C_{11}H_8O_4Na$ calc. 227.0315, found 227.0312.

m.p. 111°C, fusion enthalpy 127 J/g.

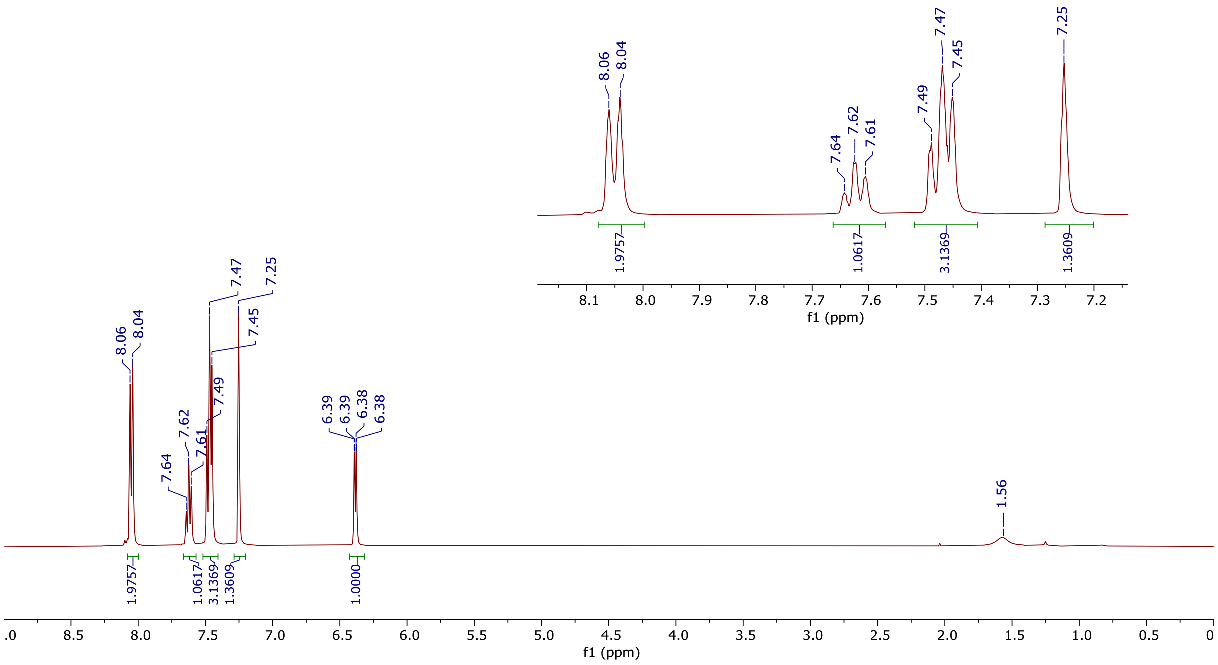
8.06
8.04
7.64
7.62
7.61
7.49
7.47
7.45
7.25
1.9757
1.0617
3.1369
1.3609
8.1
8.0
7.9
7.8
7.7
7.6
7.5
7.4
7.3
7.2
f1 (ppm)
6.39
6.39
6.38
6.38
1.56
1.0000
8.5
7.0
6.5
6.0
5.5
5.0
4.5
4.0
3.5
3.0
2.5
2.0
1.5
1.0
0.5
0

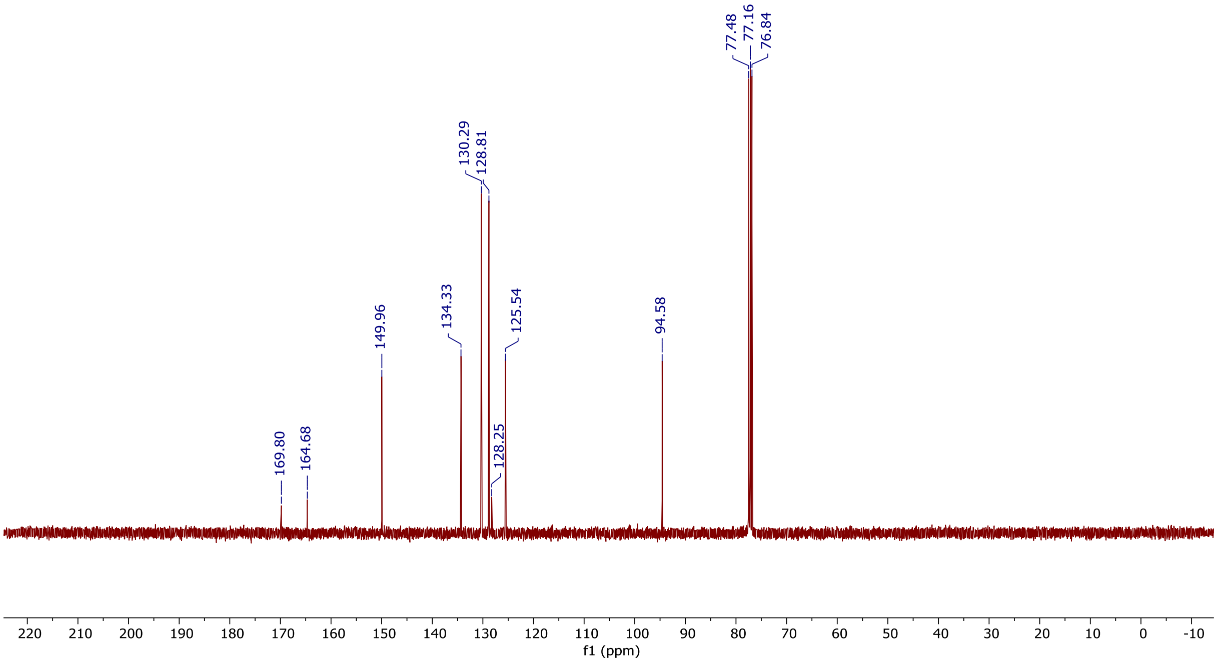
77.48
77.16
76.84
130.29
128.81
149.96
134.33
125.54
94.58
169.80
164.68
128.25
220
210
200
190
180
170
160
150
140
130
120
110
100
90
80
70
60
50
40
30
20
10
0
-10
f1 (ppm)

Lauryloxy butenolide (2e)

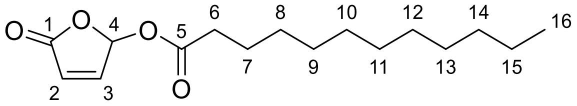

5-oxo-2,5-dihydrofuran-2-yl dodecanoate
Chemical Formula: $C_{16}H_{26}O_4$
Exact Mass: 282.1831
Molecular Weight: 282.3800

Lauryloxy butenolide was synthesized following a procedure adapted from Guo, *Chin. J. Chem.* **2005**, *23*, 1683.

A solution of lauric acid (1 eq., 1.00 g, 5.00 mmol) in anhydrous toluene (15 mL, [lauric acid] = 0.33 M) was prepared in a reflux apparatus and stirred under N_2 atmosphere. Thionyl chloride (6.5 eq., 2.4 mL, 3.9 g) was added at room temperature to the clear and colorless solution. The mixture was heated to reflux for 4 h, over which the reaction mixture turned yellow. The reaction mixture was then concentrated under vacuum (water bath = 50°C). The crude was re-dissolved in anhydrous toluene (15 mL) and hydroxy butenolide (1 eq., 500 mg, 5.00 mmol) was added to the resulting solution. The mixture was heated to reflux overnight. Upon heating, the initially insoluble hydroxy butenolide dissolved. TLC [20% AcOEt/hexane, rev. $KMnO_4$] showed appearance of a clear spot at R_f = 0.45, compared to hydroxy butenolide at R_f = 0.20. After cooling down, the mixture was concentrated. The crude was purified by automatic column chromatography (15 g SiO_2 cartridge, AcOEt/pentane 0-25% over 20 CV), using DCM as solvent for liquid injection. Concentration of the collected fraction afforded pure **2e** as a white solid (925 mg, 3.28 mmol, 66% yield).

^{1}H NMR (400 MHz, Chloroform-d) δ 7.31 (d, J = 5.7 Hz, 1H, H-3), 7.01 (t, J = 1.1 Hz, 1H, H-4), 6.31 (dd, J = 5.7 Hz, 1.1 Hz, 1H, H-2), 2.39 (t, J = 7.5 Hz, 2H, H-6), 1.65 (p, J = 7.4 Hz, 2H, H-7), 1.29 – 1.20 (m, 16H, H-8 to H-15), 0.87 ppm (t, J = 6.7 Hz, 3H, H-16).

^{13}C NMR (101 MHz, Chloroform-d) δ 171.92 (C-5), 169.83 (C-1), 149.90 (C-3), 125.34 (C-2), 93.87 (C-4), 34.00 (C-6), 32.04 (C-7), 29.73, 29.71, 29.53, 29.47, 29.31, 29.10, 24.61 (C-8 to C-14), 22.83 (C-15), 14.28 ppm (C-16).

HRMS ESI-pos $[M+Na]^+$ $C_{16}H_{26}O_4Na$ calc. 305.1733, found 305.1721.

m.p. 53°C, fusion enthalpy 165 J/g.

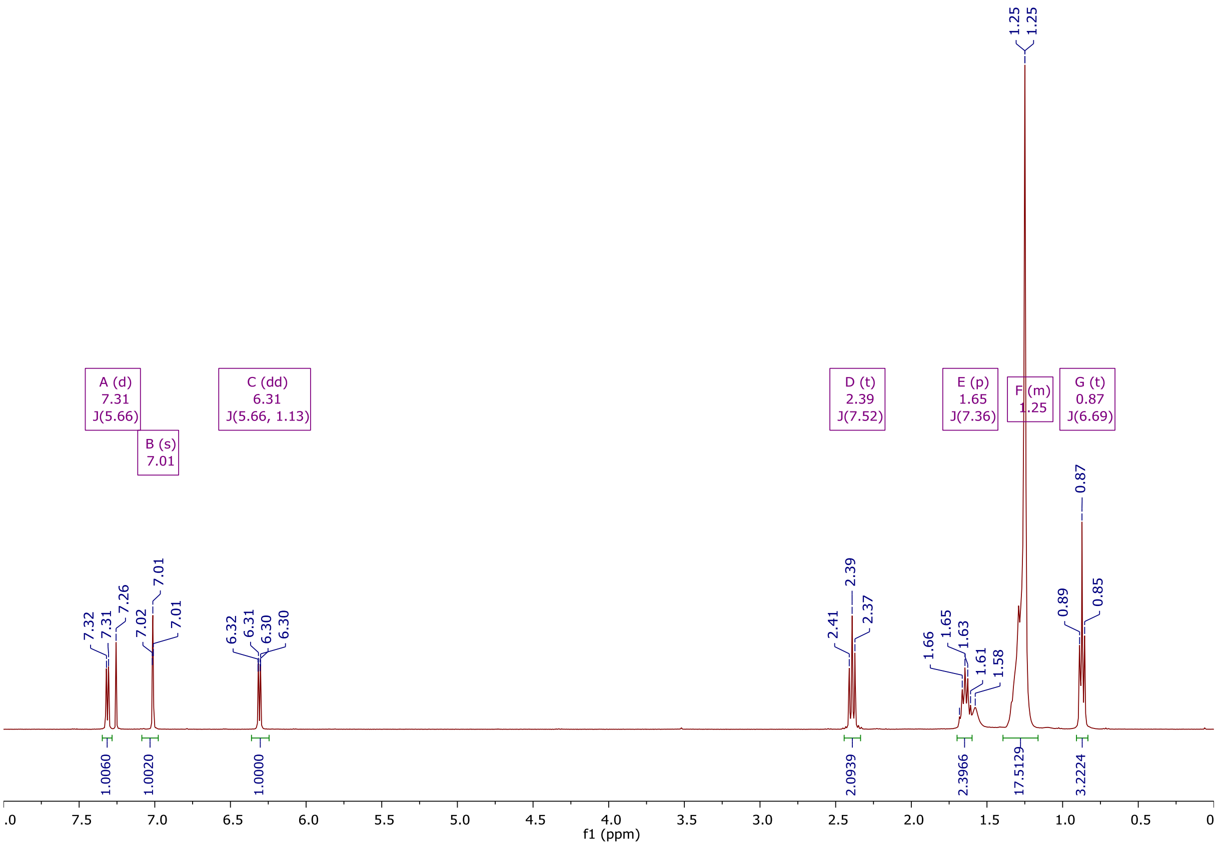
A (d)
7.31
J(5.66)
B (s)
7.01
C (dd)
6.31
J(5.66, 1.13)
D (t)
2.39
J(7.52)
E (p)
1.65
J(7.36)
F (m)
1.25
G (t)
0.87
J(6.69)
1.0060
1.0020
1.0000
2.0939
2.3966
17.5129
3.2224
f1 (ppm)

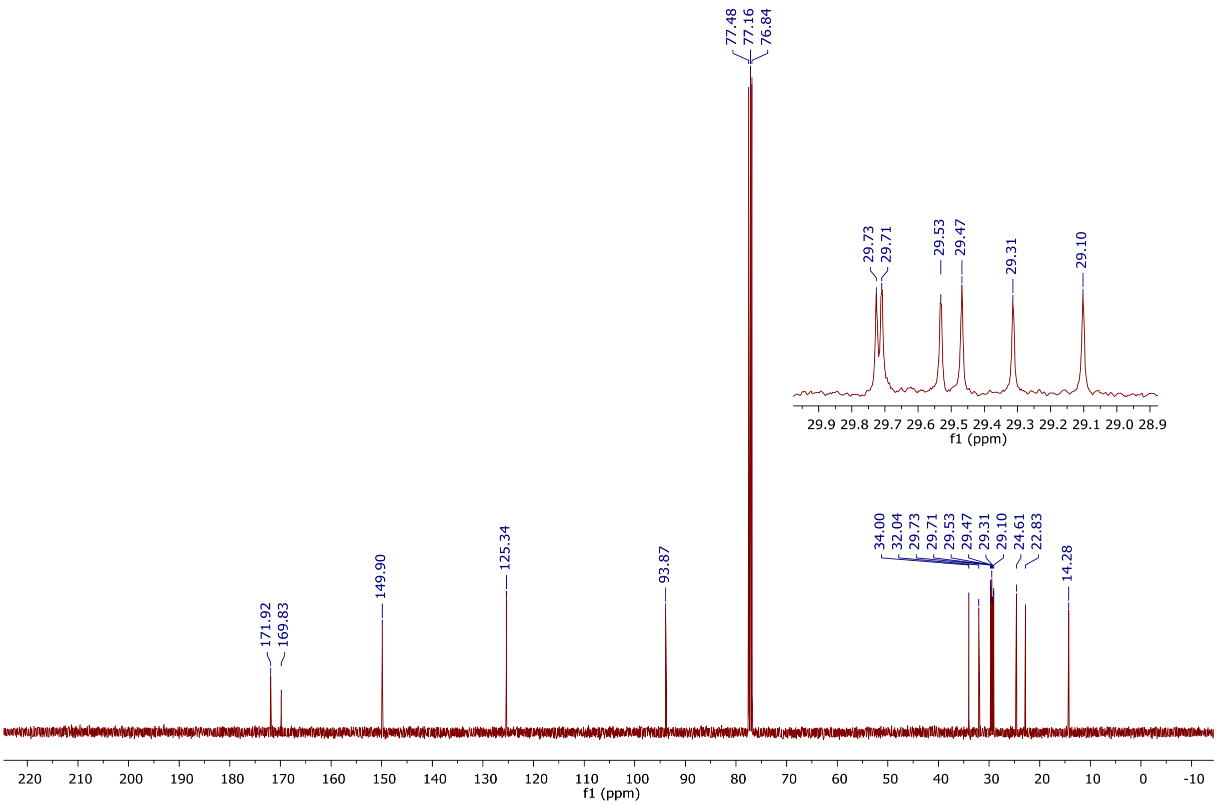
171.92
169.83
149.90
125.34
93.87
77.48
77.16
76.84
34.00
32.04
29.73
29.71
29.53
29.47
29.31
29.10
24.61
22.83
14.28
f1 (ppm)

Oleyloxy butenolide (2f)

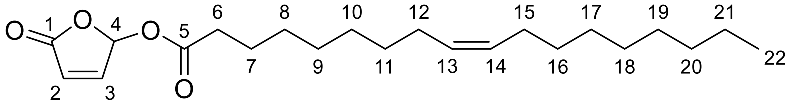

5-oxo-2,5-dihydrofuran-2-yl oleate
Chemical Formula: $C_{22}H_{36}O_4$
Exact Mass: 364.2614
Molecular Weight: 364.5260

In a flask under N_2 atmosphere, hydroxy butenolide **HB** (1.00 eq., 1.00 g, 10.0 mmol) was dissolved in anhydrous DCM (20 mL, [**HB**] = 0.5 M). Oleic acid (2.00 eq., 5.64 g, 20.0 mmol) in anhydrous DCM (2.5 mL) and *N*,*N*-dimethylaminopyridine (5 mol%, 61 mg, 0.50 mmol) were added. The clear mixture was cooled to 0°C with an ice bath. DCC (1.20 eq., 2.47 g, 12.0 mmol) was dissolved in anhydrous DCM (2.5 mL) and added dropwise at 0°C over 5 min. During the addition, a white precipitate formed. The slurry then slowly turned brown over time. After 30 min, the ice bath was removed and the mixture was stirred at room temperature for 1 h, until TLC confirmed complete consumption of hydroxy butenolide.

The reaction mixture was filtered, rinsing with DCM, affording a white solid (urea) and a brown filtrate (ca. 50 mL), which was washed with sat. aq. $NaHCO_3$ (2 x 25 mL). The organic layer was dried with sodium sulfate, filtered and then concentrated in vacuo to a brown oil. The residue was purified by automatic column chromatography (40 g SiO_2 cartridge, 0-100% DCM/pentane over 20 CV), using hexanes for liquid injection. Concentration of the collected fraction afforded pure oleyloxy butenolide (950 mg, 2.61 mmol, 26% yield) as a yellow oil that solidifies around 15°C.

^{1}H NMR (400 MHz, $CDCl_3$) δ 7.31 (dd, J = 5.7, 1.4 Hz, 1H, H-3), 7.02 (t, J = 1.3 Hz, 1H, H-4), 6.31 (dd, J = 5.7, 1.2 Hz, 1H, H-2), 5.41 – 5.24 (m, 2H, H-13 and H-14), 2.40 (t, J = 7.4 Hz, 2H, H-6), 2.01 (q, J = 6.2 Hz, 4H, H-12 and H-15), 1.66 (p, J = 7.2 Hz, 2H, H-7), 1.40 – 1.18 (m, 20H, H-8 to H-11 and H-16 to H-21), 0.88 (t, J = 6.8 Hz, 3H, H-22).

^{13}C NMR (101 MHz, $CDCl_3$) δ 171.86 (C-5), 169.77 (C-1), 149.86 (C-3), 130.22, 129.83 (C-13 and C-14), 125.35 (C-2), 93.90 (C-4), 34.01, 32.05, 29.91, 29.81, 29.67, 29.47, 29.47, 29.22, 29.18, 29.09, 27.38, 27.29, 24.61, 22.83, 14.26 (C-6 to C-12 and C-15 to C-22).

HRMS ESI-pos $[M+Na]^+$ $C_{22}H_{36}O_4Na$ calc. 387.2506, found 387.2485.

m.p. 12°C, fusion enthalpy 73 J/g.

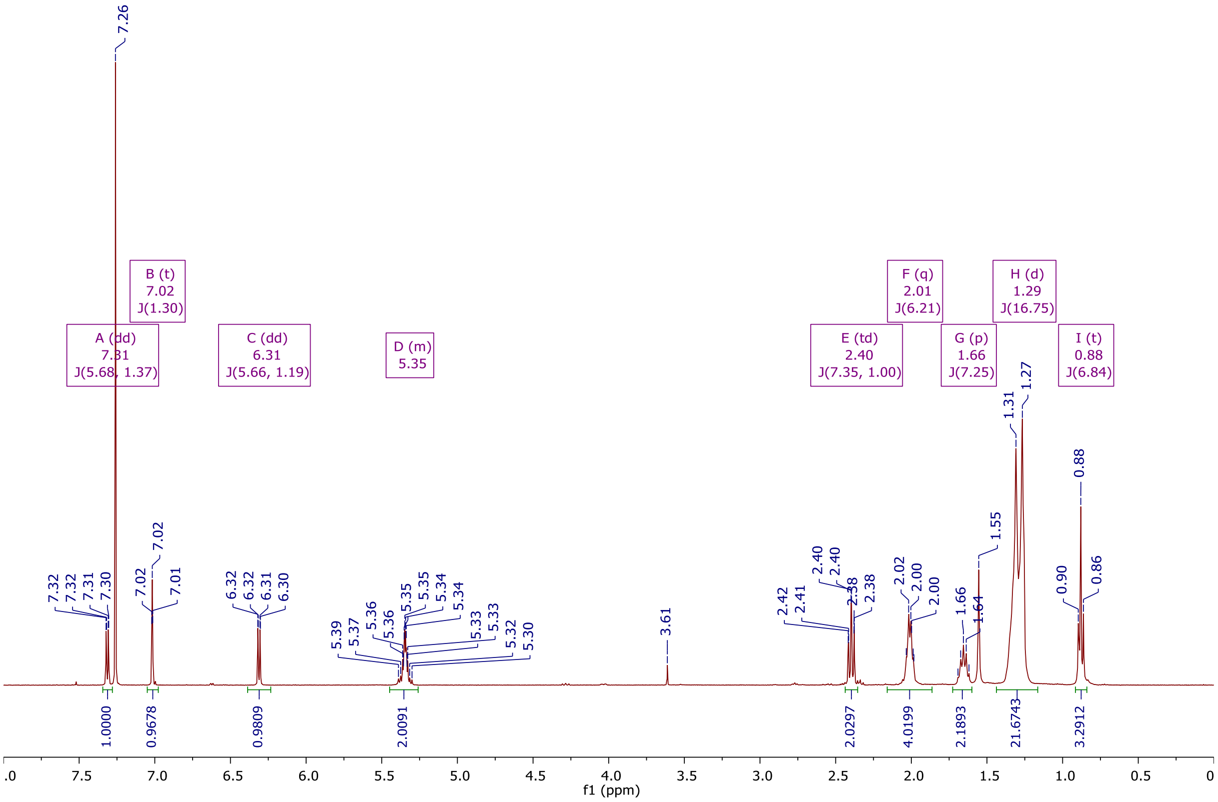
A (dd) 7.31 J(5.68, 1.37)
B (t) 7.02 J(1.30)
C (dd) 6.31 J(5.66, 1.19)
D (m) 5.35
E (td) 2.40 J(7.35, 1.00)
F (q) 2.01 J(6.21)
G (p) 1.66 J(7.25)
H (d) 1.29 J(16.75)
I (t) 0.88 J(6.84)
7.26
3.61
1.0000
0.9678
0.9809
2.0091
2.0297
4.0199
2.1893
21.6743
3.2912
f1 (ppm)

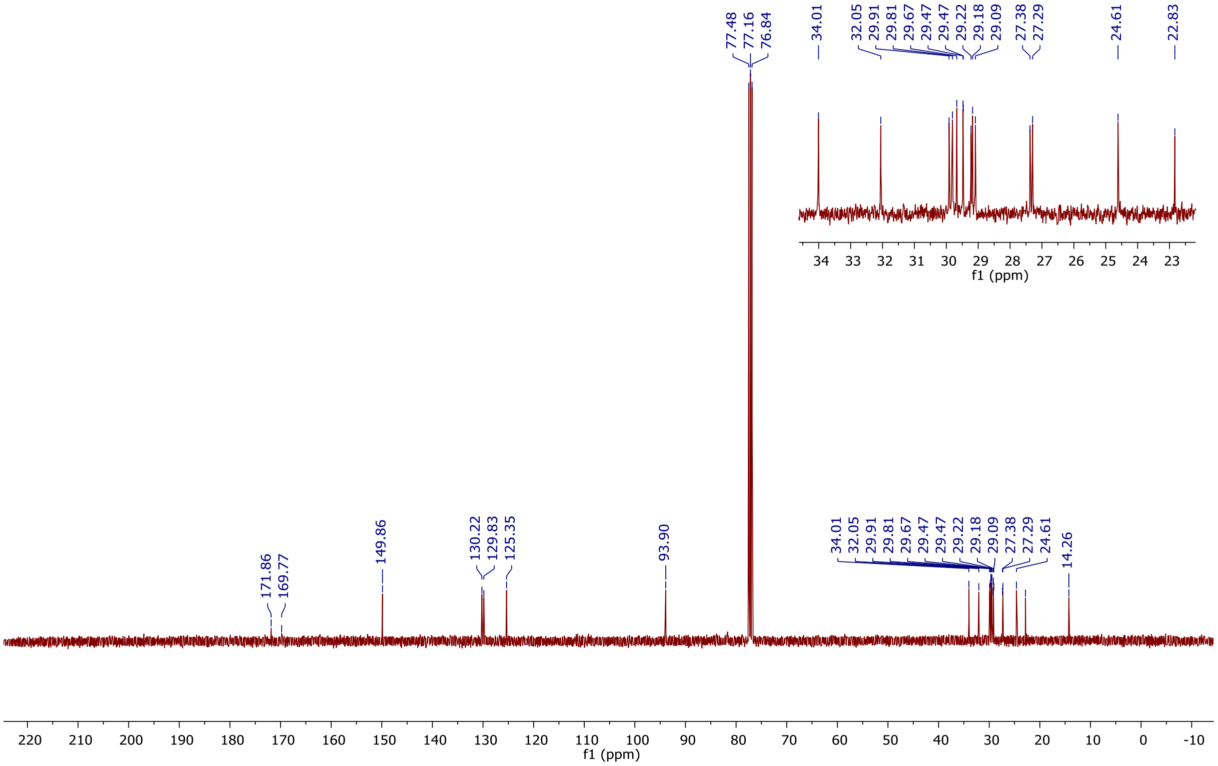
171.86
169.77
149.86
130.22
129.83
125.35
93.90
77.48
77.16
76.84
34.01
32.05
29.91
29.81
29.67
29.47
29.22
29.18
29.09
27.38
27.29
24.61
22.83
14.26
f1 (ppm)

Succinyloxy butenolide (2g)

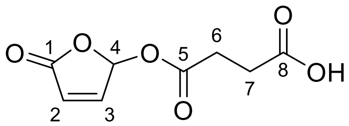

4-oxo-4-((5-oxo-2,5-dihydrofuran-2-yl)oxy)butanoic acid
Chemical Formula: $C_8H_8O_6$
Exact Mass: 200.0321
Molecular Weight: 200.1460

On small scale, **2g** was synthesized from hydroxy butenolide (1.00 g, 10.0 mmol) and succinic anhydride following **Method A**. The crude residue was purified by automatic column chromatography (25 g SiO_2 cartridge, 10-60% AcOEt/pentane over 25 CV, then 60-100% over 5 CV), using solid loading (adsorbed on SiO_2) and adding 1 vol% AcOH in AcOEt to afford pure **2g** (1.13 g, 5.63 mmol, 56% yield) as a white solid.

On a larger scale, **2g** was synthesized from hydroxy butenolide (10.0 g, 99.9 mmol) and succinic anhydride (1.10 eq.) following **Method A**. After concentration, the crude residue was a paste that was filtered on a glass frit (P4) and rinsed with ice-cold chloroform (50 mL) and then hexanes (excess) to afford **2g** as a white solid (16.4 g, 82.0 mmol, 82% yield).

^{1}H NMR (400 MHz, Chloroform-d) δ 7.34 (dd, *J* = 5.7, 1.4 Hz, 1H, H-3), 7.02 (t, *J* = 1.3 Hz, 1H, H-4), 6.33 (dd, *J* = 5.7, 1.2 Hz, 1H, H-2), 2.94 – 2.39 (m, 4H, H-6 and H-7).

^{13}C NMR (101 MHz, Chloroform-d) δ 176.83 (C-8), 170.48 (C-5), 169.67 (C-1), 149.66 (C-3), 125.48 (C-2), 94.11 (C-4), 28.72, 28.41 (C-6 and C-7).

HRMS ESI-pos $[M+Na]^+$ $C_8H_8O_6Na$ calc. 223.0213, found 223.0202.

m.p. > 90°C (decomposes).

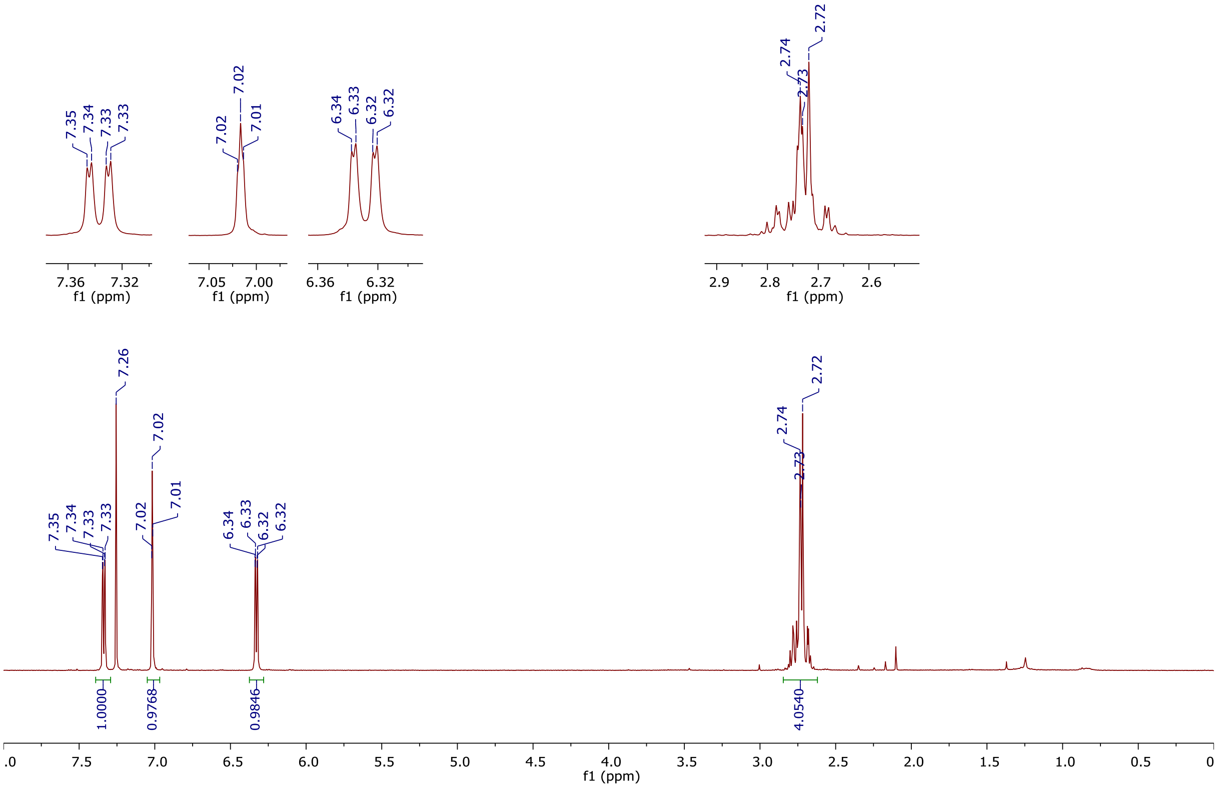
7.35
7.34
7.33
7.33
7.02
7.02
7.01
6.34
6.33
6.32
6.32
2.74
2.73
2.72
7.26
1.0000
0.9768
0.9846
4.0540
f1 (ppm)

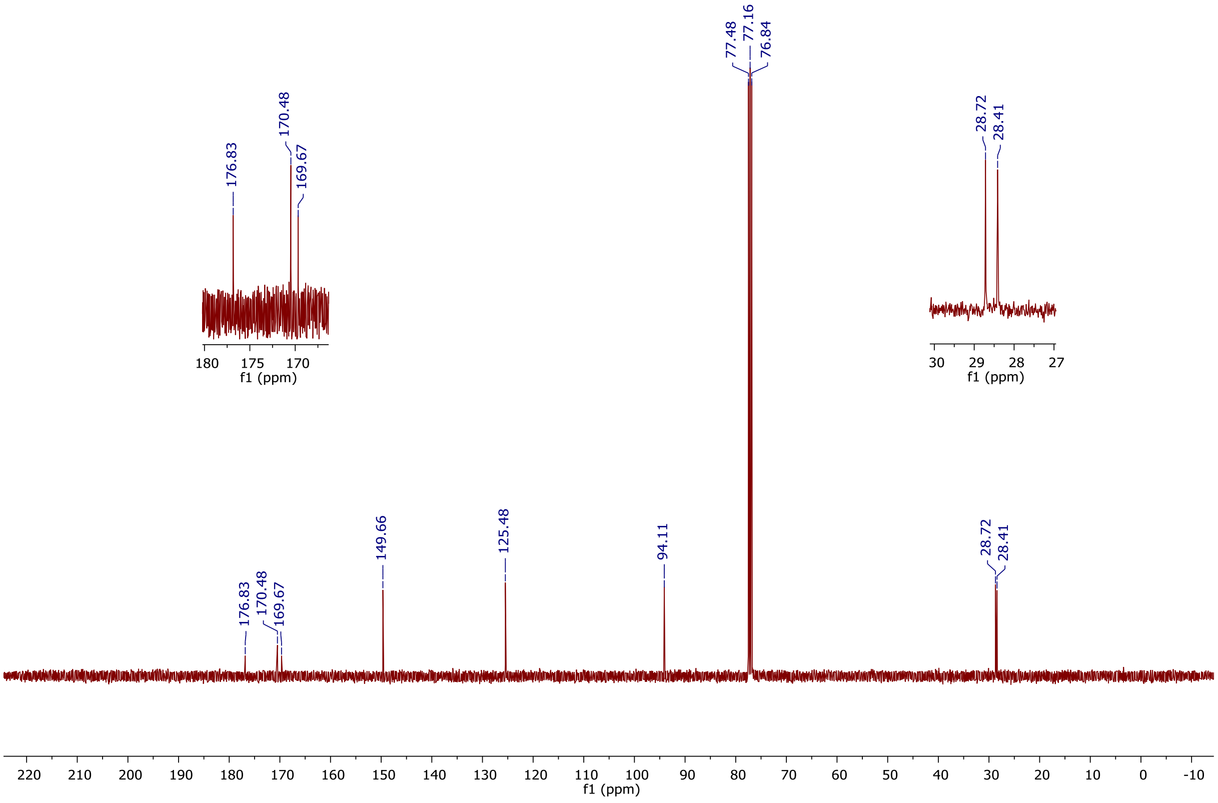
176.83
170.48
169.67
149.66
125.48
94.11
77.48
77.16
76.84
28.72
28.41
f1 (ppm)

Succinyloxy butenolide methyl ester (2h)

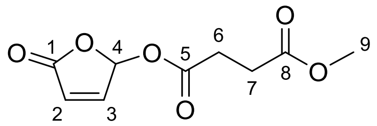

methyl (5-oxo-2,5-dihydrofuran-2-yl) succinate
Chemical Formula: $C_9H_{10}O_6$
Exact Mass: 214,0477
Molecular Weight: 214,1730

Compound **2h** was synthesized from **2g** (5.00 g, 25.0 mmol) and methanol (3.00 mL, 75.0 mmol) following **Method B**. The crude residue was purified by automatic column chromatography (80 g SiO_2 cartridge, 10-50% AcOEt/pentane over 20 CV), using DCM for liquid injection. Concentration of the collected fraction afforded pure **2h** as a light yellow oil (4.20 g, 19.6 mmol, 78% yield).

^{1}H NMR (400 MHz, $CDCl_3$) δ 7.33 (dd, J = 5.7, 1.4 Hz, 1H, H-3), 7.01 (t, J = 1.3 Hz, 1H, H-4), 6.32 (dd, J = 5.7, 1.2 Hz, 1H, H-2), 3.71 (s, 3H, H-9), 2.83-2.55 (m, 4H, H-6 and H-7).

^{13}C NMR (101 MHz, $CDCl_3$) δ 172.41 (C-8), 170.65 (C-5), 169.64 (C-1), 149.70 (C-3), 125.41 (C-2), 94.10 (C-4), 52.17 (C-9), 28.99, 28.58 (C-6 and C-7).

HRMS ESI-pos $[M+Na]^+$ $C_9H_{10}O_6Na$ calc. 237.0370, found 237.0360.

m.p. < –50°C.

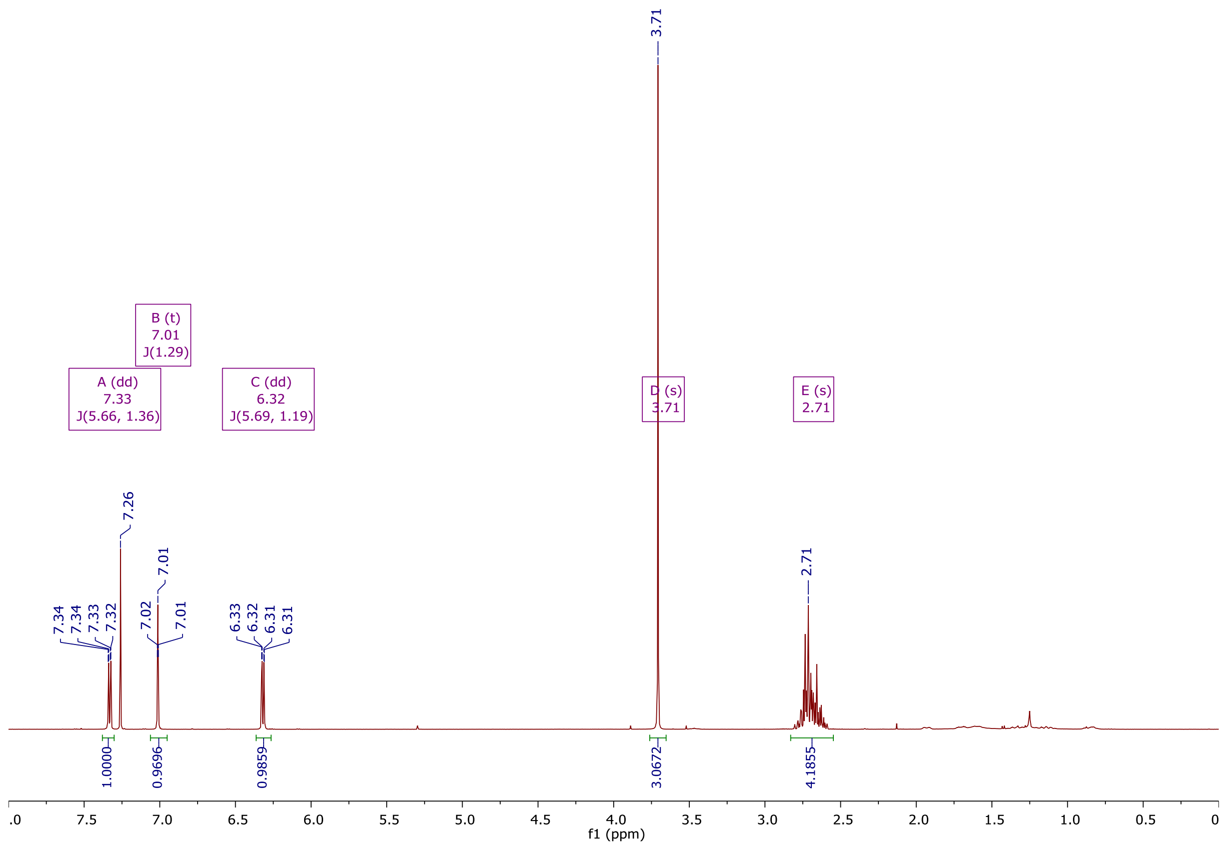
B (t)
7.01
J(1.29)
A (dd)
7.33
J(5.66, 1.36)
C (dd)
6.32
J(5.69, 1.19)
D (s)
3.71
E (s)
2.71
f1 (ppm)

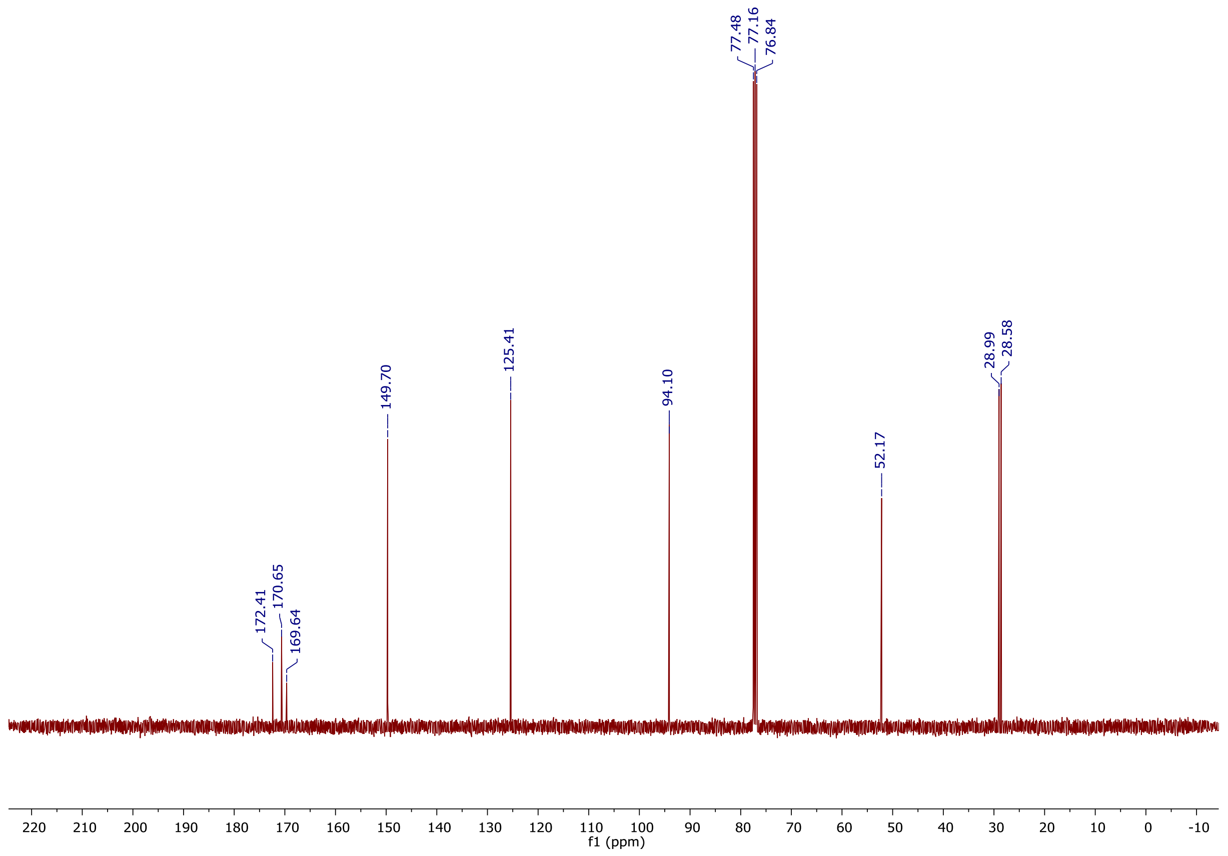
77.48
77.16
76.84
149.70
125.41
94.10
52.17
28.99
28.58
172.41
170.65
169.64
f1 (ppm)

Succinyloxy bis-butenolide (2i)

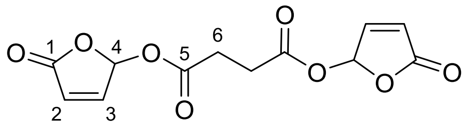

bis(5-oxo-2,5-dihydrofuran-2-yl) succinate
Chemical Formula: $C_{12}H_{10}O_8$
Exact Mass: 282,0376
Molecular Weight: 282,2040

Compound **2i** was synthesized from **2g** (2.00 g, 10.0 mmol) and hydroxy butenolide (2.00 eq., 2.00 g, 20.0 mmol) following **Method B**. The crude residue was purified by automatic column chromatography (80 g SiO_2 cartridge, 0-25% AcOEt/DCM over 20 CV), using solid injection with neutralized silica. Concentration of the collected fraction afforded pure **2i** as a white solid (1.91 g, 6.77 mmol, 68% yield).

^{1}H NMR (400 MHz, $CDCl_3$) δ 7.34 (ddd, J = 5.6, 3.4, 1.4 Hz, 2H, H-3), 7.00 (q, J = 1.4 Hz, 2H, H-4), 6.33 (dd, J = 5.7, 1.2 Hz, 2H, H-2), 2.90 – 2.65 (m, 4H, H-6).

^{13}C NMR (101 MHz, $CDCl_3$) δ 170.22, 170.15 (C-5), 169.55, 169.52 (C-1), 149.60, 149.53 (C-3), 125.53 (C-2), 94.15, 94.07 (C-4), 28.62, 28.62 (C-6 and C-7).

HRMS APPCI-neg $[M-H]^-$ $C_{12}H_9O_8$ calc. 281.0292, found 281.0304.

m.p. 116°C, fusion enthalpy 57 J/g.

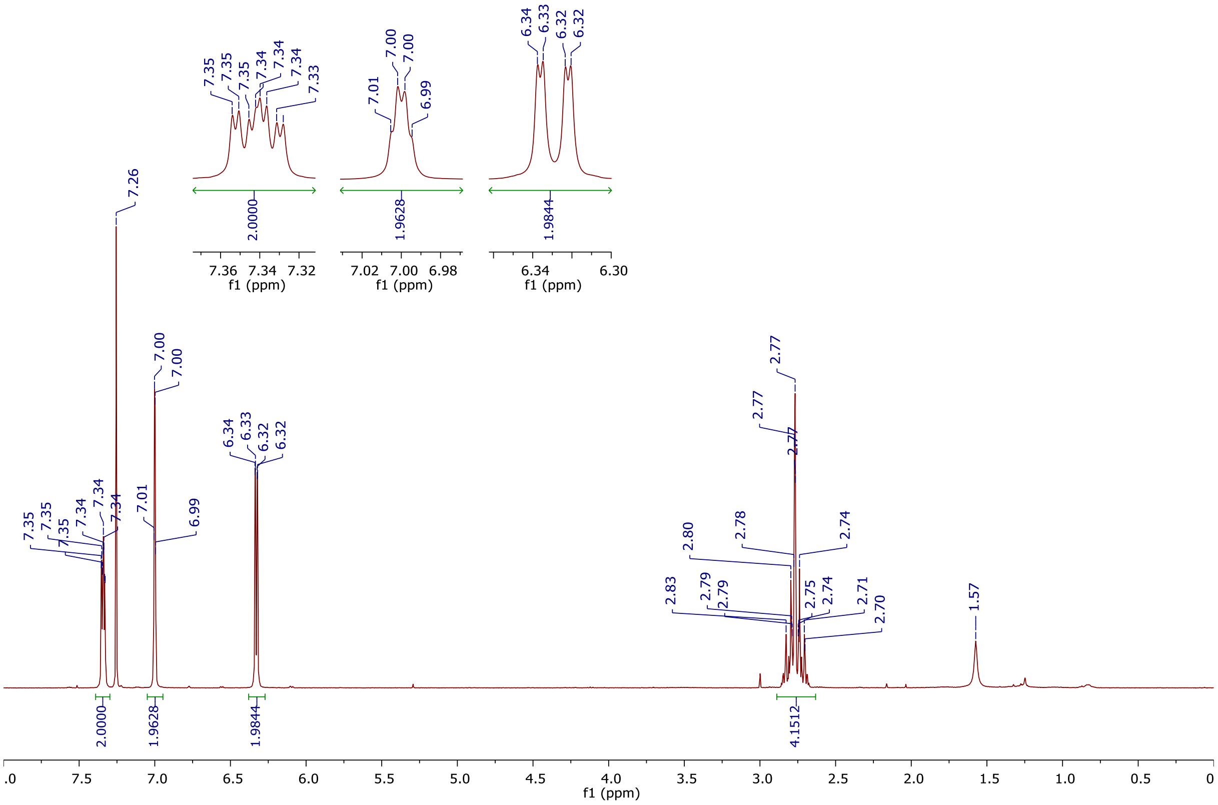
7.35
7.35
7.35
7.34
7.34
7.34
7.26
7.01
7.00
7.00
6.99
6.34
6.33
6.32
6.32
2.83
2.80
2.79
2.79
2.78
2.77
2.77
2.77
2.75
2.74
2.74
2.71
2.70
1.57
2.0000
1.9628
1.9844
4.1512
f1 (ppm)

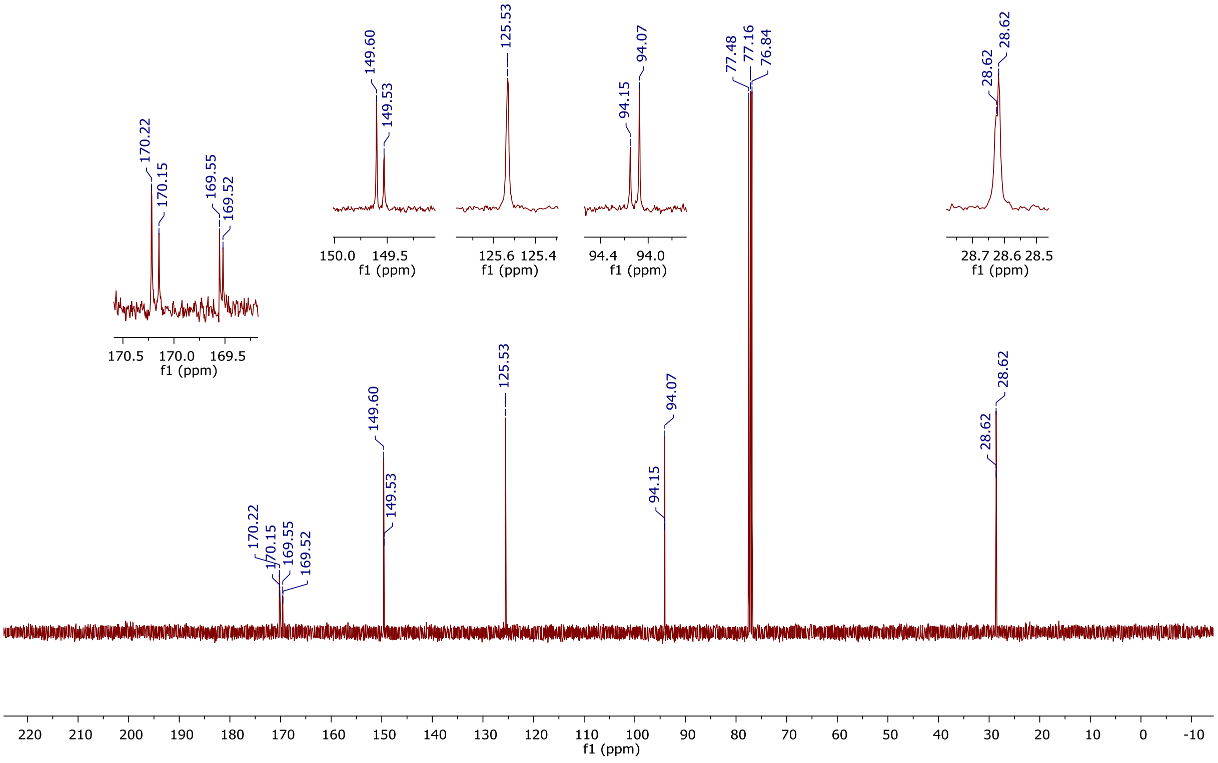
170.22
170.15
169.55
169.52
149.60
149.53
125.53
94.15
94.07
77.48
77.16
76.84
28.62
28.62
f1 (ppm)

Methyl carbonoxy butenolide (3a)

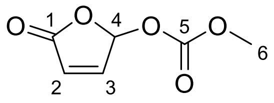

methyl (5-oxo-2,5-dihydrofuran-2-yl) carbonate
Chemical Formula: $C_6H_6O_5$
Exact Mass: 158.0215
Molecular Weight: 158.1090

Compound **3a** was synthesized from hydroxy butenolide (5.00 g, 50.0 mmol) and dimethyl dicarbonate following **Method A**. A gas release (CO_2) indicated occurrence of the reaction. The crude residue was purified by automatic column chromatography (80 g SiO_2 cartridge, 5-35% AcOEt/pentane over 20 CV), using DCM for liquid injection. Concentration of the collected fraction afforded **3a** as a colorless oil contaminated with methoxy butenolide **1a**, which was removed by rotary evaporation at high vacuum and 80°C. The pure **3a** was a colorless oil (4.85 g, 30.7 mmol, 61% yield).

^{1}H NMR (400 MHz, Chloroform-d) δ 7.32 (dd, J = 5.7, 1.3 Hz, 1H, H-3), 6.87 (t, J = 1.3 Hz, 1H, H-4), 6.34 (dd, J = 5.7, 1.2 Hz, 1H, H-2), 3.89 ppm (s, 3H, H-6).

^{13}C NMR (101 MHz, cdcl$_3$) δ 169.28 (C-1), 153.87 (C-5), 148.87 (C-3), 125.81 (C-2), 96.59 (C-4), 55.99 (C-6).

HRMS ESI-pos $[M+Na]^+$ $C_6H_6O_5Na$ calc. 181.0107, found 181.0111.

m.p. < –50°C.

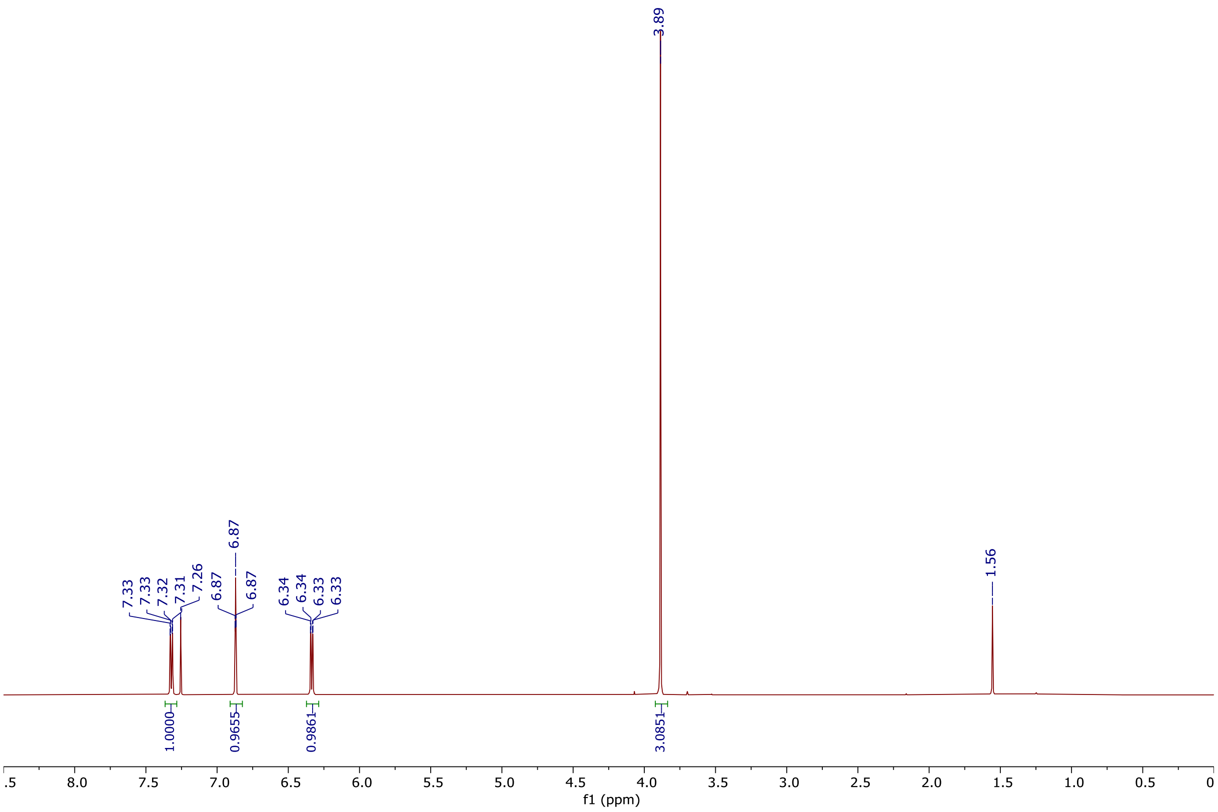
7.33
7.33
7.32
7.31
7.26
6.87
6.87
6.87
6.34
6.34
6.33
6.33
3.89
1.56
1.0000
0.9655
0.9861
3.0851
f1 (ppm)

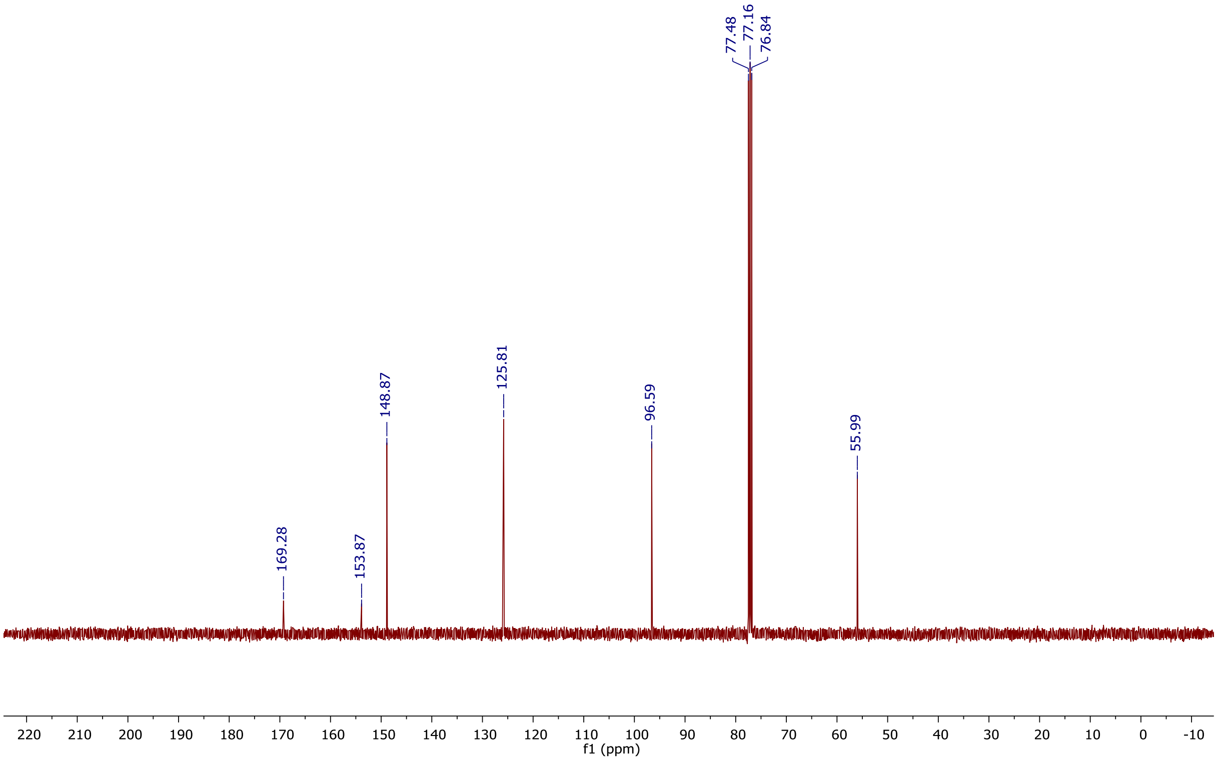
169.28
153.87
148.87
125.81
96.59
77.48
77.16
76.84
55.99
f1 (ppm)

Tert-butyl carbonoxy butenolide (3b)

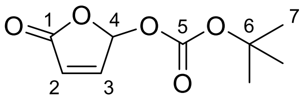

tert-butyl (5-oxo-2,5-dihydrofuran-2-yl) carbonate
Chemical Formula: $C_9H_{12}O_5$
Exact Mass: 200.0685
Molecular Weight: 200.1900

To a 50-mL round-bottom flask were added hydroxy butenolide **HB** (1.0 eq. 1.00 g, 10.0 mmol) and an olive-shaped stir bar. The flask was placed under vacuum and gently heated, causing bubbling (likely residual solvent and water removal). The flask was back-filled with N_2 and dry DCM (20.0 mL, [**HB**] = 0.5 M) was added. To the yellowish solution was added DMAP (25 mol%, 305 mg, 2.50 mmol), which dissolved rapidly. The solution was cooled to 0°C with an ice bath and Boc anhydride (1.5 eq., 3.4 mL, 15.0 mmol) was added dropwise over 5 min. No gas release was observed. The solution was stirred at 0°C for 1 h. The ice bath was removed, allowing the temperature to rise to room temperature. During the warming up process, an intense gas evolution suddenly occurred, along with a quick color change from yellowish to pink to red to brown. The gas release stopped after only a few minutes.

Sat. aq. NH_4Cl (25 mL) was added to the reaction mixture. The layers were separated and the organic one was washed once more with sat. aq. NH_4Cl (25 mL). The combined aqueous layers were extracted with DCM (25 mL). The combined organic extracts were washed with water (25 mL), dried with sodium sulfate, filtered and concentrated to a dark oil. The residue was purified by automatic column chromatography (40 g SiO_2 cartridge, 0-30 % AcOEt/pentane over 15 CV), using DCM for liquid injection. Concentration of the collected fraction afforded pure **3b** (1.06 g, 5.27 mmol, 53% yield) as a pale oil that solidified over night.

^{1}H NMR (400 MHz, $CDCl_3$) δ 7.30 (dd, J = 5.6, 1.4 Hz, 1H, H-3), 6.84 (t, J = 1.3 Hz, 1H, H-4), 6.31 (dd, J = 5.7, 1.2 Hz, 1H, H-2), 1.53 (s, 9H, H-7).

^{13}C NMR (101 MHz, $CDCl_3$) δ 169.57 (C-1), 151.20 (C-5), 149.24 (C-3), 125.60 (C-2), 96.04 (C-4), 84.98 (C-6), 27.74 (C-7).

HRMS APPCI-neg $[M-H]^-$ $C_9H_{11}O_5$ calc. 199.0601, found 199.0612.

m.p. 89°C, fusion enthalpy 96 J/g.

Decomposes above 120°C.

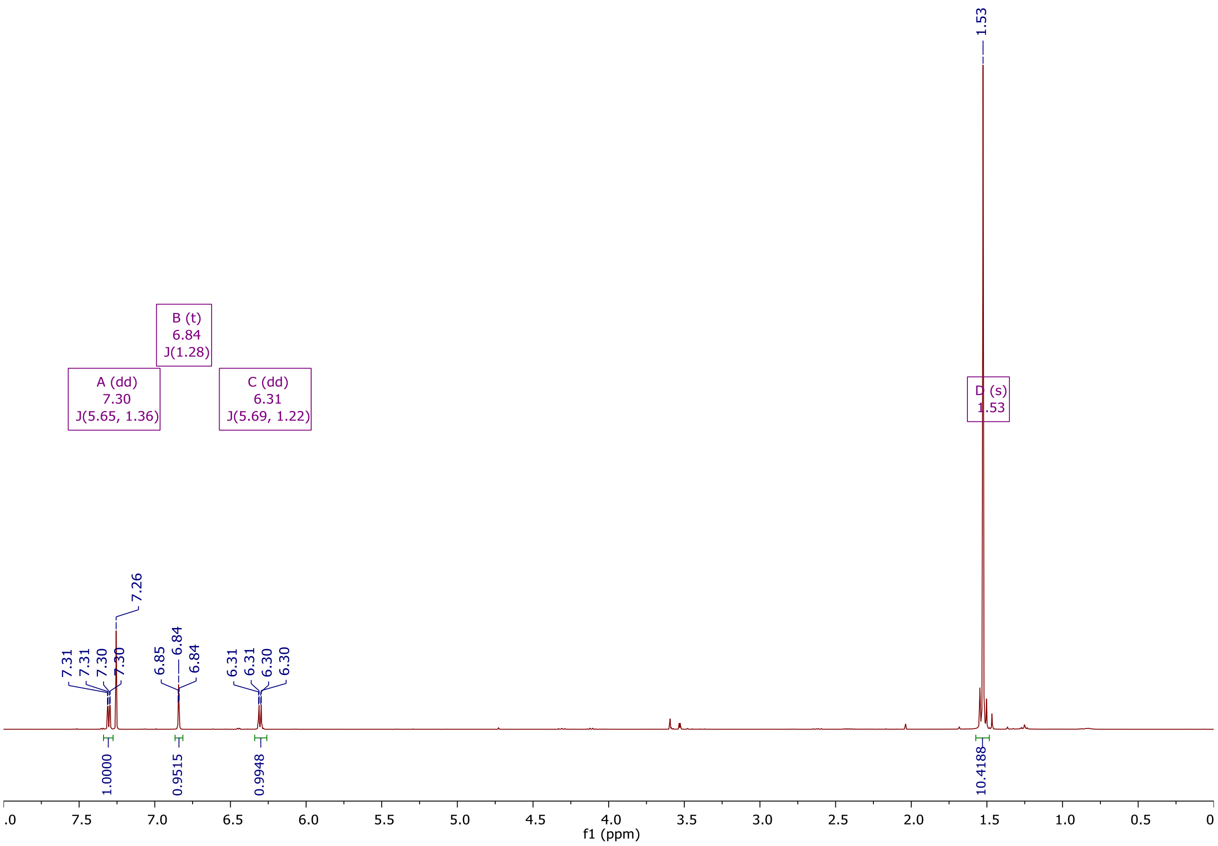
B (t)
6.84
J(1.28)
A (dd)
7.30
J(5.65, 1.36)
C (dd)
6.31
J(5.69, 1.22)
D (s)
1.53
7.31
7.31
7.30
7.30
7.26
6.85
6.84
6.84
6.31
6.31
6.30
6.30
1.53
1.0000
0.9515
0.9948
10.4188
f1 (ppm)

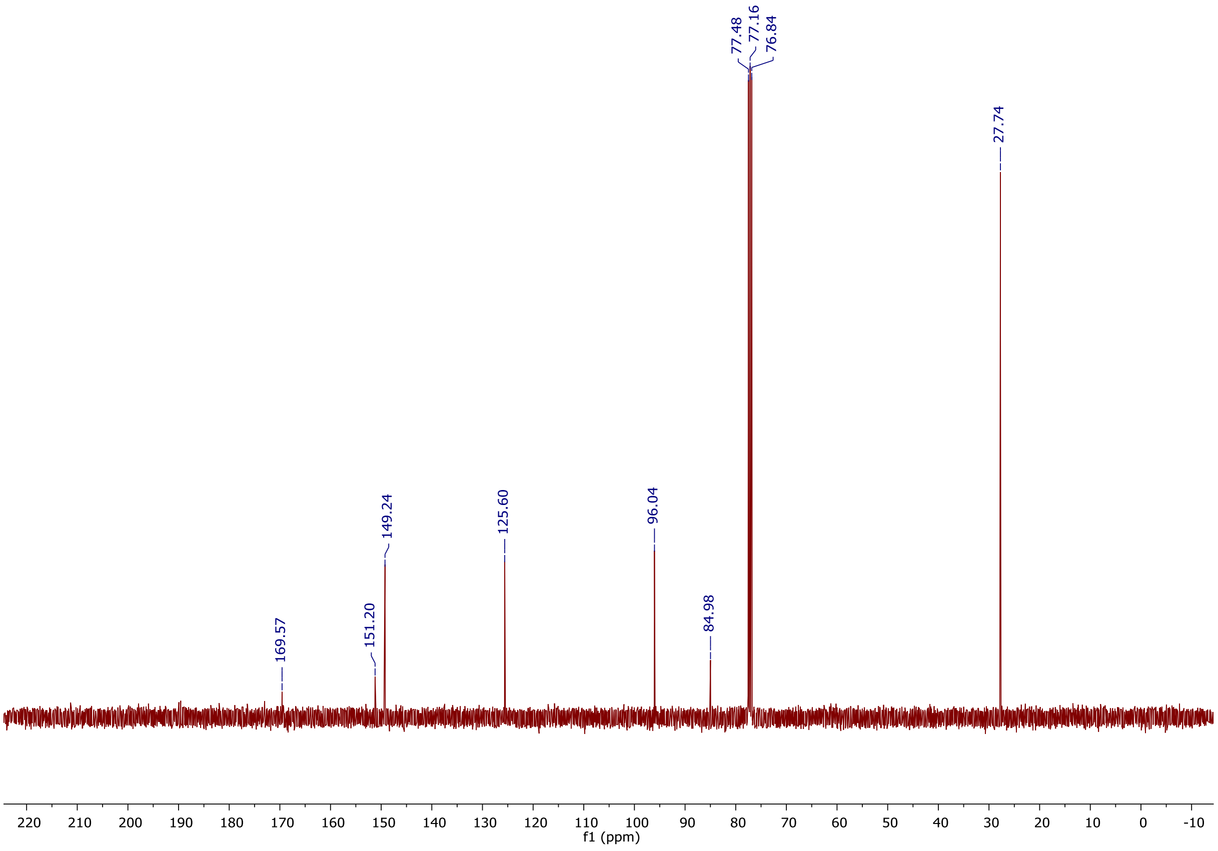
77.48
77.16
76.84
27.74
169.57
151.20
149.24
125.60
96.04
84.98
f1 (ppm)

Dodecyl carbamoxy butenolide (4a)

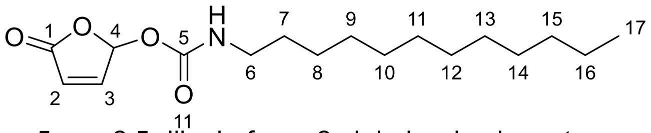

5-oxo-2,5-dihydrofuran-2-yl dodecylcarbamate
Chemical Formula: $C_{17}H_{29}NO_4$
Exact Mass: 311.2097
Molecular Weight: 311.4220

On small scale, **4a** was synthesized from hydroxy butenolide (500 mg, 5.00 mmol) and dodecyl isocyanate following **Method C**. The crude residue was purified twice by automatic column chromatography (25 g SiO_2 cartridge, 5-35% AcOEt/pentane over 40 CV), using DCM for liquid injection. Concentration of the collected fraction afforded **4a** as a white solid (502 mg, 1.61 mmol, 32% yield).

On larger scale, **4a** was synthesized from hydroxy butenolide (1.50 g, 15.0 mmol) and dodecyl isocyanate following **Method C**. When the crude mixture started to solidify upon concentration, the concentration was stopped and pentane (20 mL) was added. Upon sonication a yellowish paste was obtained, which was then filtered on a glass frit (P4). The yellowish paste in the glass frit was then resuspended in pentane (25 mL) and filtered again. This operation was repeated twice more until the filtrate was colorless. The off-white pasty solid was air-dried to a fluffy off-white powder, and then resuspended in Et_2O (200 mL). The hazy orange suspension was then filtered on a glass frit (P4) and the clear yellowish filtrate was concentrated to afford pure **4a** as an off-white powder (3.31 g, 10.6 mmol, 71% yield).

Two rotamers are detected on the timescale of the ^{1}H NMR experiment. They are noted *major* and *minor*. Both in $CDCl_3$ and DMSO-d_6, the ratio is around 10:1. In DMSO-$d_{6,}$ most peak but one overlap.

^{1}H NMR (400 MHz, $CDCl_3$) δ 7.33 (d, J = 5.5 Hz, 1H, H-3 minor), 7.28 (dd, J = 5.7, 1.3 Hz, 1H, H-3 major), 7.02 (s, 1H, H-4 minor), 7.00 (s, 1H, H-4 major), 6.30 (d, J = 6.2 Hz, 1H, H-2 minor), 6.27 (dd, J = 5.6, 1.2 Hz, 1H, H-2 major), 4.89 (br s, 1H, NH major), 4.71 (br s, 1H, NH minor), 3.22 (q, J = 6.8 Hz, 2H, H-6 major), 3.13 (q, J = 6.4, 6.0 Hz, 2H, H-6 minor), 1.52 (p, J = 6.9 Hz, 2H, H-7 both rotamers), 1.33 – 1.22 (m, 18H, H-8 to H-16 both rotamers), 0.87 (t, J = 6.8 Hz, 3H, H-17 both rotamers).

^{1}H NMR (400 MHz, DMSO-d_6) δ 7.78 – 7.64 (m, 2H, H-3 and NH), 6.95 (q, J = 1.4 Hz, 1H, H-4), 6.50 (dd, J = 5.7, 1.3 Hz, 1H, H-2), 3.01 (q, J = 6.6 Hz, 1H, H-6 major), 2.92 (m, 1H, H-6 minor), 1.40 (m, 2H, H-6), 1.30 – 1.14 (m, 18H, H-8 to H-16), 0.85 (t, J = 6.4 Hz, 3H, H-17).

^{13}C NMR (101 MHz, $CDCl_3$) δ 169.97 (C-1), 153.58 (C-5), 150.04 (C-3), 125.22 (C-2), 94.95 (C-4), 41.48 (C-6), 32.04 (C-7), 29.78, 29.76, 29.75, 29.70, 29.64, 29.47, 29.35, 26.81, 22.81, 14.25 (C-8 to C-17).

HRMS ESI-pos $[M+Na]^+$ $C_{17}H_{29}NO_4Na$ calc. 334.1989, found 334.1986.

m.p. 78°C, fusion enthalpy 106 J/g.

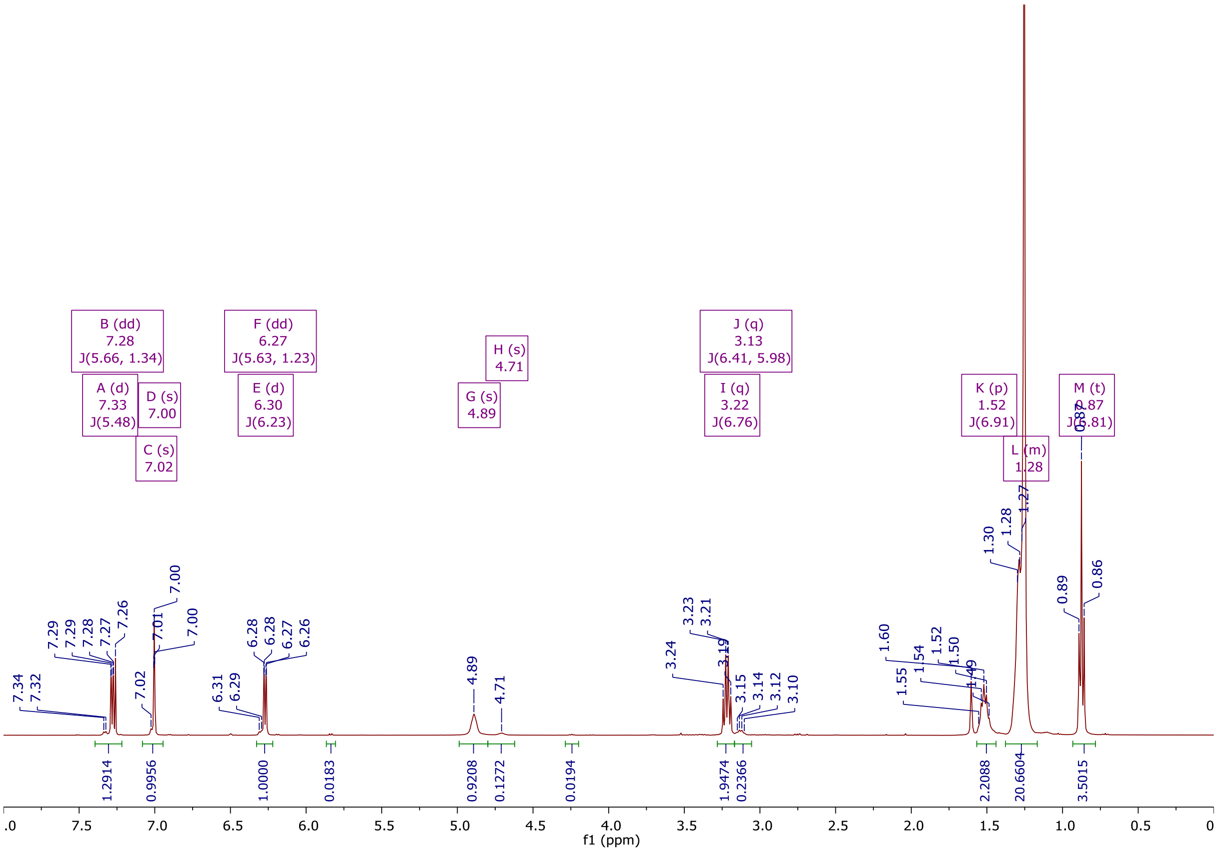
B (dd)
7.28
J(5.66, 1.34)
A (d)
7.33
J(5.48)
D (s)
7.00
C (s)
7.02
F (dd)
6.27
J(5.63, 1.23)
E (d)
6.30
J(6.23)
H (s)
4.71
G (s)
4.89
J (q)
3.13
J(6.41, 5.98)
I (q)
3.22
J(6.76)
K (p)
1.52
J(6.91)
M (t)
0.87
J(6.81)
L (m)
1.28
f1 (ppm)

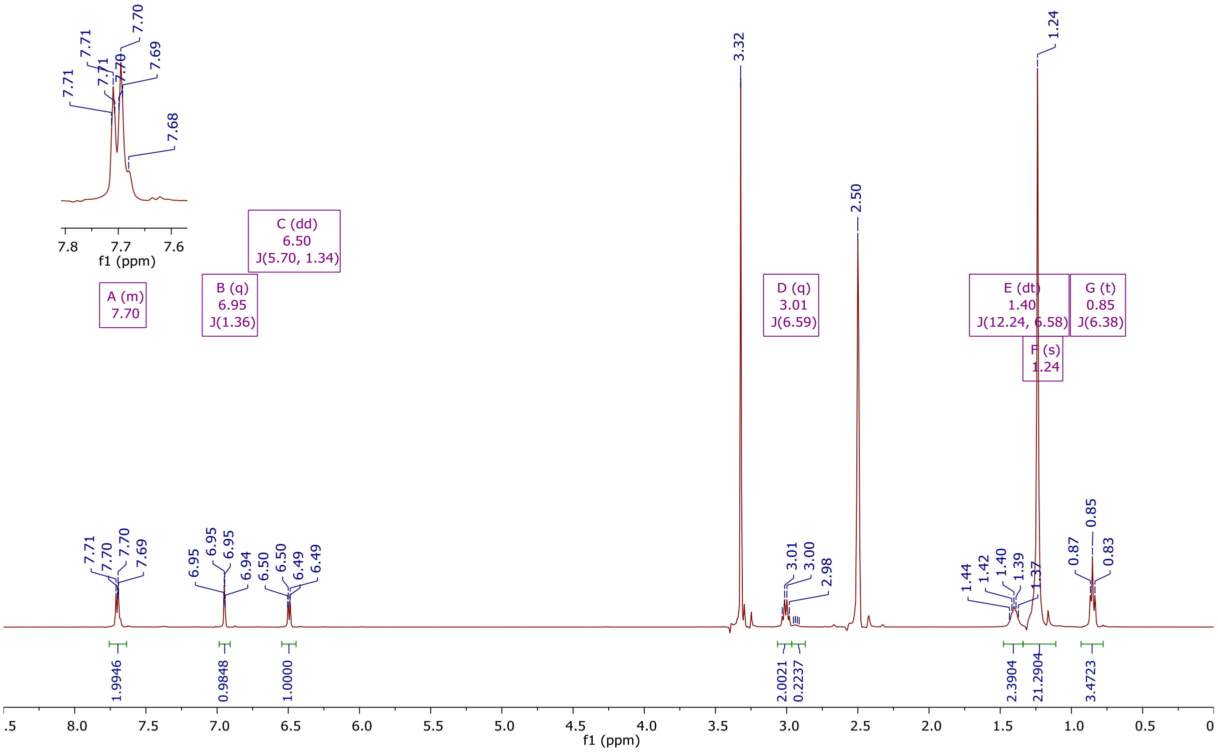
f1 (ppm)
C (dd)
6.50
J(5.70, 1.34)
A (m)
7.70
B (q)
6.95
J(1.36)
D (q)
3.01
J(6.59)
E (dt)
1.40
J(12.24, 6.58)
G (t)
0.85
J(6.38)
F (s)
1.24
f1 (ppm)

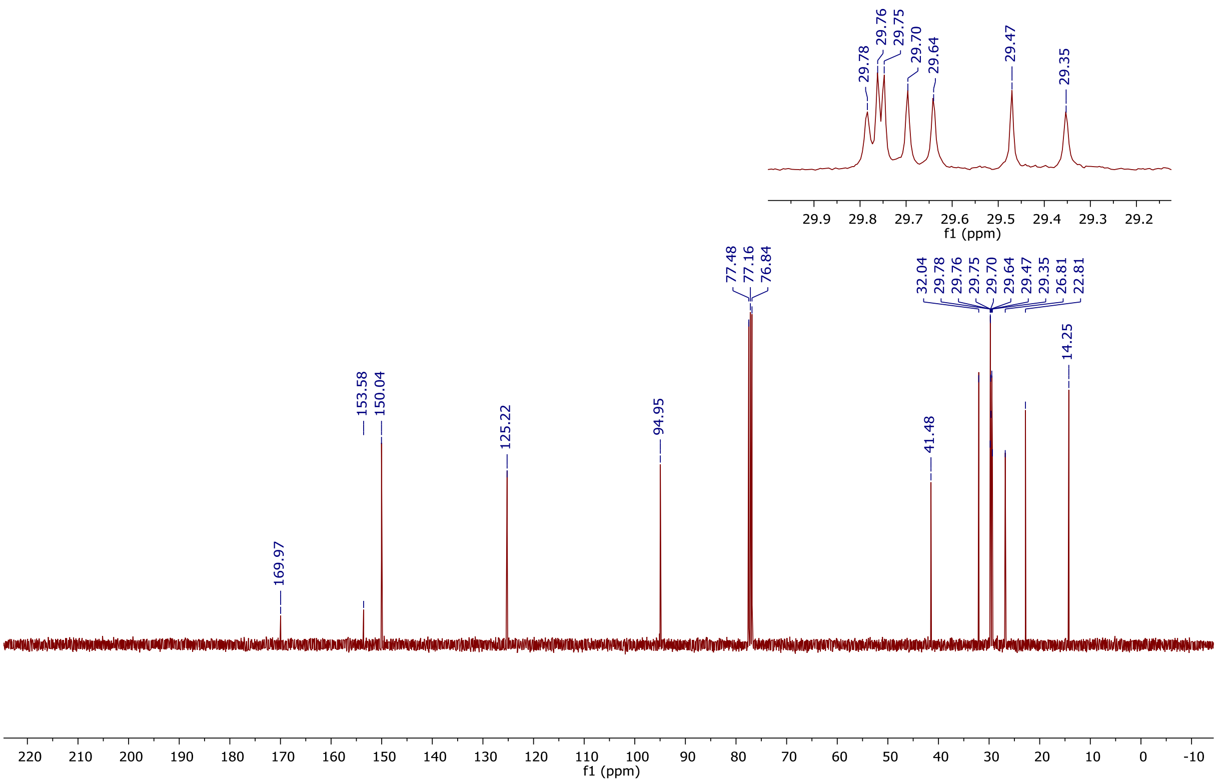
29.78
29.76
29.75
29.70
29.64
29.47
29.35
29.9 29.8 29.7 29.6 29.5 29.4 29.3 29.2
f1 (ppm)
77.48
77.16
76.84
32.04
29.78
29.76
29.75
29.70
29.64
29.47
29.35
26.81
22.81
14.25
153.58
150.04
125.22
94.95
41.48
169.97
220 210 200 190 180 170 160 150 140 130 120 110 100 90 80 70 60 50 40 30 20 10 0 -10
f1 (ppm)

Cyclohexyl carbamoxy butenolide (4b)

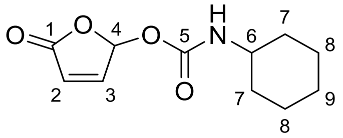

5-oxo-2,5-dihydrofuran-2-yl cyclohexylcarbamate
Chemical Formula: $C_{11}H_{15}NO_4$
Exact Mass: 225.1001
Molecular Weight: 225.2440

Compound **4b** was synthesized from hydroxy butenolide (5.00 g, 50.0 mmol) and cyclohexyl isocyanate following **Method C**. The crude residue was purified by automatic column chromatography (80 g SiO_2 cartridge, 10-50% AcOEt/pentane over 20 CV), using DCM for liquid injection. Concentration of the collected fraction afforded a yellow viscous oil containing fine white particles, which was resuspended in DCM (25 mL) and filtered on a glass frit (P4). Concentration of the filtrate afforded pure **4b** as a viscous yellow oil which solidified overnight into an off-white solid (9.52 g, 42.3 mmol, 85% yield).

Two rotamers are detected on the timescale of the ^{1}H NMR experiment in $CDCl_3$. They are noted *major* and *minor*. In $CDCl_3$, the ratio is around 10:1.

^{1}H NMR (400 MHz, $CDCl_3$) δ 7.33 (m, 1H, H-3 minor), 7.28 (dd, J = 5.6, 1.4 Hz, 1H, H-3 major), 7.04 (m, 1H, H-4 minor), 7.00 (s, 1H, H-4 major), 6.30 (m, 1H, H-2 minor), 6.27 (dd, J = 5.6, 1.2 Hz, 1H, H-2 major), 4.79 (d, J = 8.1 Hz, 1H, N-H major), 4.65 (m, 1H, N-H minor), 3.52 (m, 1H, H-6 major), 3.37 (m, 1H, H-6 minor), 1.95 (m, 2H, H-7, H-8 or H-9), 1.79-1.50 (m, 4H, H-7, H-8 or H-9), 1.44 – 0.98 (m, 4H, H-7, H-8 or H-9).

^{1}H NMR (400 MHz, DMSO-d_6) δ 7.71 (d, J = 5.7 Hz, 1H, H-3), 7.67 (d, J = 7.9 Hz, 1H, NH), 6.95 (s, 1H, H-4), 6.49 (dd, J = 5.7, 1.3 Hz, 1H, H-2), 3.31 (m, 1H, H-6), 1.77 (m, 2H, H-7 or H-8 or H-9), 1.67 (m, 2H, H-7 or H-8 or H-9), 1.54 (m, 2H, H-7, H-8 or H-9), 1.36-0.96 (m, 6H, H-7, H-8 or H-9).

^{13}C NMR (101 MHz, $CDCl_3$) δ 170.01 (C-1), 152.66 (C-5), 150.10 (C-3), 125.19 (C-2), 94.87 (C-4), 50.45 (C-6), 33.23 (C-7a), 33.16 (C-7b), 25.47 (C-9), 24.80 (C-8a), 24.77 (C-8b).

HRMS APPCI-neg $[M-H]^-$ $C_{11}H_{14}NO_4$ calc. 224.0917, found 224.0928.

m.p. 106°C, fusion enthalpy 114 J/g.

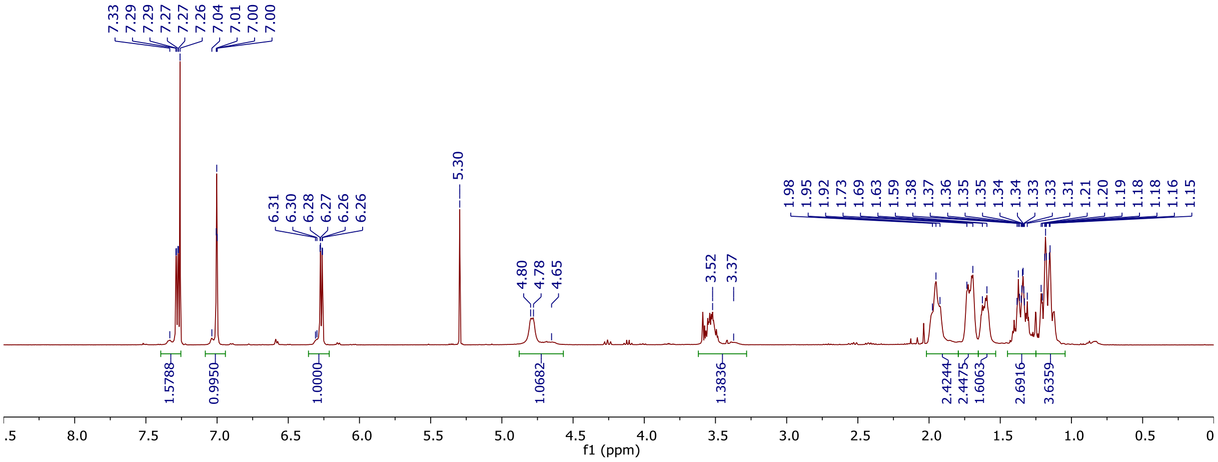
7.33 7.29 7.29 7.27 7.27 7.26 7.04 7.01 7.00 7.00
6.31 6.30 6.28 6.27 6.26 6.26
5.30
4.80 4.78 4.65
3.52 3.37
1.98 1.95 1.92 1.73 1.69 1.63 1.59 1.38 1.37 1.36 1.35 1.35 1.34 1.34 1.33 1.33 1.31 1.21 1.20 1.19 1.18 1.18 1.16 1.15
1.5788 0.9950 1.0000 1.0682 1.3836 2.4244 2.4475 1.6063 2.6916 3.6359
f1 (ppm)

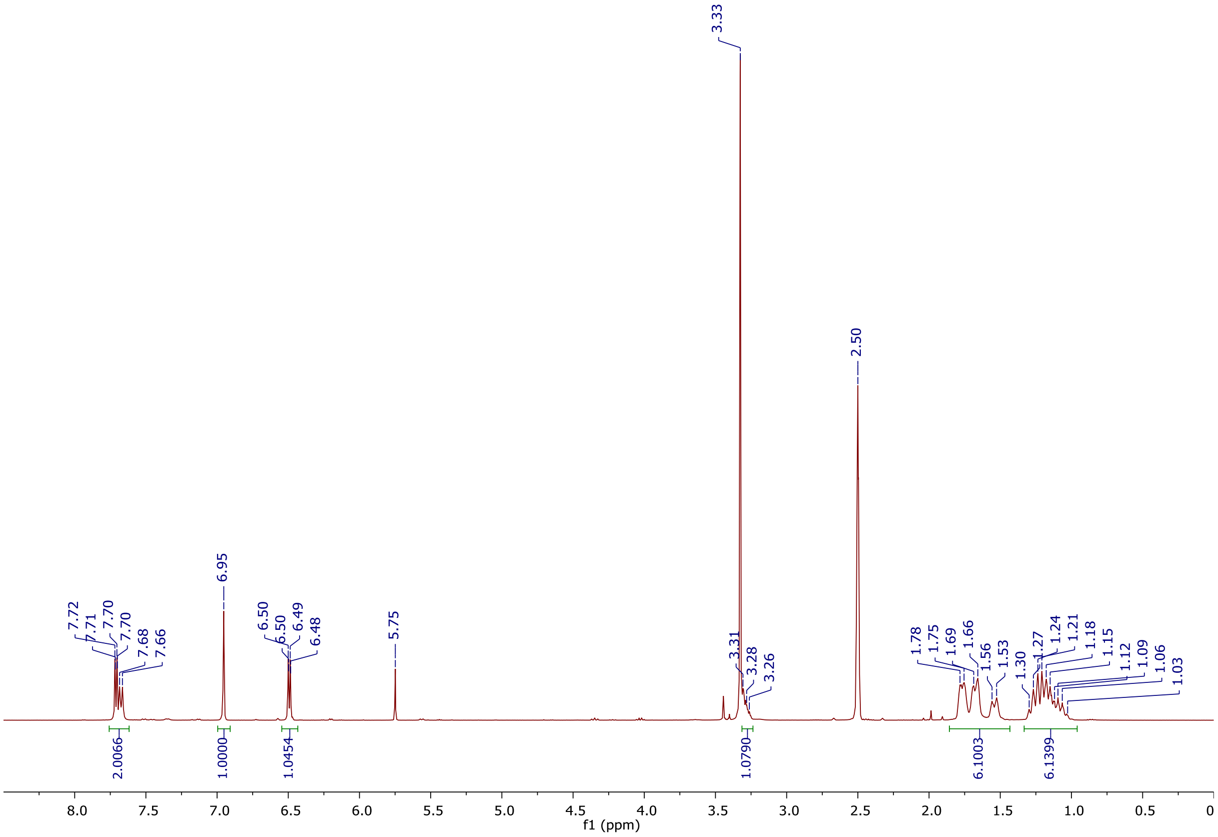
3.33
2.50
6.95
7.72 7.71 7.70 7.70 7.68 7.66
6.50 6.50 6.49 6.48
5.75
3.31 3.28 3.26
1.78 1.75 1.69 1.66 1.56 1.53 1.30 1.27 1.24 1.21 1.18 1.15 1.12 1.09 1.06 1.03
2.0066 1.0000 1.0454 1.0790 6.1003 6.1399
f1 (ppm)

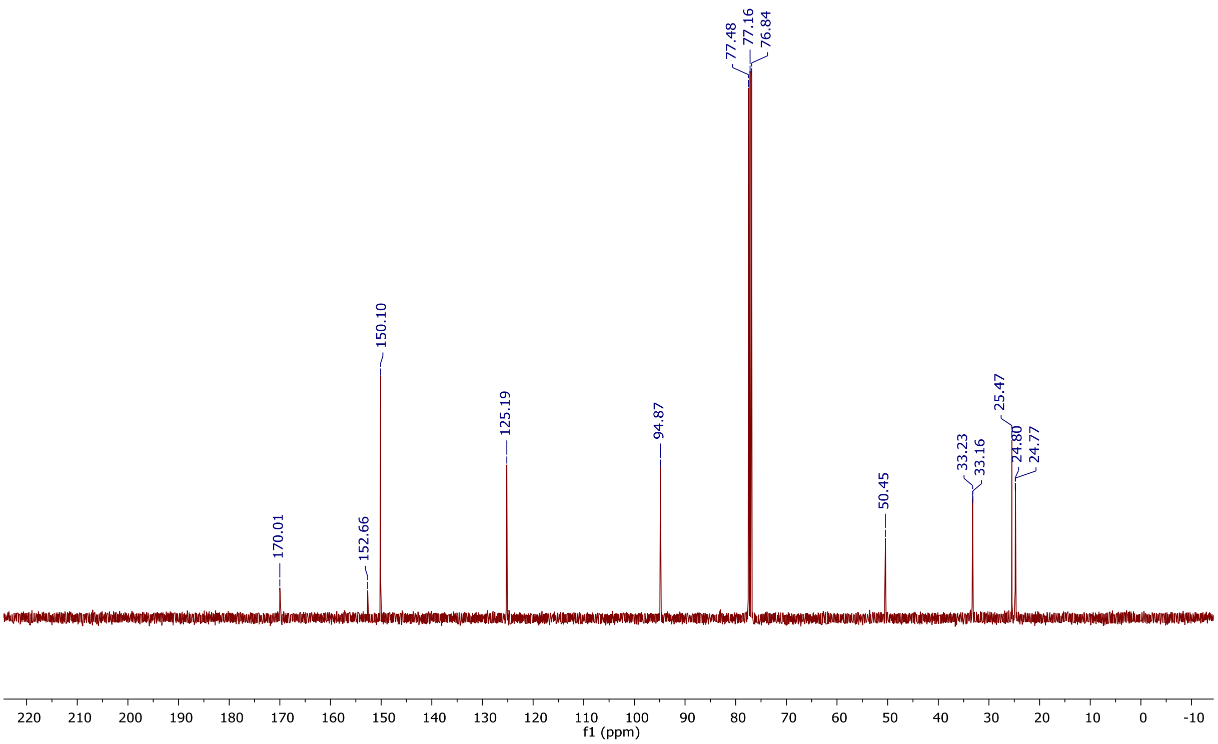

77.48
77.16
76.84
150.10
125.19
94.87
25.47
24.80
24.77
33.23
33.16
50.45
170.01
152.66
220 210 200 190 180 170 160 150 140 130 120 110 100 90 80 70 60 50 40 30 20 10 0 -10
f1 (ppm)

Methods

(Co)polymerizations for conversion and kinetic rate measurement

To a screw-cap 4-mL vial equipped with a 10-mm stir bar and a septum were added monomers (2 mmol in total), an internal standard (typically 1,3,5-trimethoxybenzene, 0.5 mmol) and a solvent (1-methoxypropan-2-ol, butyl acetate, NMP, gamma-valerolactone, or a mixture thereof, 500 µL, [monomers] = 4 M). The mixture was homogenized, briefly heating if needed (*e.g.* in case of insoluble monomers), and then a 20-40 µL sample was diluted in an NMR tube with $CDCl_3$ (500-600 µL) for reference. The vial was then closed and pre-heated at 120°C for 1-2 min. Trigonox 42S (60 µmol, 3 mol% versus monomers) was added to the hot mixture via a microsyringe through the septum, corresponding to t = 0. At various time points, 20-40 µL samples were taken from the reaction mixture with a microsyringe and diluted in an NMR tube with $CDCl_3$ (500-600 µL). All samples were analyzed by ^{1}H NMR on a 400 MHz spectrometer (typically D1 = 5, ns = 8). After correcting processed spectra for phase and baseline, integration and normalization of relevant peaks (one for each monomer) allowed monitoring of conversion. Rates of (co)polymerizations were calculated according to the method described below.

Note: The solvents and reagents were <u>not</u> degassed prior to experiments.

Note: We recommend to neutralize the $CDCl_3$ used in preparing the samples, *e.g.* by flushing it through a pad of basic alumina. This is to prevent side-reactions that occur *in the NMR tube after sampling* (and not in the reaction mixture), where vinylic moieties (*e.g.* of DVE or NVP) can undergo acid-catalyzed acetalization with nucleophiles (*e.g.* traces of water or an alcohol solvent):

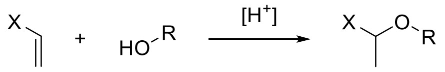

Copolymerizations with DVE for molecular weight distribution and glass transition temperature

To measure the molecular weight distribution (Mn, Mw and PDI) and glass transition temperature (T_g) of some (co)polymers prepared with DVE, copolymerizations were performed as described above, but <u>without internal standard</u>. The remaining solvent were removed by rotary evaporation under strong vacuum at 80°C. The dried polymers were then analyzed by gel permeation chromatography (GPC) in THF (25-50 mg/mL), with detection by refraction index against a polystyrene calibration. The glass transition temperature was measured by differential scanning calorimetry (DSC) on Heat-Cool-Heat cycles between temperatures ranging from –75°C to 150°C at 10°C/min. The T_g was determined from the 2nd heating ramp (*i.e.* the 3rd step).

Reaction kinetics

The rate of polymerization $\boldsymbol{R_p}$ has been determined for each (co)polymer by:

Equation S1. Rate of polymerization.

$$R_p = -\frac{dc_M}{dt} = k_p \cdot \left(f\frac{k_d}{k_t}\right)^{0,5} \cdot c_M \cdot (c_{ini})^{0.5}$$

In the case of copolymerization C_M (monomer concentration) was substituted for the sum of each monomer's concentration:

Equation S2. Substitution of C_M in copolymerization.

$$c_M = ([M_1] + [M_2])$$

Equation S1 is integrated from 0 to t giving:

Equation S3. Integration of rate of polymerization from 0 to t.

$$\ln\left(\frac{c_M^0}{c_M}\right) = k_p\left(f\frac{k_d}{k_t}c_{ini}\right)^{0.5} \cdot t$$

The conversion of (co)polymerization is determined by:

Equation S4. Conversion of polymer.

$$U = \frac{c_M^0 - c_M}{c_M^0}$$

Substituting ***U*** in **equation S3** and combining the constants and variables we obtain:

Equation S5. Rate of polymerization as a function of t.

$$\ln\left(\frac{1}{1-U}\right) = k_{obs} \cdot t$$

By plotting $\ln\left(\frac{1}{1-U}\right)$ against t (**equation S5**) it is possible to obtain the rate constant k_{obs} in s^{-1}. This rate constant is a combination of different parameters, which are specific to reaction and reaction conditions. It includes:

$k_p\left[\frac{L}{mol\ s}\right]$ = rate constant for propagation

$k_d[s^{-1}]$ = rate constant for dissociation

$k_t\left[\frac{L}{mol\ s}\right]$ = rate constant for termination

Additionally, it is assumed that the radical concentration c_{ini} is constant and does not change over time. A substance specific correction value *f* is included as well. Without specific determination of the different rate constants (k_p, k_d, k_t), the relative reactivity between the different butenolides and the reactivity between butenolides and commonly used monomers can be compared through the comparison of the value for k_{obs}, provided that these values are obtained under the same reaction conditions.

DFT calculations

All computational input files were prepared in GaussView 6.0 on a local Windows 10 terminal. Input files were then transferred to the Rijksuniversiteit Groningen Peregrine HPC cluster where DFT calculations were carried out using the Gaussian 16 (g16) suite of programs.

HOMO and LUMO energies for the S0 ground state of various carbonyl monomers (Figure 9B) were obtained at the MN15/Def2TZVPP/SMD=THF level of theory. Geometry optimization to a local minimum was carried out using the g16 opt command at the MN15 functional and Def2TZVPP basis set level of theory with implicit solvation using the Solvation Model based on Density (SMD = tetrahydrofuran),[1–3] and the energies reported were read from the g16 output file. Visualizations of the HOMO and LUMO orbitals of these optimized structures were extracted from their cube files containing atom density and position data of the ground state geometry. Cube files for the HOMO and LUMO of the shown carbonyl monomers were generated from the g16 cubegen utility from checkpoint files of the previously completed optimizations.

The DFT thermochemistry of radical propagation reactions (see table on page S50) was extrapolated from free energies of reactants, transition states and products calculated at the MN15/Def2TZVPP/SMD=THF level. Geometry optimization to either ground state S0 stationary or transition states (TS) using the g16 opt command at the MN15 functional and Def2TZVPP basis set level of theory with implicit solvation using the Solvation Model based on Density (SMD = tetrahydrofuran).[1–3] Transition state geometry inputs were the result of rational guess based on steric clashes expected during the stereochemical inversion event, or were the result of potential energy surface relaxed coordinate scans using the g16 scan command at the MN15/Def2SVP/SMD=THF level.

After optimization, frequency DFT calculations of all obtained optimized stationary point or transition state structures were carried out using the g16 freq command at the MN15/Def2TZVPP/SMD=THF level, to confirm that minima structures had zero imaginary frequencies and that transition states had a single imaginary frequency. All shown free energies (see table on page S50) are ZPE and thermally corrected and were obtained from the frequency calculations. All shown free energies are obtained at 298.15 K and 1 atm, and reported in Hartrees, and also converted and reported to kcal/mol.

References:

[1] H. S. Yu, X. He, S. L. Li, D. G. Truhlar, *Chem. Sci.* **2016**, *7*, 5032–5051.

[2] J. Zheng, X. Xu, D. G. Truhlar, *Theor. Chem. Acc.* **2011**, *128*, 295–305.

[3] A. V. Marenich, C. J. Cramer, D. G. Truhlar, *J. Phys. Chem. B* **2009**, *113*, 6378–6396.

Data summary

Summary of (co)polymerization data

Entry	Butenolide (or equiv.)	Comonomer	(% but.)	Solvent	Conv. Butenolide	Conv. Comonomer	Combined conv.	k_{ini} (10^{-3} s^{-1})
1	***1a***	*DVE*	*50%*	*AcOBu*	*99%*	*92%*	***95%***	*0.61*
2	***1a***	*DVE*	*50%*	*AcOBu*	*99%*	*80%*	***89%***	*0.88*
3	***1a***	*DVE*	*50%*	*AcOBu*	*99%*	*80%*	***90%***	*0.87*
**4 (ave. 1–3)**	**1a**	DVE	50%	AcOBu	99% ± 0%	84% ± 7%	**91%** ± 3%	0.79 ± 0.15
**5**	**1a**	DVE	50%	GVL	100%	100%	**100%**	1.14
**6**	**1a**	DVE	50%	Et_2CO_3	100%	73%	**87%**	0.70
**7**	**1a**	DVE	50%	1M2P	100%	90%	**95%**	1.44
**8**	**1b**	DVE	50%	AcOBu	100%	92%	**96%**	1.04
9	***2a***	*DVE*	*50%*	*AcOBu*	*100%*	*97%*	***98%***	*2.25*
10	***2a***	*DVE*	*50%*	*AcOBu*	*100%*	*88%*	***94%***	*2.10*
11	***2a***	*DVE*	*50%*	*AcOBu*	*100%*	*87%*	***93%***	*2.03*
**12 (ave. 9–11)**	**2a**	DVE	50%	AcOBu	100% ± 0%	91% ± 7%	**95%** ± 3%	2.13 ± 0.11
**13**	**2a**	DVE	50%	1M2P	100%	85%	**93%**	3.39
**14**	**2b**	DVE	50%	AcOBu	100%	98%	**99%**	2.55
**15**	**2c**	DVE	50%	AcOBu	100%	99%	**99%**	2.59
**16**	**2d**	DVE	50%	AcOBu	98%	93%	**96%**	3.68
**17**	**2d**	DVE	50%	1M2P	99%	94%	**97%**	4.48
**18**	**2e**	DVE	50%	AcOBu	99%	97%	**98%**	2.66
**19**	**2e**	DVE	50%	1M2P	99%	89%	**94%**	3.08
**20**	**2f**	DVE	50%	AcOBu	99%	88%	**94%**	0.77
**21**	**2h**	DVE	50%	AcOBu	100%	98%	**99%**	3.25
**22**	**2i**	DVE	50%	AcOBu/NMP 4:1	*CROSSLINKING, gelation time = 3 min*			
**23**	**3a**	DVE	50%	AcOBu	100%	83%	**91%**	2.35
**24**	**3b**	DVE	50%	AcOBu	100%	88%	**94%**	1.75
**25**	**4a**	DVE	50%	AcOBu	99%	75%	**87%**	1.49
**26**	**4b**	DVE	50%	AcOBu	97%	98%	**98%**	1.38
**27**	–	DVE	0%	AcOBu	–	40%	**40%**	0.16
**28**	–	NVP	0%	NMP	–	99%	**99%**	6.29
**29**	**1a**	DVE	25%	AcOBu	97%	53%	**64%**	0.69
**30**	**1a**	DVE	75%	AcOBu	69%	100%	**77%**	0.44
**31**	**1a**	–	100%	AcOBu	17%	–	**17%**	0.05
**32**	**1a**	NVP	25%	NMP	99%	99%	**99%**	7.51
**33**	**1a**	NVP	50%	NMP	100%	99%	**99%**	7.24
**34**	**1a**	NVP	75%	NMP	79%	99%	**84%**	4.14
**35**	**1a**	–	100%	NMP	28%	–	**28%**	0.19
**36**	**1a**	NVP	50%	1M2P	100%	100%	**100%**	2.99
**37**	**2a**	DVE	25%	AcOBu	100%	51%	**63%**	1.33
**38**	**2a**	DVE	75%	AcOBu	70%	100%	**78%**	1.31
**39**	**2a**	–	100%	AcOBu	15%	–	**15%**	0.05
**40**	**2a**	NVP	25%	NMP	98%	98%	**98%**	11.2
**41**	**2a**	NVP	50%	NMP	99%	98%	**99%**	16.7
**42**	**2a**	NVP	75%	NMP	84%	96%	**87%**	6.66

**43**	**2a**	–	100%	NMP	30%	–	**30%**	0.22
**44**	**2a**	EGVE	50%	NMP	99%	86%	**92%**	11.1
**45**	**2a**	VeoVa-10	50%	AcOBu	90%	97%	**94%**	0.73
**46**	**2a**	NVC	50%	NMP	97%	93%	**95%**	4.85
**47**	**2a**	butyl acrylate	50%	AcOBu	30%	100%	**65%**	1.14
**48**	**2a**	butyl methacrylate	50%	AcOBu	16%	97%	**57%**	0.39
**49**	**2a**	styrene	50%	AcOBu	21%	97%	**59%**	0.34
**50**	butyl acrylate	DVE	50%	AcOBu	100%	66%	**83%**	4.73
**51**	2(5*H*)-furanone	DVE	50%	AcOBu	41%	37%	**39%**	0.20
**52**	Methyl furanone	DVE	50%	AcOBu	24%	22%	**23%**	0.08
**53**	Methoxy Methyl butenolide	DVE	50%	AcOBu	63%	60%	**61%**	0.20
**54**	Maleic anhydride	DVE	50%	AcOBu	100%	100%	**100%**	52.60

Summary of DFT results for radical propagation reactions

Radical	Energy	Monomer	Energy	Total		Product	Energy	ΔG (kcal/mol)	TS energy	ΔG‡ (kcal/mol)
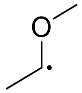	-193.463984	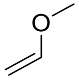	-192.906332	-386.370316	→	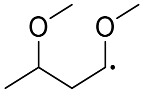	-386.382824	**-7.8**	-386.340831	**+18.5**
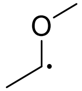	-193.463984	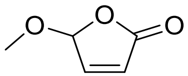	-419.441938	-612.905922	→	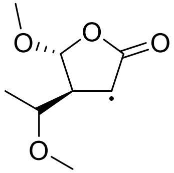	-612.918115	**-7.7**	-612.884866	**+13.2**
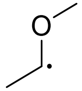	-193.463984	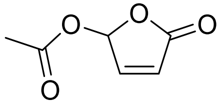	-532.727121	-726.191105	→	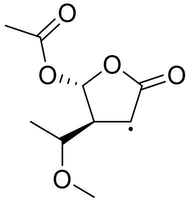	-726.206964	**-10.0**	-726.174076	**+10.7**
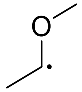	-193.463984	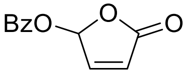	-724.251278	-917.715262	→	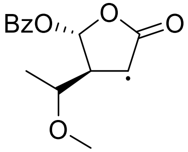	-917.730925	**-9.8**	-917.699258	**+10.0**

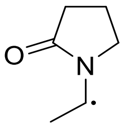	-364.197703	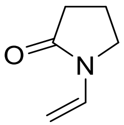	-363.635294	-727.832997	→	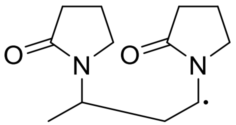	-727.842537	**-6.0**	-727.805699	**+17.1**
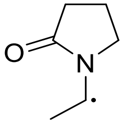	-364.197703	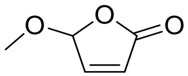	-419.441938	-783.639641	→	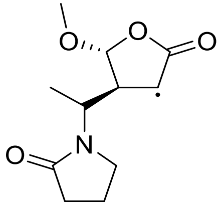	-783.641759	**-1.3**	-783.610911	**+18.0**
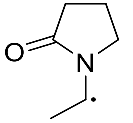	-364.197703	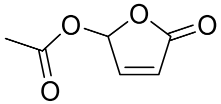	-532.727121	-896.924824	→	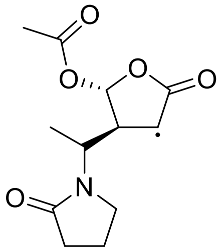	-896.930241	**-3.4**	-896.900512	**+15.3**
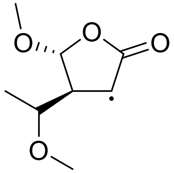	-612.918115	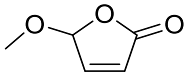	-419.441938	-1032.360053	→	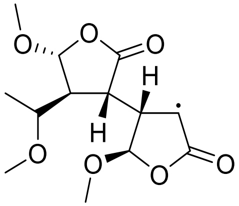	-1032.369776	**-6.1**	-1 032.333018	**+17.0**
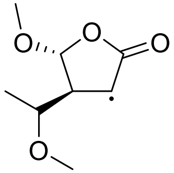	-612.918115	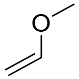	-192.906332	-805.824447	→	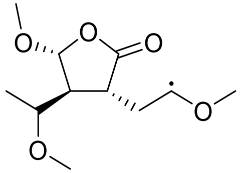	-805.835913	**-7.2**	-805.801188	**+14.6**

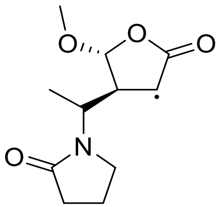	-783.641759	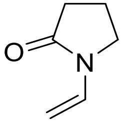	-363.635294	-1147.277053	→	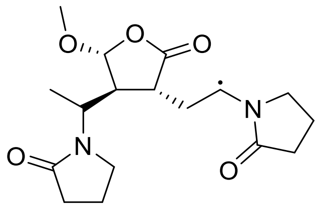	-1 147.284728	**-4.8**	-1 147.252871	**+15.2**
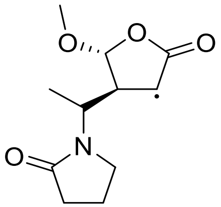	-783.641759	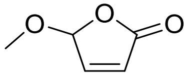	-419.441938	-1203.083697	→	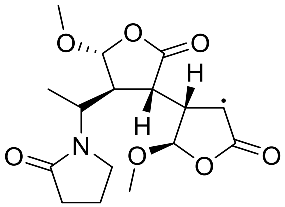	-1203.087675	**-2.5**	-1 203.052246	**+19.7**
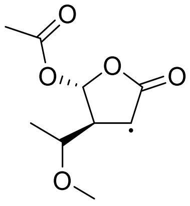	-726.206964	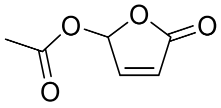	-532.727121	-1 258.934085	→	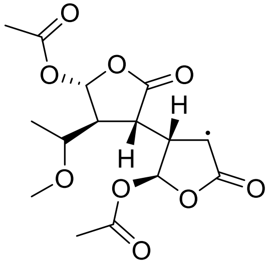	-1 258.943300	**-5.8**	-1258.905372	**+18.0**
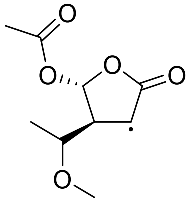	-726.206964	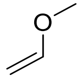	-192.906332	-919.113296	→	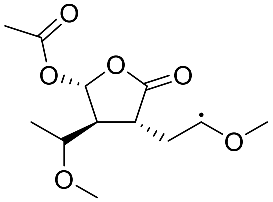	-919.124927	**-7.3**	-919.087969	**+15.9**

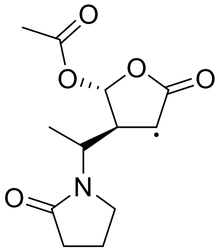	-896.930241	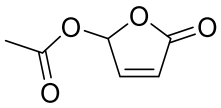	-532.727121	-1 429.657362	→	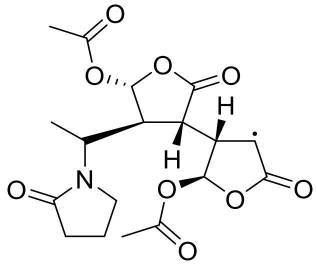	-1 429.662339	**-3.1**	-1 429.625275	**+20.1**
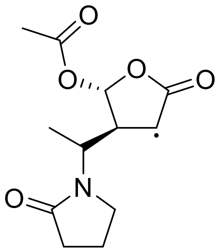	-896.930241	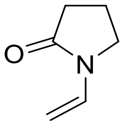	-363.635294	-1 260.565535	→	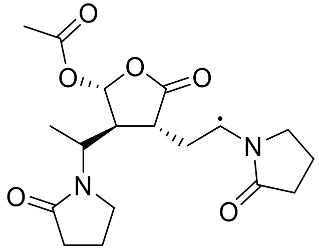	-1 260.574628	**-5.7**	-1 260.541455	**+15.1**

(Co)polymerization data

Methoxy butenolide (1a) and DVE in AcOBu – replicate 1

Poly(1a-co-DVE)					k1(obs)	6.10 . 10^-4 [s^-1]
	1a		Dodecyl vinyl ether		k2(obs)	n/a
Time [s]	Integral	Concentration [mol/L]	Integral	Concentration [mol/L]	Conversion	ln(1/[1-conversion])
0	1.02	2.00	0.95	2.00	0%	0.00
60	0.98	1.92	0.86	1.80	7%	0.07
120	0.97	1.89	0.84	1.76	9%	0.09
240	0.94	1.84	0.80	1.67	12%	0.13
480	0.86	1.68	0.73	1.52	20%	0.22
720	0.75	1.47	0.64	1.35	30%	0.35
960	0.63	1.23	0.57	1.19	40%	0.50
1440	0.42	0.82	0.42	0.89	57%	0.85
2160	0.21	0.42	0.29	0.60	75%	1.37
3120	0.09	0.17	0.19	0.40	86%	1.94
7200	0.01	0.02	0.08	0.17	95%	3.06

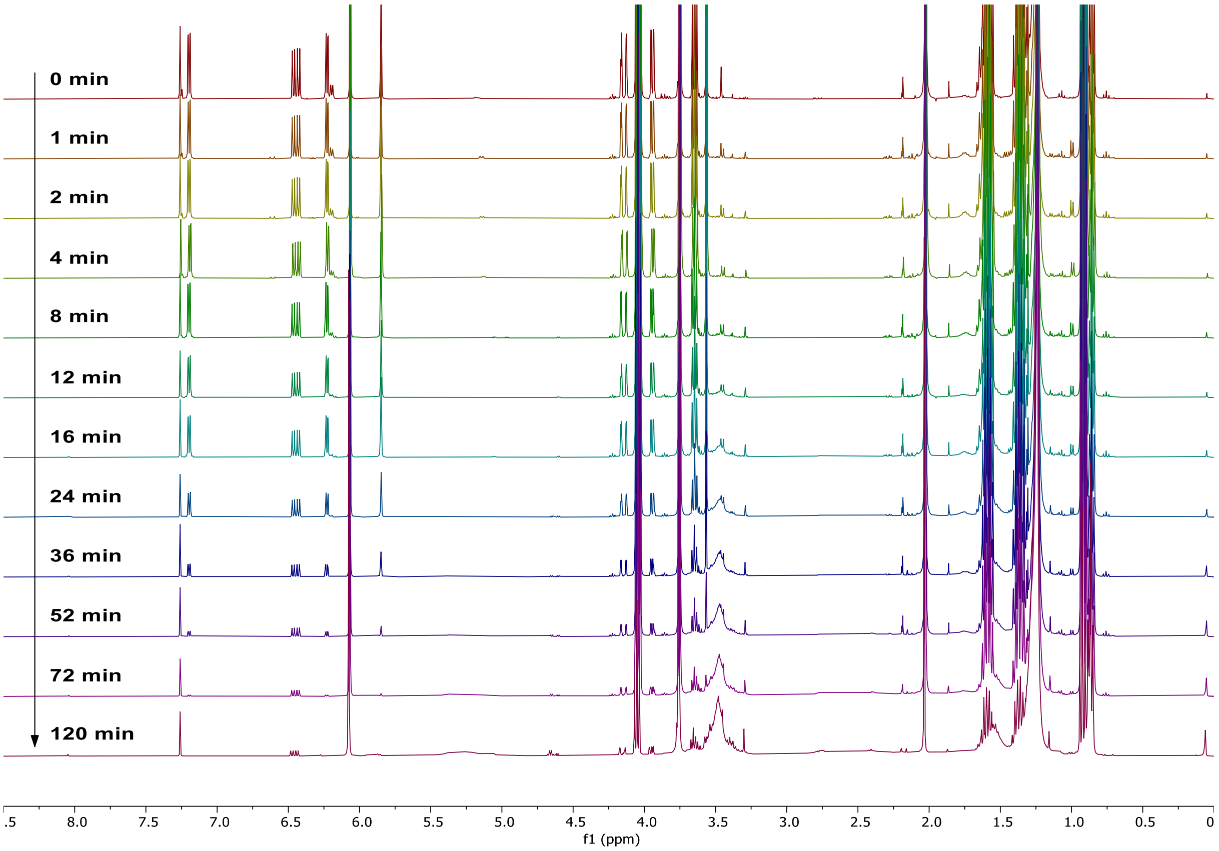

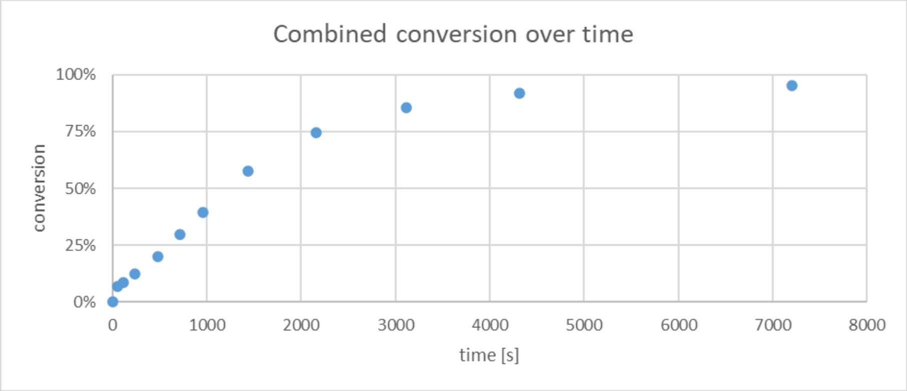
Combined conversion over time
conversion
100%
75%
50%
25%
0%
0
1000
2000
3000
4000
5000
6000
7000
8000
time [s]

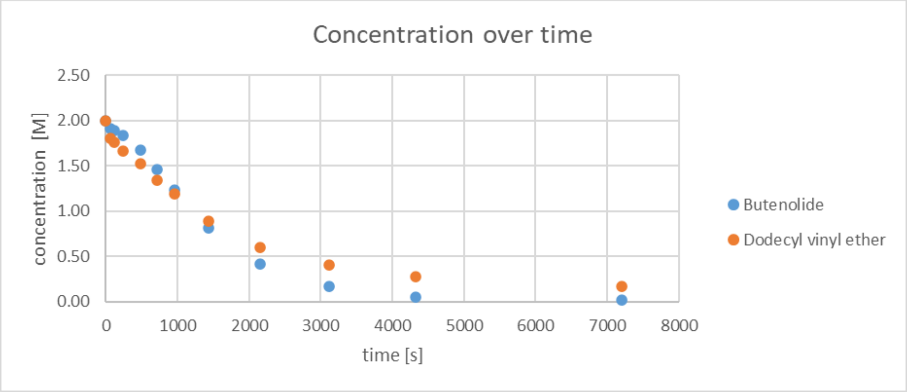
Concentration over time
concentration [M]
2.50
2.00
1.50
1.00
0.50
0.00
0
1000
2000
3000
4000
5000
6000
7000
8000
time [s]
Butenolide
Dodecyl vinyl ether

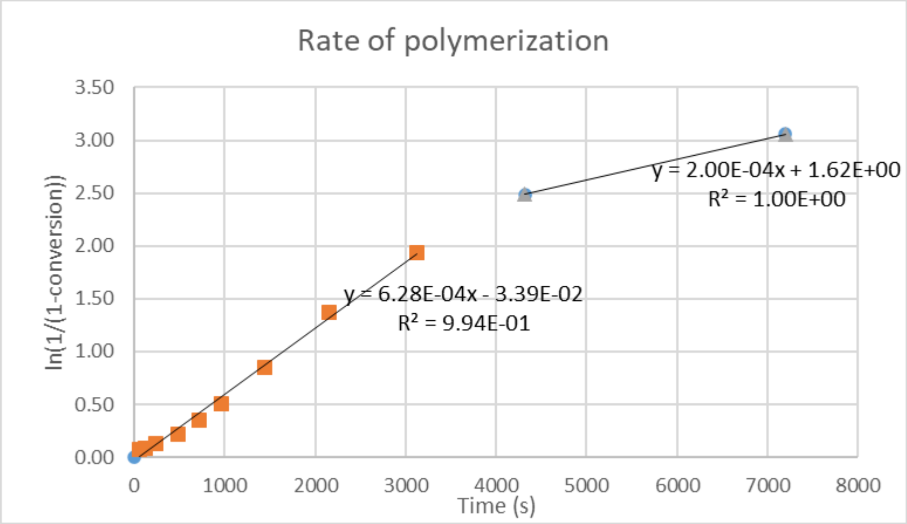
Rate of polymerization
ln(1/(1-conversion))
3.50
3.00
2.50
2.00
1.50
1.00
0.50
0.00
0
1000
2000
3000
4000
5000
6000
7000
8000
Time (s)
y = 2.00E-04x + 1.62E+00
R² = 1.00E+00
y = 6.28E-04x - 3.39E-02
R² = 9.94E-01

Methoxy butenolide (1a) and DVE in AcOBu – replicate 2

Poly(1a-co-DVE) in AcOBu					k1(obs)	8.78 x 10^{-4} [s^{-1}]
	1a		Dodecyl vinyl ether		k2(obs)	1.38 x 10^{-4} [s^{-1}]
Time [s]	Integral	Concentration [mol/L]	Integral	Concentration [mol/L]	Conversion	ln(1/[1-conversion])
0	0.90	2.00	0.97	2.00	0%	0.00
120	0.86	1.92	0.93	1.92	4%	0.04
240	0.81	1.79	0.88	1.82	10%	0.10
480	0.63	1.40	0.76	1.57	26%	0.30
960	0.34	0.75	0.55	1.14	53%	0.75
1800	0.08	0.17	0.35	0.73	77%	1.49
7200	0.01	0.02	0.20	0.41	89%	2.23

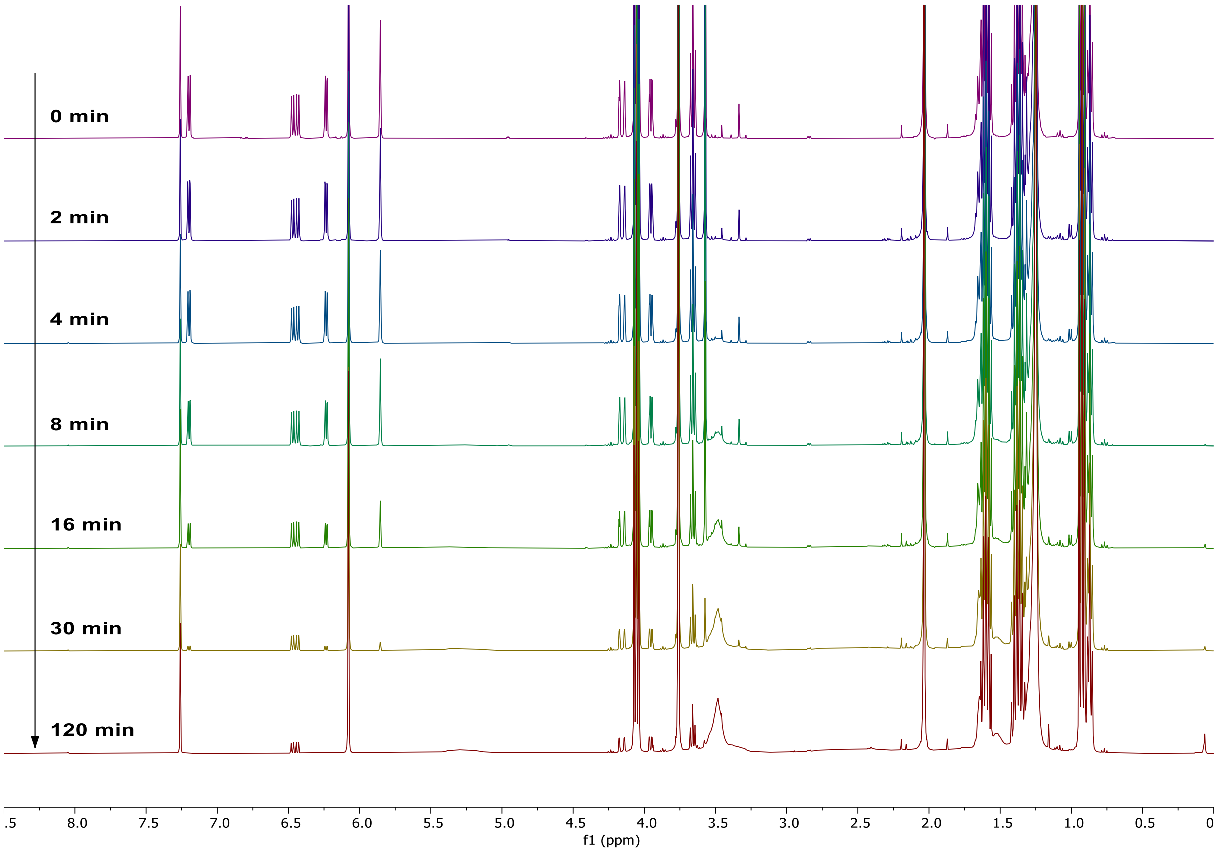

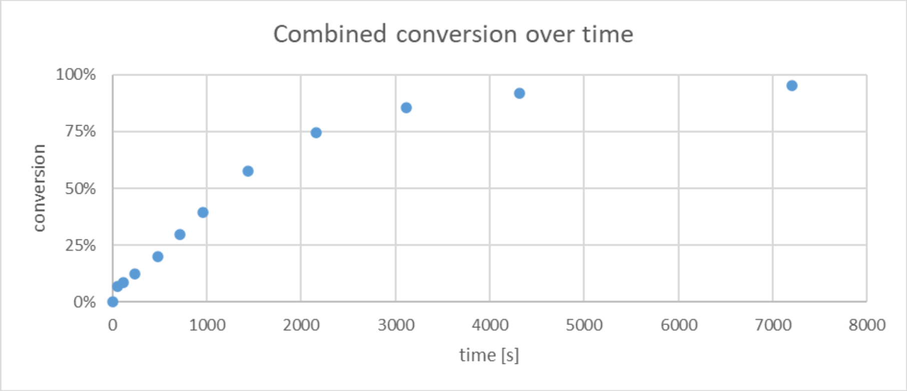
Combined conversion over time
100%
75%
50%
25%
0%
conversion
0
1000
2000
3000
4000
5000
6000
7000
8000
time [s]

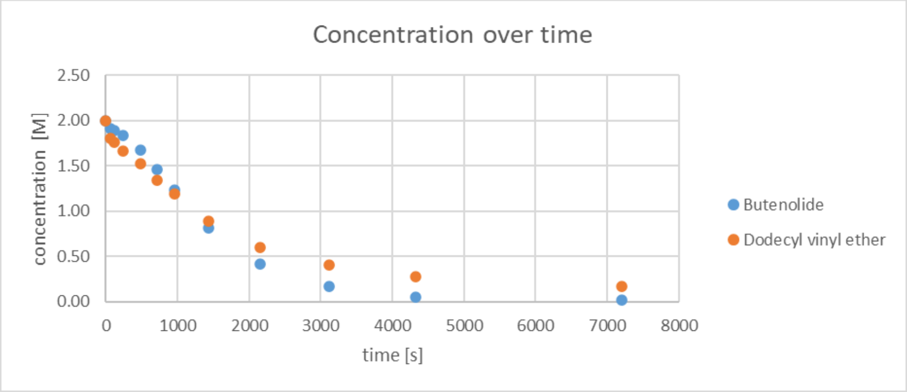
Concentration over time
2.50
2.00
1.50
1.00
0.50
0.00
concentration [M]
0
1000
2000
3000
4000
5000
6000
7000
8000
time [s]
Butenolide
Dodecyl vinyl ether

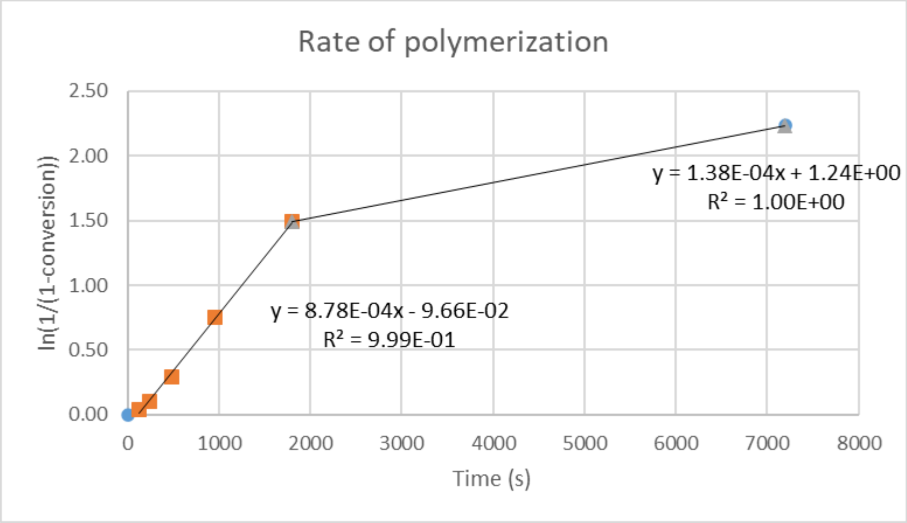
Rate of polymerization
2.50
2.00
1.50
1.00
0.50
0.00
ln(1/(1-conversion))
0
1000
2000
3000
4000
5000
6000
7000
8000
Time (s)
y = 1.38E-04x + 1.24E+00
R² = 1.00E+00
y = 8.78E-04x - 9.66E-02
R² = 9.99E-01

Methoxy butenolide (1a) and DVE in AcOBu – replicate 3

Poly(1a-co-DVE) in AcOBu					k1(obs)	8.73×10^{-4} [s^{-1}]
	1a		Dodecyl vinyl ether		k2(obs)	1.41×10^{-4} [s^{-1}]
Time [s]	Integral	Concentration [mol/L]	Integral	Concentration [mol/L]	Conversion	ln(1/[1-conversion])
0	0.90	2.00	0.96	2.00	0%	0.00
120	0.87	1.92	0.93	1.93	4%	0.04
240	0.78	1.72	0.86	1.79	12%	0.13
480	0.61	1.35	0.74	1.55	27%	0.32
960	0.33	0.74	0.55	1.13	53%	0.76
1800	0.08	0.17	0.35	0.72	78%	1.49
7200	0.01	0.02	0.19	0.40	90%	2.26

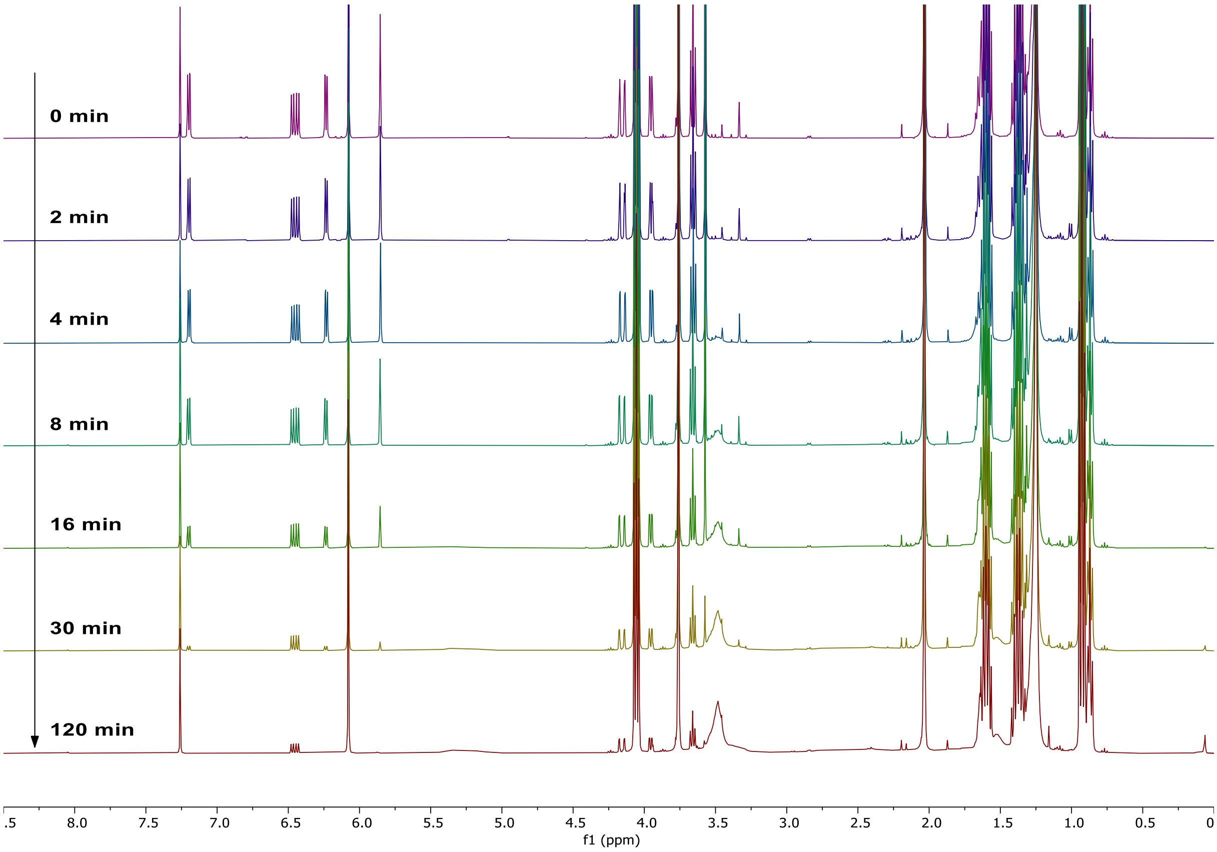

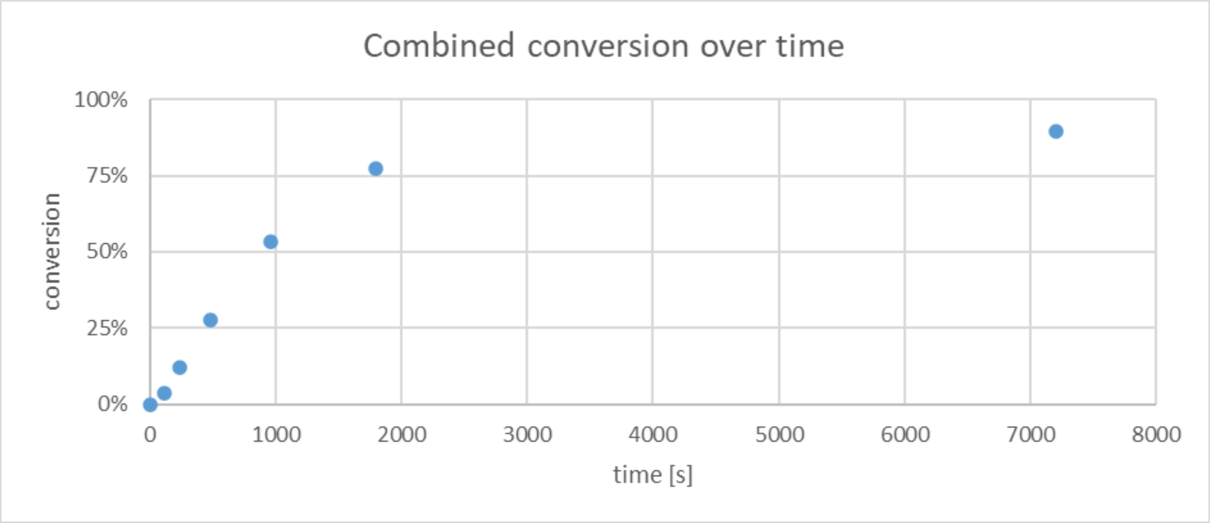

Combined conversion over time
100%
75%
50%
25%
0%
conversion
0
1000
2000
3000
4000
5000
6000
7000
8000
time [s]

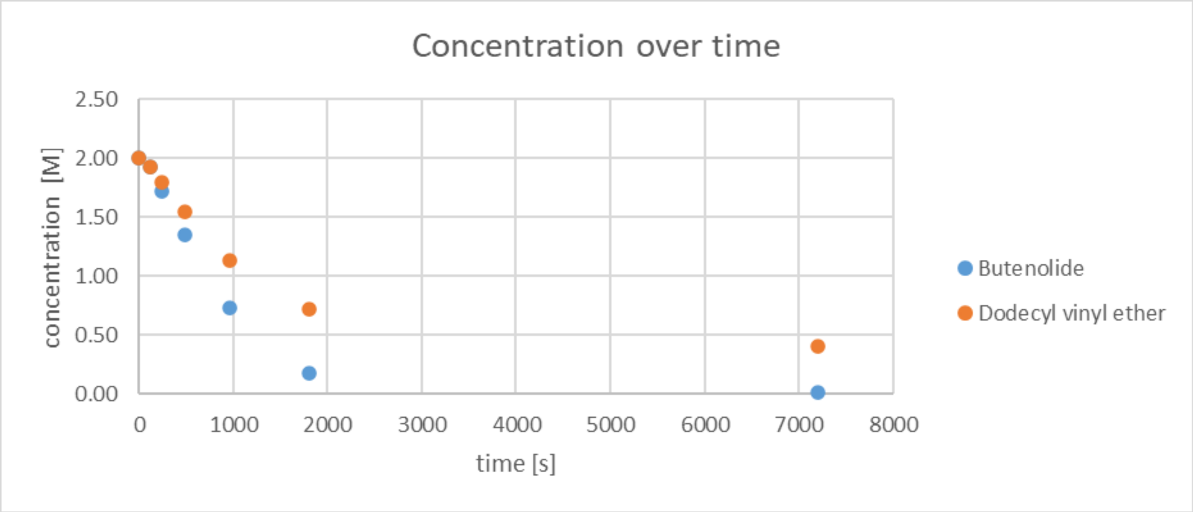

Concentration over time
2.50
2.00
1.50
1.00
0.50
0.00
concentration [M]
0
1000
2000
3000
4000
5000
6000
7000
8000
time [s]
Butenolide
Dodecyl vinyl ether

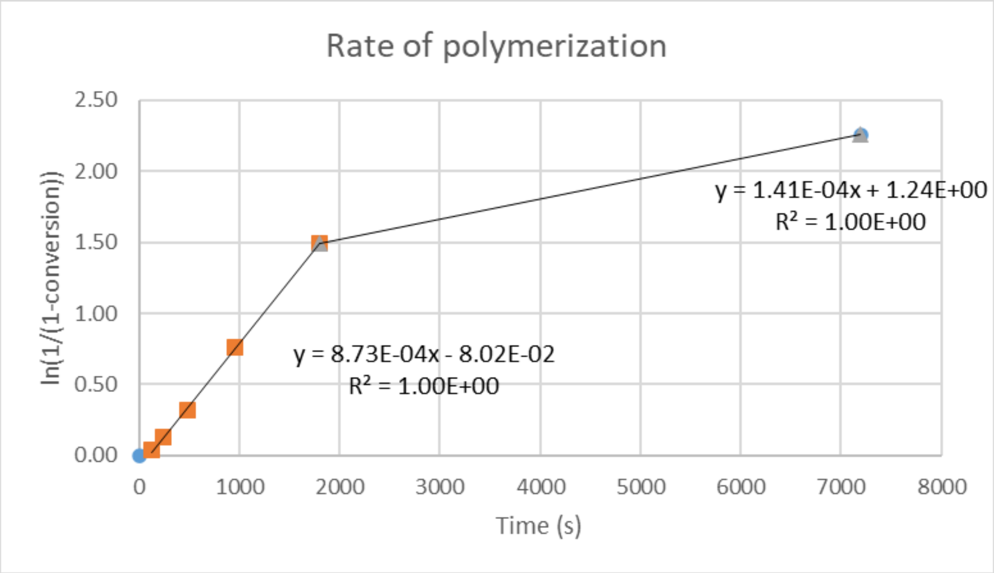

Rate of polymerization
2.50
2.00
1.50
1.00
0.50
0.00
ln(1/(1-conversion))
0
1000
2000
3000
4000
5000
6000
7000
8000
Time (s)
y = 1.41E-04x + 1.24E+00
R² = 1.00E+00
y = 8.73E-04x - 8.02E-02
R² = 1.00E+00

Methoxy butenolide (1a) and DVE in GVL

Poly(1a-co-DVE) in GVL					k1(obs)	1.14×10^{-3} [s^{-1}]
	1a		Dodecyl vinyl ether		k2(obs)	n/a
Time [s]	Integral	Concentration [mol/L]	Integral	Concentration [mol/L]	Conversion	ln(1/[1-conversion])
0	0.82	2.00	0.74	2.00	0%	0.00
300	0.70	1.71	0.64	1.73	14%	0.15
600	0.51	1.24	0.49	1.32	36%	0.44
1200	0.19	0.46	0.25	0.68	72%	1.26
1800	0.08	0.20	0.11	0.30	88%	2.09
2400	0.03	0.07	0.07	0.19	93%	2.72
3600	0.00	0.00	0.03	0.08	98%	3.90
7200	0.00	0.00	0.00	0.00	100%	n/a

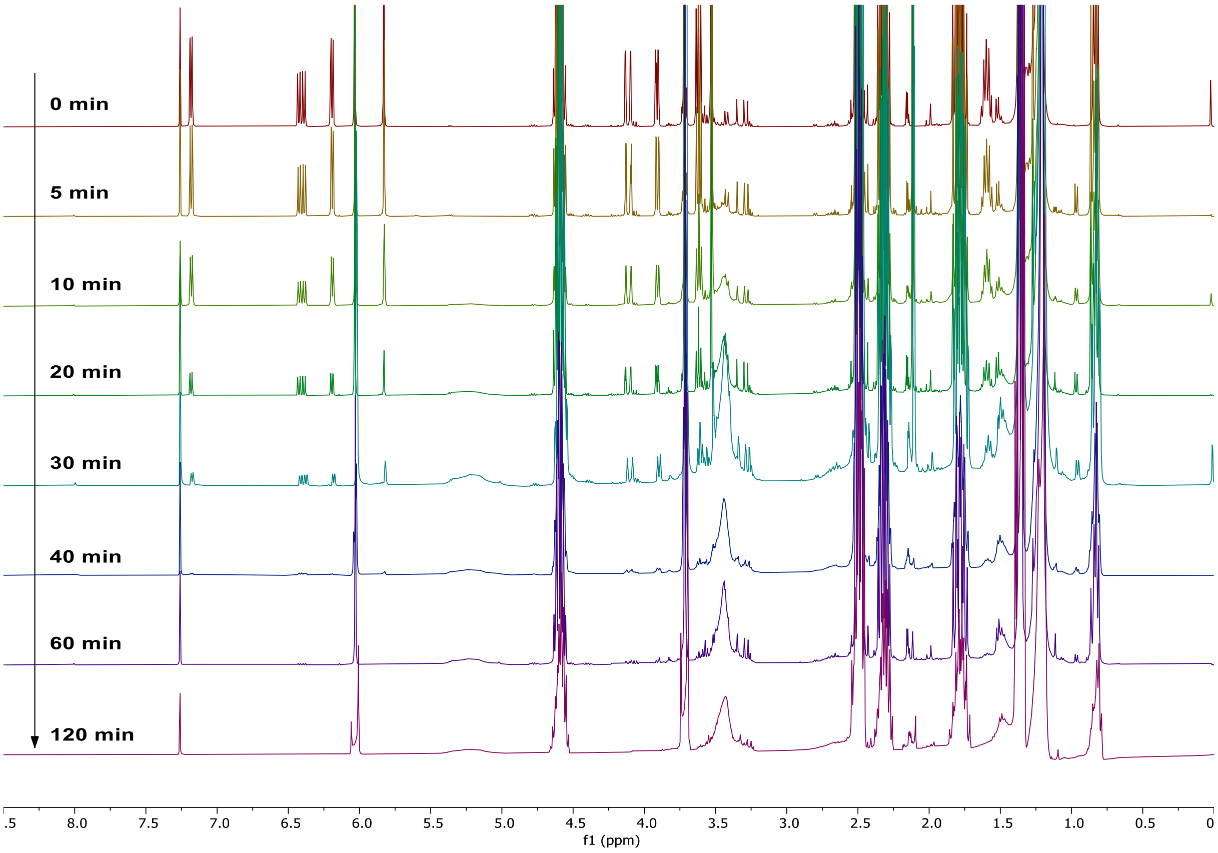

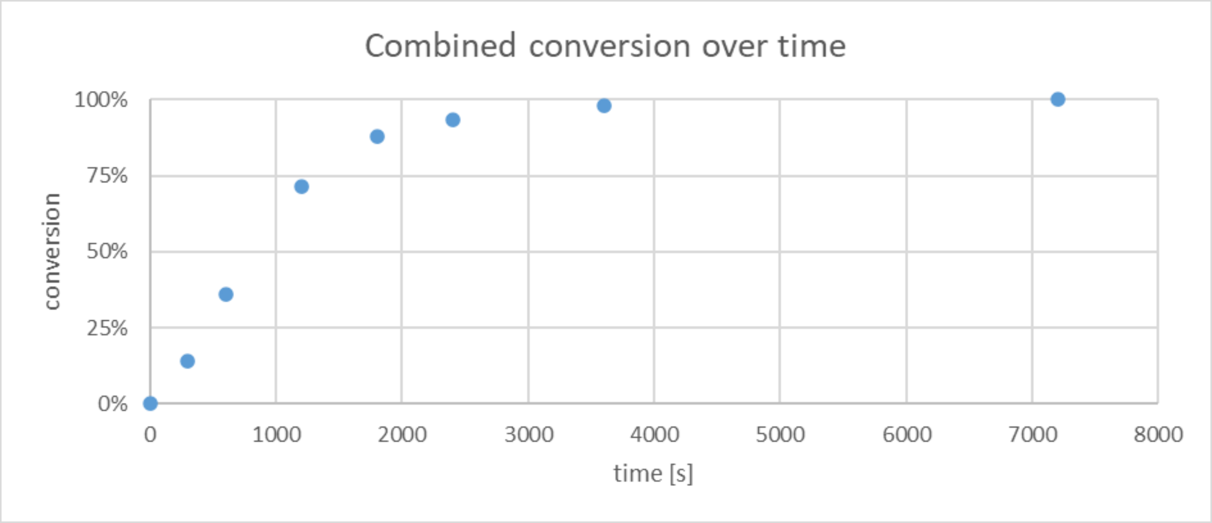
Combined conversion over time
conversion
100%
75%
50%
25%
0%
0
1000
2000
3000
4000
5000
6000
7000
8000
time [s]

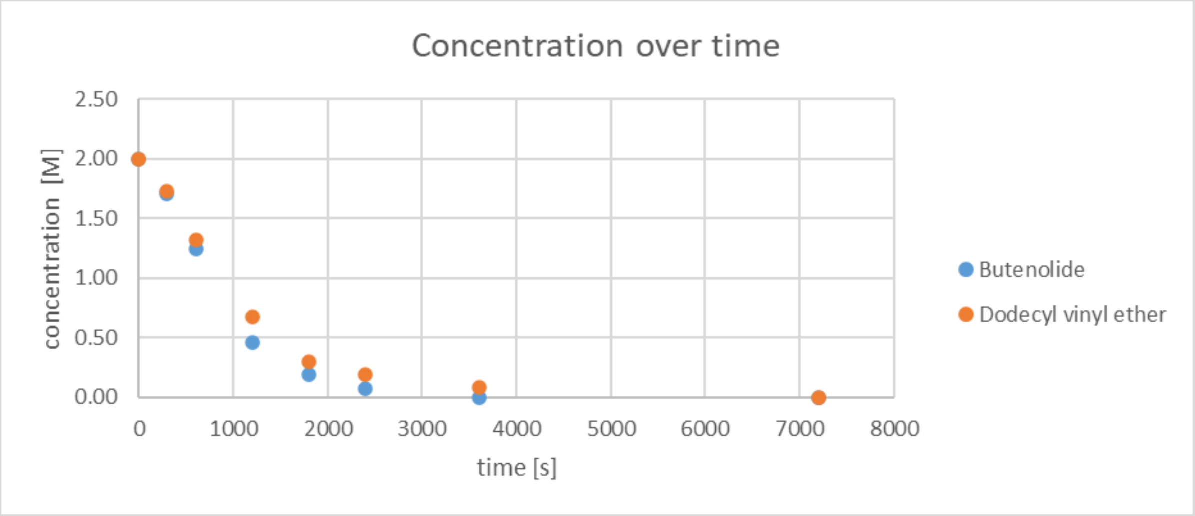
Concentration over time
concentration [M]
2.50
2.00
1.50
1.00
0.50
0.00
0
1000
2000
3000
4000
5000
6000
7000
8000
time [s]
Butenolide
Dodecyl vinyl ether

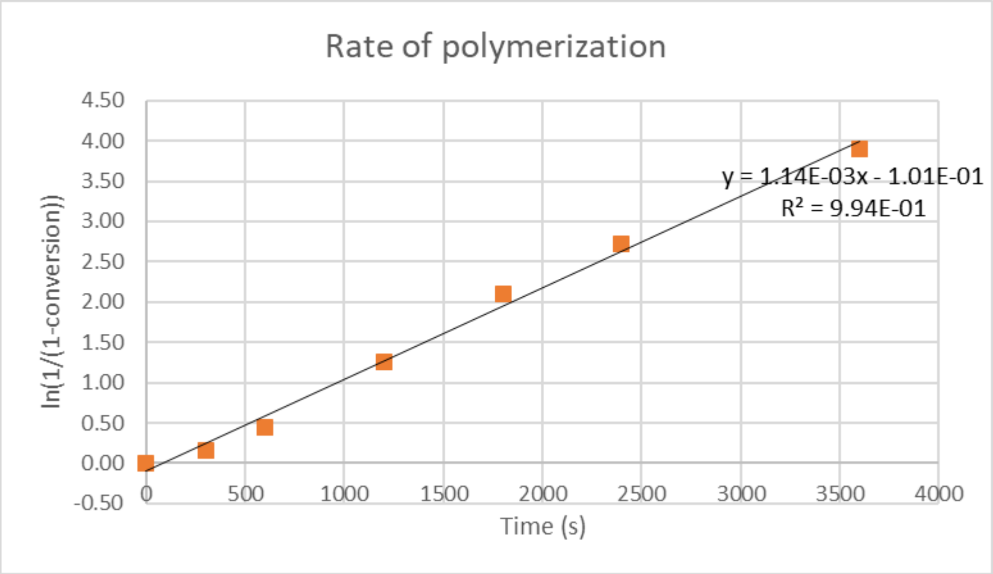
Rate of polymerization
ln(1/(1-conversion))
4.50
4.00
3.50
3.00
2.50
2.00
1.50
1.00
0.50
0.00
-0.50
0
500
1000
1500
2000
2500
3000
3500
4000
Time (s)
y = 1.14E-03x - 1.01E-01
R² = 9.94E-01

Methoxy butenolide (1a) and DVE in diethyl carbonate

Poly(1a-co-DVE) in diethyl carbonate					k1(obs)	7.00×10^{-4} [s^{-1}]
	1a		Dodecyl vinyl ether		k2(obs)	3.06×10^{-4} [s^{-1}]
Time [s]	Integral	Concentration [mol/L]	Integral	Concentration [mol/L]	Conversion	ln(1/[1-conversion])
0	0.82	2.15	0.86	2.15	0%	0.00
300	0.67	1.76	0.75	1.88	16%	0.17
600	0.48	1.26	0.61	1.53	35%	0.43
1200	0.25	0.66	0.45	1.13	59%	0.88
1800	0.12	0.31	0.35	0.88	72%	1.28
2400	0.04	0.10	0.29	0.73	81%	1.65
3600	0.00	0.00	0.23	0.58	87%	2.01

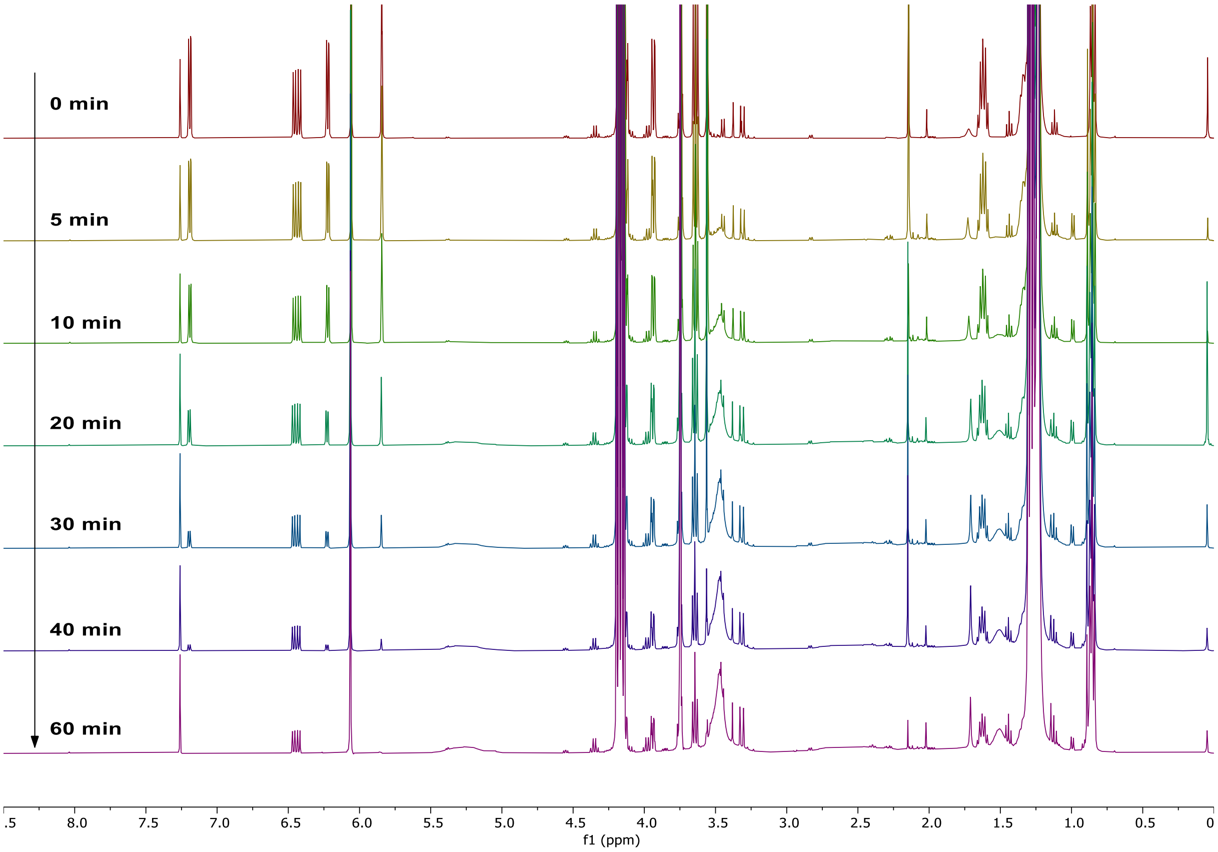

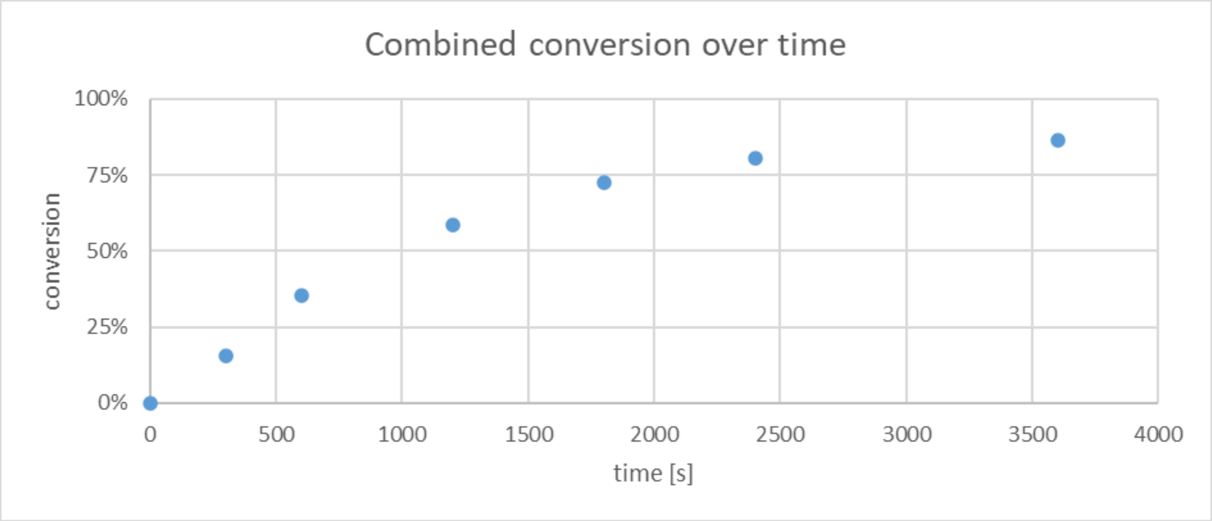
Combined conversion over time
100%
75%
50%
25%
0%
conversion
0
500
1000
1500
2000
2500
3000
3500
4000
time [s]

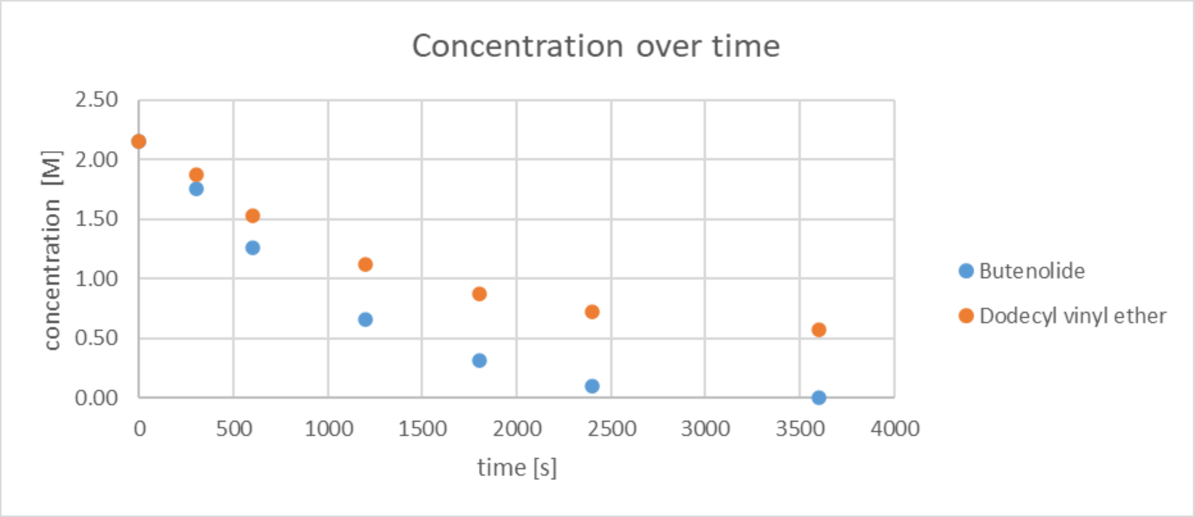
Concentration over time
2.50
2.00
1.50
1.00
0.50
0.00
concentration [M]
0
500
1000
1500
2000
2500
3000
3500
4000
time [s]
Butenolide
Dodecyl vinyl ether

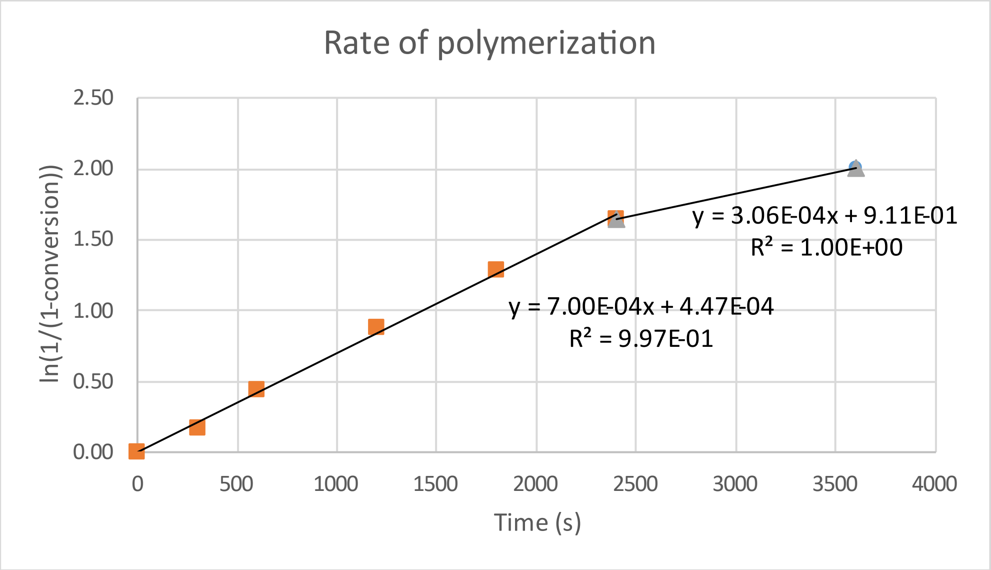
Rate of polymerization
2.50
2.00
1.50
1.00
0.50
0.00
ln(1/(1-conversion))
y = 3.06E-04x + 9.11E-01
R² = 1.00E+00
y = 7.00E-04x + 4.47E-04
R² = 9.97E-01
0
500
1000
1500
2000
2500
3000
3500
4000
Time (s)

Methoxy butenolide (1a) and DVE in 1M2P

See Hermens, Feringa *et al.*, *Sci. Adv.* **2020**, *6*, eabe0026.

Tert-butoxy butenolide (1b) and DVE in AcOBu

Poly(1b-co-DVE) in AcOBu					k0(obs)	1.08 . 10^{-4} [s^{-1}]
	1b		Dodecyl Vinyl Ether		k1(obs)	1.04 . 10^{-3} [s^{-1}]
Time [s]	Integral	Concentration [mol/L]	Integral	Concentration [mol/L]	Conversion	ln(1/[1-conversion])
0	0.88	2.00	0.98	2.00	0%	0.00
60	0.87	1.99	0.96	1.97	1%	0.01
120	0.87	1.98	0.95	1.95	2%	0.02
240	0.87	1.99	0.94	1.93	2%	0.02
480	0.81	1.84	0.86	1.75	10%	0.11
720	0.62	1.41	0.71	1.44	29%	0.34
960	0.45	1.03	0.58	1.19	45%	0.59
1440	0.23	0.51	0.40	0.82	67%	1.10
2160	0.09	0.19	0.28	0.57	81%	1.66
3120	0.02	0.05	0.20	0.41	89%	2.17
7200	0.00	0.00	0.08	0.16	96%	3.17

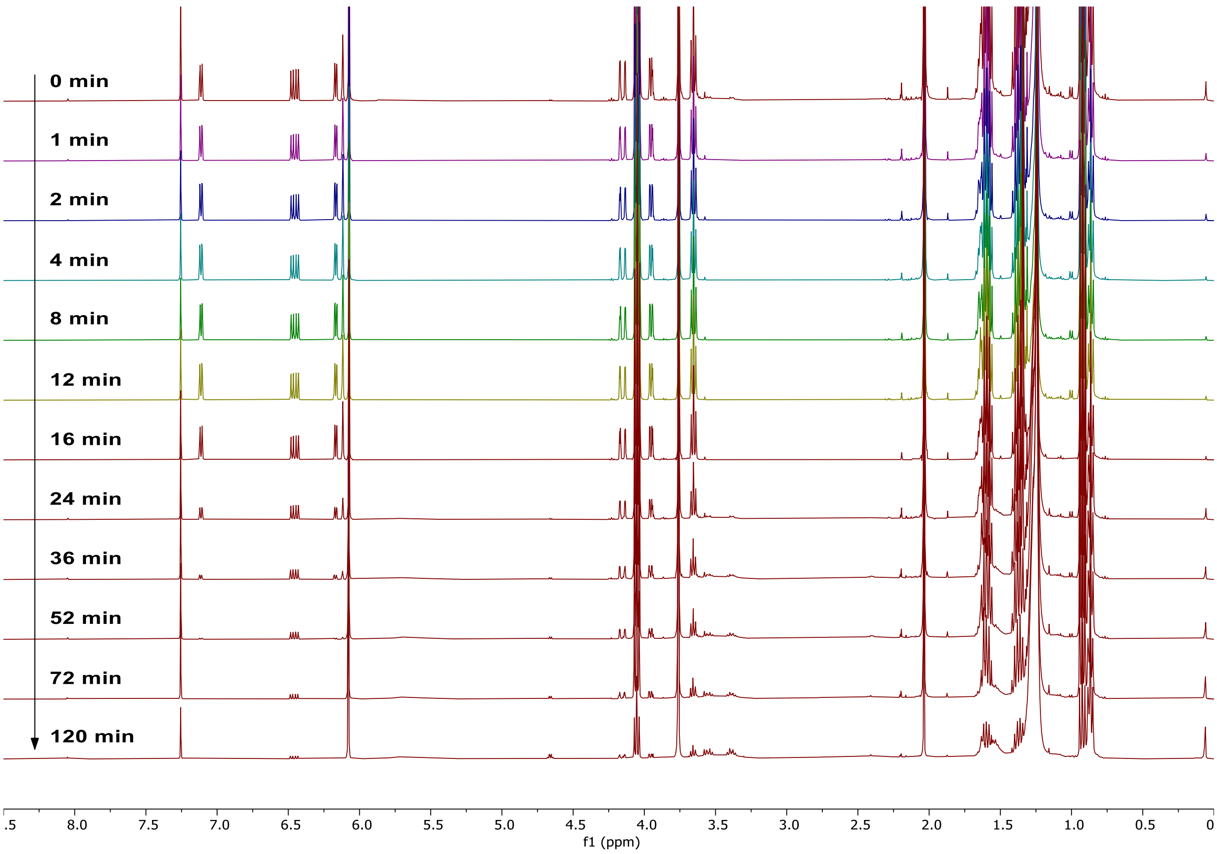

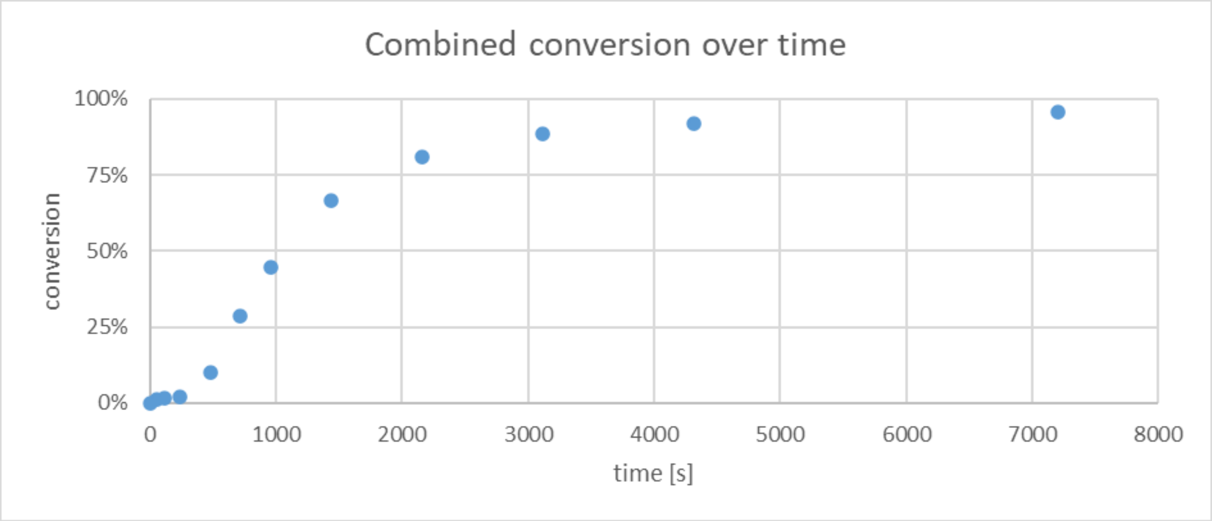

Combined conversion over time
100%
75%
50%
25%
0%
conversion
0
1000
2000
3000
4000
5000
6000
7000
8000
time [s]

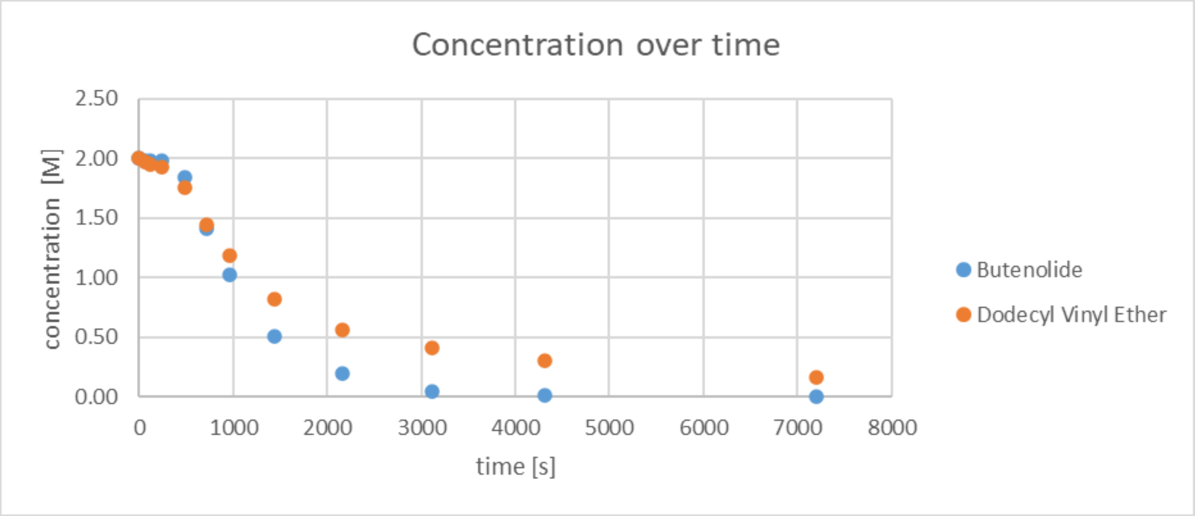

Concentration over time
2.50
2.00
1.50
1.00
0.50
0.00
concentration [M]
0
1000
2000
3000
4000
5000
6000
7000
8000
time [s]
Butenolide
Dodecyl Vinyl Ether

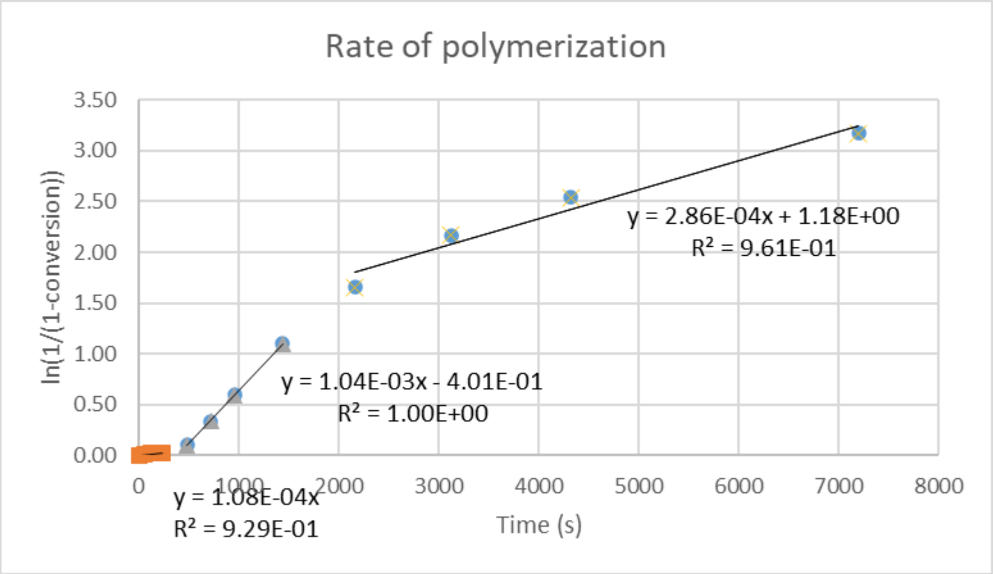

Rate of polymerization
3.50
3.00
2.50
2.00
1.50
1.00
0.50
0.00
ln(1/(1-conversion))
y = 2.86E-04x + 1.18E+00
R² = 9.61E-01
y = 1.04E-03x - 4.01E-01
R² = 1.00E+00
y = 1.08E-04x
R² = 9.29E-01
0
1000
2000
3000
4000
5000
6000
7000
8000
Time (s)

Acetoxy butenolide (2a) and DVE in AcOBu – replicate 1

Poly(2a-co-DVE) in AcOBu					k1(obs)	2.25×10^{-3} [s^{-1}]
	2a		Dodecyl vinyl ether		k2(obs)	3.78×10^{-4} [s^{-1}]
Time [s]	Integral	Concentration [mol/L]	Integral	Concentration [mol/L]	Conversion	ln(1/[1-conversion])
0	0.95	2.00	0.96	2.00	0%	0.00
60	0.85	1.80	0.87	1.82	10%	0.10
120	0.73	1.55	0.75	1.57	22%	0.25
240	0.52	1.09	0.59	1.23	42%	0.55
480	0.28	0.58	0.38	0.80	65%	1.06
900	0.05	0.12	0.23	0.47	85%	1.92
1800	0.00	0.01	0.18	0.37	91%	2.36
2700	0.00	0.01	0.11	0.22	94%	2.86
3600	0.00	0.01	0.10	0.22	94%	2.88
5400	0.00	0.01	0.03	0.06	98%	4.10
7200	0.00	0.01	0.03	0.06	98%	4.04

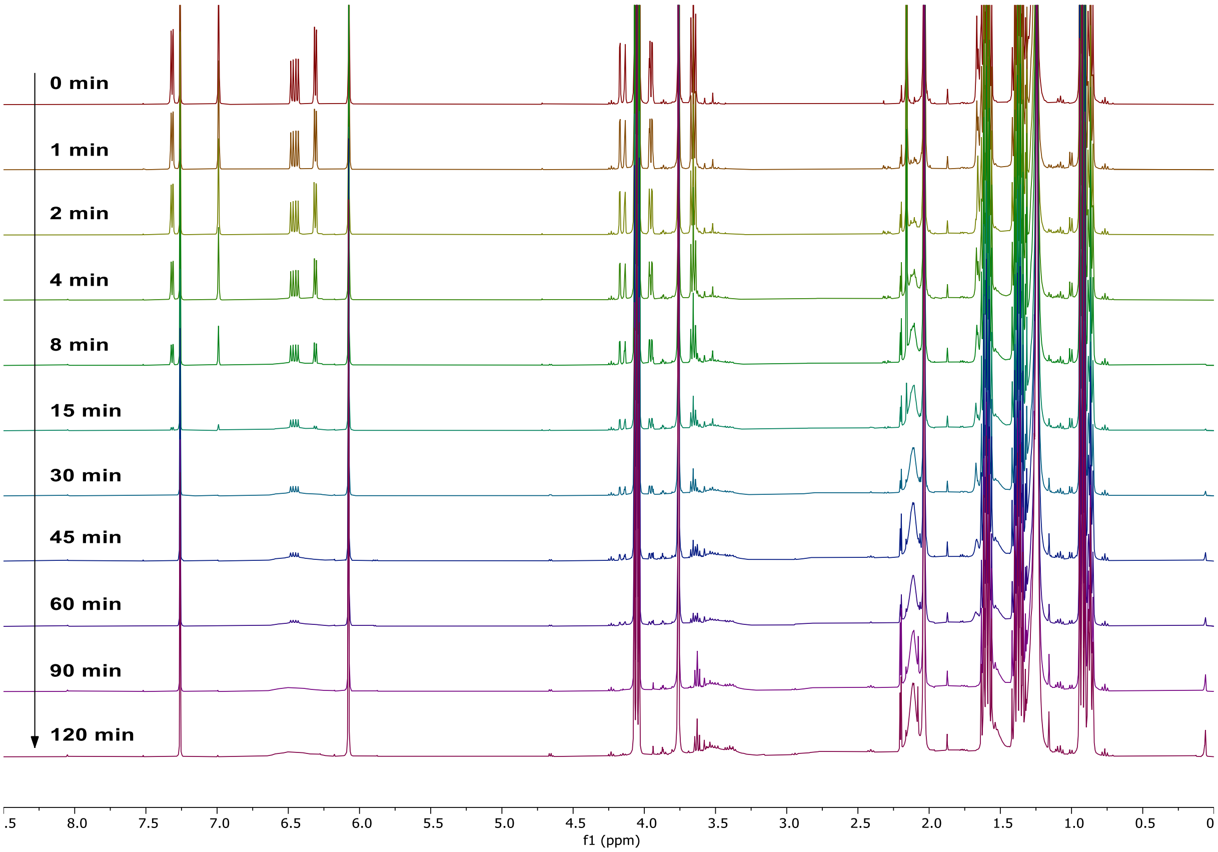

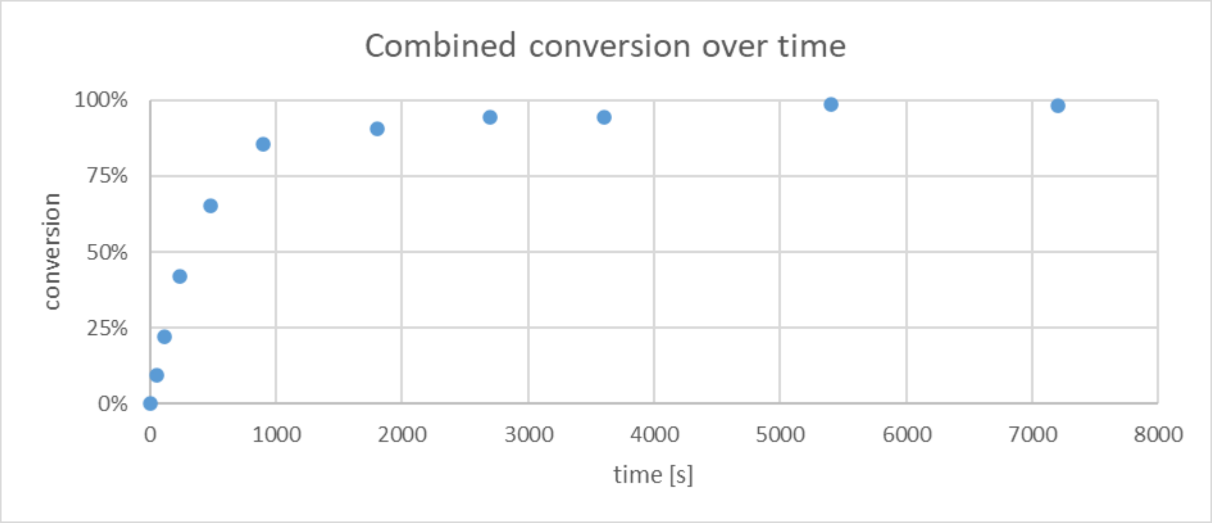
Combined conversion over time
conversion
100%
75%
50%
25%
0%
0
1000
2000
3000
4000
5000
6000
7000
8000
time [s]

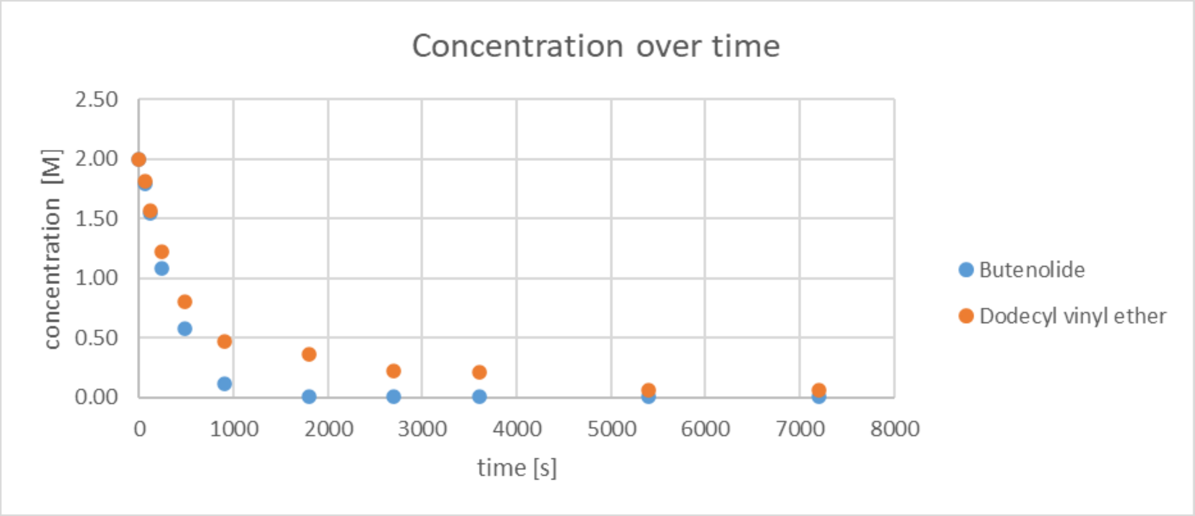
Concentration over time
concentration [M]
2.50
2.00
1.50
1.00
0.50
0.00
0
1000
2000
3000
4000
5000
6000
7000
8000
time [s]
Butenolide
Dodecyl vinyl ether

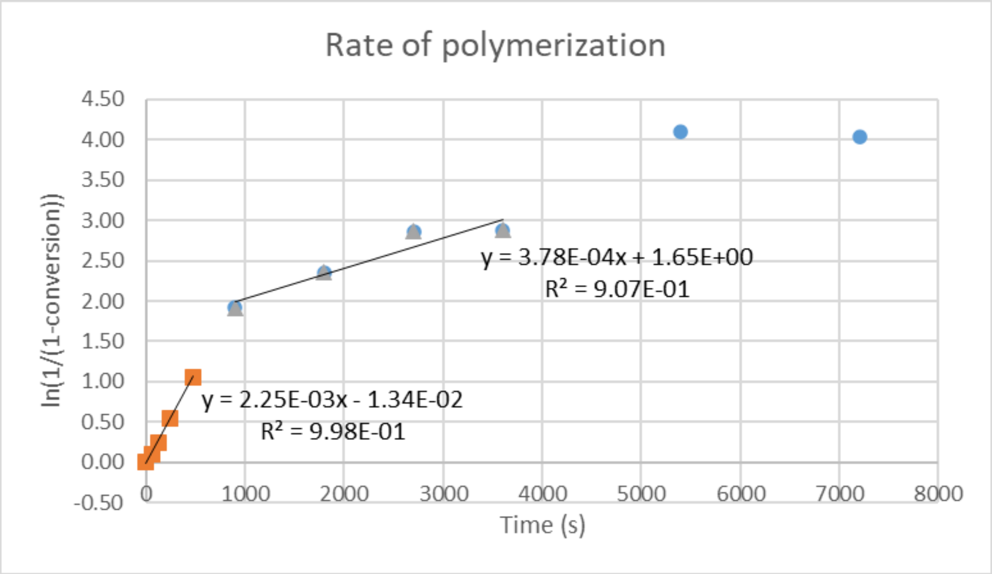
Rate of polymerization
ln(1/(1-conversion))
4.50
4.00
3.50
3.00
2.50
2.00
1.50
1.00
0.50
0.00
-0.50
0
1000
2000
3000
4000
5000
6000
7000
8000
Time (s)
y = 3.78E-04x + 1.65E+00
R² = 9.07E-01
y = 2.25E-03x - 1.34E-02
R² = 9.98E-01

Acetoxy butenolide (2a) and DVE in AcOBu – replicate 2

Poly(2a-co-DVE) in AcOBu					k1(obs)	2.10×10^{-3} [s^{-1}]
	2a		Dodecyl vinyl ether		k2(obs)	1.56×10^{-4} [s^{-1}]
Time [s]	Integral	Concentration [mol/L]	Integral	Concentration [mol/L]	Conversion	ln(1/[1-conversion])
0	0.92	2.00	0.96	2.00	0%	0.00
120	0.67	1.47	0.78	1.63	22%	0.25
240	0.48	1.05	0.64	1.34	40%	0.52
480	0.23	0.51	0.46	0.95	64%	1.01
960	0.04	0.10	0.30	0.63	82%	1.70
1800	0.00	0.01	0.24	0.51	87%	2.05
7200	0.00	0.00	0.12	0.25	94%	2.77

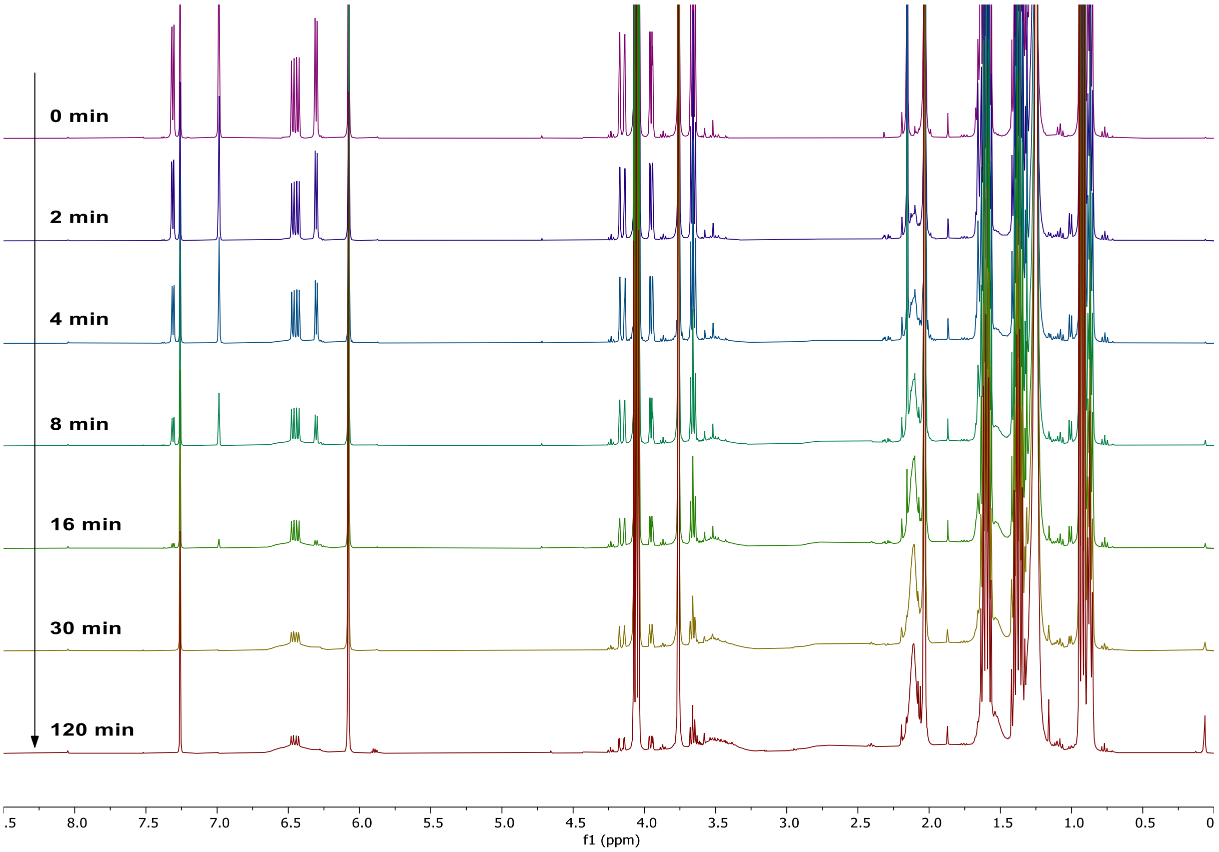

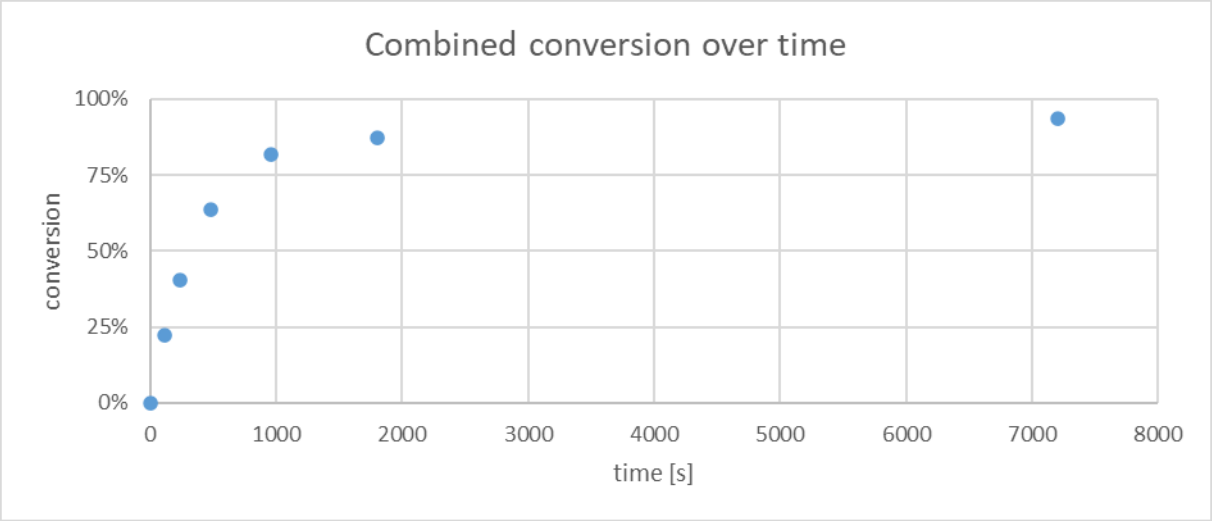
Combined conversion over time
conversion
100%
75%
50%
25%
0%
0
1000
2000
3000
4000
5000
6000
7000
8000
time [s]

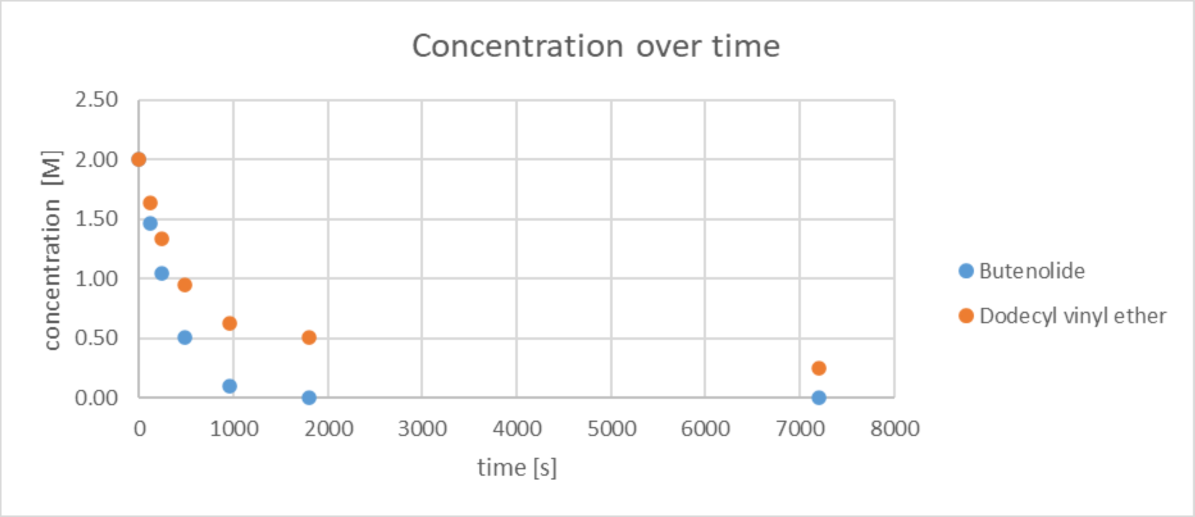
Concentration over time
concentration [M]
2.50
2.00
1.50
1.00
0.50
0.00
0
1000
2000
3000
4000
5000
6000
7000
8000
time [s]
Butenolide
Dodecyl vinyl ether

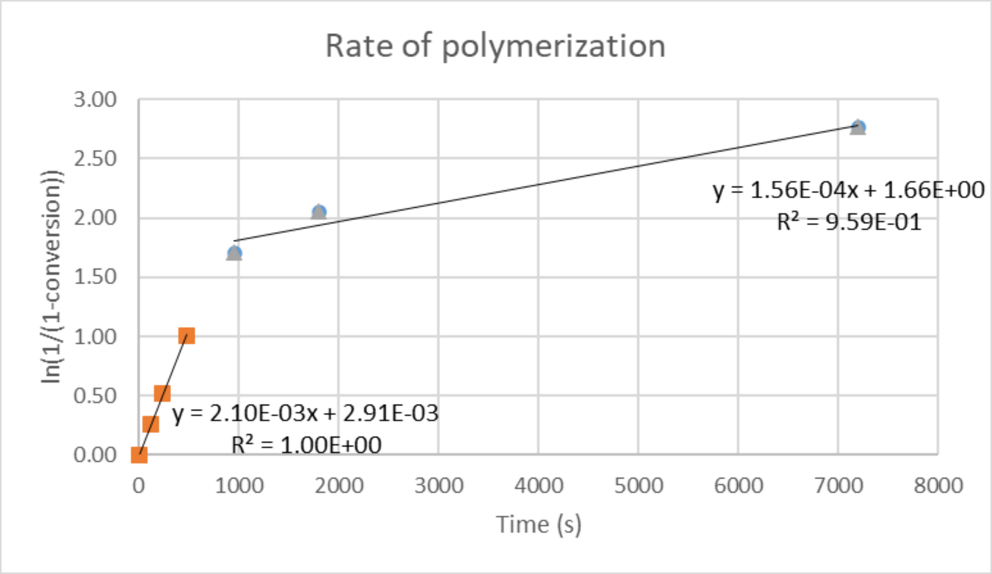
Rate of polymerization
ln(1/(1-conversion))
3.00
2.50
2.00
1.50
1.00
0.50
0.00
0
1000
2000
3000
4000
5000
6000
7000
8000
Time (s)
y = 1.56E-04x + 1.66E+00
R² = 9.59E-01
y = 2.10E-03x + 2.91E-03
R² = 1.00E+00

Acetoxy butenolide (2a) and DVE in AcOBu – replicate 3

Poly(2a-co-DVE) in AcOBu					k1(obs)	2.03×10^{-3} [s^{-1}]
	2a		Dodecyl vinyl ether		k2(obs)	1.43×10^{-4} [s^{-1}]
Time [s]	Integral	Concentration [mol/L]	Integral	Concentration [mol/L]	Conversion	ln(1/[1-conversion])
0	0.95	2.00	0.96	2.00	0%	0.00
120	0.78	1.65	0.84	1.75	15%	0.16
240	0.58	1.23	0.69	1.43	33%	0.41
480	0.27	0.57	0.47	0.97	62%	0.96
960	0.05	0.11	0.28	0.58	83%	1.75
1800	0.00	0.01	0.23	0.48	88%	2.10
7200	0.00	0.00	0.12	0.26	93%	2.73

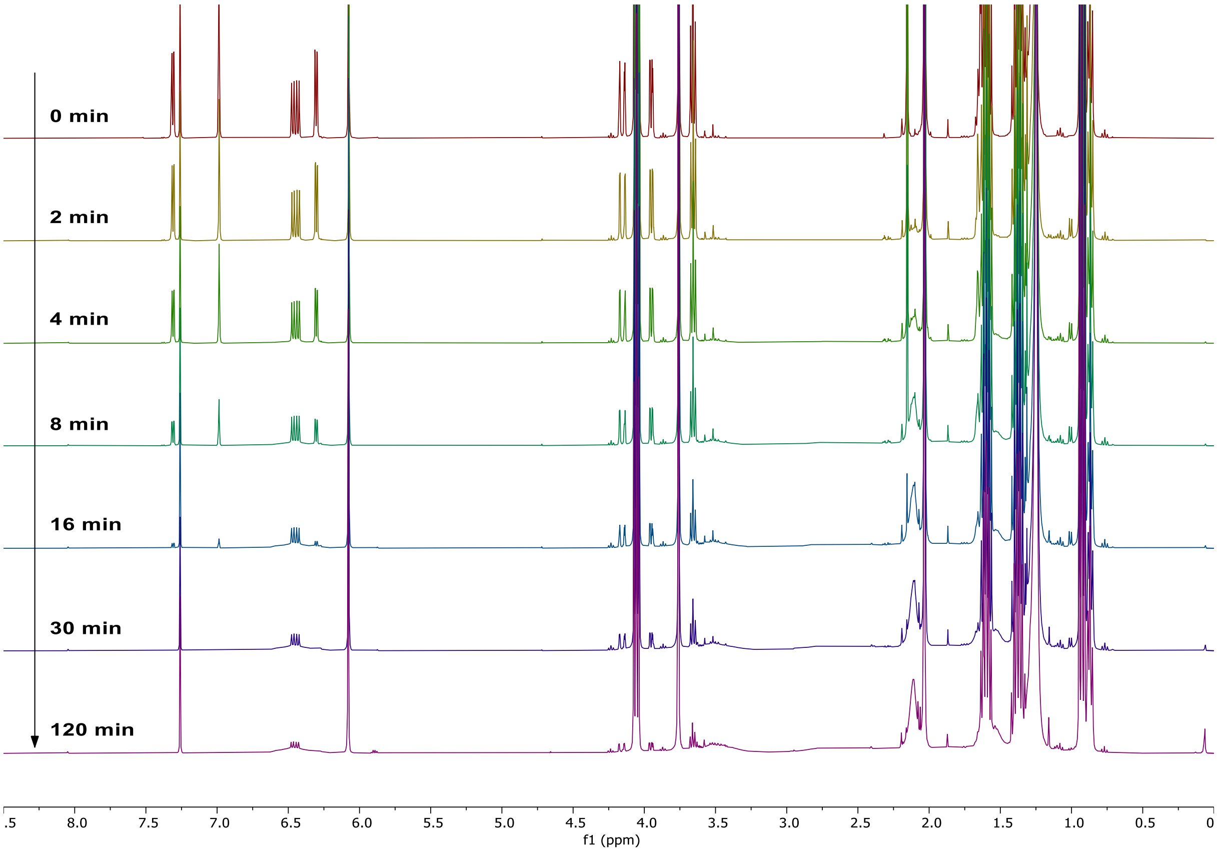

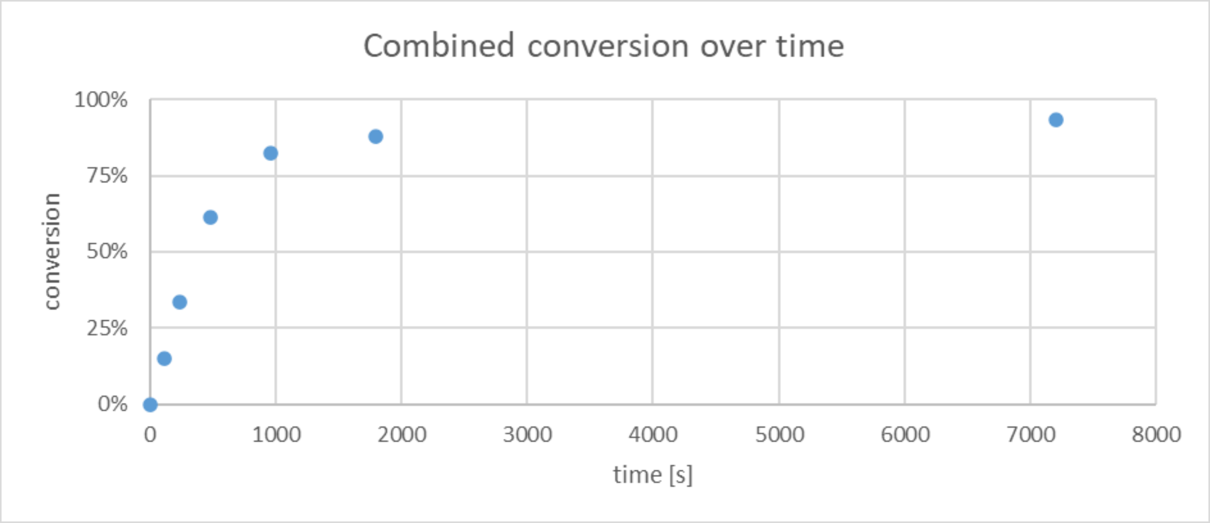
Combined conversion over time
100%
75%
50%
25%
0%
conversion
0
1000
2000
3000
4000
5000
6000
7000
8000
time [s]

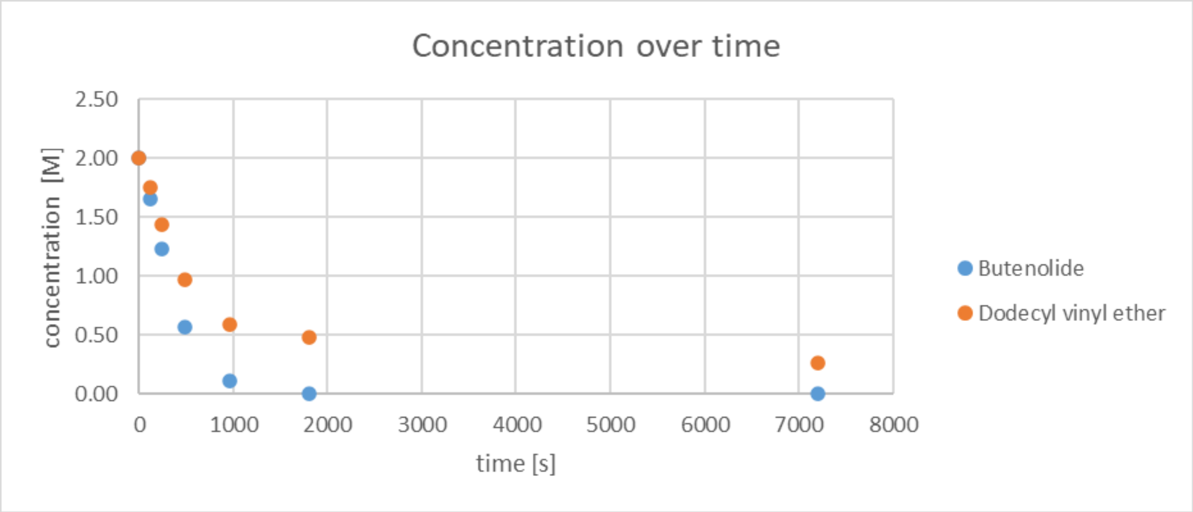
Concentration over time
2.50
2.00
1.50
1.00
0.50
0.00
concentration [M]
0
1000
2000
3000
4000
5000
6000
7000
8000
time [s]
Butenolide
Dodecyl vinyl ether

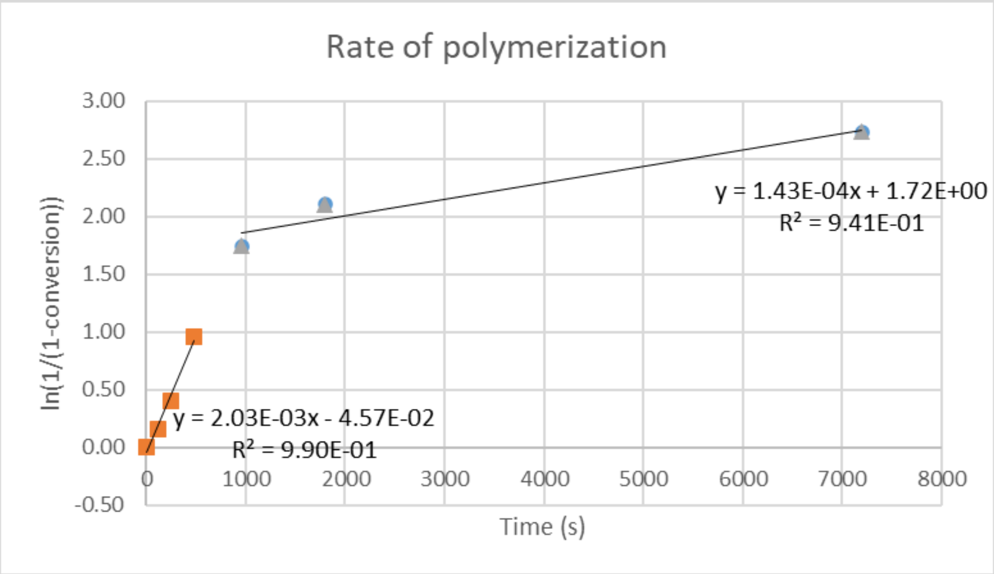
Rate of polymerization
3.00
2.50
2.00
1.50
1.00
0.50
0.00
-0.50
ln(1/(1-conversion))
y = 1.43E-04x + 1.72E+00
R² = 9.41E-01
y = 2.03E-03x - 4.57E-02
R² = 9.90E-01
0
1000
2000
3000
4000
5000
6000
7000
8000
Time (s)

Acetoxy butenolide (2a) and DVE in 1-methoxy-2-propanol

Poly(2a-co-DVE)					k1(obs)	$3.39 \cdot 10^{-3}$ [s^{-1}]
	2a		Dodecyl vinyl ether		k2(obs)	$1.26 \cdot 10^{-4}$ [s^{-1}]
Time [s]	Integral	Concentration [mol/L]	Integral	Concentration [mol/L]	Conversion	ln(1/[1-conversion])
0	0.96	2.00	1.08	2.00	0%	0.00
60	0.74	1.55	0.92	1.71	19%	0.21
120	0.51	1.06	0.75	1.39	39%	0.49
240	0.33	0.69	0.63	1.16	54%	0.77
480	0.14	0.30	0.47	0.88	71%	1.22
720	0.06	0.13	0.41	0.75	78%	1.51
1080	0.02	0.04	0.35	0.65	83%	1.75
1440	0.01	0.01	0.33	0.61	84%	1.86
2160	0.00	0.00	0.29	0.54	87%	2.01
3120	0.00	0.00	0.26	0.49	88%	2.10
7200	0.00	0.00	0.16	0.29	93%	2.61

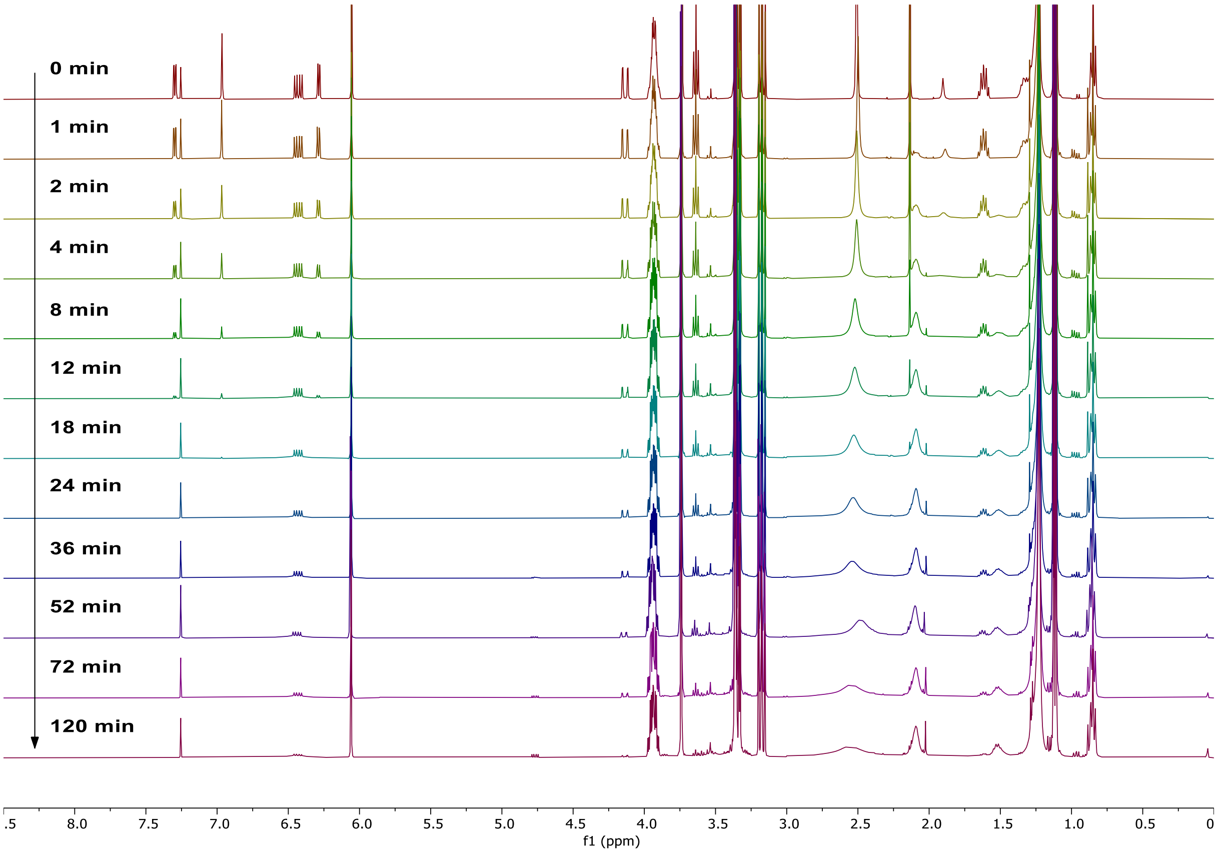

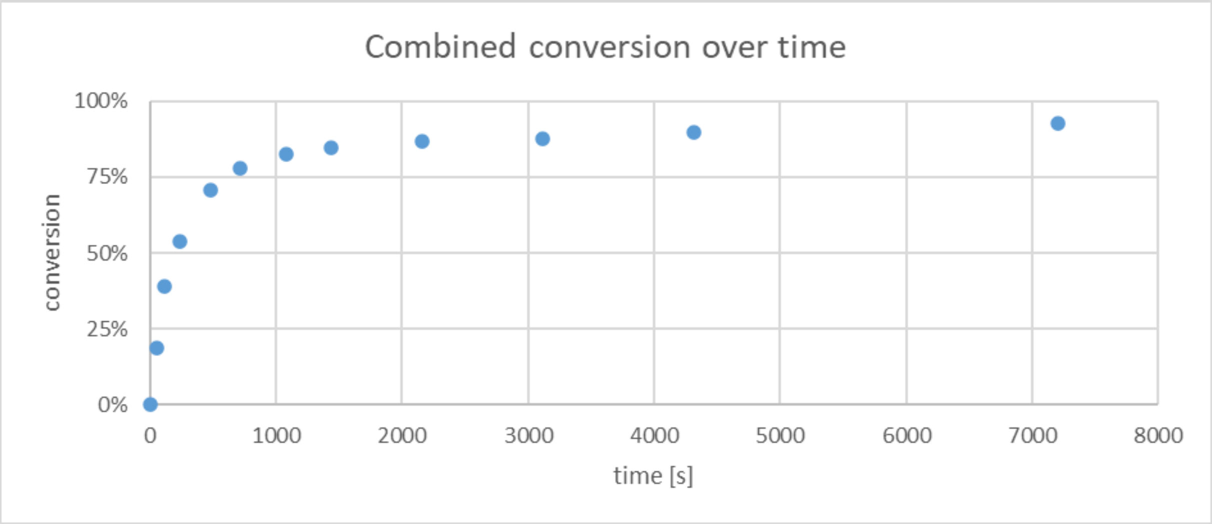

Combined conversion over time
conversion
100%
75%
50%
25%
0%
0
1000
2000
3000
4000
5000
6000
7000
8000
time [s]

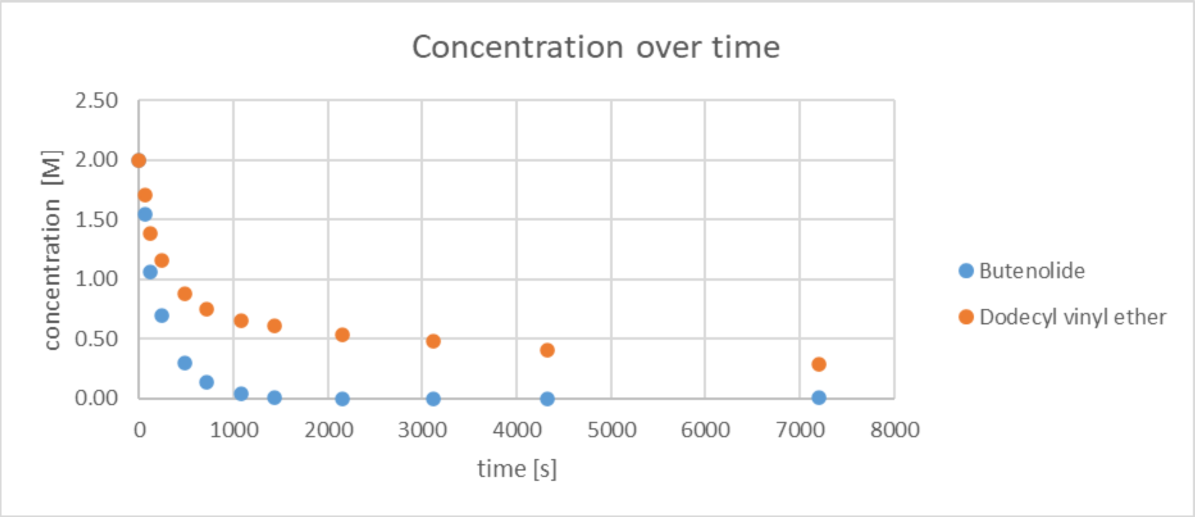

Concentration over time
concentration [M]
2.50
2.00
1.50
1.00
0.50
0.00
0
1000
2000
3000
4000
5000
6000
7000
8000
time [s]
Butenolide
Dodecyl vinyl ether

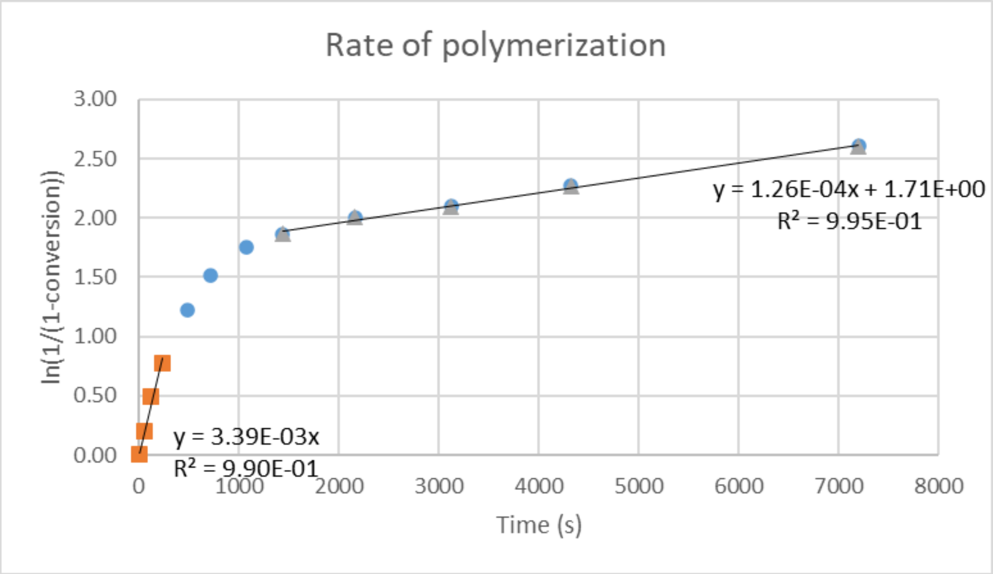

Rate of polymerization
ln(1/(1-conversion))
3.00
2.50
2.00
1.50
1.00
0.50
0.00
y = 1.26E-04x + 1.71E+00
R² = 9.95E-01
y = 3.39E-03x
R² = 9.90E-01
0
1000
2000
3000
4000
5000
6000
7000
8000
Time (s)

Isobutyroxy butenolide (2b) and DVE in AcOBu

Poly(2b-co-DVE) in AcOBu					k1(obs)	2.55 x 10^{-3} [s^{-1}]
	2b		Dodecyl vinyl ether		k2(obs)	n/a
Time [s]	Integral	Concentration [mol/L]	Integral	Concentration [mol/L]	Conversion	ln(1/[1-conversion])
0	0.93	2.00	0.92	2.00	0%	0.00
60	0.92	1.98	0.80	1.73	7%	0.07
120	0.93	1.99	0.73	1.58	11%	0.11
240	0.78	1.68	0.63	1.38	23%	0.27
480	0.42	0.89	0.32	0.69	60%	0.92
720	0.22	0.48	0.17	0.36	79%	1.56
1080	0.10	0.22	0.02	0.04	94%	2.74
1680	0.02	0.03	-0.02	-0.05	100%	n/a
2280	0.00	0.00	-0.06	-0.13	103%	n/a
3120	0.00	0.01	0.02	0.04	99%	4.43
4320	0.01	0.01	0.02	0.04	99%	4.26

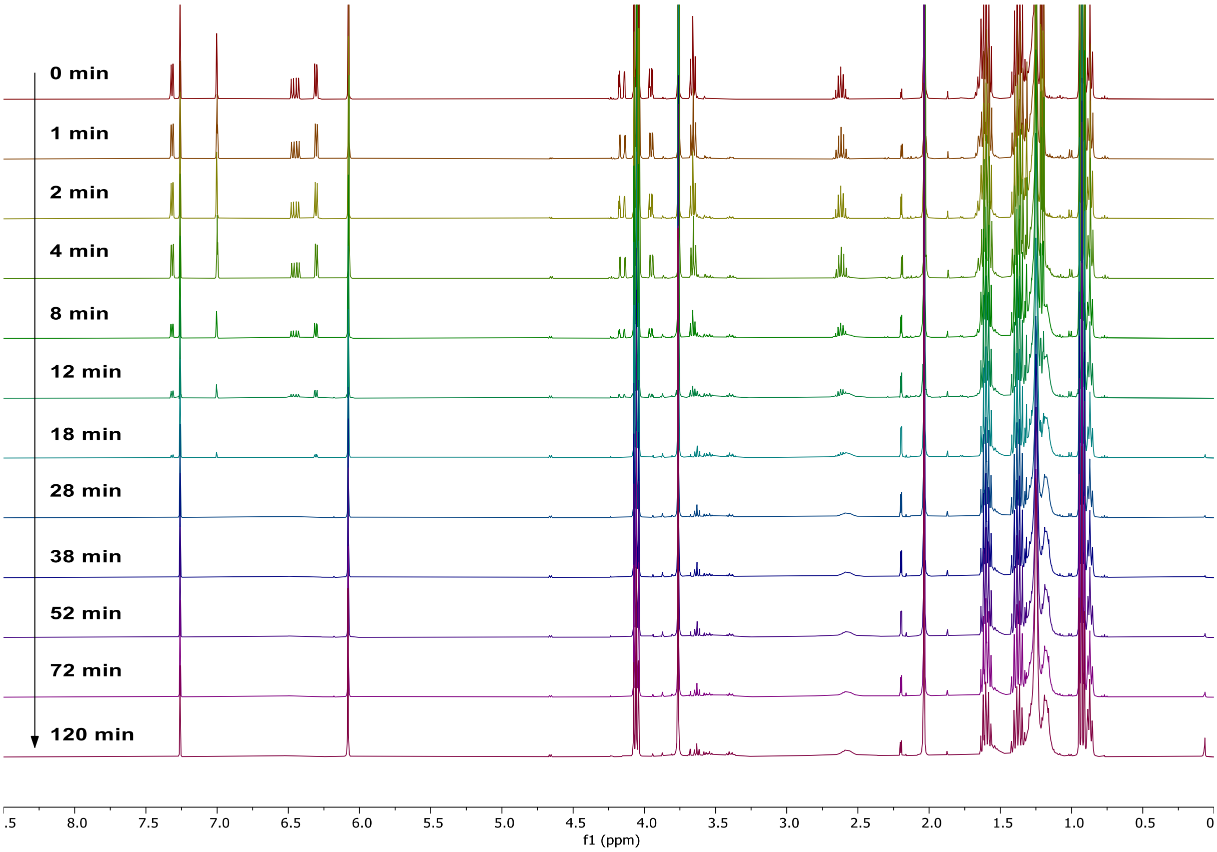

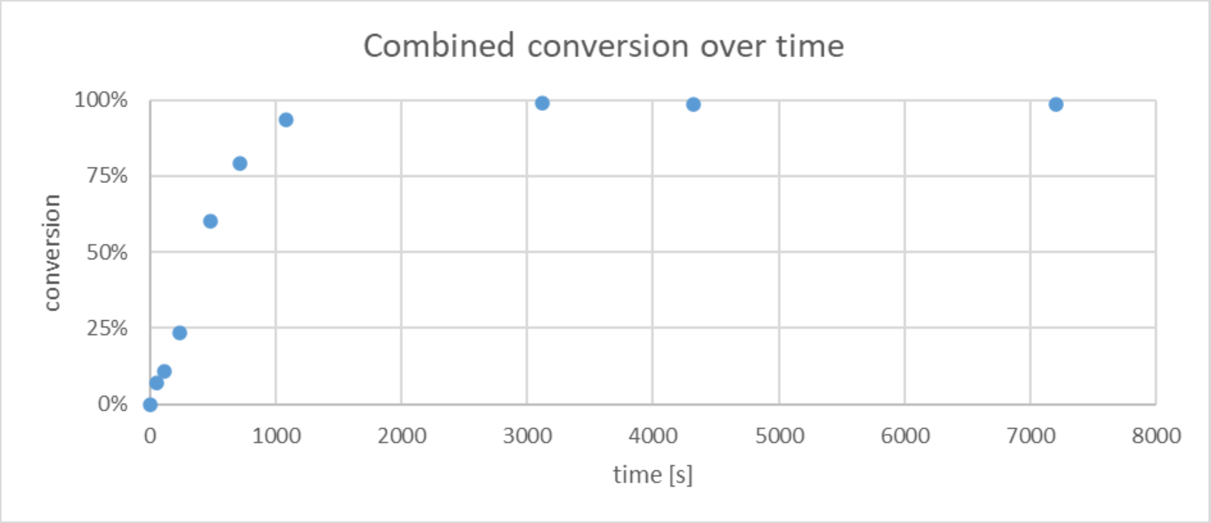
Combined conversion over time
100%
75%
50%
25%
0%
conversion
0
1000
2000
3000
4000
5000
6000
7000
8000
time [s]

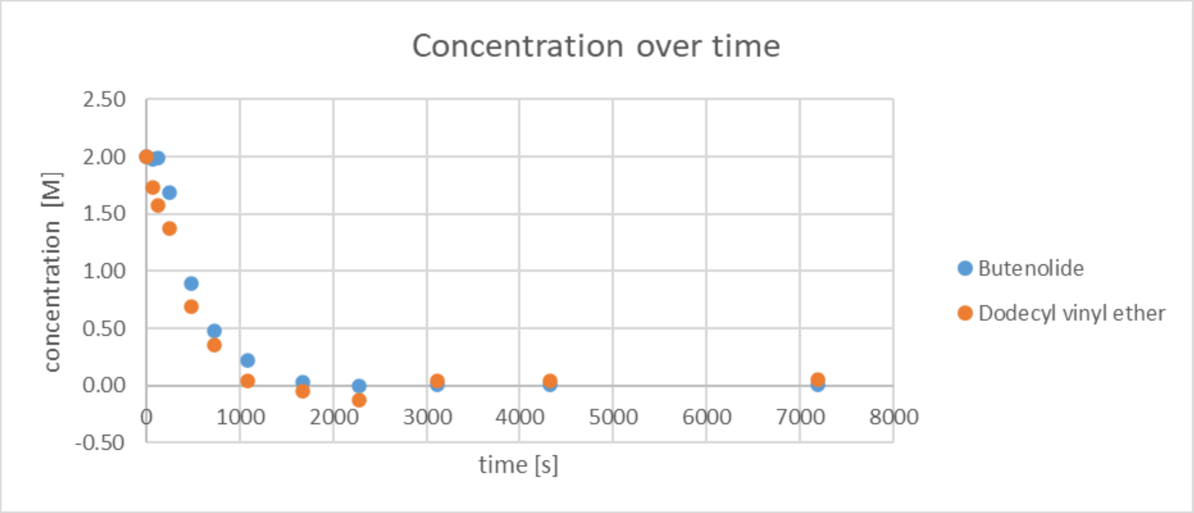
Concentration over time
2.50
2.00
1.50
1.00
0.50
0.00
-0.50
concentration [M]
0
1000
2000
3000
4000
5000
6000
7000
8000
time [s]
Butenolide
Dodecyl vinyl ether

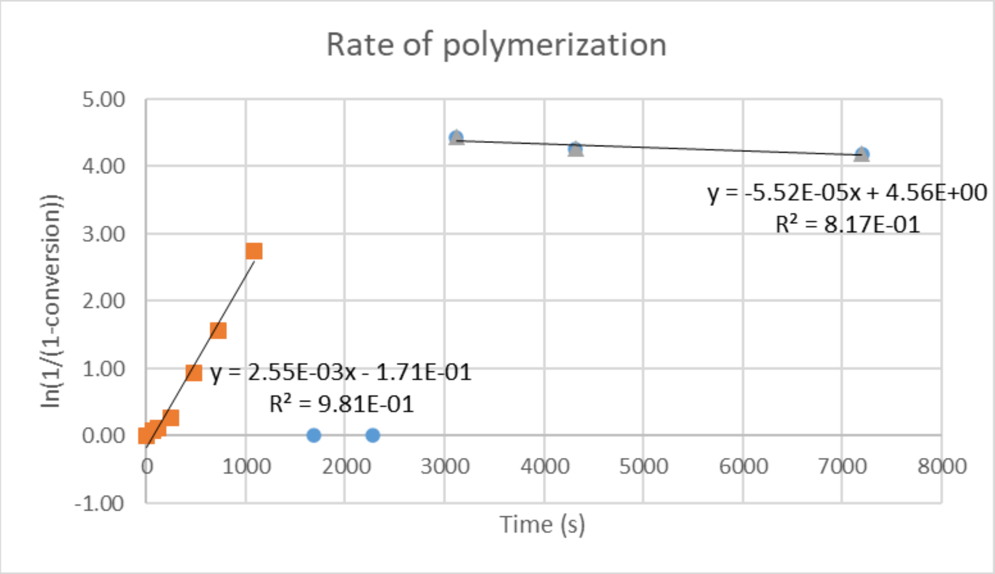
Rate of polymerization
5.00
4.00
3.00
2.00
1.00
0.00
-1.00
ln(1/(1-conversion))
y = -5.52E-05x + 4.56E+00
R² = 8.17E-01
y = 2.55E-03x - 1.71E-01
R² = 9.81E-01
0
1000
2000
3000
4000
5000
6000
7000
8000
Time (s)

Pivaloyloxy butenolide (2c) and DVE in AcOBu

Poly(2c-co-DVE) in AcOBu					k1(obs)	2.59×10^{-3} [s^{-1}]
	2c		Dodecyl vinyl ether		k2(obs)	1.44×10^{-4} [s^{-1}]
Time [s]	Integral	Concentration [mol/L]	Integral	Concentration [mol/L]	Conversion	ln(1/[1-conversion])
0	0.96	2.00	0.93	2.00	0%	0.00
60	0.89	1.86	0.86	1.84	8%	0.08
120	0.75	1.56	0.75	1.60	21%	0.23
240	0.42	0.87	0.47	1.02	53%	0.75
480	0.21	0.44	0.31	0.66	73%	1.29
720	0.09	0.19	0.20	0.42	85%	1.88
960	0.03	0.07	0.13	0.28	91%	2.42
1440	0.01	0.02	0.03	0.07	98%	3.79
2160	0.00	0.01	0.02	0.04	99%	4.45
3240	0.00	0.01	0.02	0.05	98%	4.19
7200	0.00	0.01	0.01	0.03	99%	4.73

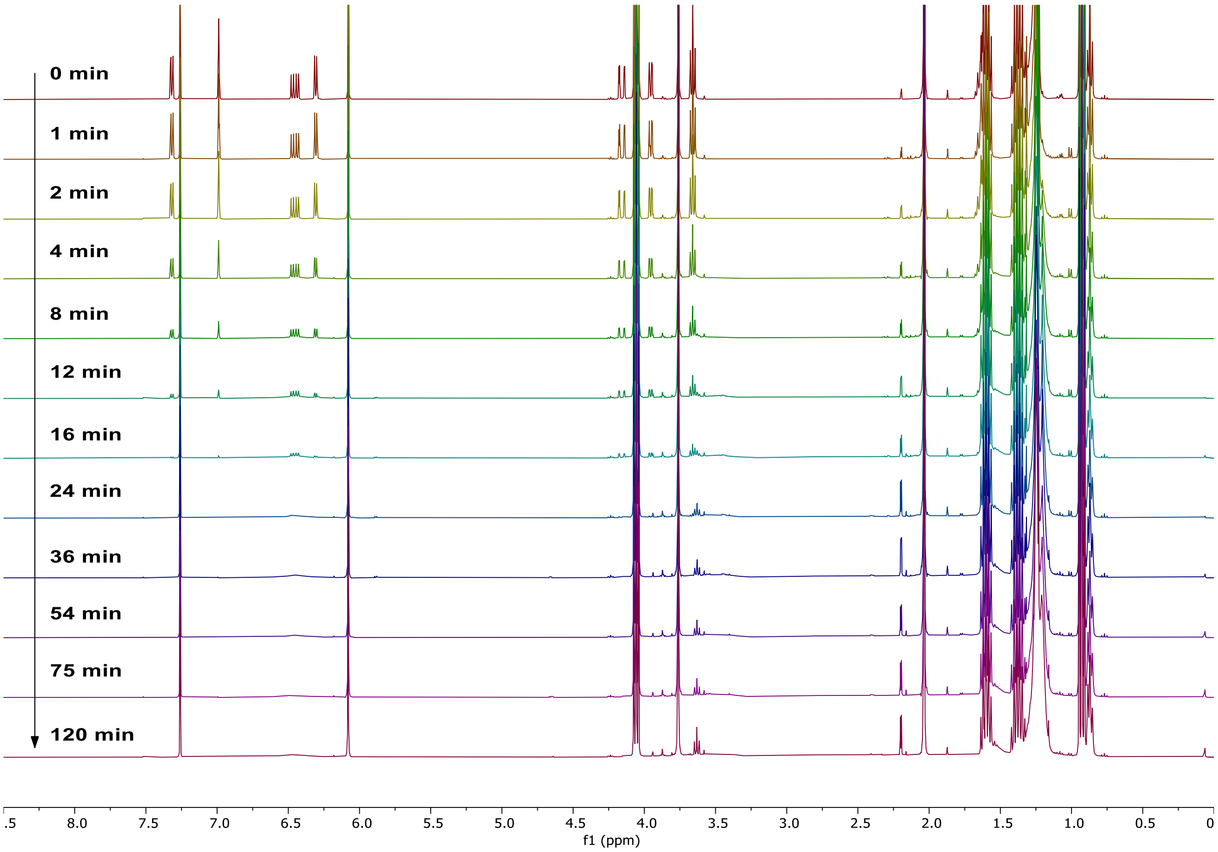

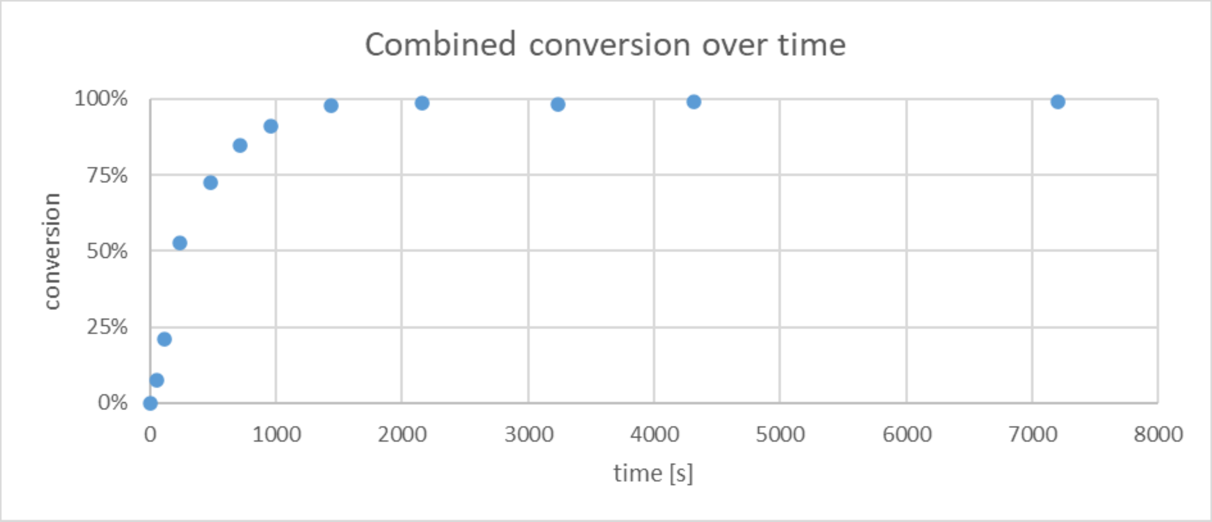
Combined conversion over time
conversion
100%
75%
50%
25%
0%
0
1000
2000
3000
4000
5000
6000
7000
8000
time [s]

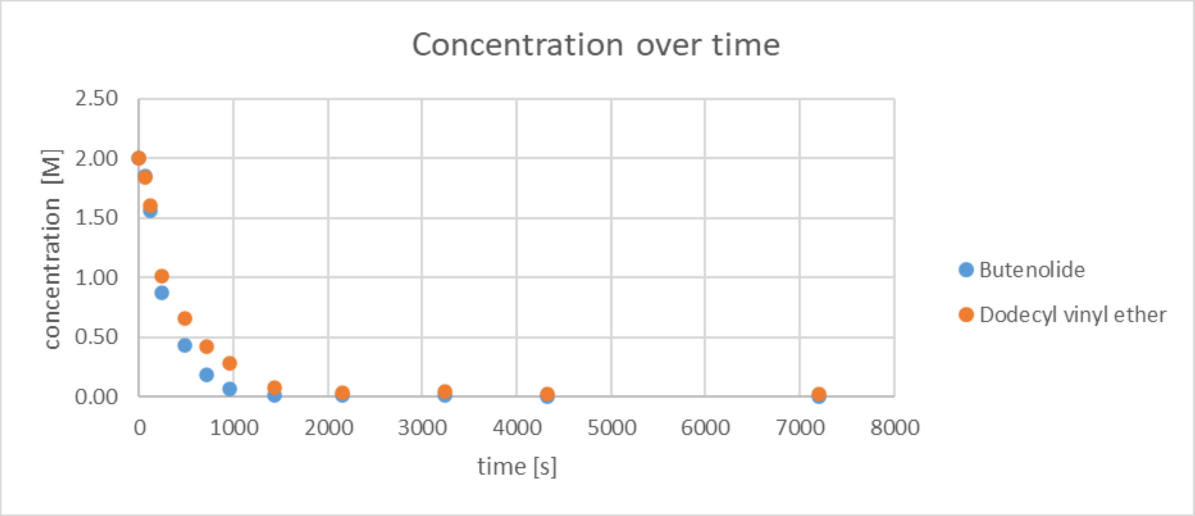
Concentration over time
concentration [M]
2.50
2.00
1.50
1.00
0.50
0.00
0
1000
2000
3000
4000
5000
6000
7000
8000
time [s]
Butenolide
Dodecyl vinyl ether

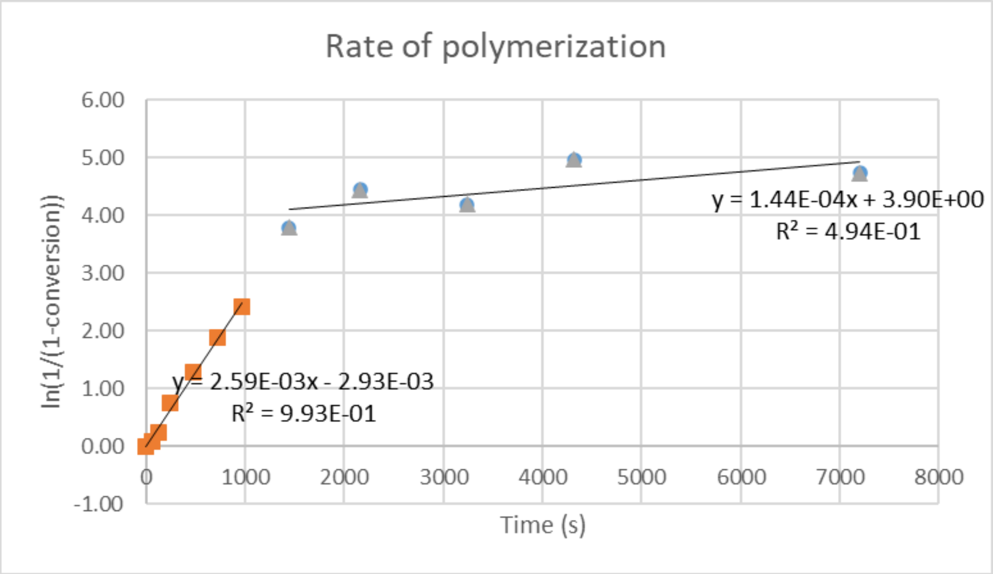
Rate of polymerization
ln(1/(1-conversion))
6.00
5.00
4.00
3.00
2.00
1.00
0.00
-1.00
0
1000
2000
3000
4000
5000
6000
7000
8000
Time (s)
y = 1.44E-04x + 3.90E+00
R² = 4.94E-01
y = 2.59E-03x - 2.93E-03
R² = 9.93E-01

Benzoyloxy butenolide and DVE in AcOBu

Poly(2d-co-DVE)					k1(obs)	3.68 x 10^{-3} [s^{-1}]
	2d		Dodecyl vinyl ether		k2(obs)	n/a
Time [s]	Integral	Concentration [mol/L]	Integral	Concentration [mol/L]	Conversion	ln(1/[1-conversion])
0	0.98	2.00	0.96	2.00	0%	0.00
60	0.88	1.79	0.74	1.54	17%	0.18
120	0.68	1.39	0.57	1.19	36%	0.44
240	0.40	0.82	0.37	0.78	60%	0.92
480	0.16	0.34	0.17	0.36	82%	1.74
900	0.05	0.11	0.09	0.19	92%	2.58
1800	0.02	0.04	0.07	0.14	95%	3.10
3600	0.02	0.04	0.06	0.12	96%	3.24
7200	0.02	0.04	0.06	0.13	96%	3.18

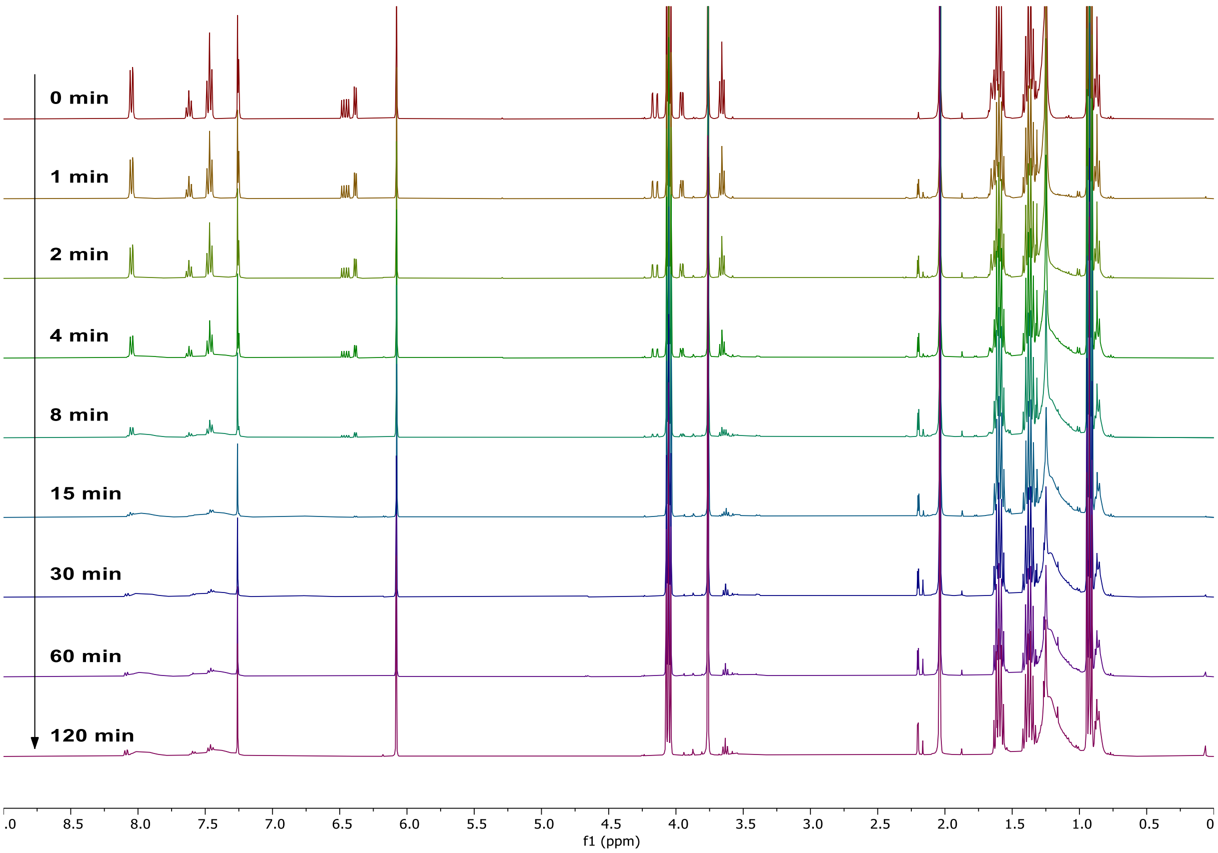

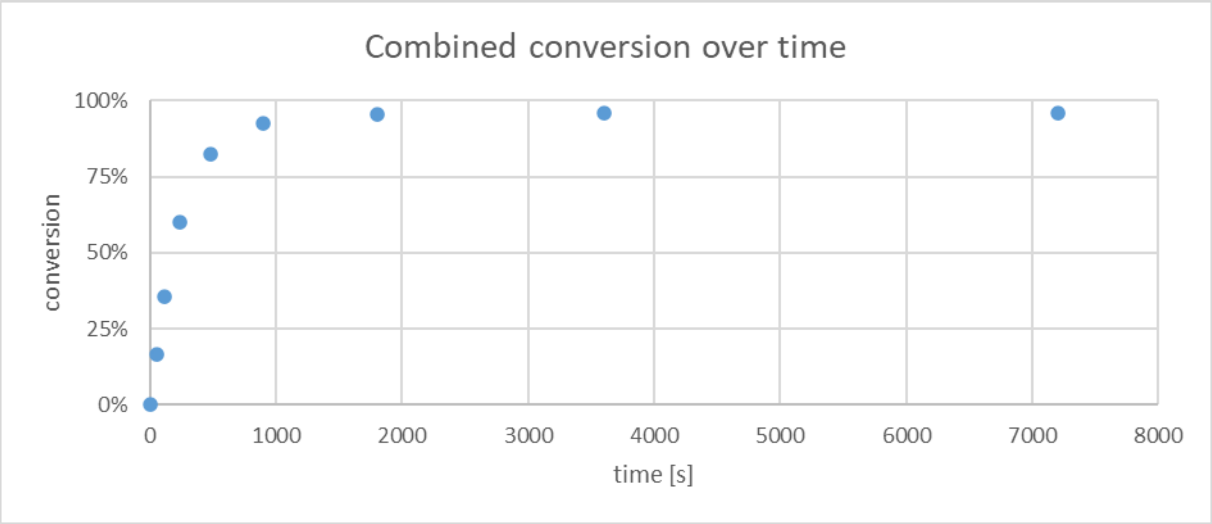
Combined conversion over time
conversion
100%
75%
50%
25%
0%
0
1000
2000
3000
4000
5000
6000
7000
8000
time [s]

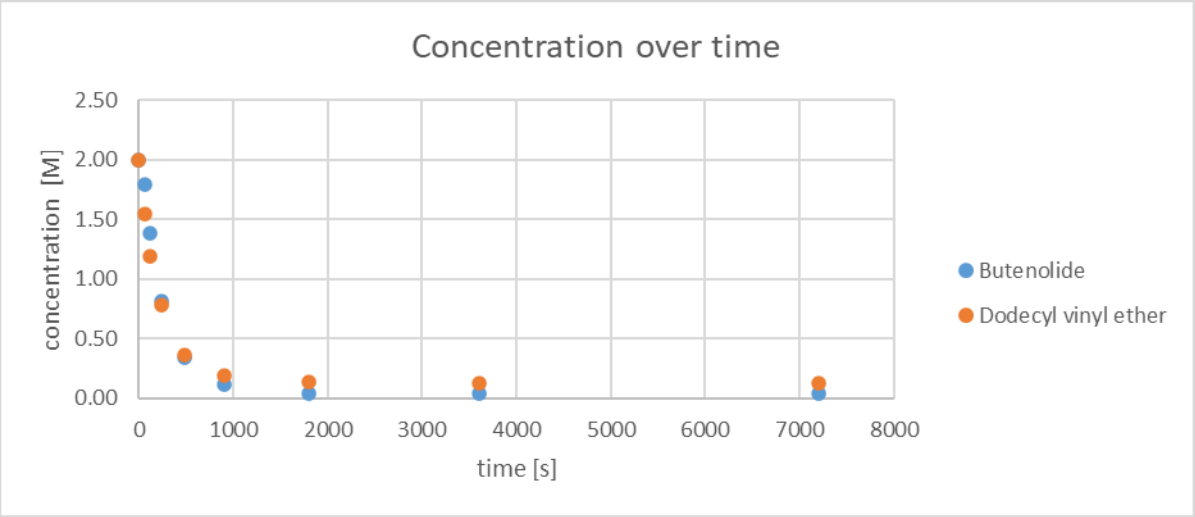
Concentration over time
concentration [M]
2.50
2.00
1.50
1.00
0.50
0.00
0
1000
2000
3000
4000
5000
6000
7000
8000
time [s]
Butenolide
Dodecyl vinyl ether

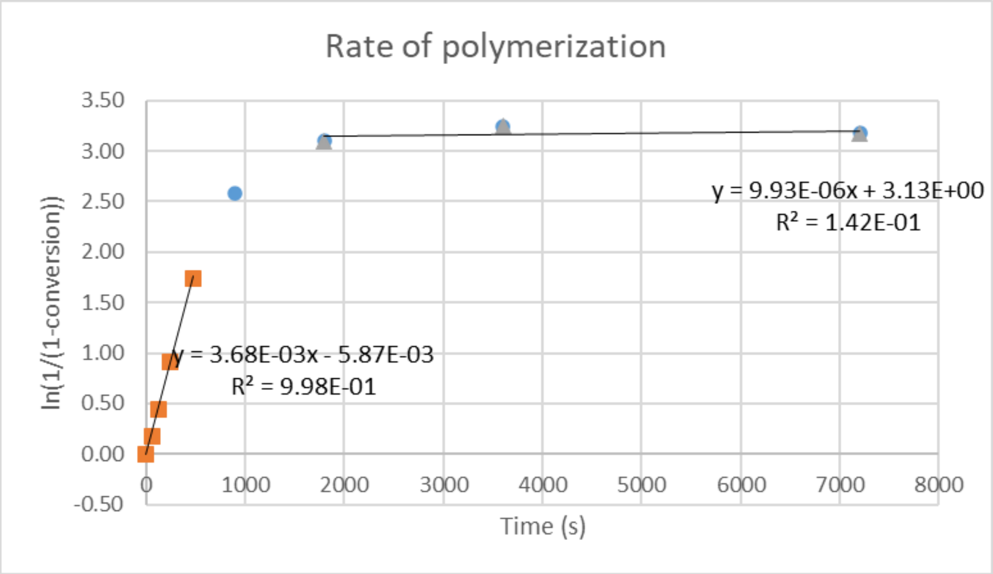
Rate of polymerization
ln(1/(1-conversion))
3.50
3.00
2.50
2.00
1.50
1.00
0.50
0.00
-0.50
0
1000
2000
3000
4000
5000
6000
7000
8000
Time (s)
y = 9.93E-06x + 3.13E+00
R² = 1.42E-01
y = 3.68E-03x - 5.87E-03
R² = 9.98E-01

Benzoyloxy butenolide (2d) and DVE in 1-methoxy-2-propanol

Poly(2d-co-DVE)					k1(obs)	4.48 . 10^{-3} [s^{-1}]
	2d		Dodecyl vinyl ether		k2(obs)	1.85 . 10^{-4} [s^{-1}]
Time [s]	Integral	Concentration [mol/L]	Integral	Concentration [mol/L]	Conversion	ln(1/[1-conversion])
0	0.96	2.00	0.99	2.00	0%	0.00
60	0.70	1.46	0.78	1.59	24%	0.27
120	0.48	1.00	0.62	1.25	44%	0.58
240	0.24	0.51	0.44	0.89	65%	1.05
480	0.10	0.22	0.32	0.65	78%	1.53
720	0.05	0.11	0.26	0.54	84%	1.82
960	0.03	0.06	0.23	0.47	87%	2.02
1440	0.01	0.03	0.19	0.38	90%	2.28
2160	0.01	0.02	0.15	0.30	92%	2.52
3120	0.01	0.02	0.12	0.24	94%	2.75
7200	0.01	0.02	0.06	0.12	97%	3.37

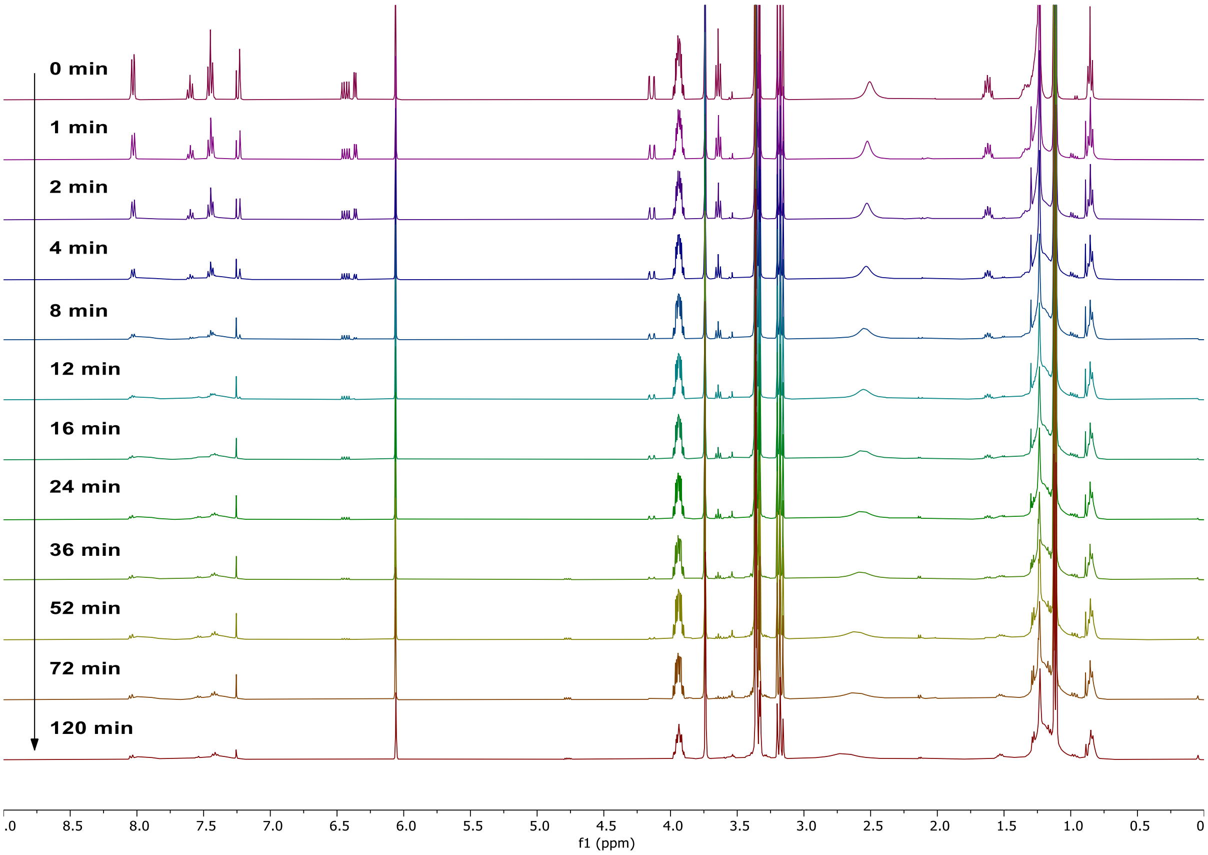

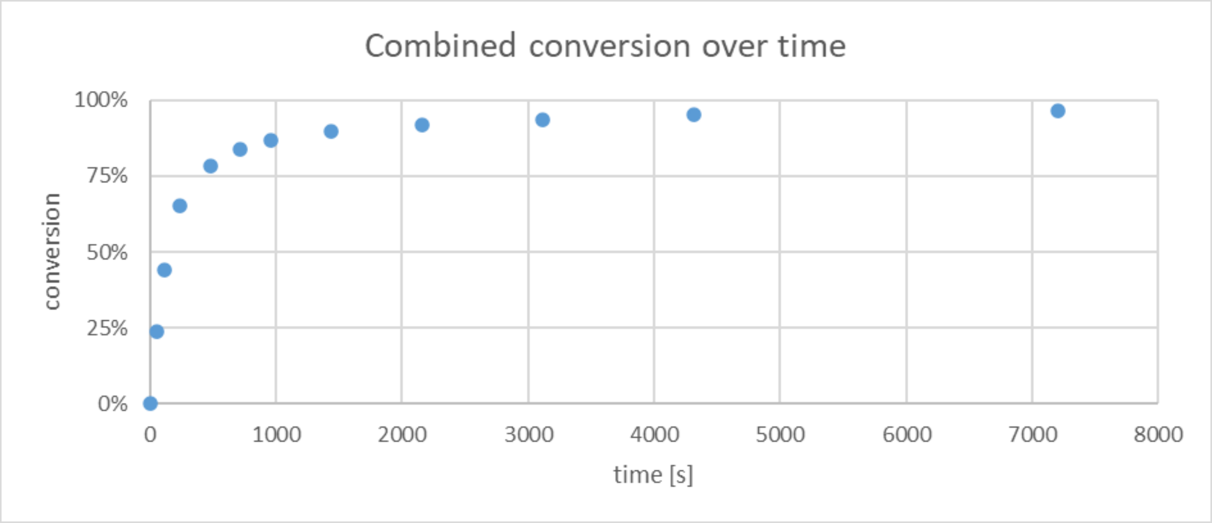
Combined conversion over time
100%
75%
50%
25%
0%
conversion
0
1000
2000
3000
4000
5000
6000
7000
8000
time [s]

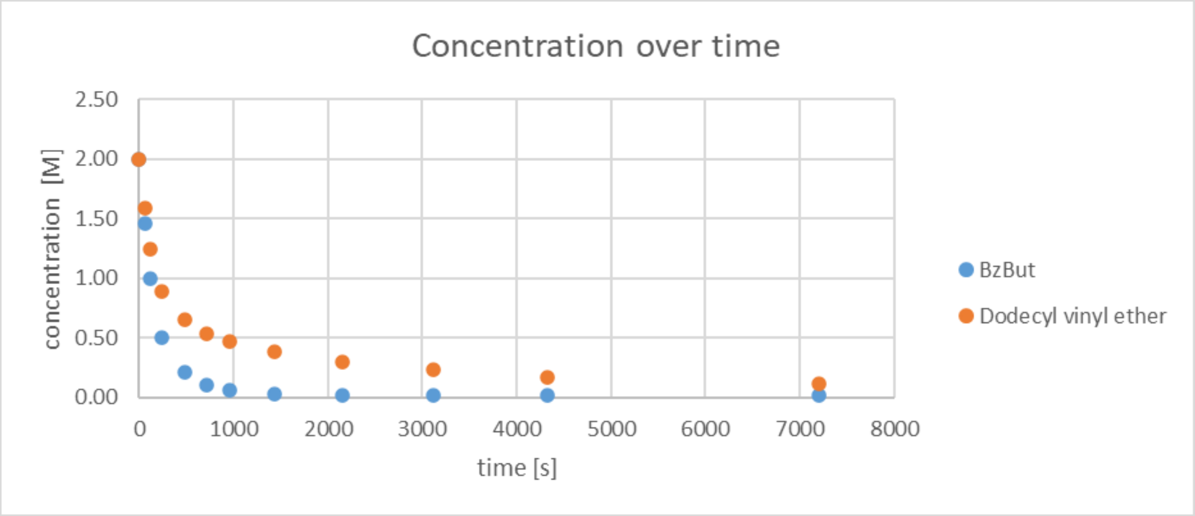
Concentration over time
2.50
2.00
1.50
1.00
0.50
0.00
concentration [M]
0
1000
2000
3000
4000
5000
6000
7000
8000
time [s]
BzBut
Dodecyl vinyl ether

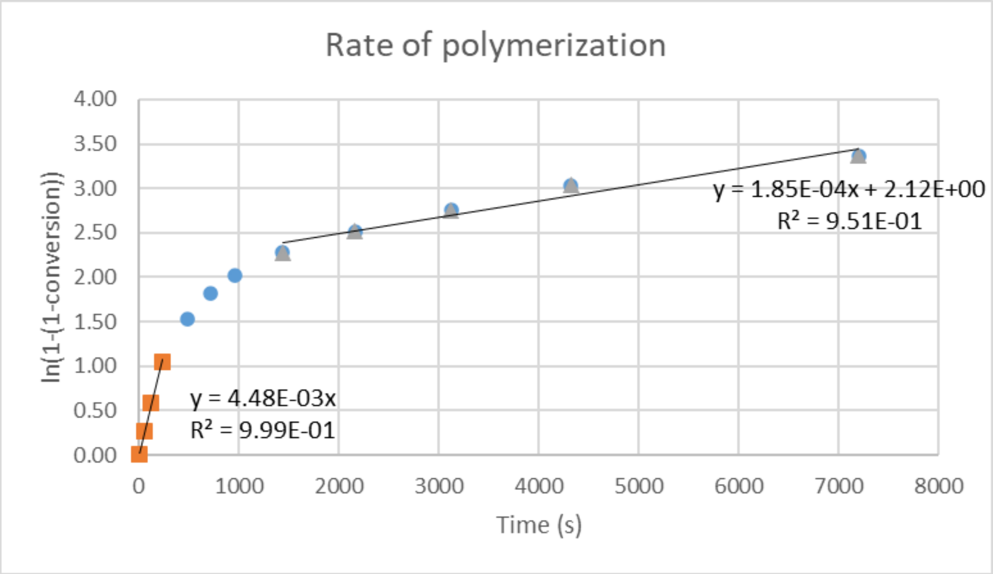
Rate of polymerization
4.00
3.50
3.00
2.50
2.00
1.50
1.00
0.50
0.00
ln(1-(1-conversion))
y = 1.85E-04x + 2.12E+00
R² = 9.51E-01
y = 4.48E-03x
R² = 9.99E-01
0
1000
2000
3000
4000
5000
6000
7000
8000
Time (s)

Lauryloxy butenolide (2e) and DVE in AcOBu

Poly(2e-co-DVE) in AcOBu					k1(obs)	2.66 x 10^{-3} [s^{-1}]
	2e		Dodecyl vinyl ether		k2(obs)	n/a
Time [s]	Integral	Concentration [mol/L]	Integral	Concentration [mol/L]	Conversion	ln(1/[1-conversion])
0	0.94	2.00	0.91	2.00	0%	0.00
60	0.85	1.81	0.80	1.76	11%	0.11
120	0.72	1.53	0.70	1.54	23%	0.26
240	0.46	0.98	0.49	1.09	48%	0.66
480	0.22	0.47	0.31	0.68	71%	1.24
900	0.06	0.12	0.19	0.41	87%	2.02
1800	0.01	0.01	0.03	0.07	98%	3.81
2700	0.01	0.01	0.02	0.03	99%	4.47
3600	0.01	0.01	0.02	0.05	98%	4.11
5400	0.00	0.01	0.03	0.06	98%	4.01
7200	0.01	0.01	0.03	0.06	98%	4.06

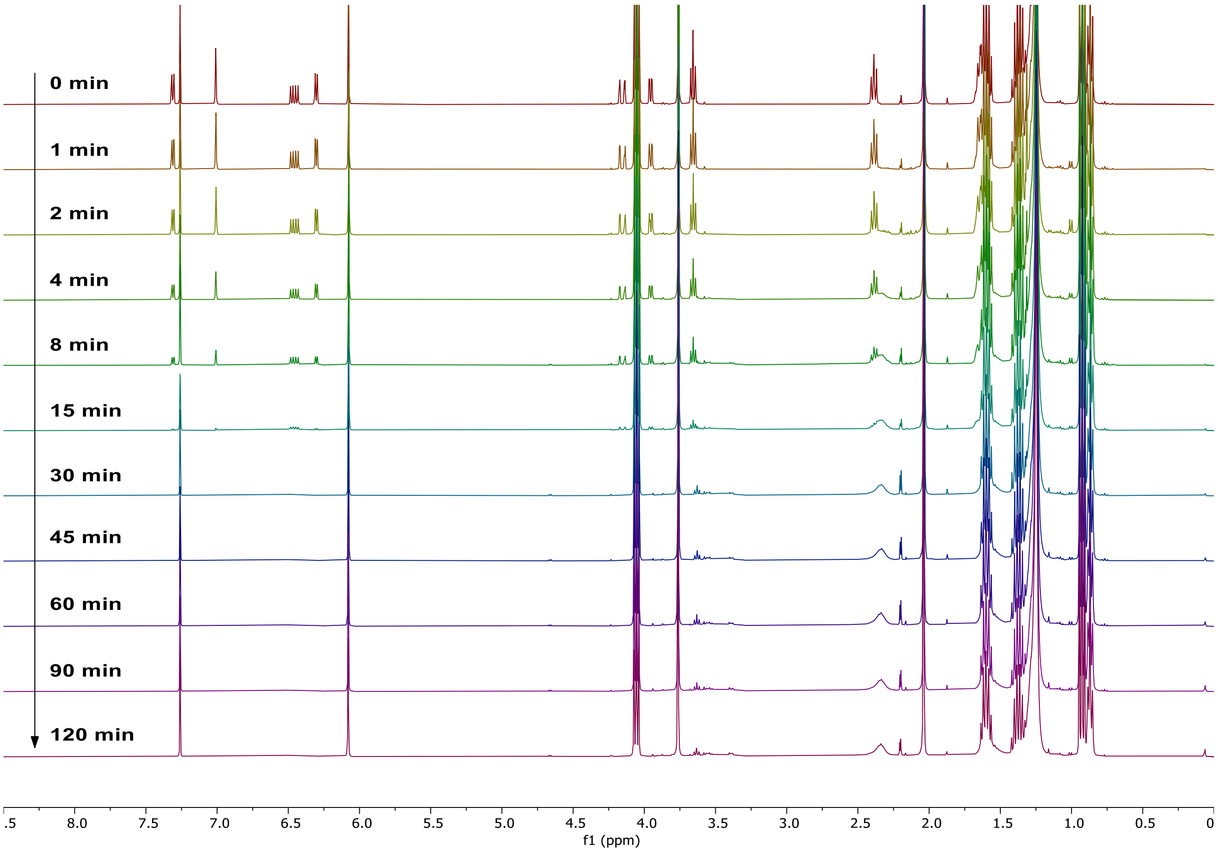

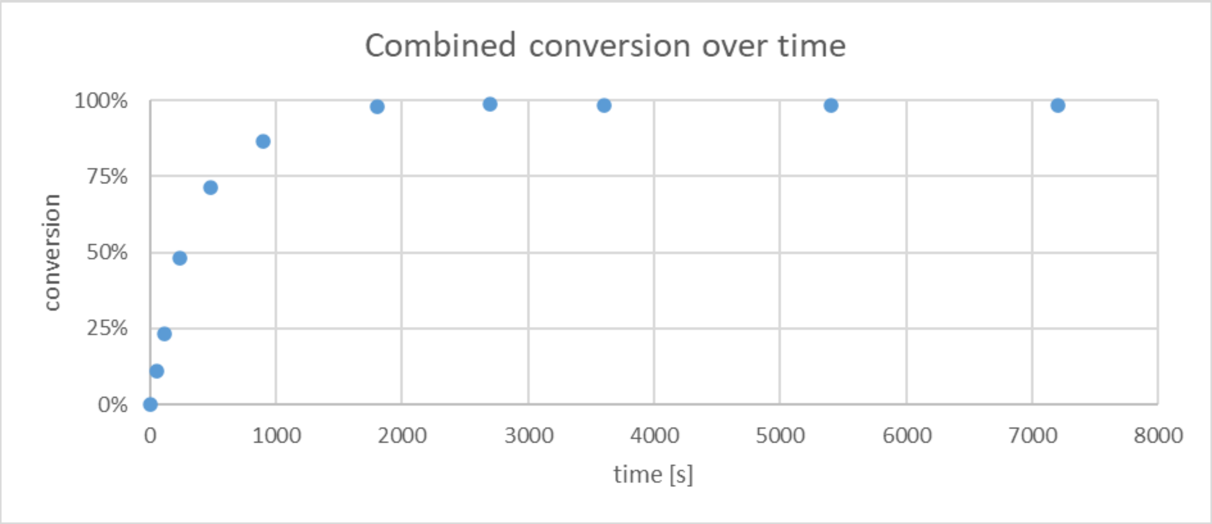

Combined conversion over time
conversion
100%
75%
50%
25%
0%
0
1000
2000
3000
4000
5000
6000
7000
8000
time [s]

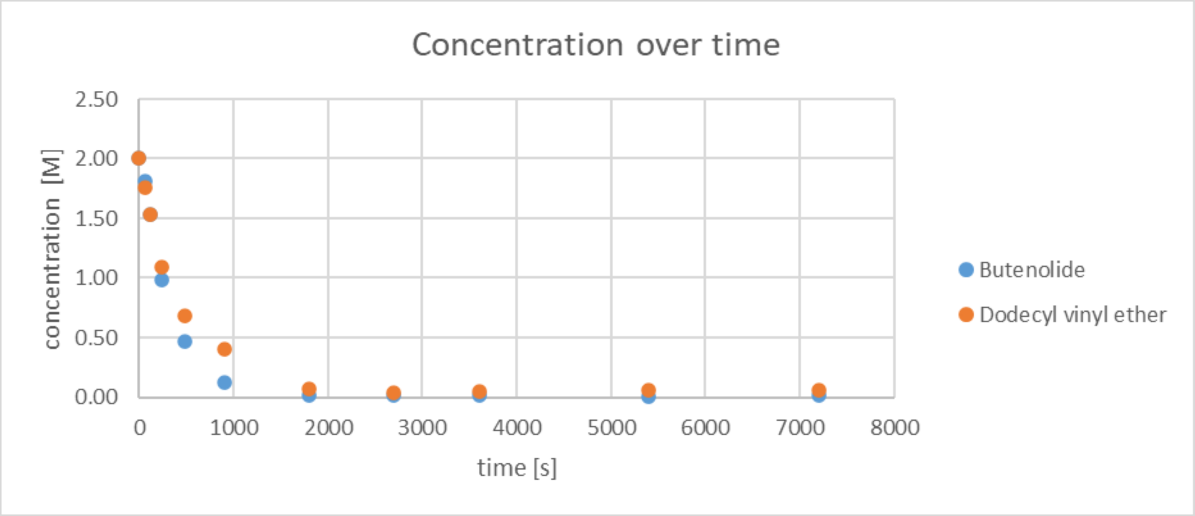

Concentration over time
concentration [M]
2.50
2.00
1.50
1.00
0.50
0.00
0
1000
2000
3000
4000
5000
6000
7000
8000
time [s]
Butenolide
Dodecyl vinyl ether

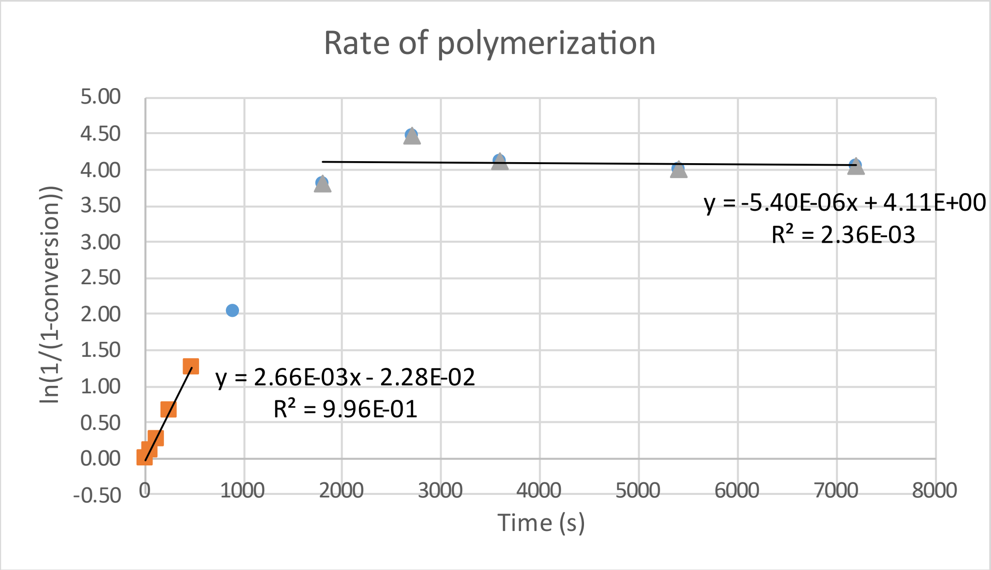

Rate of polymerization
ln(1/(1-conversion))
5.00
4.50
4.00
3.50
3.00
2.50
2.00
1.50
1.00
0.50
0.00
-0.50
0
1000
2000
3000
4000
5000
6000
7000
8000
Time (s)
y = -5.40E-06x + 4.11E+00
R² = 2.36E-03
y = 2.66E-03x - 2.28E-02
R² = 9.96E-01

Lauryloxy butenolide (2e) and DVE in 1-methoxy-2-propanol

Poly(2e-co-DVE)					k1(obs)	3.08 . 10^{-3} [s^{-1}]
	2e		Dodecyl vinyl ether		k2(obs)	1.68 . 10^{-4} [s^{-1}]
Time [s]	Integral	Concentration [mol/L]	Integral	Concentration [mol/L]	Conversion	ln(1/[1-conversion])
0	0.96	2.00	1.05	2.00	0%	0.00
60	0.84	1.75	0.64	1.22	26%	0.30
120	0.71	1.47	0.54	1.02	38%	0.47
240	0.48	0.99	0.35	0.67	58%	0.88
480	0.30	0.62	0.21	0.39	75%	1.37
720	0.21	0.44	0.16	0.30	82%	1.70
960	0.15	0.32	0.12	0.23	86%	1.98
1440	0.08	0.17	0.10	0.19	91%	2.41
2160	0.03	0.06	0.11	0.21	93%	2.71
3360	0.01	0.02	0.11	0.20	94%	2.87
5400	0.01	0.02	0.11	0.21	94%	2.84

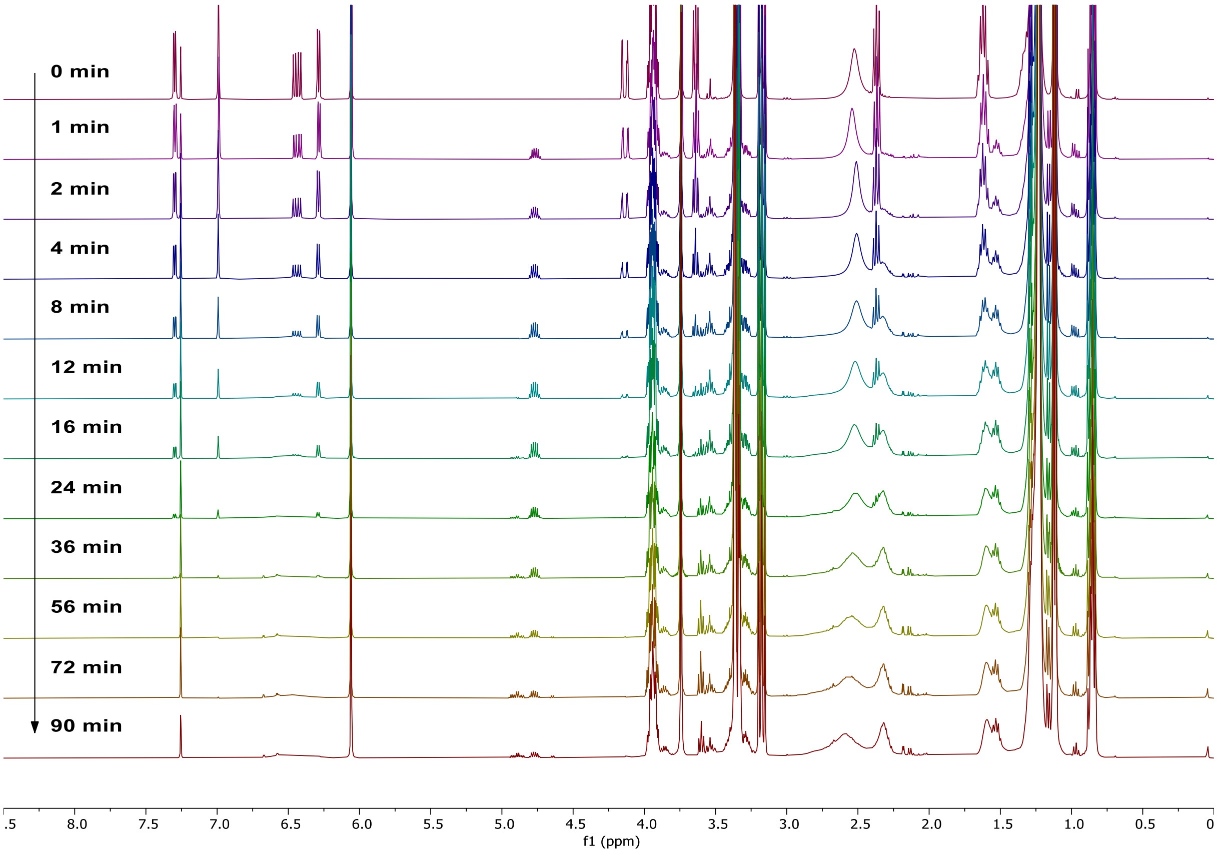

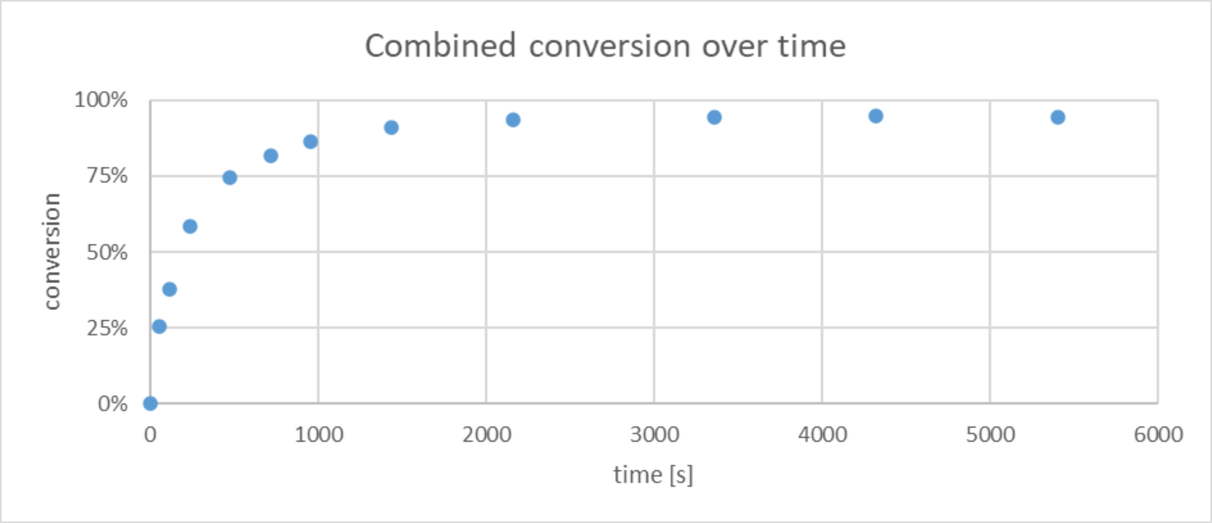
Combined conversion over time
conversion
100%
75%
50%
25%
0%
0
1000
2000
3000
4000
5000
6000
time [s]

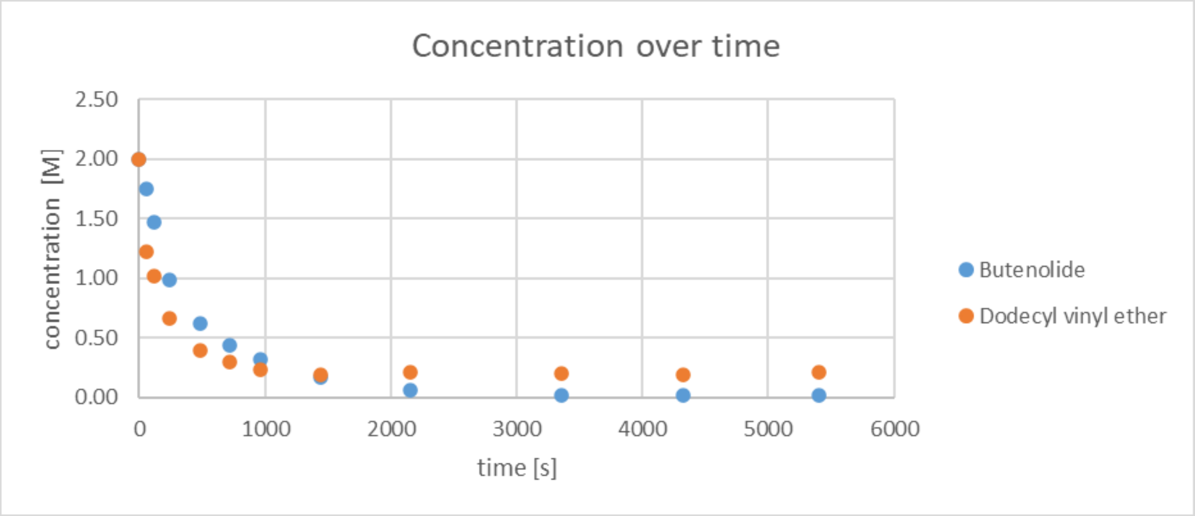
Concentration over time
concentration [M]
2.50
2.00
1.50
1.00
0.50
0.00
0
1000
2000
3000
4000
5000
6000
time [s]
Butenolide
Dodecyl vinyl ether

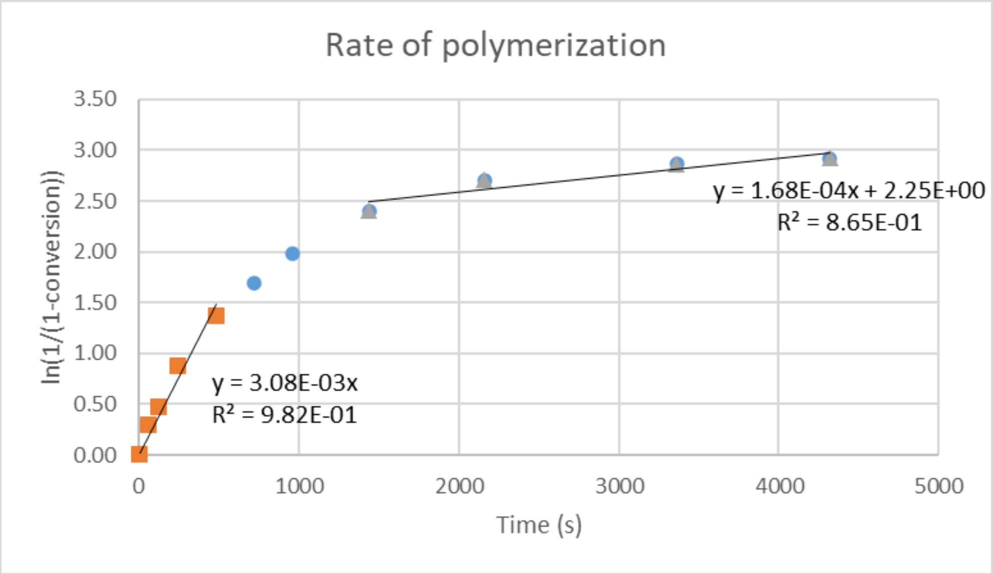
Rate of polymerization
ln(1/(1-conversion))
3.50
3.00
2.50
2.00
1.50
1.00
0.50
0.00
0
1000
2000
3000
4000
5000
Time (s)
y = 1.68E-04x + 2.25E+00
R² = 8.65E-01
y = 3.08E-03x
R² = 9.82E-01

Oleyloxy butenolide (2f) and DVE in AcOBu

Poly(2f-co-DVE) in AcOBu					k1(obs)	7.71 x 10^{-4} [s^{-1}]
	2f		Dodecyl vinyl ether		k2(obs)	2.36 x 10^{-4} [s^{-1}]
Time [s]	Integral	Concentration [mol/L]	Integral	Concentration [mol/L]	Conversion	ln(1/[1-conversion])
0	0.95	2.00	0.96	2.00	0%	0.00
60	0.95	1.98	0.96	2.01	0%	0.00
120	0.92	1.93	0.92	1.93	3%	0.03
240	0.81	1.70	0.83	1.74	14%	0.15
480	0.65	1.37	0.73	1.52	28%	0.32
720	0.53	1.11	0.61	1.27	41%	0.52
960	0.41	0.86	0.53	1.10	51%	0.71
1800	0.16	0.33	0.33	0.70	74%	1.35
2160	0.11	0.23	0.28	0.58	80%	1.61
2700	0.07	0.14	0.28	0.58	82%	1.72
3600	0.03	0.07	0.18	0.38	89%	2.20
5400	0.01	0.02	0.11	0.23	94%	2.76
7200	0.01	0.01	0.12	0.26	93%	2.69

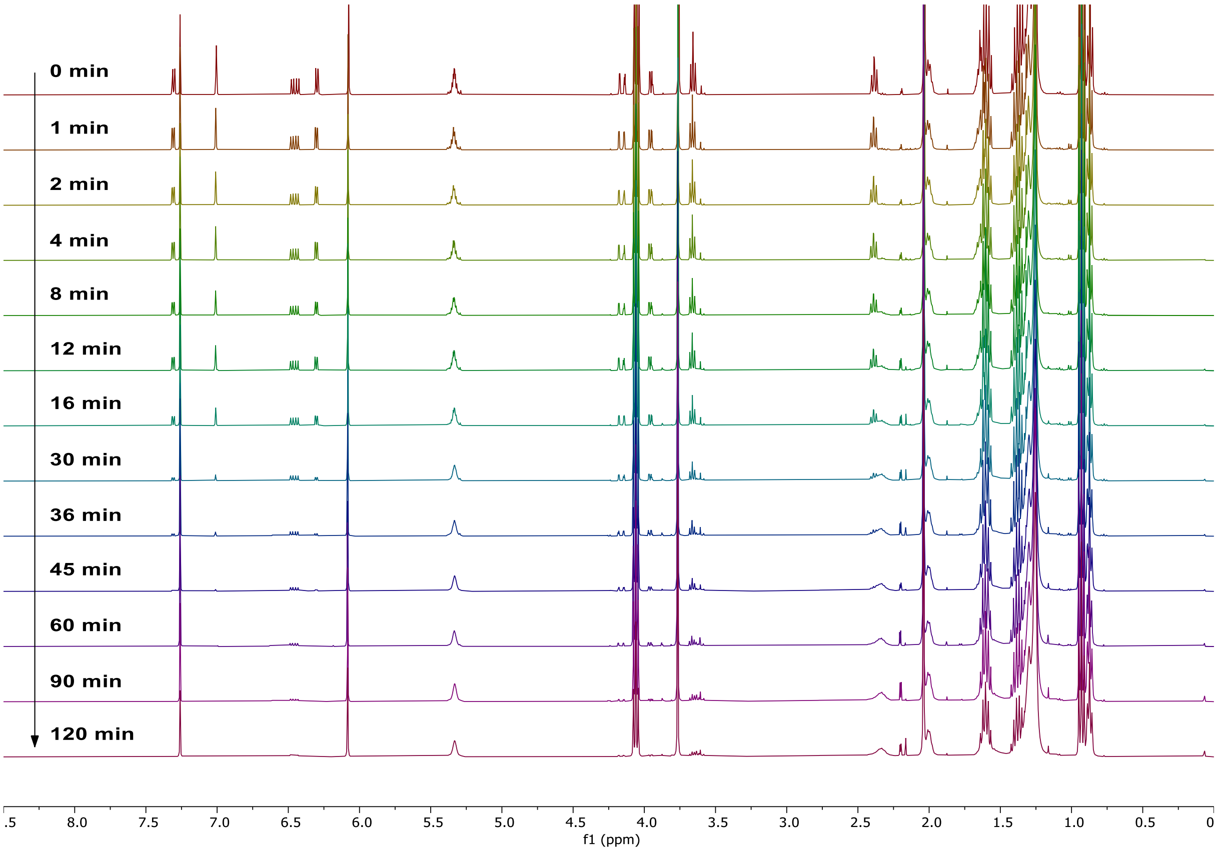

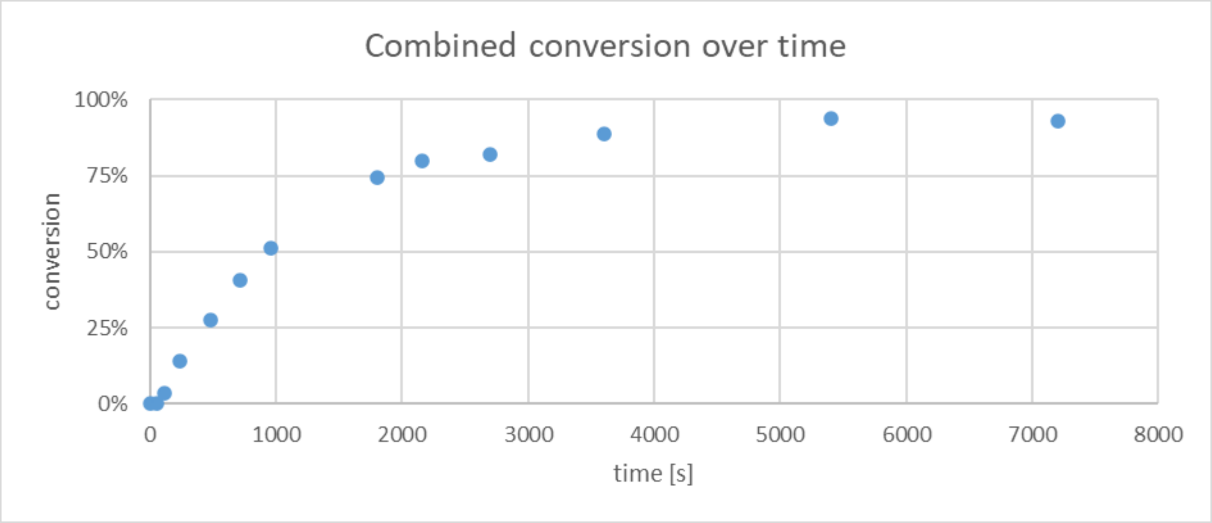
Combined conversion over time
conversion
100%
75%
50%
25%
0%
0
1000
2000
3000
4000
5000
6000
7000
8000
time [s]

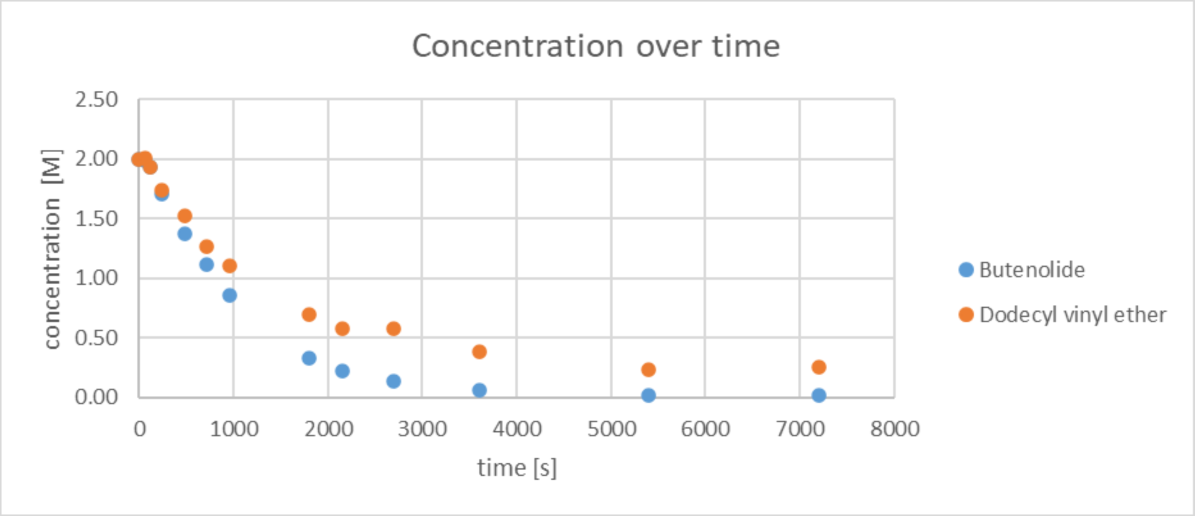
Concentration over time
concentration [M]
2.50
2.00
1.50
1.00
0.50
0.00
0
1000
2000
3000
4000
5000
6000
7000
8000
time [s]
Butenolide
Dodecyl vinyl ether

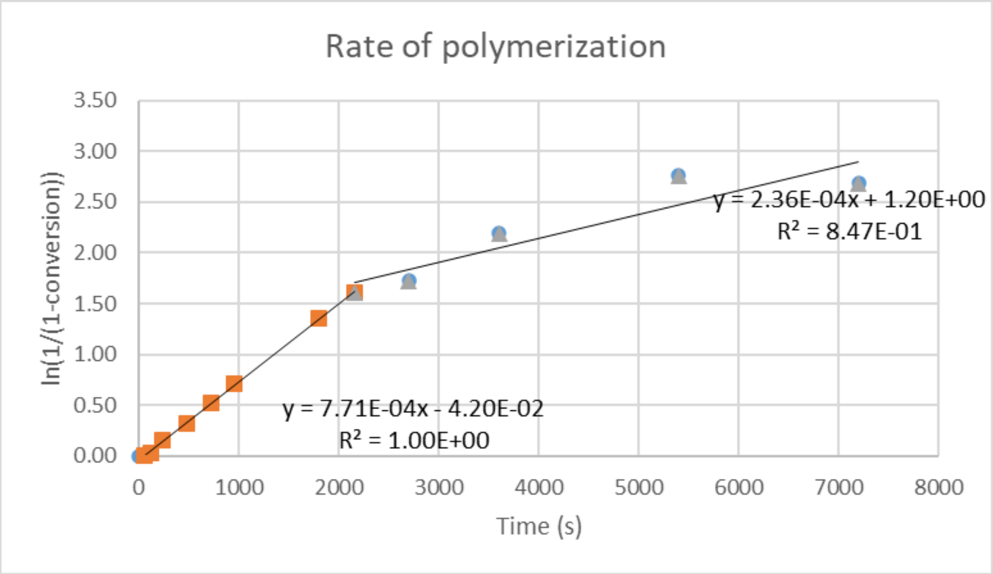
Rate of polymerization
ln(1/(1-conversion))
3.50
3.00
2.50
2.00
1.50
1.00
0.50
0.00
0
1000
2000
3000
4000
5000
6000
7000
8000
Time (s)
y = 2.36E-04x + 1.20E+00
R² = 8.47E-01
y = 7.71E-04x - 4.20E-02
R² = 1.00E+00

Methyl succinyloxy butenolide (2h) and DVE in AcOBu

Poly(2h-co-DVE) in AcOBu					k1(obs)	3.25×10^{-3} [s-1]
	2h		Dodecyl vinyl ether		k2(obs)	1.03×10^{-5} [s-1]
Time [s]	Integral	Concentration [mol/L]	Integral	Concentration [mol/L]	Conversion	ln(1/[1-conversion])
0	0.93	2.00	0.87	2.00	0%	0.00
120	0.64	1.38	0.62	1.43	30%	0.35
240	0.40	0.85	0.42	0.97	54%	0.79
480	0.16	0.34	0.22	0.51	79%	1.55
960	0.02	0.04	0.10	0.23	93%	2.67
1800	0.00	0.01	0.02	0.03	99%	4.50
7200	0.00	0.01	0.01	0.03	99%	4.55

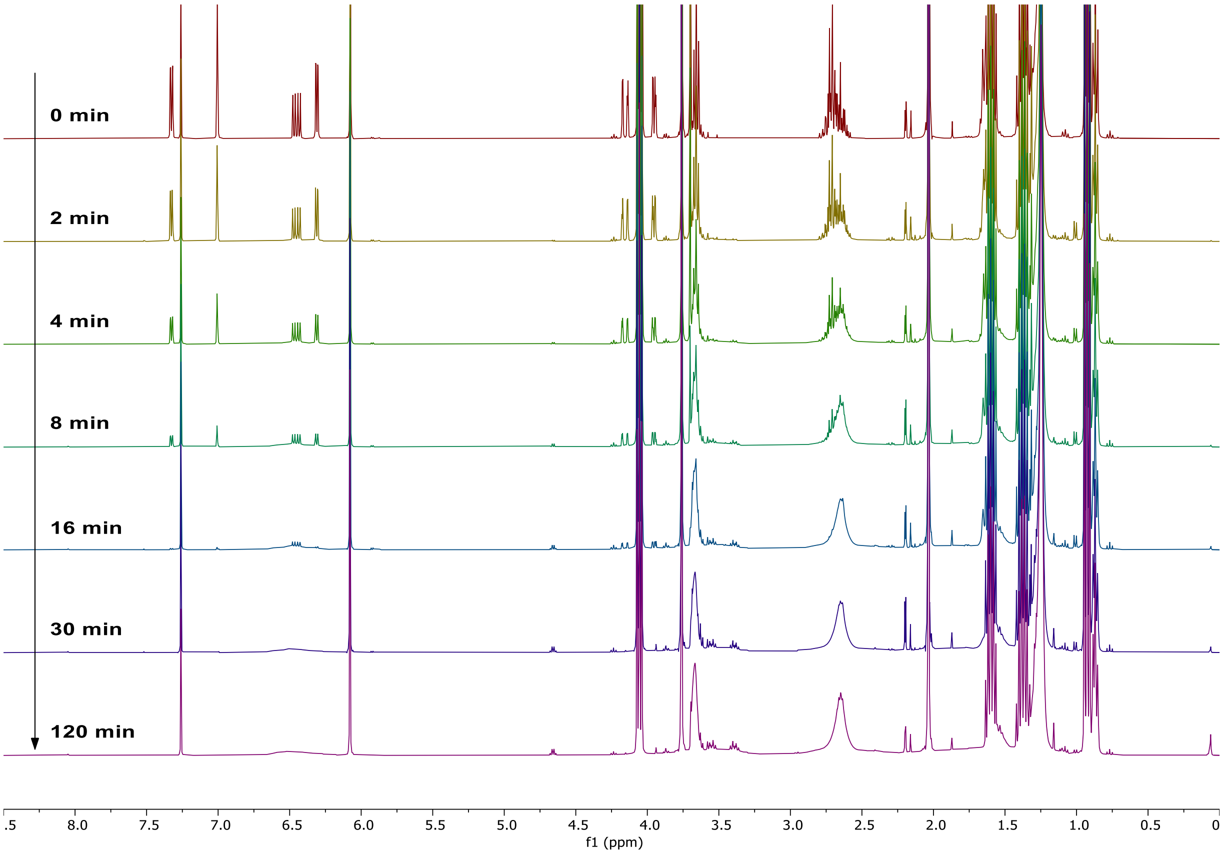

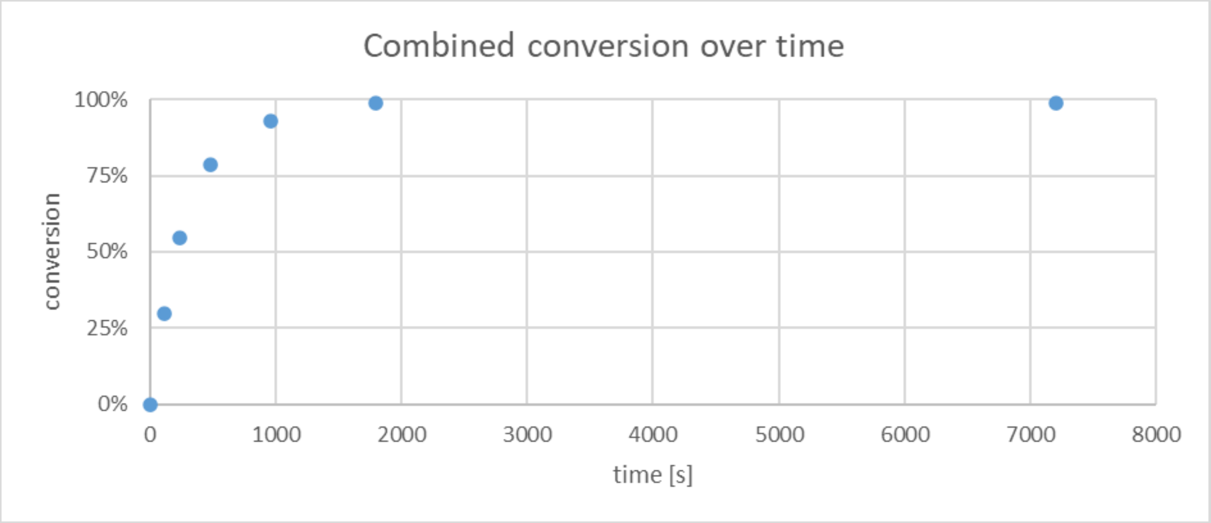
Combined conversion over time
conversion
100%
75%
50%
25%
0%
0
1000
2000
3000
4000
5000
6000
7000
8000
time [s]

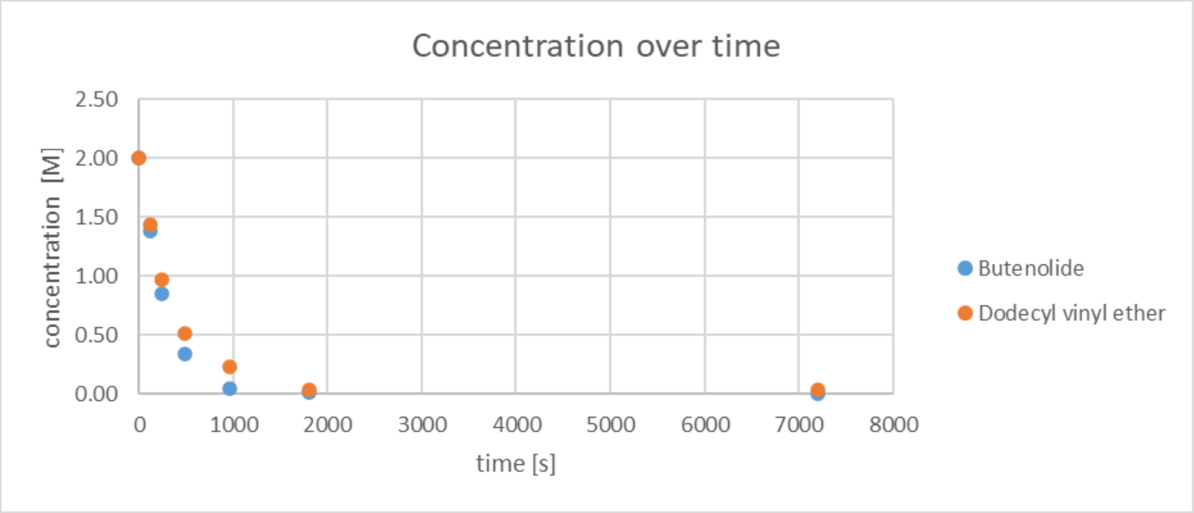
Concentration over time
concentration [M]
2.50
2.00
1.50
1.00
0.50
0.00
0
1000
2000
3000
4000
5000
6000
7000
8000
time [s]
Butenolide
Dodecyl vinyl ether

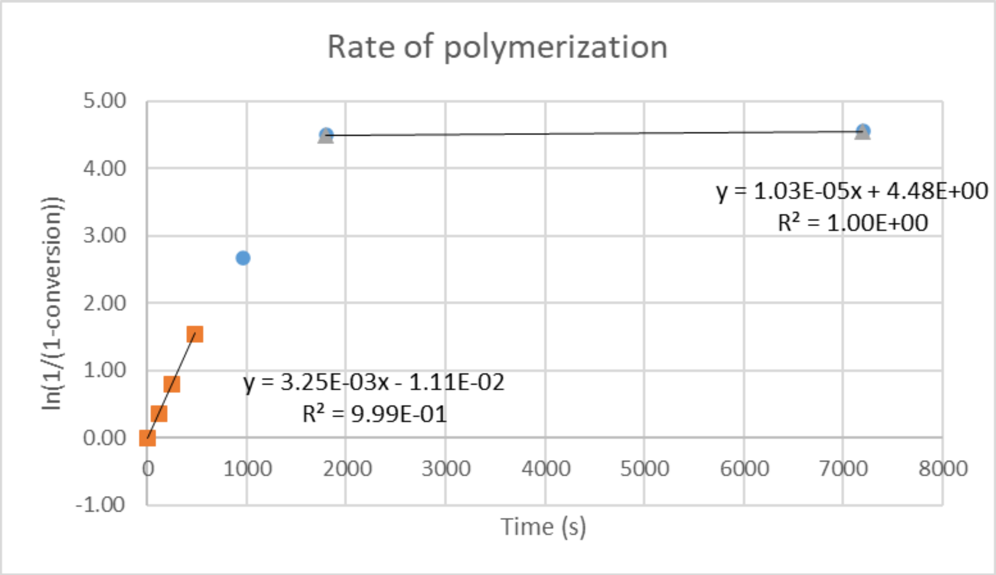
Rate of polymerization
ln(1/(1-conversion))
5.00
4.00
3.00
2.00
1.00
0.00
-1.00
0
1000
2000
3000
4000
5000
6000
7000
8000
Time (s)
y = 1.03E-05x + 4.48E+00
R² = 1.00E+00
y = 3.25E-03x - 1.11E-02
R² = 9.99E-01

Methyl carbonoxy butenolide (3a) and DVE in AcOBu

Poly(3a-co-DVE)					k1(obs)	2.35×10^{-3} [s^{-1}]
	3a		Dodecyl vinyl ether		k2(obs)	7.08×10^{-5} [s^{-1}]
Time [s]	Integral	Concentration [mol/L]	Integral	Concentration [mol/L]	Conversion	ln(1/[1-conversion])
0	0.96	2.00	0.98	2.00	0%	0.00
60	0.89	1.85	0.93	1.89	7%	0.07
120	0.78	1.64	0.86	1.74	15%	0.17
240	0.45	0.95	0.63	1.27	44%	0.59
480	0.18	0.37	0.43	0.88	69%	1.16
720	0.06	0.14	0.33	0.67	80%	1.61
960	0.02	0.04	0.30	0.61	84%	1.81
1440	0.00	0.01	0.26	0.53	87%	2.02
2160	0.00	0.00	0.24	0.48	88%	2.10
3120	0.00	0.00	0.22	0.44	89%	2.20
7200	0.00	0.00	0.17	0.35	91%	2.44

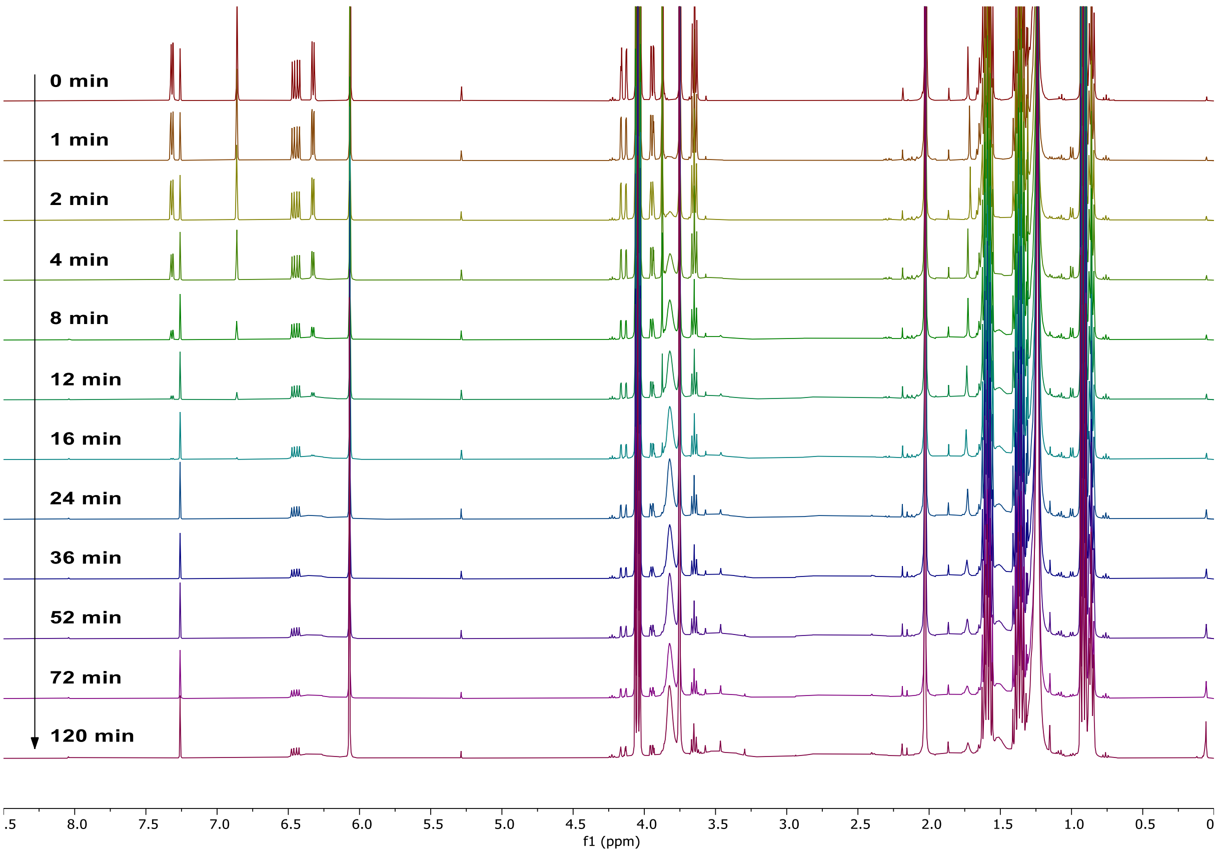

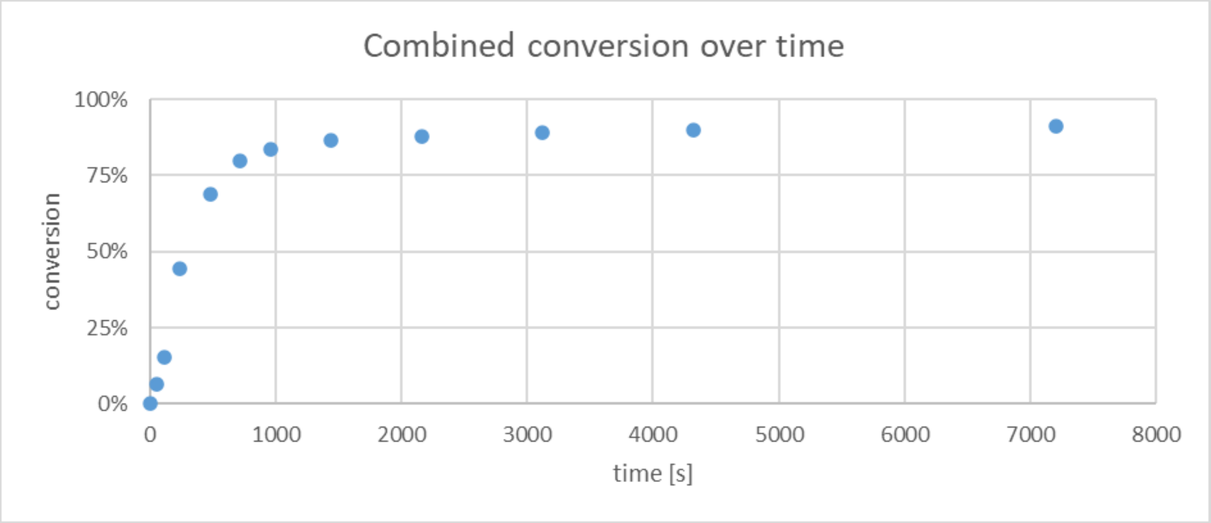

Combined conversion over time
conversion
100%
75%
50%
25%
0%
0
1000
2000
3000
4000
5000
6000
7000
8000
time [s]

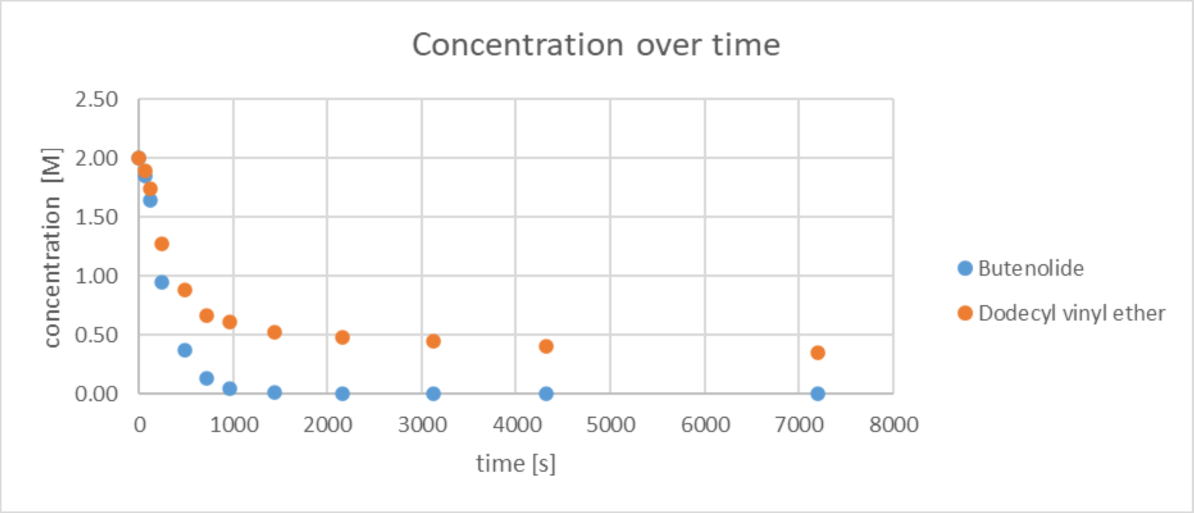

Concentration over time
concentration [M]
2.50
2.00
1.50
1.00
0.50
0.00
0
1000
2000
3000
4000
5000
6000
7000
8000
time [s]
Butenolide
Dodecyl vinyl ether

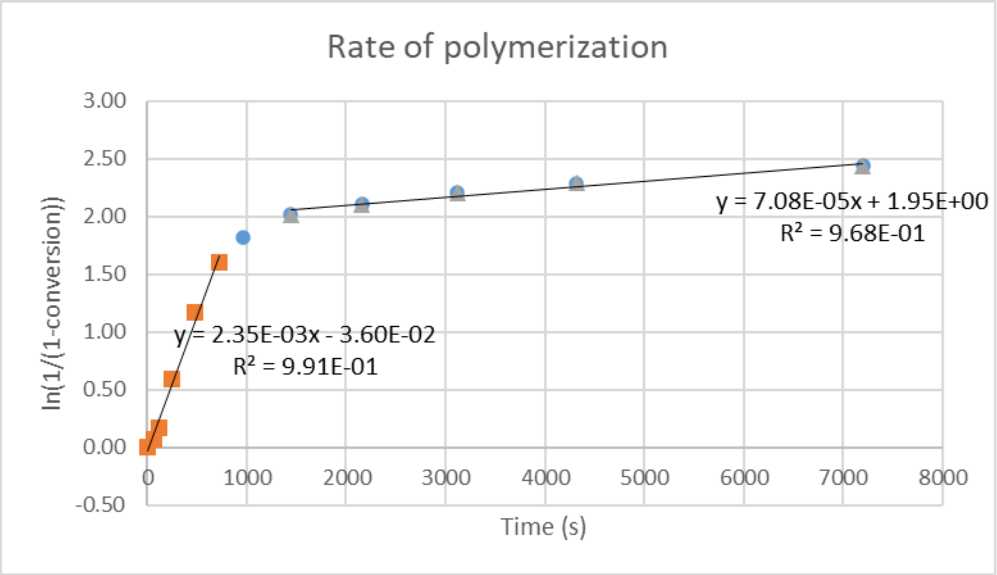

Rate of polymerization
ln(1/(1-conversion))
3.00
2.50
2.00
1.50
1.00
0.50
0.00
-0.50
0
1000
2000
3000
4000
5000
6000
7000
8000
Time (s)
y = 7.08E-05x + 1.95E+00
R² = 9.68E-01
y = 2.35E-03x - 3.60E-02
R² = 9.91E-01

Tert-butyl carbonoxy butenolide (3b) and DVE in AcOBu

Poly(3b-co-DVE) in AcOBu					k1(obs)	1.75×10^{-3} [s^{-1}]
	3b		Dodecyl vinyl ether		k2(obs)	1.34×10^{-4} [s^{-1}]
Time [s]	Integral	Concentration [mol/L]	Integral	Concentration [mol/L]	Conversion	ln(1/[1-conversion])
0	0.86	2.00	0.98	2.00	0%	0.00
60	0.85	1.98	0.97	1.98	1%	0.01
120	0.82	1.91	0.94	1.92	4%	0.04
240	0.68	1.58	0.83	1.70	18%	0.20
480	0.40	0.92	0.61	1.24	46%	0.61
720	0.21	0.49	0.40	0.83	67%	1.11
1020	0.08	0.18	0.32	0.65	79%	1.58
1440	0.01	0.02	0.26	0.52	86%	1.99
2160	0.00	0.00	0.21	0.44	89%	2.21
2880	0.00	0.00	0.21	0.43	89%	2.23
3600	0.00	0.00	0.18	0.36	91%	2.41
4800	0.00	0.00	0.15	0.30	92%	2.57
7200	0.00	0.00	0.12	0.25	94%	2.78

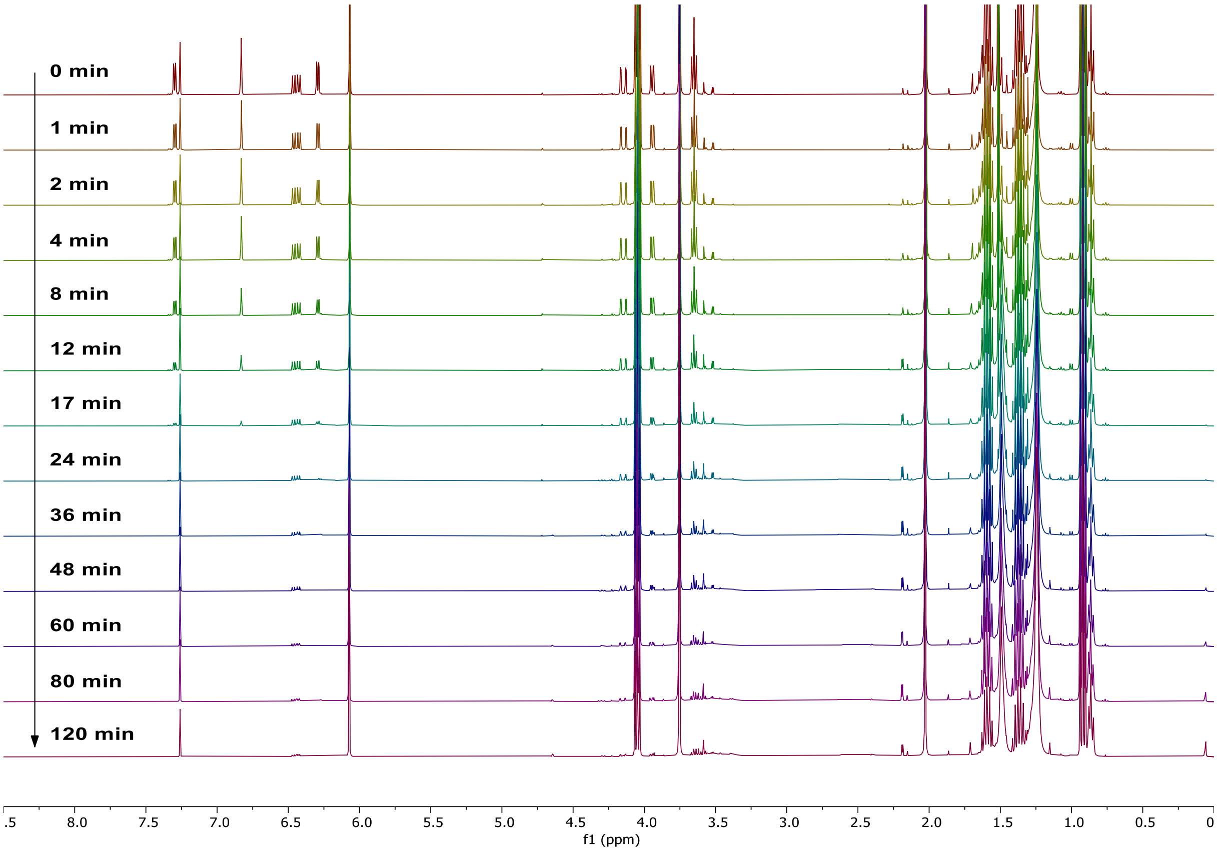

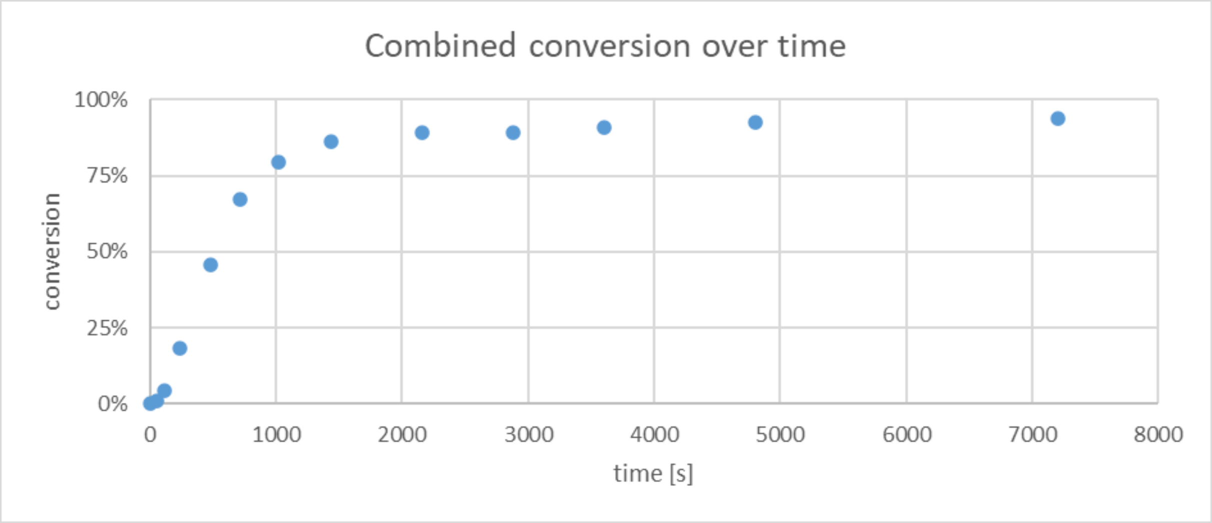
Combined conversion over time
conversion
100%
75%
50%
25%
0%
0
1000
2000
3000
4000
5000
6000
7000
8000
time [s]

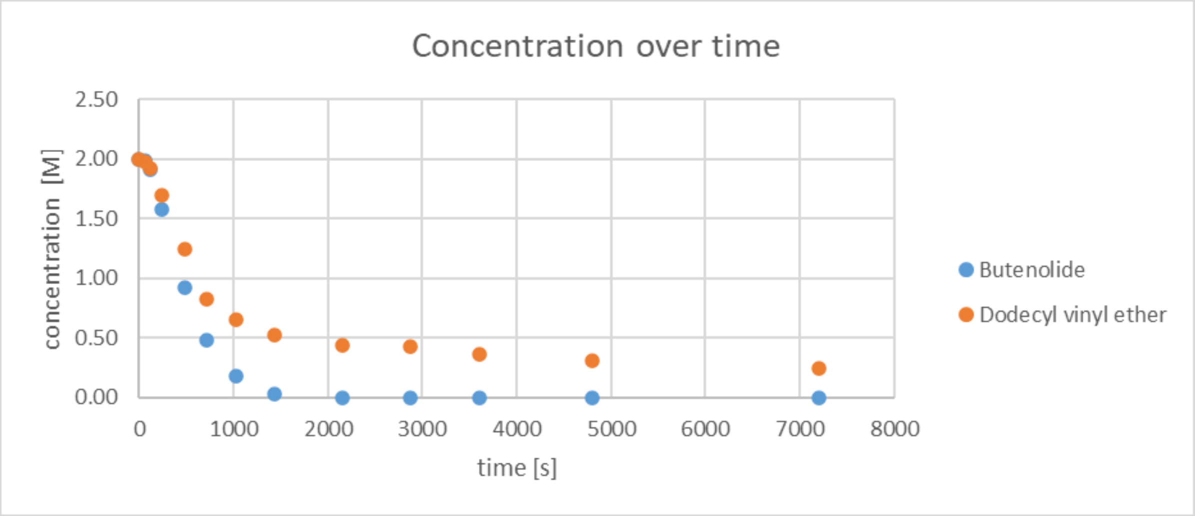
Concentration over time
concentration [M]
2.50
2.00
1.50
1.00
0.50
0.00
0
1000
2000
3000
4000
5000
6000
7000
8000
time [s]
Butenolide
Dodecyl vinyl ether

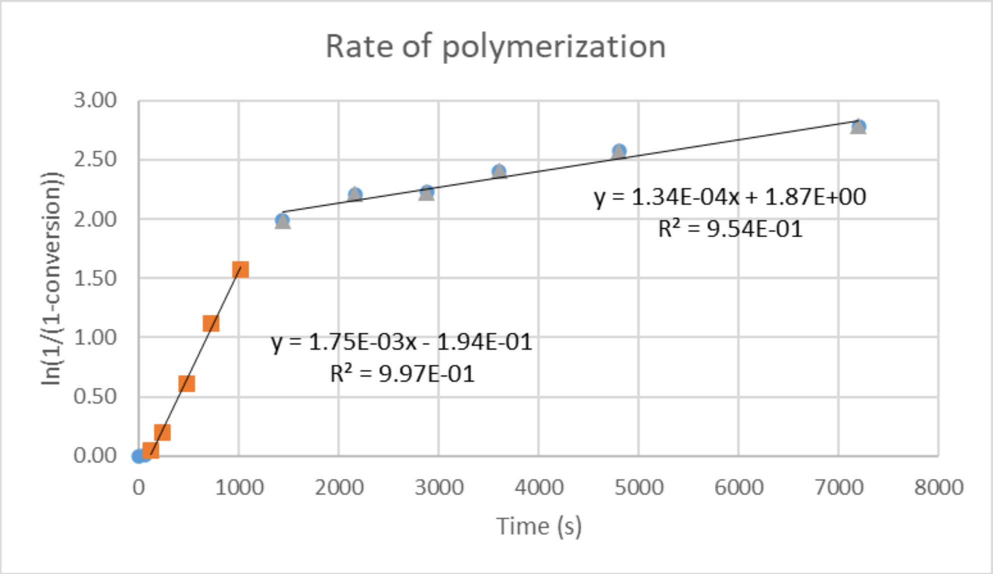
Rate of polymerization
ln(1/(1-conversion))
3.00
2.50
2.00
1.50
1.00
0.50
0.00
0
1000
2000
3000
4000
5000
6000
7000
8000
Time (s)
y = 1.34E-04x + 1.87E+00
R² = 9.54E-01
y = 1.75E-03x - 1.94E-01
R² = 9.97E-01

Dodecyl carbamoxy butenolide (4a) and DVE in AcOBu

Poly(4a-co-DVE) in AcOBu					k1(obs)	1.49 x 10^{-3} [s^{-1}]
	4a		Dodecyl vinyl ether		k2(obs)	6,84 x 10^{-5} [s^{-1}]
Time [s]	Integral	Concentration [mol/L]	Integral	Concentration [mol/L]	Conversion	ln(1/[1-conversion])
0	0.97	2.00	0.94	2.00	0%	0.00
120	0.89	1.82	0.89	1.88	7%	0.08
240	0.64	1.32	0.73	1.56	28%	0.33
480	0.39	0.80	0.57	1.22	50%	0.68
960	0.18	0.37	0.41	0.88	69%	1.17
1800	0.05	0.11	0.32	0.67	80%	1.63
3600	0.02	0.03	0.27	0.57	85%	1.90
7200	0.01	0.03	0.23	0.50	87%	2.03

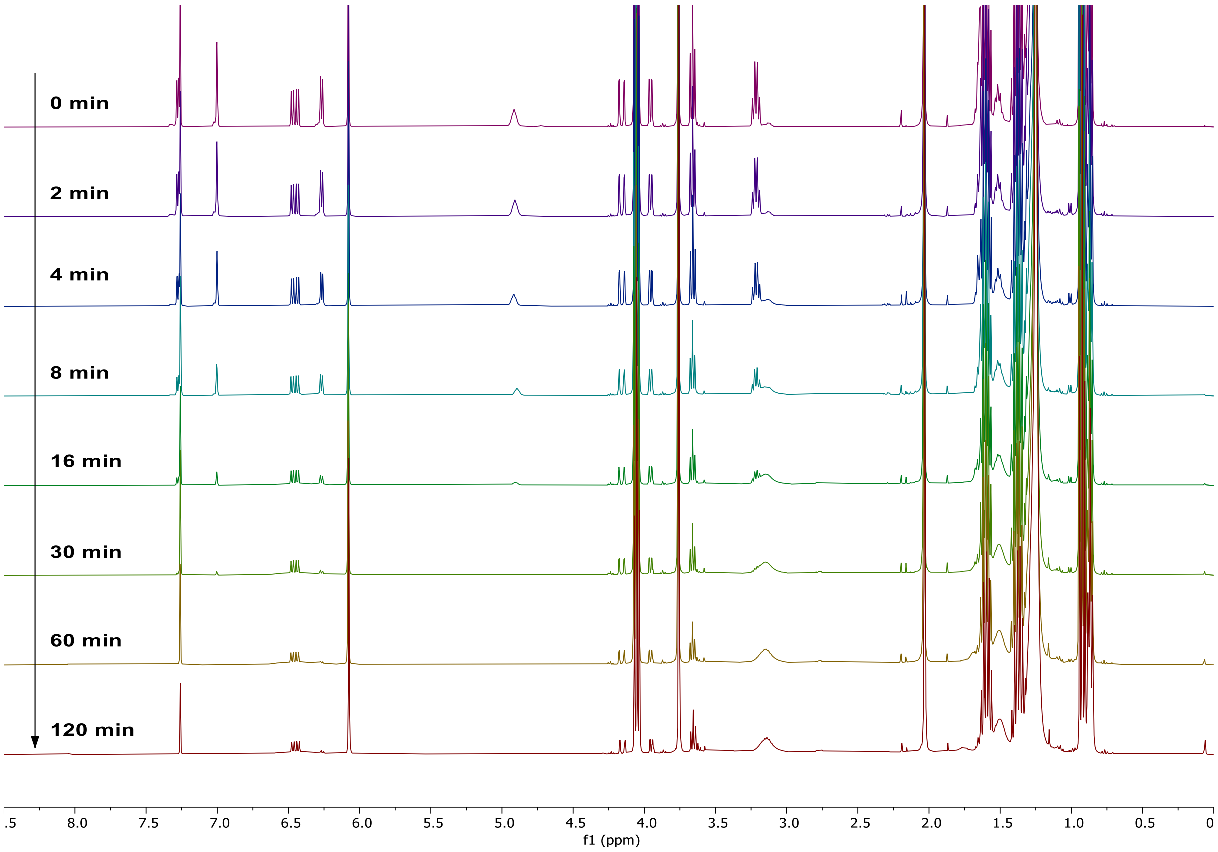

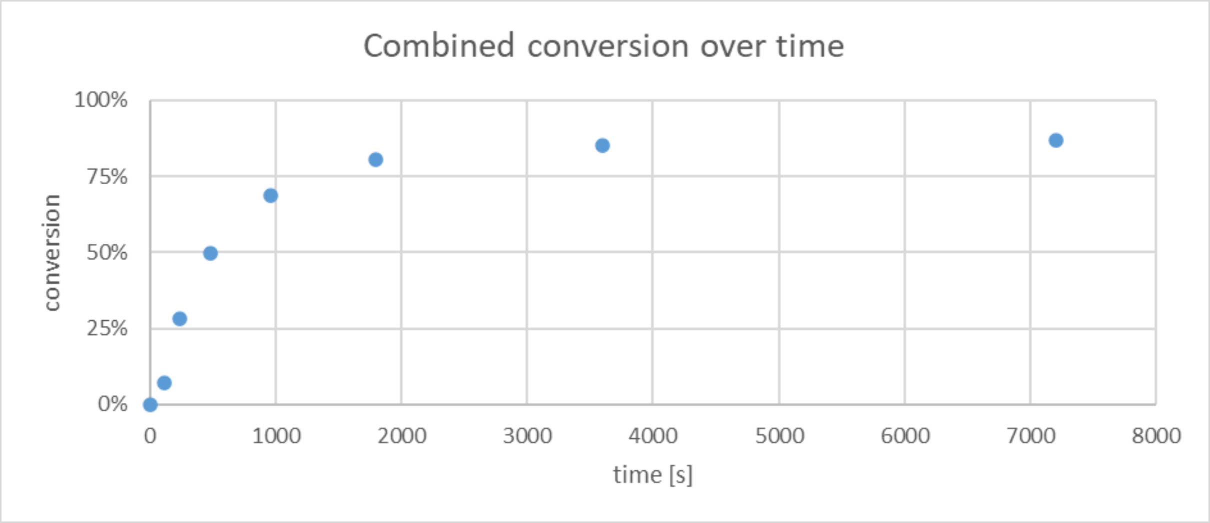
Combined conversion over time
conversion
100%
75%
50%
25%
0%
0
1000
2000
3000
4000
5000
6000
7000
8000
time [s]

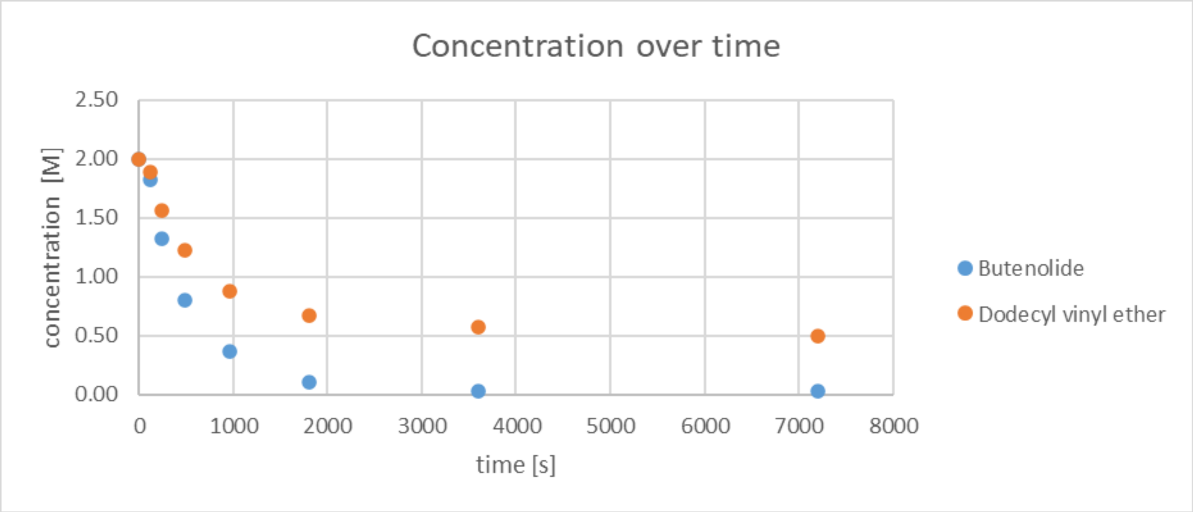
Concentration over time
concentration [M]
2.50
2.00
1.50
1.00
0.50
0.00
0
1000
2000
3000
4000
5000
6000
7000
8000
time [s]
Butenolide
Dodecyl vinyl ether

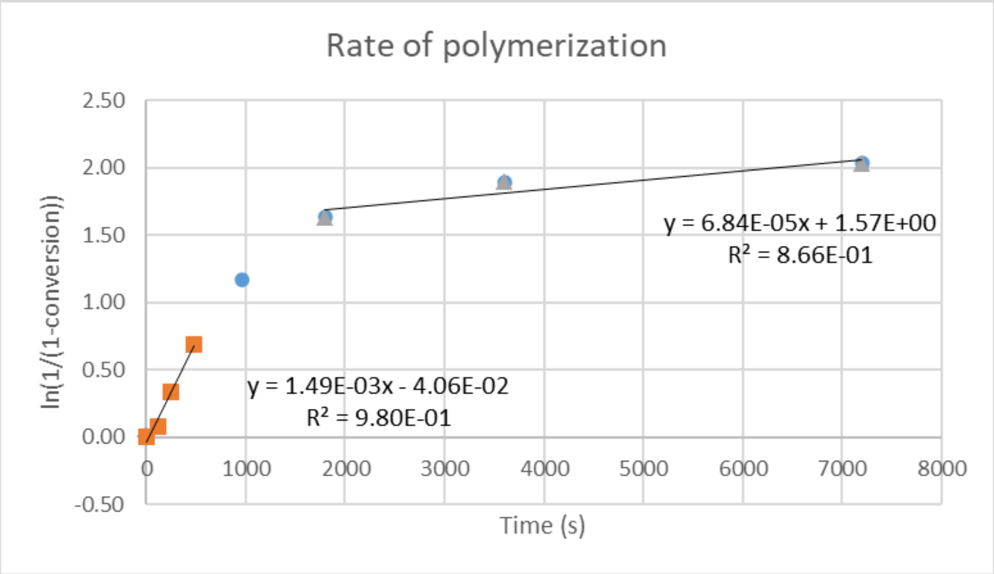
Rate of polymerization
ln(1/(1-conversion))
2.50
2.00
1.50
1.00
0.50
0.00
-0.50
0
1000
2000
3000
4000
5000
6000
7000
8000
Time (s)
y = 6.84E-05x + 1.57E+00
R² = 8.66E-01
y = 1.49E-03x - 4.06E-02
R² = 9.80E-01

Cyclohexyl carbamoxy butenolide (4b) and DVE in AcOBu

Poly(4b-co-DVE) in AcOBu					k1(obs)	1.38×10^{-3} [s^{-1}]
	4b		Dodecyl vinyl ether		k2(obs)	3.46×10^{-4} [s^{-1}]
Time [s]	Integral	Concentration [mol/L]	Integral	Concentration [mol/L]	Conversion	ln(1/[1-conversion])
0	0.89	2.00	0.96	2.00	0%	0.00
60	0.84	1.90	0.91	1.90	5%	0.05
120	0.80	1.81	0.88	1.84	9%	0.09
240	0.69	1.56	0.74	1.54	23%	0.26
480	0.47	1.05	0.54	1.13	45%	0.61
720	0.31	0.70	0.42	0.88	60%	0.93
1080	0.19	0.42	0.24	0.50	77%	1.47
1440	0.12	0.27	0.22	0.46	82%	1.69
2880	0.03	0.07	0.18	0.37	89%	2.20
3600	0.02	0.05	0.17	0.36	90%	2.26
5400	0.02	0.05	0.12	0.24	93%	2.63
7200	0.02	0.05	0.01	0.03	98%	3.84

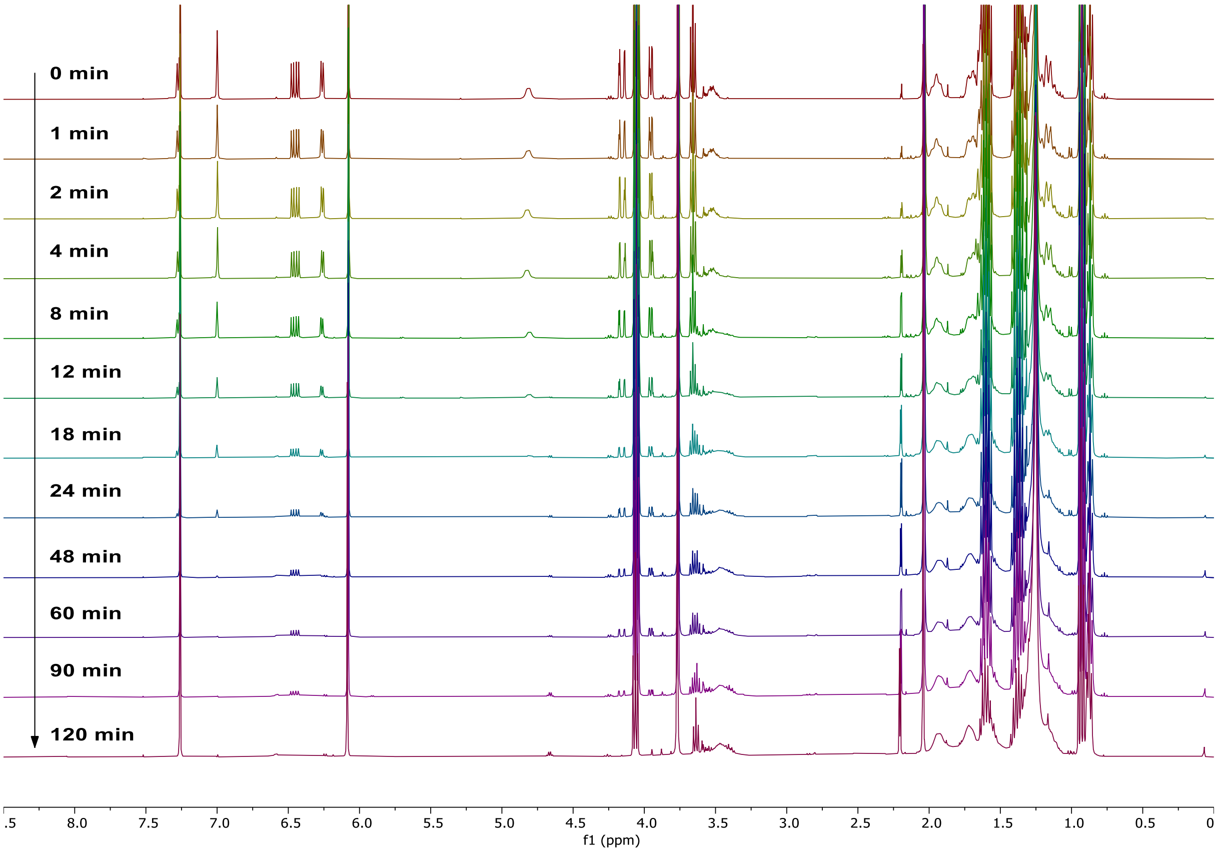

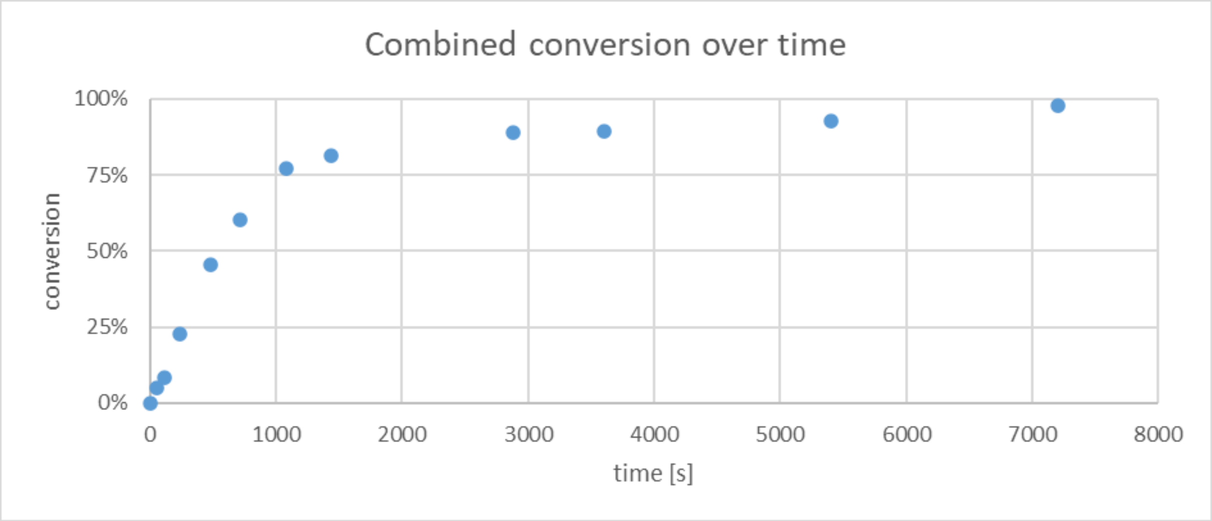
Combined conversion over time
conversion
100%
75%
50%
25%
0%
0
1000
2000
3000
4000
5000
6000
7000
8000
time [s]

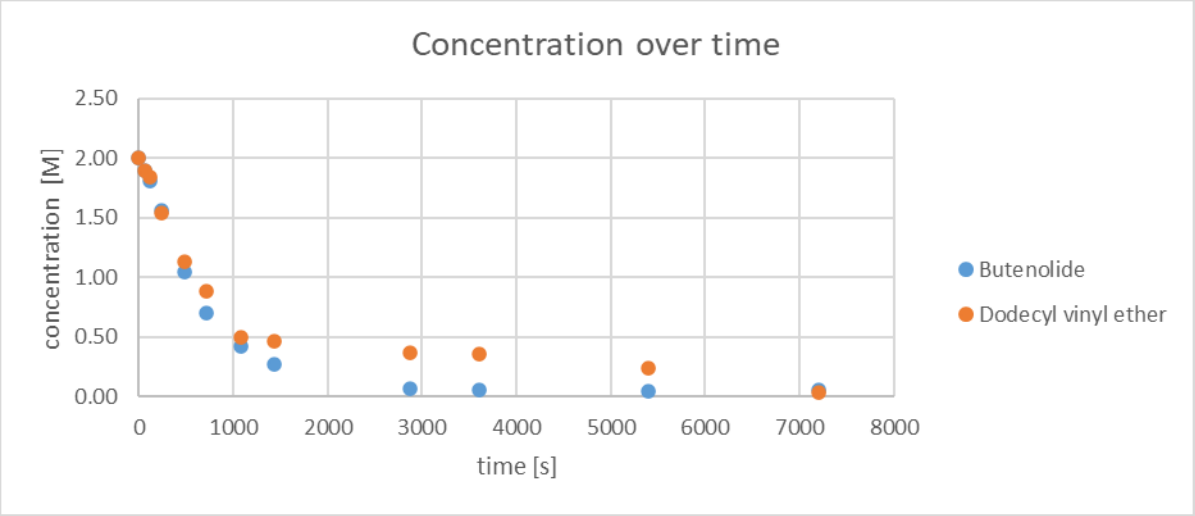
Concentration over time
concentration [M]
2.50
2.00
1.50
1.00
0.50
0.00
0
1000
2000
3000
4000
5000
6000
7000
8000
time [s]
Butenolide
Dodecyl vinyl ether

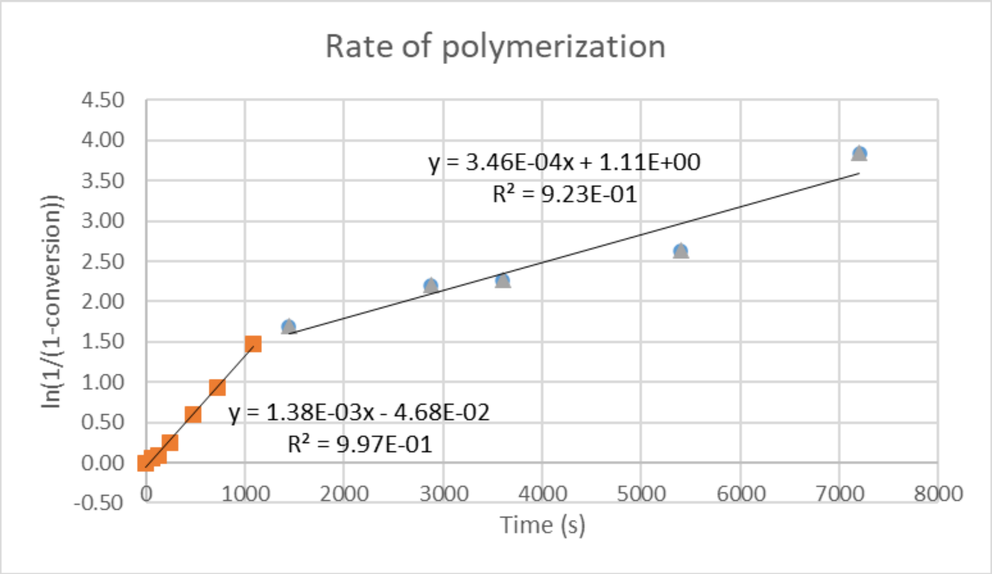
Rate of polymerization
ln(1/(1-conversion))
4.50
4.00
3.50
3.00
2.50
2.00
1.50
1.00
0.50
0.00
-0.50
0
1000
2000
3000
4000
5000
6000
7000
8000
Time (s)
y = 3.46E-04x + 1.11E+00
R² = 9.23E-01
y = 1.38E-03x - 4.68E-02
R² = 9.97E-01

Homopolymerization of DVE in AcOBu

Poly(DVE) in AcOBu			k1(obs)	1.64 x 10^{-4} [s^{-1}]
	DVE			
Time [s]	Integral	Concentration [mol/L]	Conversion	ln(1/[1-conversion])
0	1.88	4.00	0%	0.00
120	1.89	4.01	0%	0.00
240	1.86	3.96	1%	0.01
480	1.80	3.82	4%	0.05
960	1.64	3.48	13%	0.14
1800	1.44	3.07	23%	0.27
3600	1.25	2.65	34%	0.41
7200	1.14	2.41	40%	0.51

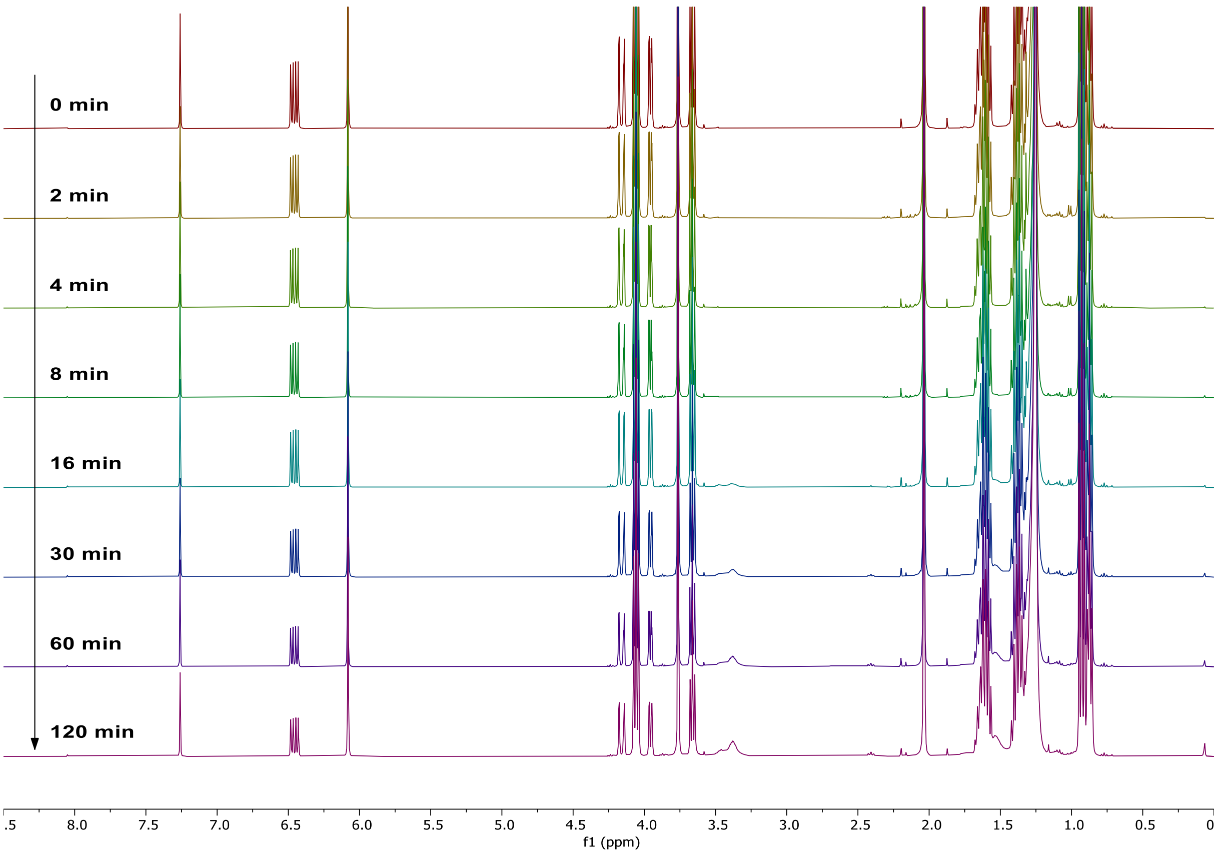

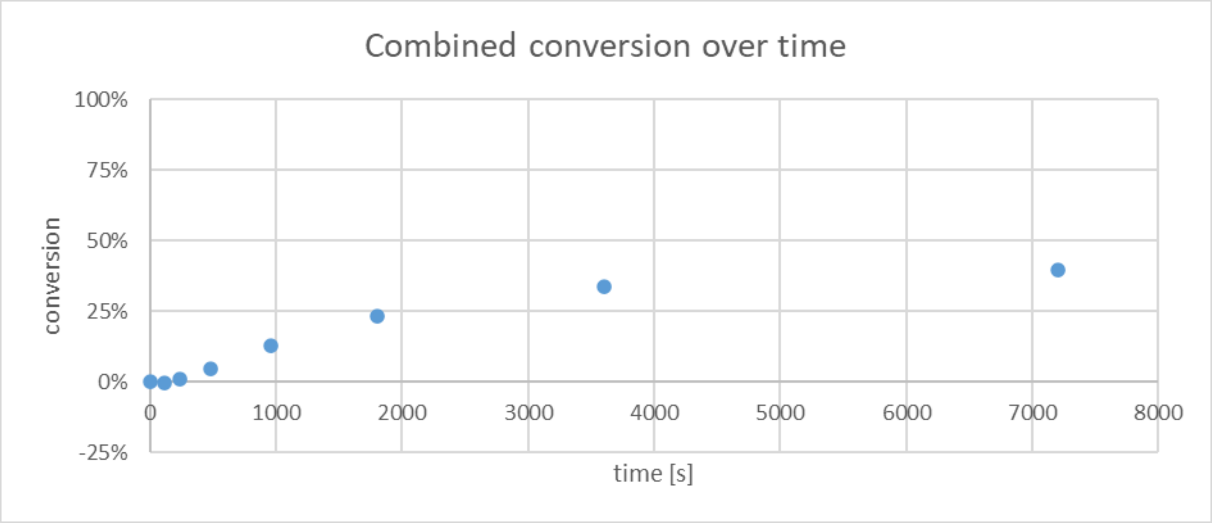
Combined conversion over time
conversion
100%
75%
50%
25%
0%
-25%
0
1000
2000
3000
4000
5000
6000
7000
8000
time [s]

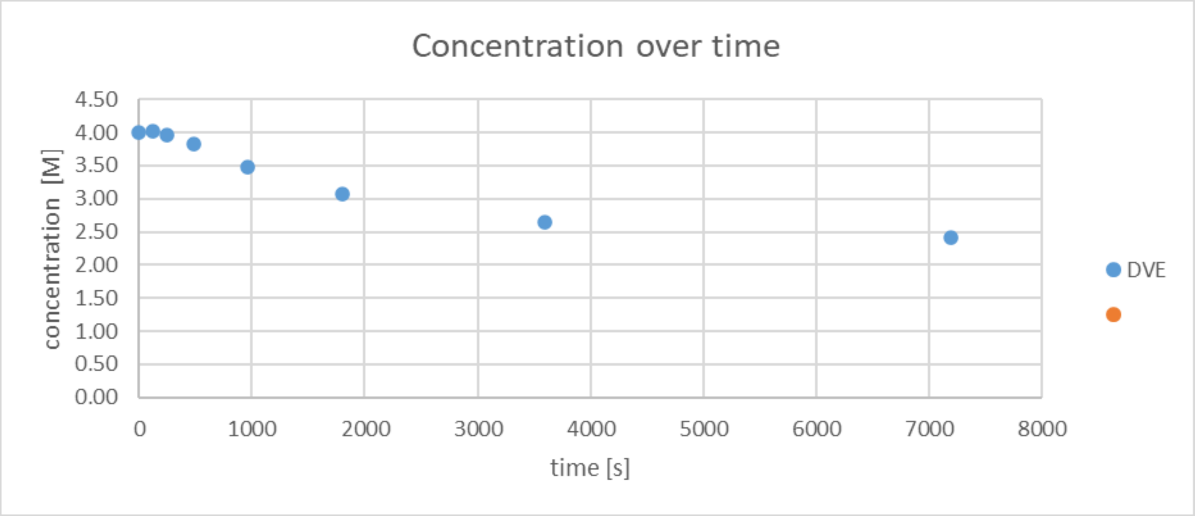
Concentration over time
concentration [M]
4.50
4.00
3.50
3.00
2.50
2.00
1.50
1.00
0.50
0.00
0
1000
2000
3000
4000
5000
6000
7000
8000
time [s]
DVE

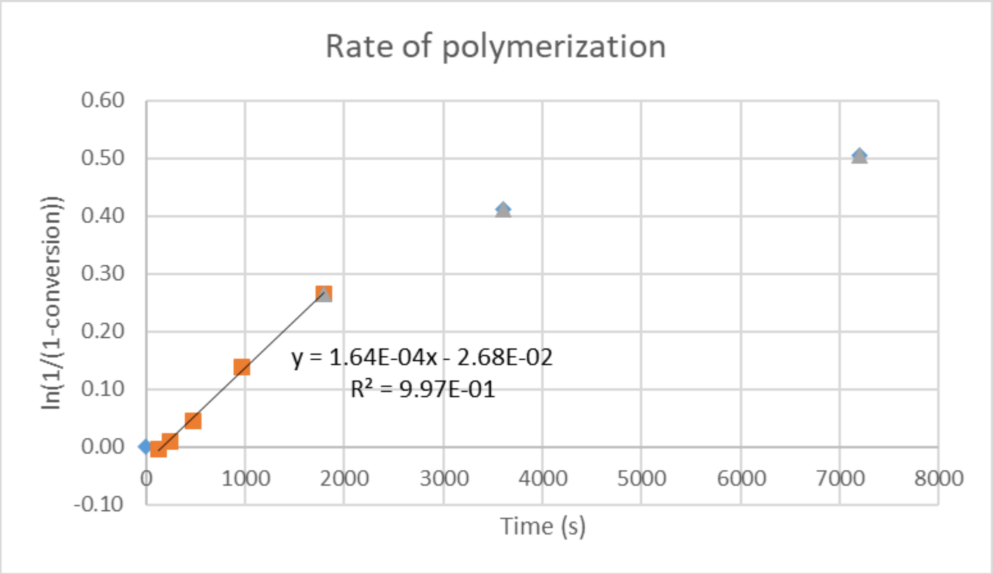
Rate of polymerization
ln(1/(1-conversion))
0.60
0.50
0.40
0.30
0.20
0.10
0.00
-0.10
0
1000
2000
3000
4000
5000
6000
7000
8000
Time (s)
y = 1.64E-04x - 2.68E-02
R² = 9.97E-01

Homopolymerization of NVP in NMP

Poly(NVP)			k1(obs)	6.29×10^{-3} [s^{-1}]
	NVP			
Time [s]	Integral	Concentration [mol/L]	Conversion	ln(1/[1-conversion])
0	1.93	4.00	0%	0.00
60	1.28	2.66	33%	0.41
180	0.56	1.16	71%	1.23
360	0.21	0.44	89%	2.21
600	0.12	0.25	94%	2.78
1200	0.03	0.07	98%	4.01
2400	0.03	0.06	99%	4.21
3600	0.04	0.07	98%	3.98

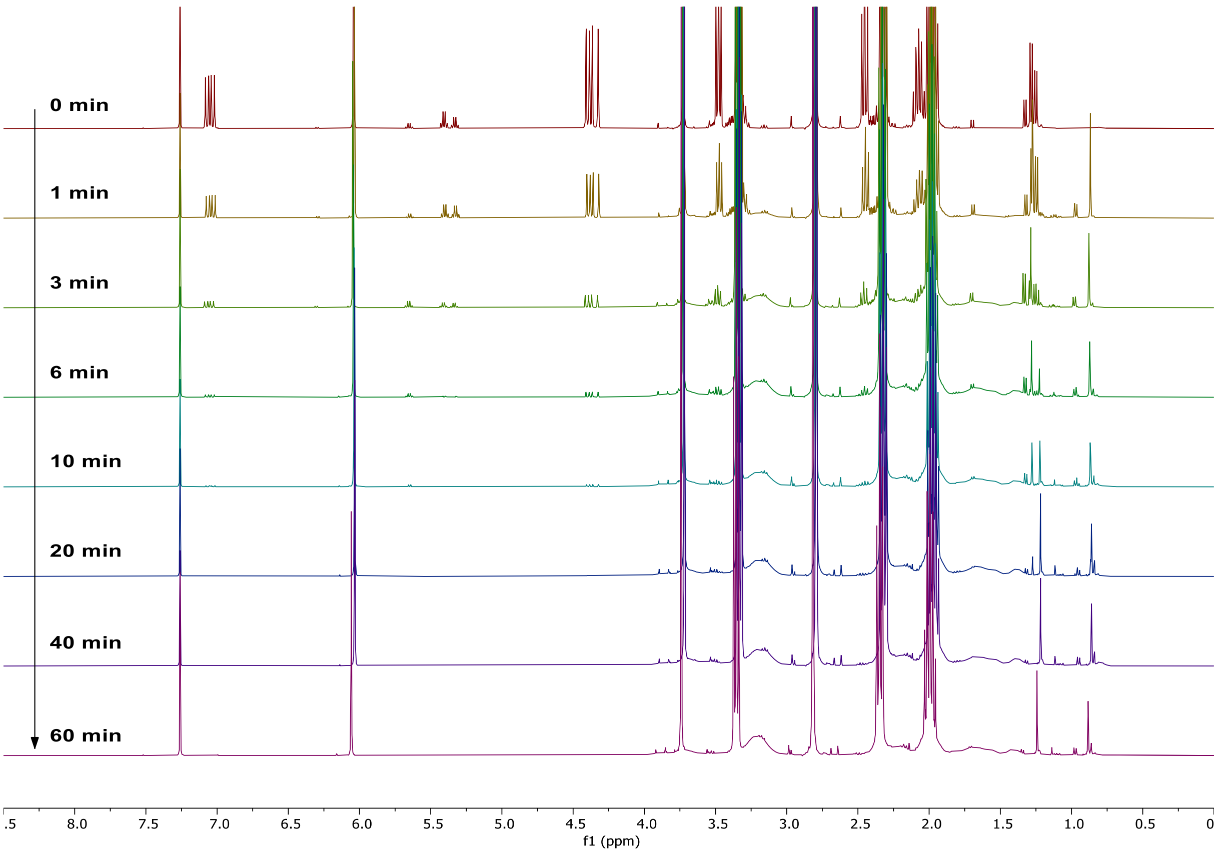

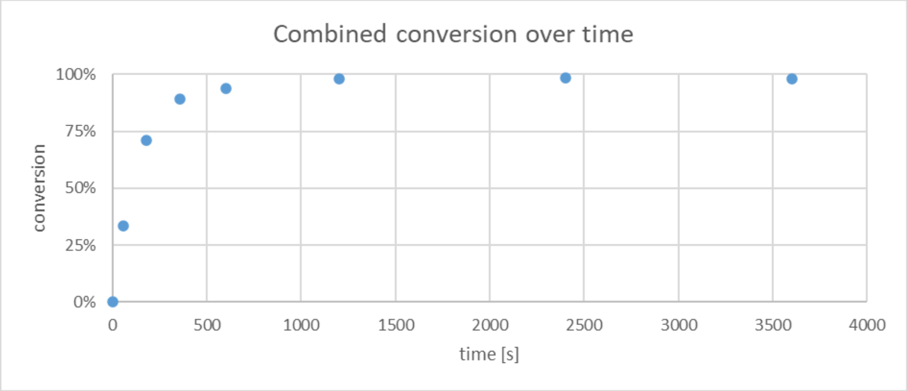
Combined conversion over time
conversion
100%
75%
50%
25%
0%
0
500
1000
1500
2000
2500
3000
3500
4000
time [s]

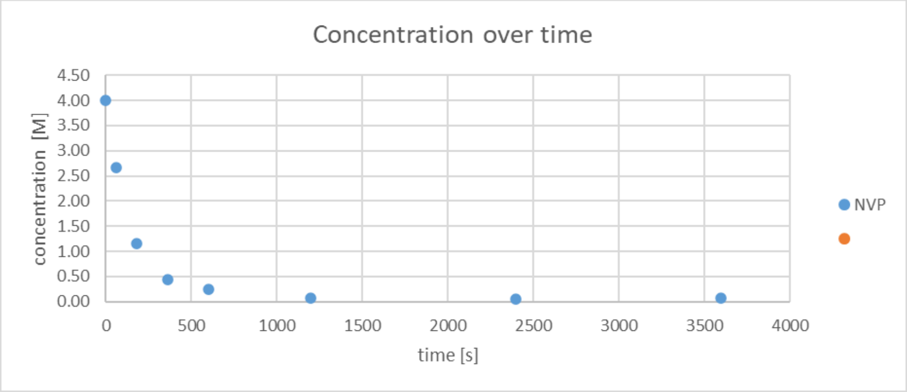
Concentration over time
concentration [M]
4.50
4.00
3.50
3.00
2.50
2.00
1.50
1.00
0.50
0.00
0
500
1000
1500
2000
2500
3000
3500
4000
time [s]
NVP

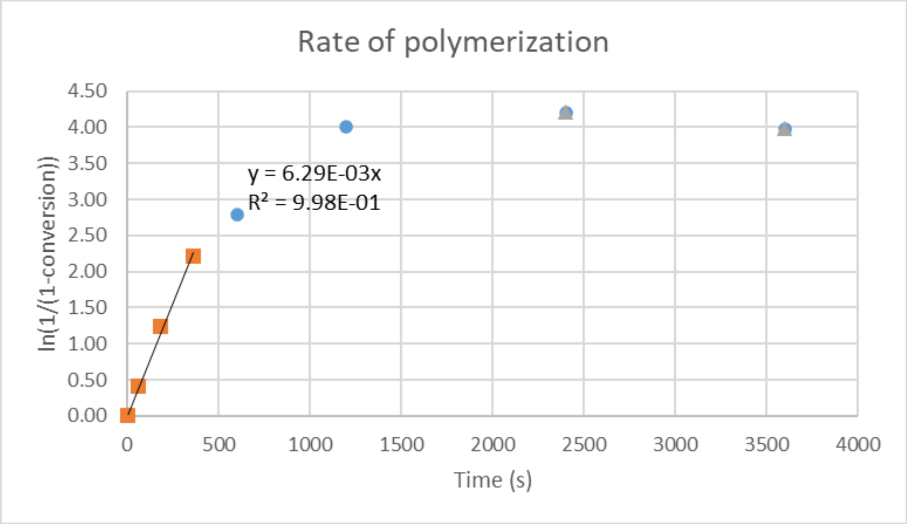
Rate of polymerization
ln(1/(1-conversion))
4.50
4.00
3.50
3.00
2.50
2.00
1.50
1.00
0.50
0.00
0
500
1000
1500
2000
2500
3000
3500
4000
Time (s)
y = 6.29E-03x
R² = 9.98E-01

Methoxy butenolide (1a) and DVE (1:3) in AcOBu

Poly(1a-co-DVE) 1-3 in AcOBu					k1(obs)	6.87×10^{-4} [s^{-1}]
	1a		Dodecyl vinyl ether		k2(obs)	4.59×10^{-5} [s^{-1}]
Time [s]	Integral	Concentration [mol/L]	Integral	Concentration [mol/L]	Conversion	ln(1/[1-conversion])
0	0.47	1.00	1.41	3.00	0%	0.00
120	0.42	0.89	1.37	2.92	5%	0.05
240	0.34	0.73	1.31	2.78	12%	0.13
480	0.16	0.34	1.15	2.45	30%	0.36
960	0.02	0.04	0.98	2.09	47%	0.63
1800	0.01	0.03	0.86	1.82	54%	0.77
7200	0.01	0.03	0.67	1.42	64%	1.02

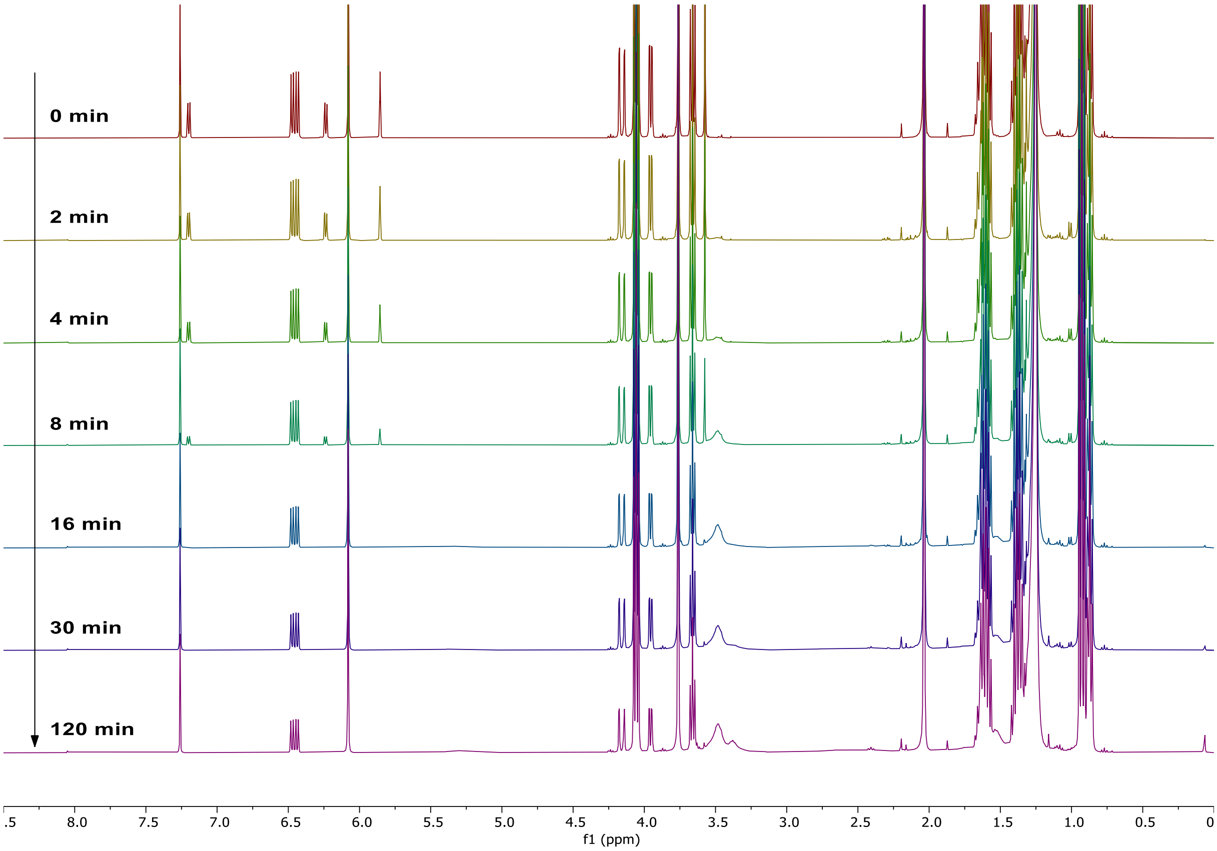

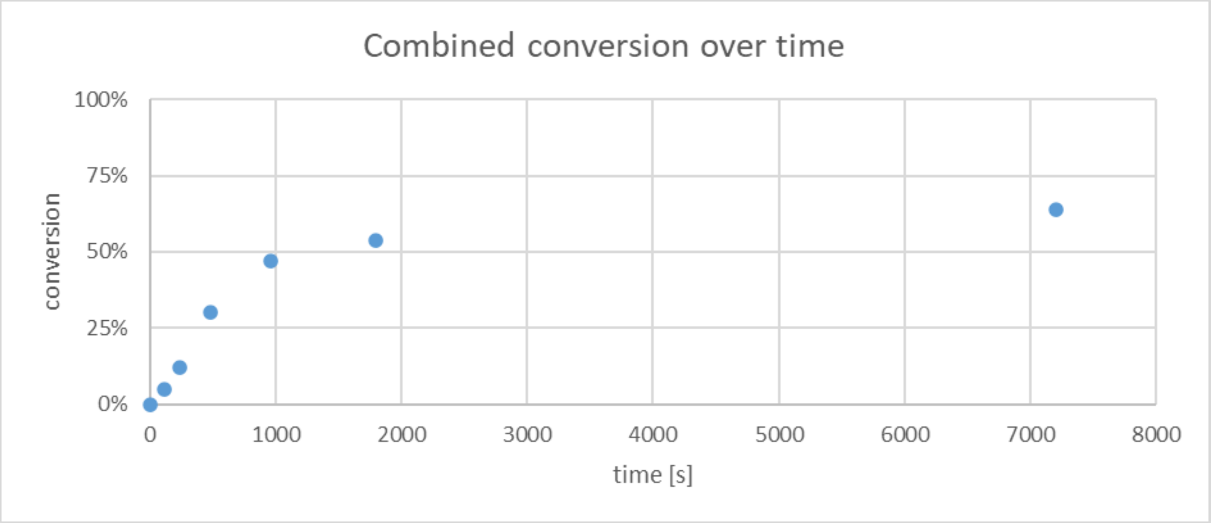

Combined conversion over time
100%
75%
50%
25%
0%
conversion
0
1000
2000
3000
4000
5000
6000
7000
8000
time [s]

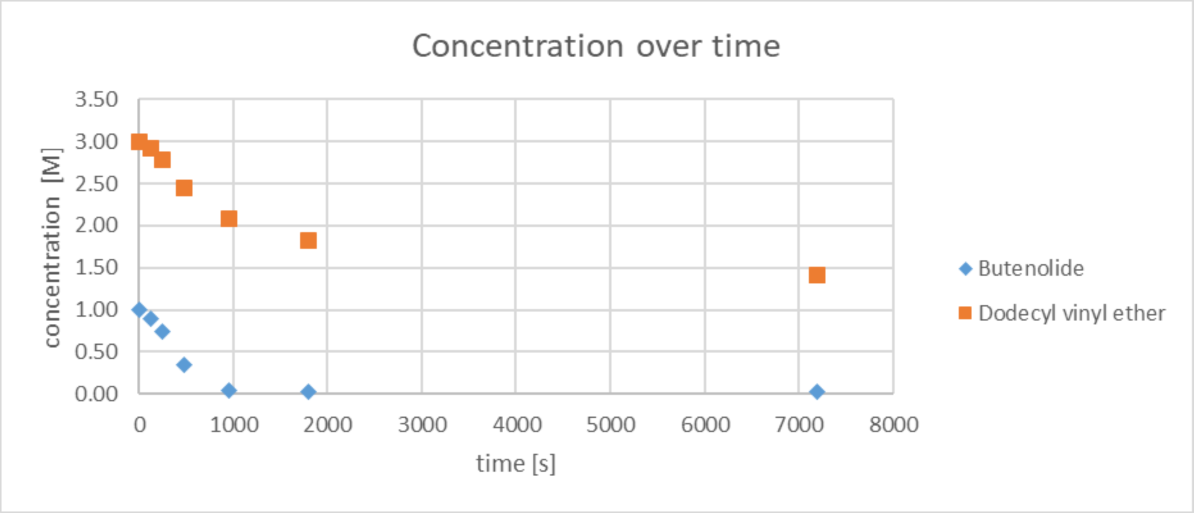

Concentration over time
3.50
3.00
2.50
2.00
1.50
1.00
0.50
0.00
concentration [M]
0
1000
2000
3000
4000
5000
6000
7000
8000
time [s]
Butenolide
Dodecyl vinyl ether

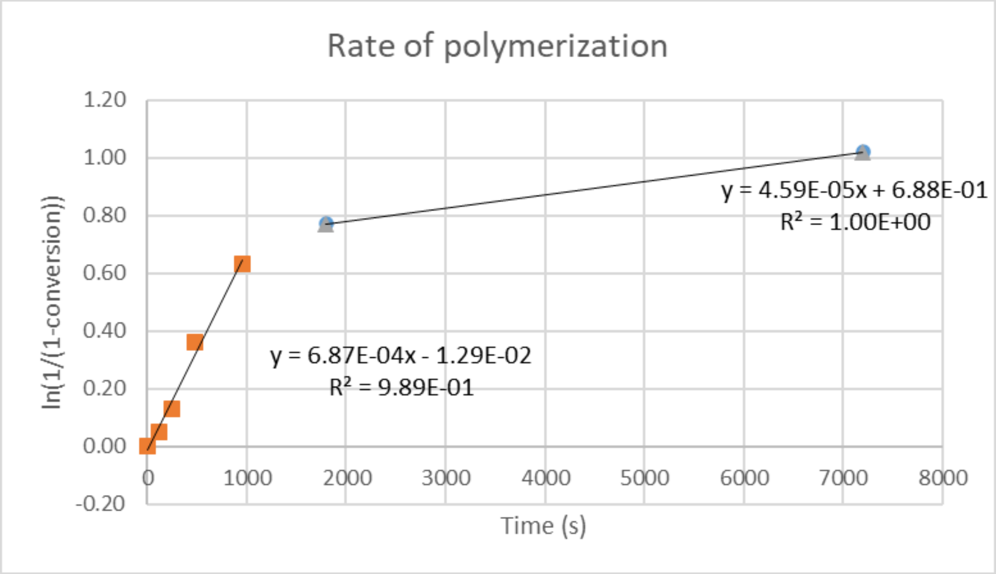

Rate of polymerization
1.20
1.00
0.80
0.60
0.40
0.20
0.00
-0.20
ln(1/(1-conversion))
0
1000
2000
3000
4000
5000
6000
7000
8000
Time (s)
y = 4.59E-05x + 6.88E-01
R² = 1.00E+00
y = 6.87E-04x - 1.29E-02
R² = 9.89E-01

Methoxy butenolide (1a) and DVE (3:1) in AcOBu

Poly(1a-co-DVE) 3-1 in AcOBu					k1(obs)	4.44 x 10^{-4} [s^{-1}]
	1a		Dodecyl vinyl ether		k2(obs)	n/a
Time [s]	Integral	Concentration [mol/L]	Integral	Concentration [mol/L]	Conversion	ln(1/[1-conversion])
0	1.42	3.00	0.47	1.00	0%	0.00
120	1.40	2.96	0.45	0.95	2%	0.02
240	1.37	2.90	0.42	0.90	5%	0.05
480	1.25	2.64	0.36	0.76	15%	0.16
960	1.01	2.14	0.24	0.50	34%	0.42
1800	0.77	1.63	0.12	0.26	53%	0.75
7200	0.44	0.93	0.00	0.00	77%	1.46

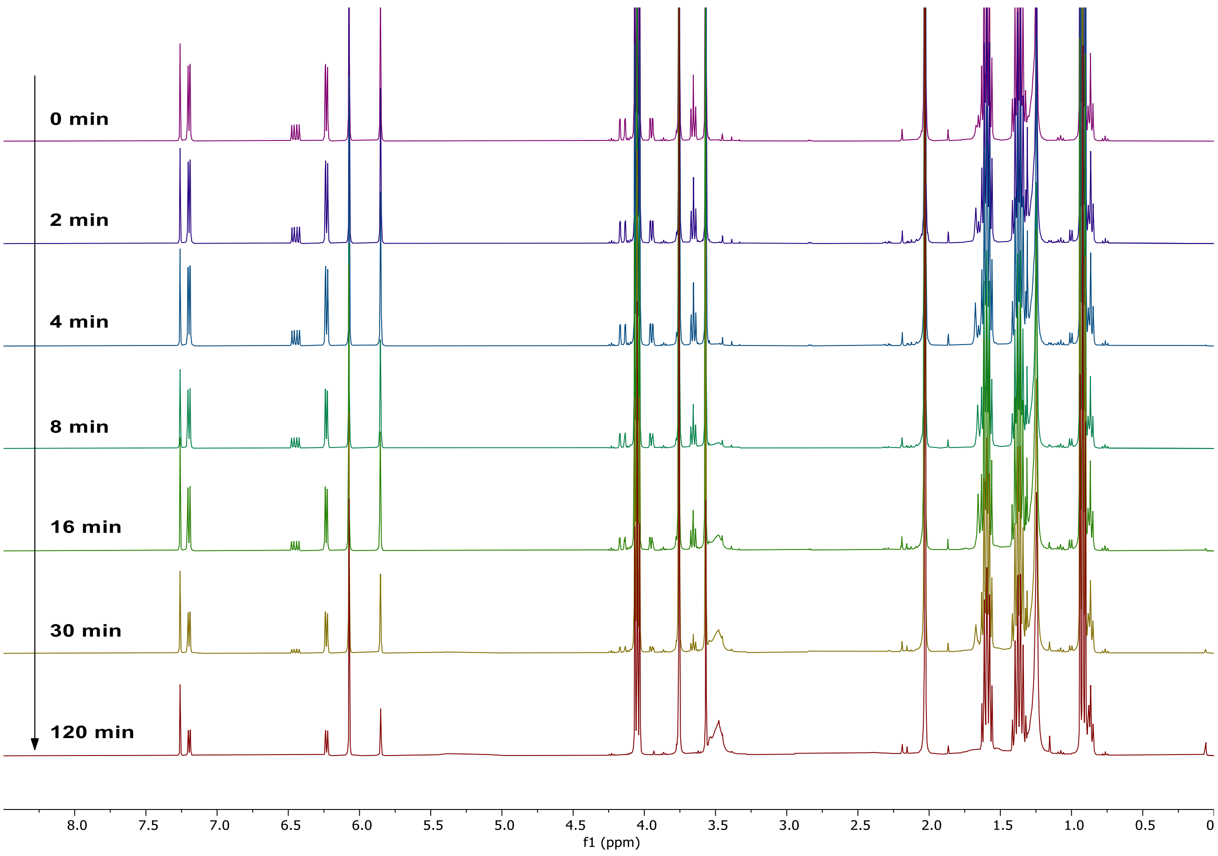

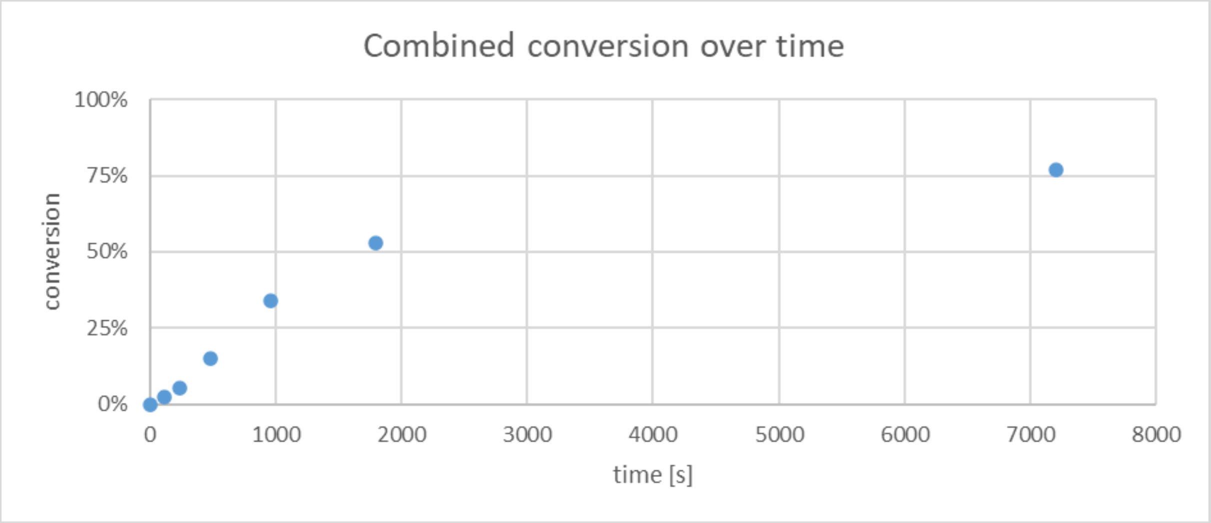
Combined conversion over time
conversion
100%
75%
50%
25%
0%
0
1000
2000
3000
4000
5000
6000
7000
8000
time [s]

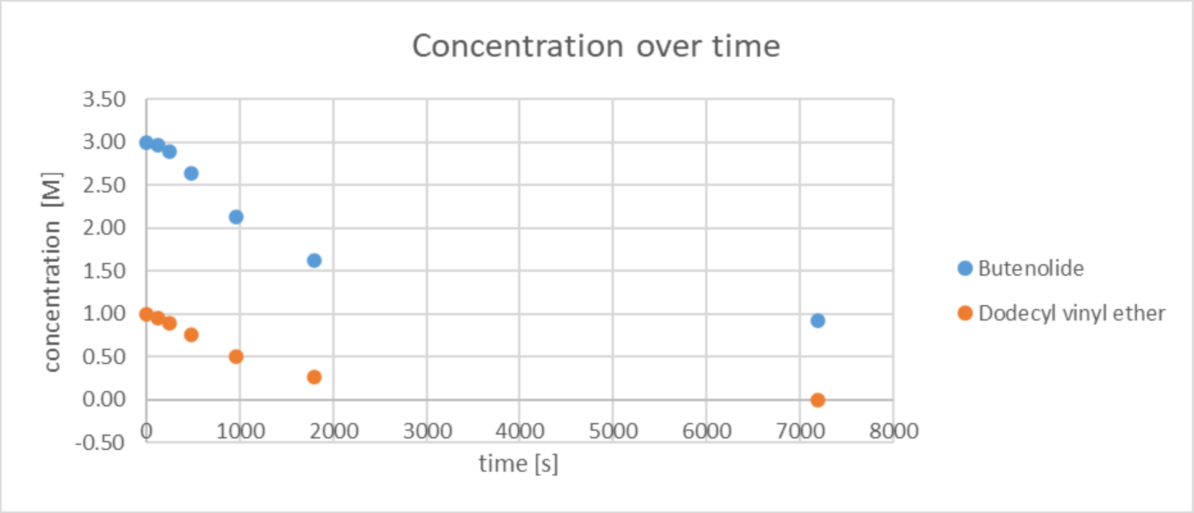
Concentration over time
concentration [M]
3.50
3.00
2.50
2.00
1.50
1.00
0.50
0.00
-0.50
0
1000
2000
3000
4000
5000
6000
7000
8000
time [s]
Butenolide
Dodecyl vinyl ether

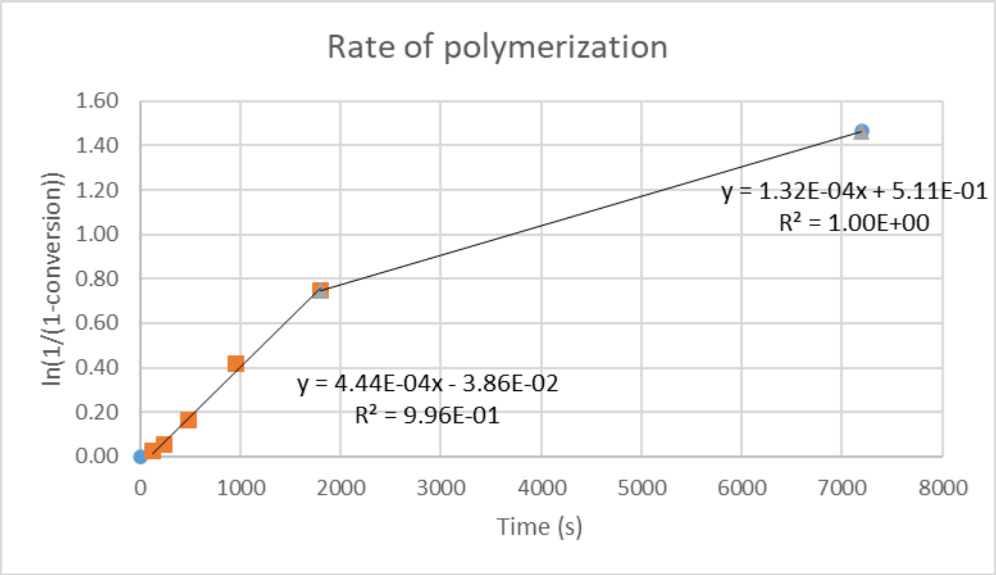
Rate of polymerization
ln(1/(1-conversion))
1.60
1.40
1.20
1.00
0.80
0.60
0.40
0.20
0.00
0
1000
2000
3000
4000
5000
6000
7000
8000
Time (s)
y = 1.32E-04x + 5.11E-01
R² = 1.00E+00
y = 4.44E-04x - 3.86E-02
R² = 9.96E-01

Methoxy butenolide (1a) in AcOBu

Poly(1a) in AcOBu			k1(obs)	4.55 x 10^{-5} [s^{-1}]
	1a		k2(obs)	n/a
Time [s]	Integral	Concentration [mol/L]	Conversion	ln(1/[1-conversion])
0	1.83	4.00	0%	0.00
120	1.83	3.99	0%	0.00
240	1.83	4.00	0%	0.00
480	1.82	3.98	0%	0.00
960	1.79	3.91	2%	0.02
1800	1.71	3.74	7%	0.07
7200	1.51	3.31	17%	0.19

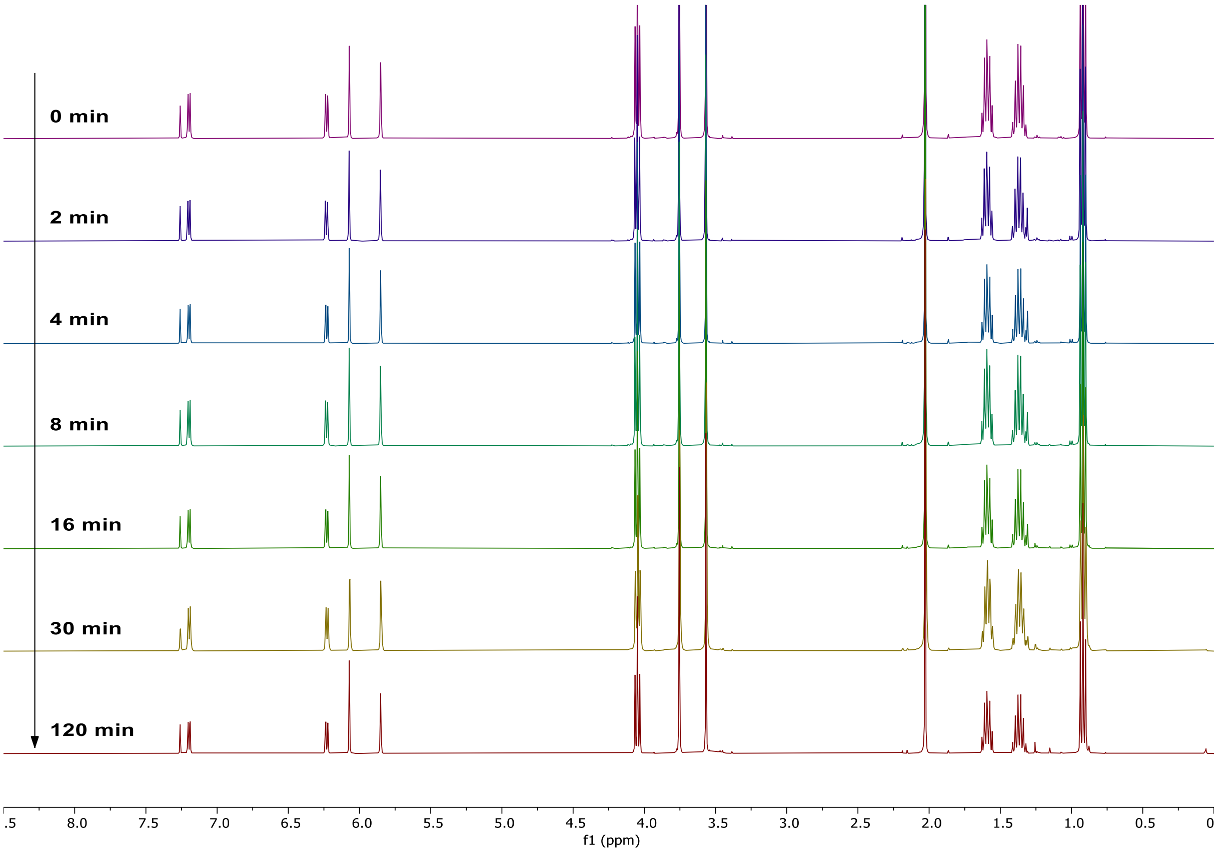

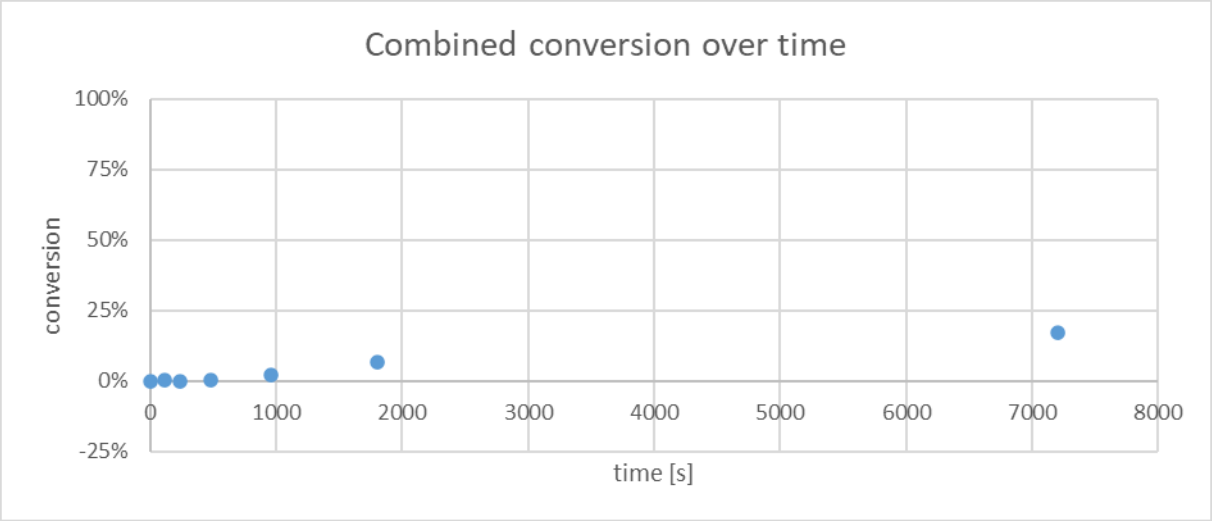
Combined conversion over time
conversion
100%
75%
50%
25%
0%
-25%
0
1000
2000
3000
4000
5000
6000
7000
8000
time [s]

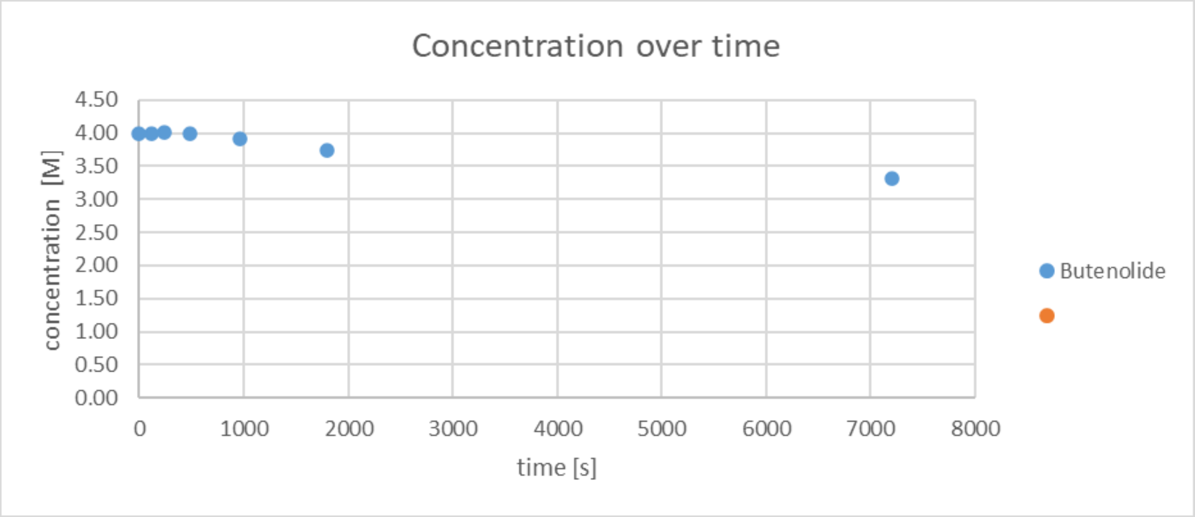
Concentration over time
concentration [M]
4.50
4.00
3.50
3.00
2.50
2.00
1.50
1.00
0.50
0.00
0
1000
2000
3000
4000
5000
6000
7000
8000
time [s]
Butenolide

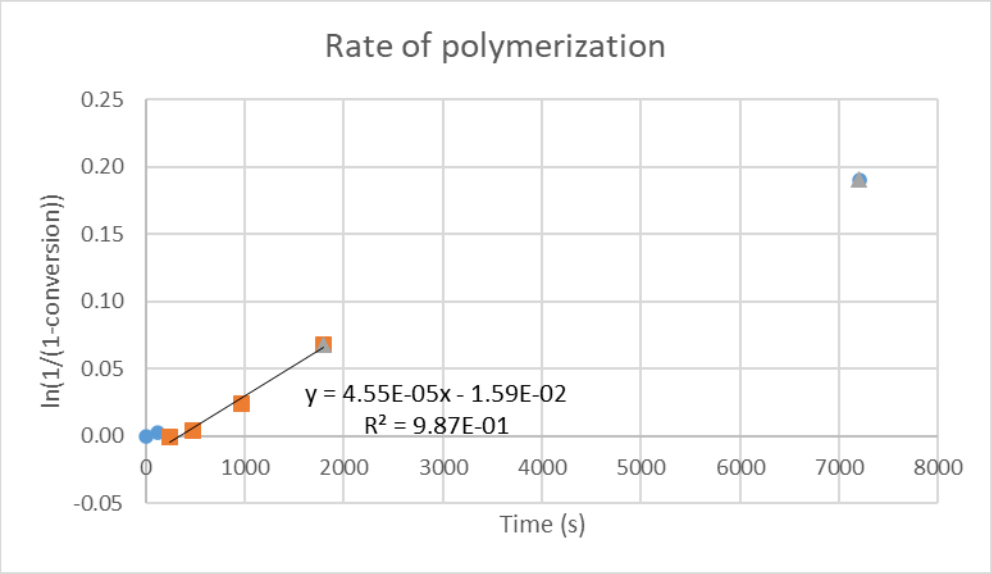
Rate of polymerization
ln(1/(1-conversion))
0.25
0.20
0.15
0.10
0.05
0.00
-0.05
0
1000
2000
3000
4000
5000
6000
7000
8000
Time (s)
y = 4.55E-05x - 1.59E-02
R² = 9.87E-01

Methoxy butenolide (1a) and NVP (1:3) in NMP

Poly(1a-co-NVP) 1:3 in NMP					k1(obs)	7.51×10^{-3} [s^{-1}]
	1a		N-vinyl pyrrolidone		k2(obs)	3.29×10^{-3} [s^{-1}]
Time [s]	Integral	Concentration [mol/L]	Integral	Concentration [mol/L]	Conversion	ln(1/[1-conversion])
0	0.45	1.00	1.44	3.00	0%	0.00
120	0.05	0.12	0.72	1.51	59%	0.90
240	0.00	0.01	0.43	0.89	78%	1.49
480	0.00	0.01	0.20	0.42	89%	2.23
960	0.00	0.01	0.04	0.09	98%	3.74
1800	0.00	0.00	0.02	0.04	99%	4.58
7200	0.00	0.01	0.01	0.03	99%	4.72

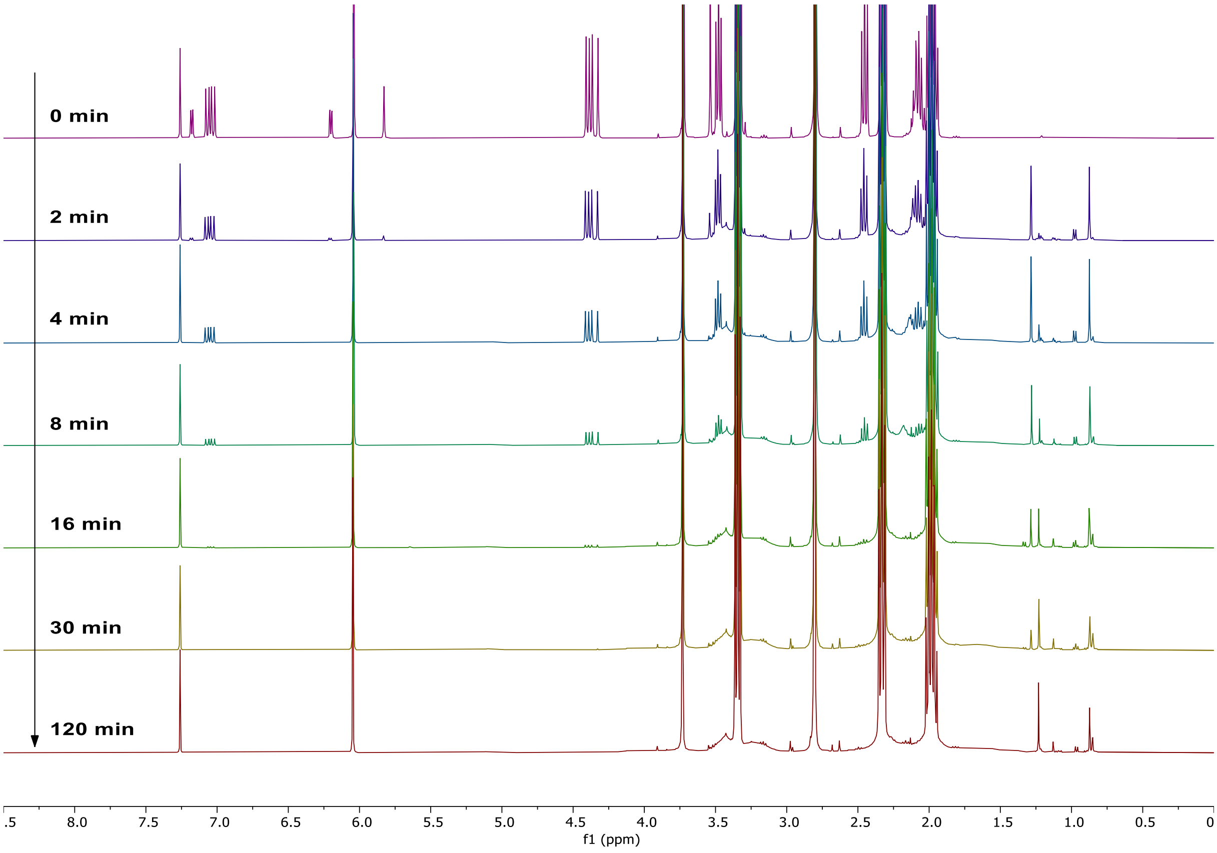

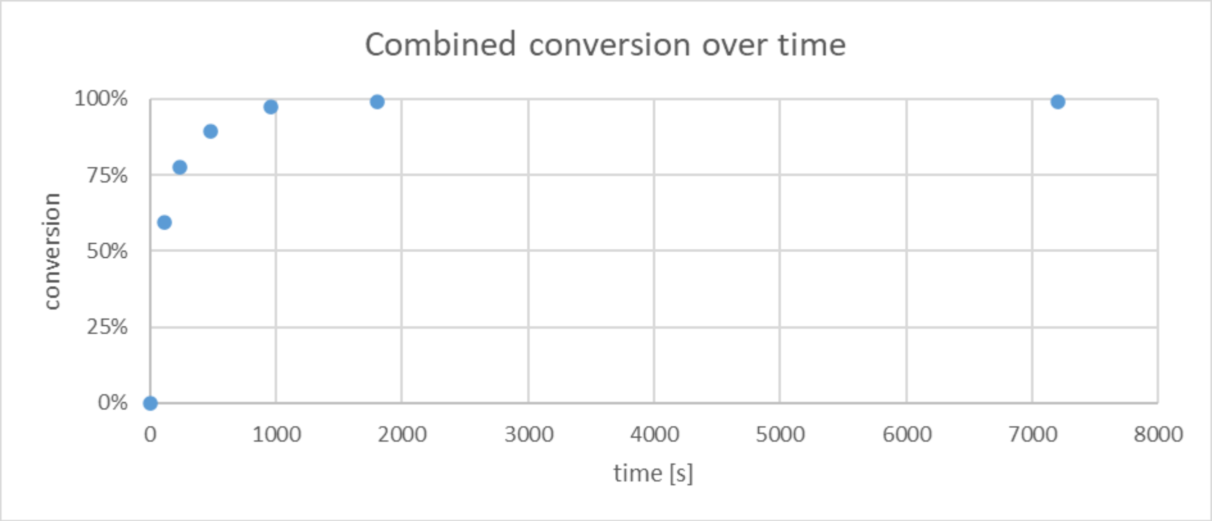
Combined conversion over time
100%
75%
50%
25%
0%
conversion
0
1000
2000
3000
4000
5000
6000
7000
8000
time [s]

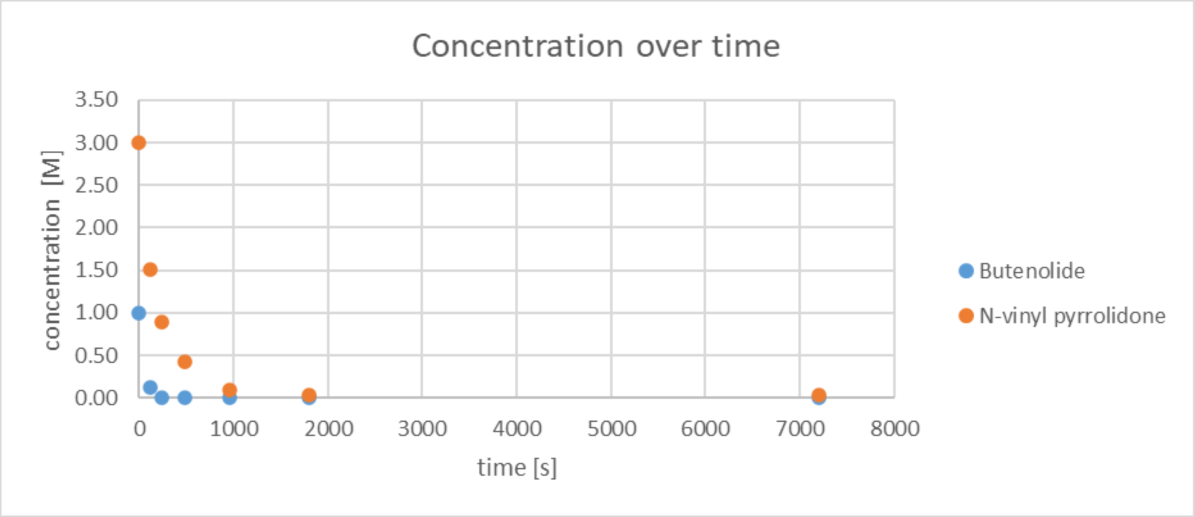
Concentration over time
3.50
3.00
2.50
2.00
1.50
1.00
0.50
0.00
concentration [M]
0
1000
2000
3000
4000
5000
6000
7000
8000
time [s]
Butenolide
N-vinyl pyrrolidone

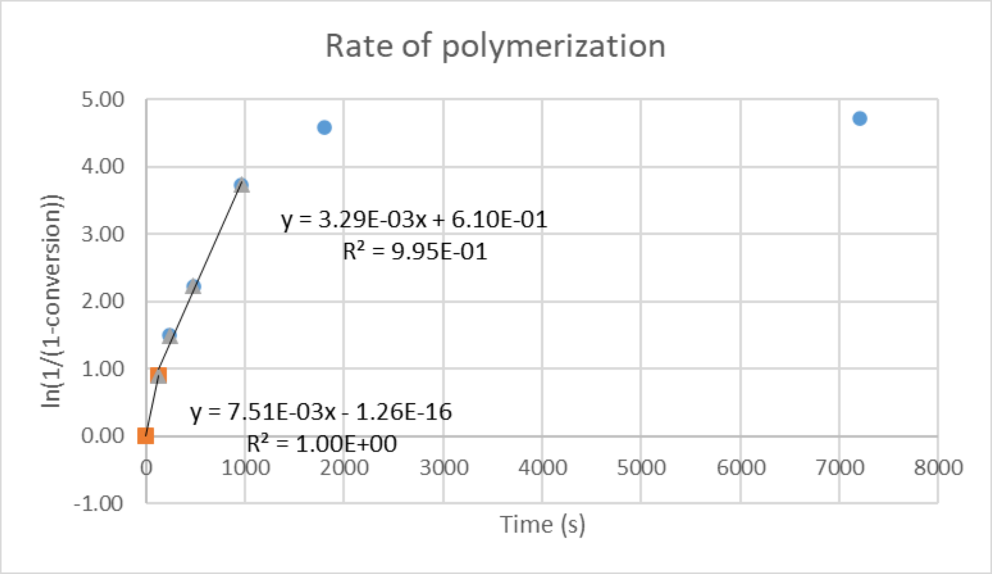
Rate of polymerization
5.00
4.00
3.00
2.00
1.00
0.00
-1.00
ln(1/(1-conversion))
y = 3.29E-03x + 6.10E-01
R² = 9.95E-01
y = 7.51E-03x - 1.26E-16
R² = 1.00E+00
0
1000
2000
3000
4000
5000
6000
7000
8000
Time (s)

Methoxy butenolide (1a) and NVP (1:1) in NMP

Poly(1a-co-NVP) in NMP					k1(obs)	7.24 x 10^{-3} [s^{-1}]
	1a		N-vinyl pyrrolidone		k2(obs)	1.68 x 10^{-3} [s^{-1}]
Time [s]	Integral	Concentration [mol/L]	Integral	Concentration [mol/L]	Conversion	ln(1/[1-conversion])
0	0.89	2.00	0.90	2.00	0%	0.00
60	0.65	1.46	0.49	1.09	36%	0.45
120	0.43	0.97	0.29	0.66	59%	0.90
240	0.20	0.45	0.11	0.25	82%	1.74
480	0.07	0.16	0.04	0.10	94%	2.76
960	0.01	0.03	0.02	0.04	98%	4.02
1800	0.00	0.01	0.01	0.02	99%	5.06
7200	0.00	0.01	0.01	0.03	99%	4.72

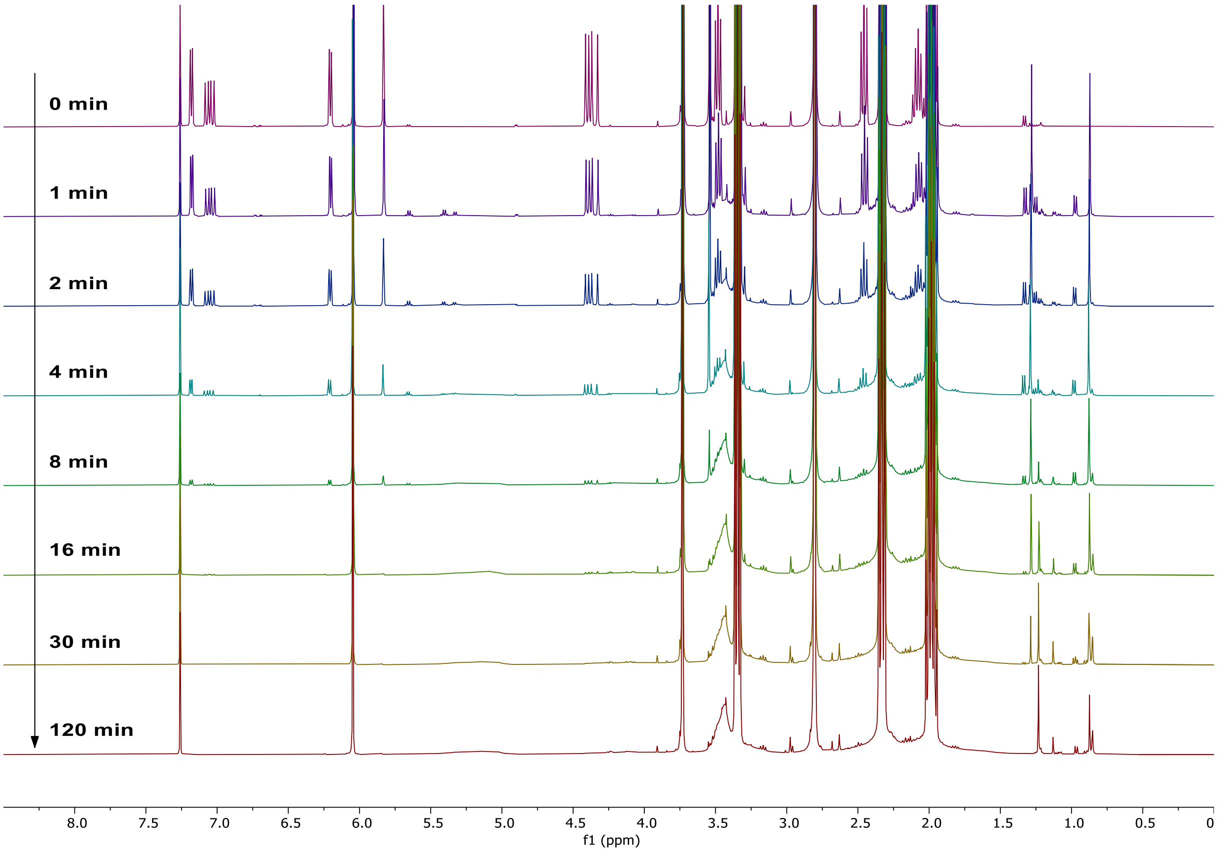

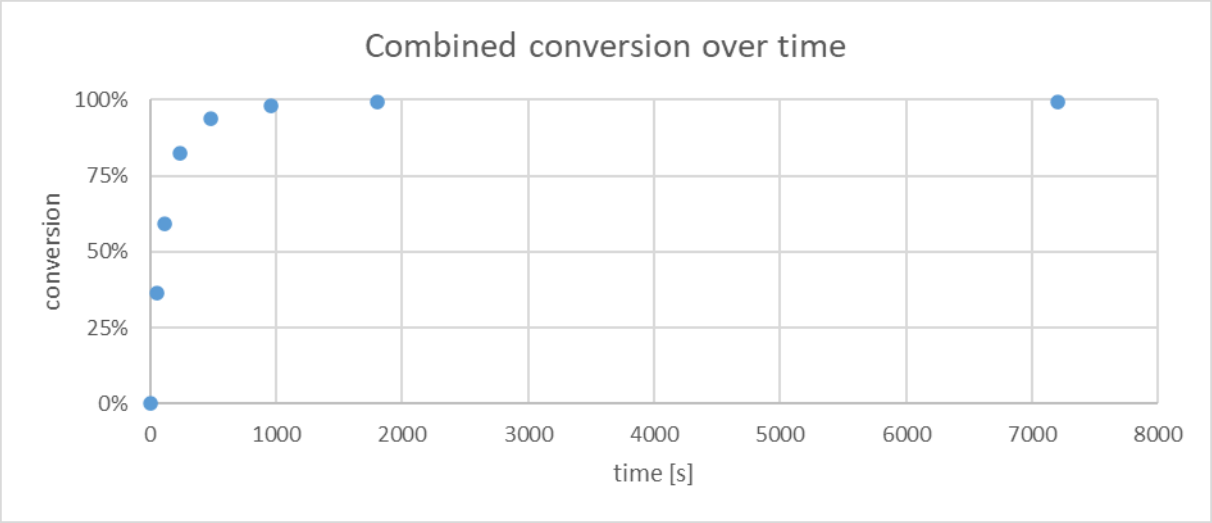
Combined conversion over time
conversion
100%
75%
50%
25%
0%
0
1000
2000
3000
4000
5000
6000
7000
8000
time [s]

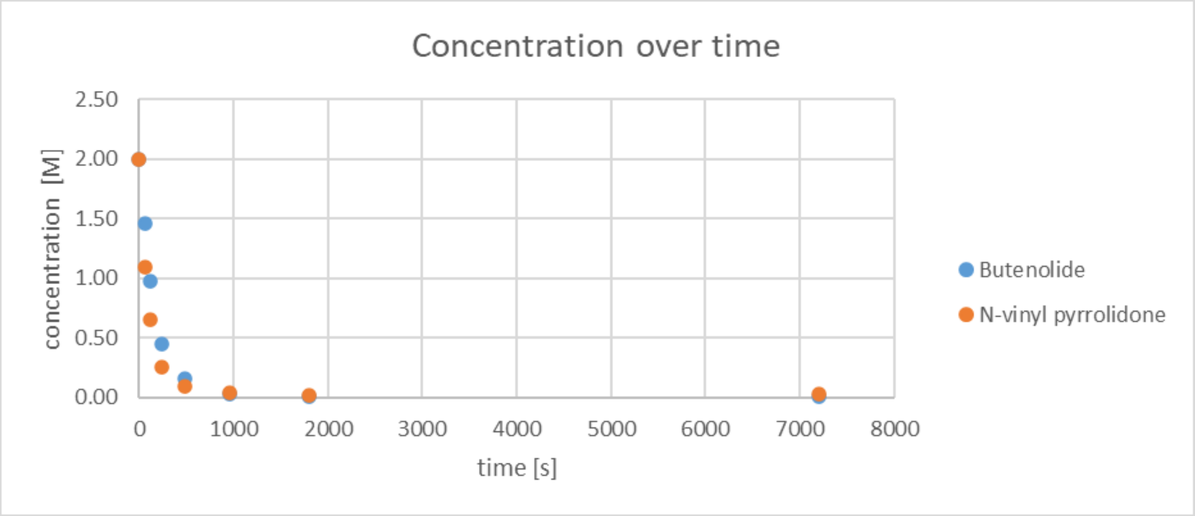
Concentration over time
concentration [M]
2.50
2.00
1.50
1.00
0.50
0.00
0
1000
2000
3000
4000
5000
6000
7000
8000
time [s]
Butenolide
N-vinyl pyrrolidone

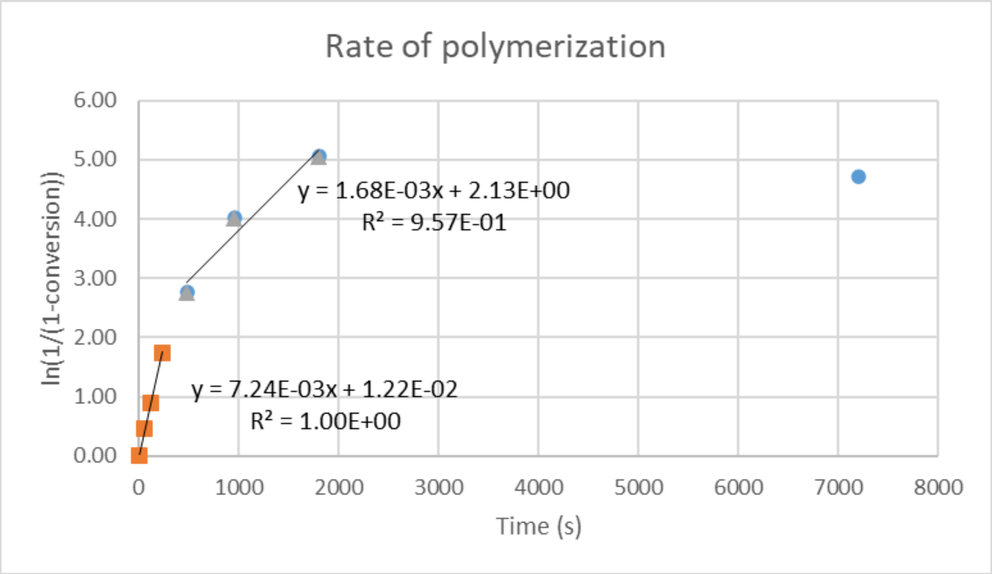
Rate of polymerization
ln(1/(1-conversion))
6.00
5.00
4.00
3.00
2.00
1.00
0.00
y = 1.68E-03x + 2.13E+00
R² = 9.57E-01
y = 7.24E-03x + 1.22E-02
R² = 1.00E+00
0
1000
2000
3000
4000
5000
6000
7000
8000
Time (s)

Methoxy butenolide (1a) and NVP (3:1) in NMP

Poly(1a-co-NVP) 3:1 in NMP					k1(obs)	4.14 x 10^{-3} [s^{-1}]
	1a		N-vinyl pyrrolidone		k2(obs)	4.33 x 10^{-4} [s^{-1}]
Time [s]	Integral	Concentration [mol/L]	Integral	Concentration [mol/L]	Conversion	ln(1/[1-conversion])
0	1.29	3.00	0.48	1.00	0%	0.00
120	0.97	2.26	0.09	0.18	39%	0.50
240	0.77	1.80	0.03	0.07	53%	0.76
480	0.66	1.54	0.01	0.02	61%	0.94
960	0.54	1.26	0.01	0.01	68%	1.15
1800	0.39	0.91	0.01	0.01	77%	1.46
7200	0.27	0.63	0.01	0.01	84%	1.83

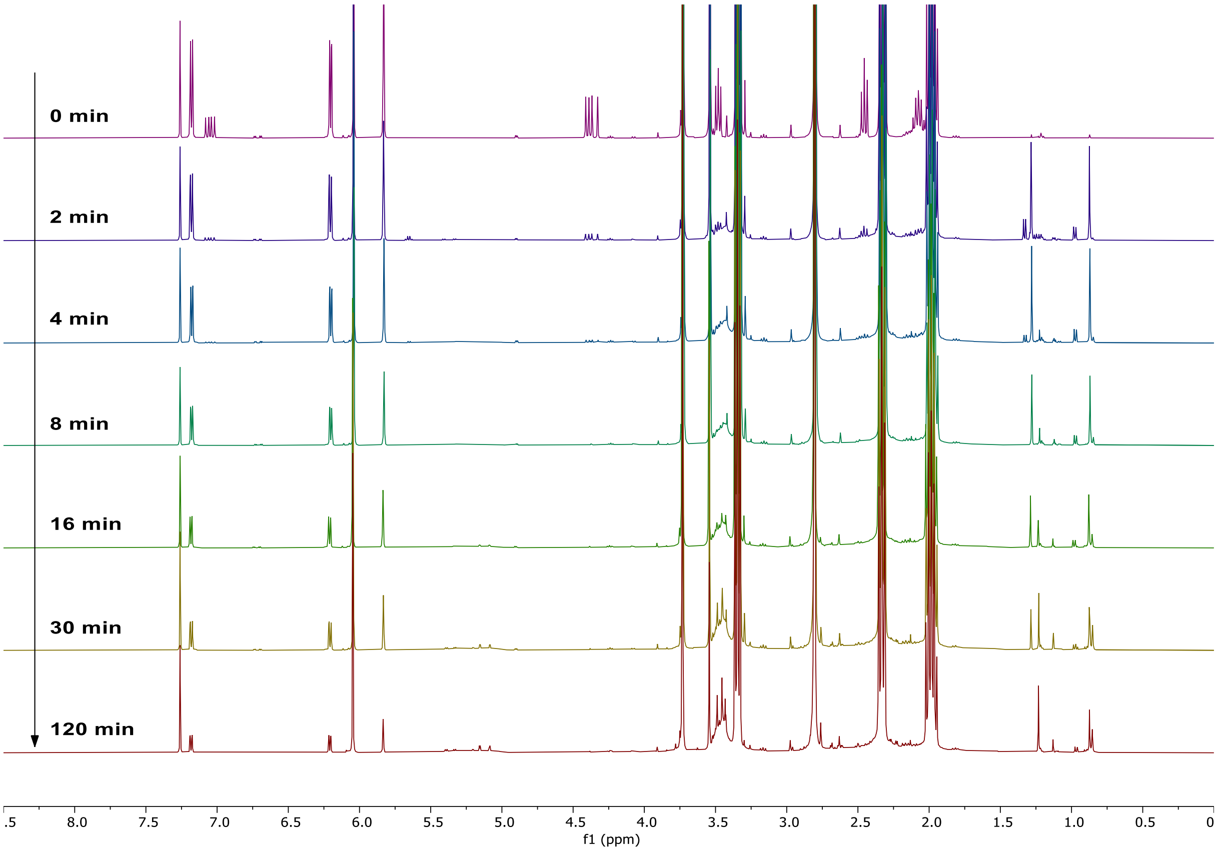

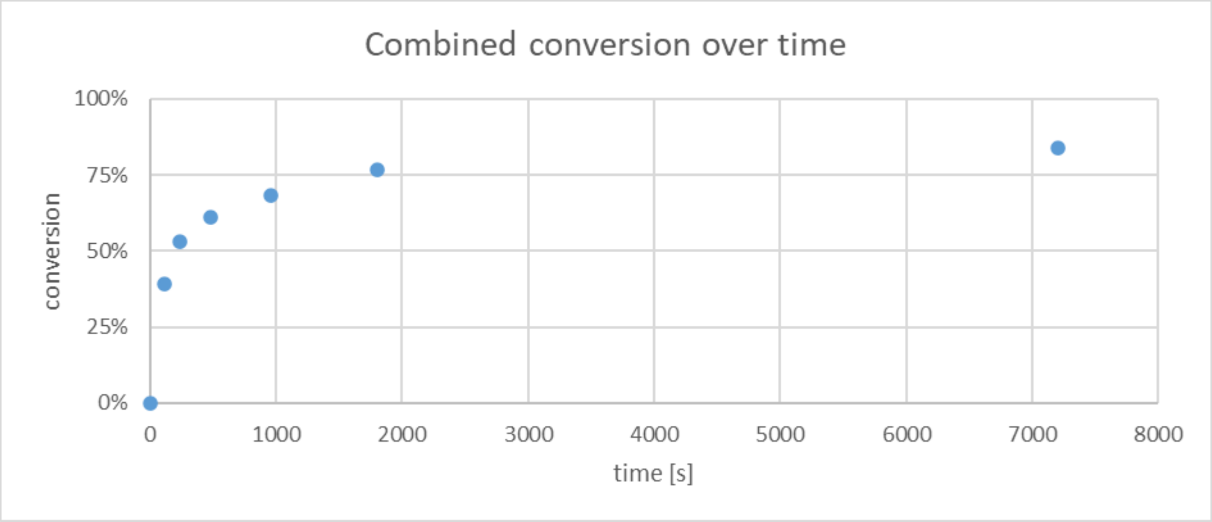
Combined conversion over time
conversion
100%
75%
50%
25%
0%
0
1000
2000
3000
4000
5000
6000
7000
8000
time [s]

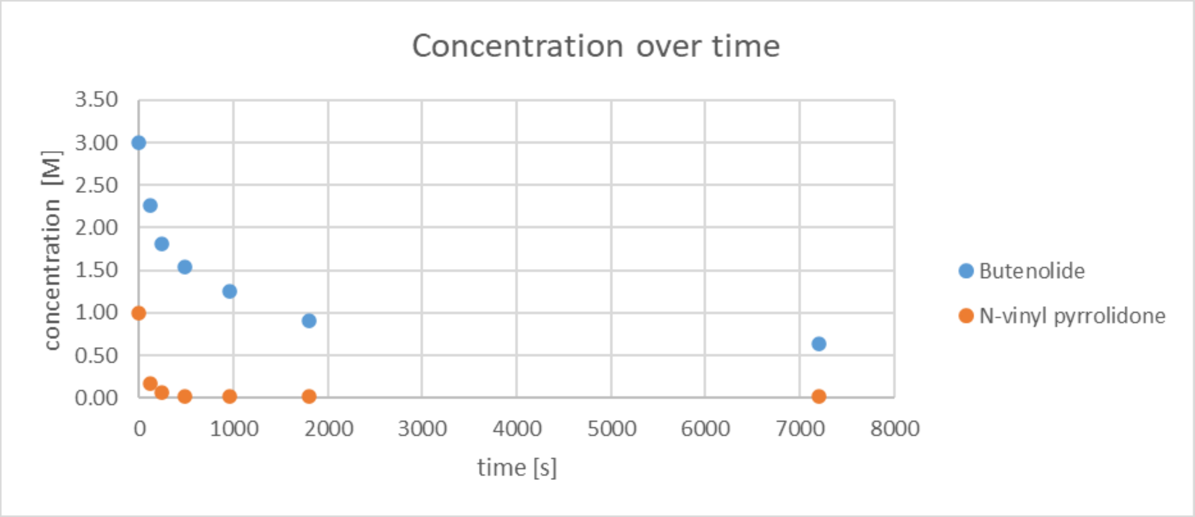
Concentration over time
concentration [M]
3.50
3.00
2.50
2.00
1.50
1.00
0.50
0.00
0
1000
2000
3000
4000
5000
6000
7000
8000
time [s]
Butenolide
N-vinyl pyrrolidone

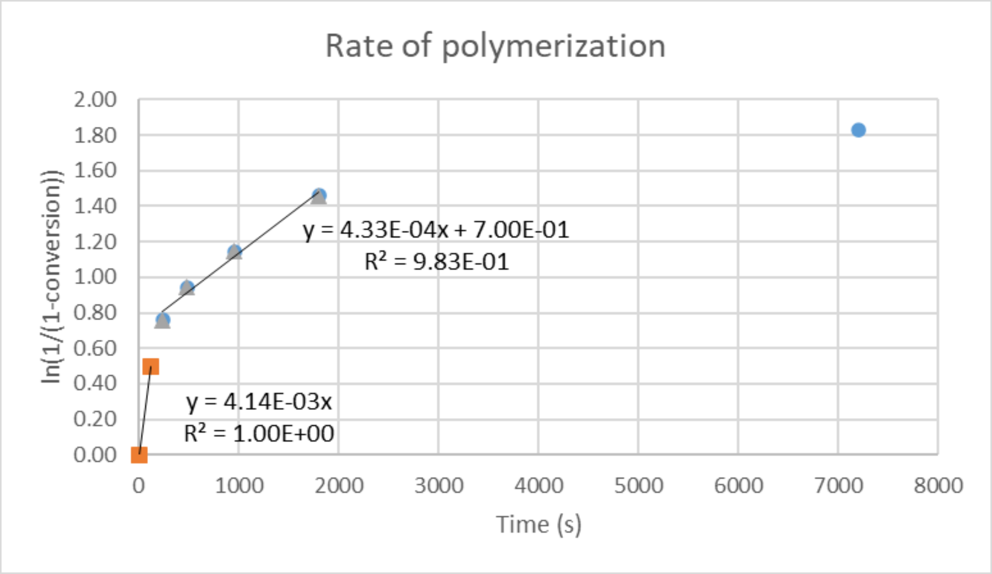
Rate of polymerization
ln(1/(1-conversion))
2.00
1.80
1.60
1.40
1.20
1.00
0.80
0.60
0.40
0.20
0.00
y = 4.33E-04x + 7.00E-01
R² = 9.83E-01
y = 4.14E-03x
R² = 1.00E+00
0
1000
2000
3000
4000
5000
6000
7000
8000
Time (s)

Methoxy butenolide (1a) in NMP

Poly(1a) in NMP			k1(obs)	1.93 x 10^{-4} [s^{-1}]
	1a		k2(obs)	n/a
Time [s]	Integral	Concentration [mol/L]	Conversion	ln(1/[1-conversion])
0	1.69	4.00	0%	0.00
120	1.67	3.95	1%	0.01
240	1.61	3.81	5%	0.05
480	1.55	3.67	8%	0.09
960	1.41	3.33	17%	0.18
1800	1.29	3.06	24%	0.27
7200	1.22	2.89	28%	0.33

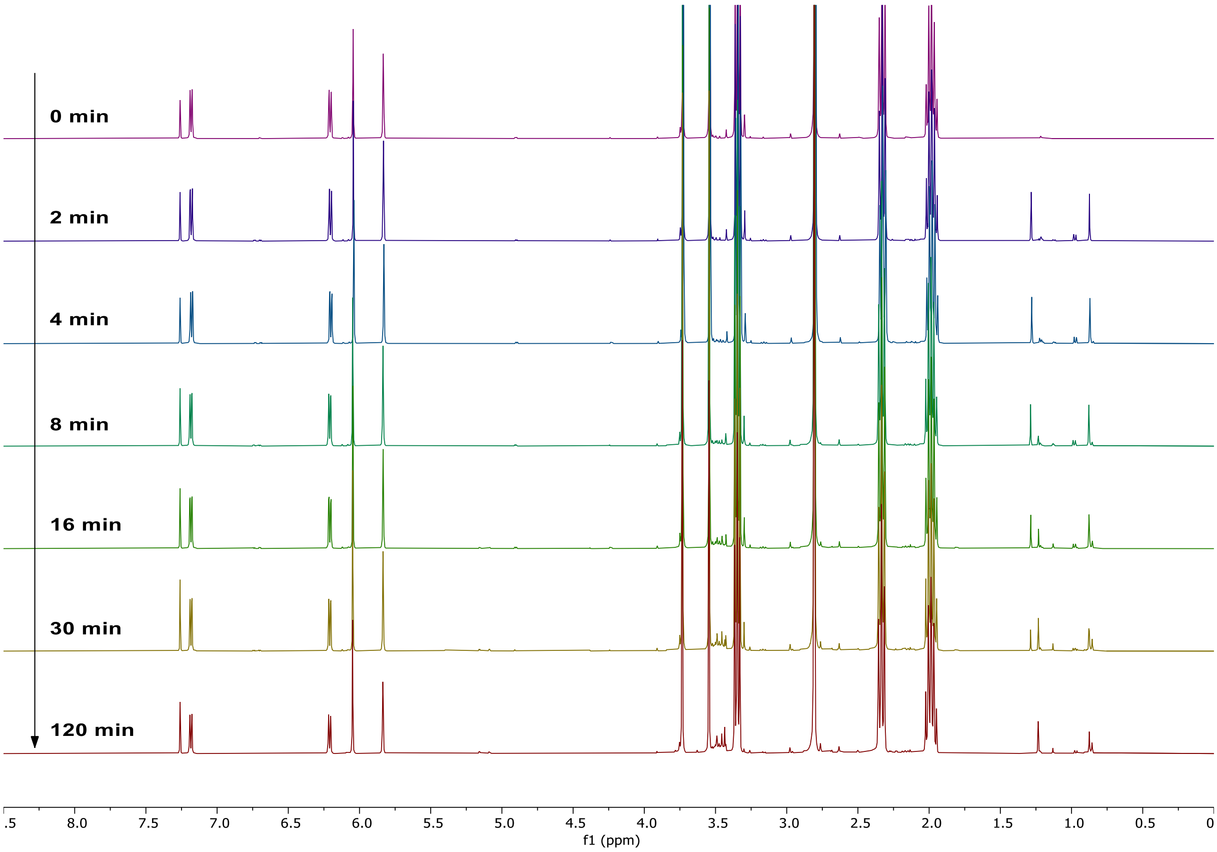

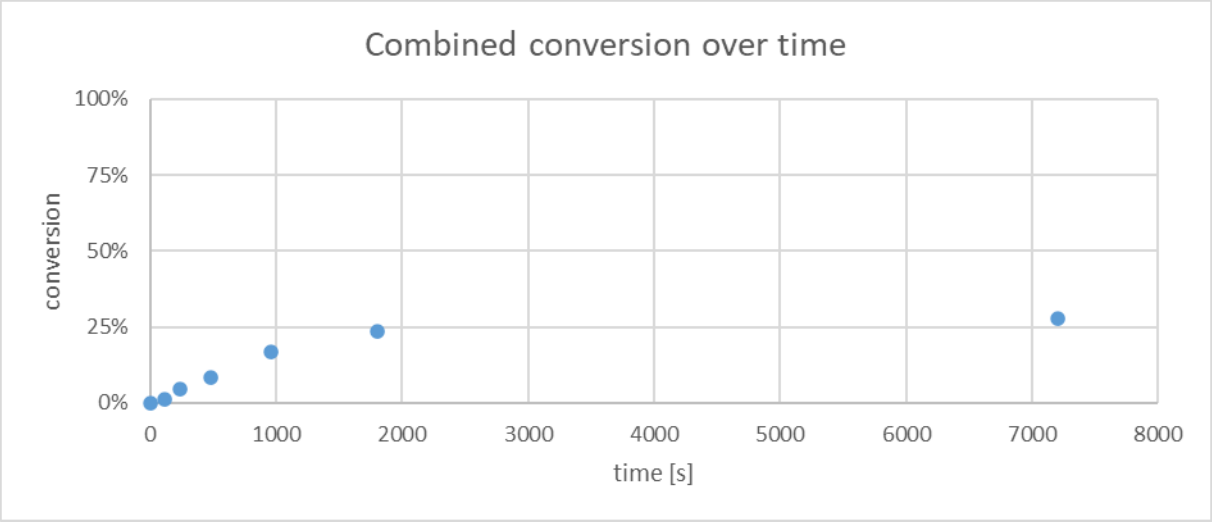
Combined conversion over time
conversion
100%
75%
50%
25%
0%
0
1000
2000
3000
4000
5000
6000
7000
8000
time [s]

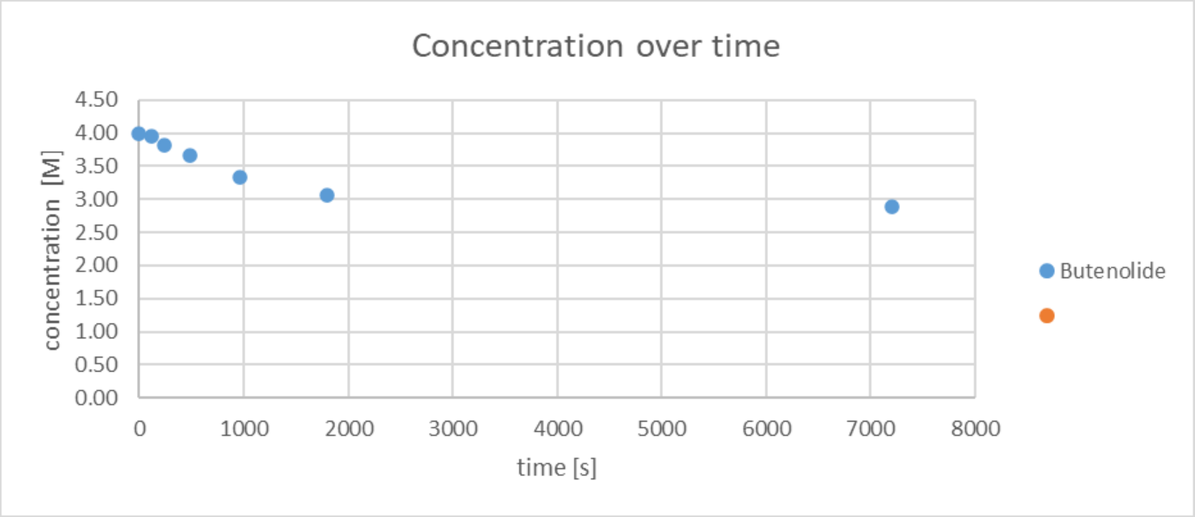
Concentration over time
concentration [M]
4.50
4.00
3.50
3.00
2.50
2.00
1.50
1.00
0.50
0.00
0
1000
2000
3000
4000
5000
6000
7000
8000
time [s]
Butenolide

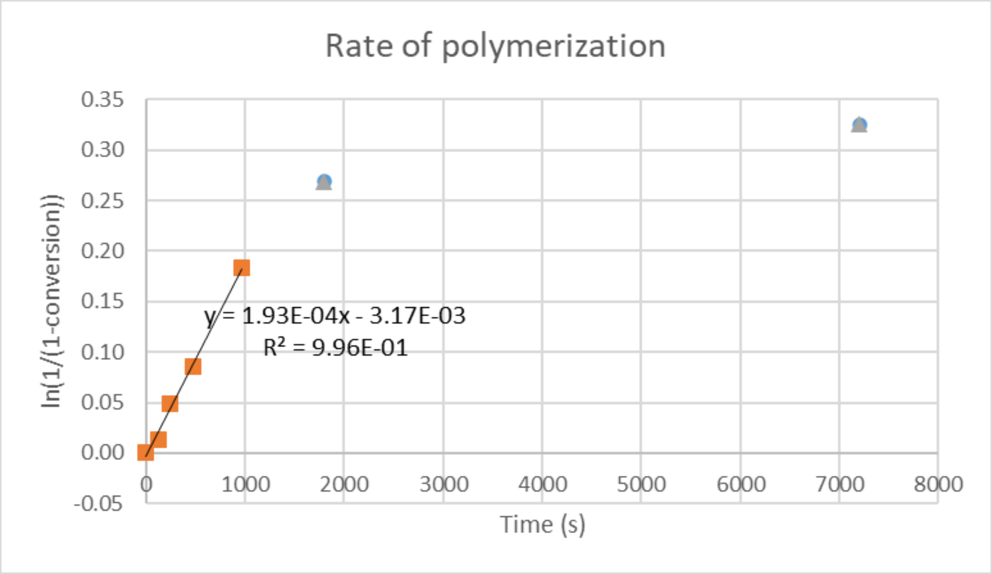
Rate of polymerization
ln(1/(1-conversion))
0.35
0.30
0.25
0.20
0.15
0.10
0.05
0.00
-0.05
y = 1.93E-04x - 3.17E-03
R² = 9.96E-01
0
1000
2000
3000
4000
5000
6000
7000
8000
Time (s)

Methoxy butenolide (1a) and NVP (1:1) in 1M2P

Poly(1a-co-NVP)					k1(obs)	2.99 . 10^{-3} [s^{-1}]
	1a		N-vinylpyrrolidone		k2(obs)	n/a
Time [s]	Integral	Concentration [mol/L]	Integral	Concentration [mol/L]	Conversion	ln(1/[1-conversion])
0	0.91	2.00	0.98	2.00	0%	0.00
60	0.83	1.83	0.83	1.70	12%	0.12
120	0.72	1.58	0.68	1.40	25%	0.29
240	0.50	1.09	0.41	0.85	52%	0.72
480	0.25	0.54	0.15	0.30	79%	1.56
900	0.10	0.23	0.03	0.06	93%	2.64
1800	0.02	0.05	0.00	0.00	99%	4.31
2700	0.00	0.01	0.00	0.00	100%	6.22
3600	0.00	0.00	0.00	0.00	100%	6.24
5400	0.00	0.00	0.00	0.01	100%	5.77

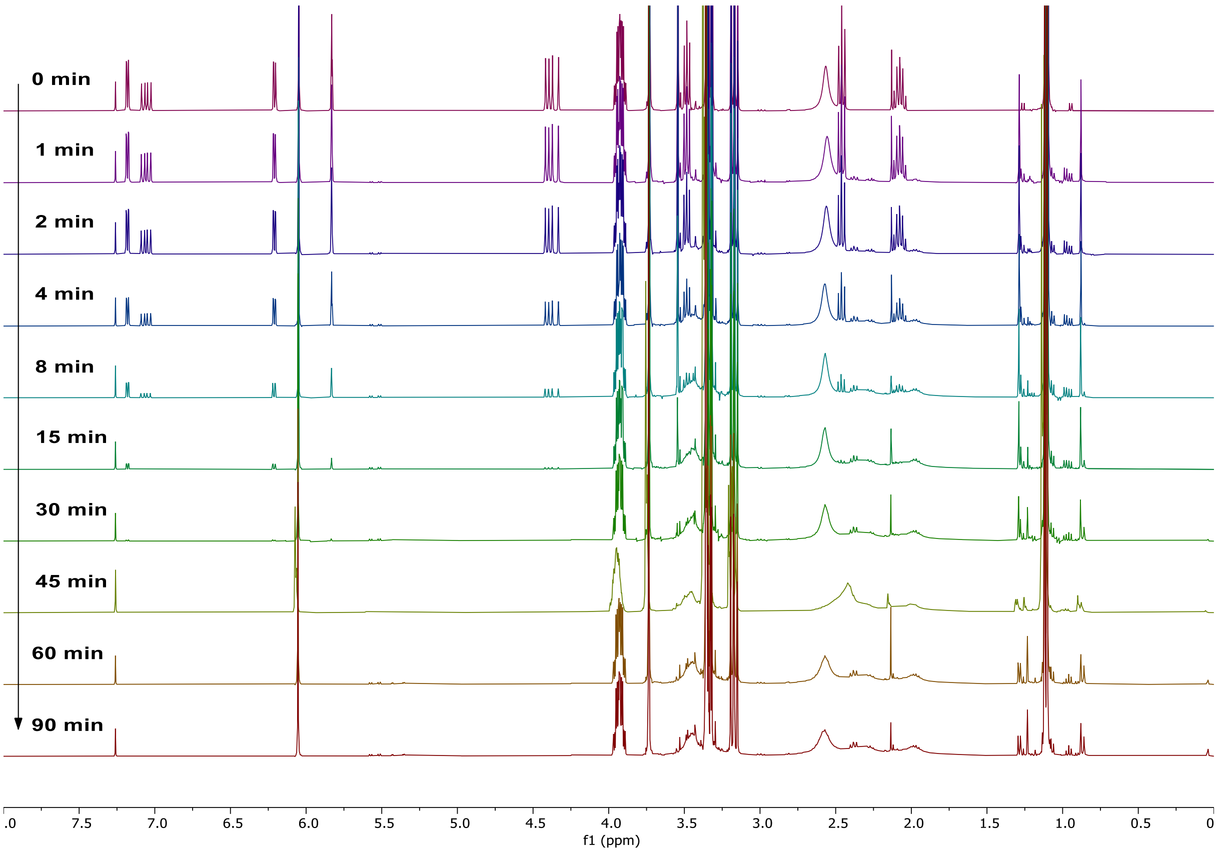

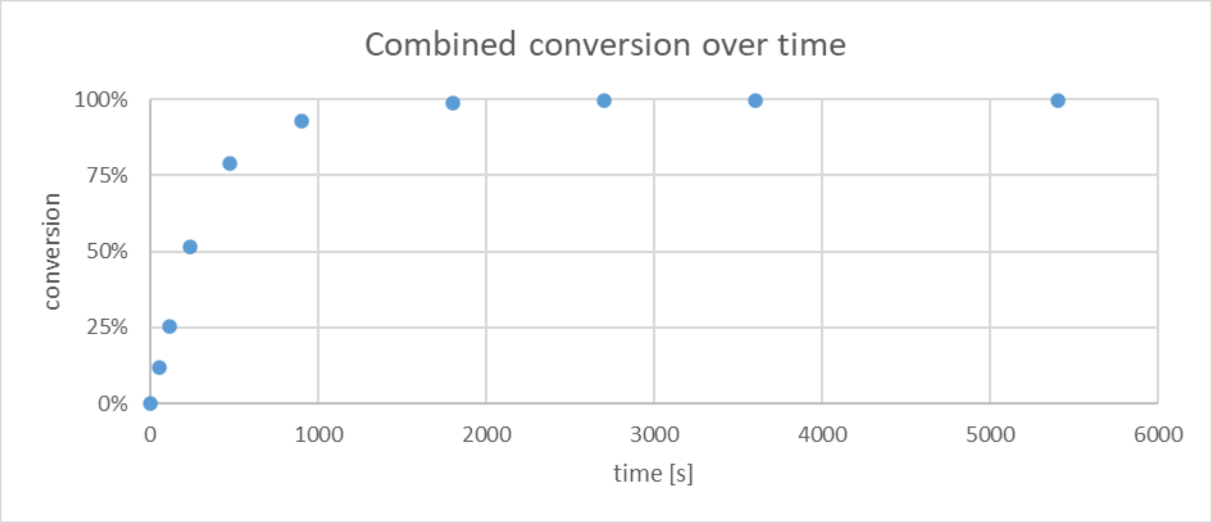
Combined conversion over time
conversion
100%
75%
50%
25%
0%
0
1000
2000
3000
4000
5000
6000
time [s]

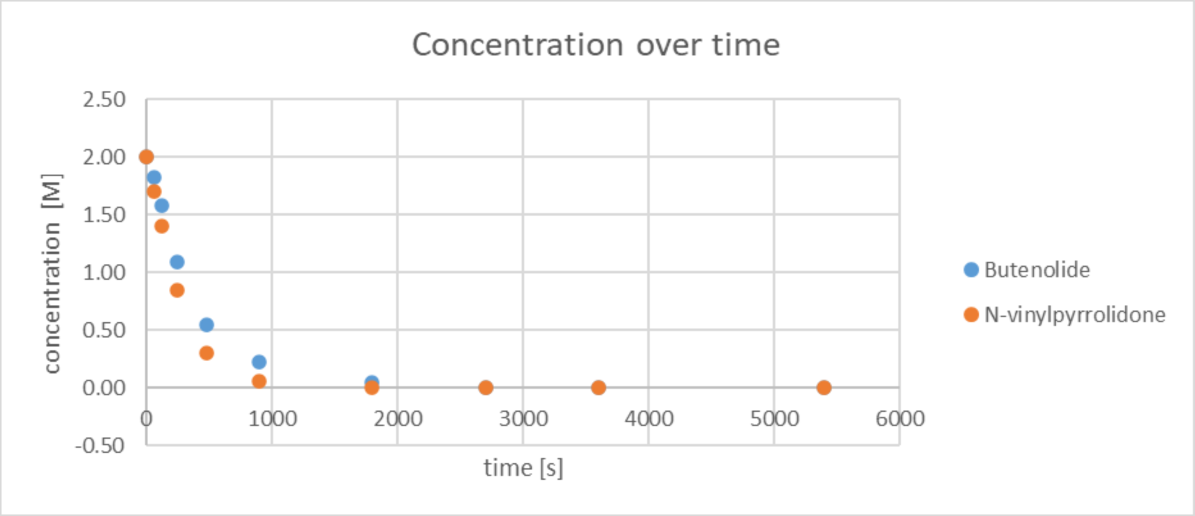
Concentration over time
concentration [M]
2.50
2.00
1.50
1.00
0.50
0.00
-0.50
0
1000
2000
3000
4000
5000
6000
time [s]
Butenolide
N-vinylpyrrolidone

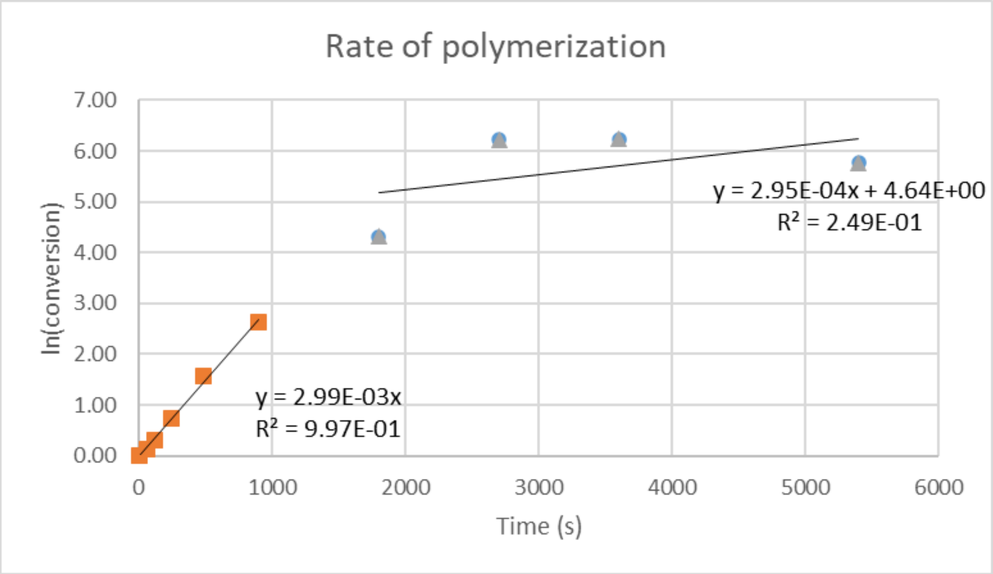
Rate of polymerization
ln(conversion)
7.00
6.00
5.00
4.00
3.00
2.00
1.00
0.00
0
1000
2000
3000
4000
5000
6000
Time (s)
y = 2.95E-04x + 4.64E+00
R² = 2.49E-01
y = 2.99E-03x
R² = 9.97E-01

Acetoxy butenolide (2a) and DVE (1:3) in AcOBu

Poly(2a-co-DVE) 1:3 in AcOBu					k1(obs)	1.33×10^{-3} [s^{-1}]
	2a		Dodecyl vinyl ether		k2(obs)	4.53×10^{-5} [s^{-1}]
Time [s]	Integral	Concentration [mol/L]	Integral	Concentration [mol/L]	Conversion	ln(1/[1-conversion])
0	0.46	1.00	1.45	3.00	0%	0.00
120	0.41	0.89	1.41	2.92	5%	0.05
240	0.21	0.45	1.25	2.59	24%	0.28
480	0.02	0.05	1.03	2.13	45%	0.61
960	0.00	0.00	0.96	1.99	50%	0.70
1800	0.00	0.00	0.87	1.80	55%	0.80
7200	0.00	0.00	0.71	1.47	63%	1.00

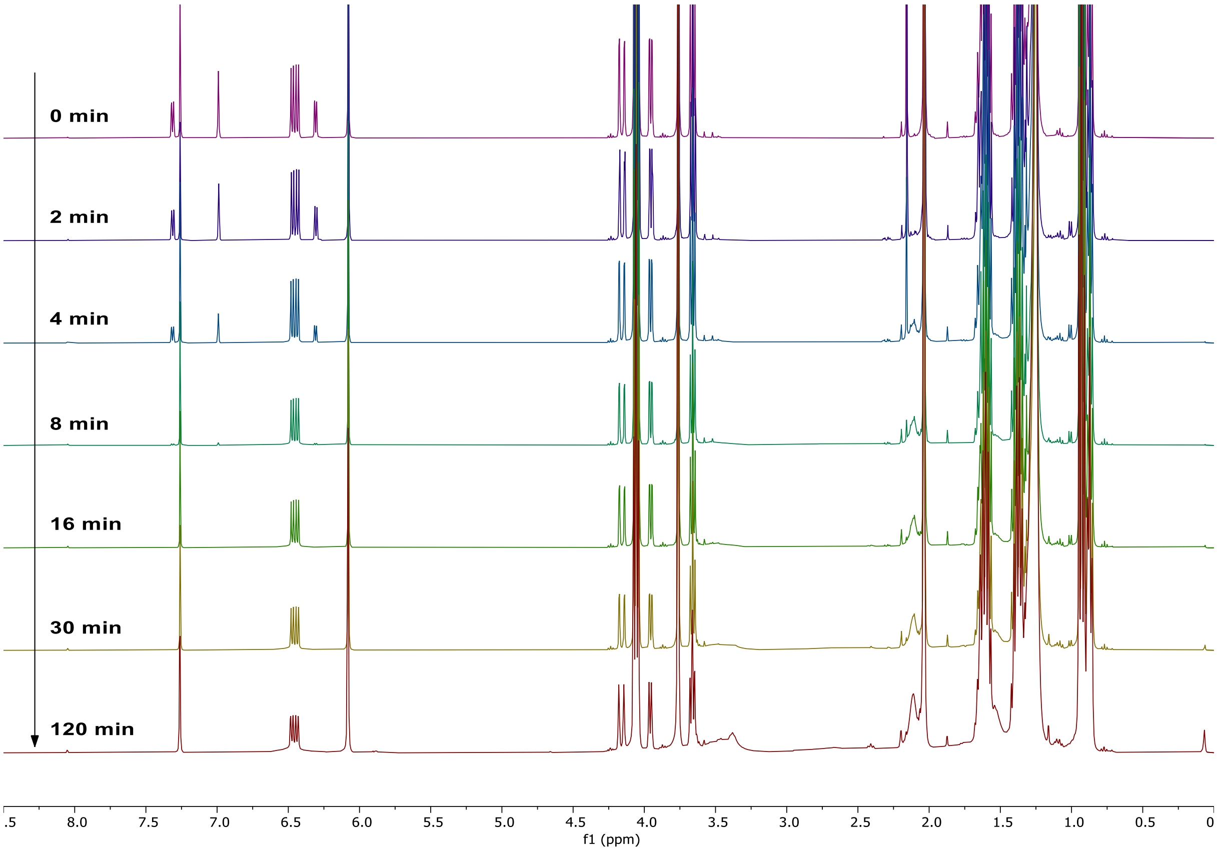

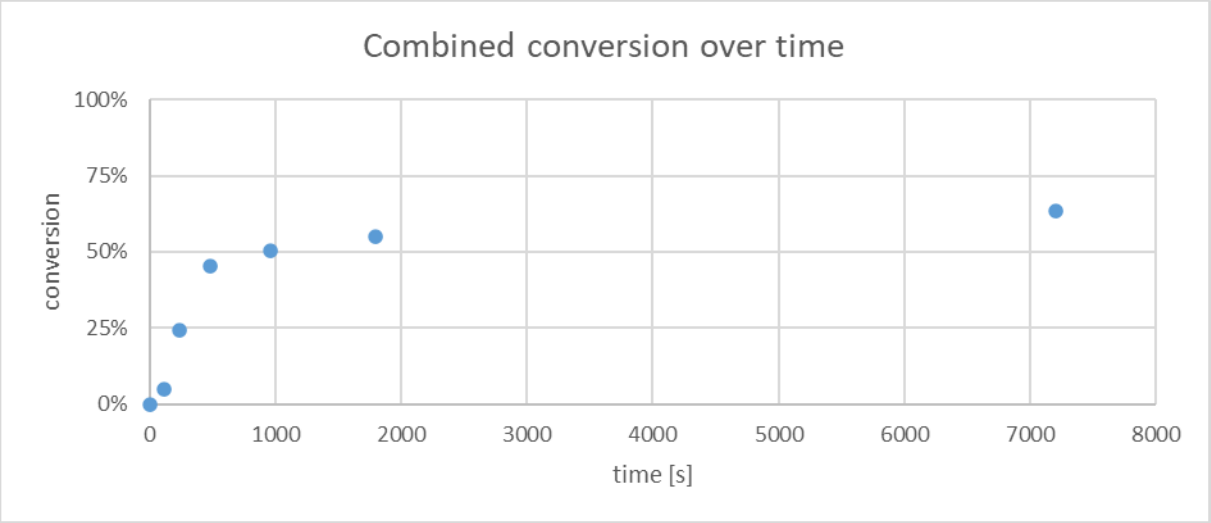
Combined conversion over time
conversion
100%
75%
50%
25%
0%
0
1000
2000
3000
4000
5000
6000
7000
8000
time [s]

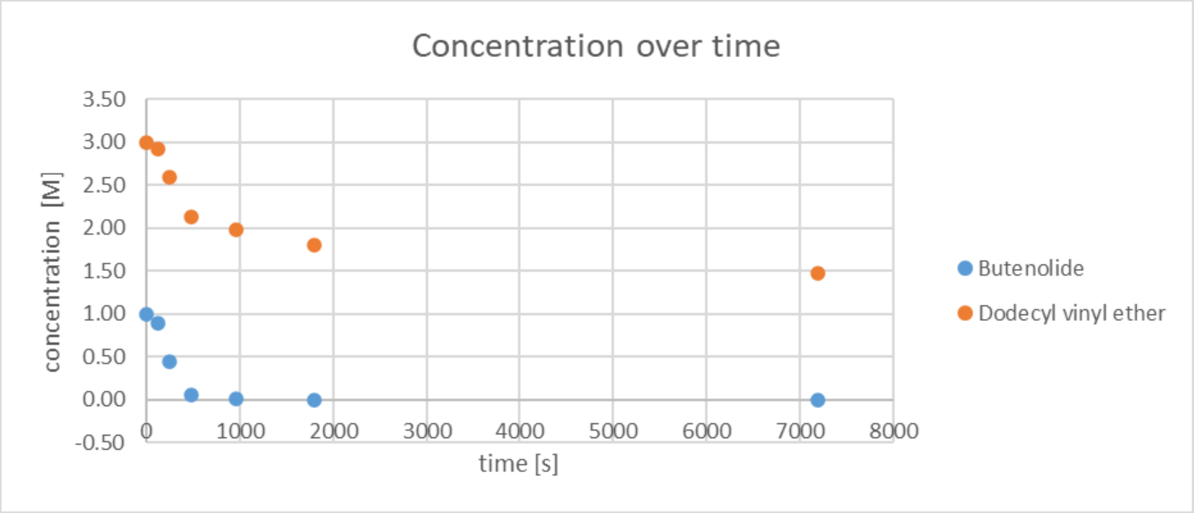
Concentration over time
concentration [M]
3.50
3.00
2.50
2.00
1.50
1.00
0.50
0.00
-0.50
0
1000
2000
3000
4000
5000
6000
7000
8000
time [s]
Butenolide
Dodecyl vinyl ether

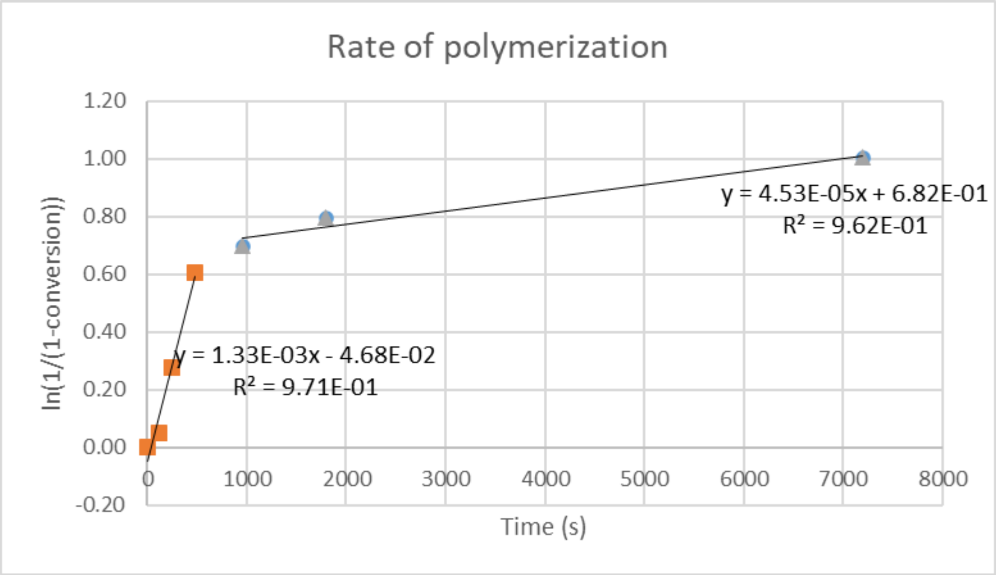
Rate of polymerization
ln(1/(1-conversion))
1.20
1.00
0.80
0.60
0.40
0.20
0.00
-0.20
0
1000
2000
3000
4000
5000
6000
7000
8000
Time (s)
y = 4.53E-05x + 6.82E-01
R² = 9.62E-01
y = 1.33E-03x - 4.68E-02
R² = 9.71E-01

Acetoxy butenolide (2a) and DVE (3:1) in AcOBu

Poly(2a-co-DVE) 3:1 in AcOBu					k1(obs)	1.31×10^{-3} [s^{-1}]
	2a		Dodecyl vinyl ether		k2(obs)	6.45×10^{-5} [s^{-1}]
Time [s]	Integral	Concentration [mol/L]	Integral	Concentration [mol/L]	Conversion	ln(1/[1-conversion])
0	1.38	3.00	0.44	1.00	0%	0.00
120	1.26	2.73	0.35	0.79	12%	0.13
240	1.11	2.41	0.25	0.58	25%	0.29
480	0.86	1.87	0.12	0.28	46%	0.62
960	0.65	1.40	0.01	0.02	64%	1.03
1800	0.52	1.13	0.00	0.00	72%	1.26
7200	0.41	0.89	0.00	0.00	78%	1.50

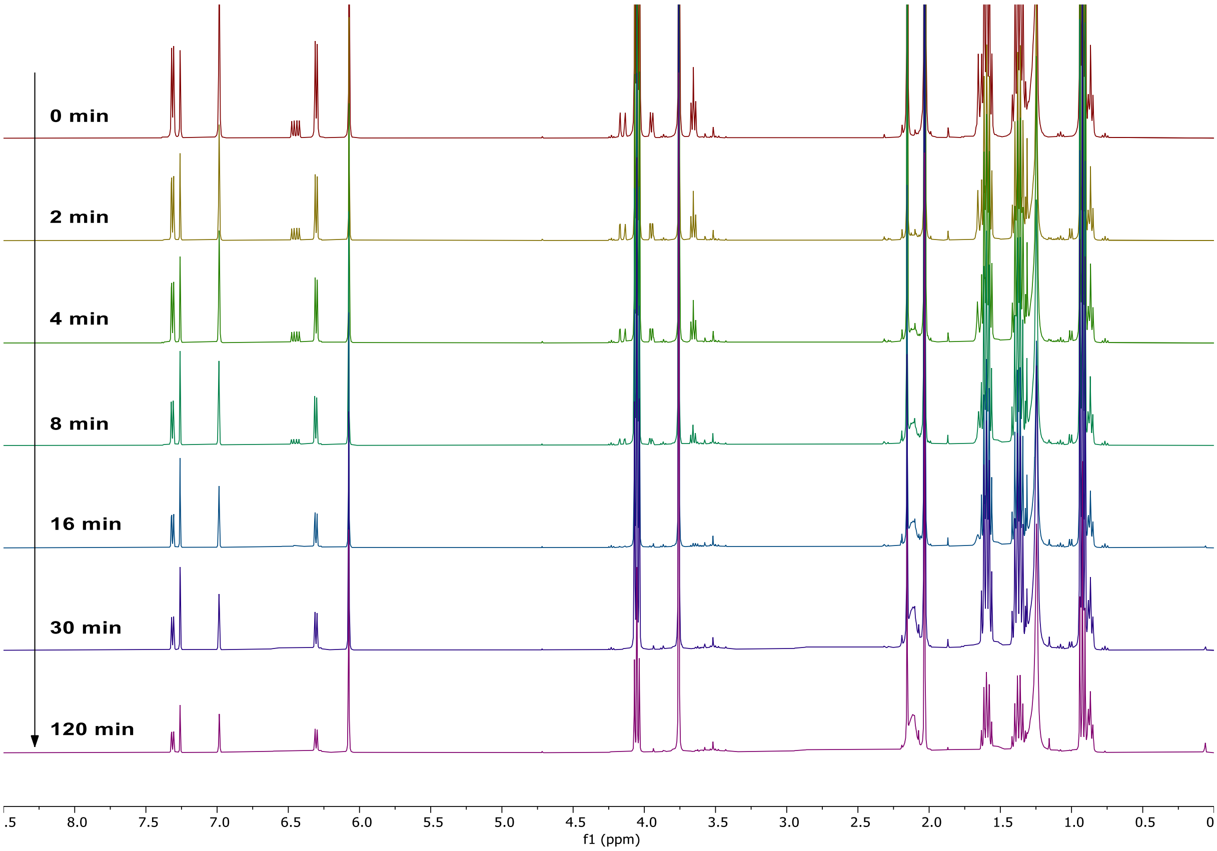

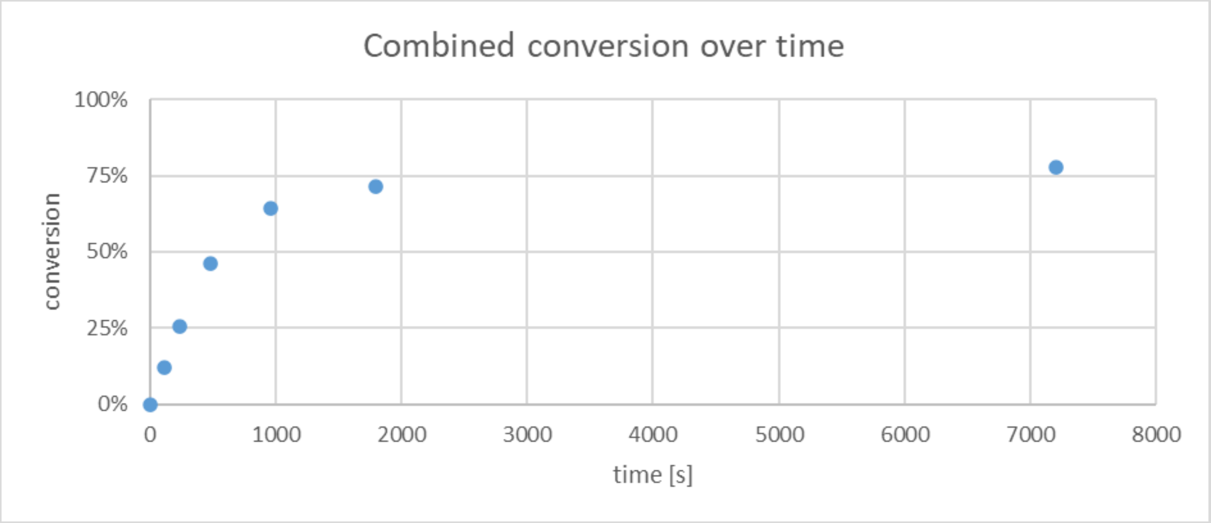
Combined conversion over time
conversion
100%
75%
50%
25%
0%
0
1000
2000
3000
4000
5000
6000
7000
8000
time [s]

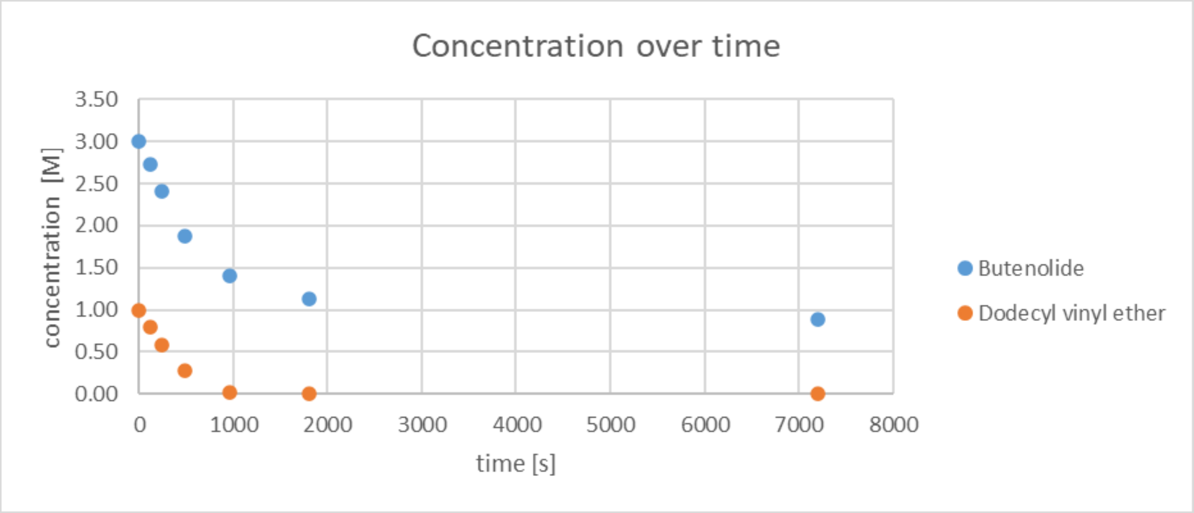
Concentration over time
concentration [M]
3.50
3.00
2.50
2.00
1.50
1.00
0.50
0.00
0
1000
2000
3000
4000
5000
6000
7000
8000
time [s]
Butenolide
Dodecyl vinyl ether

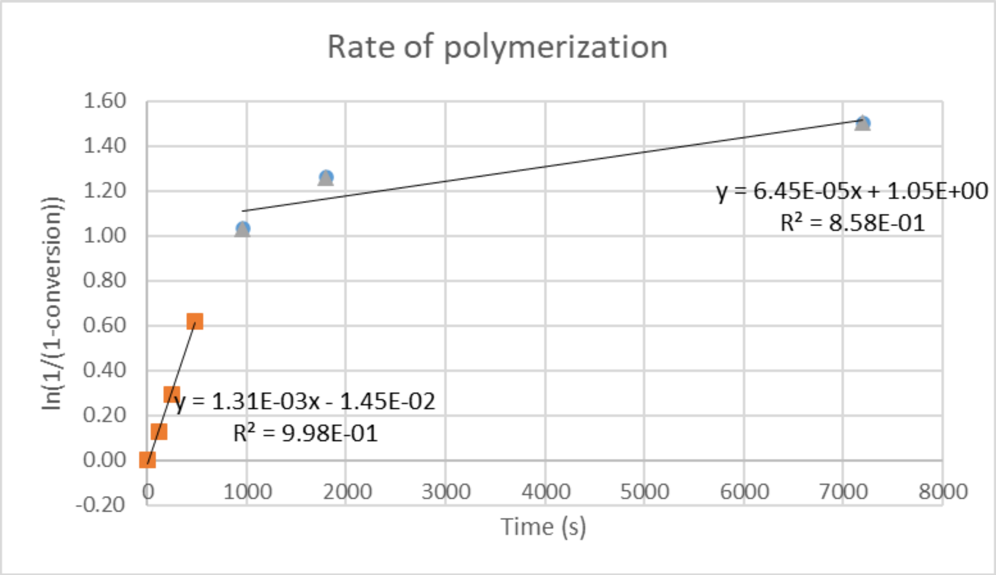
Rate of polymerization
ln(1/(1-conversion))
1.60
1.40
1.20
1.00
0.80
0.60
0.40
0.20
0.00
-0.20
0
1000
2000
3000
4000
5000
6000
7000
8000
Time (s)
y = 6.45E-05x + 1.05E+00
R² = 8.58E-01
y = 1.31E-03x - 1.45E-02
R² = 9.98E-01

Acetoxy butenolide (2a) in AcOBu

Poly(2a) in AcOBu			k1(obs)	4.52 x 10^{-5} [s^{-1}]
	2a		k2(obs)	n/a
Time [s]	Integral	Concentration [mol/L]	Conversion	ln(1/[1-conversion])
0	1.84	4.00	0%	0.00
120	1.87	4.06	-2%	-0.01
240	1.86	4.04	-1%	-0.01
480	1.86	4.03	-1%	-0.01
960	1.81	3.93	2%	0.02
1800	1.74	3.77	6%	0.06
7200	1.57	3.40	15%	0.16

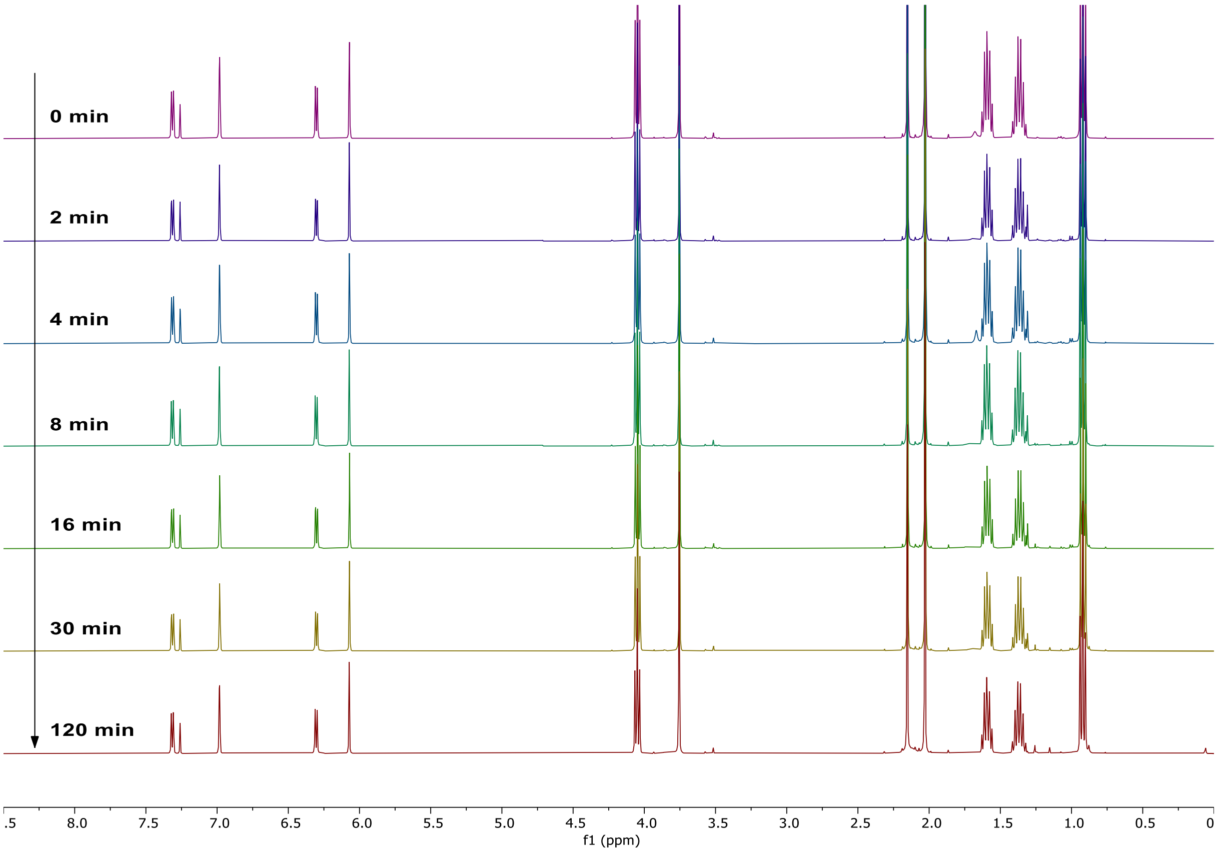

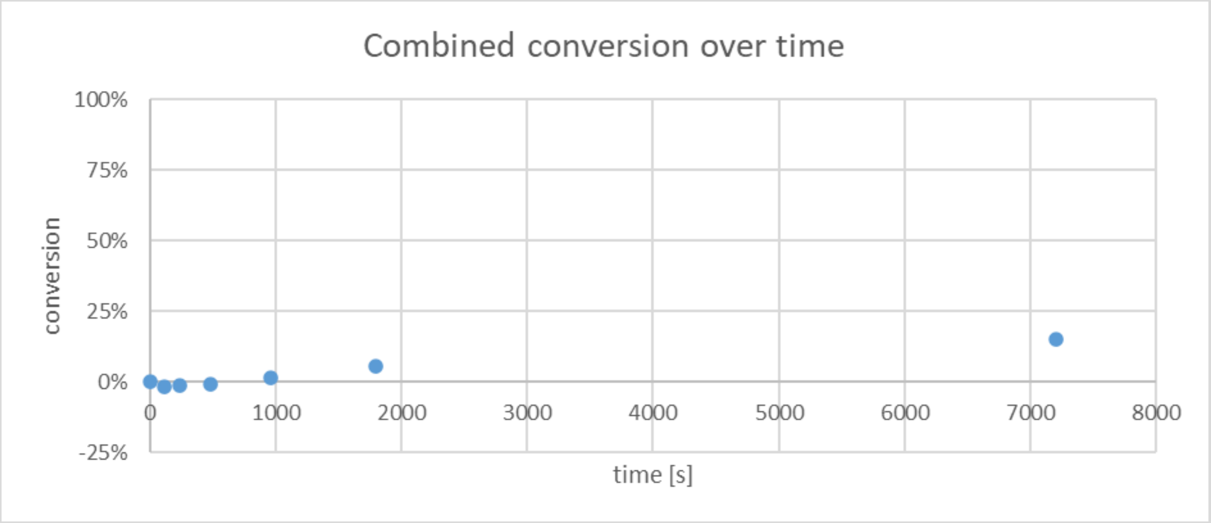
Combined conversion over time
conversion
100%
75%
50%
25%
0%
-25%
0
1000
2000
3000
4000
5000
6000
7000
8000
time [s]

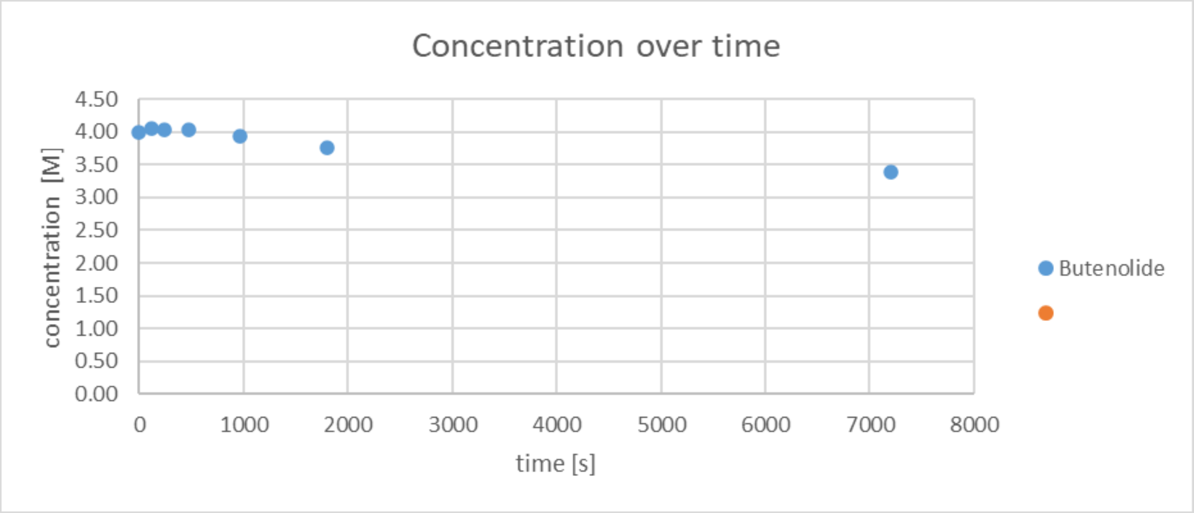
Concentration over time
concentration [M]
4.50
4.00
3.50
3.00
2.50
2.00
1.50
1.00
0.50
0.00
0
1000
2000
3000
4000
5000
6000
7000
8000
time [s]
Butenolide

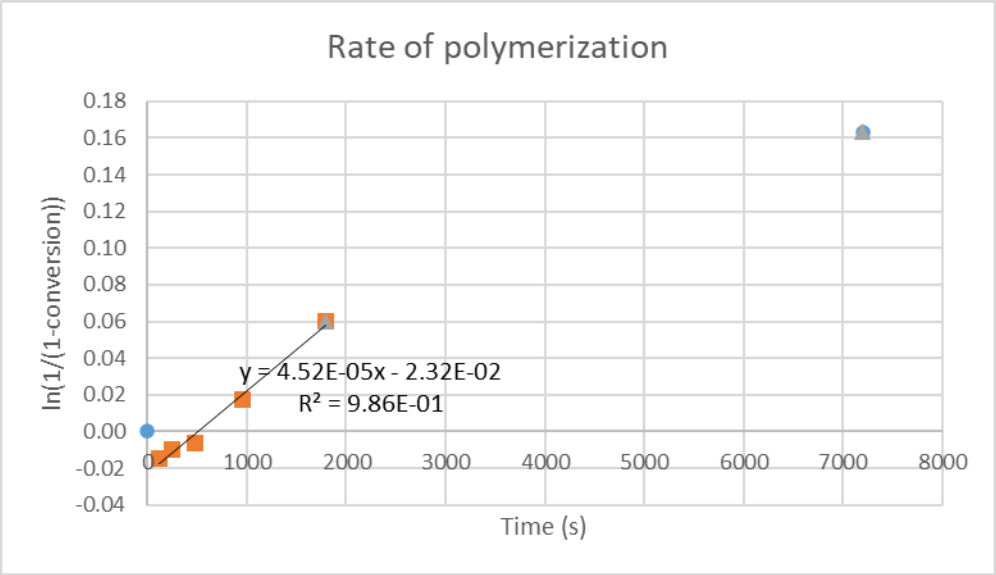
Rate of polymerization
ln(1/(1-conversion))
0.18
0.16
0.14
0.12
0.10
0.08
0.06
0.04
0.02
0.00
-0.02
-0.04
0
1000
2000
3000
4000
5000
6000
7000
8000
y = 4.52E-05x - 2.32E-02
R² = 9.86E-01
Time (s)

Acetoxy butenolide (2a) and EGVE in NMP

Poly(2aco-EGVE) in NMP					k1(obs)	1.11×10^{-2} [s^{-1}]
	2a		Ethylene glycol vinyl ether		k2(obs)	9.66×10^{-5} [s^{-1}]
Time [s]	Integral	Concentration [mol/L]	Integral	Concentration [mol/L]	Conversion	ln(1/[1-conversion])
0	0.95	2.00	1.02	2.00	0%	0.00
30	0.46	0.98	0.64	1.25	44%	0.58
75	0.13	0.27	0.40	0.79	74%	1.33
150	0.03	0.06	0.34	0.67	82%	1.70
240	0.01	0.02	0.31	0.62	84%	1.84
480	0.01	0.01	0.29	0.57	85%	1.92
960	0.00	0.01	0.28	0.55	86%	1.97
2400	0.01	0.02	0.24	0.46	88%	2.12
7200	0.02	0.03	0.14	0.27	92%	2.58

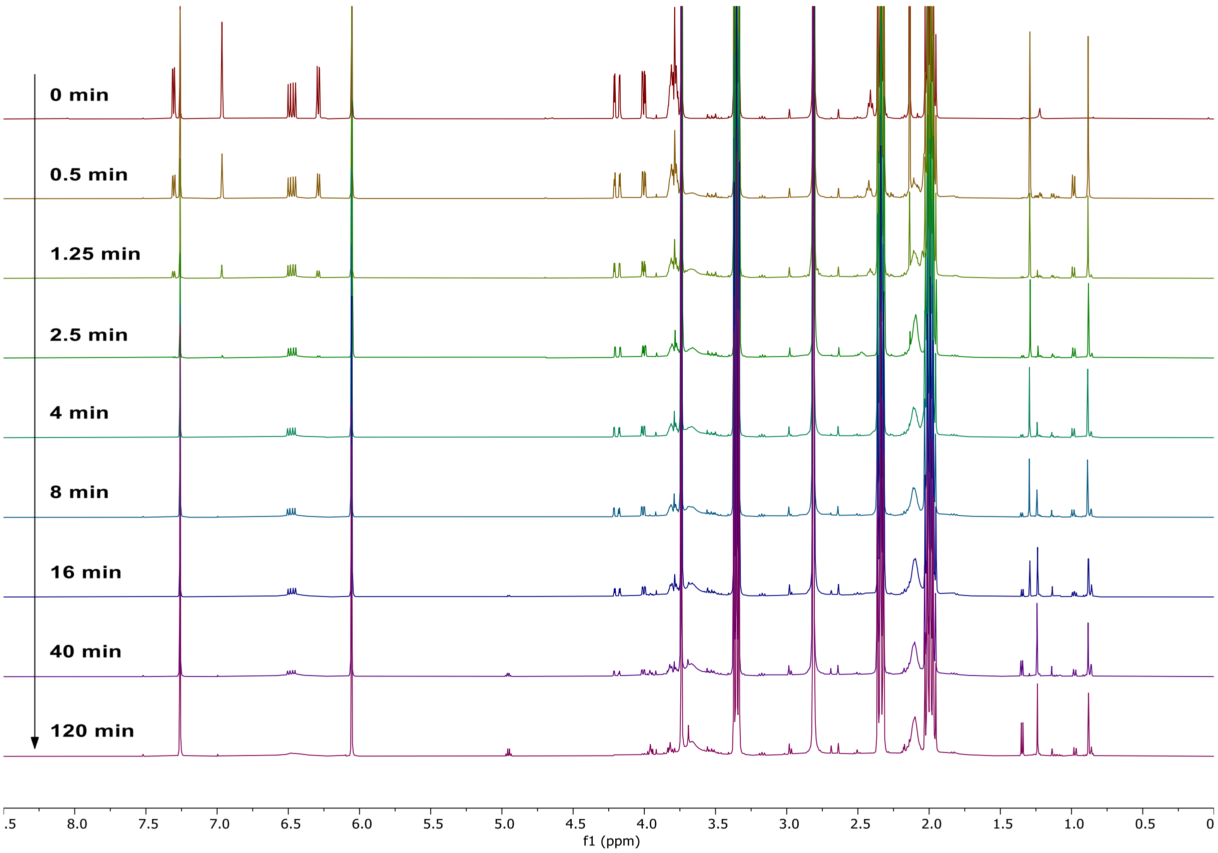

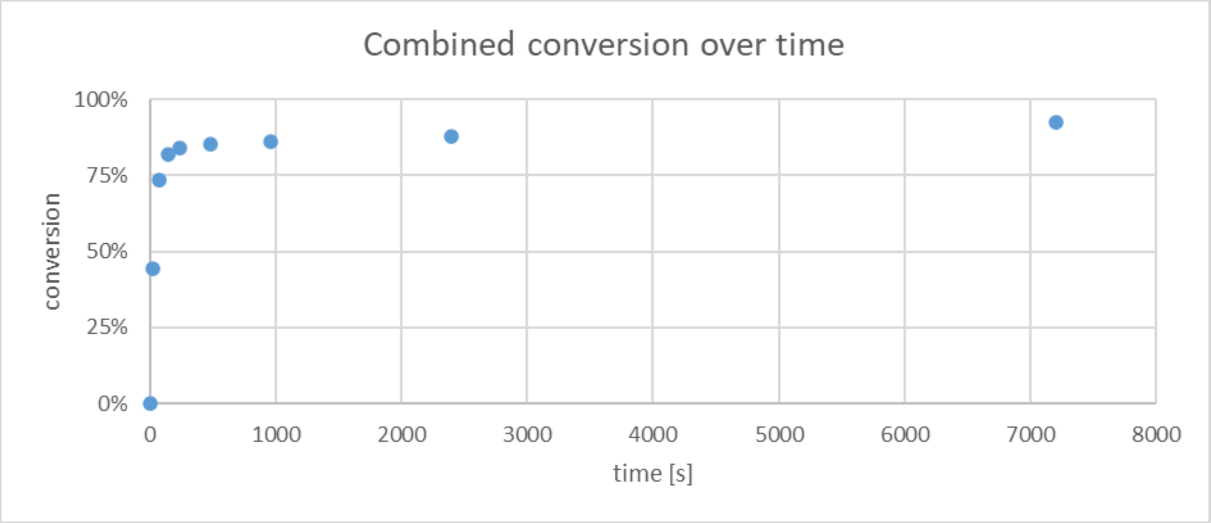
Combined conversion over time
conversion
100%
75%
50%
25%
0%
0
1000
2000
3000
4000
5000
6000
7000
8000
time [s]

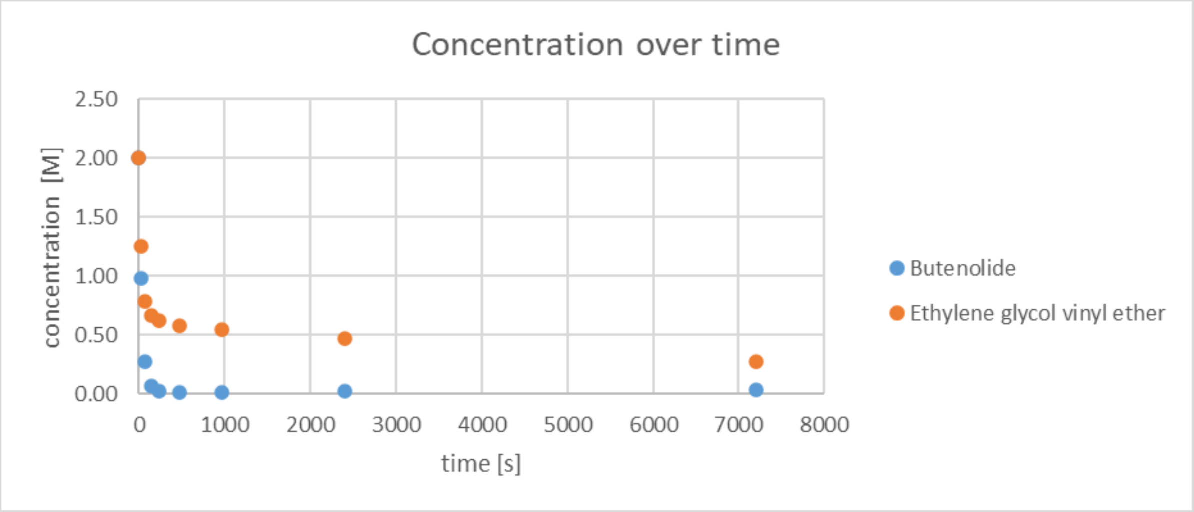
Concentration over time
concentration [M]
2.50
2.00
1.50
1.00
0.50
0.00
0
1000
2000
3000
4000
5000
6000
7000
8000
time [s]
Butenolide
Ethylene glycol vinyl ether

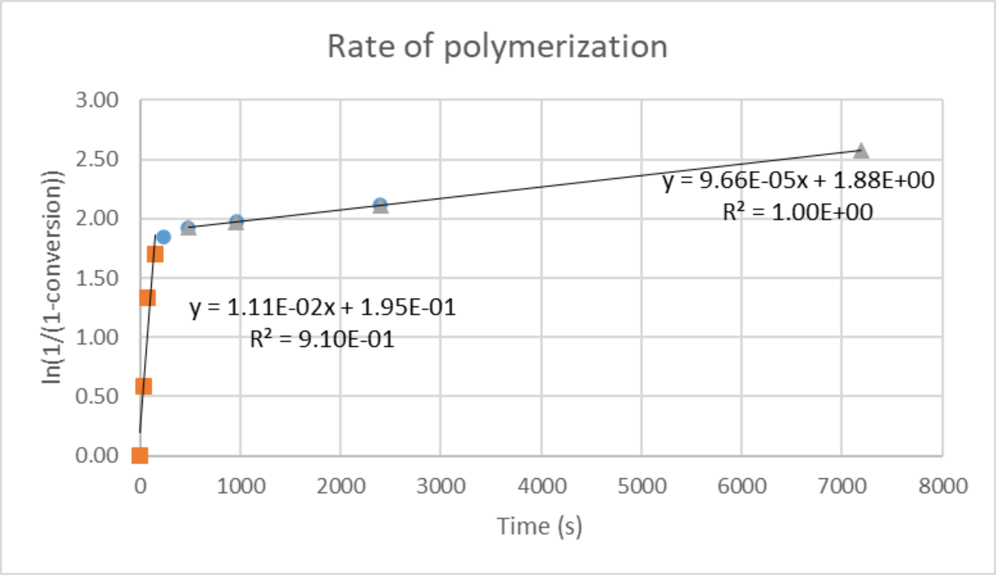
Rate of polymerization
ln(1/(1-conversion))
3.00
2.50
2.00
1.50
1.00
0.50
0.00
0
1000
2000
3000
4000
5000
6000
7000
8000
Time (s)
y = 9.66E-05x + 1.88E+00
R² = 1.00E+00
y = 1.11E-02x + 1.95E-01
R² = 9.10E-01

Acetoxy butenolide (2a) and NVP (1:3) in NMP

Poly(2a-co-NVP) (1:3)						k1(obs)	1.12×10^{-2} [s^{-1}]
	2a		N-vinyl pyrrolidone			k2(obs)	2.54×10^{-3} [s^{-1}]
**Time [s]**	**Integral**	**Concentration [mol/L]**	**Integral**	**Concentration [mol/L]**		**Conversion**	**ln(1/[1-conversion])**
0	0.46	1.00	1.43	3.00		0%	0.00
60	0.05	0.10	0.78	1.64		56%	0.83
120	0.00	0.00	0.54	1.13		72%	1.26
240	0.00	0.01	0.34	0.70		82%	1.73
480	0.00	0.01	0.13	0.28		93%	2.64
1020	0.00	0.01	0.05	0.10		97%	3.60
1500	0.01	0.01	0.03	0.06		98%	4.01
2400	0.01	0.01	0.02	0.04		99%	4.29
3600	0.01	0.01	0.01	0.03		99%	4.54
7200	0.01	0.02	0.02	0.05		98%	4.18

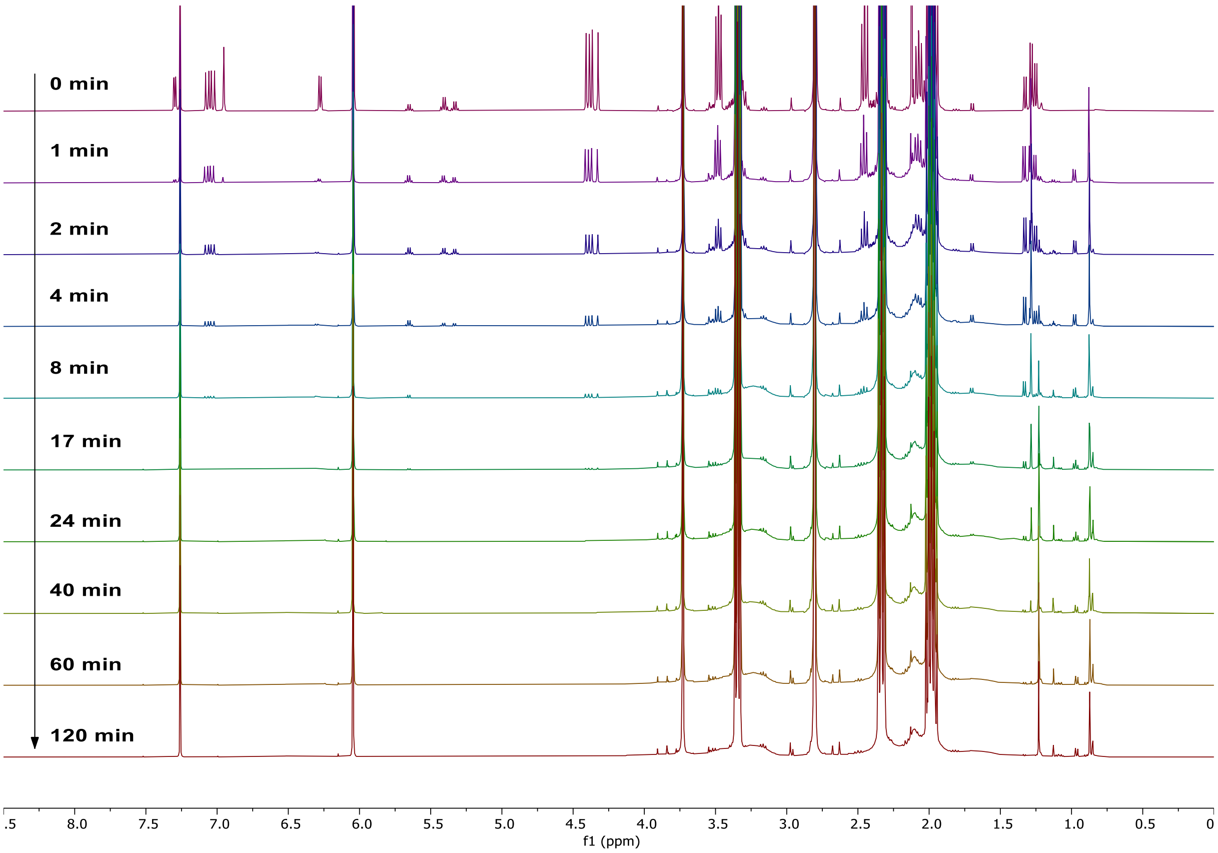

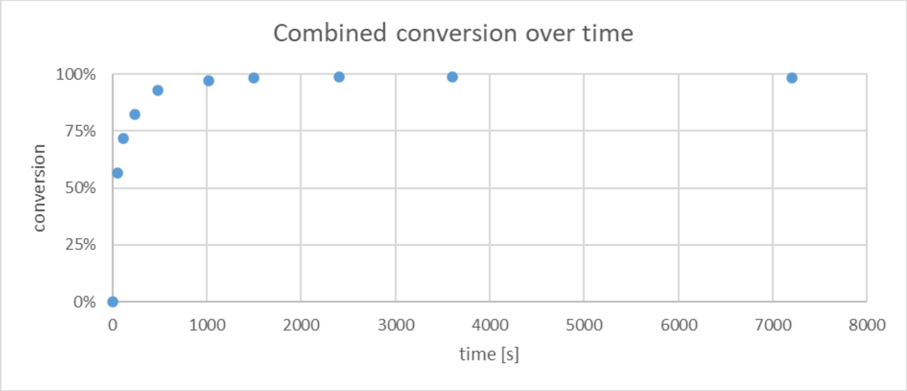
Combined conversion over time
conversion
100%
75%
50%
25%
0%
0
1000
2000
3000
4000
5000
6000
7000
8000
time [s]

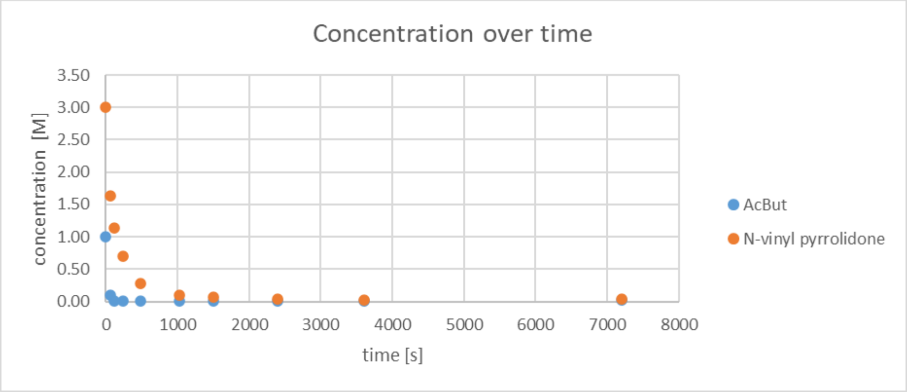
Concentration over time
concentration [M]
3.50
3.00
2.50
2.00
1.50
1.00
0.50
0.00
0
1000
2000
3000
4000
5000
6000
7000
8000
time [s]
AcBut
N-vinyl pyrrolidone

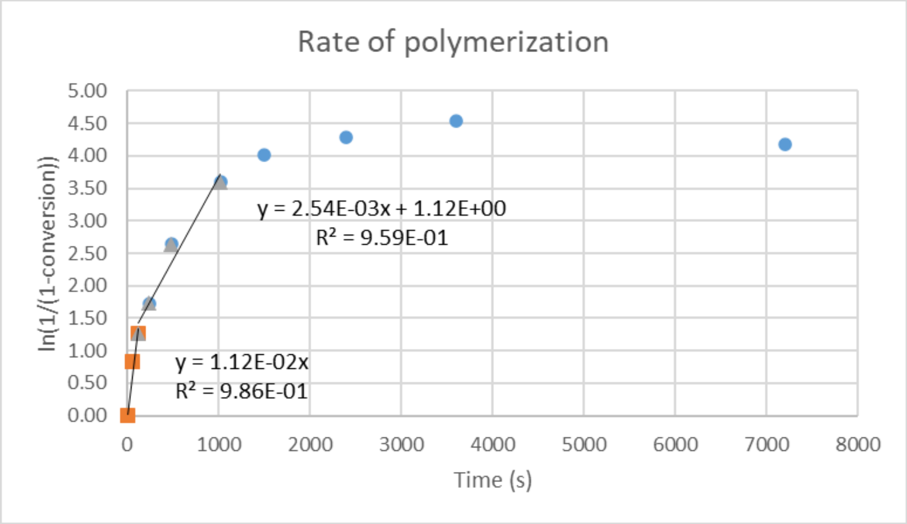
Rate of polymerization
ln(1/(1-conversion))
5.00
4.50
4.00
3.50
3.00
2.50
2.00
1.50
1.00
0.50
0.00
y = 2.54E-03x + 1.12E+00
R² = 9.59E-01
y = 1.12E-02x
R² = 9.86E-01
0
1000
2000
3000
4000
5000
6000
7000
8000
Time (s)

Acetoxy butenolide (2a) and NVP (1:1) in NMP

Poly(2a-co-NVP) in NMP 1:1					k1(obs)	1.67×10^{-2} [s^{-1}]
	2a		N-vinyl pyrrolidone		k2(obs)	4.81×10^{-5} [s^{-1}]
Time [s]	Integral	Concentration [mol/L]	Integral	Concentration [mol/L]	Conversion	ln(1/[1-conversion])
0	0.90	2.00	1.04	2.00	0%	0.00
30	0.49	1.10	0.58	1.12	45%	0.59
60	0.26	0.58	0.32	0.62	70%	1.21
120	0.11	0.24	0.15	0.30	87%	2.01
240	0.04	0.09	0.09	0.17	94%	2.76
480	0.01	0.02	0.05	0.10	97%	3.49
960	0.01	0.02	0.03	0.07	98%	3.90
1800	0.01	0.01	0.02	0.04	99%	4.29
7200	0.01	0.02	0.02	0.03	99%	4.34

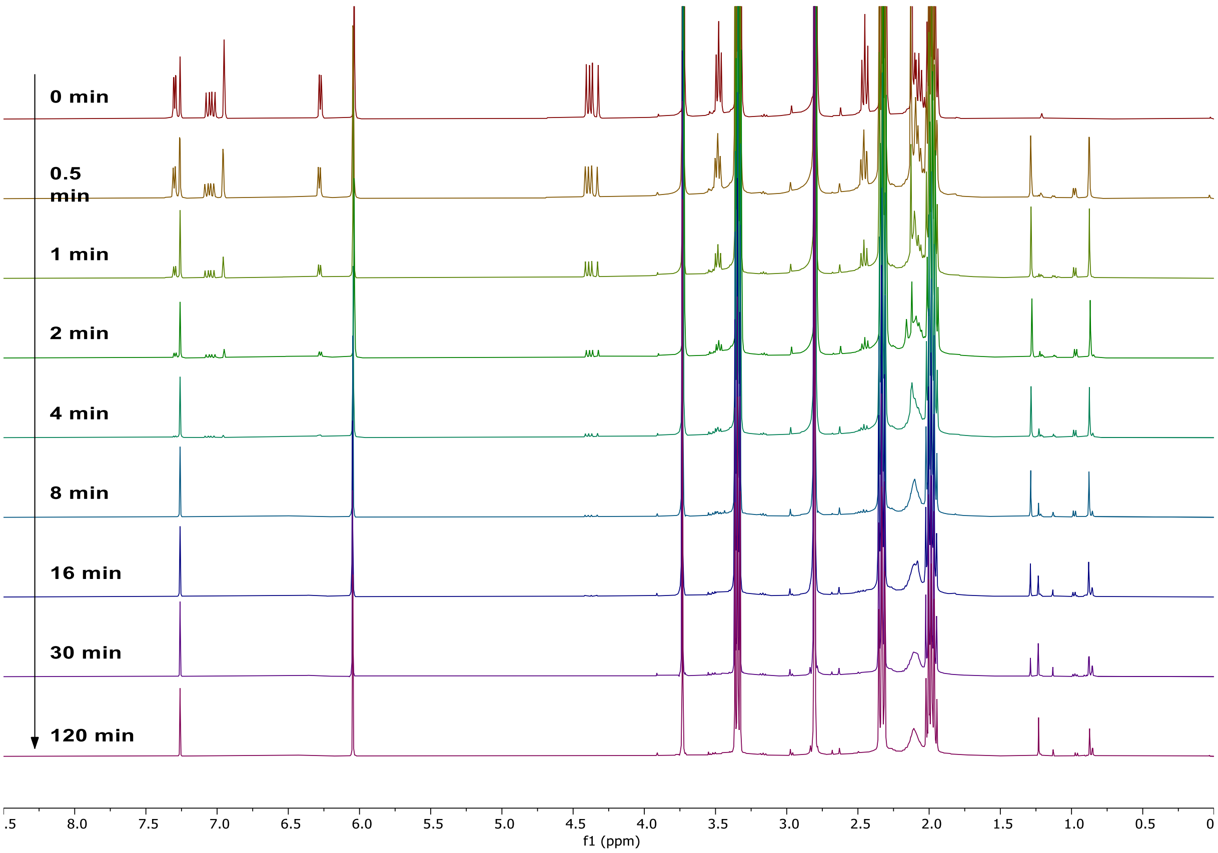

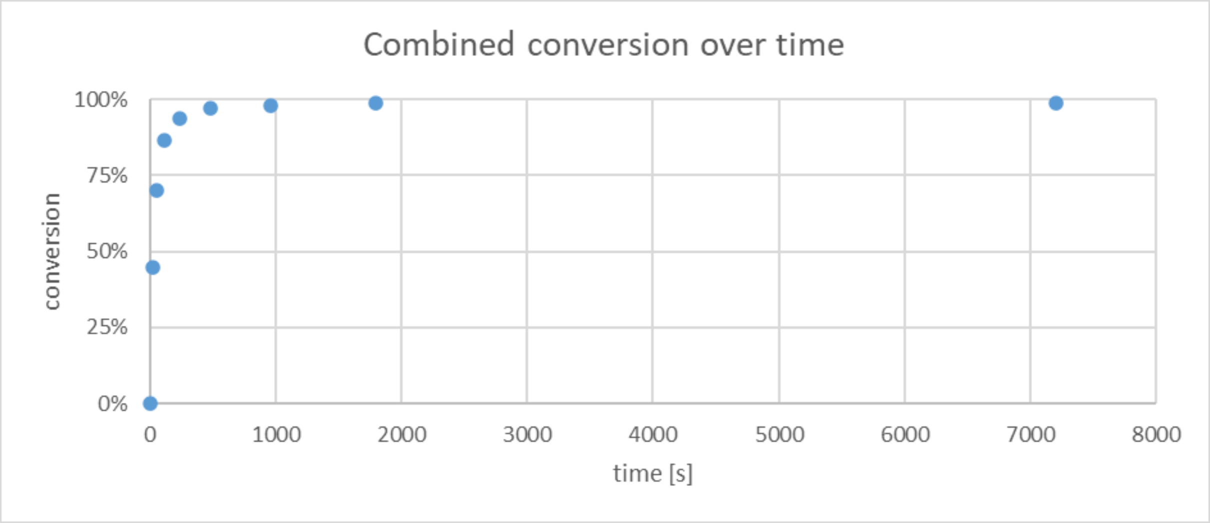
Combined conversion over time
conversion
100%
75%
50%
25%
0%
0
1000
2000
3000
4000
5000
6000
7000
8000
time [s]

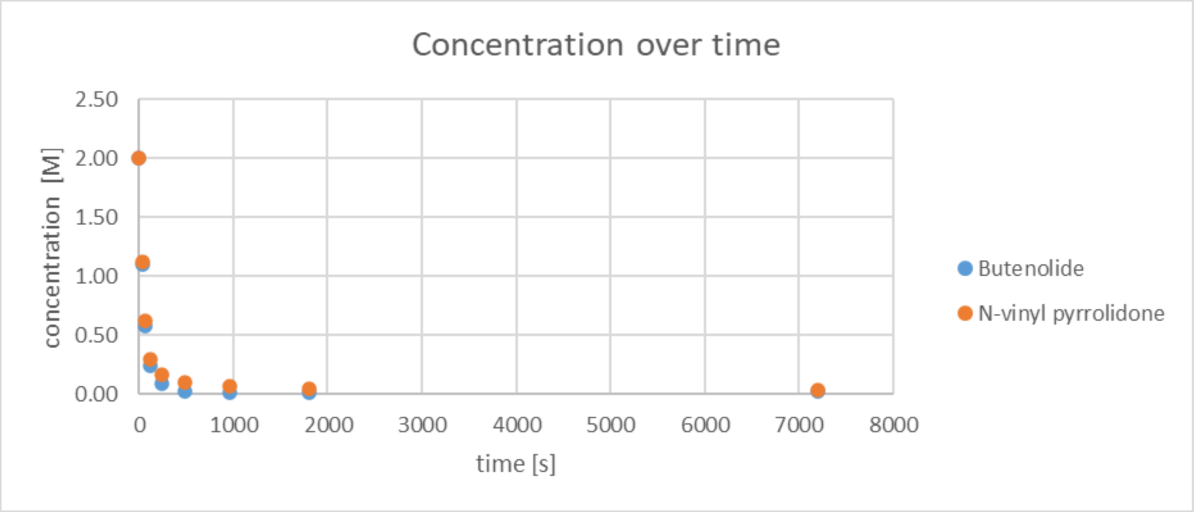
Concentration over time
concentration [M]
2.50
2.00
1.50
1.00
0.50
0.00
0
1000
2000
3000
4000
5000
6000
7000
8000
time [s]
Butenolide
N-vinyl pyrrolidone

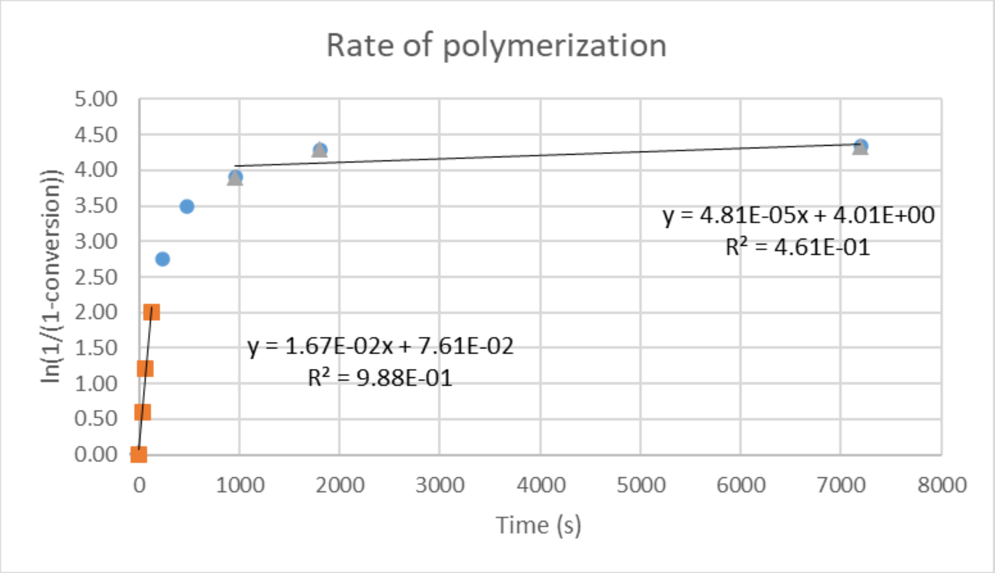
Rate of polymerization
ln(1/(1-conversion))
5.00
4.50
4.00
3.50
3.00
2.50
2.00
1.50
1.00
0.50
0.00
0
1000
2000
3000
4000
5000
6000
7000
8000
Time (s)
y = 4.81E-05x + 4.01E+00
R² = 4.61E-01
y = 1.67E-02x + 7.61E-02
R² = 9.88E-01

Acetoxy butenolide (2a) and NVP (3:1) in NMP

Poly(2a-co-NVP) (3:1)					k1(obs)	6.66×10^{-3} [s^{-1}]
	2a		N-vinyl pyrrolidone		k2(obs)	5.03×10^{-4} [s^{-1}]
Time [s]	Integral	Concentration [mol/L]	Integral	Concentration [mol/L]	Conversion	ln(1/[1-conversion])
0	1.41	3.00	0.50	1.00	0%	0.00
60	1.00	2.13	0.14	0.27	40%	0.51
120	0.85	1.81	0.05	0.09	52%	0.74
240	0.76	1.62	0.03	0.06	58%	0.87
480	0.66	1.41	0.04	0.09	63%	0.99
960	0.52	1.11	0.02	0.05	71%	1.24
1440	0.41	0.88	0.02	0.04	77%	1.47
2400	0.30	0.63	0.02	0.05	83%	1.78
3600	0.23	0.49	0.02	0.04	87%	2.02
5400	0.22	0.47	0.02	0.04	87%	2.07
7200	0.22	0.47	0.02	0.04	87%	2.05

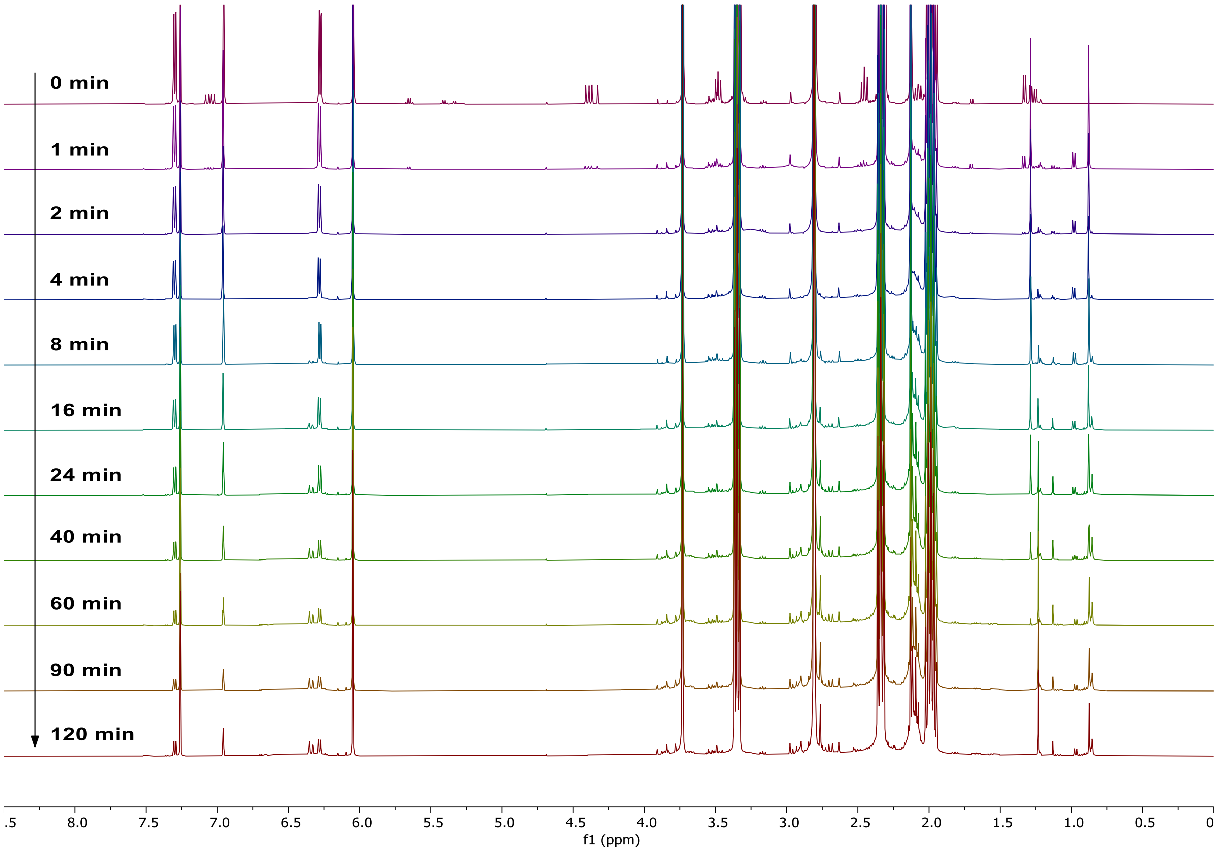

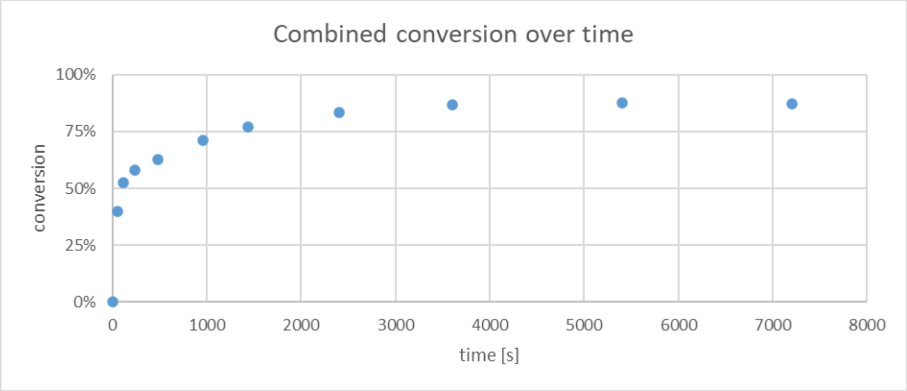
Combined conversion over time
conversion
100%
75%
50%
25%
0%
0
1000
2000
3000
4000
5000
6000
7000
8000
time [s]

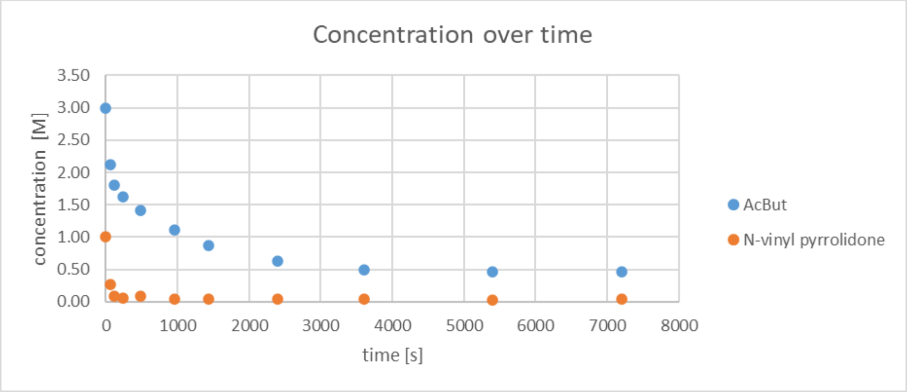
Concentration over time
concentration [M]
3.50
3.00
2.50
2.00
1.50
1.00
0.50
0.00
0
1000
2000
3000
4000
5000
6000
7000
8000
time [s]
AcBut
N-vinyl pyrrolidone

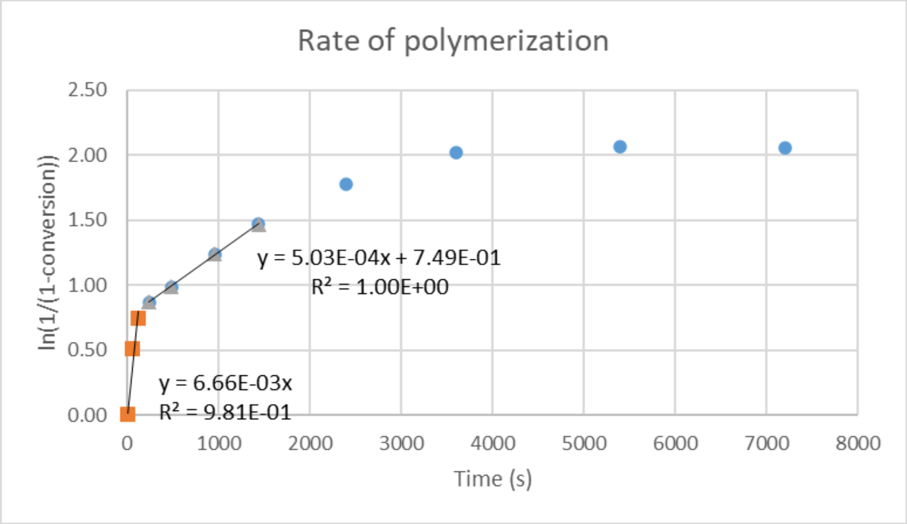
Rate of polymerization
ln(1/(1-conversion))
2.50
2.00
1.50
1.00
0.50
0.00
y = 5.03E-04x + 7.49E-01
R² = 1.00E+00
y = 6.66E-03x
R² = 9.81E-01
0
1000
2000
3000
4000
5000
6000
7000
8000
Time (s)

Acetoxy butenolide (2a) in NMP

Poly(2a)			k1(obs)	2.15×10^{-4} [s^{-1}]
	2a			
Time [s]	Integral	Concentration [mol/L]	Conversion	ln(1/[1-conversion])
0	1.85	4.00	0%	0.00
120	1.81	3.90	2%	0.02
300	1.74	3.74	6%	0.07
600	1.63	3.52	12%	0.13
1800	1.35	2.91	27%	0.32
3600	1.30	2.81	30%	0.35
7200	1.31	2.82	30%	0.35

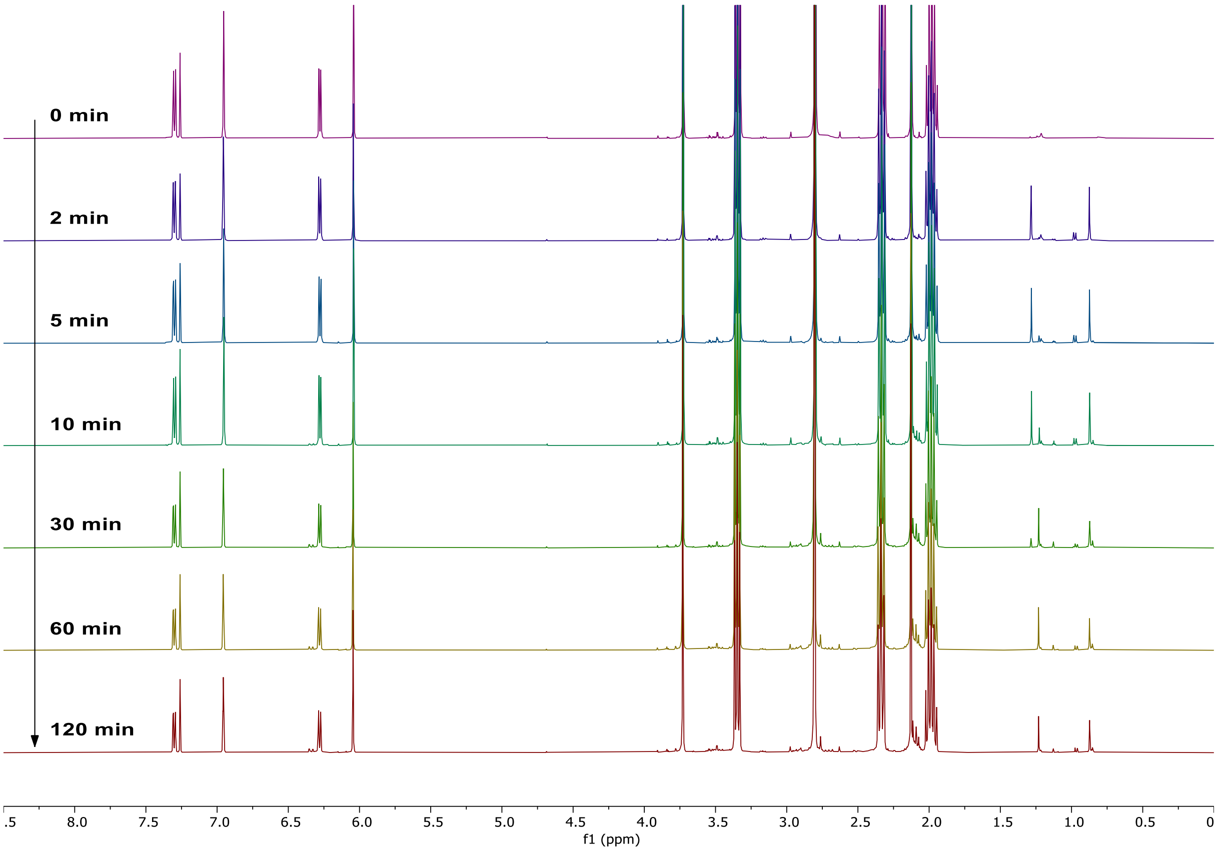

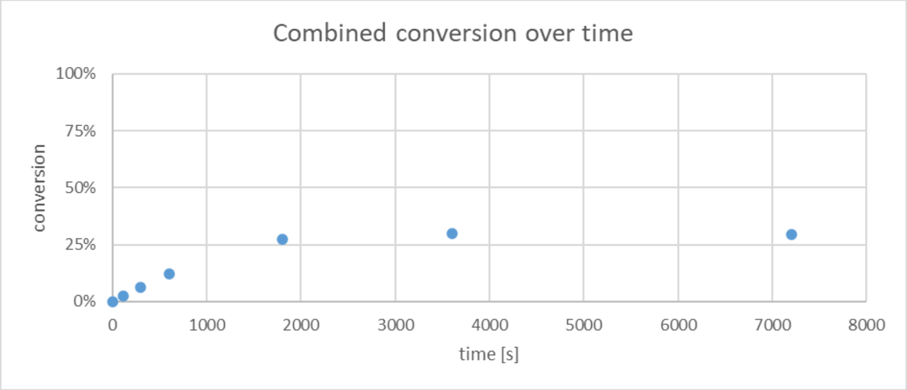
Combined conversion over time
100%
75%
50%
25%
0%
conversion
0
1000
2000
3000
4000
5000
6000
7000
8000
time [s]

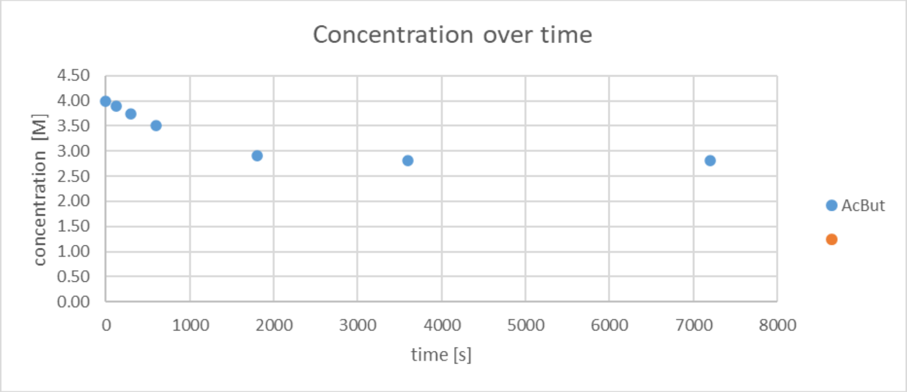
Concentration over time
4.50
4.00
3.50
3.00
2.50
2.00
1.50
1.00
0.50
0.00
concentration [M]
0
1000
2000
3000
4000
5000
6000
7000
8000
time [s]
AcBut

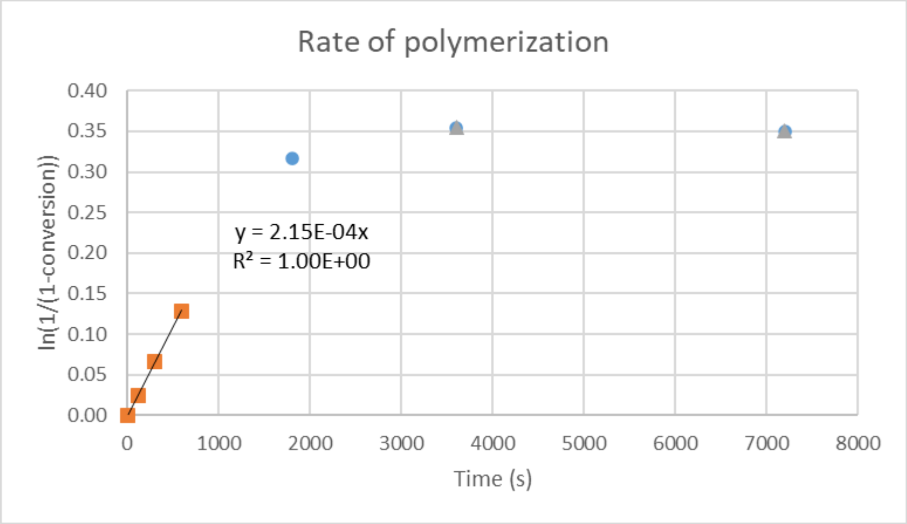
Rate of polymerization
0.40
0.35
0.30
0.25
0.20
0.15
0.10
0.05
0.00
ln(1/(1-conversion))
y = 2.15E-04x
R² = 1.00E+00
0
1000
2000
3000
4000
5000
6000
7000
8000
Time (s)

Acetoxy butenolide (2a) and VeoVa-10 in AcOBu

Poly(2a-co-VeoVa10)					k1(obs)	7.33×10^{-4} [s^{-1}]
	2a		VeoVa-10		k2(obs)	2.72×10^{-4} [s^{-1}]
Time [s]	Integral	Concentration [mol/L]	Integral	Concentration [mol/L]	Conversion	ln(1/[1-conversion])
0	0.97	2.00	1.04	2.00	0%	0.00
60	0.97	2.00	1.04	1.99	0%	0.00
120	0.96	1.98	1.04	1.99	1%	0.01
240	0.93	1.93	1.01	1.93	3%	0.04
480	0.83	1.73	0.87	1.66	15%	0.16
960	0.58	1.21	0.57	1.10	42%	0.55
1800	0.33	0.68	0.27	0.53	70%	1.20
3600	0.15	0.32	0.10	0.20	87%	2.05
7200	0.09	0.20	0.03	0.06	94%	2.75

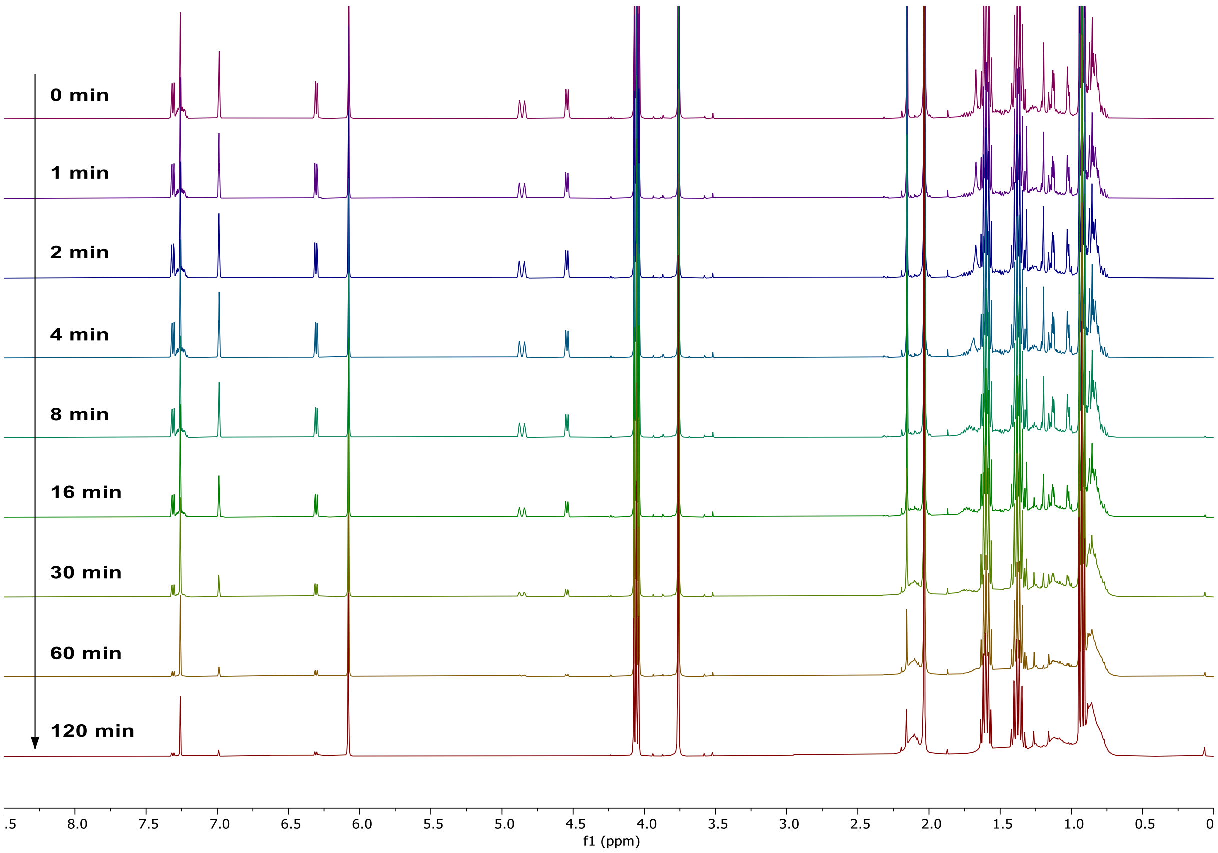

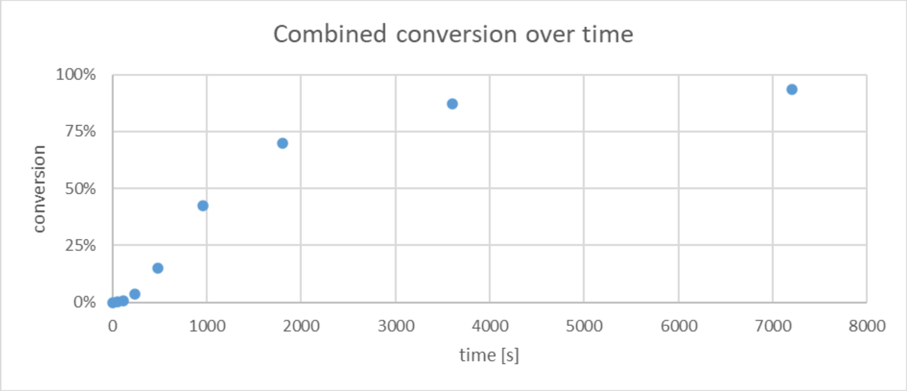
Combined conversion over time
conversion
100%
75%
50%
25%
0%
0
1000
2000
3000
4000
5000
6000
7000
8000
time [s]

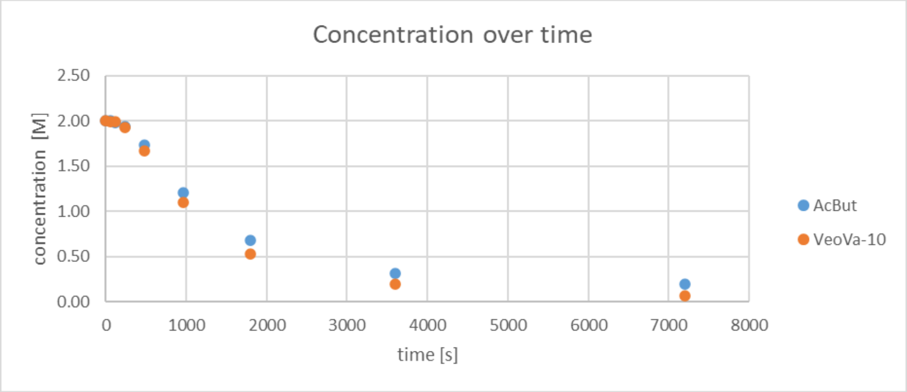
Concentration over time
concentration [M]
2.50
2.00
1.50
1.00
0.50
0.00
0
1000
2000
3000
4000
5000
6000
7000
8000
time [s]
AcBut
VeoVa-10

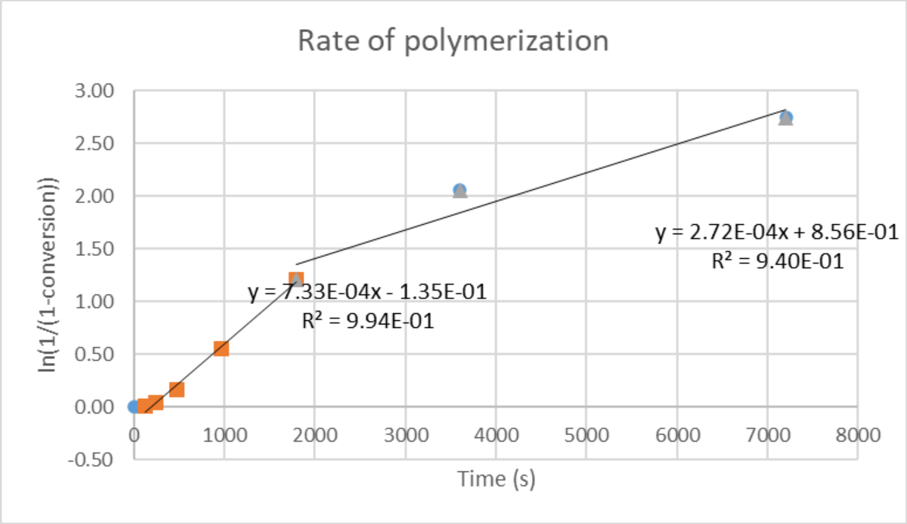
Rate of polymerization
ln(1/(1-conversion))
3.00
2.50
2.00
1.50
1.00
0.50
0.00
-0.50
0
1000
2000
3000
4000
5000
6000
7000
8000
Time (s)
y = 7.33E-04x - 1.35E-01
R² = 9.94E-01
y = 2.72E-04x + 8.56E-01
R² = 9.40E-01

Acetoxy butenolide (2a) and NVC in NMP

Poly(2a-co-NVC)					k1(obs)	4.85 x 10^{-3} [s^{-1}]
	2a		NVC		k2(obs)	1.74 x 10^{-5} [s^{-1}]
Time [s]	Integral	Concentration [mol/L]	Integral	Concentration [mol/L]	Conversion	ln(1/[1-conversion])
0	0.92	2.00	2.01	2.00	0%	0.00
60	0.70	1.54	1.53	1.52	24%	0.27
120	0.43	0.94	1.01	1.01	51%	0.72
240	0.23	0.50	0.55	0.55	74%	1.34
480	0.07	0.15	0.25	0.25	90%	2.31
960	0.03	0.06	0.17	0.17	94%	2.89
1800	0.03	0.06	0.17	0.17	94%	2.86
3600	0.03	0.06	0.15	0.15	95%	2.95
7200	0.03	0.06	0.14	0.14	95%	2.98

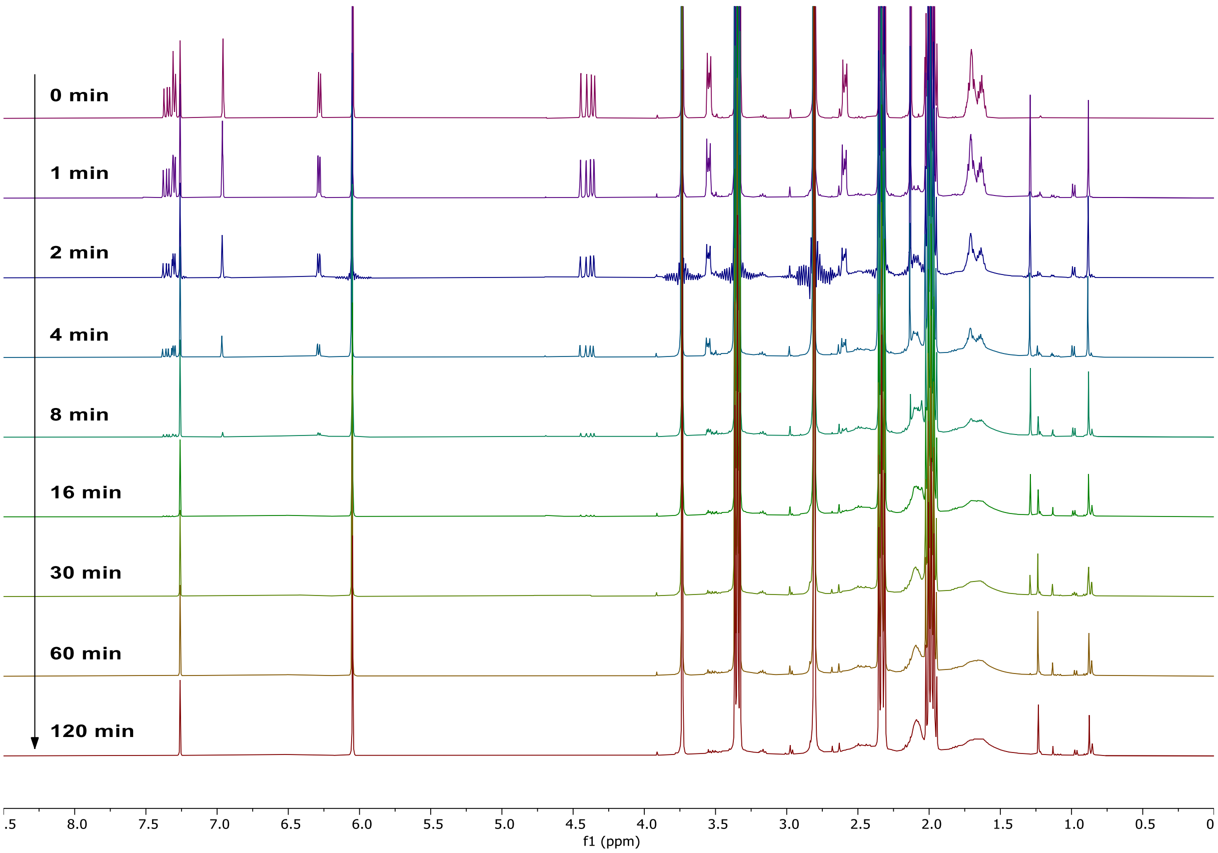

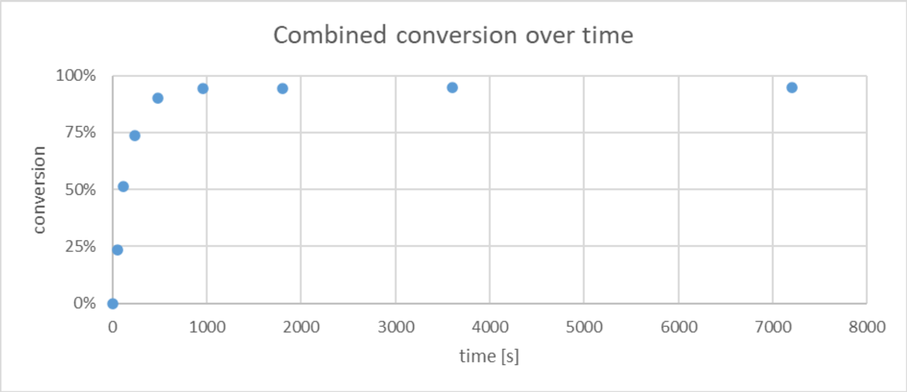
Combined conversion over time
100%
75%
50%
25%
0%
conversion
0
1000
2000
3000
4000
5000
6000
7000
8000
time [s]

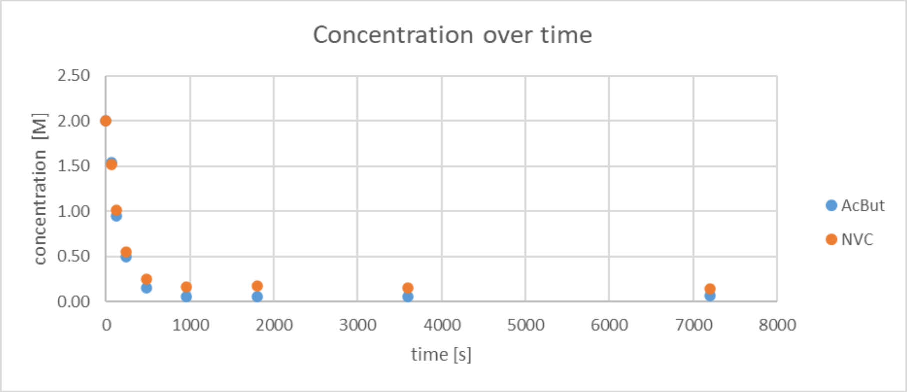
Concentration over time
2.50
2.00
1.50
1.00
0.50
0.00
concentration [M]
0
1000
2000
3000
4000
5000
6000
7000
8000
time [s]
AcBut
NVC

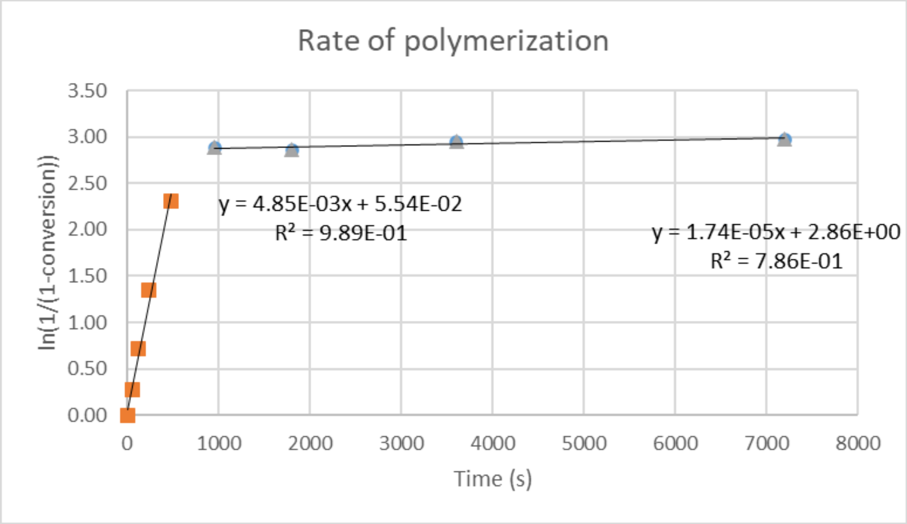
Rate of polymerization
3.50
3.00
2.50
2.00
1.50
1.00
0.50
0.00
ln(1/(1-conversion))
y = 4.85E-03x + 5.54E-02
R² = 9.89E-01
y = 1.74E-05x + 2.86E+00
R² = 7.86E-01
0
1000
2000
3000
4000
5000
6000
7000
8000
Time (s)

Acetoxy butenolide (2a) and butyl acrylate in AcOBu

Poly(2a-co-Butyl acrylate)					k1(obs)	1.14×10^{-3} [s^{-1}]
	2a		Butyl acrylate		k2(obs)	4.49×10^{-5} [s^{-1}]
Time [s]	Integral	Concentration [mol/L]	Integral	Concentration [mol/L]	Conversion	ln(1/[1-conversion])
0	0.93	2.00	0.99	2.00	0%	0.00
60	0.93	1.99	0.97	1.97	1%	0.01
120	0.90	1.94	0.91	1.83	6%	0.06
240	0.88	1.89	0.59	1.19	23%	0.26
480	0.88	1.89	0.29	0.59	38%	0.48
1020	0.82	1.76	0.07	0.13	53%	0.75
1800	0.78	1.68	0.02	0.05	57%	0.84
3600	0.71	1.54	0.01	0.01	61%	0.95
7200	0.65	1.40	0.00	0.00	65%	1.05

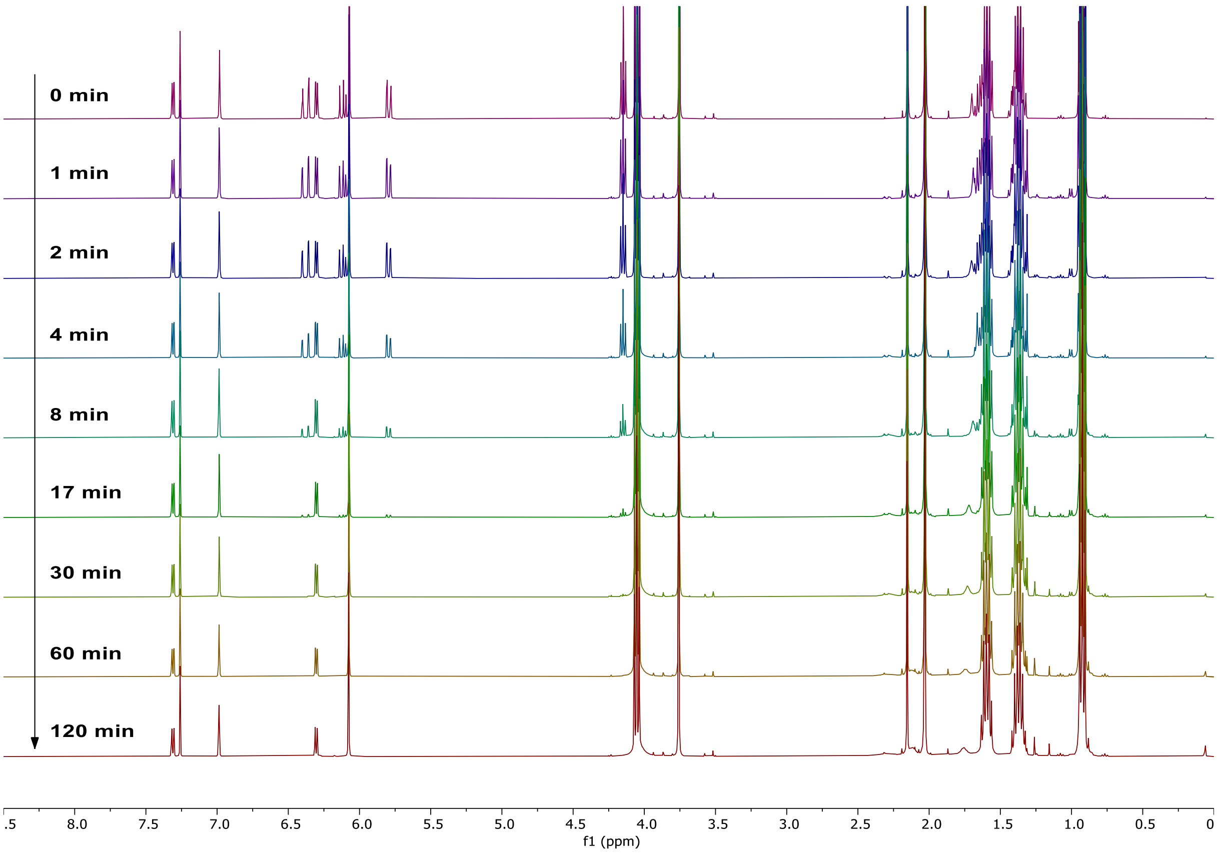

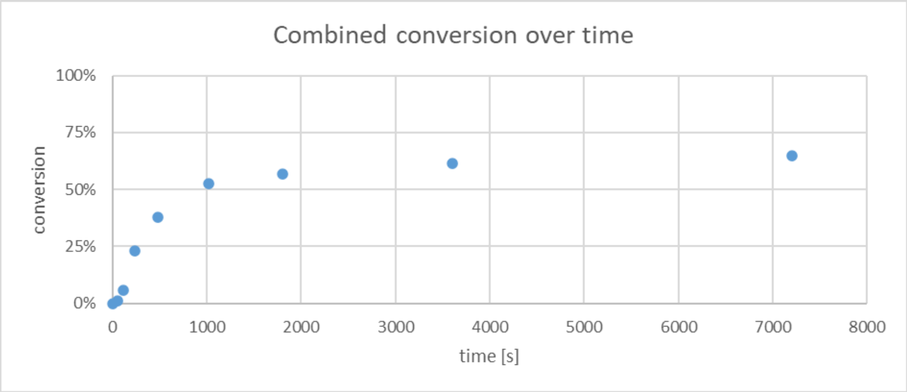
Combined conversion over time
100%
75%
50%
25%
0%
conversion
0
1000
2000
3000
4000
5000
6000
7000
8000
time [s]

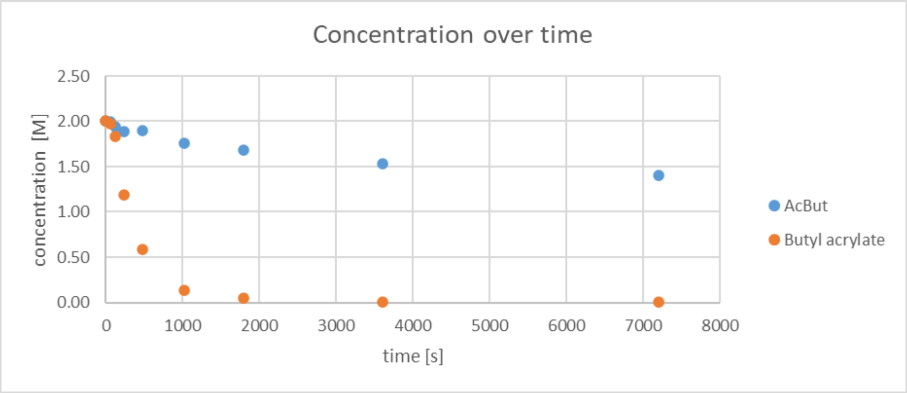
Concentration over time
2.50
2.00
1.50
1.00
0.50
0.00
concentration [M]
0
1000
2000
3000
4000
5000
6000
7000
8000
time [s]
AcBut
Butyl acrylate

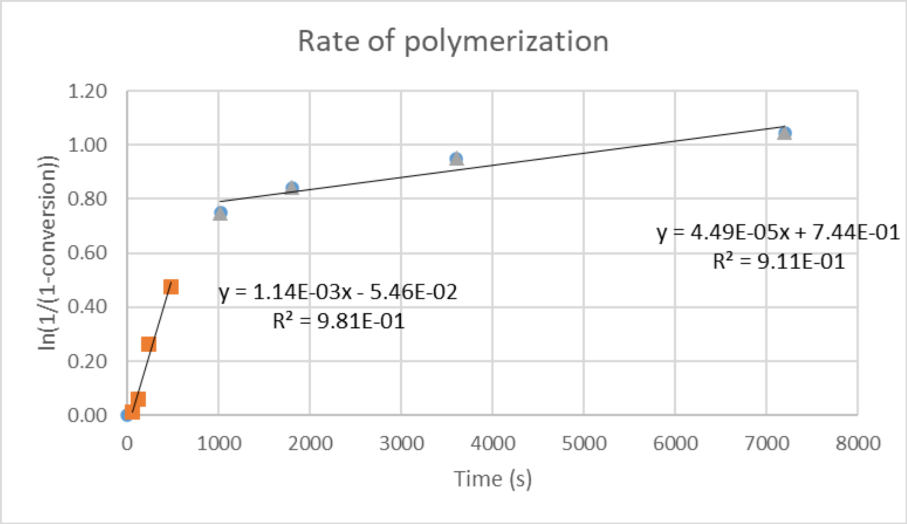
Rate of polymerization
1.20
1.00
0.80
0.60
0.40
0.20
0.00
ln(1/(1-conversion))
y = 4.49E-05x + 7.44E-01
R² = 9.11E-01
y = 1.14E-03x - 5.46E-02
R² = 9.81E-01
0
1000
2000
3000
4000
5000
6000
7000
8000
Time (s)

Acetoxy butenolide (2a) and butyl methacrylate in AcOBu

Poly(2a-co-Butyl methacrylate)					k1(obs)	3.94 x 10^{-4} [s^{-1}]
	2a		Butyl methacrylate		k2(obs)	4.65 x 10^{-5} [s^{-1}]
Time [s]	Integral	Concentration [mol/L]	Integral	Concentration [mol/L]	Conversion	ln(1/[1-conversion])
0	0.91	2.00	1.01	2.00	0%	0.00
60	0.92	2.00	0.99	1.95	1%	0.01
120	0.91	1.98	0.96	1.89	3%	0.03
240	0.91	1.98	0.88	1.74	7%	0.07
480	0.90	1.96	0.70	1.38	17%	0.18
1080	0.89	1.95	0.36	0.70	34%	0.41
1800	0.85	1.86	0.20	0.39	44%	0.58
3600	0.83	1.82	0.08	0.15	51%	0.71
7200	0.77	1.68	0.03	0.05	57%	0.84

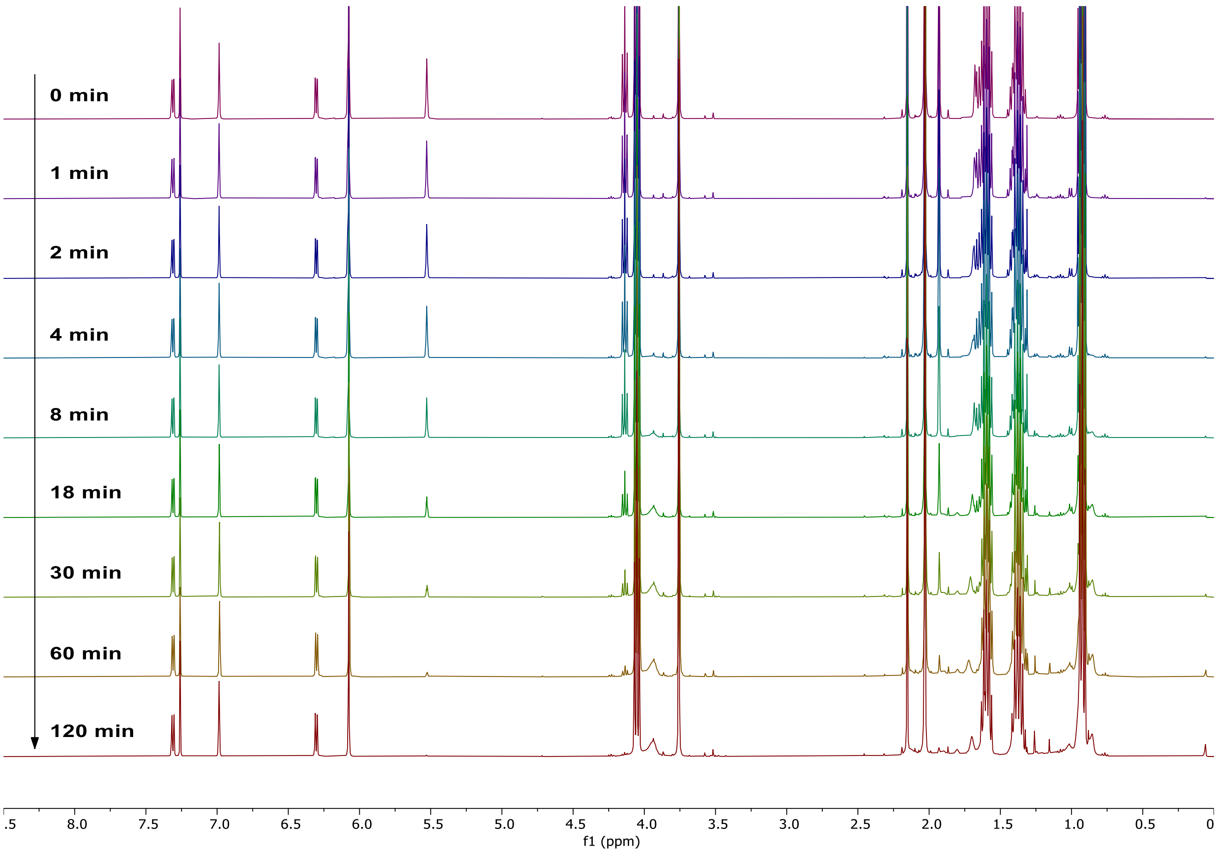

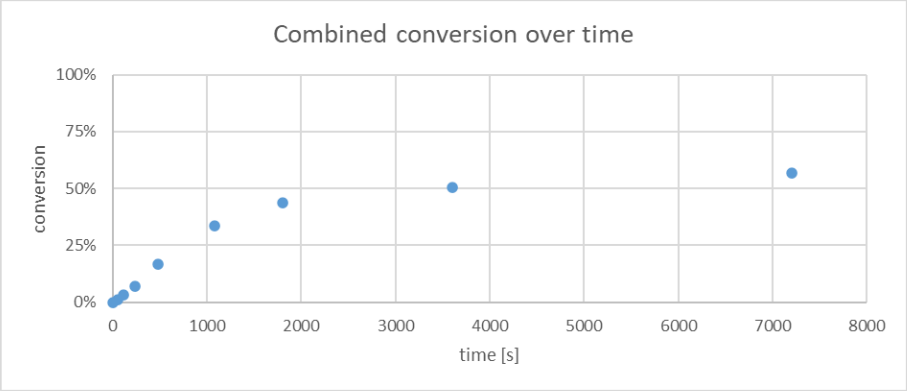
Combined conversion over time
conversion
100%
75%
50%
25%
0%
0
1000
2000
3000
4000
5000
6000
7000
8000
time [s]

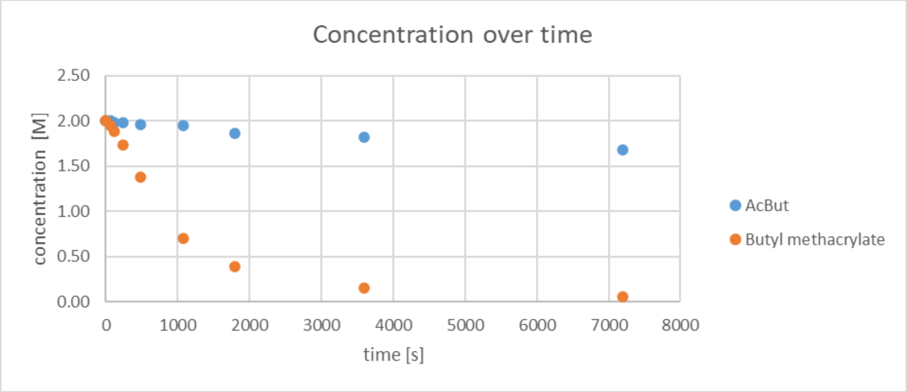
Concentration over time
concentration [M]
2.50
2.00
1.50
1.00
0.50
0.00
0
1000
2000
3000
4000
5000
6000
7000
8000
time [s]
AcBut
Butyl methacrylate

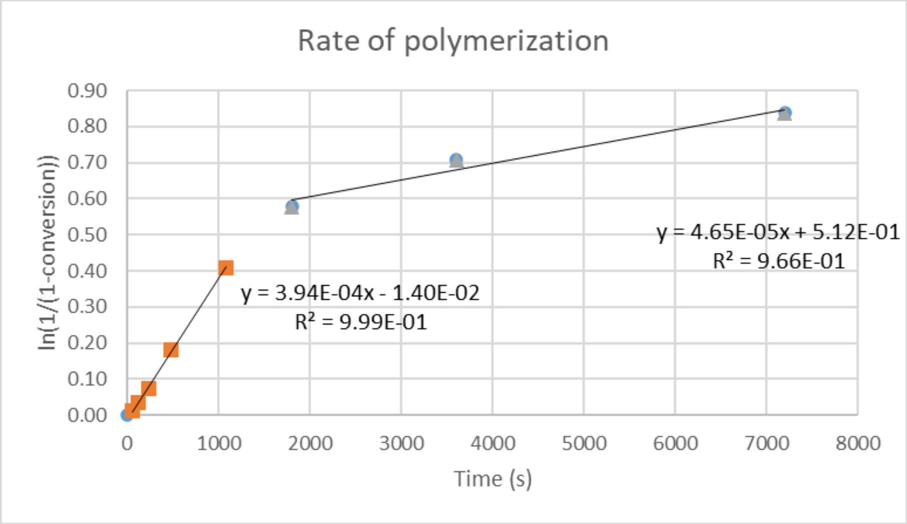
Rate of polymerization
ln(1/(1-conversion))
0.90
0.80
0.70
0.60
0.50
0.40
0.30
0.20
0.10
0.00
0
1000
2000
3000
4000
5000
6000
7000
8000
Time (s)
y = 4.65E-05x + 5.12E-01
R² = 9.66E-01
y = 3.94E-04x - 1.40E-02
R² = 9.99E-01

Acetoxy butenolide (2a) and styrene in AcOBu

Poly(2a-co-St)					k_{obs} (2nd order)	3.37 x 10^{-4} [s^{-1}]
	2a		Styrene			
Time [s]	Integral	Concentration [mol/L]	Integral	Concentration [mol/L]	Conversion	ln(1/[1-conversion])
0	0.98	2.00	1.01	2.00	0%	0.00
60	0.98	1.99	0.95	1.88	3%	0.03
120	0.98	1.98	0.92	1.81	5%	0.05
240	0.97	1.98	0.83	1.63	10%	0.10
480	0.97	1.97	0.71	1.41	16%	0.17
720	0.96	1.94	0.61	1.21	21%	0.24
1080	0.95	1.92	0.50	0.99	27%	0.32
1440	0.93	1.88	0.40	0.79	33%	0.40
2160	0.90	1.84	0.26	0.51	41%	0.53
3120	0.87	1.77	0.14	0.29	49%	0.67
7200	0.78	1.59	0.03	0.05	59%	0.89

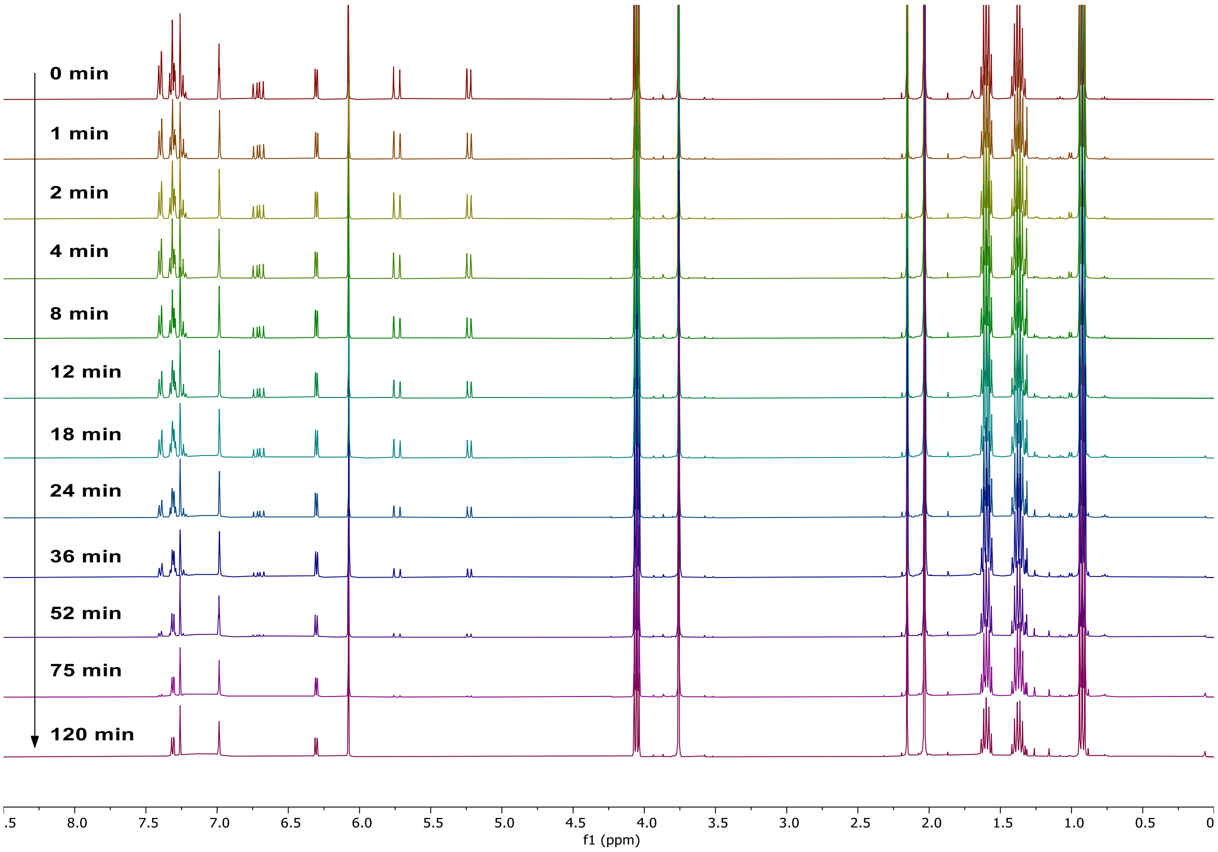

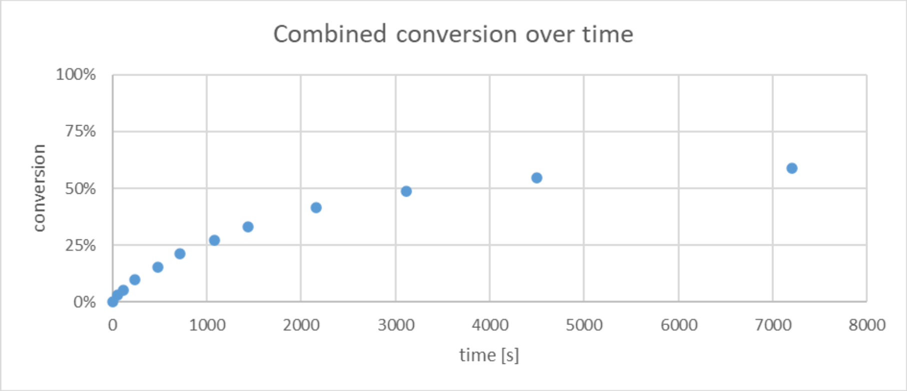
Combined conversion over time
conversion
100%
75%
50%
25%
0%
0
1000
2000
3000
4000
5000
6000
7000
8000
time [s]

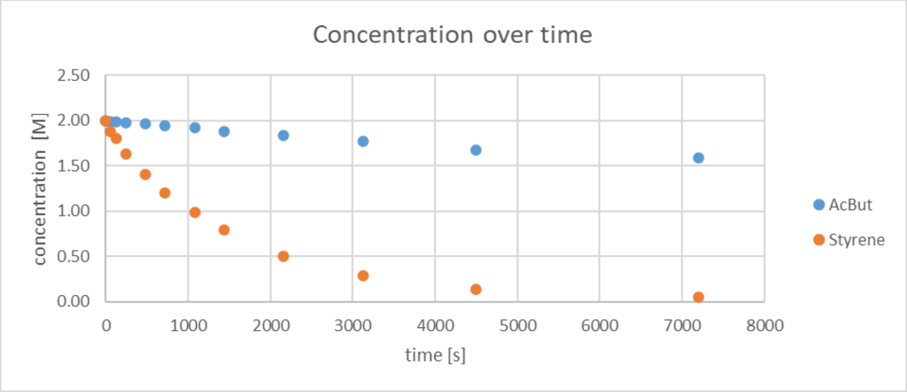
Concentration over time
concentration [M]
2.50
2.00
1.50
1.00
0.50
0.00
0
1000
2000
3000
4000
5000
6000
7000
8000
time [s]
AcBut
Styrene

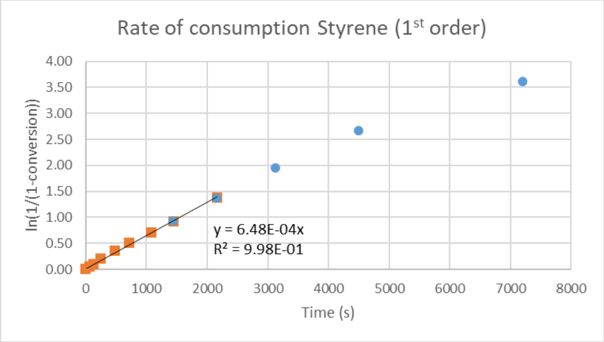
Rate of consumption Styrene (1st order)
ln(1/(1-conversion))
4.00
3.50
3.00
2.50
2.00
1.50
1.00
0.50
0.00
0
1000
2000
3000
4000
5000
6000
7000
8000
Time (s)
y = 6.48E-04x
R² = 9.98E-01

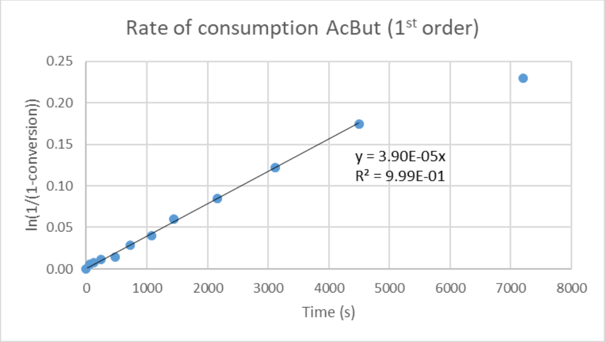
Rate of consumption AcBut (1st order)
ln(1/(1-conversion))
0.25
0.20
0.15
0.10
0.05
0.00
0
1000
2000
3000
4000
5000
6000
7000
8000
Time (s)
y = 3.90E-05x
R² = 9.99E-01

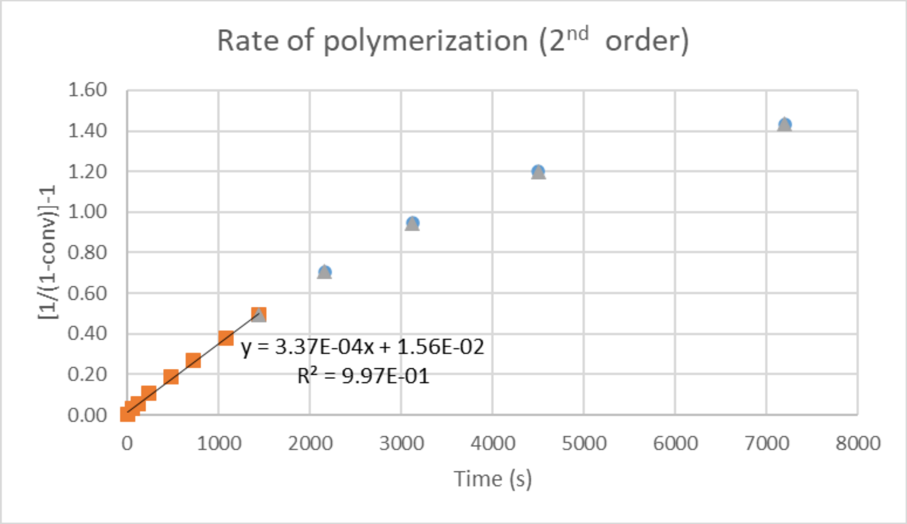
Rate of polymerization (2nd order)
[1/(1-conv)]-1
1.60
1.40
1.20
1.00
0.80
0.60
0.40
0.20
0.00
0
1000
2000
3000
4000
5000
6000
7000
8000
Time (s)
y = 3.37E-04x + 1.56E-02
R² = 9.97E-01

Butyl acrylate and DVE in AcOBu

Poly(butyl acrylate-co-DVE) in AcOBu					k1(obs)	4.73 x 10^{-3} [s^{-1}]
	Butyl acrylate		Dodecyl vinyl ether		k2(obs)	1.31 x 10^{-4} [s^{-1}]
Time [s]	Integral	Concentration [mol/L]	Integral	Concentration [mol/L]	Conversion	ln(1/[1-conversion])
0	0.98	2.00	0.91	2.00	0%	0.00
60	0.87	1.77	0.89	1.96	7%	0.07
120	0.34	0.69	0.74	1.62	42%	0.55
240	0.03	0.07	0.60	1.31	66%	1.07
480	0.00	0.00	0.55	1.21	70%	1.19
960	0.00	0.01	0.51	1.11	72%	1.28
2100	0.01	0.01	0.42	0.93	77%	1.45
3600	0.00	0.01	0.36	0.79	80%	1.61
7200	0.00	0.01	0.31	0.68	83%	1.77

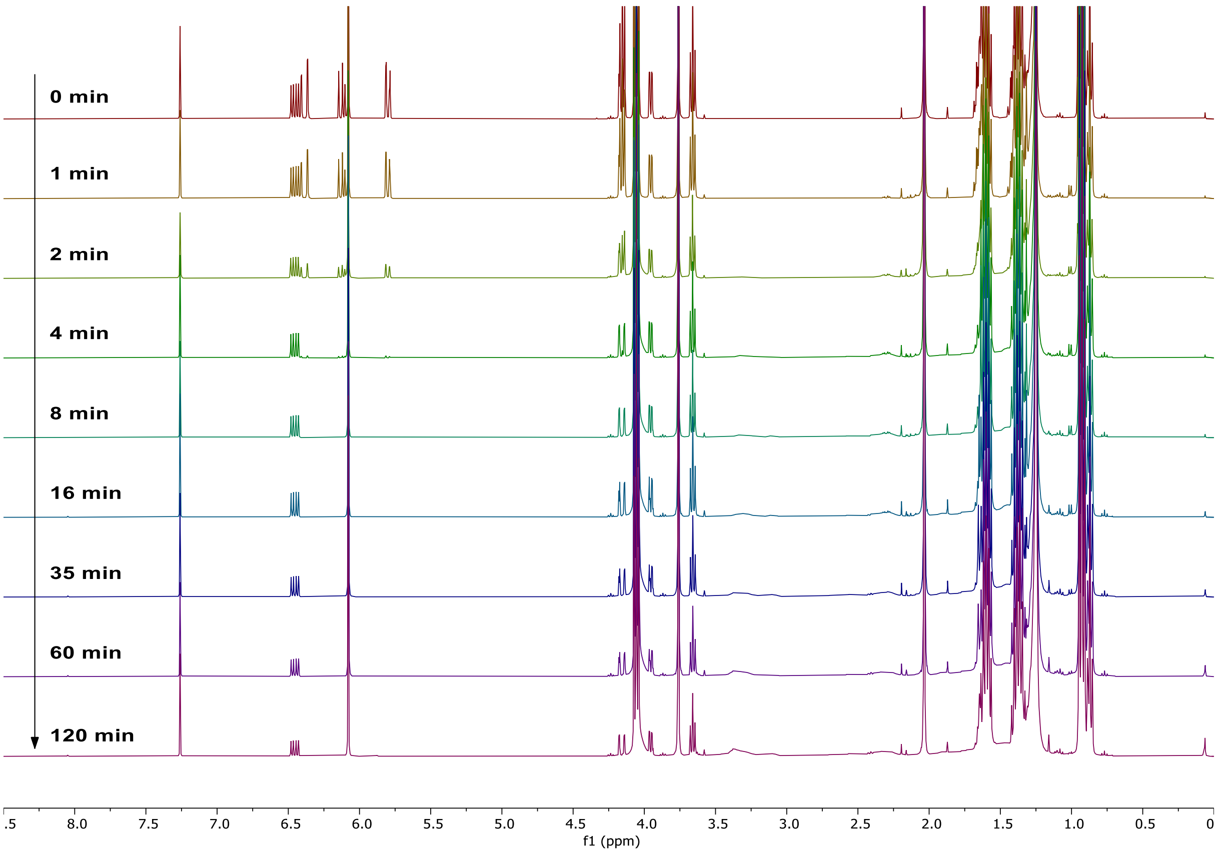

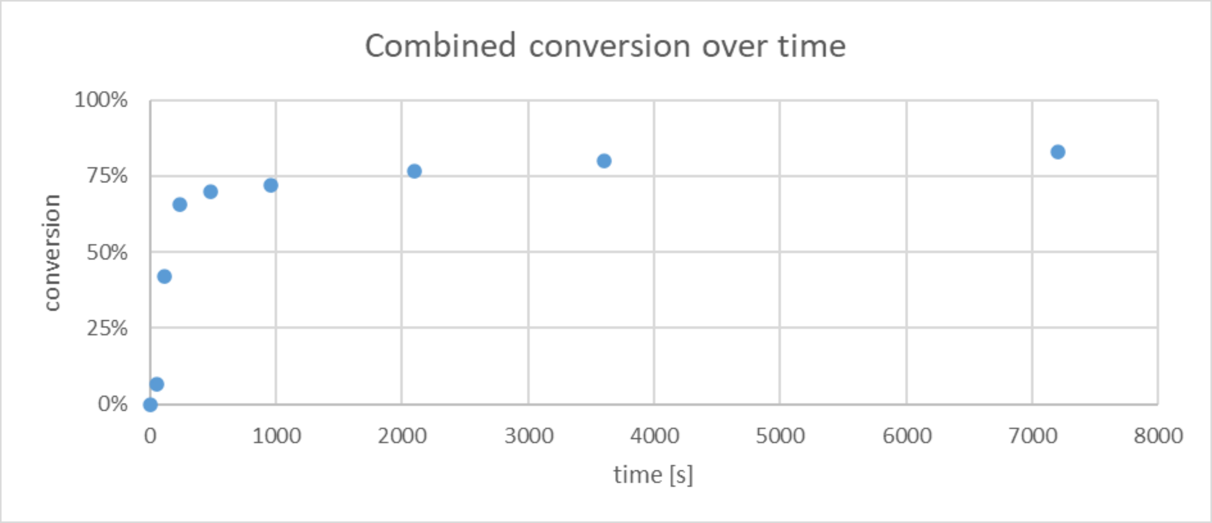
Combined conversion over time
conversion
100%
75%
50%
25%
0%
0
1000
2000
3000
4000
5000
6000
7000
8000
time [s]

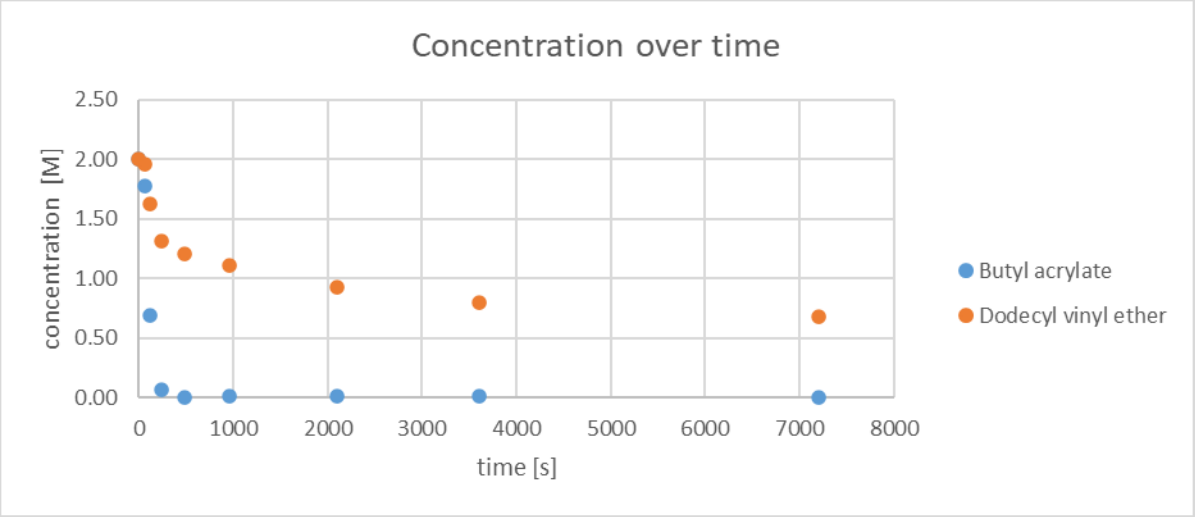
Concentration over time
concentration [M]
2.50
2.00
1.50
1.00
0.50
0.00
0
1000
2000
3000
4000
5000
6000
7000
8000
time [s]
Butyl acrylate
Dodecyl vinyl ether

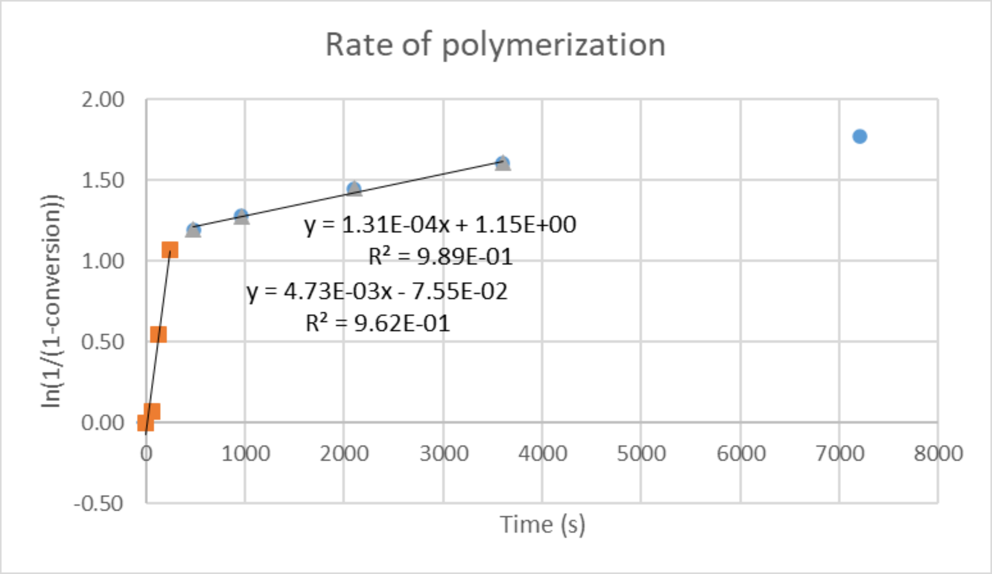
Rate of polymerization
ln(1/(1-conversion))
2.00
1.50
1.00
0.50
0.00
-0.50
0
1000
2000
3000
4000
5000
6000
7000
8000
Time (s)
y = 1.31E-04x + 1.15E+00
R² = 9.89E-01
y = 4.73E-03x - 7.55E-02
R² = 9.62E-01

Furanone and DVE in AcOBu

Poly(Furanone-co-DVE) in AcOBu					k1(obs)	2.01×10^{-4} [s^{-1}]
	2(5H)-Furanone		Dodecyl vinyl ether		k2(obs)	5.50×10^{-5} [s^{-1}]
Time [s]	Integral	Concentration [mol/L]	Integral	Concentration [mol/L]	Conversion	ln(1/[1-conversion])
0	0.98	2.00	1.00	2.00	0%	0.00
60	0.97	1.97	0.94	1.88	4%	0.04
120	0.96	1.94	0.94	1.87	5%	0.05
240	0.95	1.93	0.90	1.80	7%	0.07
480	0.93	1.88	0.85	1.71	10%	0.11
960	0.86	1.76	0.84	1.69	14%	0.15
1800	0.79	1.60	0.78	1.57	21%	0.23
7200	0.58	1.18	0.63	1.27	39%	0.49

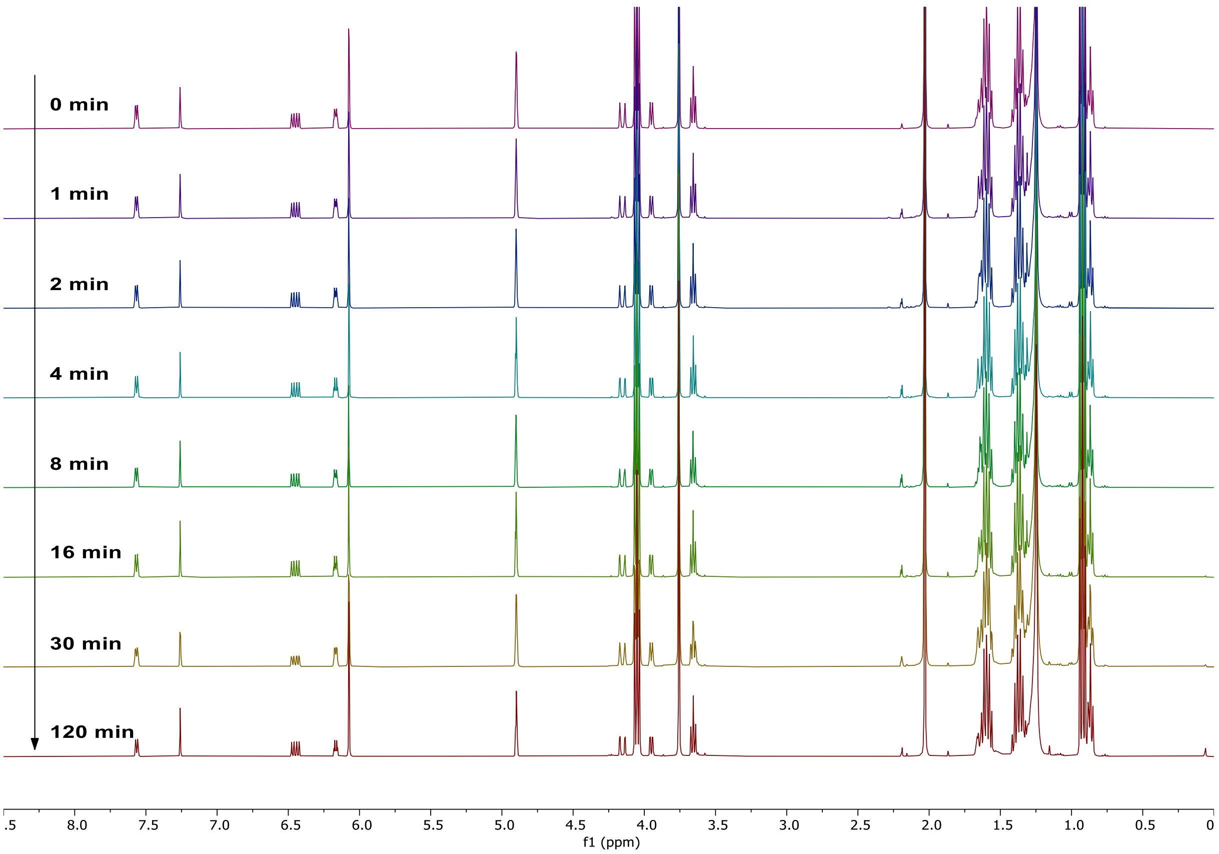

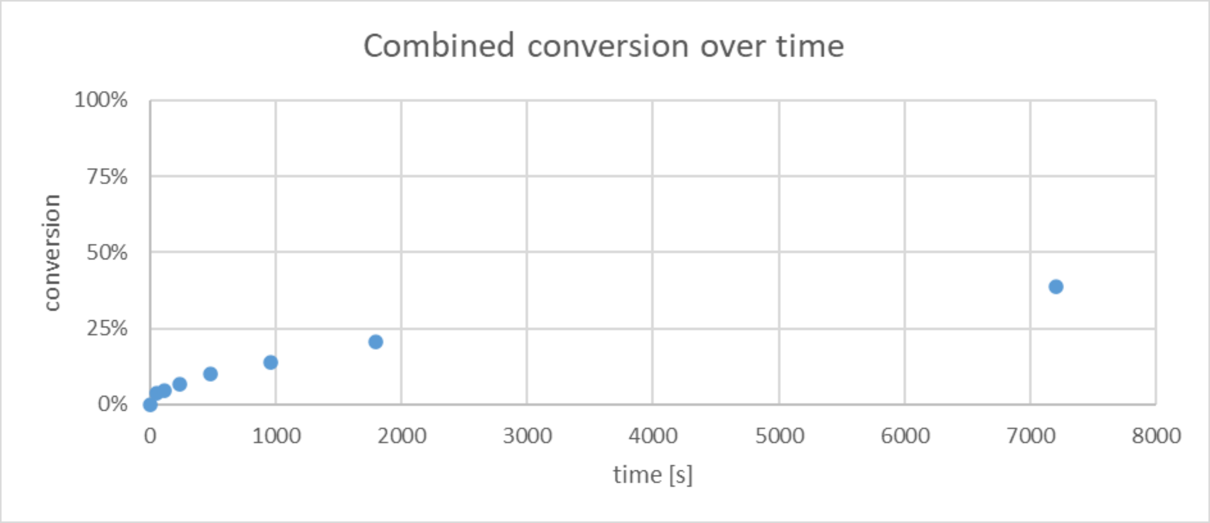
Combined conversion over time
100%
75%
50%
25%
0%
conversion
0
1000
2000
3000
4000
5000
6000
7000
8000
time [s]

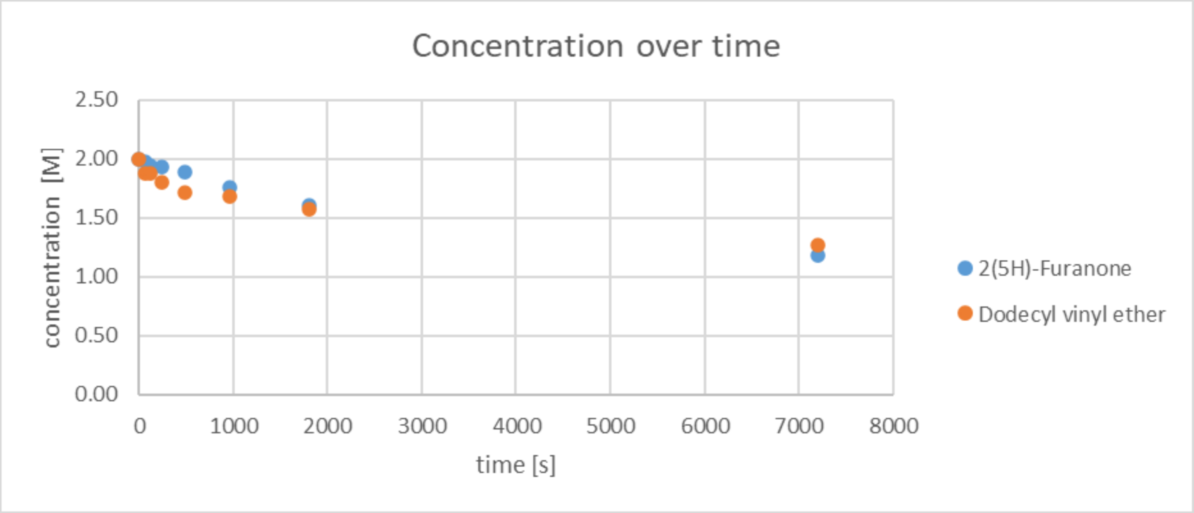
Concentration over time
2.50
2.00
1.50
1.00
0.50
0.00
concentration [M]
0
1000
2000
3000
4000
5000
6000
7000
8000
time [s]
2(5H)-Furanone
Dodecyl vinyl ether

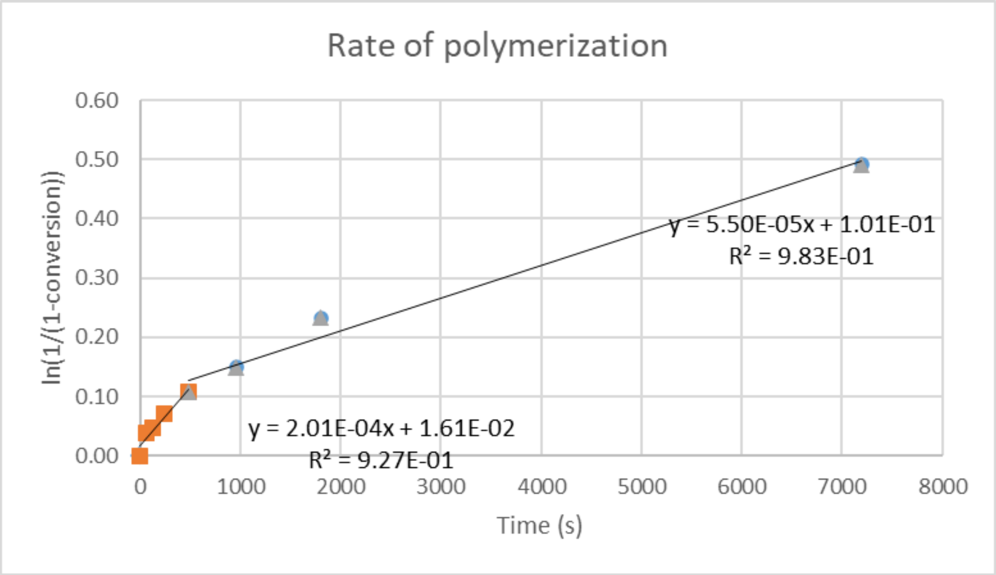
Rate of polymerization
0.60
0.50
0.40
0.30
0.20
0.10
0.00
ln(1/(1-conversion))
y = 5.50E-05x + 1.01E-01
R² = 9.83E-01
y = 2.01E-04x + 1.61E-02
R² = 9.27E-01
0
1000
2000
3000
4000
5000
6000
7000
8000
Time (s)

Methyl furanone and DVE in AcOBu

Poly(MeFuranone-co-DVE) in AcOBu					k1(obs)	7.80×10^{-5} [s^{-1}]
	5-Methyl-2(5H)-furanone		Dodecyl vinyl ether		k2(obs)	2.56×10^{-5} [s^{-1}]
Time [s]	Integral	Concentration [mol/L]	Integral	Concentration [mol/L]	Conversion	ln(1/[1-conversion])
0	0.86	2.00	0.90	2.00	0%	0.00
60	0.86	1.99	0.90	2.00	0%	0.00
120	0.87	2.02	0.90	2.00	-1%	-0.01
240	0.86	2.00	0.88	1.95	1%	0.01
480	0.85	1.97	0.87	1.92	3%	0.03
960	0.81	1.88	0.83	1.84	7%	0.07
1800	0.77	1.80	0.80	1.77	11%	0.11
3600	0.71	1.65	0.75	1.66	17%	0.19
7200	0.66	1.53	0.71	1.56	23%	0.26

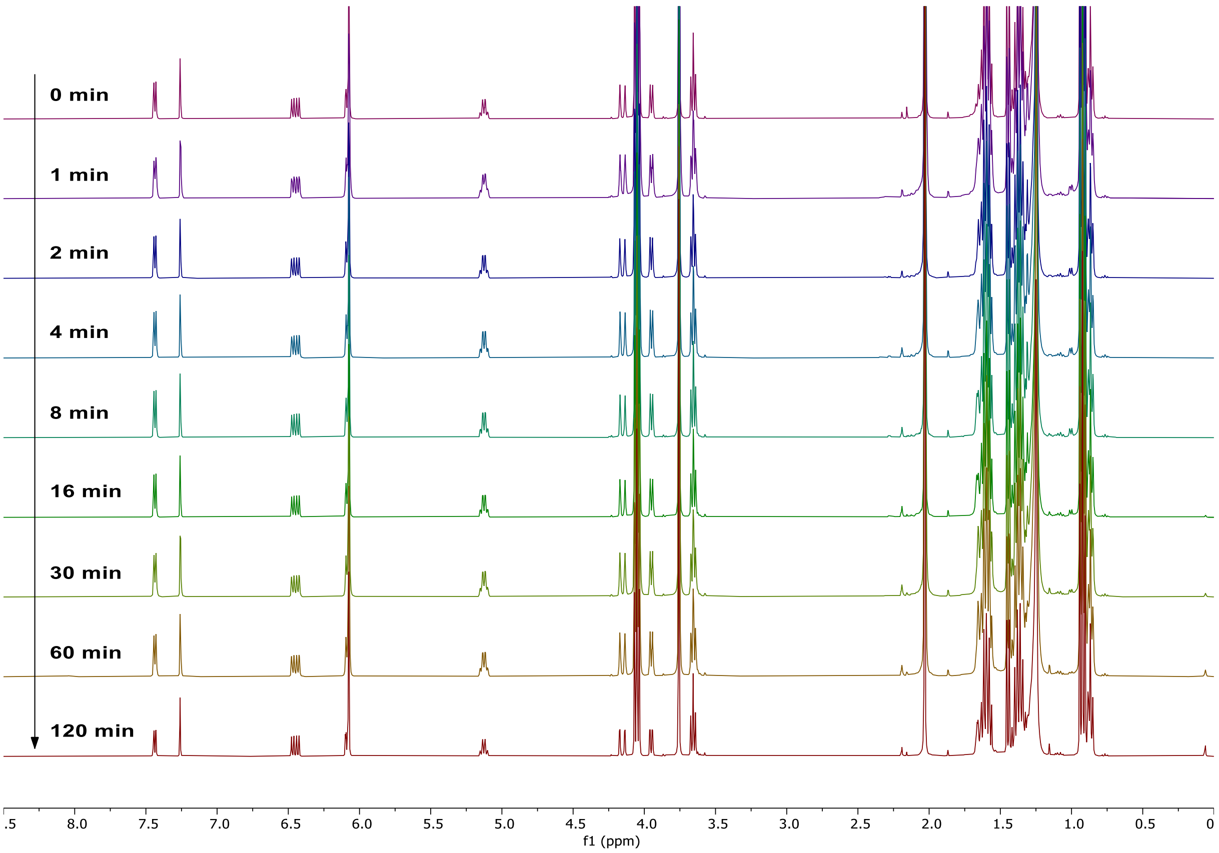

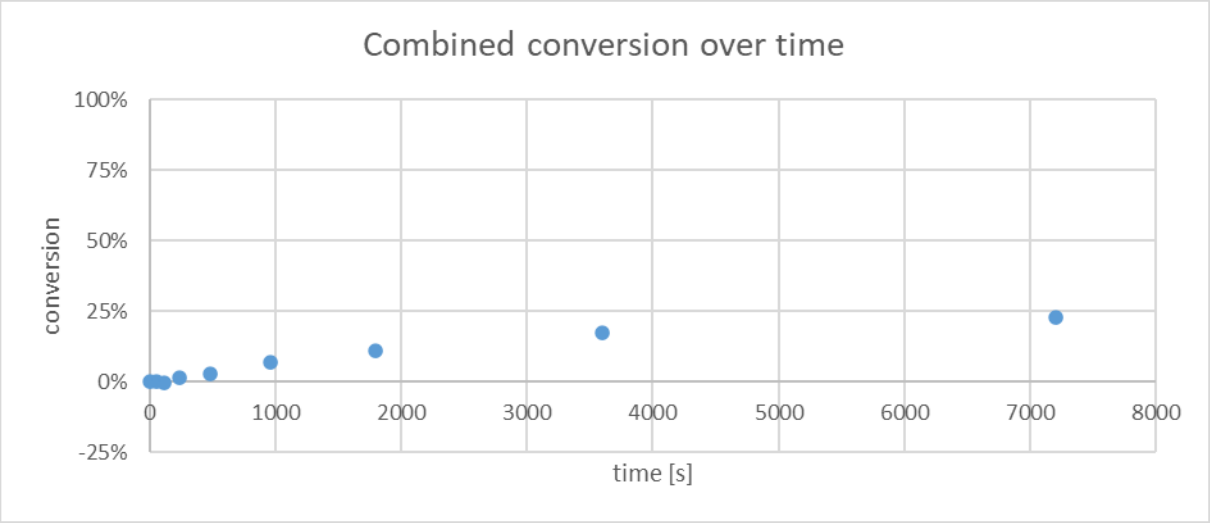
Combined conversion over time
conversion
100%
75%
50%
25%
0%
-25%
0
1000
2000
3000
4000
5000
6000
7000
8000
time [s]

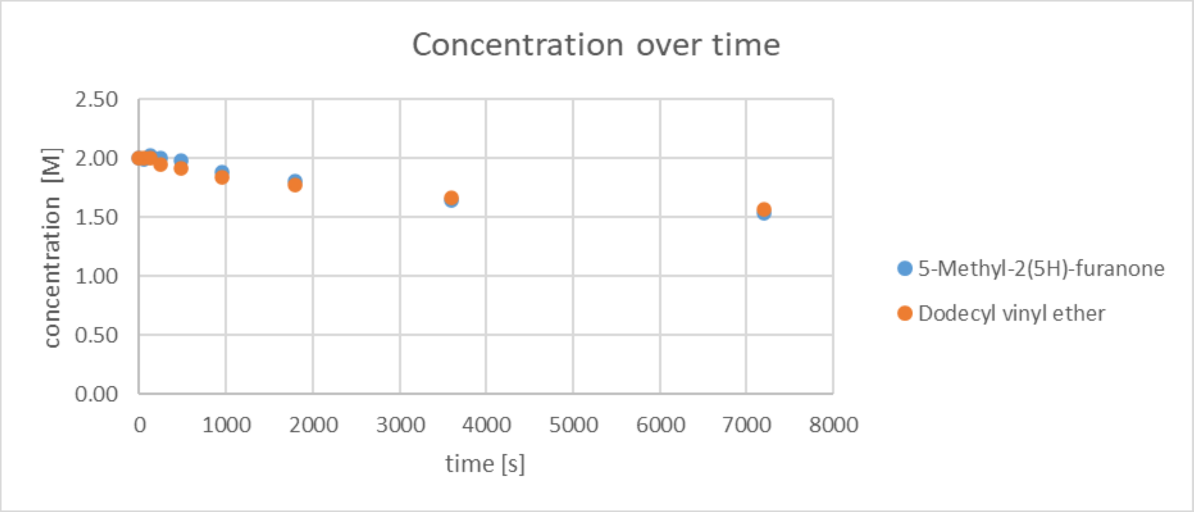
Concentration over time
concentration [M]
2.50
2.00
1.50
1.00
0.50
0.00
0
1000
2000
3000
4000
5000
6000
7000
8000
time [s]
5-Methyl-2(5H)-furanone
Dodecyl vinyl ether

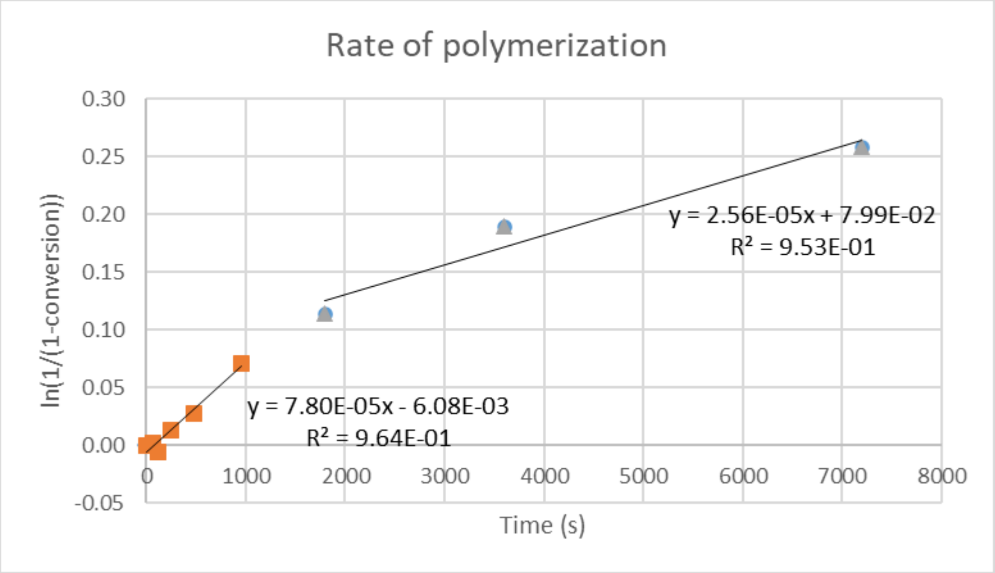
Rate of polymerization
ln(1/(1-conversion))
0.30
0.25
0.20
0.15
0.10
0.05
0.00
-0.05
y = 2.56E-05x + 7.99E-02
R² = 9.53E-01
y = 7.80E-05x - 6.08E-03
R² = 9.64E-01
0
1000
2000
3000
4000
5000
6000
7000
8000
Time (s)

Methoxy methyl butenolide and DVE in AcOBu

Poly(Methyl methoxy butenolide-co-DVE) in AcOBu					k1(obs)	2.04 x 10^{-4} [s^{-1}]
	Methyl methoxy butenolide		Dodecyl vinyl ether		k2(obs)	n/a
Time [s]	Integral	Concentration [mol/L]	Integral	Concentration [mol/L]	Conversion	ln(1/[1-conversion])
0	0.94	2.00	0.97	2.00	0%	0.00
60	0.92	1.96	0.86	1.79	6%	0.06
120	0.93	1.99	0.86	1.77	6%	0.06
240	0.93	1.98	0.83	1.72	8%	0.08
480	0.91	1.93	0.80	1.66	10%	0.11
960	0.81	1.72	0.72	1.50	20%	0.22
1800	0.66	1.41	0.62	1.28	33%	0.40
7200	0.35	0.74	0.39	0.80	61%	0.95

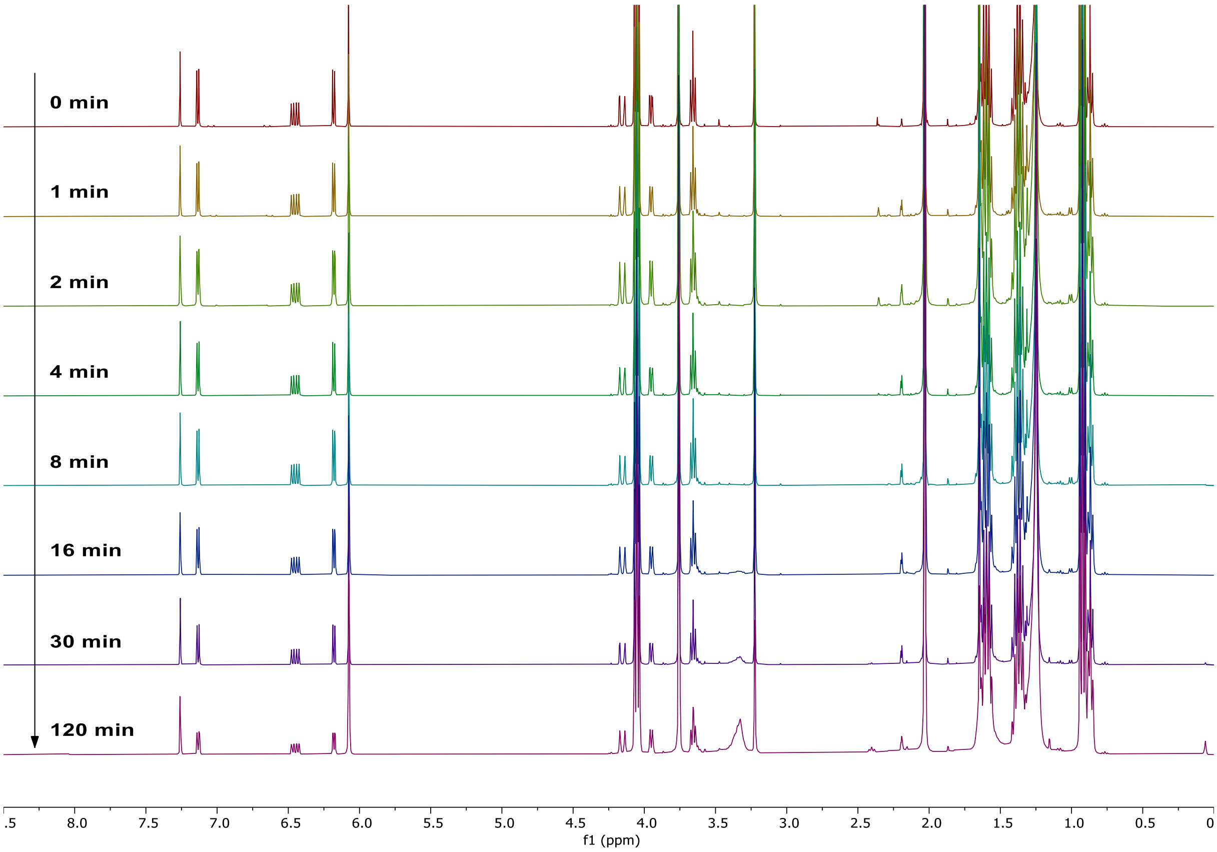

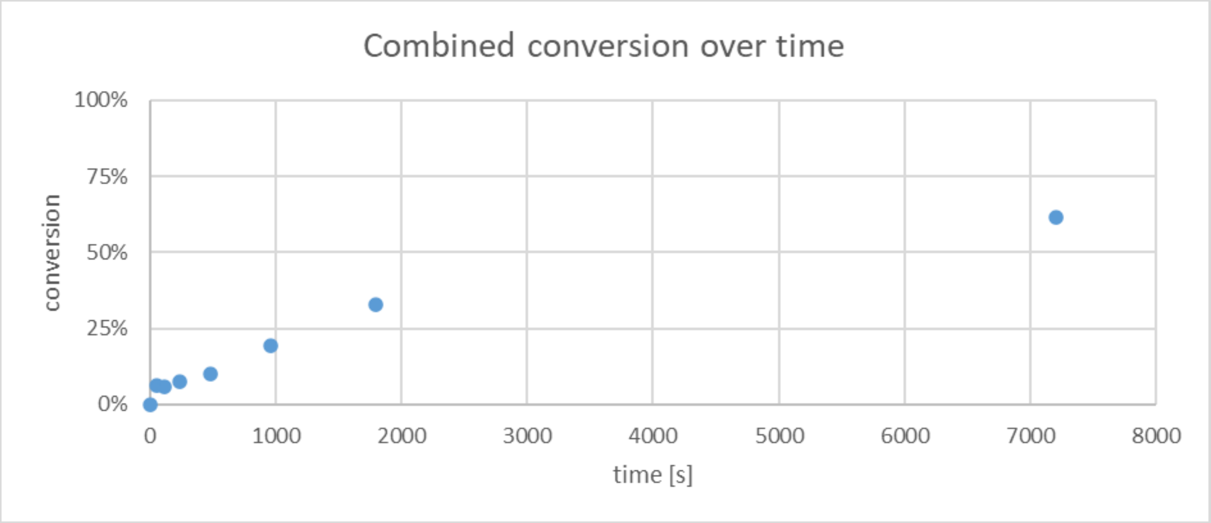
Combined conversion over time
conversion
100%
75%
50%
25%
0%
0
1000
2000
3000
4000
5000
6000
7000
8000
time [s]

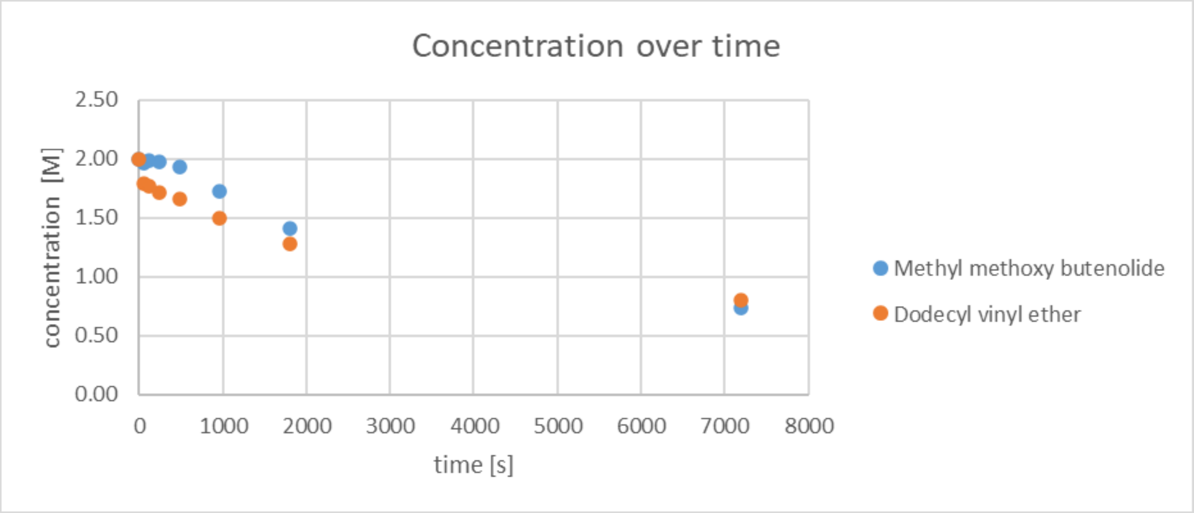
Concentration over time
concentration [M]
2.50
2.00
1.50
1.00
0.50
0.00
0
1000
2000
3000
4000
5000
6000
7000
8000
time [s]
Methyl methoxy butenolide
Dodecyl vinyl ether

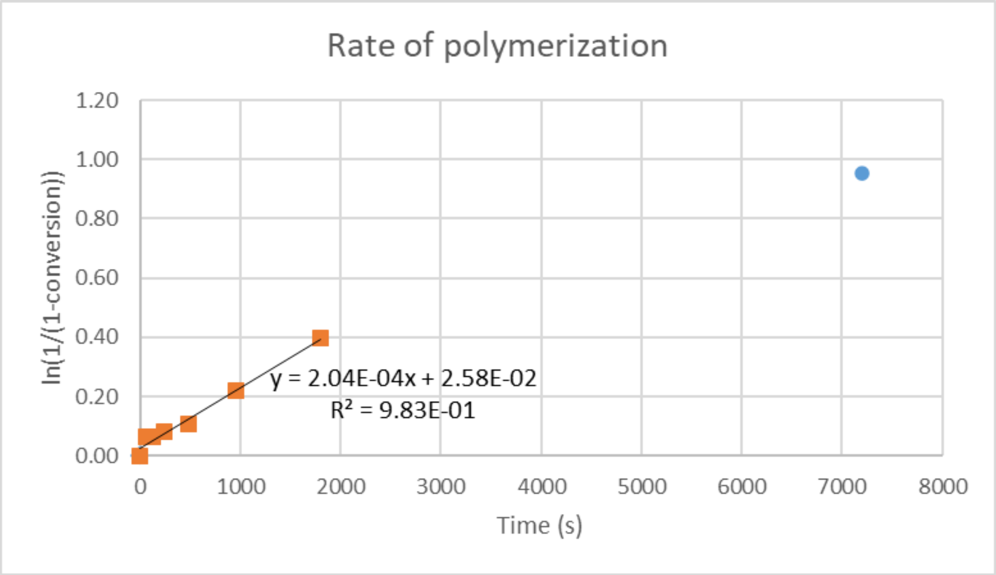
Rate of polymerization
ln(1/(1-conversion))
1.20
1.00
0.80
0.60
0.40
0.20
0.00
0
1000
2000
3000
4000
5000
6000
7000
8000
Time (s)
y = 2.04E-04x + 2.58E-02
R² = 9.83E-01

Maleic anhydride and DVE in AcOBu

Poly(MA-co-DVE) in AcOBu					k1(obs)	> 5.26 x 10^{-2} [s^{-1}]
	Maleic anhydride		Dodecyl vinyl ether		k2(obs)	3.21 x 10^{-3} [s^{-1}]
Time [s]	Integral	Concentration [mol/L]	Integral	Concentration [mol/L]	Conversion	ln(1/[1-conversion])
0	1.84	2.00	0.92	2.00	0%	0.00
60	0.00	0.00	0.08	0.17	96%	3.16
120	0.00	0.00	0.06	0.14	96%	3.35
240	0.00	0.00	0.04	0.09	98%	3.81
480	0.00	0.00	0.02	0.04	99%	4.50
960	0.00	0.00	0.00	0.00	100%	9.82
1800	0.00	0.00	0.00	0.00	100%	10.52
7200	0.00	0.00	0.00	0.00	100%	7.63

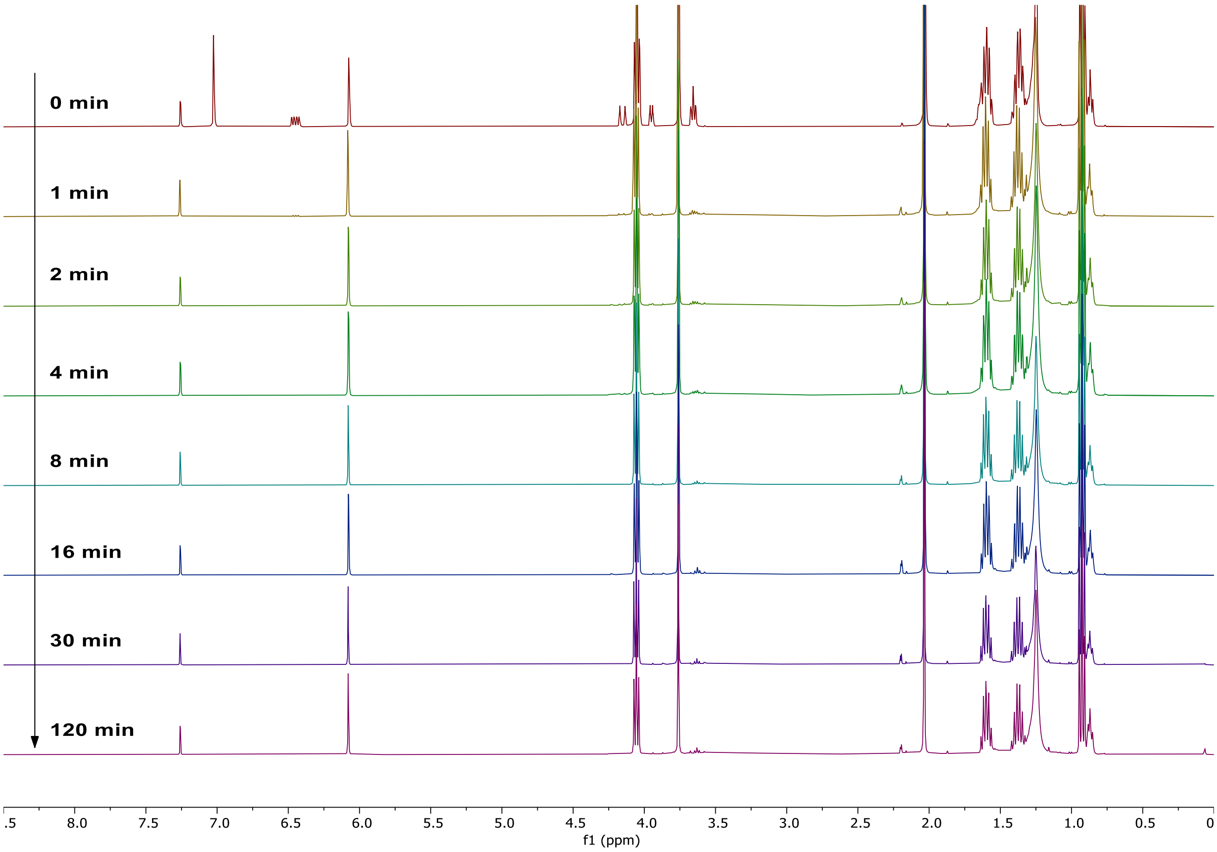

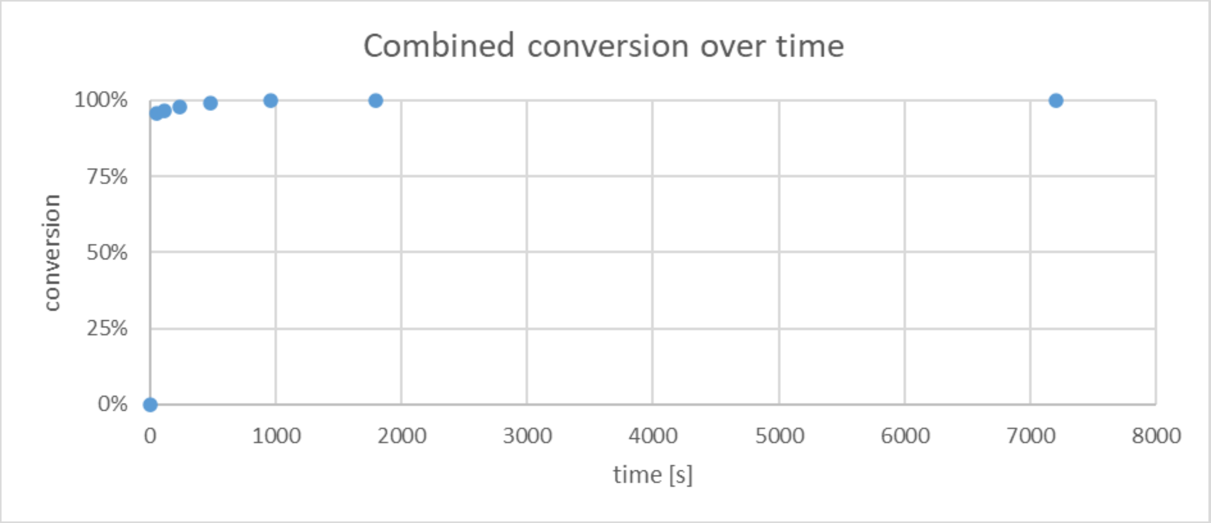

Combined conversion over time
conversion
100%
75%
50%
25%
0%
0
1000
2000
3000
4000
5000
6000
7000
8000
time [s]

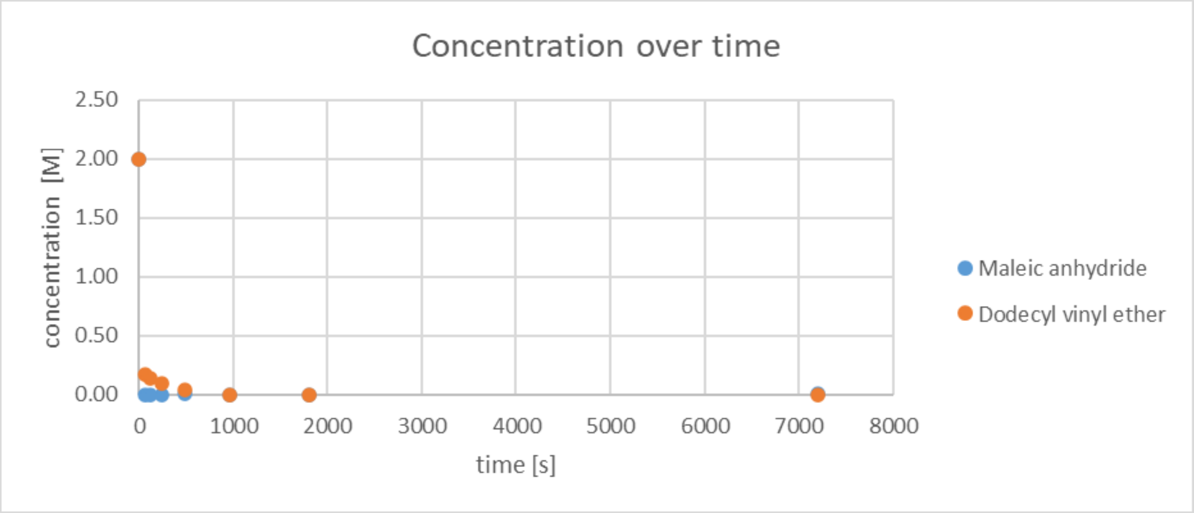

Concentration over time
concentration [M]
2.50
2.00
1.50
1.00
0.50
0.00
0
1000
2000
3000
4000
5000
6000
7000
8000
time [s]
Maleic anhydride
Dodecyl vinyl ether

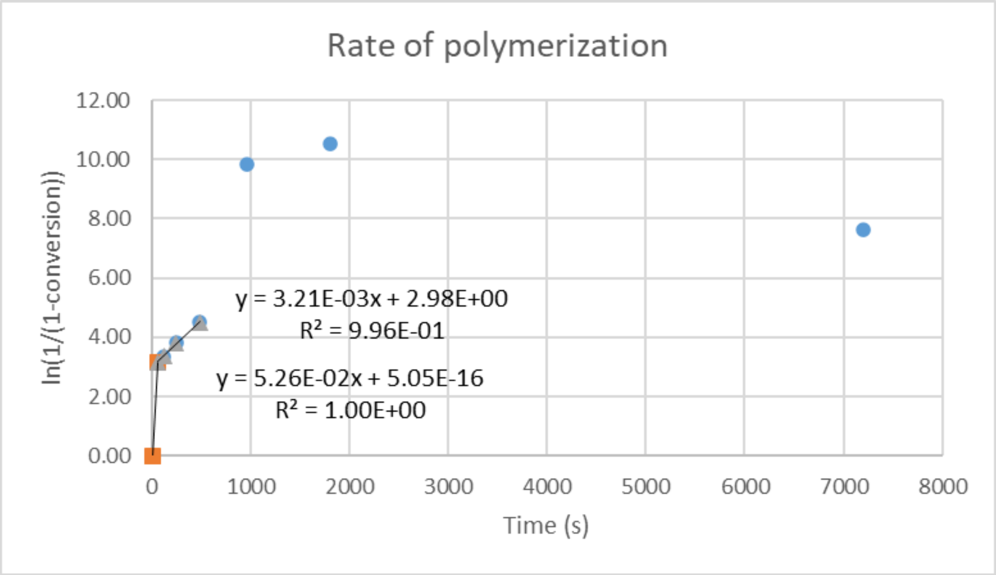

Rate of polymerization
ln(1/(1-conversion))
12.00
10.00
8.00
6.00
4.00
2.00
0.00
y = 3.21E-03x + 2.98E+00
R² = 9.96E-01
y = 5.26E-02x + 5.05E-16
R² = 1.00E+00
0
1000
2000
3000
4000
5000
6000
7000
8000
Time (s)

Differential scanning calorimetry (DSC)

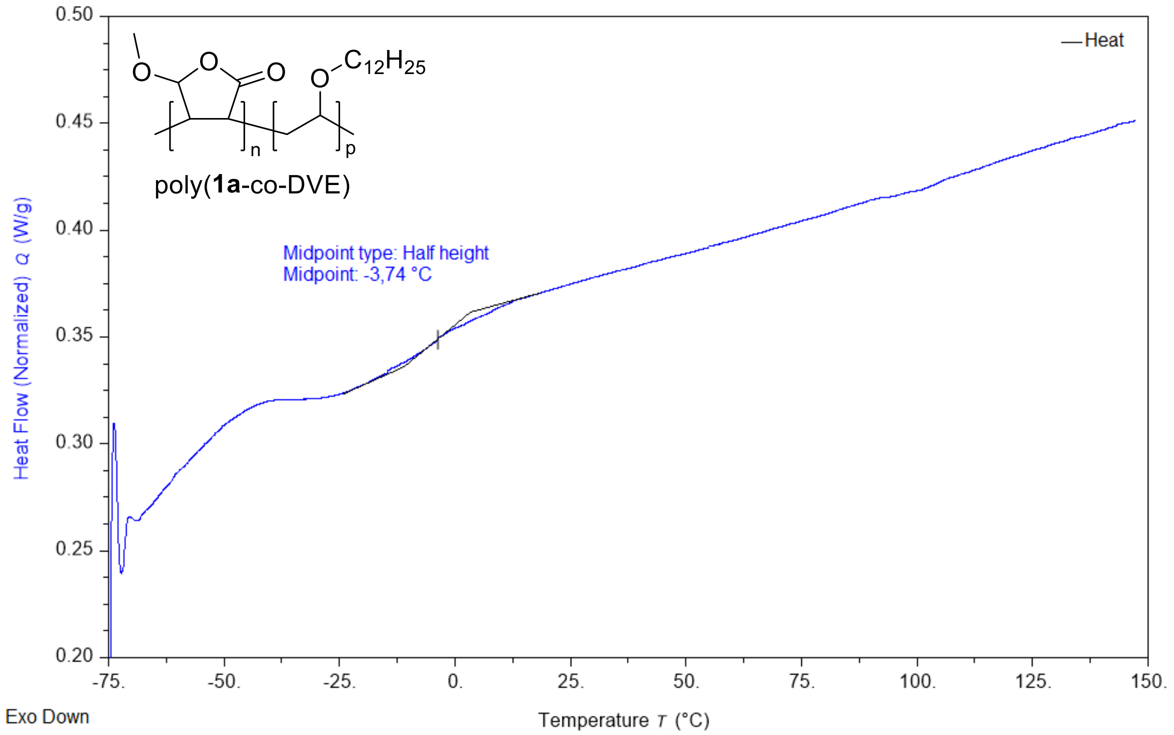

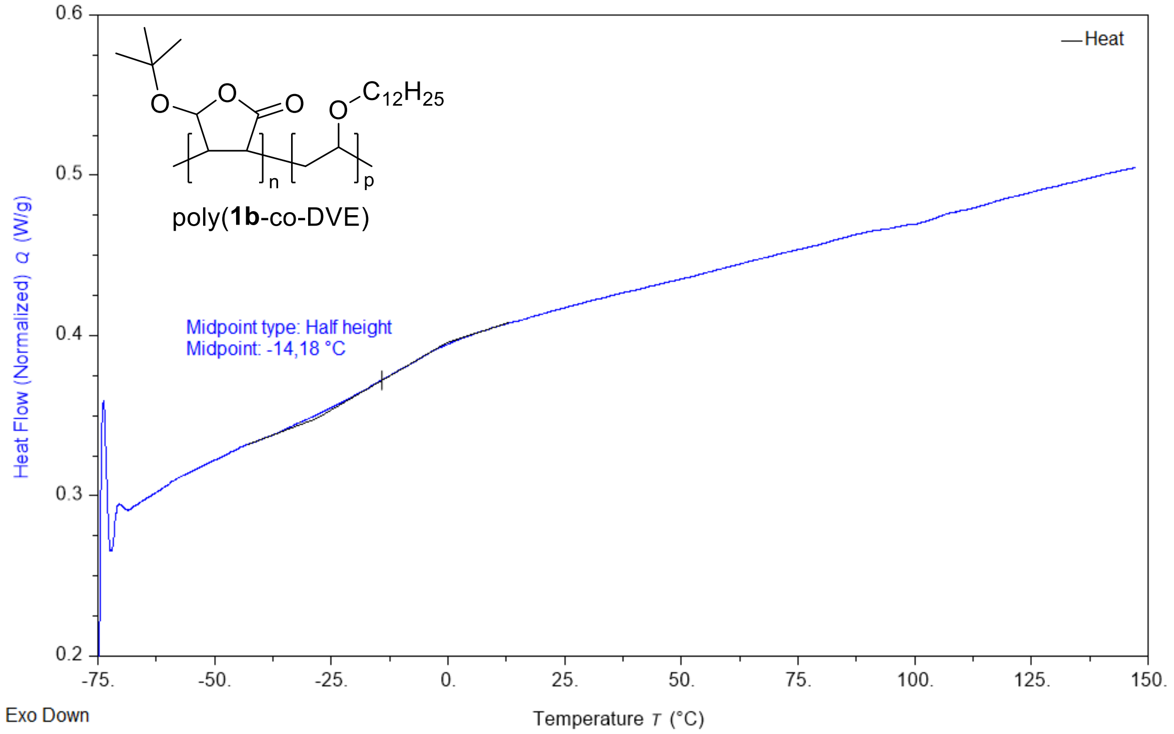

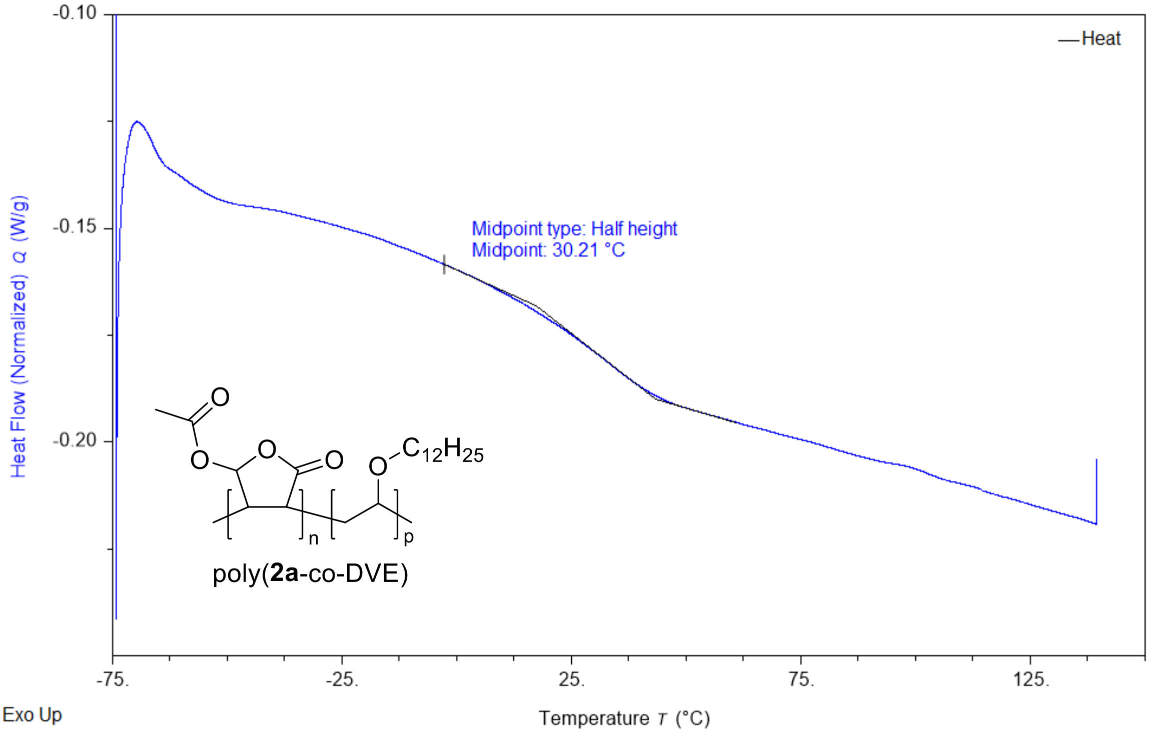
Heat
Midpoint type: Half height
Midpoint: 30.21 °C
Heat Flow (Normalized) Q (W/g)
-0.10
-0.15
-0.20
-75.
-25.
25.
75.
125.
Exo Up
Temperature T (°C)
poly(**2a**-co-DVE)

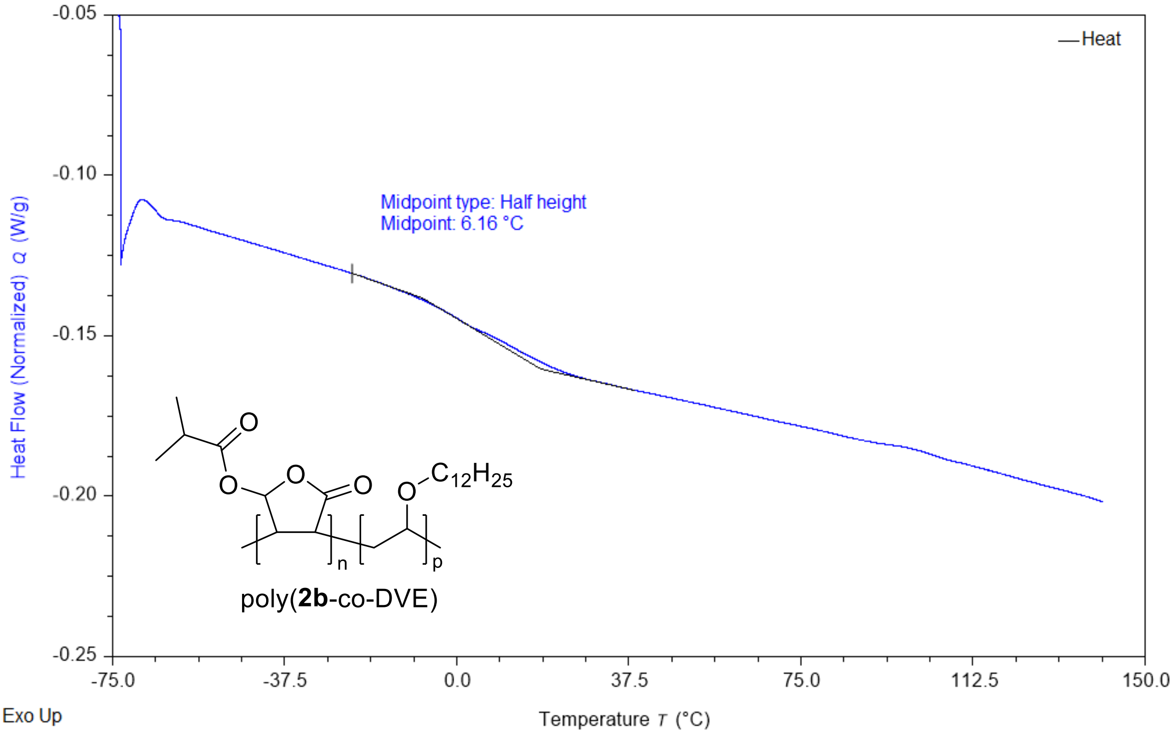
Heat
Midpoint type: Half height
Midpoint: 6.16 °C
Heat Flow (Normalized) Q (W/g)
-0.05
-0.10
-0.15
-0.20
-0.25
-75.0
-37.5
0.0
37.5
75.0
112.5
150.0
Exo Up
Temperature T (°C)
poly(**2b**-co-DVE)

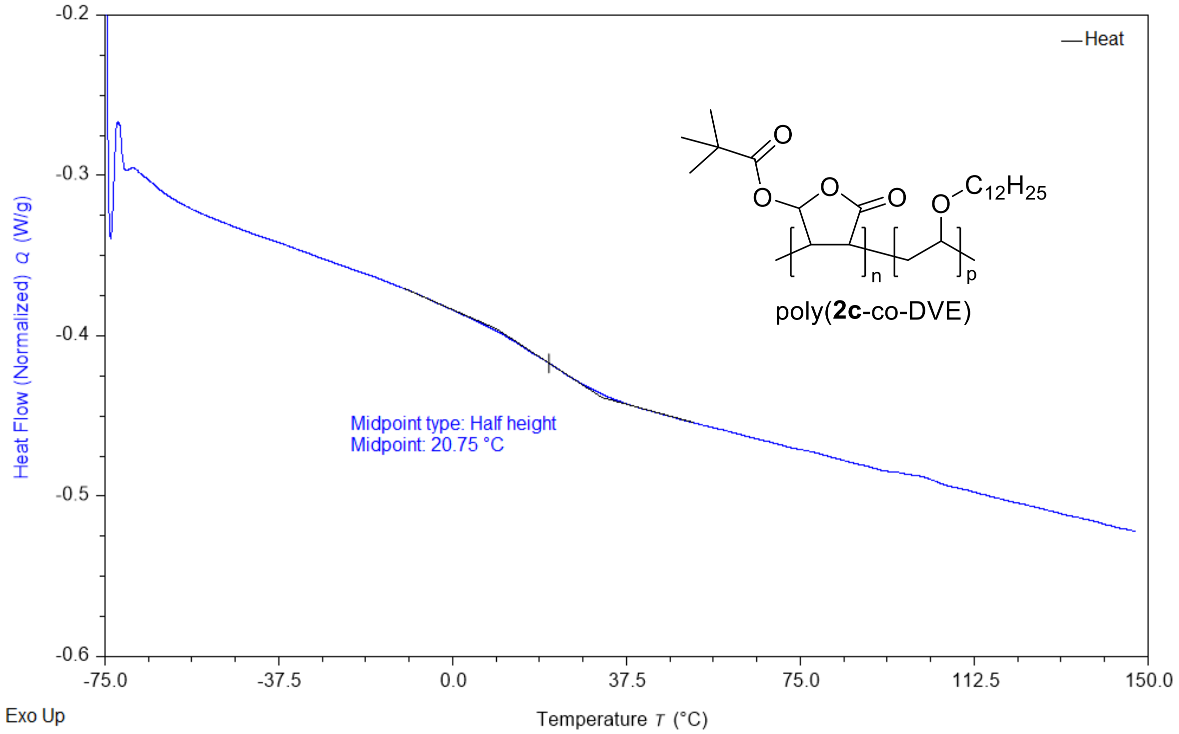
Heat
poly(2c-co-DVE)
Midpoint type: Half height
Midpoint: 20.75 °C
Heat Flow (Normalized) Q (W/g)
Temperature T (°C)
Exo Up

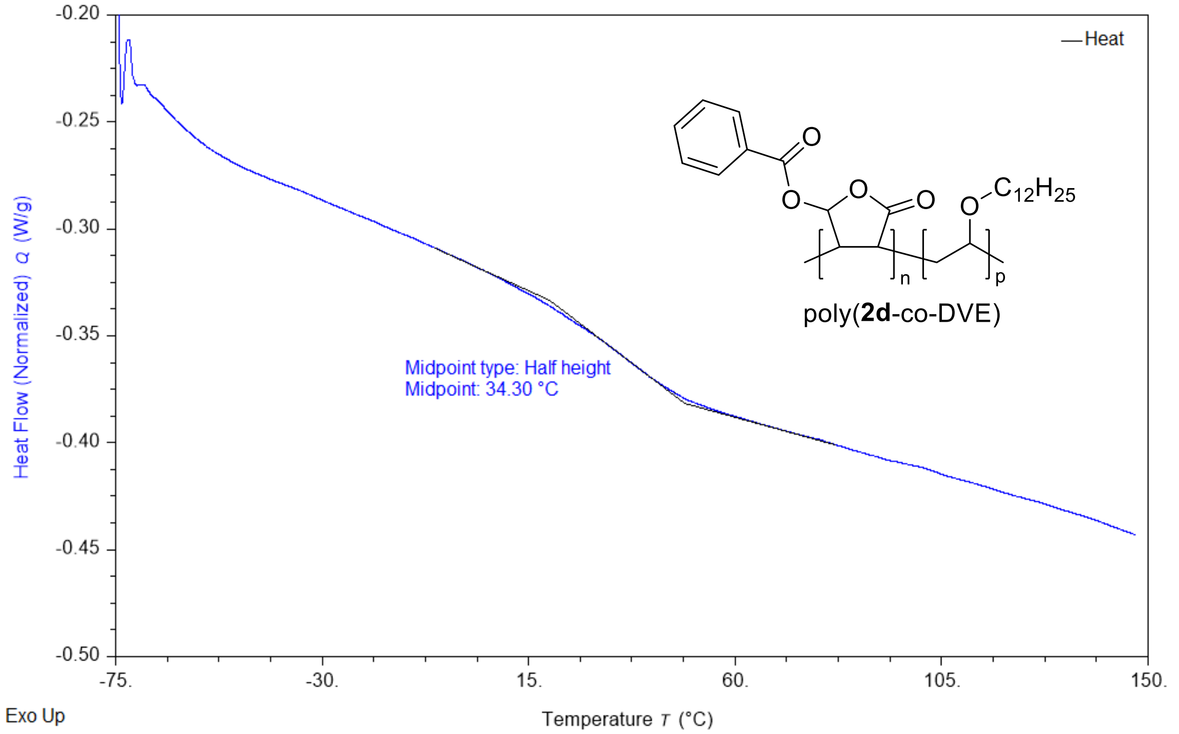
Heat
poly(2d-co-DVE)
Midpoint type: Half height
Midpoint: 34.30 °C
Heat Flow (Normalized) Q (W/g)
Temperature T (°C)
Exo Up

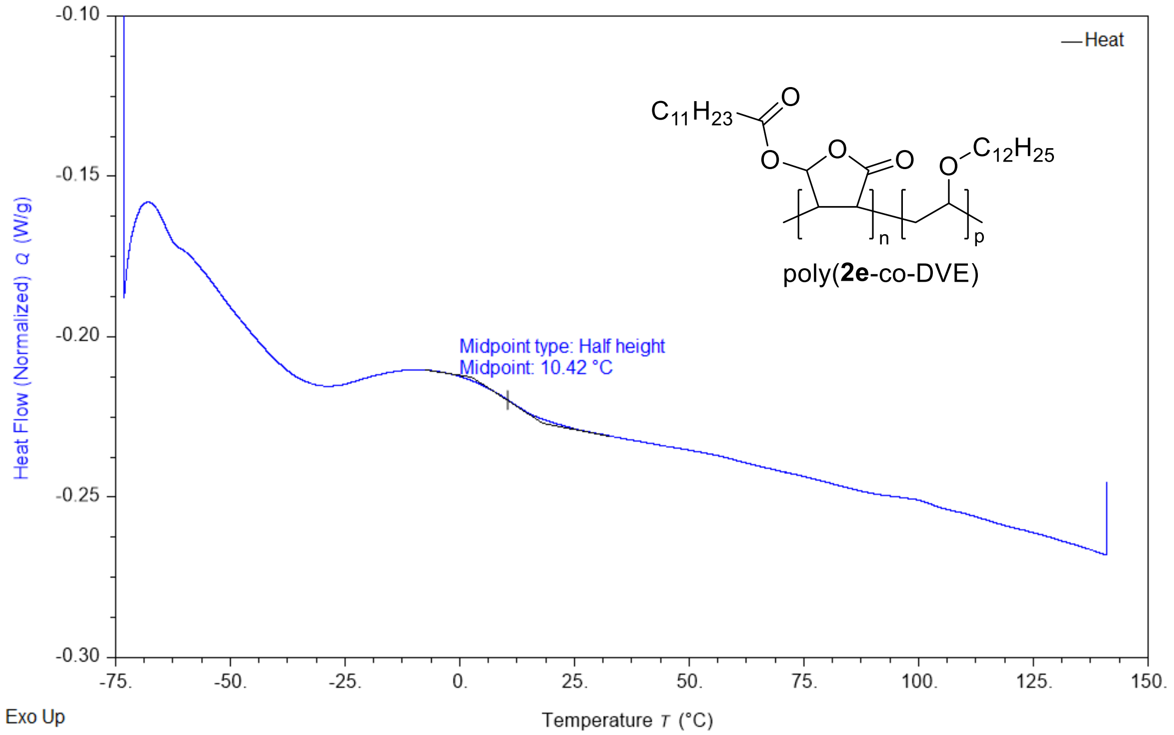
Heat
Midpoint type: Half height
Midpoint: 10.42 °C
poly(2e-co-DVE)
Heat Flow (Normalized) Q (W/g)
Temperature T (°C)
Exo Up

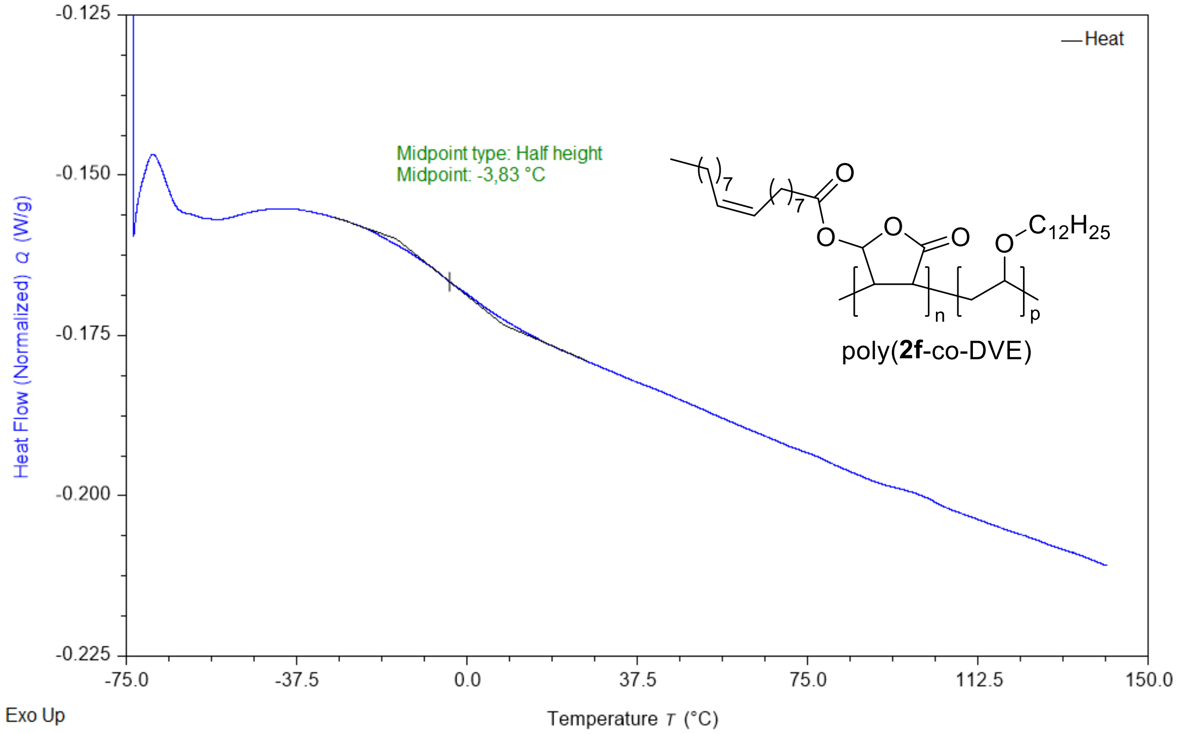
Heat
Midpoint type: Half height
Midpoint: -3,83 °C
poly(2f-co-DVE)
Heat Flow (Normalized) Q (W/g)
Temperature T (°C)
Exo Up

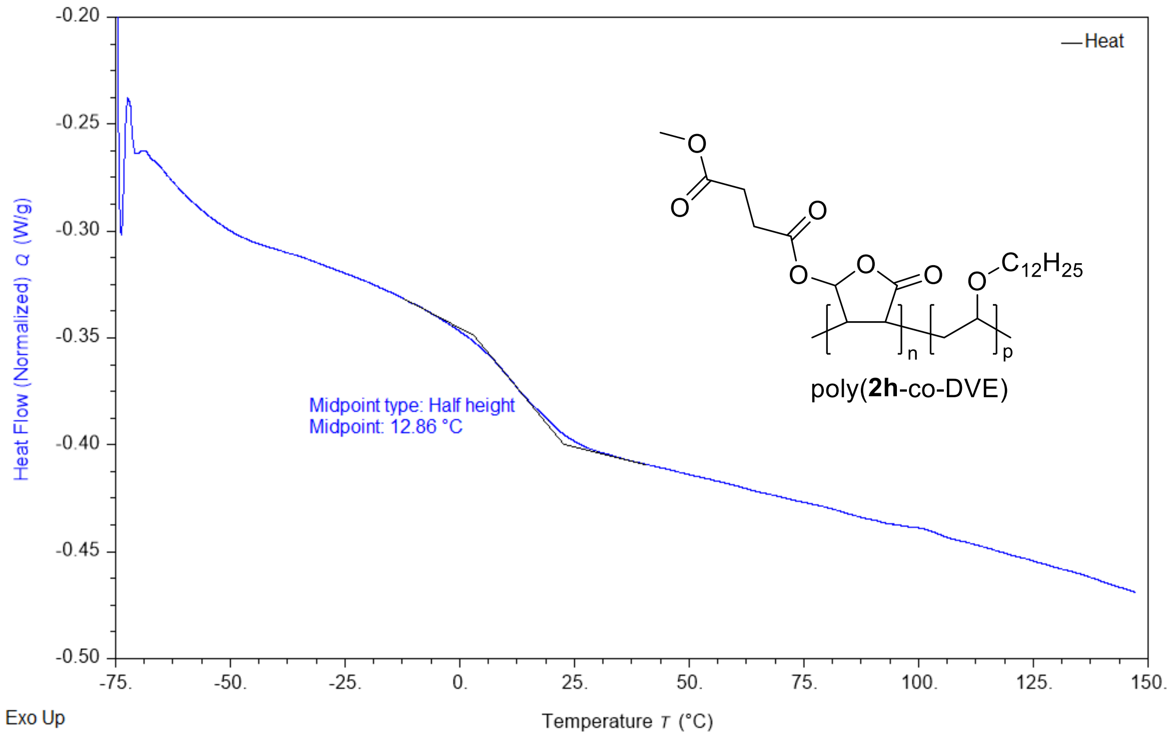
Heat
Heat Flow (Normalized) Q (W/g)
Midpoint type: Half height
Midpoint: 12.86 °C
poly(**2h**-co-DVE)
Exo Up
Temperature T (°C)

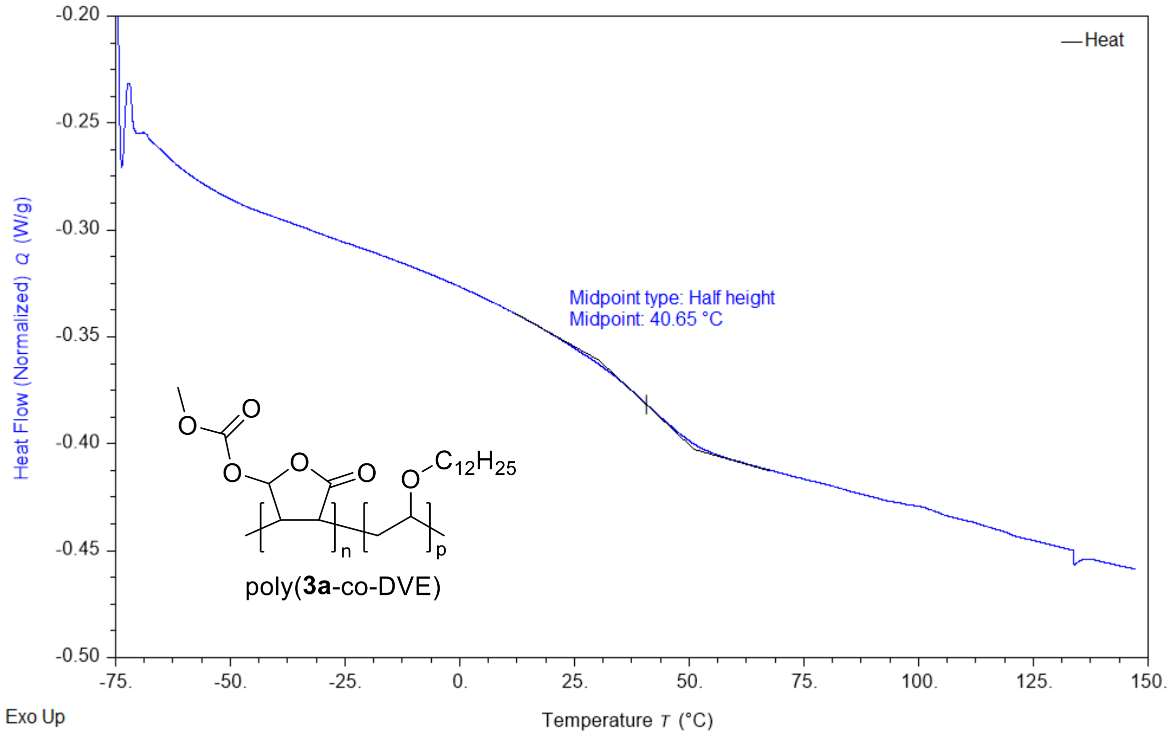
Heat
Heat Flow (Normalized) Q (W/g)
Midpoint type: Half height
Midpoint: 40.65 °C
poly(**3a**-co-DVE)
Exo Up
Temperature T (°C)

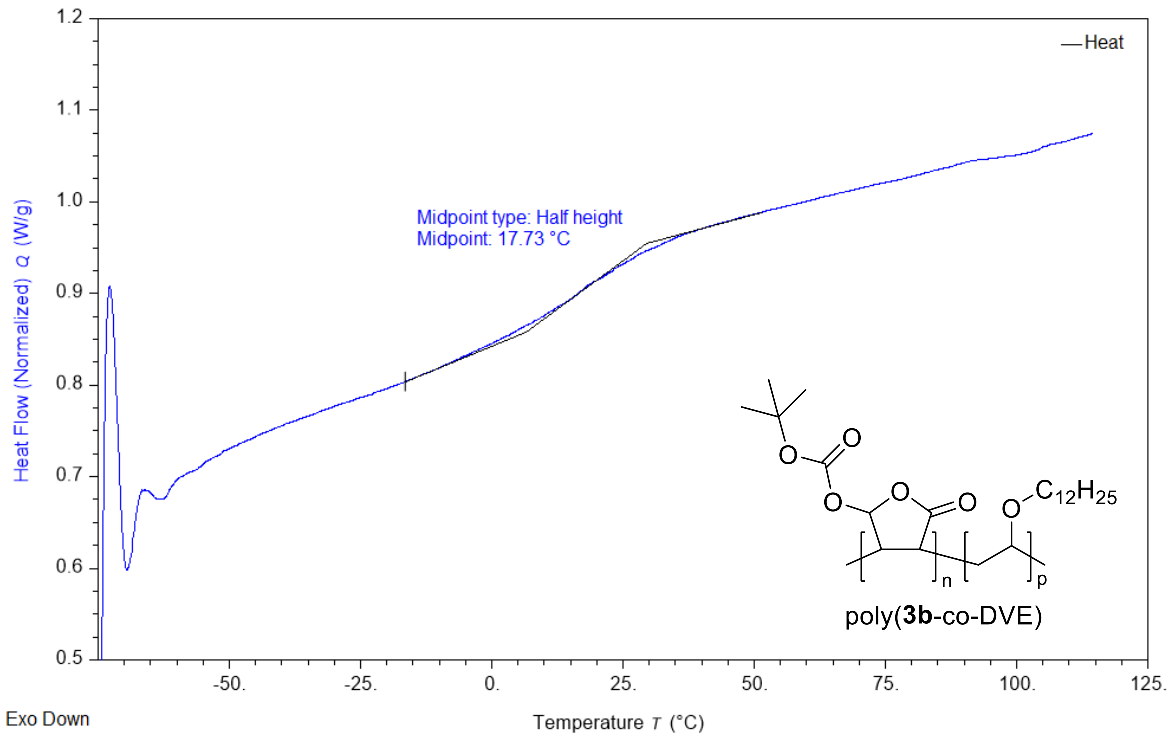
Heat
Midpoint type: Half height
Midpoint: 17.73 °C
Heat Flow (Normalized) Q (W/g)
1.2
1.1
1.0
0.9
0.8
0.7
0.6
0.5
-50.
-25.
0.
25.
50.
75.
100.
125.
Exo Down
Temperature T (°C)
O
O
O
O
O
O
$C_{12}H_{25}$
n
p
poly(**3b**-co-DVE)

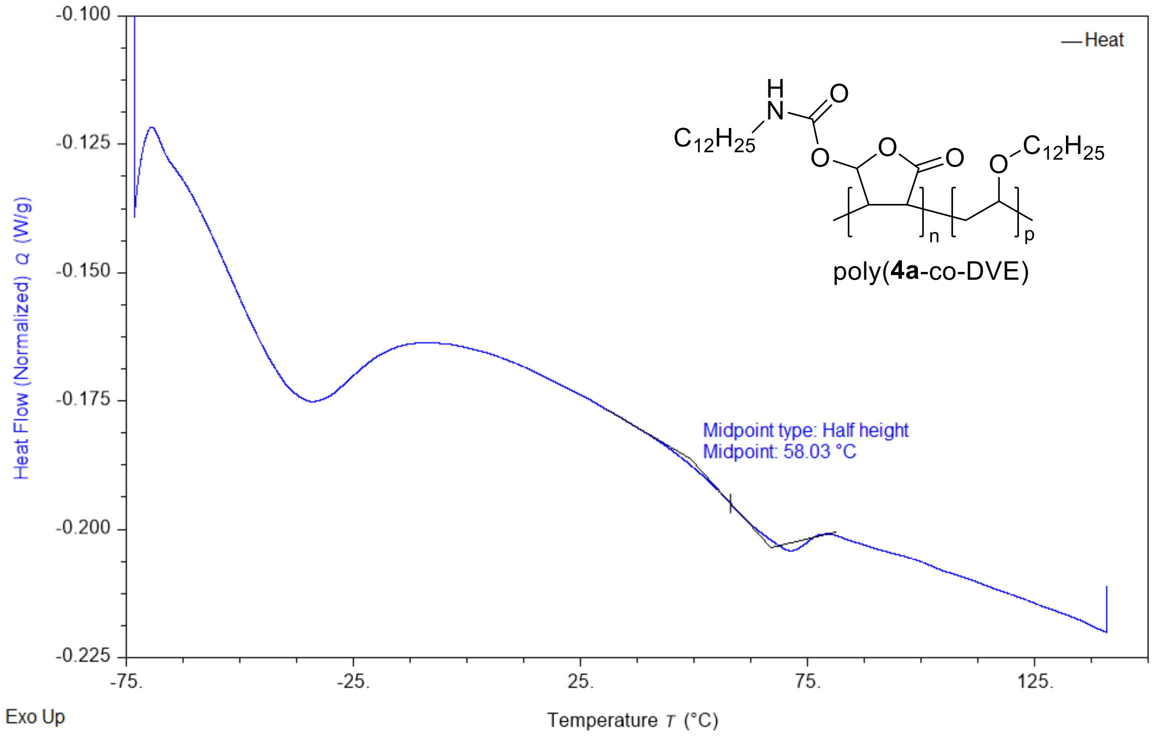
Heat
H
N
O
$C_{12}H_{25}$
O
O
O
O
$C_{12}H_{25}$
n
p
poly(**4a**-co-DVE)
Midpoint type: Half height
Midpoint: 58.03 °C
Heat Flow (Normalized) Q (W/g)
-0.100
-0.125
-0.150
-0.175
-0.200
-0.225
-75.
-25.
25.
75.
125.
Exo Up
Temperature T (°C)

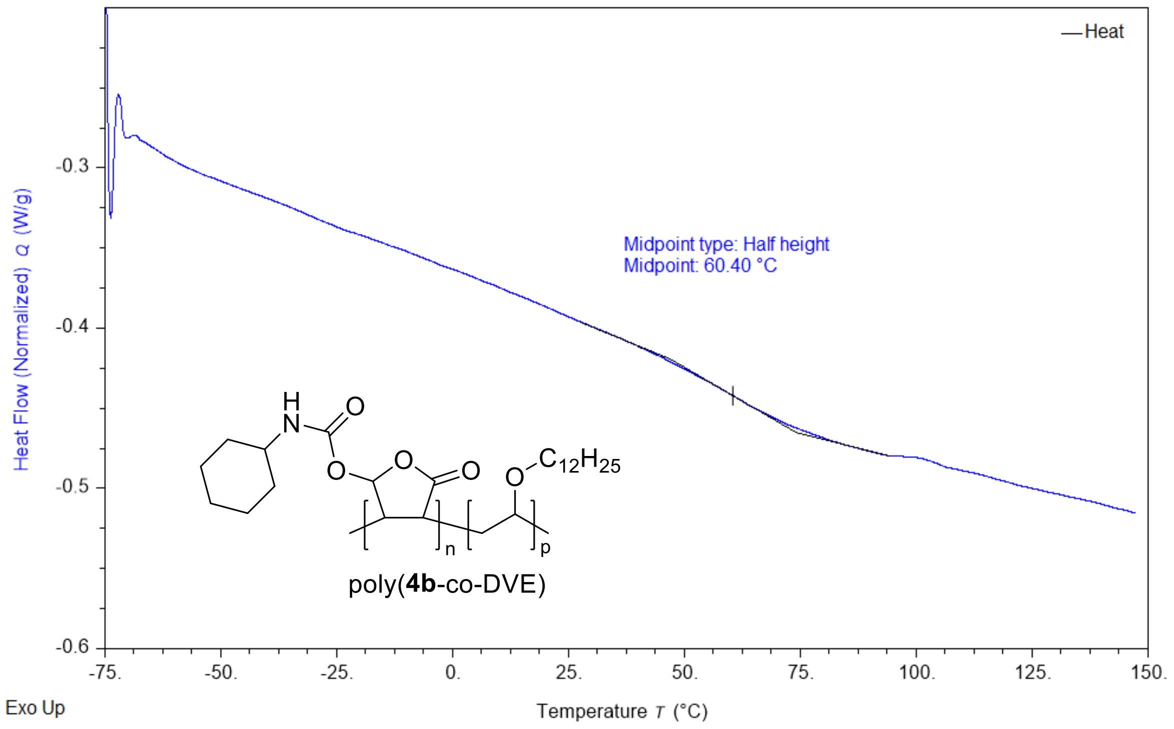
Heat
Midpoint type: Half height
Midpoint: 60.40 °C
poly(**4b**-co-DVE)
Heat Flow (Normalized) Q (W/g)
-0.3
-0.4
-0.5
-0.6
-75.
-50.
-25.
0.
25.
50.
75.
100.
125.
150.
Exo Up
Temperature T (°C)

Gel permeation chromatography (GPC)

Copolymers with DVE

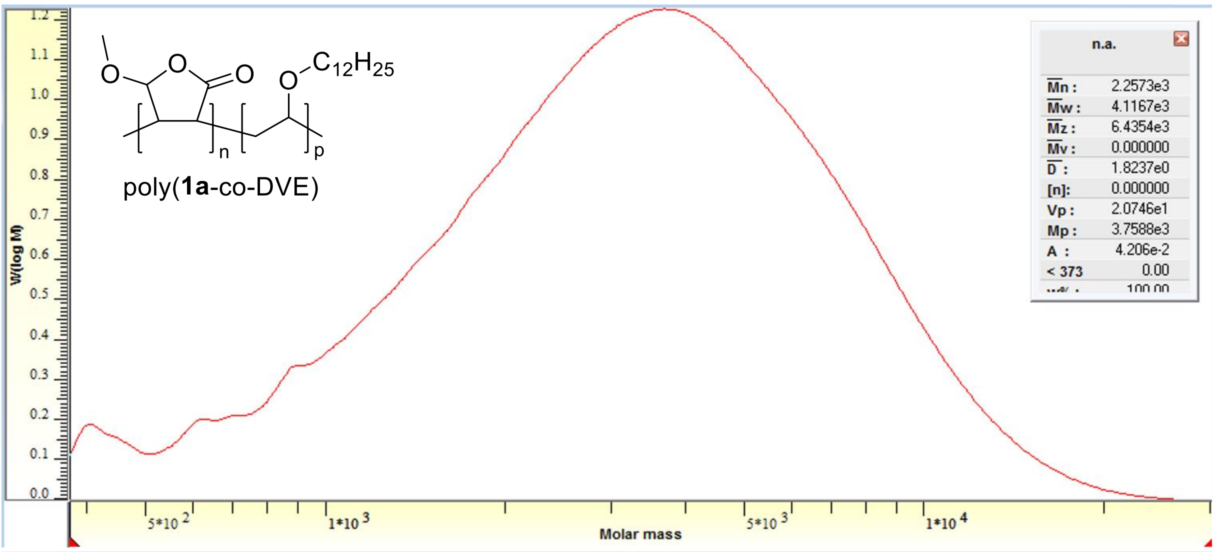

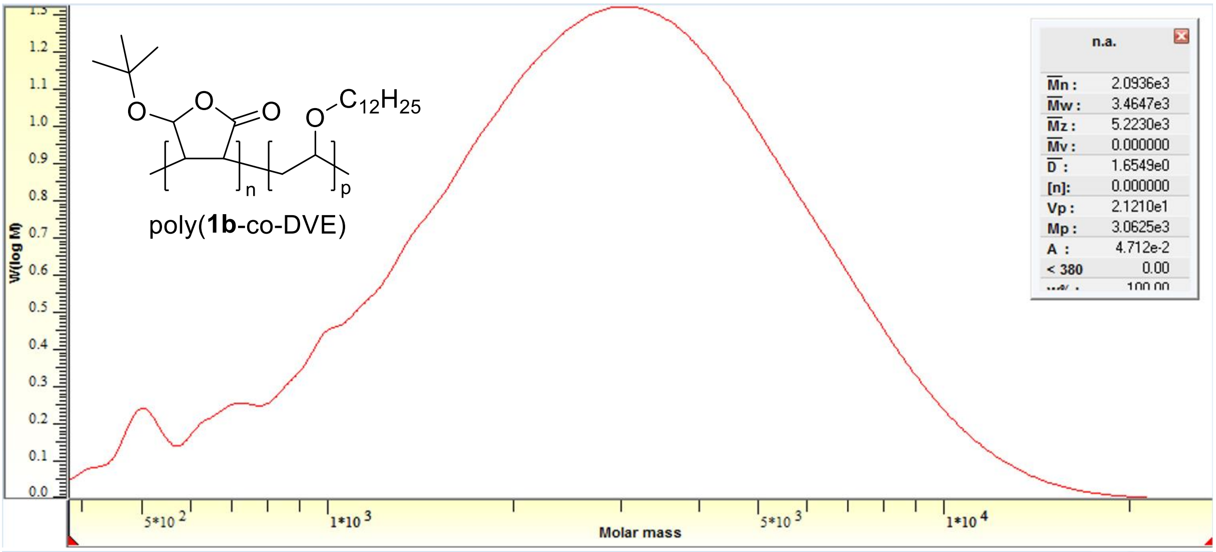

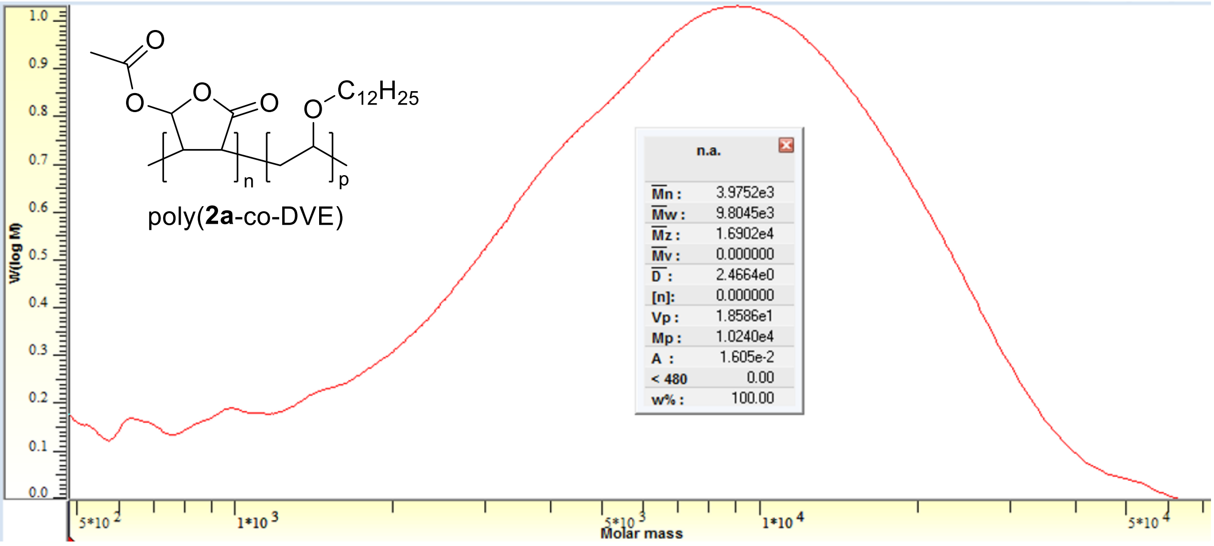

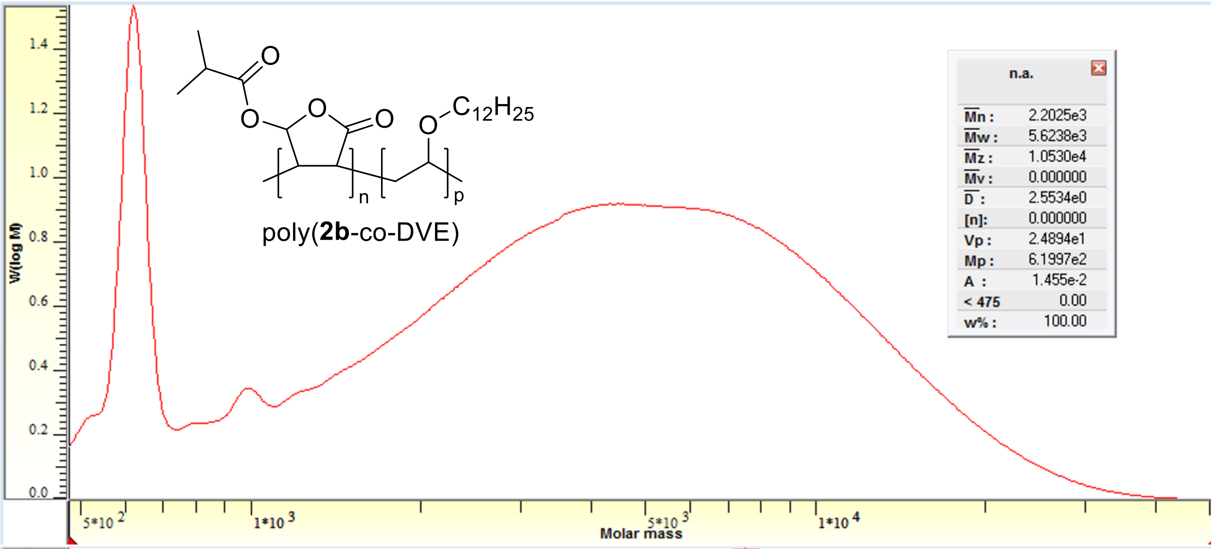
poly(2b-co-DVE)
W(log M)
Molar mass
n.a.
Mn : 2.2025e3
Mw : 5.6238e3
Mz : 1.0530e4
Mv : 0.000000
D : 2.5534e0
[n]: 0.000000
Vp : 2.4894e1
Mp : 6.1997e2
A : 1.455e-2
< 475 0.00
w% : 100.00

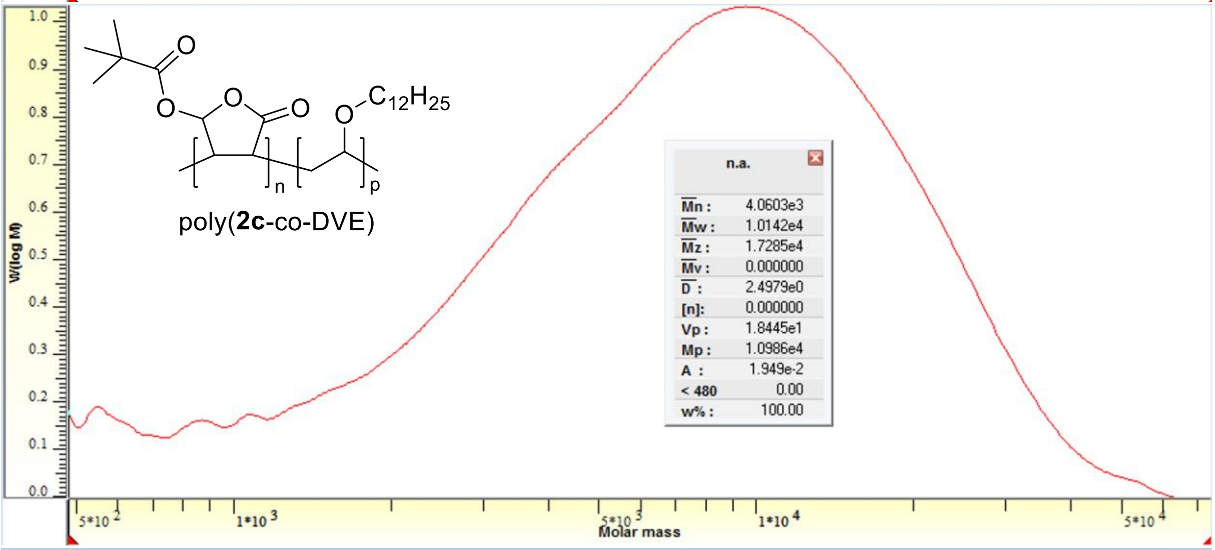
poly(2c-co-DVE)
W(log M)
Molar mass
n.a.
Mn : 4.0603e3
Mw : 1.0142e4
Mz : 1.7285e4
Mv : 0.000000
D : 2.4979e0
[n]: 0.000000
Vp : 1.8445e1
Mp : 1.0986e4
A : 1.949e-2
< 480 0.00
w% : 100.00

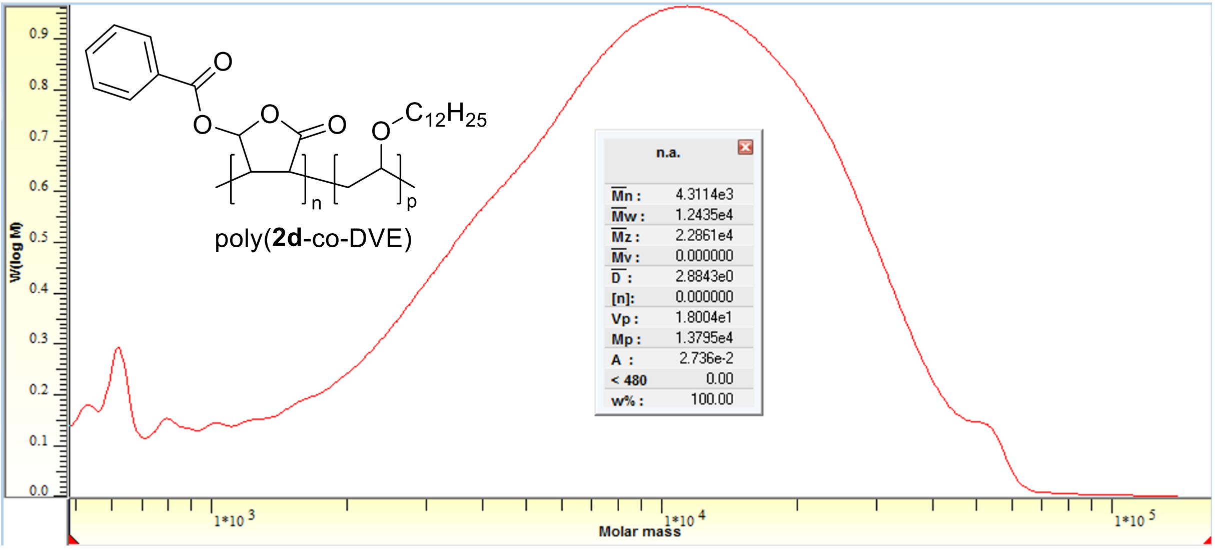
poly(2d-co-DVE)
W(log M)
Molar mass
n.a.
Mn : 4.3114e3
Mw : 1.2435e4
Mz : 2.2861e4
Mv : 0.000000
D : 2.8843e0
[n]: 0.000000
Vp : 1.8004e1
Mp : 1.3795e4
A : 2.736e-2
< 480 0.00
w% : 100.00

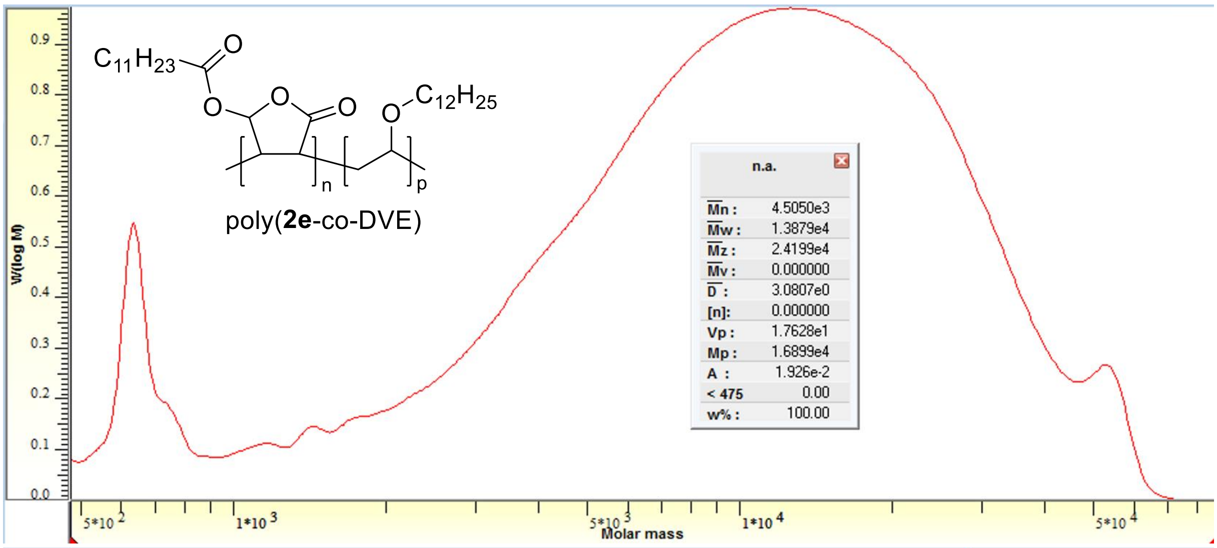

poly(2e-co-DVE)
n.a.
Mn : 4.5050e3
Mw : 1.3879e4
Mz : 2.4199e4
Mv : 0.000000
D : 3.0807e0
[n]: 0.000000
Vp : 1.7628e1
Mp : 1.6899e4
A : 1.926e-2
< 475 0.00
w% : 100.00
W(log M)
Molar mass

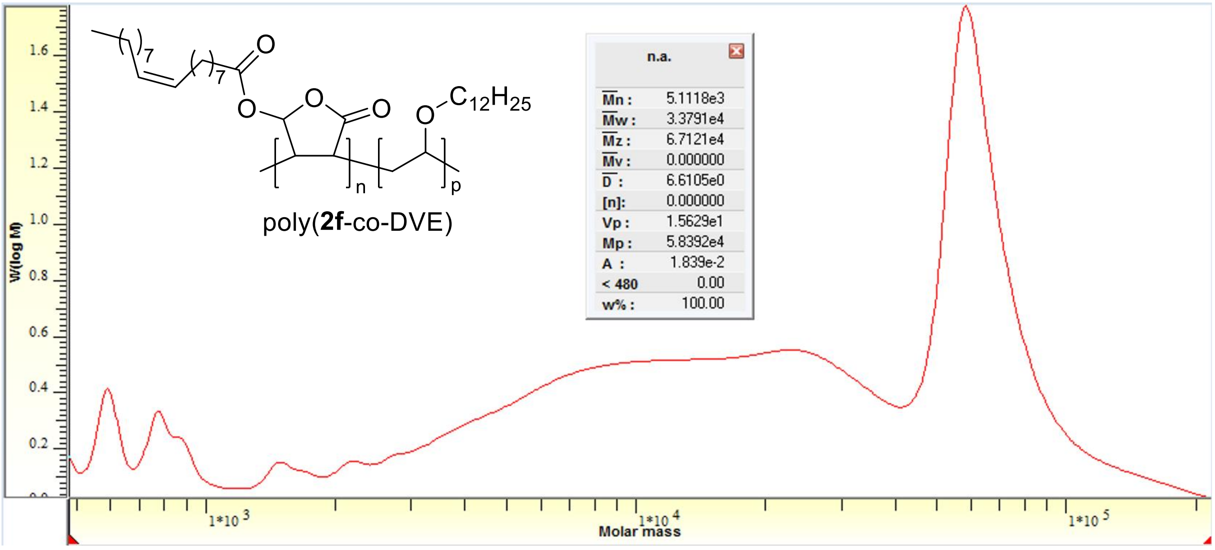

poly(2f-co-DVE)
n.a.
Mn : 5.1118e3
Mw : 3.3791e4
Mz : 6.7121e4
Mv : 0.000000
D : 6.6105e0
[n]: 0.000000
Vp : 1.5629e1
Mp : 5.8392e4
A : 1.839e-2
< 480 0.00
w% : 100.00
W(log M)
Molar mass

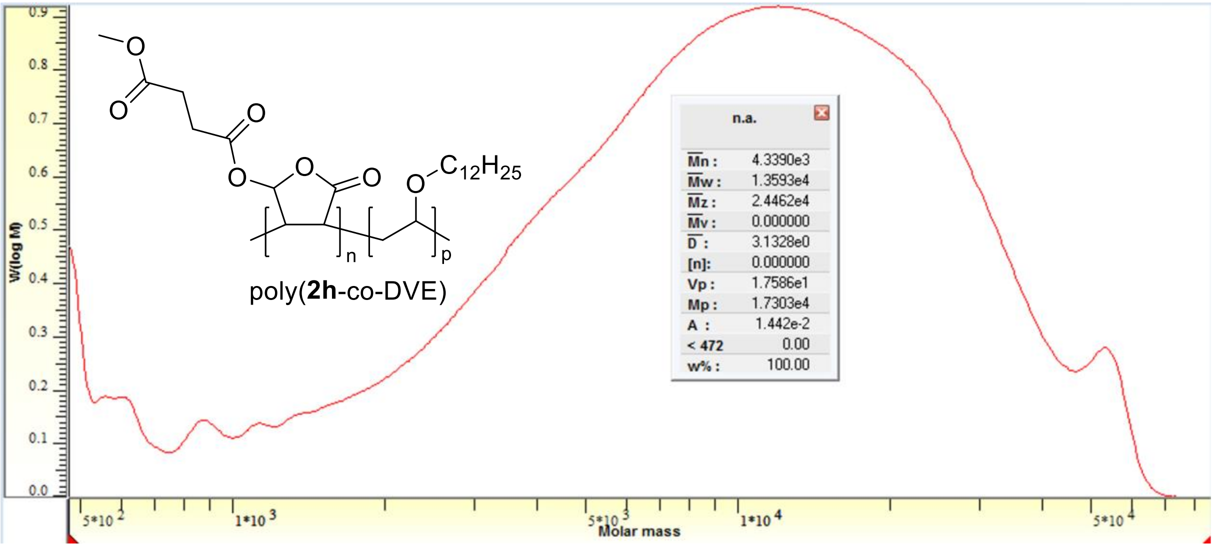

poly(2h-co-DVE)
n.a.
Mn : 4.3390e3
Mw : 1.3593e4
Mz : 2.4462e4
Mv : 0.000000
D : 3.1328e0
[n]: 0.000000
Vp : 1.7586e1
Mp : 1.7303e4
A : 1.442e-2
< 472 0.00
w% : 100.00
W(log M)
Molar mass

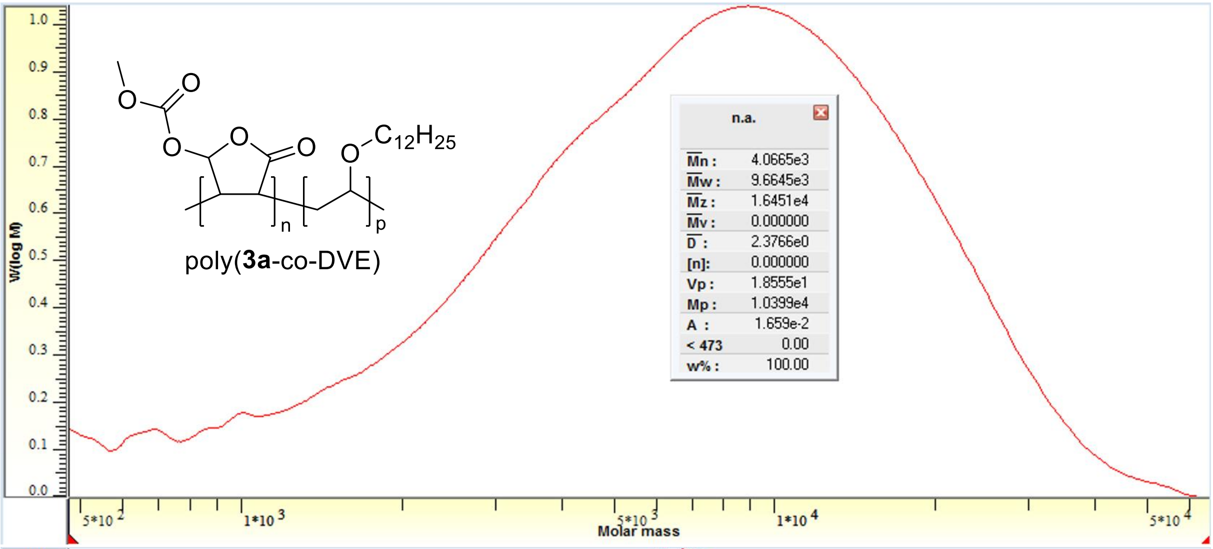
poly(3a-co-DVE)
n.a.
Mn : 4.0665e3
Mw : 9.6645e3
Mz : 1.6451e4
Mv : 0.000000
D : 2.3766e0
[n]: 0.000000
Vp : 1.8555e1
Mp : 1.0399e4
A : 1.659e-2
< 473 0.00
w% : 100.00
W(log M)
Molar mass

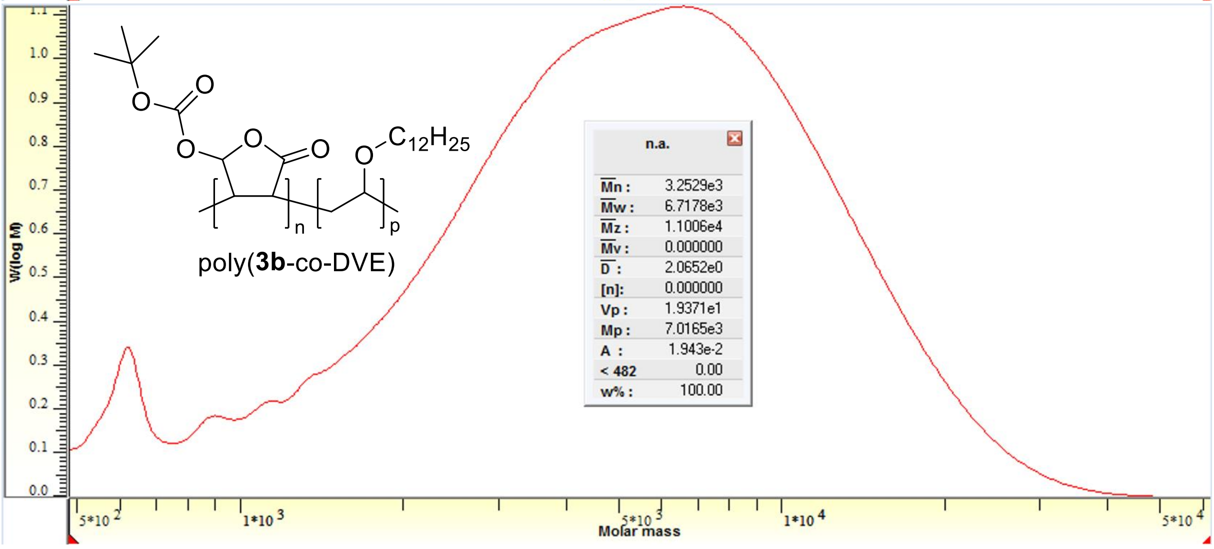
poly(3b-co-DVE)
n.a.
Mn : 3.2529e3
Mw : 6.7178e3
Mz : 1.1006e4
Mv : 0.000000
D : 2.0652e0
[n]: 0.000000
Vp : 1.9371e1
Mp : 7.0165e3
A : 1.943e-2
< 482 0.00
w% : 100.00
W(log M)
Molar mass

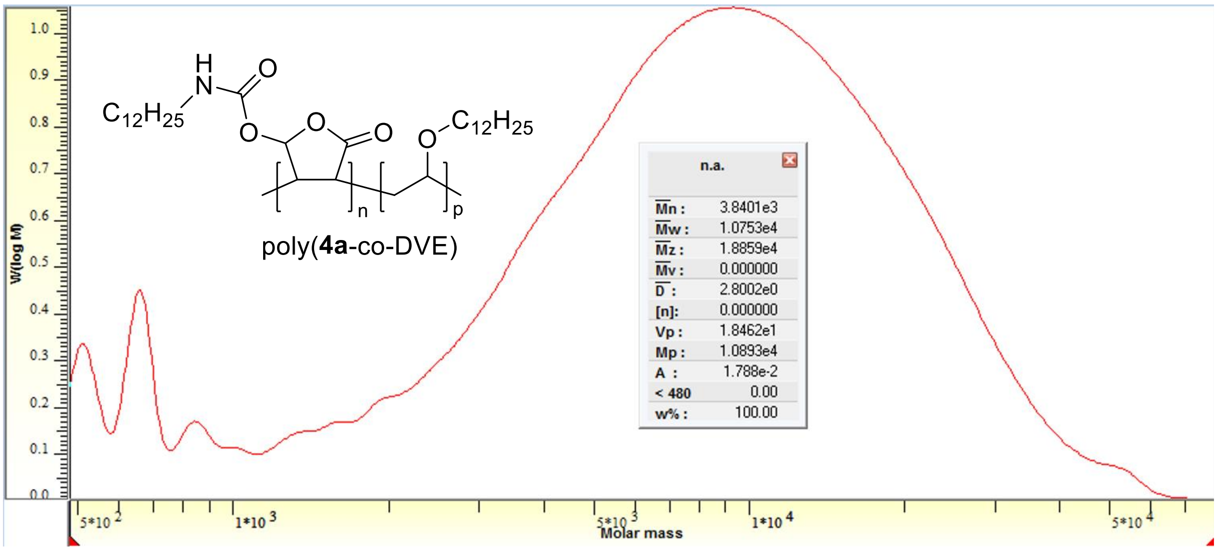
poly(4a-co-DVE)
n.a.
Mn : 3.8401e3
Mw : 1.0753e4
Mz : 1.8859e4
Mv : 0.000000
D : 2.8002e0
[n]: 0.000000
Vp : 1.8462e1
Mp : 1.0893e4
A : 1.788e-2
< 480 0.00
w% : 100.00
W(log M)
Molar mass

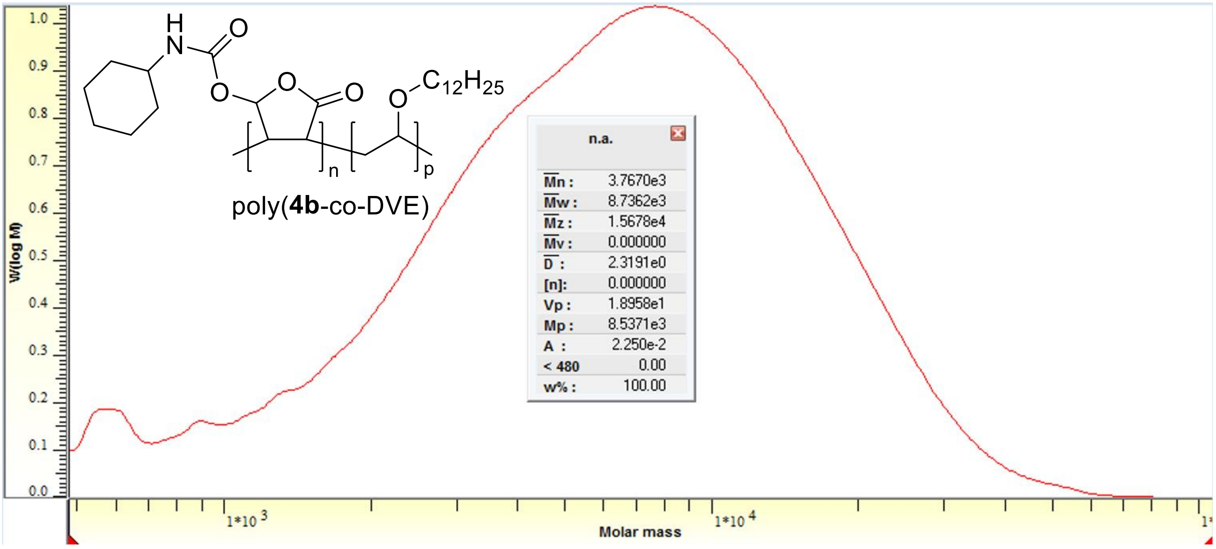
poly(4b-co-DVE)
n.a.
Mn : 3.7670e3
Mw : 8.7362e3
Mz : 1.5678e4
Mv : 0.000000
D : 2.3191e0
[n]: 0.000000
Vp : 1.8958e1
Mp : 8.5371e3
A : 2.250e-2
< 480 0.00
w% : 100.00
W(log M)
Molar mass
1*10 3
1*10 4

Homopolymer of 2a

Peak **A** indicates a M_n of 670 g/mol, corresponding to a DP of 4.7. Peak **B** corresponds to unreacted monomer **2a** (actual molecular weight of 142.11 g/mol).

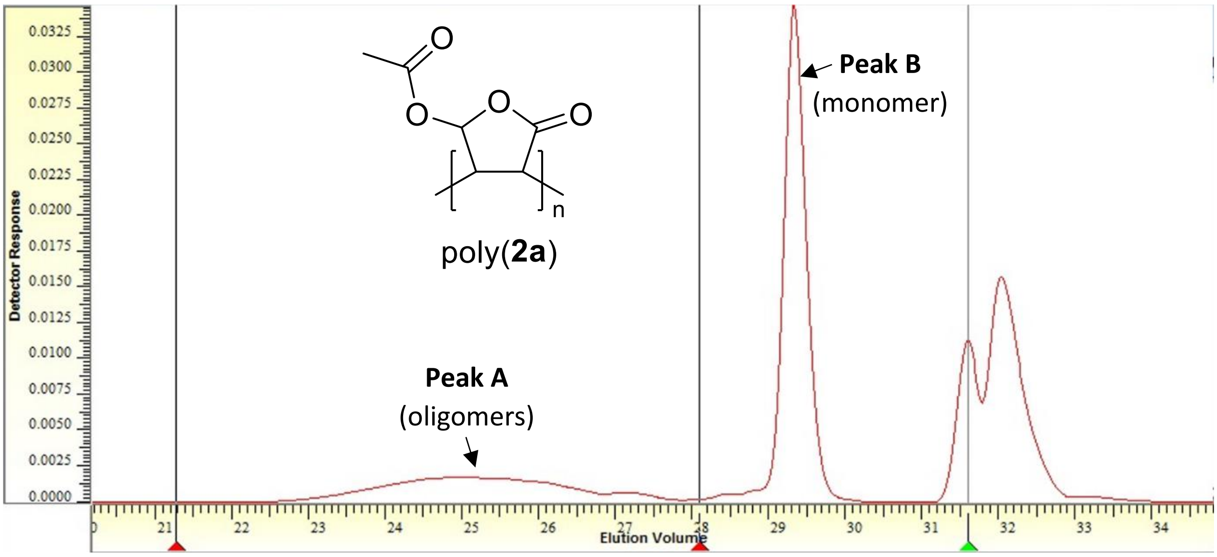

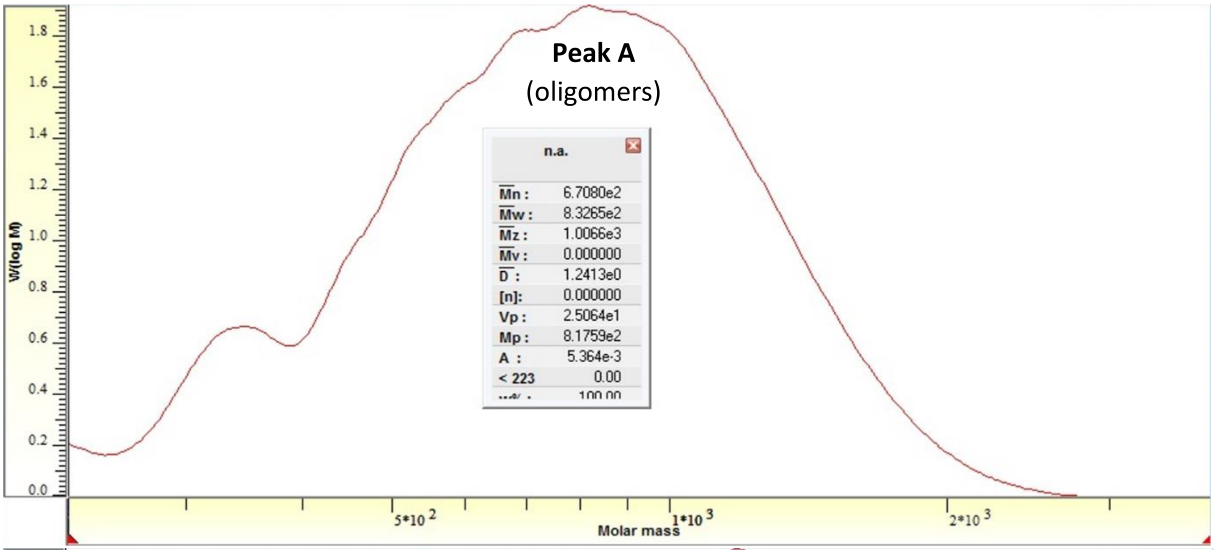

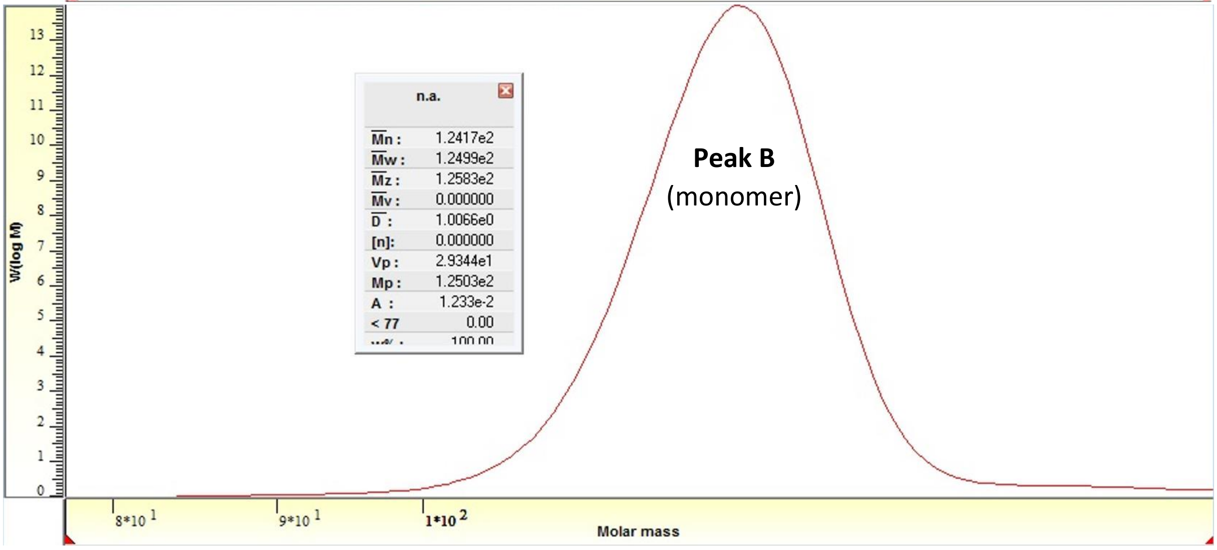

DFT data: optimized geometries, energies and XYZ Coordinates

All energies indicated are electronic energies + thermal energy correction, expressed in hartrees.

Butyl acrylate

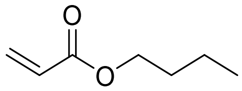

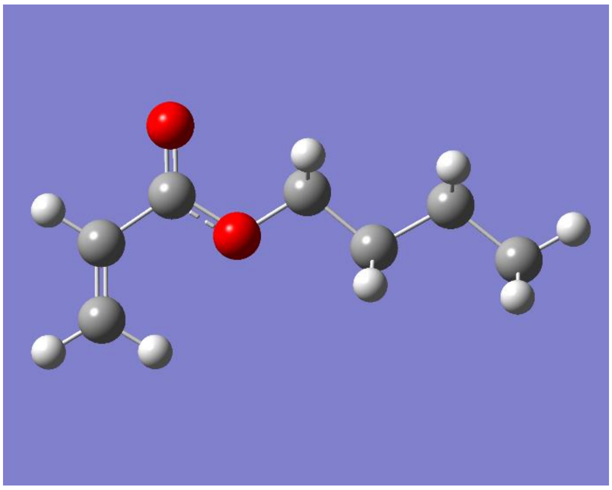

-423.938580

Tag	Symbol	X	Y	Z
1	C	-3.0771860	-1.6262040	-0.0004530
2	C	-3.0855240	-0.2981820	0.0004070
3	C	-1.8619140	0.5338120	0.0001700
4	O	-0.7303200	-0.1703490	0.0002760
5	H	-4.0010780	-2.1896540	-0.0003760
6	H	-4.0064940	0.2707840	0.0011210
7	O	-1.8872400	1.7463230	-0.0001490
8	H	-2.1494560	-2.1833640	-0.0011770
9	C	0.4865860	0.5877010	-0.0002290
10	H	0.5036840	1.2301900	-0.8831840
11	H	0.5037590	1.2312760	0.8819270
12	C	1.6476870	-0.3765740	0.0003070
13	H	1.5833660	-1.0228760	-0.8795460
14	H	1.5836130	-1.0215910	0.8811220
15	C	2.9773850	0.3649020	-0.0004270
16	H	3.0262950	1.0170700	-0.8773000
17	H	3.0264940	1.0184820	0.8753830
18	C	4.1601790	-0.5908040	0.0002060
19	H	4.1382230	-1.2359020	-0.8807820
20	H	5.1103400	-0.0556760	-0.0003690
21	H	4.1384640	-1.2344320	0.8822740

Furanone

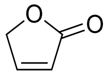

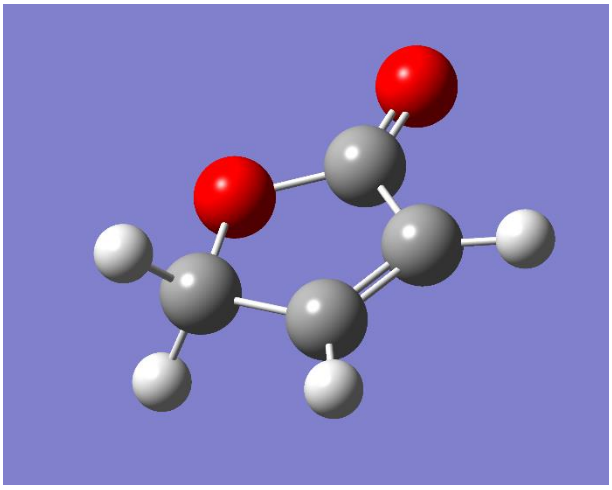

-305.019153

Tag	Symbol	X	Y	Z
1	C	1.3226730	-0.7159170	0.0000790
2	C	1.3139530	0.7718430	-0.0000590
3	C	0.0553880	1.2006830	-0.0000950
4	C	-0.8307760	0.0231940	0.0000100
5	O	-0.0513330	-1.0845370	-0.0001600
6	H	2.2175190	1.3630630	0.0000810
7	H	-0.3182180	2.2118130	0.0000420
8	O	-2.0341930	-0.0393850	0.0000880
9	H	1.8085040	-1.1307710	0.8858490
10	H	1.8089720	-1.1315480	-0.8850040

Furanol

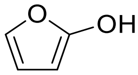

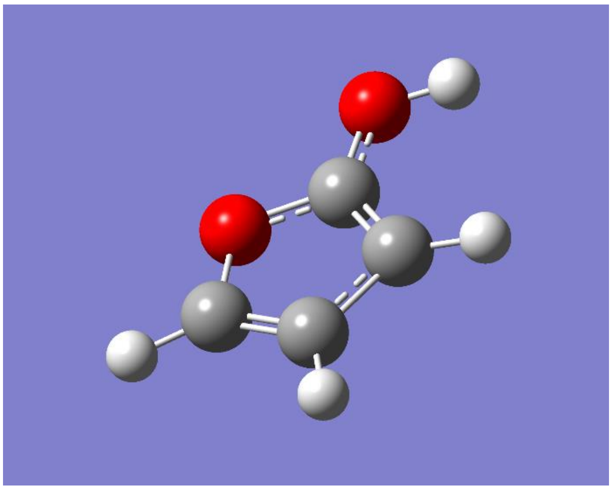

-304.999574

Tag	Symbol	X	Y	Z
1	C	1.3661820	-0.7000840	-0.0001580
2	C	1.4672350	0.6456240	-0.0000590
3	C	0.1261250	1.1626980	0.0001000
4	C	-0.6695070	0.0637340	-0.0000330
5	O	0.0463360	-1.0655190	0.0001480
6	H	2.3822000	1.2148310	-0.0003090
7	H	-0.1942060	2.1907630	0.0003030
8	O	-1.9908000	-0.1397770	-0.0001730
9	H	-2.4533200	0.7088870	0.0005990
10	H	2.0808190	-1.5039430	0.0005100

Methyl furanone

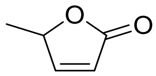

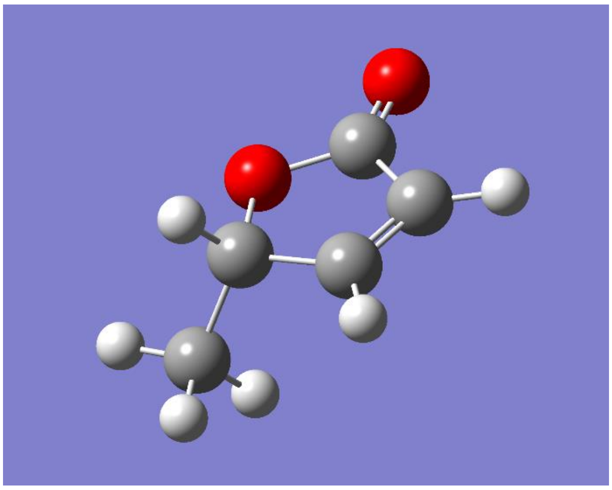

-344.273848

Tag	Symbol	X	Y	Z
1	C	1.0469430	-0.0673730	0.4647010
2	C	0.5414660	1.2968890	0.1297210
3	C	-0.7536320	1.2348280	-0.1620280
4	C	-1.1862570	-0.1677620	-0.0241330
5	O	-0.1109540	-0.9003880	0.3450840
6	H	1.1816330	2.1673520	0.1282630
7	H	-1.4238810	2.0264690	-0.4559520
8	O	-2.2744720	-0.6587770	-0.1896810
9	H	1.3843550	-0.1133270	1.5035690
10	C	2.1374670	-0.5590460	-0.4645920
11	H	3.0177280	0.0766830	-0.3708610
12	H	2.4174430	-1.5806610	-0.2122510
13	H	1.7902100	-0.5284210	-1.4980000

Methoxy methyl butenolide

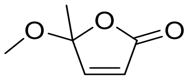

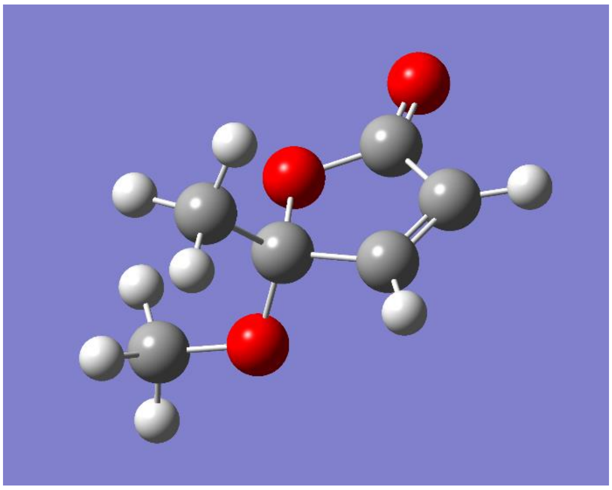

-458.697051

Tag	Symbol	X	Y	Z
1	C	0.5251040	0.3605080	0.0820840
2	C	-0.3710000	1.3747350	-0.5763080
3	C	-1.6189750	0.9300270	-0.5890660
4	C	-1.6391910	-0.4034040	0.0498190
5	O	-0.3791930	-0.7028860	0.4393050
6	H	0.0194270	2.3041670	-0.9639650
7	H	-2.5064250	1.3993840	-0.9823560
8	O	-2.5670650	-1.1451030	0.2380980
9	O	1.4199010	-0.0937220	-0.8716520
10	C	2.3908890	-1.0215810	-0.4131120
11	H	1.9302240	-1.8189210	0.1735820
12	H	2.8500570	-1.4559710	-1.2977830
13	H	3.1641790	-0.5299350	0.1808660
14	C	1.1742350	0.8680690	1.3539110
15	H	1.6597200	0.0519090	1.8870670
16	H	1.9131850	1.6311360	1.1102580
17	H	0.4141180	1.3017980	2.0023540

Furanol

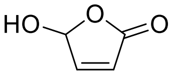

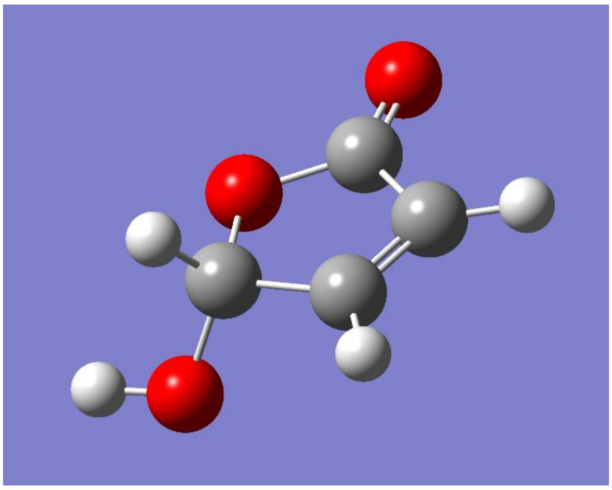

-380.208096

Tag	Symbol	X	Y	Z
1	C	1.0797920	-0.1119720	0.4006930
2	C	0.5909810	1.2774260	0.1201580
3	C	-0.7121550	1.2458500	-0.1200170
4	C	-1.1654460	-0.1589510	-0.0213940
5	O	-0.0900290	-0.9252550	0.2795150
6	H	1.2560490	2.1278150	0.1210980
7	H	-1.3806450	2.0566650	-0.3606730
8	O	-2.2668190	-0.6191310	-0.1606130
9	H	1.4410010	-0.2255290	1.4268430
10	O	2.0381530	-0.4782720	-0.5222820
11	H	2.4741220	-1.2918110	-0.2368710

Hydroxy furanol

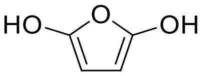

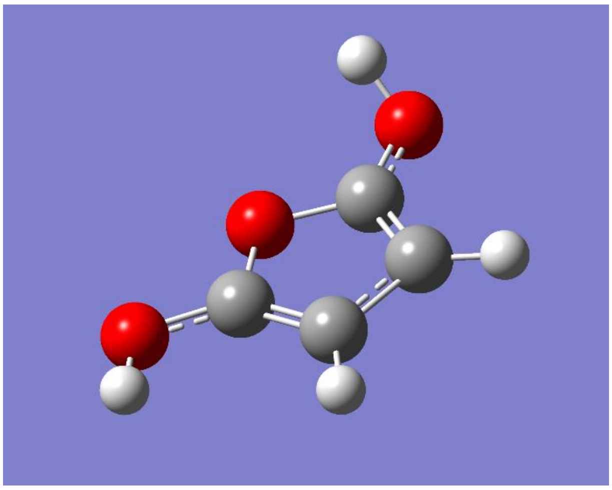

-380.187757

Tag	Symbol	X	Y	Z
1	C	-1.0963680	-0.0072590	0.0019890
2	C	-0.6982030	1.2788080	0.0177600
3	C	0.7473360	1.2467610	0.0031890
4	C	1.0763990	-0.0634380	-0.0072440
5	O	-0.0214430	-0.8471840	-0.0186330
6	H	-1.3395460	2.1433440	0.0358950
7	H	1.4281290	2.0812740	0.0112750
8	O	2.2200270	-0.7592130	-0.0013860
9	O	-2.3045670	-0.5811980	-0.0748390
10	H	2.9706560	-0.1525100	0.0498850
11	H	-2.3863610	-1.3005910	0.5676430

Methoxy butenolide 1a

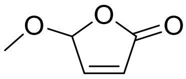

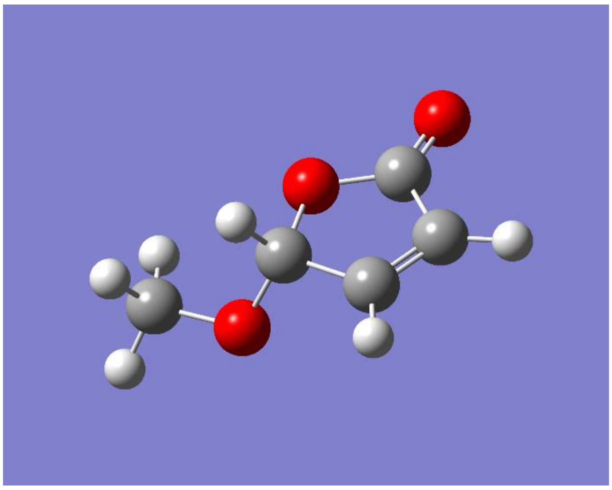

-419.441938

Tag	Symbol	X	Y	Z
1	C	0.6217350	0.3821430	0.4290610
2	C	-0.2818300	1.5288490	0.0898560
3	C	-1.4979440	1.0686510	-0.1673420
4	C	-1.4757640	-0.4032940	-0.0233130
5	O	-0.2200550	-0.7720200	0.3257870
6	H	0.0722470	2.5483030	0.0682650
7	H	-2.3864710	1.6107800	-0.4485000
8	O	-2.3656860	-1.1986990	-0.1638620
9	H	0.9832520	0.4165130	1.4647020
10	O	1.6700400	0.3134000	-0.4553010
11	C	2.6320740	-0.6700240	-0.1060860
12	H	2.2115160	-1.6743410	-0.1810040
13	H	3.4609390	-0.5749320	-0.8026900
14	H	2.9945030	-0.5057350	0.9131800

Methoxy furanol

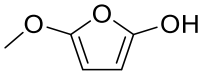

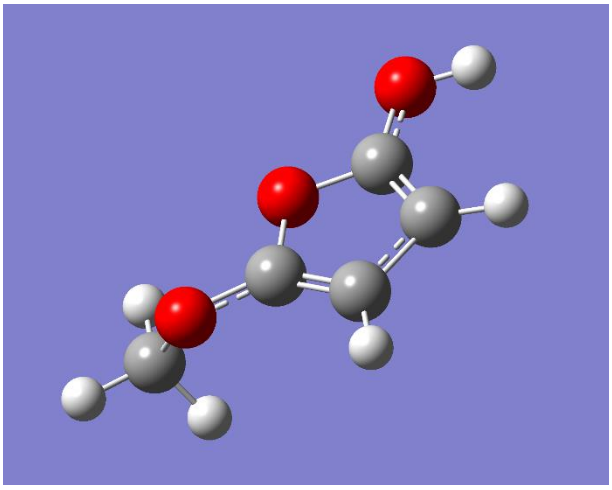

-419.420238

Tag	Symbol	X	Y	Z
1	C	-0.6455770	0.4007310	-0.2002090
2	C	0.0454590	1.5259400	0.0705330
3	C	1.4276620	1.1276290	0.1999780
4	C	1.4324930	-0.2104370	0.0060080
5	O	0.1961480	-0.6792110	-0.2437050
6	H	-0.3670230	2.5165040	0.1607530
7	H	2.2799790	1.7501690	0.4143520
8	O	2.3703570	-1.1656280	0.0108160
9	O	-1.9276480	0.1683320	-0.4821750
10	H	3.2303080	-0.7771350	0.2199550
11	C	-2.5864590	-0.7200180	0.4222080
12	H	-3.6322970	-0.7513900	0.1290220
13	H	-2.1621520	-1.7224350	0.3603510
14	H	-2.5011360	-0.3467310	1.4449780

Acetoxy butenolide 2a

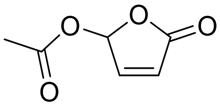

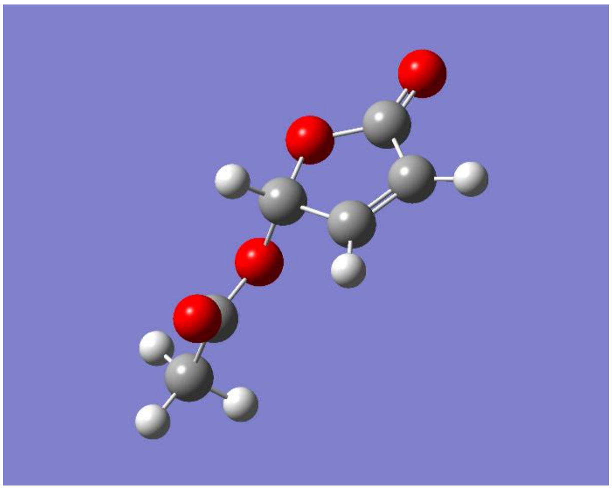

-532.727121

Tag	Symbol	X	Y	Z
1	C	0.1331960	0.1327330	-0.5566190
2	C	0.6310160	1.2914640	0.2567350
3	C	1.8873560	1.0557220	0.6083790
4	C	2.2823740	-0.2549540	0.0518650
5	O	1.2115070	-0.7598890	-0.6248080
6	H	0.0107090	2.1447080	0.4835080
7	H	2.5567690	1.6620270	1.1968140
8	O	3.3252620	-0.8417610	0.1182810
9	H	-0.1789190	0.4156040	-1.5626140
10	O	-0.9206950	-0.5668870	0.0855300
11	C	-2.1459210	-0.0175010	-0.0309090
12	C	-3.1907950	-0.8590910	0.6209150
13	H	-4.1626960	-0.3897440	0.5075750
14	H	-3.1969990	-1.8504780	0.1687570
15	H	-2.9542620	-0.9797100	1.6778990
16	O	-2.3233180	1.0319570	-0.6007700

Acetoxy furanol

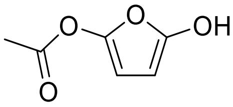

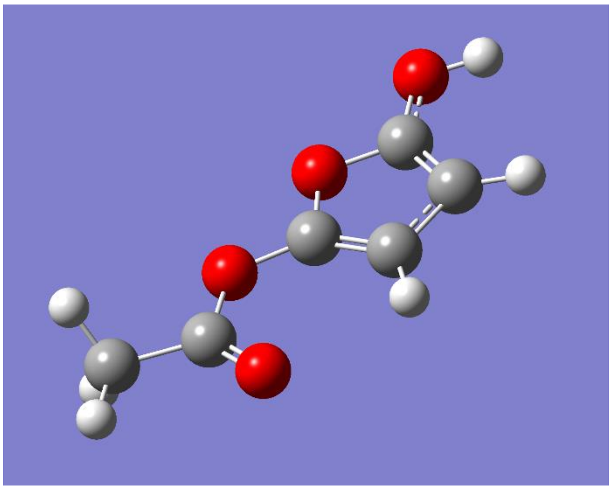

-532.704948

Tag	Symbol	X	Y	Z
1	C	-0.1022360	0.0064570	0.0000000
2	C	-0.5504000	1.2792210	-0.0001040
3	C	-1.9906420	1.1766140	-0.0001160
4	C	-2.2611520	-0.1473100	0.0000070
5	O	-1.1297320	-0.8785550	0.0000770
6	H	0.0420320	2.1740740	-0.0001810
7	H	-2.7053740	1.9820060	-0.0001810
8	O	-3.3687930	-0.8944390	0.0000640
9	O	1.0870110	-0.6390880	0.0001380
10	C	2.2568820	0.0487370	0.0001070
11	C	3.4167640	-0.8890260	-0.0002000
12	H	4.3424830	-0.3227990	0.0008890
13	H	3.3702820	-1.5315930	0.8787600
14	H	3.3712390	-1.5295210	-0.8807410
15	O	2.3006740	1.2500330	0.0001910
16	H	-4.1492410	-0.3239310	-0.0004590

Benzoyloxy butenolide 2d

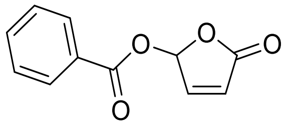

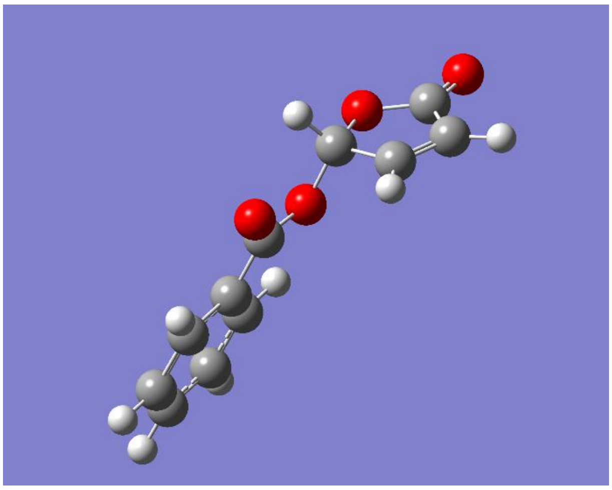

-724.251278

Tag	Symbol	X	Y	Z
1	C	1.7706410	0.5116180	-0.5114830
2	C	2.3746480	1.1197020	0.7206710
3	C	3.4607820	0.4319150	1.0444100
4	C	3.6289990	-0.6546490	0.0570010
5	O	2.6038950	-0.5653950	-0.8388380
6	H	1.9404200	1.9764580	1.2122290
7	H	4.1407830	0.5780320	1.8678900
8	O	4.4768530	-1.4967280	-0.0317130
9	H	1.7129880	1.2060400	-1.3502520
10	O	0.4869460	-0.0424650	-0.2695750
11	C	-0.5335390	0.8333170	-0.2553950
12	O	-0.3575320	2.0239200	-0.3792150
13	C	-1.8517420	0.1828300	-0.0733420
14	C	-1.9841420	-1.2017880	0.0231820
15	C	-2.9768130	1.0030320	-0.0035300
16	C	-3.2433200	-1.7606540	0.1888010
17	H	-1.1090070	-1.8339870	-0.0329720
18	C	-4.2324660	0.4403730	0.1628000
19	H	-2.8513400	2.0748700	-0.0811840
20	C	-4.3651190	-0.9415760	0.2587560
21	H	-3.3500160	-2.8348070	0.2626560
22	H	-5.1068870	1.0752730	0.2171600
23	H	-5.3458040	-1.3812500	0.3879650

Maleic anhydride

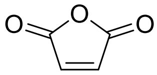

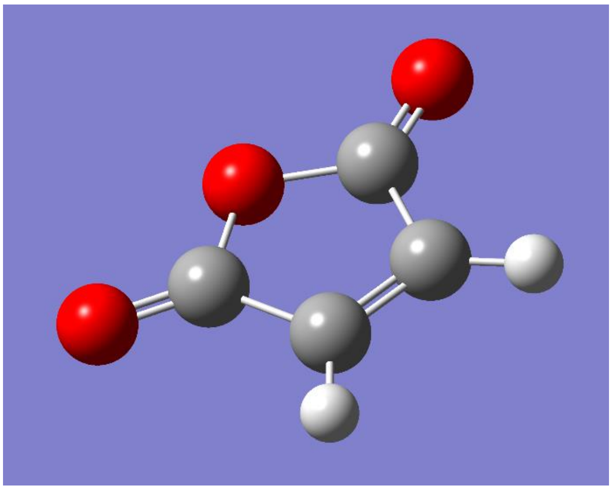

-379.034458

Tag	Symbol	X	Y	Z
1	C	-1.1181280	-0.1540300	-0.0000350
2	C	-0.6640310	1.2570590	0.0001180
3	C	0.6640920	1.2568210	-0.0001740
4	C	1.1176140	-0.1546830	0.0001980
5	O	0.0001280	-0.9568210	-0.0001480
6	H	-1.3530370	2.0864070	0.0001230
7	H	1.3533600	2.0860070	-0.0002090
8	O	2.2206050	-0.6091320	0.0000400
9	O	-2.2204330	-0.6094740	0.0000380

Methyl vinyl ether (MVE)

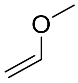

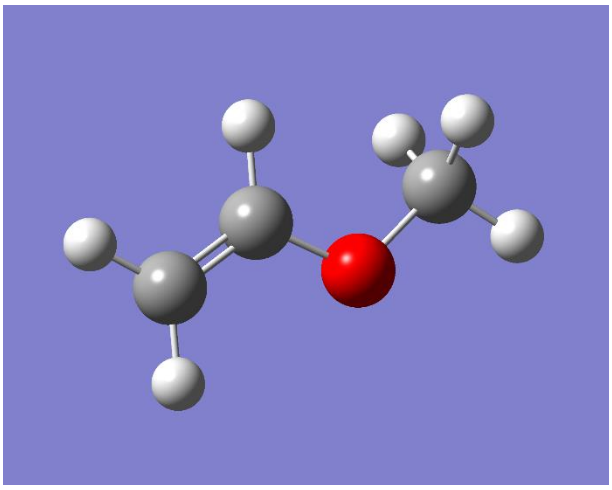

-192.906332

Tag	Symbol	X	Y	Z
1	C	-0.6048470	0.3426370	-0.0000660
2	H	-0.3392610	1.3978640	-0.0001560
3	C	-1.8695660	-0.0646060	0.0000620
4	H	-2.1246360	-1.1164330	0.0001680
5	H	-2.6648650	0.6658640	0.0000720
6	O	0.4413780	-0.5096870	-0.0001110
7	C	1.7146710	0.1128180	0.0000770
8	H	2.4617060	-0.6767220	-0.0000860
9	H	1.8472790	0.7311220	-0.8909020
10	H	1.8472040	0.7307030	0.8913520

Methyl vinyl ether (MVE) radical

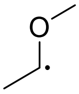

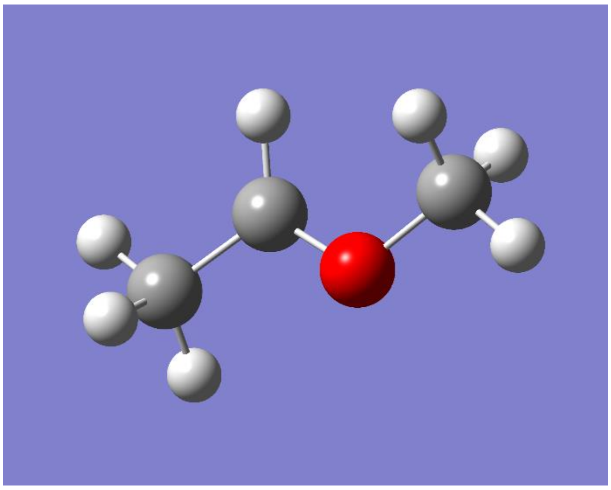

-193.463984

Tag	Symbol	X	Y	Z
1	C	-0.4950420	-0.4382330	0.0807040
2	H	-0.2403700	-1.4541550	-0.2026180
3	C	-1.8710320	0.0903360	-0.0080110
4	H	-2.0317400	0.8927450	0.7168400
5	H	-2.0944430	0.5064080	-1.0002870
6	H	-2.5939820	-0.7002630	0.1898220
7	O	0.4943100	0.4803350	-0.0209830
8	C	1.7970310	-0.0661810	0.0017070
9	H	2.5043290	0.7532340	-0.1012930
10	H	1.9806390	-0.5878880	0.9448800
11	H	1.9353400	-0.7682930	-0.8258820

MVE-MVE radical

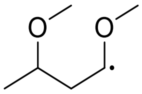

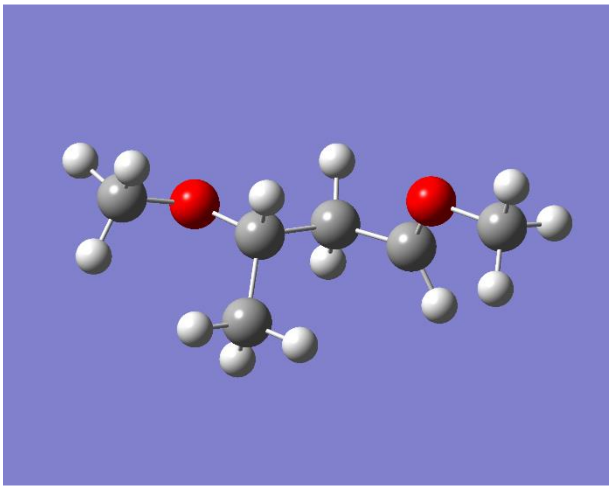

-386.382824

Tag	Symbol	X	Y	Z
1	C	0.8701350	-0.0416580	-0.0173670
2	C	-0.1388400	0.6586560	0.8983220
3	H	-0.0776430	1.7317810	0.6866780
4	O	2.1050200	0.6198880	0.1792700
5	C	3.1058200	0.2413400	-0.7305480
6	H	3.9194050	0.9612910	-0.6509960
7	H	3.5054820	-0.7554590	-0.5186080
8	H	2.7276460	0.2515550	-1.7604670
9	C	0.9633010	-1.5269810	0.2781840
10	H	1.6431430	-2.0351010	-0.4061730
11	H	1.3143970	-1.6801920	1.3017580
12	H	-0.0198010	-1.9901350	0.1752700
13	C	-1.5272870	0.1787820	0.7370110
14	H	-1.9749760	-0.5885230	1.3576250
15	H	0.1769700	0.5070560	1.9334620
16	H	0.5563690	0.1018950	-1.0617710
17	C	-3.3913570	-0.1393020	-0.6164890
18	H	-3.3842060	-1.2218840	-0.4605400
19	H	-4.0748320	0.3202650	0.1023420
20	H	-3.7306160	0.0761760	-1.6266010
21	O	-2.0890160	0.3921430	-0.4751020

Transition state for addition of MVE radical to MVE

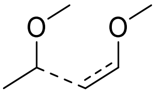

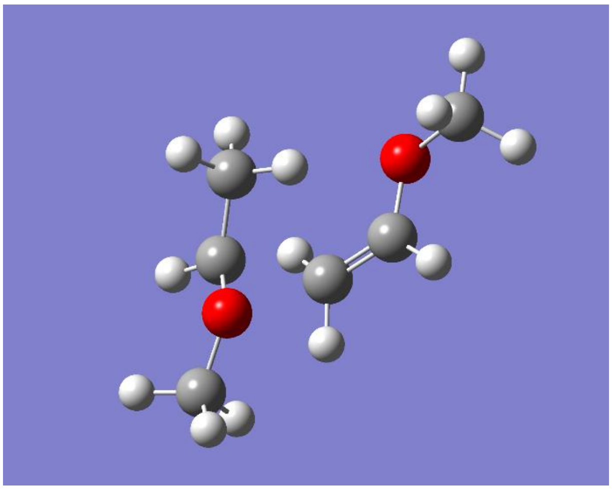

-386.340831

Tag	Symbol	X	Y	Z
1	C	1.2806800	0.5461930	0.4792980
2	C	-0.1547680	-1.1401970	0.7399270
3	H	-0.2341660	-0.9378010	1.8017250
4	O	2.0259010	0.2760900	-0.6295730
5	C	3.0857510	-0.6268070	-0.4071360
6	H	3.6107060	-0.7655470	-1.3496140
7	H	3.7825780	-0.2290700	0.3377600
8	H	2.7156620	-1.5950260	-0.0583550
9	C	0.3642390	1.6975150	0.2978260
10	H	0.9116920	2.6327330	0.1350860
11	H	-0.2761610	1.5290110	-0.5739060
12	H	-0.2774360	1.8169540	1.1707340
13	C	-1.2378600	-0.9360900	-0.0569330
14	H	-1.2859150	-1.2643520	-1.0910360
15	H	0.5723960	-1.8791390	0.4322770
16	H	1.8219560	0.4467100	1.4195850
17	C	-3.1620490	0.2361560	-0.6425070
18	H	-2.6410410	0.9219920	-1.3182850
19	H	-3.5513500	-0.6051790	-1.2238280
20	H	-3.9928040	0.7587640	-0.1739270
21	O	-2.3024090	-0.2124240	0.3831900

N-vinyl pyrrolidone (NVP)

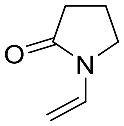

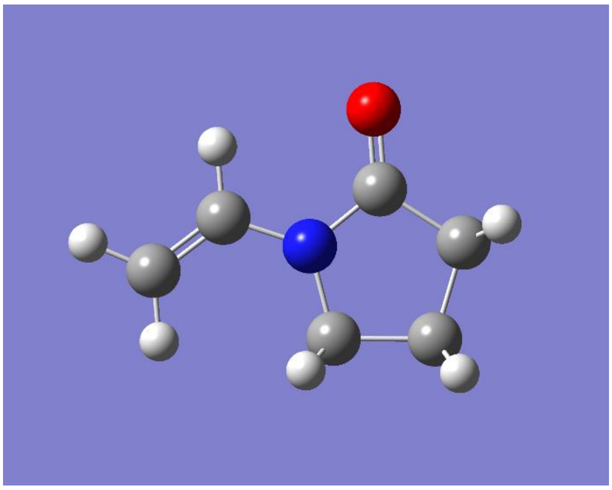

-363.635294

Tag	Symbol	X	Y	Z
1	C	-1.6954010	0.4549720	0.0001470
2	H	-1.7847690	1.5340690	0.0009110
3	C	-2.7566180	-0.3522460	-0.0000900
4	H	-2.6667060	-1.4307880	-0.0006820
5	H	-3.7506350	0.0710290	0.0004990
6	N	-0.3800680	0.0207490	0.0000390
7	C	-0.0175820	-1.3831160	-0.0001120
8	C	0.6831330	0.8863040	-0.0000510
9	C	1.5223840	-1.3926180	0.0001050
10	H	-0.4389050	-1.8723830	0.8809010
11	C	1.9550630	0.0768890	0.0000670
12	H	1.8984570	-1.9145970	0.8764880
13	H	2.5395510	0.3552640	0.8766380
14	O	0.5897260	2.1003030	-0.0001830
15	H	-0.4386840	-1.8719170	-0.8814960
16	H	1.8986450	-1.9146930	-0.8761410
17	H	2.5398370	0.3552330	-0.8763210

N-vinyl pyrrolidone (NVP) radical

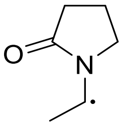

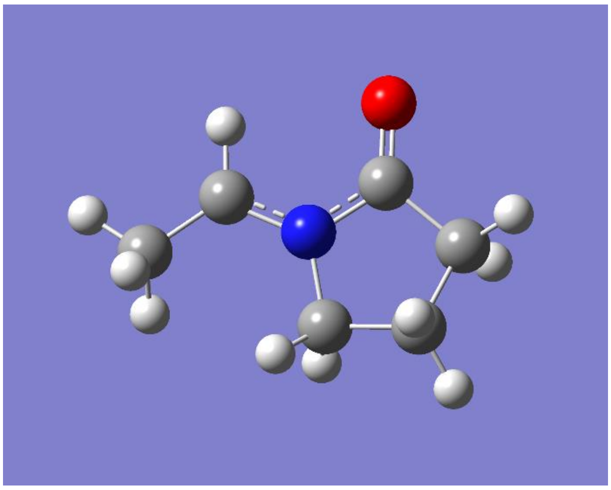

-364.197703

Tag	Symbol	X	Y	Z
1	C	1.5918700	0.6051400	0.0118290
2	H	1.6403630	1.6787770	-0.0667210
3	C	2.7814840	-0.2748820	-0.0303550
4	H	2.7660430	-1.0320880	0.7603090
5	H	2.8707180	-0.8175060	-0.9806580
6	H	3.6870590	0.3164320	0.0926280
7	N	0.3264120	0.0778250	0.0068030
8	C	0.0333480	-1.3434990	0.1635530
9	C	-0.8109160	0.8497370	0.0033410
10	C	-1.4365800	-1.4380950	-0.2407380
11	H	0.6870560	-1.9428470	-0.4681130
12	H	0.1917720	-1.6415430	1.2040970
13	C	-1.9984840	-0.0725550	0.1386330
14	O	-0.8113400	2.0709160	-0.0617080
15	H	-1.5059810	-1.5787260	-1.3197460
16	H	-1.9412570	-2.2689030	0.2467140
17	H	-2.8263180	0.2644640	-0.4816070
18	H	-2.3279540	-0.0452460	1.1815590

NVP-NVP radical

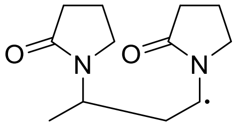

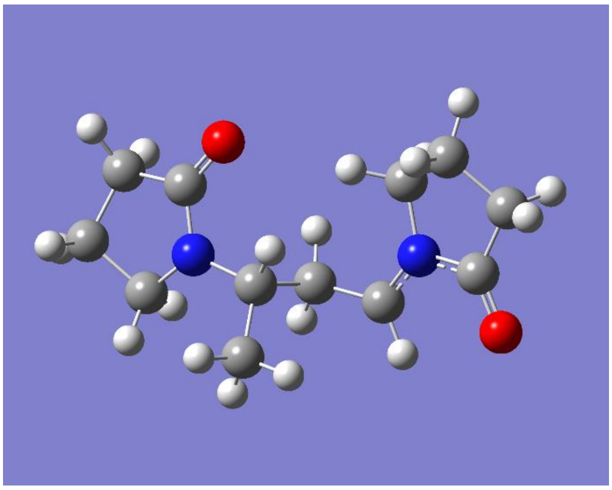

-727.842537

Tag	Symbol	X	Y	Z
1	C	-0.8538080	-0.7563400	0.4402130
2	C	-0.1235240	-0.7914180	-0.9235160
3	H	-0.2726490	0.1828800	-1.4013750
4	C	-0.8681010	-2.1213810	1.1014870
5	H	-1.4371420	-2.1008460	2.0317740
6	H	-1.3057640	-2.8729850	0.4405130
7	H	0.1526190	-2.4299790	1.3287140
8	C	1.3182280	-1.1050790	-0.8011340
9	H	1.7105370	-2.1090060	-0.8386710
10	H	-0.6087250	-1.5445840	-1.5499770
11	H	-0.3335430	-0.0438160	1.0851120
12	N	-2.1970290	-0.2328510	0.2663120
13	C	-3.3180870	-1.0282000	-0.2133080
14	C	-2.4113680	1.1022820	0.1485790
15	C	-4.4885570	-0.0513790	-0.0921590
16	H	-3.1554540	-1.3421700	-1.2506920
17	H	-3.4557930	-1.9204190	0.3955370
18	C	-3.8372600	1.3096170	-0.3123660
19	O	-1.5697490	1.9701880	0.3479120
20	H	-5.2867310	-0.2735210	-0.7964780
21	H	-4.8957000	-0.1064950	0.9179160
22	H	-3.7958040	1.5736200	-1.3727170
23	H	-4.3071660	2.1341050	0.2190670
24	N	2.2009740	-0.1547850	-0.3542810
25	C	1.9312150	1.2830670	-0.3434950
26	C	3.5130460	-0.4393190	-0.0519800
27	C	3.0705240	1.8338840	0.5102050
28	H	1.9680010	1.6676180	-1.3673580
29	H	0.9446380	1.4958780	0.0668810
30	C	4.2153870	0.8643470	0.2395600
31	O	3.9911130	-1.5640050	-0.0648260
32	H	3.3040520	2.8664390	0.2617720
33	H	2.7881700	1.7885550	1.5625980
34	H	4.7806700	1.1466570	-0.6537830
35	H	4.9210890	0.7515690	1.0597470

Transition state for addition of NVP radical to NVP

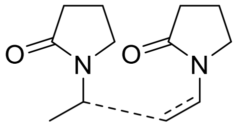

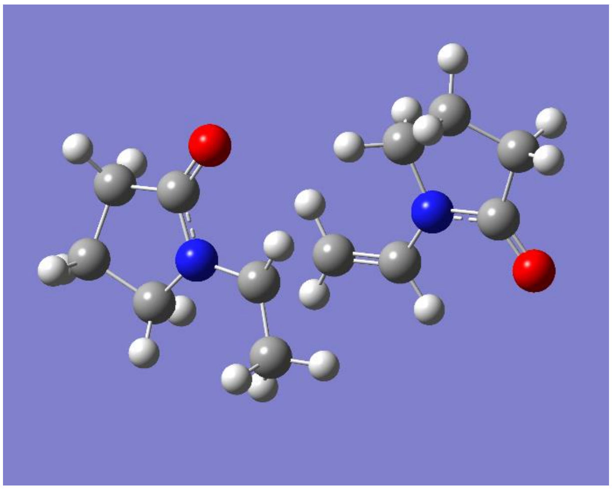

-727.805699

Tag	Symbol	X	Y	Z
1	C	1.1038120	0.7943490	0.7597880
2	C	-0.0682530	0.7632300	-1.1015070
3	H	0.2394150	-0.2665280	-1.2433240
4	C	1.0717860	2.2276120	1.1550030
5	H	1.6242360	2.4151960	2.0828810
6	H	1.4955590	2.8713030	0.3805620
7	H	0.0377320	2.5346870	1.3154250
8	C	-1.3730750	1.0714190	-0.8447420
9	H	-1.7595470	2.0804420	-0.8843670
10	H	0.5521930	1.5195150	-1.5659300
11	H	0.4759470	0.0928660	1.2932640
12	N	2.3093570	0.2334280	0.3638440
13	C	3.4096560	0.9956520	-0.2142290
14	C	2.4552160	-1.1162320	0.1809400
15	C	4.5319100	-0.0386880	-0.2920050
16	H	3.1255720	1.3706690	-1.2033400
17	H	3.6638550	1.8468700	0.4151180
18	C	3.7910040	-1.3581650	-0.4804800
19	O	1.6079580	-1.9480560	0.4811600
20	H	5.2379500	0.1825150	-1.0888070
21	H	5.0741050	-0.0512670	0.6537770
22	H	3.6029140	-1.5720870	-1.5364930
23	H	4.2895570	-2.2239650	-0.0497240
24	N	-2.2862420	0.1417060	-0.3716170
25	C	-2.0002870	-1.2822200	-0.2617440
26	C	-3.5949550	0.4482200	-0.1050550
27	C	-3.1679420	-1.7998290	0.5779790
28	H	-1.9746280	-1.7342930	-1.2585490
29	H	-1.0301740	-1.4454370	0.2093150
30	C	-4.3043670	-0.8392320	0.2442490
31	O	-4.0767370	1.5677250	-0.1782180
32	H	-3.3986720	-2.8400880	0.3613640
33	H	-2.9152020	-1.7184100	1.6355110
34	H	-4.8587970	-1.1584910	-0.6428950
35	H	-5.0206220	-0.6834860	1.0478930

MVE-1a radical

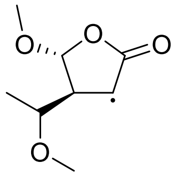

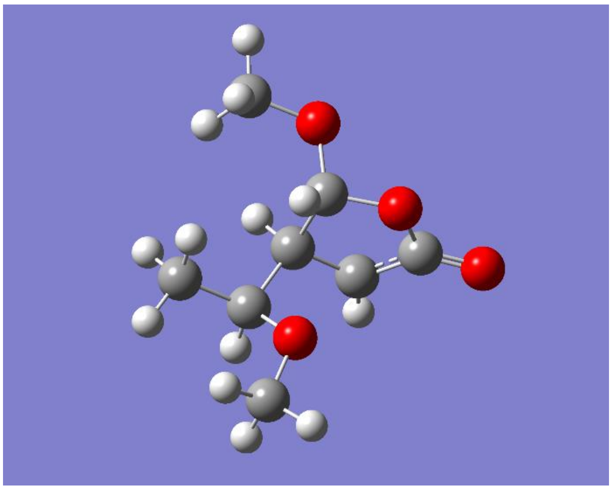

-612.918115

Tag	Symbol	X	Y	Z
1	C	0.8277250	-0.5442430	-0.4451780
2	C	0.0380350	-0.2089120	0.8411660
3	C	0.5832140	1.1141310	1.1899350
4	C	1.3924780	1.6097790	0.1064360
5	O	1.4594180	0.6482780	-0.8554310
6	H	0.2444380	-0.9618400	1.6076480
7	H	0.3745410	1.6833530	2.0803740
8	O	1.9426320	2.6795710	-0.0259740
9	O	1.8363400	-1.4761310	-0.2181200
10	C	-1.4797000	-0.1657560	0.6163250
11	H	-1.9481530	0.2520950	1.5187200
12	C	-2.0533050	-1.5465200	0.3530420
13	H	-3.1377900	-1.5077010	0.2525400
14	H	-1.6383560	-1.9762900	-0.5611390
15	H	-1.8192330	-2.2089200	1.1877030
16	O	-1.6771470	0.7269590	-0.4595440
17	H	0.1816690	-0.8795520	-1.2631460
18	C	-3.0271050	1.0410250	-0.7085340
19	H	-3.0481980	1.8974450	-1.3798430
20	H	-3.5605220	0.2126180	-1.1831040
21	H	-3.5452700	1.3066280	0.2203020
22	C	1.3421270	-2.7973270	-0.1939230
23	H	2.1895080	-3.4622810	-0.0465150
24	H	0.6277040	-2.9507070	0.6229720
25	H	0.8489020	-3.0473240	-1.1395780
23	H	2.1895080	-3.4622810	-0.0465150
24	H	0.6277040	-2.9507070	0.6229720
25	H	0.8489020	-3.0473240	-1.1395780

Transition state for addition of MVE radical to 1a

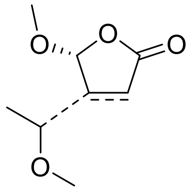

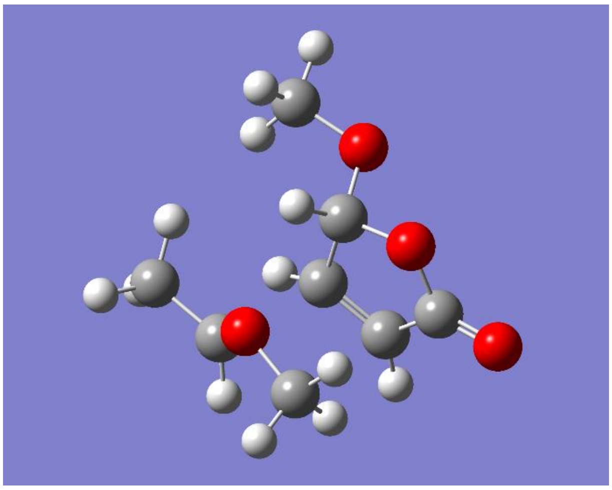

-612.884866

Tag	Symbol	X	Y	Z
1	C	-0.8292210	-0.5125190	-0.3609590
2	C	-0.3870970	-0.1145690	1.0310560
3	C	0.6167890	-0.9566420	1.3918470
4	C	1.0078370	-1.7287540	0.2252330
5	O	0.1559140	-1.4141900	-0.8019440
6	H	-1.0397620	0.4408090	1.6893330
7	H	1.1077200	-1.0528240	2.3465870
8	O	1.9092660	-2.5166220	0.0644730
9	O	-2.0541490	-1.1821120	-0.3469240
10	C	0.4987720	1.9454110	0.4401360
11	H	1.1790140	2.0044500	1.2839470
12	C	-0.7372870	2.7594430	0.4085820
13	H	-0.5358040	3.7682000	0.0321200
14	H	-1.4849340	2.3081650	-0.2514120
15	H	-1.1602640	2.8462740	1.4086650
16	O	1.0580350	1.7036710	-0.7556090
17	H	-0.8777430	0.3215120	-1.0698590
18	C	2.3768220	1.1698960	-0.7298090
19	H	2.5044600	0.5541000	-1.6163110
20	H	3.1054750	1.9828070	-0.7373030
21	H	2.5291800	0.5609820	0.1623080
22	C	-3.1411710	-0.2804770	-0.3300930
23	H	-4.0560200	-0.8678520	-0.3476750
24	H	-3.1384340	0.3419200	0.5717320
25	H	-3.1180710	0.3747510	-1.2080520

NVP-1a radical

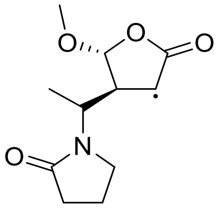

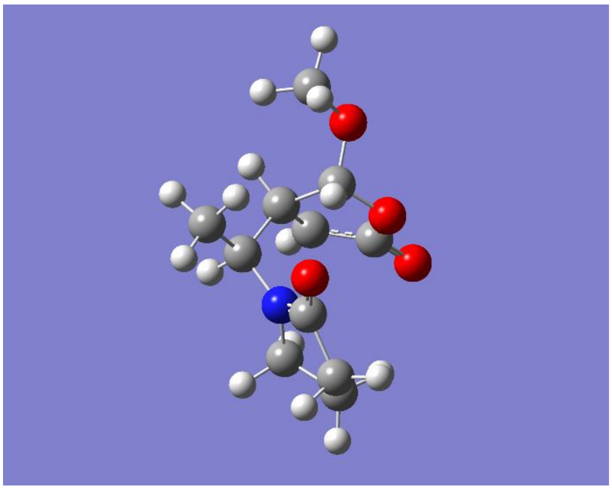

-783.641759

Tag	Symbol	X	Y	Z
1	C	1.4754460	0.0312820	-0.4766970
2	C	1.1019840	0.0130040	1.0201780
3	C	0.8243580	1.4371550	1.2645060
4	C	0.6510180	2.1137820	0.0018170
5	O	0.9481340	1.2361040	-0.9971990
6	H	1.9397070	-0.3637520	1.6138230
7	H	0.6938960	1.9225760	2.2173690
8	O	0.2857460	3.2442140	-0.2304220
9	O	2.8532510	0.0729160	-0.6688670
10	C	-0.1169600	-0.9024490	1.3122380
11	H	-0.4292210	-0.7010080	2.3404340
12	C	0.2848630	-2.3665300	1.2184050
13	H	-0.5880090	-3.0130400	1.3055560
14	H	0.7811390	-2.5965330	0.2776710
15	H	0.9670620	-2.5907900	2.0395450
16	H	1.0347850	-0.7982960	-1.0371720
17	N	-1.2533610	-0.5273430	0.4820230
18	C	-2.2478460	0.4058870	1.0024140
19	C	-1.6230040	-1.0676500	-0.7124660
20	C	-3.0248550	0.8067170	-0.2458560
21	H	-2.8830700	-0.0943250	1.7412820
22	H	-1.7640490	1.2531500	1.4915910
23	C	-2.9533500	-0.4539070	-1.0977890
24	O	-1.0056450	-1.9050550	-1.3551820
25	H	-4.0376090	1.1330100	-0.0219810
26	H	-2.4988440	1.6208770	-0.7484920
27	H	-3.7350270	-1.1691140	-0.8267230
28	H	-3.0027990	-0.2873010	-2.1709600
29	C	3.4353030	-1.2115290	-0.5945340
30	H	4.4999510	-1.1006700	-0.7847860
31	H	3.2996940	-1.6633230	0.3948010
32	H	3.0022860	-1.8800680	-1.3460580

Transition state for addition of NVP radical to 1a

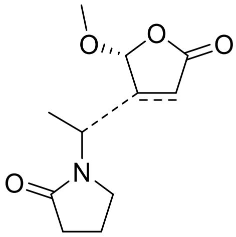

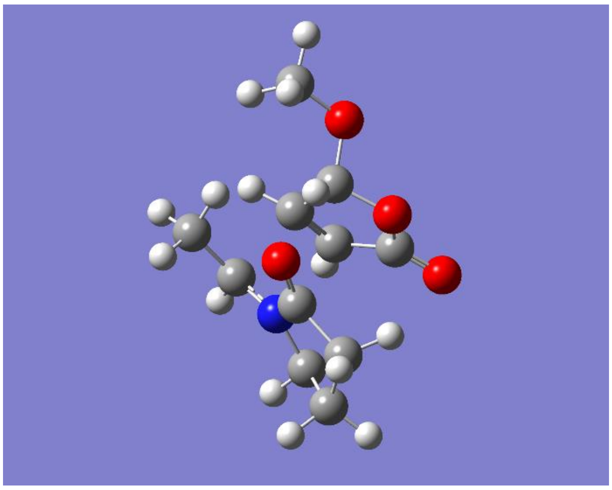

-783.610911

Tag	Symbol	X	Y	Z
1	C	1.5327920	-0.0342390	-0.4141520
2	C	1.3891260	0.1625120	1.0819180
3	C	1.1746100	1.4949090	1.2881280
4	C	0.9413890	2.1290770	0.0045760
5	O	1.1005180	1.1824760	-0.9755990
6	H	1.8568750	-0.5217540	1.7768030
7	H	1.1491910	2.0373990	2.2190630
8	O	0.6423340	3.2660450	-0.2751190
9	O	2.8642140	-0.2319520	-0.7823330
10	C	-0.4894700	-0.9804690	1.4213280
11	H	-0.6114400	-0.5630910	2.4122120
12	C	0.0193050	-2.3781550	1.3318630
13	H	-0.8073720	-3.0947140	1.3468830
14	H	0.5811760	-2.5699890	0.4182560
15	H	0.6590340	-2.5765250	2.1911980
16	H	0.9179510	-0.8407340	-0.8258270
17	N	-1.4266470	-0.4895130	0.5354230
18	C	-2.1743840	0.7320390	0.8438280
19	C	-1.7300350	-0.9697040	-0.7260580
20	C	-3.3574390	0.6652930	-0.1151540
21	H	-2.4592140	0.7338780	1.8947950
22	H	-1.5547490	1.6115720	0.6505190
23	C	-2.7648420	-0.0472960	-1.3235990
24	O	-1.2238270	-1.9453870	-1.2511950
25	H	-4.1500680	0.0580660	0.3232090
26	H	-3.7578050	1.6499160	-0.3422590
27	H	-3.4753970	-0.6037570	-1.9296550
28	H	-2.2332320	0.6520990	-1.9768960
29	C	3.2466310	-1.5875720	-0.6730300
30	H	4.2843010	-1.6606690	-0.9892100
31	H	3.1666320	-1.9516530	0.3571580
32	H	2.6286390	-2.2212750	-1.3181320

MVE-2a radical

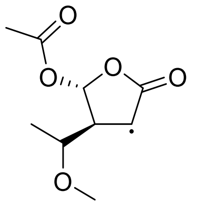

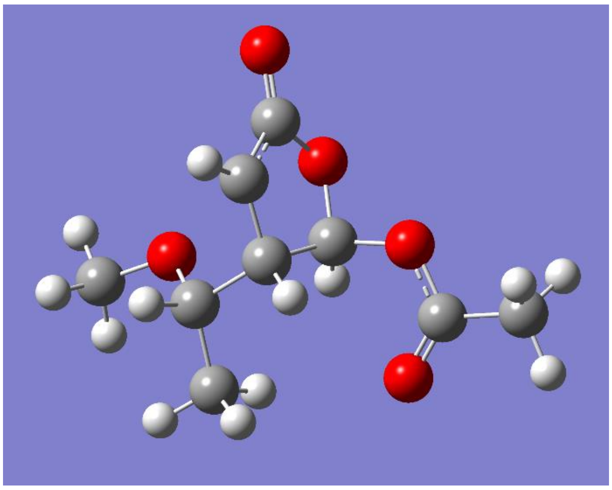

-726.206964

Tag	Symbol	X	Y	Z
1	C	-0.4558310	-0.2807100	-0.4765220
2	C	0.2625870	0.0806030	0.8344840
3	C	0.7959030	-1.2372940	1.2297480
4	C	0.7349520	-2.1350260	0.1047810
5	O	0.0731910	-1.5005850	-0.9120050
6	H	-0.4403180	0.5040180	1.5548000
7	H	1.2613490	-1.4919760	2.1667800
8	O	1.1712220	-3.2535170	-0.0291100
9	O	-1.8379800	-0.5408740	-0.2402970
10	C	-2.6436600	0.5290790	-0.1429310
11	C	-4.0655190	0.1323010	0.0823120
12	H	-4.6886650	1.0183830	0.1497450
13	H	-4.4027680	-0.5018880	-0.7371320
14	H	-4.1413040	-0.4490410	1.0010660
15	O	-2.2264750	1.6600110	-0.2318630
16	C	1.4041470	1.0909910	0.6239180
17	H	1.9811060	1.1351250	1.5585840
18	C	0.9043940	2.4811560	0.2808640
19	H	1.7326450	3.1893270	0.2516960
20	H	0.4021570	2.4885850	-0.6868870
21	H	0.1961730	2.8207570	1.0364600
22	O	2.2135310	0.5342650	-0.3904710
23	H	-0.3514140	0.4599360	-1.2657460
24	C	3.4111520	1.2378560	-0.6241920
25	H	4.0521450	0.6031300	-1.2330660
26	H	3.2374630	2.1752980	-1.1594500
27	H	3.9247710	1.4601970	0.3183410

Transition state for addition of MVE radical to 2a

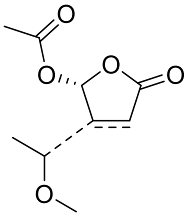

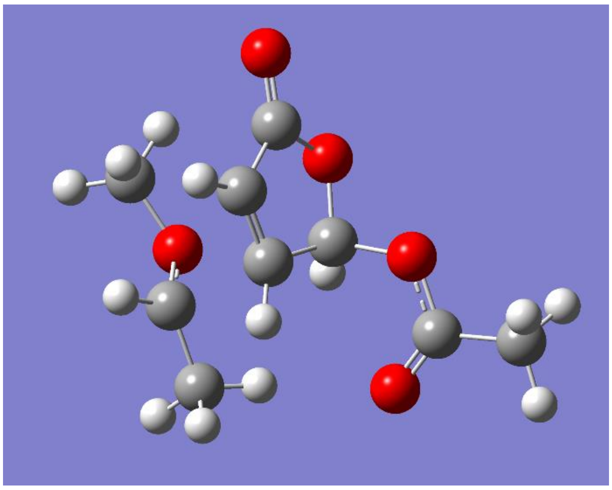

-726.174076

Tag	Symbol	X	Y	Z
1	C	-0.4468190	-0.3078660	-0.3420960
2	C	0.0906460	-0.0387480	1.0400190
3	C	0.8718640	-1.0970400	1.3738270
4	C	1.0478070	-1.9299030	0.1973090
5	O	0.2857450	-1.3962730	-0.8187630
6	H	-0.3848490	0.6722230	1.6986450
7	H	1.3516430	-1.3098690	2.3150040
8	O	1.7184810	-2.9145480	0.0114330
9	O	-1.8067280	-0.7477690	-0.2835760
10	C	-2.7371120	0.2075920	-0.1332770
11	C	-4.1176980	-0.3617720	-0.1379510
12	H	-4.8437780	0.4310660	0.0109920
13	H	-4.3007100	-0.8626020	-1.0884960
14	H	-4.2092950	-1.1079630	0.6507830
15	O	-2.4511210	1.3762350	-0.0133230
16	C	1.5310900	1.7302670	0.3905870
17	H	2.1399870	1.6352390	1.2840640
18	C	0.5986360	2.8668690	0.2331590
19	H	1.1436420	3.7788550	-0.0382510
20	H	-0.1320640	2.6725760	-0.5532260
21	H	0.0645970	3.0560890	1.1633840
22	O	2.0678310	1.2587690	-0.7441840
23	H	-0.3745030	0.5210470	-1.0442240
24	C	3.2338630	0.4557850	-0.6027000
25	H	3.2775720	-0.2221560	-1.4515440
26	H	4.1204200	1.0927850	-0.5966280
27	H	3.2000050	-0.1197130	0.3235510

NVP-2a radical

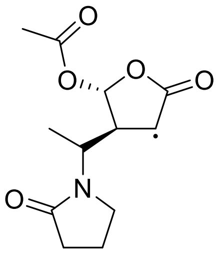

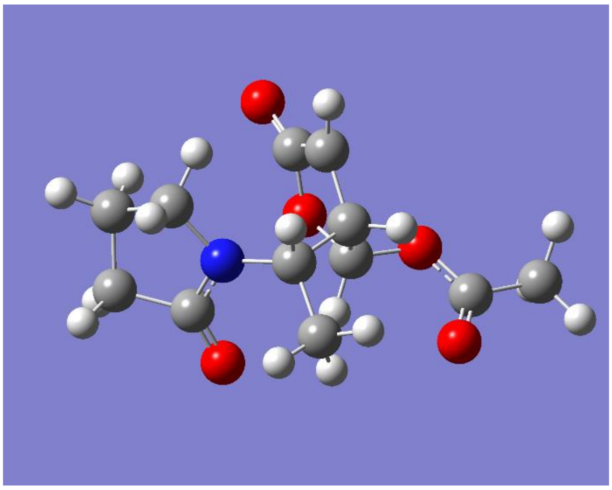

-896.930241

Tag	Symbol	X	Y	Z
1	C	1.0486660	0.2155890	-0.3682500
2	C	0.5914790	0.0692160	1.0902730
3	C	0.1204140	1.4395920	1.3616990
4	C	-0.1258600	2.1070700	0.1067130
5	O	0.3300930	1.2972800	-0.9005580
6	H	1.4206060	-0.2476370	1.7256610
7	H	-0.1064520	1.8756670	2.3199440
8	O	-0.6533900	3.1692550	-0.1254530
9	O	2.4162940	0.6139880	-0.4259520
10	C	3.3296540	-0.3596940	-0.2714220
11	C	4.7218780	0.1664440	-0.3903640
12	H	5.4344280	-0.6378690	-0.2378210
13	H	4.8602790	0.6061570	-1.3778720
14	H	4.8804940	0.9539130	0.3459640
15	O	3.0176200	-1.5083540	-0.0629380
16	C	-0.5518330	-0.9711770	1.2604710
17	H	-0.9070040	-0.8624410	2.2882070
18	C	-0.0453460	-2.3944440	1.0864540
19	H	-0.8784750	-3.0950160	1.1513900
20	H	0.4558610	-2.5418870	0.1347030
21	H	0.6597680	-2.6172930	1.8876990
22	H	0.8844680	-0.6631710	-0.9859720
23	N	-1.6949390	-0.6332030	0.4241280
24	C	-2.8324690	0.0800830	0.9958220
25	C	-1.9200680	-1.0381420	-0.8561370
26	C	-3.6039650	0.5352550	-0.2384220
27	H	-3.4225400	-0.5924820	1.6272640
28	H	-2.4930060	0.9134580	1.6141480
29	C	-3.3064480	-0.5687600	-1.2453250
30	O	-1.1503720	-1.6755170	-1.5604860
31	H	-4.6632310	0.6792840	-0.0397080
32	H	-3.1852060	1.4785740	-0.5943840
33	H	-3.9849990	-1.4182000	-1.1272610
34	H	-3.3289930	-0.2580520	-2.2868520

Transition state for addition of NVP radical to 2a

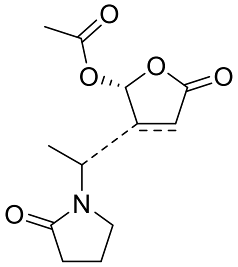

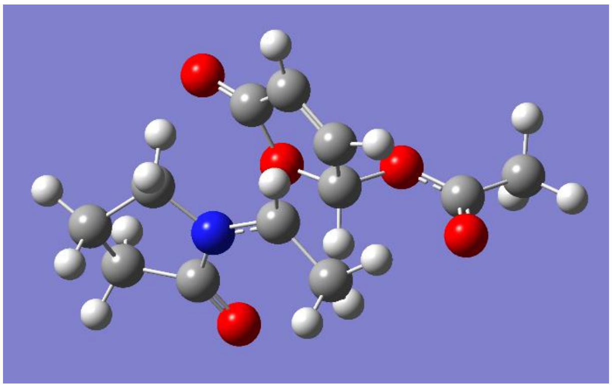

-896.900512

Tag	Symbol	X	Y	Z
1	C	1.1371470	0.2867120	-0.2485170
2	C	0.7814260	0.2650060	1.2191310
3	C	0.1545670	1.4496590	1.4801020
4	C	-0.1652940	2.0880920	0.2190720
5	O	0.3892450	1.3331080	-0.7930570
6	H	1.3390630	-0.3482850	1.9117440
7	H	-0.1463490	1.8562030	2.4316420
8	O	-0.8049580	3.0804330	-0.0344290
9	O	2.5106060	0.6494210	-0.4255200
10	C	3.4067930	-0.3330450	-0.2429180
11	C	4.7997020	0.1312460	-0.5156470
12	H	5.5017380	-0.6708120	-0.3109850
13	H	4.8799160	0.4394520	-1.5580500
14	H	5.0274820	0.9986330	0.1029600
15	O	3.0838350	-1.4470840	0.0985210
16	C	-0.8213110	-1.3083400	1.2070540
17	H	-1.0259350	-1.1386930	2.2563590
18	C	-0.0788050	-2.5543170	0.8674530
19	H	-0.7803870	-3.3926080	0.7904940
20	H	0.4581540	-2.5009460	-0.0750040
21	H	0.6288980	-2.7762500	1.6646730
22	H	0.9414730	-0.6282190	-0.8013690
23	N	-1.8224160	-0.8040100	0.4049020
24	C	-2.8650160	0.0600690	0.9650910
25	C	-1.9098770	-0.8911880	-0.9746840
26	C	-3.9112660	0.1063880	-0.1439910
27	H	-3.2352820	-0.3675110	1.8954700
28	H	-2.4593670	1.0531390	1.1765230
29	C	-3.0705910	-0.0300850	-1.4065310
30	O	-1.1536520	-1.5233870	-1.6877730
31	H	-4.5827100	-0.7475710	-0.0502430
32	H	-4.5018170	1.0182810	-0.1112340
33	H	-3.5828880	-0.4627730	-2.2620460
34	H	-2.6505290	0.9349200	-1.7108770

MVE-2d radical

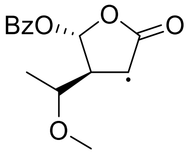

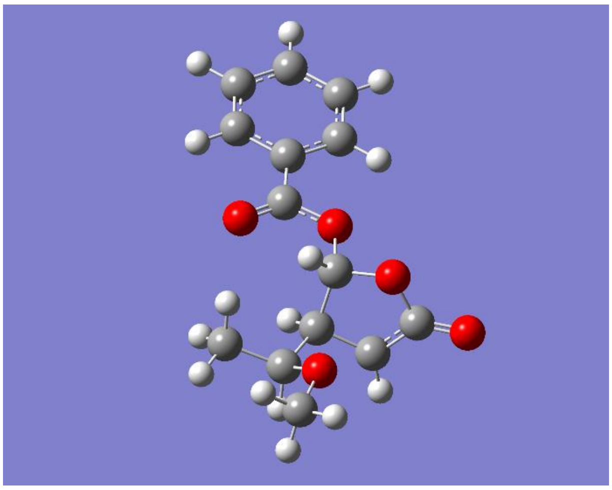

-917.730925

Tag	Symbol	X	Y	Z
1	C	-0.9029670	0.2590430	-0.5211210
2	C	-1.5695560	-0.0892980	0.8206480
3	C	-2.0554940	1.2389790	1.2406890
4	C	-2.0242750	2.1379850	0.1158410
5	O	-1.4211780	1.4911210	-0.9305700
6	H	-0.8453050	-0.5302200	1.5084490
7	H	-2.4724530	1.5017660	2.1980290
8	O	-2.4405480	3.2660070	0.0019250
9	O	0.4949770	0.4871530	-0.3460070
10	C	1.2743150	-0.6005660	-0.2779580
11	O	0.8250760	-1.7234620	-0.3416410
12	C	-2.7413640	-1.0737550	0.6588520
13	H	-3.2769790	-1.1081310	1.6182230
14	C	-2.2876860	-2.4732480	0.2909990
15	H	-3.1312790	-3.1636140	0.2994080
16	H	-1.8316280	-2.4890310	-0.6992730
17	H	-1.5521370	-2.8292660	1.0120880
18	O	-3.5820730	-0.4960680	-0.3175280
19	H	-1.0568790	-0.4773410	-1.3061640
20	C	-4.8025260	-1.1747510	-0.5021100
21	H	-5.4551530	-0.5260130	-1.0831450
22	H	-4.6700210	-2.1144340	-1.0451390
23	H	-5.2808850	-1.3883980	0.4607720
24	C	2.7127370	-0.2757640	-0.1183050
25	C	3.1708090	1.0401770	-0.0737920
26	C	3.6122080	-1.3354570	-0.0131780
27	C	4.5274880	1.2908390	0.0760690
28	H	2.4697030	1.8586930	-0.1581380
29	C	4.9665420	-1.0808510	0.1367860
30	H	3.2358660	-2.3491250	-0.0510640
31	C	5.4238130	0.2327250	0.1815660
32	H	4.8857440	2.3112270	0.1098770
33	H	5.6657610	-1.9025660	0.2183100
34	H	6.4813520	0.4320790	0.2984170

Transition state for addition of MVE radical to 2d

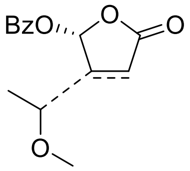

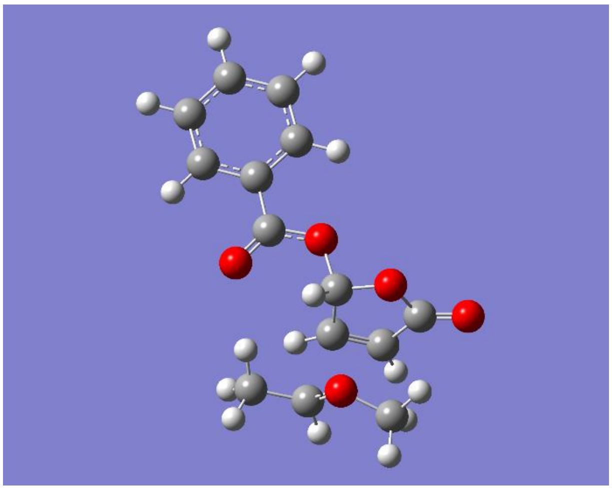

-917.699258

Tag	Symbol	X	Y	Z
1	C	-0.8743080	0.2170300	-0.3208160
2	C	-1.4548160	-0.0316050	1.0477160
3	C	-2.1120510	1.0985580	1.4096210
4	C	-2.1747750	1.9844120	0.2606610
5	O	-1.4663410	1.3985690	-0.7665530
6	H	-1.0774830	-0.8174300	1.6842570
7	H	-2.5759290	1.3350620	2.3530870
8	O	-2.7226000	3.0464080	0.1019460
9	O	0.5281570	0.4916690	-0.2374090
10	C	1.3323710	-0.5696880	-0.0981130
11	O	0.9058000	-1.6997440	-0.0047670
12	C	-3.0871340	-1.6008220	0.3214350
13	H	-3.6824220	-1.4731300	1.2199380
14	C	-2.2915580	-2.8295130	0.1139960
15	H	-2.9402270	-3.6660650	-0.1722470
16	H	-1.5578130	-2.6951400	-0.6819760
17	H	-1.7664380	-3.1060490	1.0272730
18	O	-3.5605580	-1.0200520	-0.7904260
19	H	-1.0328040	-0.5748640	-1.0507620
20	C	-4.6325080	-0.1014170	-0.6127030
21	H	-4.6007770	0.6110890	-1.4332310
22	H	-5.5830930	-0.6378780	-0.6297580
23	H	-4.5376710	0.4286740	0.3361020
24	C	2.7709350	-0.2080260	-0.0720900
25	C	3.2017330	1.1106100	-0.2099810
26	C	3.6985850	-1.2352490	0.0928410
27	C	4.5594680	1.3964300	-0.1829070
28	H	2.4785780	1.9037270	-0.3389010
29	C	5.0539320	-0.9455580	0.1197780
30	H	3.3429880	-2.2516770	0.1976200
31	C	5.4840420	0.3706650	-0.0182610
32	H	4.8965950	2.4189210	-0.2906840
33	H	5.7749740	-1.7421510	0.2476560
34	H	6.5423760	0.5971350	0.0022380

MVE-1a-1a radical

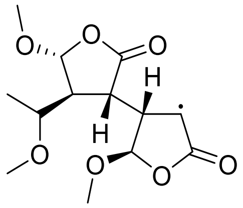

-1032.369776

Tag	Symbol	X	Y	Z
1	C	-1.8879390	1.2565940	-0.2697440
2	C	-1.3263720	-0.1572620	-0.5012960
3	C	-0.0704630	-0.1635760	0.3648160
4	C	-0.3559720	0.9084840	1.3952270
5	O	-1.3422620	1.7065870	0.9540750
6	H	-1.1000410	-0.2823000	-1.5611040
7	H	0.0821680	-1.1197310	0.8692870
8	O	0.1985980	1.1020080	2.4428450
9	O	-1.4860640	2.1579980	-1.2504580
10	C	-2.3090780	-1.2400150	-0.0783680
11	H	-1.7829290	-2.2061190	-0.1022460
12	C	-3.5167080	-1.3161000	-0.9961280
13	H	-4.1821670	-2.1279570	-0.7034080
14	H	-4.0865000	-0.3847500	-0.9823710
15	H	-3.1890440	-1.5084820	-2.0190240
16	O	-2.6488480	-0.9410820	1.2606250
17	H	-2.9771200	1.2734470	-0.1647570
18	C	-3.3637780	-1.9602470	1.9198470
19	H	-3.3765640	-1.7213170	2.9814310
20	H	-4.3957740	-2.0308400	1.5651710
21	H	-2.8760640	-2.9317860	1.7805270
22	C	1.2172280	0.2167950	-0.4081240
23	C	1.5238890	-0.7634630	-1.4685390
24	H	1.1162730	1.2355960	-0.7910700
25	H	1.0107350	-0.8922220	-2.4062400
26	C	2.6162010	-1.6069350	-1.0429960
27	C	2.4712400	0.1115670	0.4726110
28	H	2.2658260	0.1071680	1.5458160
29	O	3.0524200	-1.1633290	0.1671330
30	O	3.0988860	-2.5740880	-1.5862950
31	O	3.3354640	1.1266830	0.1292480
32	C	-2.3152960	2.0945410	-2.3928060
33	H	-3.3505000	2.3409920	-2.1352310
34	H	-1.9417330	2.8196980	-3.1111180
35	H	-2.2965960	1.1002310	-2.8533740
36	C	4.4905540	1.1829840	0.9506290
37	H	5.1192420	0.3023150	0.8081260
38	H	5.0479810	2.0720110	0.6667970
39	H	4.2062220	1.2556350	2.0046320

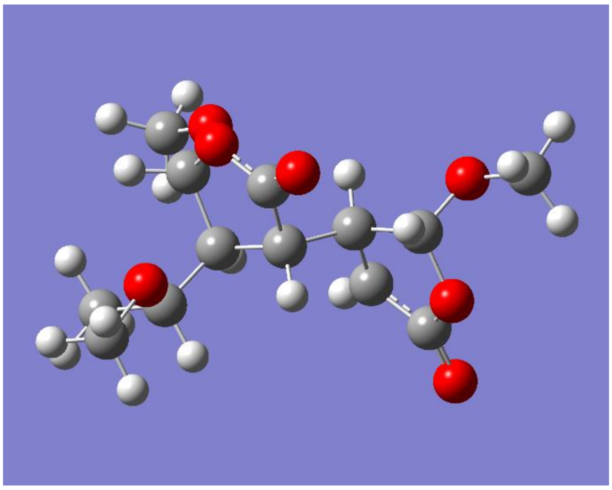

Transition state for addition of MVE-1a radical to 1a

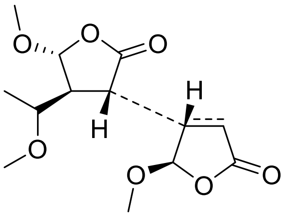

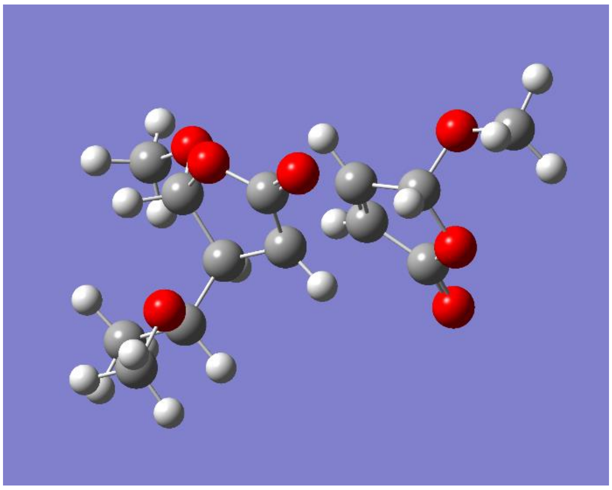

-1032.333018

Tag	Symbol	X	Y	Z
1	C	-1.7879310	0.0040800	1.2889590
2	C	-1.3759460	0.4506300	-0.1284330
3	C	-0.2332900	-0.4579530	-0.4010250
4	C	-0.3448830	-1.5971900	0.5059390
5	O	-1.2332720	-1.2822650	1.4800420
6	H	-1.0918470	1.5051980	-0.1187240
7	H	0.2034490	-0.5798620	-1.3820750
8	O	0.2401800	-2.6530430	0.4923410
9	O	-1.2507380	0.8276720	2.2755230
10	C	-2.4984700	0.2711450	-1.1534930
11	H	-2.0740290	0.4253230	-2.1558770
12	C	-3.6246050	1.2658270	-0.9341470
13	H	-4.3880470	1.1693120	-1.7058210
14	H	-4.0992100	1.1170670	0.0380290
15	H	-3.2327220	2.2830160	-0.9802120
16	O	-2.9167790	-1.0722660	-1.0340110
17	H	-2.8712580	-0.0801850	1.4158390
18	C	-3.8189200	-1.4866370	-2.0336320
19	H	-3.8913810	-2.5713110	-1.9838420
20	H	-4.8161040	-1.0627620	-1.8863500
21	H	-3.4582150	-1.2005130	-3.0283210
22	C	1.5407540	0.4041950	0.5703780
23	C	1.6242320	1.6486370	0.0234270
24	H	1.1736630	0.1745480	1.5611810
25	H	1.1246480	2.5523140	0.3322310
26	C	2.5068230	1.5812020	-1.1440320
27	C	2.5924930	-0.4537160	-0.0931410
28	H	2.2350290	-1.4174000	-0.4680200
29	O	2.9966020	0.3155380	-1.2322830
30	O	2.7926550	2.4359310	-1.9433270
31	O	3.6389040	-0.6266420	0.7829060
32	C	4.6149950	-1.5429580	0.3102530
33	H	5.1146770	-1.1585410	-0.5808810
34	H	5.3463400	-1.6751230	1.1033610
35	H	4.1531900	-2.5072550	0.0791530
36	C	-2.0168090	2.0003210	2.4608600
37	H	-3.0426550	1.7524200	2.7523460
38	H	-1.5480050	2.5762630	3.2545430
39	H	-2.0485960	2.6126000	1.5524380

MVE-1a-MVE radical

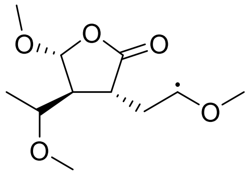

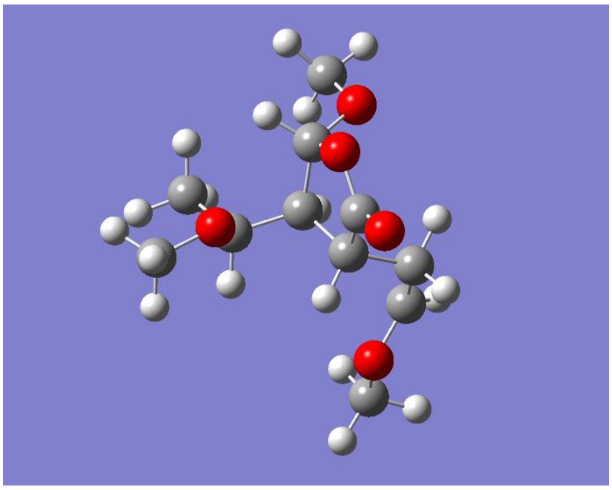

-805.835913

Tag	Symbol	X	Y	Z
1	C	1.4826140	-0.9291350	-0.2852640
2	C	0.2809340	0.0219470	-0.4151430
3	C	-0.5427140	-0.2977570	0.8260830
4	C	0.4437200	-0.9557800	1.7544870
5	O	1.5509580	-1.3104450	1.0726100
6	H	-0.2732140	-0.2178770	-1.3262350
7	H	-0.9282040	0.6037610	1.3031420
8	O	0.3182980	-1.1989520	2.9246090
9	O	1.3244120	-2.0993940	-1.0262370
10	C	0.7048000	1.4816740	-0.4688250
11	H	-0.2014690	2.1006830	-0.3818110
12	C	1.4188430	1.8337150	-1.7626600
13	H	1.6643500	2.8949500	-1.8034700
14	H	2.3446580	1.2642670	-1.8702270
15	H	0.7717350	1.6091020	-2.6122450
16	O	1.5091640	1.6929790	0.6753100
17	H	2.4357300	-0.4539590	-0.5390810
18	C	1.7880800	3.0457190	0.9450580
19	H	2.2211910	3.1027810	1.9420030
20	H	2.5008020	3.4687410	0.2311110
21	H	0.8709260	3.6459450	0.9228480
22	C	-1.7096620	-1.2686650	0.5604800
23	C	-2.7181870	-0.6789730	-0.3452330
24	H	-1.3255770	-2.1866060	0.1090710
25	H	-3.3018790	-1.2659050	-1.0439070
26	H	-2.1476810	-1.5404180	1.5308160
27	C	-4.2130880	1.0334090	-0.8375860
28	H	-5.0646150	0.3501240	-0.8939790
29	H	-4.5425690	1.9871440	-0.4335520
30	H	-3.8067290	1.1834200	-1.8410900
31	O	-3.2193290	0.5181960	0.0284220
32	C	1.6698830	-1.9108820	-2.3813040
33	H	2.7157430	-1.5992830	-2.4775950
34	H	1.5317270	-2.8605530	-2.8922560
35	H	1.0357190	-1.1570130	-2.8618170

Transition state for addition of MVE-1a radical to MVE

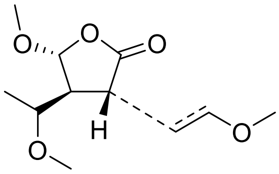

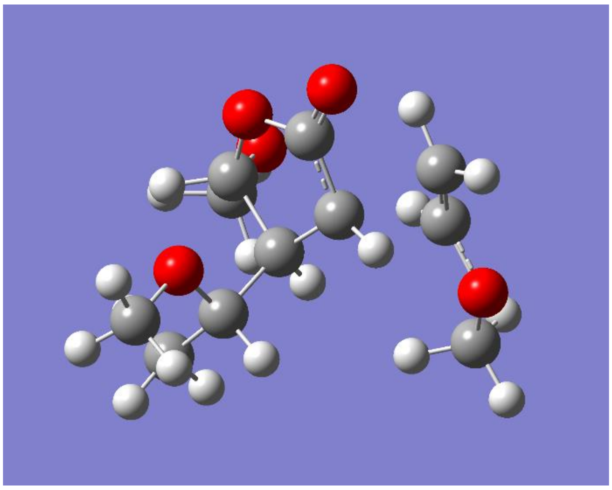

-805.801188

Tag	Symbol	X	Y	Z
1	C	0.5987360	0.2374060	1.2770180
2	C	0.3125880	-0.3228610	-0.1312320
3	C	0.0586440	0.9296590	-0.8901860
4	C	0.6448740	2.0254610	-0.1552830
5	O	0.9429210	1.5900960	1.1075210
6	H	-0.5605120	-0.9819750	-0.0946680
7	H	-0.0031500	0.9613830	-1.9669470
8	O	0.8443500	3.1755600	-0.4775640
9	O	-0.5284510	0.2050800	2.1042120
10	C	1.4804030	-1.1402210	-0.6840030
11	H	1.2977250	-1.3198470	-1.7537800
12	C	1.6095820	-2.4765160	0.0289140
13	H	2.4204020	-3.0741450	-0.3875390
14	H	1.8014990	-2.3322370	1.0946400
15	H	0.6848970	-3.0454550	-0.0835450
16	O	2.6349040	-0.3378530	-0.5477450
17	H	1.4348260	-0.2658090	1.7746370
18	C	3.7619140	-0.8287830	-1.2313060
19	H	4.5159000	-0.0436540	-1.2339770
20	H	4.1847540	-1.7148020	-0.7483150
21	H	3.5117440	-1.0815440	-2.2685280
22	C	-2.0497890	1.5087890	-0.5005620
23	C	-2.7203880	0.3368110	-0.3219010
24	H	-1.8588780	2.1093320	0.3775430
25	H	-2.8723060	-0.0997490	0.6613050
26	H	-2.0836090	2.0153370	-1.4556110
27	C	-3.6933200	-1.6571580	-1.0251600
28	H	-4.5923230	-1.5241070	-0.4203930
29	H	-3.9527160	-2.1590850	-1.9530190
30	H	-2.9711570	-2.2655740	-0.4737630
31	O	-3.1281430	-0.3996390	-1.3659680
32	C	-0.7651040	-1.0776880	2.6353030
33	H	-1.6202070	-1.0058720	3.3037990
34	H	-0.9923180	-1.8123720	1.8524630
35	H	0.1019330	-1.4351570	3.2024350

NVP-1a-1a radical

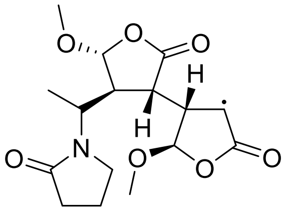

-1203.087675

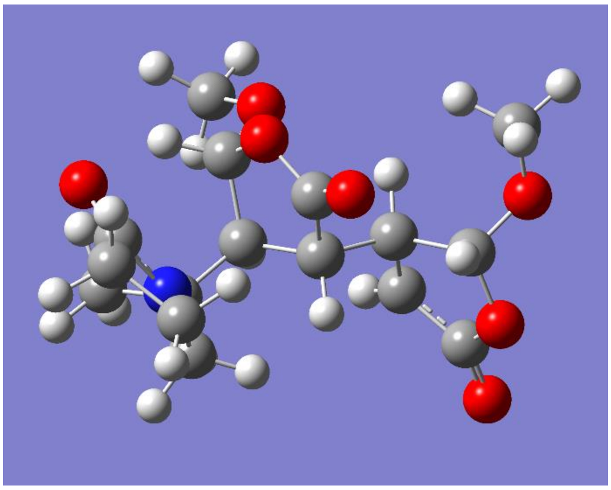

Tag	Symbol	X	Y	Z
1	C	0.8404780	1.6243480	-0.5958030
2	C	0.4562210	0.8955080	0.7013120
3	C	-0.4716770	-0.1975310	0.1798780
4	C	0.0725210	-0.4416900	-1.2132530
5	O	0.7486680	0.6465570	-1.6208780
6	H	-0.0549100	1.5970340	1.3633240
7	H	-0.4240820	-1.1162860	0.7657420
8	O	-0.0671220	-1.4086450	-1.9136940
9	O	-0.0568940	2.6406250	-0.9156810
10	C	1.6548040	0.3177400	1.4645150
11	H	1.2362960	-0.2666850	2.2887390
12	C	2.5190690	1.4147520	2.0715710
13	H	3.3976370	0.9854620	2.5540440
14	H	2.8604690	2.1332010	1.3293020
15	H	1.9372650	1.9427690	2.8285390
16	H	1.8654490	1.9995840	-0.5983510
17	N	2.3982600	-0.6570120	0.6631680
18	C	2.3460900	-2.0792300	1.0002100
19	C	3.4372520	-0.3697740	-0.1723470
20	C	3.0740130	-2.7311670	-0.1698840
21	H	2.8586150	-2.2593470	1.9515640
22	H	1.3152430	-2.4188220	1.1000230
23	C	4.1018230	-1.6749360	-0.5539610
24	O	3.7861230	0.7457140	-0.5301970
25	H	3.5078250	-3.6927820	0.0935350
26	H	2.3716140	-2.8789980	-0.9913570
27	H	5.0126620	-1.7588480	0.0455580
28	H	4.3875810	-1.6706390	-1.6030320
29	C	0.2846320	3.8578410	-0.2817870
30	H	-0.4601300	4.5968470	-0.5656110
31	H	0.2855510	3.7585670	0.8096100
32	H	1.2739600	4.1978990	-0.6031180
33	C	-2.8720740	-0.9045180	-0.4268880
34	C	-1.9527870	0.2401820	0.0587630
35	C	-2.5484340	0.5548430	1.3704350
36	C	-3.4709670	-0.4903190	1.7444550
37	O	-3.5406500	-1.3865050	0.7203060
38	H	-2.0117130	1.0853220	-0.6328090
39	H	-2.3307790	1.4008960	2.0001230
40	O	-4.0892280	-0.6451250	2.7714810
41	O	-3.8460690	-0.4448150	-1.3027780
42	H	-2.3174820	-1.7366720	-0.8710130
43	C	-3.3512660	-0.3004050	-2.6203210
44	H	-4.1717520	0.0536670	-3.2389950
45	H	-2.5339490	0.4282650	-2.6699160
46	H	-2.9899840	-1.2576570	-3.0079130

Transition state for addition of NVP-1a radical to 1a

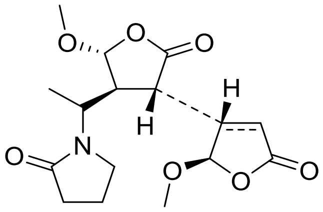

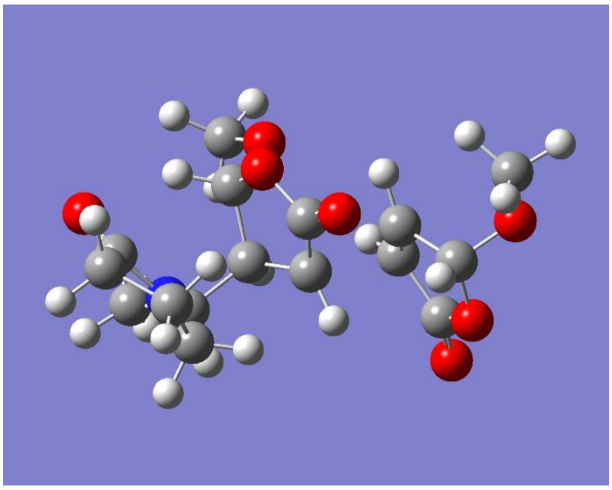

-1203.052246

Tag	Symbol	X	Y	Z
1	C	0.8471020	0.9851980	-1.1973910
2	C	0.5098410	0.9840630	0.3045370
3	C	-0.3296140	-0.2358050	0.4042920
4	C	0.0371570	-1.1052690	-0.7112840
5	O	0.7151540	-0.3626340	-1.6223270
6	H	-0.0275740	1.8969820	0.5718600
7	H	-0.6684500	-0.6523870	1.3424650
8	O	-0.2085920	-2.2744020	-0.8917550
9	O	-0.0639770	1.7364910	-1.9366710
10	C	1.7711000	0.9119940	1.1945100
11	H	1.4298660	0.7124780	2.2139100
12	C	2.4938640	2.2520180	1.1959230
13	H	3.4437550	2.1771250	1.7248610
14	H	2.6969940	2.6111850	0.1887560
15	H	1.8678040	2.9826920	1.7096210
16	H	1.8715100	1.3017450	-1.4095040
17	N	2.6086710	-0.2295170	0.8423880
18	C	2.5132320	-1.4649910	1.6157200
19	C	3.6634160	-0.2365790	-0.0194030
20	C	3.2942020	-2.4602790	0.7651120
21	H	2.9630840	-1.3256650	2.6044930
22	H	1.4710650	-1.7559080	1.7566060
23	C	4.3491580	-1.5807090	0.1070630
24	O	4.0074520	0.6800430	-0.7522670
25	H	3.7041400	-3.2787920	1.3517480
26	H	2.6331110	-2.8754830	0.0021100
27	H	5.2201760	-1.4415280	0.7536050
28	H	4.6980400	-1.9281540	-0.8621690
29	C	0.2544800	3.1139730	-1.9264630
30	H	-0.4924330	3.6270550	-2.5268950
31	H	0.2369230	3.5272650	-0.9116690
32	H	1.2459490	3.2862990	-2.3571230
33	C	-3.0769500	-0.9530170	0.2179480
34	C	-2.3221810	0.2119820	-0.3955760
35	C	-2.6860590	1.3341160	0.2866650
36	C	-3.4381970	0.9206750	1.4728270
37	O	-3.5902630	-0.4341270	1.4261780
38	H	-1.9831310	0.1910760	-1.4220450
39	H	-2.4628850	2.3634960	0.0557080
40	O	-3.8735920	1.5768650	2.3828270
41	O	-4.1509150	-1.3348600	-0.5743670
42	H	-2.4495820	-1.8215770	0.4445250
43	C	-3.7653140	-2.2273910	-1.6051880
44	H	-4.6647680	-2.4914110	-2.1552740
45	H	-3.0514350	-1.7656300	-2.2939500
46	H	-3.3164080	-3.1331400	-1.1870510

NVP-1a-NVP radical

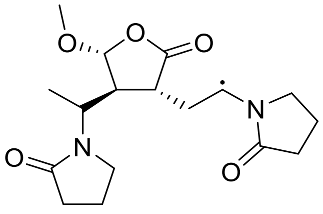

-1147.284728

Tag	Symbol	X	Y	Z
1	C	-1.7756850	1.5593150	0.4566260
2	C	-1.0509760	0.9733240	-0.7669100
3	C	0.2363540	0.4425880	-0.1477590
4	C	-0.1918100	0.1104050	1.2590990
5	O	-1.2900740	0.8313400	1.5721440
6	H	-0.8532300	1.7726810	-1.4856770
7	H	0.6139500	-0.4515930	-0.6427110
8	O	0.3244790	-0.6342250	2.0483470
9	O	-1.4747830	2.9026070	0.6721990
10	C	-1.8768880	-0.1110350	-1.4718430
11	H	-1.2126420	-0.5929790	-2.1952780
12	C	-3.0379930	0.5002490	-2.2454280
13	H	-3.6859250	-0.2761680	-2.6527690
14	H	-3.6469340	1.1545120	-1.6241460
15	H	-2.6352520	1.0833080	-3.0749290
16	H	-2.8585180	1.4153070	0.4200110
17	N	-2.2686090	-1.1871270	-0.5563650
18	C	-1.5828320	-2.4781030	-0.6058200
19	C	-3.4319760	-1.2571040	0.1511430
20	C	-2.1276340	-3.2009970	0.6203020
21	H	-1.8345930	-3.0015520	-1.5348750
22	H	-0.5016230	-2.3500190	-0.5696400
23	C	-3.5436040	-2.6528350	0.7265520
24	O	-4.2559600	-0.3630500	0.2825960
25	H	-2.0786080	-4.2828510	0.5222090
26	H	-1.5496020	-2.9032360	1.4964930
27	H	-4.2418140	-3.2076880	0.0933850
28	H	-3.9511400	-2.6229630	1.7341090
29	C	-2.3099470	3.7514230	-0.0871630
30	H	-2.0201970	4.7770430	0.1274350
31	H	-2.1995050	3.5713420	-1.1626640
32	H	-3.3608600	3.6081470	0.1845520
33	C	1.3638690	1.5131370	-0.1035910
34	H	1.5437670	1.8122500	-1.1407360
35	H	0.9763160	2.3910600	0.4151820
36	C	2.6130660	1.0708910	0.5557260
37	N	3.5209220	0.2573070	-0.0728330
38	C	3.4359450	-0.1856290	-1.4614450
39	C	4.5984810	-0.2954370	0.5847330
40	C	4.8491940	-0.6880030	-1.7511920
41	H	2.6965860	-0.9864330	-1.5539880
42	H	3.1404900	0.6361540	-2.1111030
43	C	5.3358280	-1.1781030	-0.3928420
44	O	4.8579980	-0.0975480	1.7610490
45	H	4.8592870	-1.4545350	-2.5224390
46	H	5.4671420	0.1457270	-2.0856100
47	H	5.0371380	-2.2132750	-0.2036210
48	H	6.4110690	-1.1075160	-0.2444940
49	H	2.8048800	1.2345140	1.6038800

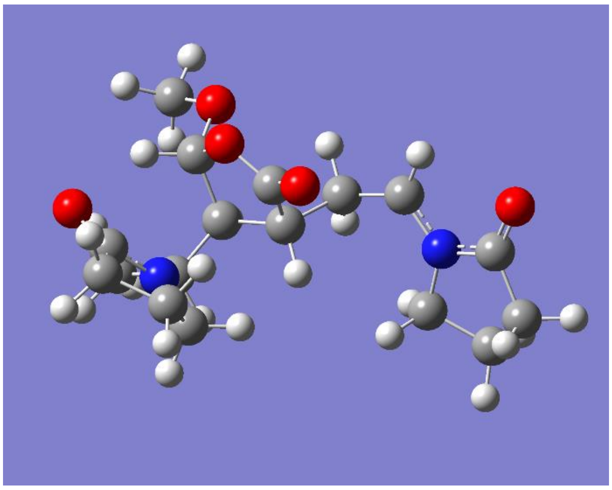

Transition state for addition of NVP-1a radical to NVP

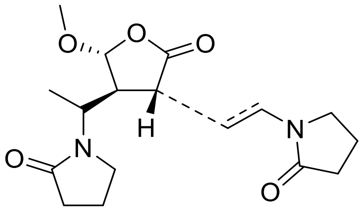

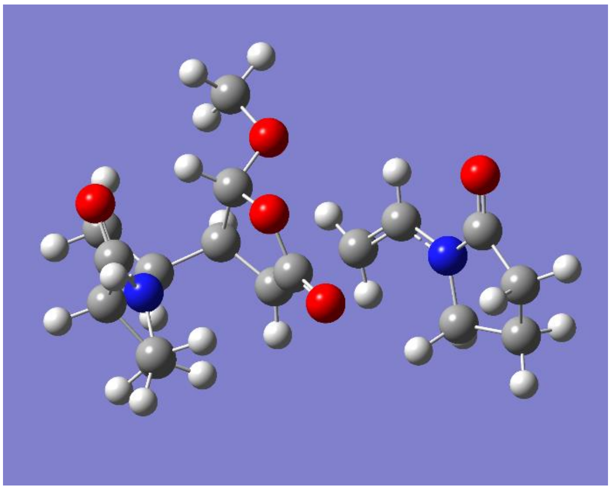

-1147.252871

Tag	Symbol	X	Y	Z
1	C	1.0391230	1.3069610	-0.5228520
2	C	1.2260620	0.9893300	0.9736220
3	C	0.1046460	0.0332850	1.1999310
4	C	-0.2159660	-0.5592050	-0.0802490
5	O	0.3168510	0.2197480	-1.0650360
6	H	1.1380320	1.9099580	1.5582140
7	H	0.0062060	-0.5543460	2.1003840
8	O	-0.8720100	-1.5422200	-0.3513780
9	O	0.2798160	2.4595700	-0.7334140
10	C	2.6159620	0.3886390	1.2713740
11	H	2.5823770	-0.0054110	2.2915720
12	C	3.6891910	1.4674710	1.2216890
13	H	4.6846940	1.0337500	1.3155760
14	H	3.6564520	2.0347270	0.2932370
15	H	3.5295030	2.1524010	2.0558720
16	H	1.9883130	1.3825860	-1.0624960
17	N	2.8878080	-0.7711850	0.4255240
18	C	2.6153950	-2.1150450	0.9286310
19	C	3.5647160	-0.7884590	-0.7548550
20	C	2.7081760	-2.9740760	-0.3269810
21	H	3.3658640	-2.3960630	1.6758710
22	H	1.6325390	-2.1598750	1.3982040
23	C	3.7520240	-2.2376160	-1.1553460
24	O	3.9680650	0.1834710	-1.3793020
25	H	2.9642410	-4.0082390	-0.1088820
26	H	1.7467840	-2.9530520	-0.8437280
27	H	4.7690600	-2.5254810	-0.8740950
28	H	3.6514940	-2.3547240	-2.2315570
29	C	1.0667710	3.6283630	-0.6549270
30	H	0.4176110	4.4766860	-0.8583210
31	H	1.5099750	3.7549220	0.3397670
32	H	1.8735940	3.6066160	-1.3954150
33	C	-1.6760020	1.2767920	1.4883640
34	H	-1.9212420	0.7646900	2.4092950
35	H	-1.0708400	2.1678990	1.5726960
36	C	-2.5208020	1.1898770	0.4090600
37	N	-3.4744120	0.2161370	0.2737980
38	C	-3.7395590	-0.8101550	1.2727640
39	C	-4.1452950	-0.0058600	-0.9163510
40	C	-5.0101110	-1.4810820	0.7494780
41	H	-2.8904880	-1.4979160	1.3171860
42	H	-3.8786510	-0.3580410	2.2545380
43	C	-4.9250790	-1.2868980	-0.7602760
44	O	-4.0640590	0.7107550	-1.8935880
45	H	-5.0716310	-2.5243470	1.0484090
46	H	-5.8840800	-0.9595950	1.1402400
47	H	-4.3343370	-2.0759650	-1.2347500
48	H	-5.8833060	-1.2283890	-1.2705740
49	H	-2.4007520	1.8180200	-0.4631910

MVE-2a-2a radical

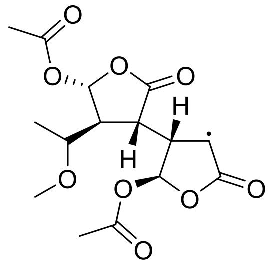

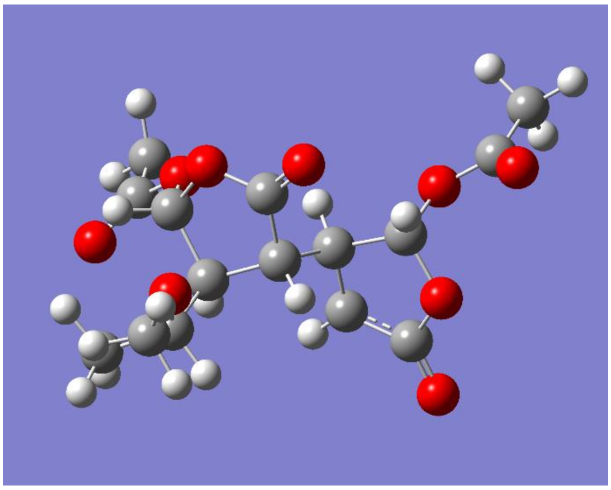

-1258.943300

Tag	Symbol	X	Y	Z
1	C	2.0724250	0.6672690	-0.7856400
2	C	1.6415490	-0.2559220	0.3586560
3	C	0.2605300	-0.7184080	-0.1031680
4	C	0.3356980	-0.5588270	-1.6081270
5	O	1.3565560	0.2658240	-1.9234890
6	H	1.6197890	0.3047860	1.2927340
7	H	0.0781480	-1.7676540	0.1372650
8	O	-0.3853240	-1.0212040	-2.4463820
9	O	1.6741340	2.0135740	-0.5509060
10	C	2.4773490	2.7397680	0.2503720
11	C	1.9965500	4.1458790	0.3853160
12	H	0.9756760	4.1508540	0.7665720
13	H	2.6490480	4.6952160	1.0563800
14	H	1.9839390	4.6201230	-0.5958770
15	O	3.4578330	2.2658940	0.7725350
16	C	2.5813810	-1.4467120	0.5169430
17	H	2.1166260	-2.1354600	1.2385020
18	C	3.9614870	-1.0671140	1.0198060
19	H	4.5489760	-1.9593470	1.2361830
20	H	4.5004060	-0.4687210	0.2844400
21	H	3.8773120	-0.4876670	1.9392060
22	O	2.6130690	-2.0701180	-0.7511810
23	H	3.1357520	0.6370420	-1.0108880
24	C	3.2655410	-3.3192980	-0.7666490
25	H	3.0316280	-3.8023340	-1.7132270
26	H	4.3505220	-3.2120930	-0.6859870
27	H	2.9112550	-3.9540930	0.0534730
28	C	-0.9138190	0.1359860	0.4374440
29	C	-1.0075000	0.0615320	1.9096640
30	H	-0.8175010	1.1601030	0.0700500
31	H	-0.3557230	0.5252530	2.6300110
32	C	-2.0969280	-0.8122490	2.2743090
33	C	-2.2712790	-0.4529620	0.0244090
34	H	-2.2418700	-1.1024860	-0.8461030
35	O	-2.7292710	-1.2072170	1.1273390
36	O	-2.4460250	-1.2043440	3.3612210
37	O	-3.1551530	0.6225160	-0.1955270
38	C	-4.3034310	0.3370930	-0.8641240
39	O	-4.5514350	-0.7659730	-1.2737620
40	C	-5.1589720	1.5507480	-1.0034410
41	H	-5.4288270	1.9175730	-0.0130680
42	H	-4.5992560	2.3386230	-1.5066800
43	H	-6.0544550	1.3079700	-1.5663990

Transition state for addition of MVE-2a radical to 2a

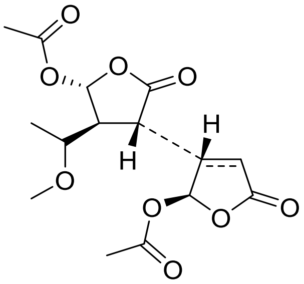

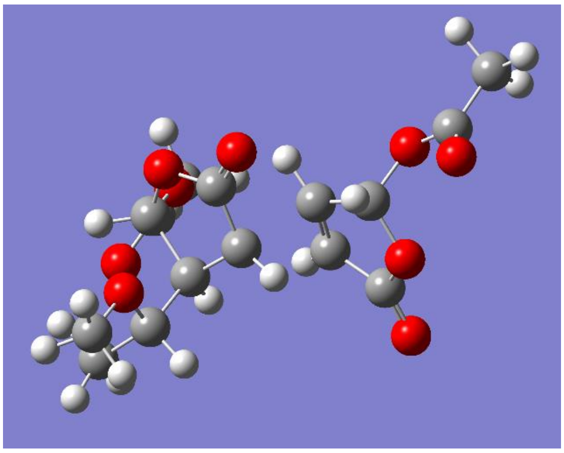

-1258.905372

Tag	Symbol	X	Y	Z
1	C	2.0731850	0.2665970	-1.0078540
2	C	1.7123510	-0.2124120	0.4050270
3	C	0.4147780	-0.8886510	0.1452210
4	C	0.3821880	-1.2446830	-1.2711230
5	O	1.3658490	-0.5471130	-1.9059310
6	H	1.6422570	0.6309710	1.0935060
7	H	-0.0760100	-1.5122290	0.8790050
8	O	-0.3677610	-1.9725100	-1.8695650
9	O	1.5816340	1.5849910	-1.2383770
10	C	2.2946340	2.5924180	-0.7005850
11	C	1.6903940	3.9215480	-1.0107300
12	H	0.6522120	3.9429310	-0.6784820
13	H	2.2566730	4.7074760	-0.5213710
14	H	1.6924990	4.0768690	-2.0895930
15	O	3.2962690	2.3946090	-0.0555000
16	C	2.7337420	-1.2092610	0.9675470
17	H	2.3038950	-1.6360940	1.8851490
18	C	4.0652780	-0.5614430	1.2971840
19	H	4.7210130	-1.2706170	1.8022330
20	H	4.5649540	-0.2081220	0.3944340
21	H	3.9122280	0.2893350	1.9609990
22	O	2.8446440	-2.2278430	-0.0047360
23	H	3.1334100	0.2237900	-1.2444250
24	C	3.6117320	-3.3364840	0.4062450
25	H	3.4360250	-4.1405160	-0.3056180
26	H	4.6810400	-3.1094350	0.4215420
27	H	3.3078770	-3.6726060	1.4041480
28	C	-1.1693720	0.6308820	-0.0155290
29	C	-0.9827050	1.3865030	1.1028820
30	H	-0.8968580	0.9112110	-1.0228030
31	H	-0.3115970	2.2176890	1.2503470
32	C	-1.8284480	0.8426610	2.1674790
33	C	-2.3486170	-0.2740000	0.2473000
34	H	-2.2151380	-1.3207400	-0.0199260
35	O	-2.5670850	-0.1800690	1.6384240
36	O	-1.9205230	1.1583610	3.3233100
37	O	-3.4530140	0.2557940	-0.4473870
38	C	-4.5285320	-0.5664390	-0.5834330
39	O	-4.5406140	-1.6844930	-0.1418120
40	C	-5.6283830	0.1110160	-1.3278660
41	H	-5.9457180	0.9957190	-0.7758440
42	H	-5.2634170	0.4414030	-2.2998550
43	H	-6.4638880	-0.5703800	-1.4514340

MVE-2a-MVE radical

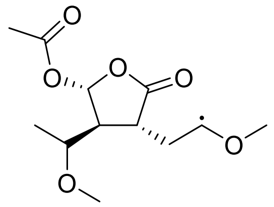

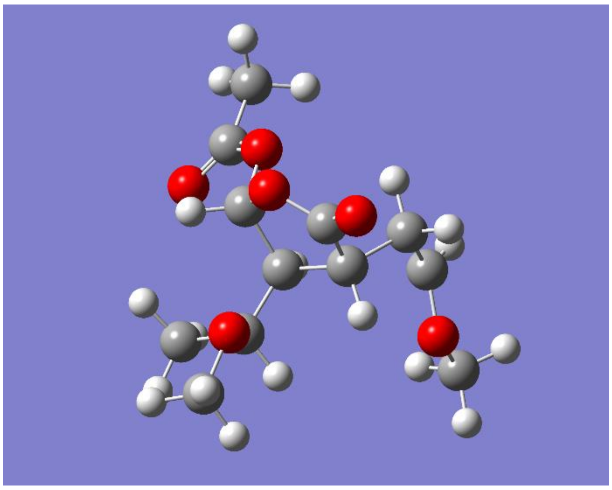

-919.124927

Tag	Symbol	X	Y	Z
1	C	-1.3042740	-0.5591760	0.4092010
2	C	0.0225770	-0.2538850	-0.2913240
3	C	0.9481790	0.0353170	0.8858370
4	C	0.2894600	-0.6842970	2.0333280
5	O	-0.9931840	-0.9810390	1.7094040
6	H	-0.0911720	0.6143380	-0.9436170
7	H	1.9418050	-0.3838890	0.7293270
8	O	0.7400440	-0.9527510	3.1117550
9	O	-2.0857970	0.6227300	0.5785520
10	C	-2.7841590	1.0349360	-0.4924750
11	C	-3.5479210	2.2854220	-0.2046010
12	H	-2.8520490	3.0807210	0.0634910
13	H	-4.1234070	2.5752090	-1.0780170
14	H	-4.2094170	2.1255470	0.6462550
15	O	-2.7653690	0.4390790	-1.5437660
16	C	0.5261320	-1.4325310	-1.1146260
17	H	1.5494780	-1.1871580	-1.4386260
18	C	-0.3208990	-1.7243010	-2.3395230
19	H	0.1431570	-2.4937970	-2.9568330
20	H	-1.3187970	-2.0619900	-2.0571930
21	H	-0.4233270	-0.8226860	-2.9437200
22	O	0.5852890	-2.5282290	-0.2215170
23	H	-1.9080800	-1.3238320	-0.0741520
24	C	1.2525520	-3.6564910	-0.7347310
25	H	1.4360740	-4.3382180	0.0936740
26	H	0.6563020	-4.1766460	-1.4897570
27	H	2.2135400	-3.3728860	-1.1799510
28	C	1.0796630	1.5311320	1.2363180
29	C	1.6544910	2.3072020	0.1173270
30	H	0.1001120	1.9464380	1.4839740
31	H	1.4215490	3.3499970	-0.0606070
32	H	1.6974580	1.6040860	2.1421460
33	C	3.3473090	2.6140150	-1.4480690
34	H	3.5524370	3.6302740	-1.1013520
35	H	4.2759940	2.1468370	-1.7653770
36	H	2.6530730	2.6555170	-2.2910860
37	O	2.8073430	1.8312210	-0.3992460

Transition state for addition of MVE-2a radical to MVE

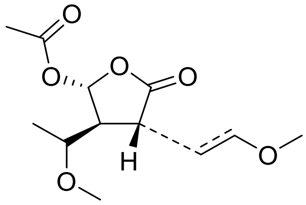

-919.087969

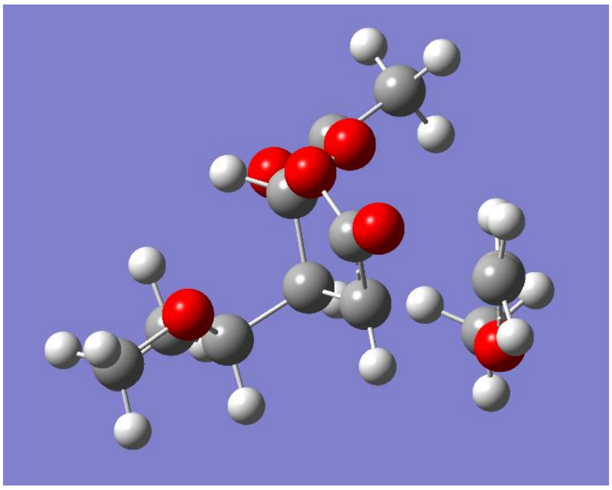

Tag	Symbol	X	Y	Z
1	C	-0.4802070	-0.3457850	-1.0978170
2	C	-0.5547060	-0.0649780	0.4113480
3	C	-0.6945240	1.4178360	0.4155620
4	C	-1.1606300	1.8230110	-0.8902420
5	O	-1.0331550	0.7564830	-1.7494640
6	H	0.3535190	-0.4246680	0.9032570
7	H	-1.0258140	1.9560560	1.2898340
8	O	-1.5816850	2.8839020	-1.2885320
9	O	0.8778500	-0.4017420	-1.5470160
10	C	1.5534470	-1.5252370	-1.2757630
11	C	2.9763370	-1.4687270	-1.7361840
12	H	3.6215020	-1.4193200	-0.8567450
13	H	3.2134760	-2.3886220	-2.2673920
14	H	3.1640400	-0.6071540	-2.3706650
15	O	1.0494960	-2.4673420	-0.7078570
16	C	-1.7575810	-0.7444780	1.0728160
17	H	-1.8567470	-0.3227750	2.0842950
18	C	-1.6055820	-2.2507460	1.1785380
19	H	-2.4265400	-2.6830480	1.7511640
20	H	-1.5865930	-2.7112490	0.1901740
21	H	-0.6727730	-2.4965720	1.6865720
22	O	-2.8864190	-0.3672370	0.3104810
23	H	-0.9952590	-1.2517130	-1.4112980
24	C	-4.1168530	-0.7514960	0.8744530
25	H	-4.9038120	-0.2103260	0.3521940
26	H	-4.3011170	-1.8243310	0.7671560
27	H	-4.1574600	-0.4938080	1.9395600
28	C	1.3152200	2.3625830	0.3819990
29	C	2.2168560	1.3929740	0.7031460
30	H	1.2937420	2.7083160	-0.6418210
31	H	2.7573580	0.8310040	-0.0521580
32	H	0.9713390	3.0494990	1.1428590
33	C	3.2190820	-0.1455340	2.1300150
34	H	4.1990350	-0.0110810	1.6671210
35	H	3.3435290	-0.3151020	3.1957190
36	H	2.7177210	-1.0091500	1.6829940
37	O	2.4271250	1.0243750	1.9748820

NVP-2a-2a radical

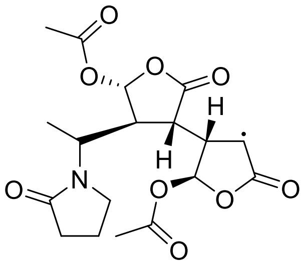

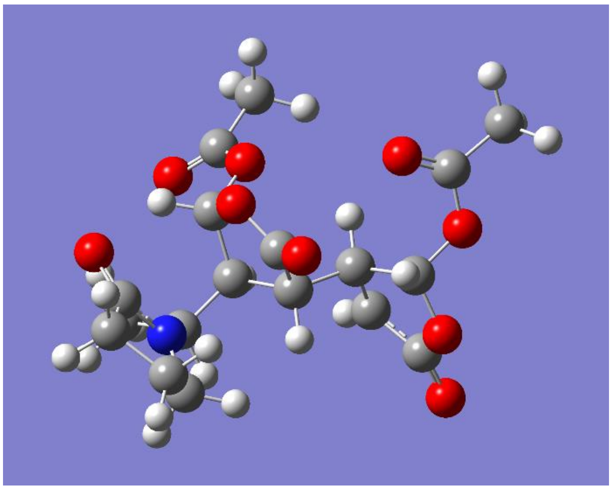

-1429.662339

Tag	Symbol	X	Y	Z
1	C	0.9526690	1.4527470	-0.3083320
2	C	0.8384460	0.4823200	0.8721410
3	C	-0.0679500	-0.6013960	0.2894080
4	C	0.2757210	-0.5400920	-1.1872340
5	O	0.7974720	0.6737540	-1.4692050
6	H	0.3901130	0.9966190	1.7231480
7	H	0.1646930	-1.5988460	0.6660750
8	O	0.1561500	-1.3945140	-2.0185870
9	O	-0.1218370	2.3873620	-0.3273120
10	C	0.0079670	3.4540990	0.4838710
11	C	-1.1425070	4.3948070	0.3444120
12	H	-1.0205200	5.2299550	1.0267190
13	H	-1.1949220	4.7545000	-0.6829120
14	H	-2.0740570	3.8702330	0.5572010
15	O	0.9559070	3.5898820	1.2198840
16	C	2.1889220	-0.0931210	1.3235060
17	H	1.9465740	-0.8502690	2.0746770
18	C	3.0756480	0.9503100	1.9874790
19	H	4.0277160	0.5014380	2.2734370
20	H	3.2772810	1.7964440	1.3363750
21	H	2.5827930	1.3172110	2.8882350
22	H	1.9014260	1.9775680	-0.3667690
23	N	2.8543910	-0.8384660	0.2527290
24	C	3.0087800	-2.2890080	0.3526210
25	C	3.6955640	-0.3005020	-0.6775980
26	C	3.5788040	-2.6634390	-1.0109600
27	H	3.6979380	-2.5393070	1.1665860
28	H	2.0514880	-2.7682290	0.5575610
29	C	4.4084160	-1.4388910	-1.3743320
30	O	3.8594530	0.8909360	-0.8932820
31	H	4.1488110	-3.5889520	-0.9835840
32	H	2.7599340	-2.7791650	-1.7221940
33	H	5.4161020	-1.4924950	-0.9527040
34	H	4.4984190	-1.2437110	-2.4402010
35	C	-2.4732210	-1.3844790	-0.1226710
36	C	-1.5724730	-0.3245240	0.5311480
37	C	-1.9091360	-0.5183250	1.9568590
38	C	-2.5703300	-1.7911850	2.1161910
39	O	-2.7546450	-2.3328080	0.8690690
40	H	-1.8179060	0.6698350	0.1576370
41	H	-1.6529760	0.1235590	2.7825450
42	O	-2.9075180	-2.3760050	3.1156680
43	O	-3.7296160	-0.8224310	-0.4808820
44	C	-3.7251330	-0.0415890	-1.5790200
45	C	-5.0937530	0.4431350	-1.9214030
46	H	-5.0441440	1.1111500	-2.7752980
47	H	-5.7312110	-0.4102740	-2.1526270
48	H	-5.5289580	0.9573160	-1.0649900
49	O	-2.7062600	0.2049460	-2.1791020
50	H	-2.0307870	-1.8794860	-0.9845470

Transition state for addition of NVP-2a radical to 2a

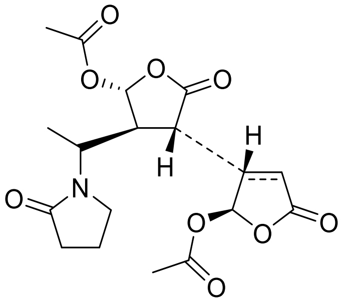

-1429.625275

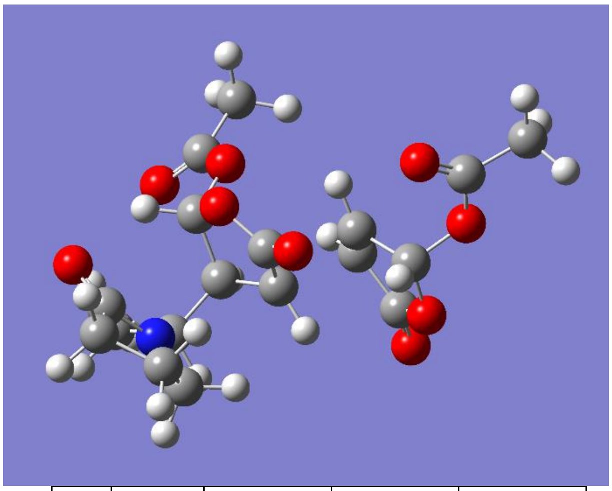

Tag	Symbol	X	Y	Z
1	C	1.1696510	1.0581550	-0.8710510
2	C	1.0031870	0.7098940	0.6128610
3	C	0.0797820	-0.4511100	0.5189410
4	C	0.3052240	-1.0558740	-0.7946300
5	O	0.9390300	-0.1356250	-1.5790890
6	H	0.6001510	1.5576900	1.1678580
7	H	-0.1671410	-1.0744760	1.3672130
8	O	0.0272190	-2.1560580	-1.1956480
9	O	0.1492230	1.9541210	-1.3038110
10	C	0.2921120	3.2456160	-0.9524110
11	C	-0.8335640	4.0805050	-1.4672180
12	H	-0.7232930	5.1032390	-1.1213520
13	H	-0.8336180	4.0537880	-2.5569910
14	H	-1.7850420	3.6686600	-1.1289100
15	O	1.2313230	3.6342620	-0.3004850
16	C	2.3360100	0.3026850	1.2865430
17	H	2.0656590	-0.0895280	2.2704350
18	C	3.2453820	1.5047190	1.4954940
19	H	4.1988960	1.1811260	1.9139660
20	H	3.4398210	2.0420520	0.5718190
21	H	2.7706620	2.1877590	2.2003450
22	H	2.1492080	1.4457560	-1.1378920
23	N	2.9712580	-0.8158190	0.5973230
24	C	2.8829790	-2.1637810	1.1521930
25	C	3.8688350	-0.7281250	-0.4237160
26	C	3.4422050	-3.0329080	0.0298110
27	H	3.4802100	-2.2350940	2.0671970
28	H	1.8497220	-2.4120370	1.4005950
29	C	4.4464050	-2.1091230	-0.6476970
30	O	4.1655970	0.2870840	-1.0366330
31	H	3.8749580	-3.9596660	0.3986120
32	H	2.6394340	-3.2774010	-0.6673780
33	H	5.4211800	-2.1361970	-0.1527610
34	H	4.5990080	-2.2969430	-1.7075840
35	C	-2.6866260	-0.9814790	0.6078800
36	C	-1.9696860	0.2874100	0.2058570
37	C	-2.0989170	1.1578640	1.2477370
38	C	-2.5925890	0.4074440	2.4042950
39	O	-2.8345560	-0.8803370	1.9965710
40	H	-1.8256070	0.5404100	-0.8336800
41	H	-1.8365850	2.2029690	1.2929510
42	O	-2.7639980	0.7495520	3.5423450
43	O	-4.0082160	-1.0024040	0.0824690
44	C	-4.1264250	-1.2944900	-1.2294490
45	C	-5.5566730	-1.3714380	-1.6473560
46	H	-5.6170080	-1.5593870	-2.7145370
47	H	-6.0514810	-2.1728360	-1.0986980
48	H	-6.0637090	-0.4394450	-1.3994990
49	O	-3.1623980	-1.4642690	-1.9339930
50	H	-2.1737740	-1.9061030	0.3447190

NVP-2a-NVP radical

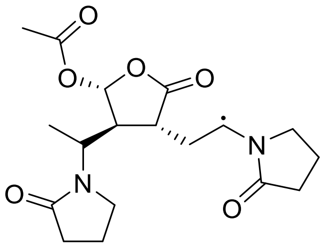

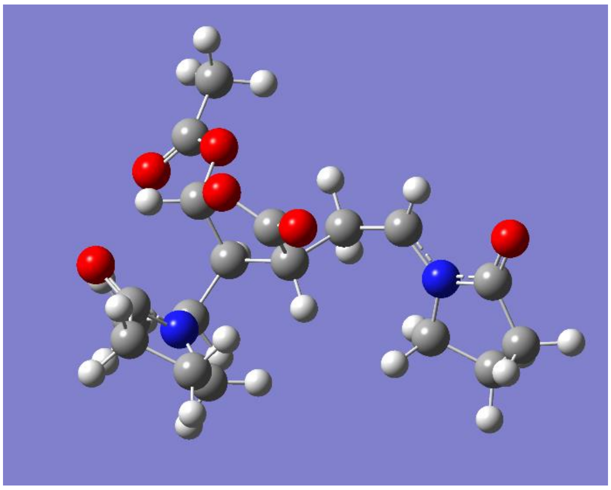

-1260.574628

Tag	Symbol	X	Y	Z
1	C	-1.8930590	0.9973840	0.4614180
2	C	-1.0701400	0.5229300	-0.7393080
3	C	0.2797570	0.2218940	-0.0935560
4	C	-0.1061130	-0.1343830	1.3199500
5	O	-1.3375520	0.3683910	1.5886210
6	H	-1.0084790	1.3247520	-1.4770970
7	H	0.7804320	-0.6298750	-0.5532700
8	O	0.5204610	-0.7349860	2.1479350
9	O	-1.7325850	2.3961880	0.6893170
10	C	-1.6575100	-0.7173540	-1.4277130
11	H	-0.9193220	-1.0093060	-2.1800520
12	C	-2.9643210	-0.4281300	-2.1521760
13	H	-3.3473650	-1.3409770	-2.6105730
14	H	-3.7265960	-0.0323300	-1.4868670
15	H	-2.7811880	0.3033280	-2.9399160
16	H	-2.9496890	0.7529140	0.4100330
17	N	-1.7286600	-1.8662540	-0.5211210
18	C	-0.8578800	-3.0216670	-0.7278350
19	C	-2.7735410	-2.1582110	0.3055150
20	C	-1.1187140	-3.8690900	0.5124370
21	H	-1.1391560	-3.5467470	-1.6475370
22	H	0.1834760	-2.7122140	-0.8174910
23	C	-2.5797590	-3.5685980	0.8199440
24	O	-3.7125270	-1.4209140	0.5664940
25	H	-0.9109290	-4.9236550	0.3477130
26	H	-0.4887760	-3.5130890	1.3288780
27	H	-3.2544670	-4.2140140	0.2503400
28	H	-2.8526250	-3.6377840	1.8701770
29	C	1.2481740	1.4377860	-0.0935300
30	H	1.3629800	1.7373210	-1.1395950
31	H	0.7615000	2.2682080	0.4197260
32	C	2.5600500	1.1849640	0.5438540
33	N	3.5591650	0.4923160	-0.0906230
34	C	3.4996540	-0.0137500	-1.4591320
35	C	4.7272610	0.1328840	0.5471640
36	C	4.9624580	-0.3159160	-1.7797500
37	H	2.8839140	-0.9171320	-1.4977780
38	H	3.0681180	0.7293330	-2.1274330
39	C	5.5559610	-0.6736090	-0.4225650
40	O	4.9905370	0.4182760	1.7043400
41	H	5.0622770	-1.1039630	-2.5223340
42	H	5.4410330	0.5844140	-2.1658720
43	H	5.4167150	-1.7322660	-0.1848430
44	H	6.6133760	-0.4424760	-0.3154830
45	H	2.7546590	1.4098980	1.5798830
46	C	-2.4683800	3.2110570	-0.0864790
47	O	-3.2039850	2.7907340	-0.9480760
48	C	-2.2459350	4.6472950	0.2561680
49	H	-1.1927580	4.8950360	0.1222440
50	H	-2.8597990	5.2771690	-0.3798070
51	H	-2.4934350	4.8165930	1.3036710

Transition state for addition of NVP-2a radical to NVP

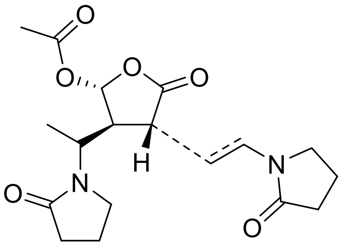

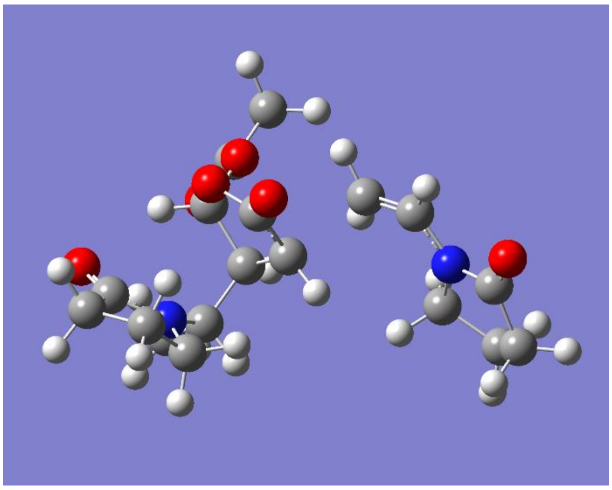

-1260.541455

Tag	Symbol	X	Y	Z
1	C	1.5948080	1.1665800	-0.4948690
2	C	0.7393840	0.4828880	0.5800170
3	C	-0.2040280	-0.2896320	-0.2789850
4	C	0.4513250	-0.5060640	-1.5491700
5	O	1.5256320	0.3521470	-1.6318060
6	H	0.2580790	1.2354180	1.2096790
7	H	-0.8711360	-1.0407760	0.1163750
8	O	0.1875140	-1.2542320	-2.4616950
9	O	1.0179010	2.4133710	-0.8889060
10	C	1.5689410	-0.4376530	1.5037940
11	H	0.8436970	-1.0109160	2.0888470
12	C	2.4277670	0.3590440	2.4752940
13	H	3.0490010	-0.3131330	3.0682900
14	H	3.0748490	1.0671240	1.9659260
15	H	1.7742930	0.9131430	3.1501490
16	H	2.6356060	1.3136550	-0.2177440
17	N	2.3084180	-1.4383070	0.7396420
18	C	1.7948480	-2.7998670	0.6224660
19	C	3.5697260	-1.3130740	0.2450680
20	C	2.6640320	-3.3990350	-0.4780960
21	H	1.9112650	-3.3306480	1.5737480
22	H	0.7356010	-2.7905900	0.3635990
23	C	3.9859230	-2.6642800	-0.2984850
24	O	4.2548290	-0.2996740	0.2513730
25	H	2.7454420	-4.4808780	-0.4036170
26	H	2.2327690	-3.1474110	-1.4485800
27	H	4.6153190	-3.1404830	0.4586980
28	H	4.5741310	-2.5496100	-1.2056220
29	C	-1.7852030	1.1118380	-0.9496840
30	H	-1.6513500	1.6697210	-0.0310610
31	H	-1.2727600	1.4923890	-1.8214310
32	C	-2.8955380	0.3358680	-1.1405330
33	N	-3.7007910	-0.0863090	-0.1138710
34	C	-3.5229330	0.3076520	1.2772650
35	C	-4.6993340	-1.0299260	-0.2840730
36	C	-4.8191800	-0.1627520	1.9386360
37	H	-2.6379150	-0.1844920	1.6927770
38	H	-3.3828120	1.3854190	1.3526200
39	C	-5.2612990	-1.3430720	1.0803180
40	O	-5.0103640	-1.5122500	-1.3540450
41	H	-4.6736420	-0.4182970	2.9849420
42	H	-5.5614370	0.6331470	1.8804480
43	H	-4.8040440	-2.2797880	1.4106810
44	H	-6.3368600	-1.4943920	1.0319270
45	H	-3.1486290	-0.0870730	-2.1035080
46	C	1.2011640	3.4542340	-0.0630390
47	O	1.8261300	3.3642530	0.9679710
48	C	0.5399410	4.6886470	-0.5841540
49	H	-0.5226370	4.4964350	-0.7371980
50	H	0.6758750	5.5058870	0.1170600
51	H	0.9687100	4.9511600	-1.5511690
